# Divergent Enzymatic Assembly of a Comprehensive 64‐Membered IgG *N*‐Glycan Library for Functional Glycomics

**DOI:** 10.1002/advs.202303832

**Published:** 2023-08-26

**Authors:** Wenjing Ma, Zhuojia Xu, Yuhan Jiang, Jialin Liu, Dandan Xu, Wei Huang, Tiehai Li

**Affiliations:** ^1^ State Key Laboratory of Chemical Biology Shanghai Institute of Materia Medica Chinese Academy of Sciences Shanghai 201203 China; ^2^ University of Chinese Academy of Sciences Beijing 100049 China; ^3^ School of Pharmaceutical Science and Technology Hangzhou Institute of Advanced Study Hangzhou 310024 China

**Keywords:** carbohydrates, enzyme‐catalyzed synthesis, functional glycomics, glycan microarray, IgG *N*‐glycan library

## Abstract

*N*‐Glycosylation, a main post‐translational modification of Immunoglobulin G (IgG), plays a significant role in modulating the immune functions of IgG. However, the precise function elucidation of IgG *N*‐glycosylation remains impeded due to the obstacles in obtaining comprehensive and well‐defined *N*‐glycans. Here, an easy‐to‐implement divergent approach is described to synthesize a 64‐membered IgG *N*‐glycan library covering all possible biantennary and bisected *N*‐glycans by reprogramming biosynthetic assembly lines based on the inherent branch selectivity and substrate specificity of enzymes. The unique binding specificities of 64 *N*‐glycans with different proteins are deciphered by glycan microarray technology. This unprecedented collection of synthetic IgG *N*‐glycans can serve as standards for *N*‐glycan structure identification in complex biological samples and the microarray data enrich *N*‐glycan glycomics to facilitate biomedical applications.

## Graphical abstract

A comprehensive 64‐Membered IgG *N*‐glycan library covering all possible biantennary and bisected *N*‐glycans is assembled through an easy‐to‐implement enzymatic approach. The key feature is that divergent enzymatic synthesis of *N*‐glycans is accomplished via reprogramming biosynthetic assembly lines based on inherent branch selectivity and substrate specificity of enzymes. Unique binding specificities of *N*‐glycans with different proteins are deciphered by glycan microarray technology.

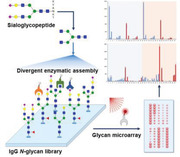

## 1 Introduction

Immunoglobulin G, one of the most common antibodies presented primarily in the blood and tissues, plays a significant role in physiological and pathological activities. It has been discovered that IgG comprises a conserved *N*‐glycosylation site at Asn297 in the Fc domain and 15–25% *N*‐glycans in the Fab region, which can remarkably alter and modulate their biofunctions through the synergistic effects of glycan structures and intrinsic peptide sequence.^[^ ^1^ ^]^ Recent studies have shown that alteration of IgG Fc glycosylation can modulate its effector functions and variations of IgG *N*‐glycans are distinctly reflected in diseases.^[^ ^2^ ^]^ Sialylation of Fc domain on IgG with immune complex can enhance the anti‐inflammatory activity.^[^ ^3^ ^]^ Removal of the core fucose on Fc domain can reinforce the antibody‐dependent cell cytotoxicity (ADCC) by specifically increasing FcγRIIIa binding,^[^ ^4^ ^]^ and bisecting GlcNAc moiety can also generate improved ADCC due to the steric hindrance.^[^ ^5^ ^]^ Through glycoengineering technologies, two FDA‐approved defucosylated antibodies named Mogamulizumab and Obinutuzumab have been produced and clinically used. Disialylated glycans on IgG Fab domain are also found to influence the antigen binding process through dynamic simulation analysis on Fab crystal structure and the Fab glycosylation can affect the activation of B cells possibly due to the interactions of sialic acid‐modified Fab glycans with immune‐associated lectins like galectin‐9 or siglec‐10.^[^ ^6^ ^]^ Meanwhile, an increased sialylation, bisection, and fucosylation of Fab *N*‐glycans were observed in patients with multiple myeloma.^[^ ^7^ ^]^ Consequently, structurally well‐defined IgG *N*‐glycans with high purity and sufficient quantity are urgently needed for elucidating the biofunctions of IgG *N*‐glycosylation and developing diagnostic tools as well as glycoengineered antibody‐based drugs. These compounds can be utilized as standards to conduct *N*‐glycan identification in complex biological samples and as probes to investigate their functional mechanisms at molecular level, also as a precursor for preparations of homogenous glycoengineered antibody.^[^ ^8^ ^]^

**Figure 1:**
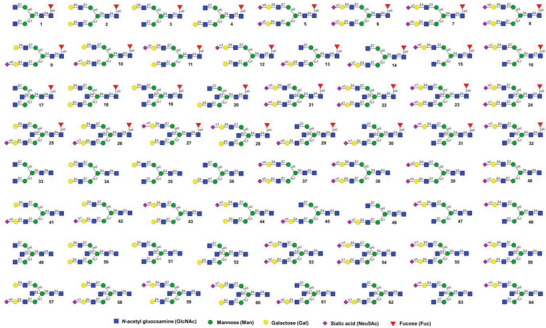
Synthetic 64‐membered IgG *N*‐glycan library containing all possible biantennary and bisected *N*‐glycans.

The structural complexity and heterogeneity of IgG *N*‐glycans make it difficult to isolate adequate and structurally well‐defined glycans from serum or tissue samples for biological function studies. Although great efforts have been devoted to preparing complex *N*‐glycans with asymmetrical or symmetrical structures through chemical and chemoenzymatic methods,^[^ ^9^ ^]^ the easy‐to‐implement systematic synthesis of comprehensive IgG *N*‐glycans has not yet been reported. The exact structural elucidations and precise functional investigations of IgG *N*‐glycosylation are still hampered due to the absence of an extensive and systematic *N*‐glycan library with well‐defined structures. To address this problem, we here report an easy‐to‐implement divergent approach to enzymatically assemble a comprehensive and unprecedented 64‐membered IgG *N*‐glycan library containing all possible biantennary and bisected *N*‐glycans with or without core fucosylation from a readily accessible precursor (**Figure** **1**). The key feature of this approach is that various asymmetrical *N*‐glycans were enzymatically prepared by reprogramming biosynthetic assembly lines based on the inherent branch selectivity and substrate specificity of enzymes. More importantly, regioselective sialylation of *N*‐glycans was achieved by differentiating the branches with specially designed assembly sequences to generate all terminal sialic acid linkage isomers of *N*‐glycans on IgG. In addition, the synthetic 64 *N*‐glycans were subjected to a systematic exploration into the binding specificities with plant lectins and immune‐associated lectins using glycan microarray technology, thereby providing abundant information for the development of diagnostic reagents and antibody‐based drugs.

## 2 Results and Discussion

### 2.1 Divergent Enzymatic Synthesis of Core‐Fucosylated Biantennary *N*‐Glycans

**Scheme 1:**
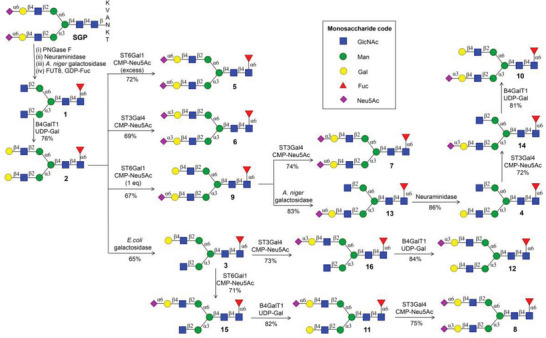
Divergent enzymatic synthesis of core‐fucosylated biantennary *N*‐glycans 1–16.

The core‐fucosylated *N*‐glycans **1–16** were synthesized by a divergent enzymatic approach. The glycan **1** was prepared by a four‐step procedure starting with a sialoglycopeptide (SGP) isolated from egg yolk or its powder.^[^ ^10^ ^]^ Removal of the peptide moiety of SGP using PNGase F afforded disialylated *N*‐glycan **37** (Scheme S1A, Supporting Information), followed by the cleavage of terminal sialic acids with *α*2‐3,6,8‐neuraminidase to provide glycan **34**. Compound **34** was then treated with galactosidase from *Aspergillus niger* (*A. niger*) for the removal of two galactoses to give glycan **33**. Core fucosylation of **33** with guanosine 5′‐diphospho‐β‐l‐fucose (GDP‐Fuc) and *α*1,6‐fucosyltransferase 8 (FUT8) in the presence of calf intestine alkaline phosphatase (CIAP), which can hydrolyze the generated nucleotide pyrophosphates to drive the completion of enzyme‐mediated reaction, smoothly afforded glycan **1** (**Scheme** **1**). Galactosylation of GlcNAc moieties of **1** with β1,4‐galactosyltransferase 1 (B4GalT1) and uridine‐5′‐diphosphogalactose (UDP‐Gal) provided a bis‐galactosylated glycan **2**. Sialylation of terminal two galactose residues of **2** using α2,6‐sialyltransferase (ST6Gal1) and α2,3‐sialyltransferase (ST3Gal4) in presence of excess cytidine‐5′‐monophospho‐*N*‐acetylneuraminic acid (CMP‐Neu5Ac, 3 equiv) afforded disialylated glycan isomers **5** and **6**, respectively. The enzymatic reaction process can be easily monitored by electrospray ionization−mass spectrometry (ESI‐MS). If any starting glycan substrate was detected, additional enzymes and sugar nucleotides were added until the homogeneous product was formed. The resulting product can be readily purified through size‐exclusion chromatography using Bio‐Gel P‐4.

With glycan **2** in hand, we turned our attention to divergent enzymatic synthesis of asymmetrical *N*‐glycans by exploring inherent branch selectivity of ST6Gal1 and *E. coli* galactosidase as well as differentiating the branches with specifically designed glycosylation sequences for regioselective sialylation. First, selective α2,6‐sialylation of **2** with CMP‐Neu5Ac (1 equiv) and ST6Gal1 that preferred the bottom antenna provided asymmetrical glycan **9**.^[^ ^11^ ^]^ And selective removal of the galactose residue at bottom antenna of **2** was achieved by galactosidase from *E. coli* to afford asymmetrical glycan **3**.^[^ ^12^ ^]^ After the rapid purification using Bio‐Gel P‐4 size‐exclusion chromatography, the desired glycans **9** and **3** were obtained with minor contamination of unreacted starting materials and byproducts that can be easily removed by semi‐preparative high performance liquid chromatography (HPLC) with a hydrophilic interaction chromatography (HILIC) column (Table S2, Supporting Information) to afford highly pure compounds. Given the fact that *α*2,6‐sialoside can block further enzymatic modifications, treatment of **9** with ST3Gal4 and galactosidase from *A. niger* for corresponding α2,3‐sialylation and removal of terminal Gal residue at upper antennae provided glycans **7** and **13**, respectively. Compound **13** was further treated with α2‐3,6,8‐neuraminidase for efficient cleavage of sialoside to give glycan **4** as the isomer of **3**. Glycan **4** was characterized with terminal GlcNAc and LacNAc moiety at upper antenna and bottom antenna, respectively. Many glycosyltransferases modify LacNAc but not GlcNAc moiety,^[^ ^13^ ^]^ thus α2,3‐sialylation of **4** at bottom antenna with ST3Gal4 and CMP‐Neu5Ac afforded glycan **14** as the isomer of **13**. Further galactosylation of **14** with B4GalT1 and UDP‐Gal generated glycan **10** as the isomer of **9**. Next, following a similar enzymatic glycosylation sequence, glycan **3** was successively treated with ST3Gal4 and B4GalT1 to give glycans **16** and **12**. In parallel, α2,6‐sialylation of compound **3** with ST6Gal1 and CMP‐Neu5Ac afforded glycan **15**, which was further galactosylated by B4GalT1 and UDP‐Gal to provide glycan **11**. Subsequently, α2,3‐sialylation of LacNAc moiety at the bottom antenna with ST3Gal4 and CMP‐Neu5Ac afforded disialylated glycan **8** as the isomers of **5–7**.

### 2.2 Divergent Enzymatic Synthesis of Bisected *N*‐Glycans and Core‐Unmodified *N*‐Glycans

**Scheme 2:**
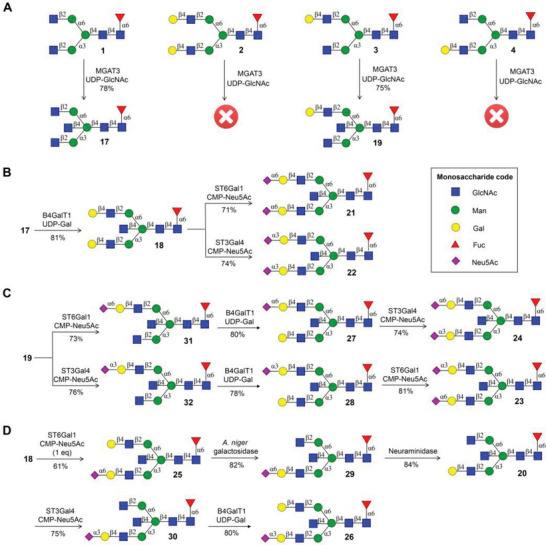
Divergent enzymatic synthesis of bisected *N*‐glycans 17–32 bearing core fucose. A) The substrate specificity study of MGAT3 and enzymatic synthesis bisected *N*‐glycans 17 and 19. B) Enzymatic synthesis of symmetrical disialylated isomers 21 and 22. C) Enzymatic synthesis of asymmetrical disialylated isomers 23 and 24. D) Enzymatic synthesis of asymmetrical monosialylated isomers 25 and 26, isomers 29 and 30.

First, attention was focused on the enzyme‐mediated preparation of various bisected *N*‐glycans with core fucose. β1,4‐Mannosyl‐glycoprotein β1,4‐*N*‐acetylglucosaminyltransferase (MGAT3) is responsible for catalyzing the addition of bisecting GlcNAc onto *N*‐glycans.^[^ ^14^ ^]^ Having glycans **1–4** in hand, we investigated the substrate specificity of MGAT3 (**Scheme** **2**A), which indicated that galactosylation of the GlcNAc residue at the bottom antenna (glycans **2** and **4**) prevented the activity of MGAT3. Bisected *N*‐glycans **17** and **19** were readily obtained by the treatment of **1** and **3** with MGAT3 in the presence of uridine 5′‐diphospho‐*N*‐acetylglucosamine (UDP‐GlcNAc), respectively. The terminal two GlcNAc residues of **17** were converted into corresponding LacNAc motifs using B4GalT1 and UDP‐Gal (1 equiv per Gal) to afford glycan **18** (Scheme 2B). It is important to mention that a high concentration of B4GalT1 and an excess of UDP‐Gal would result in the galactosylation of bisecting GlcNAc. Sialylation of both LacNAc moieties of **18** with ST6Gal1 and ST3Gal4 in the presence of CMP‐Neu5Ac (3 equiv) gave disialylated isomers **21** and **22**, respectively. To obtain asymmetrical disialylated isomers **23** and **24**, the LacNAc moiety of **19** was first mono‐sialylated by ST6Gal1 and ST3Gal4 in the presence of CMP‐Neu5Ac (1.5 equiv) to afford glycan **31** and **32**, respectively (Scheme 2C). Subsequent selective extension of terminal GlcNAc residue of **31** at the bottom antenna with two sequential enzyme‐catalyzed reactions using B4GalT1 and ST3Gal4 provided glycans **27** and **24**. Following similar enzymatic glycosylation conditions, compound **32** was successively converted into glycans **28** and **23**. To diversity‐oriented synthesize asymmetrical bisected *N*‐glycans (Scheme 2D), selective α2,6‐sialylation of the bottom antenna LacNAc moiety of **18** using ST6Gal1 and CMP‐Neu5Ac (1 equiv) was achieved to afford glycan **25**. Treatment of **25** with galactosidase from *A. niger* for the cleavage of terminal galactose residue at the upper antenna provided glycan **29**, which was further treated by α2‐3,6,8‐neuraminidase to remove terminal Neu5Ac residue to generate glycan **20** as the isomer of **19**. The resulting LacNAc moiety of **20** at bottom antenna was α2,3‐sialylated by ST3Gal4 and CMP‐Neu5Ac to afford glycan **30**. The terminal GlcNAc residue of **30** at the upper antenna was selectively galactosylated by B4GalT1 and UDP‐Gal (1 equiv) to give glycan **26** as the isomer of **25** without affecting bisecting GlcNAc. Additionally, to further diversify the IgG *N*‐glycan library, the core‐fucosylated *N*‐glycans **1–32** were treated with a robust fucosidase FucA1 for the removal of fucoside to afford core‐unmodified *N*‐glycans **33–64**, respectively.^[^ ^9k^ ^]^

### 2.3 Structural Characterization of the Representative Bisected *N*‐Glycans

**Figure 2:**
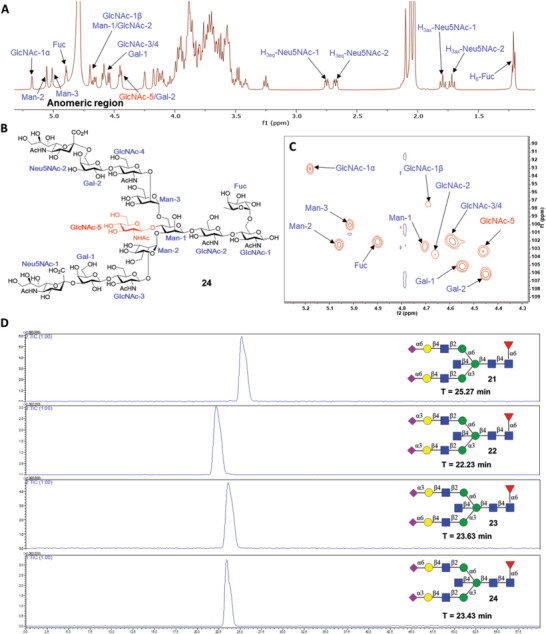
NMR and LC‐MS analysis of complex bisected *N*‐glycans. A) Anomeric proton signals, unique signals of core fucose residue, and sialic acid residue in ^1^H NMR spectra of 24. B) Chemical structure of 24. C) Anomeric signals in ^1^H‐^13^C HSQC spectra of 24. D) LC‐MS analysis of four disialylated *N*‐glycans 21–24.

The structural identifications of all synthesized *N*‐glycans were confirmed by detailed analysis of NMR and ESI‐MS spectroscopy. Herein, the representative bisected complex *N*‐glycan **24**, which contained core fucose and different sialic acid linkages (α2,6‐Neu5Ac and α2,3‐Neu5Ac) at the terminal, was fully characterized (Page S37, Supporting Information) by 1D and 2D NMR spectra shown in **Figure** **2A**‐C. In ^1^H NMR spectra, anomeric proton signals resonate from 4.45 to 5.18 ppm. Despite the highly overlapped signals, these signals can be successfully assigned by 2D ^1^H‐^13^C HSQC experiment (Figure 2C). The anomeric signal of the bisecting GlcNAc‐5 labeled in red was unique, its chemical shift (*δ* = 4.46 ppm) is obviously smaller than anomeric chemical shifts (*δ* = 4.56–5.18 ppm) of other GlcNAc residues. The characteristic signals at 1.21 ppm corresponded to methyl of core fucose residue. Interestingly, the terminal α2,3‐ and 2,6‐linked Neu5Ac residues can be readily distinguished by the chemical shifts of H‐3. The H‐3 signals (*δ*_H3ax_ = 1.84 and *δ*_H3eq_ = 2.75 ppm) of α2,3‐linked Neu5Ac displayed the lower field values than ones (*δ*_H3ax_ = 1.72 and *δ*_H3eq_ = 2.68 ppm) of α2,6‐linked Neu5Ac, respectively. Additionally, four disialylated isomeric *N*‐glycans **21**–**24** exhibited different retention times in HILIC column by LC‐MS analysis, demonstrating that all these synthesized *N*‐glycans could be utilized as standards to conduct *N*‐glycan identification in complex biological samples.

### 2.4 Glycan Microarray Analysis

Glycan microarray technologies are widely used for the rapid analysis of carbohydrate‐protein interactions, which makes it possible to probe the binding specificities of *N*‐glycans in a high‐throughput manner. Recently, Paulson and coworkers chemoenzymatically synthesized various *N*/*O*‐glycans with extended LacNAc repeats for glycan microarray profiles of receptor specificities of influenza virus and middle east respiratory syndrome coronavirus.^[^ ^15^ ^]^ Symmetrical asparagine (Asn)‐linked *N*‐glycans containing bi‐, tri‐, and tetra‐antennary structures were enzymatically synthesized and printed on glass slides by Cummings and coworkers for exploration into the unique *N*‐glycan interactions with lectins and influenza viruses.^[^ ^9s^ ^]^ Additionally, a neoglycoprotein array enabling a high‐affinity multivalent screening was developed by Gildersleeve and coworkers to decipher the recognitions between *N*‐glycans and proteins such as plant lectin, bacterial‐related lectin, and immune‐associated lectin.^[^ ^16^ ^]^ To better understand the binding specificities of diverse *N*‐glycans on IgG, the well‐characterized compounds **1**–**64** in this study were attached with 2‐[(methylamino)oxy]ethanamine spacer,^[^ ^17^ ^]^ and the resulting derivatives were printed on *N*‐hydroxysuccinimide (NHS)–activated glass slides for deciphering the interactions with glycan‐binding proteins using glycan microarray technology.^[^ ^18^ ^]^

#### 2.4.1 Glycan‐Binding Specificities of Plant Lectins

**Figure 3:**
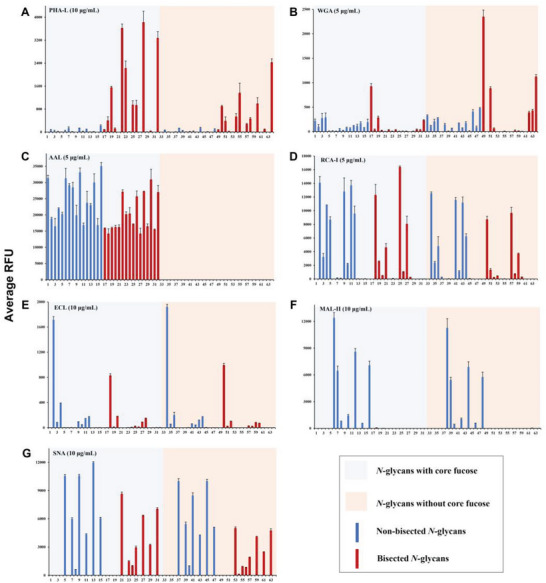
Microarray results of synthetic *N*‐glycan library at 100 µm with (A) PHA‐L (10 µg mL^−1^), B) WGA (5 µg mL^−1^), C) AAL (5 µg mL^−1^), D) RCA‐I (5 µg mL^−1^), E) ECL (10 µg mL^−1^), F) MAL‐II (10 µg mL^−1^), and G) SNA (10 µg mL^−1^). The average relative fluorescence unit (RFU) was calculated for four independent replicates on the glycoarrays after removing the highest and lowest signals. The error bars represent the standard deviation (SD) among the values of four replicate spots. The RFU and SD of data are provided in Table S1 (Supporting Information).

First, we evaluated the bindings of 7 plant lectins with *N*‐glycans and observed uniquely and remarkably distinct binding patterns. *Phaseolus vulgaris* leucoagglutinin (PHA‐L), known to bind β1,6‐GlcNAc branched triantennary and tetraantennary *N*‐glycans,^[^ ^9s^ ^]^ displayed a distinct binding specificity for bisected *N*‐ glycans in our arrays (**Figure** **3**A). Compared with non‐bisected counterparts, bisected *N*‐glycans were well recognized by PHA‐L. Additionally, α2,3‐sialylated and core fucosylated bisected *N*‐glycans displayed strong bonding. Wheat germ agglutinin (WGA) is reported to bind terminal GlcNAc and LacNAc.^[^ ^19^ ^]^ In our tests, WGA was found to strongly bind bisected *N*‐glycans **17**, **49**, **51**, and **64**, especially for the non‐fucosylated compounds (Figure 3B), which is consistent with the previous report.^[^ ^9s^ ^]^ Additionally, fucose‐specific lectin *Aleuria aurantia* lectin (AAL) was assayed. Glycans containing core fucose were all strongly bonded by AAL (RFU from ≈15 000 to 35 000), which was not affected by bisected GlcNAc or other glycosylation alterations (Figure 3C). Two galactose‐specific lectins *Ricinus communis* agglutinin (RCA‐I) and *Erythrina cristagalli* lectin (ECL) were also evaluated in our arrays. RCA‐I exhibited a powerful recognition of all terminal β1,4‐galactosylated *N*‐glycans and their derivatives with α2,6‐sialylation (Figure 3D, e.g., compounds **9**, **11**, **25,** and **27**). Differently, ECL mainly presented an exclusive binding to the terminal LacNAc moiety (Figure 3E) that is in accordance with previous observations.^[^ ^9u^ ^]^ *Maackia amurensis* lectin II (MAL‐II) and *Sambucus nigra* agglutinin (SNA) respectively recognized *N*‐glycans with α2,3‐sialylation and α2,6‐sialylation (Figure 3F,G), which was in accordance with prior studies. ^[^ ^9s,u^ ^]^ Notably, MAL‐II exclusively recognized non‐bisected *N*‐glycans, indicating that the addition of bisecting GlcNAc may block the bindings due to the conformation change. In contrast, the bisecting GlcNAc did not influence the recognition of SNA to α2,6‐sialylated *N*‐glycans. Besides, MAL‐II exhibited powerful binding to α2,3‐sialylated glycans on the α1,6‐Man branch, while SNA prefers to α2,6‐sialylated glycans on α1,3‐Man branch.

#### 2.4.2 Glycan‐Binding Specificities of Immune‐Associated Lectins

**Figure 4:**
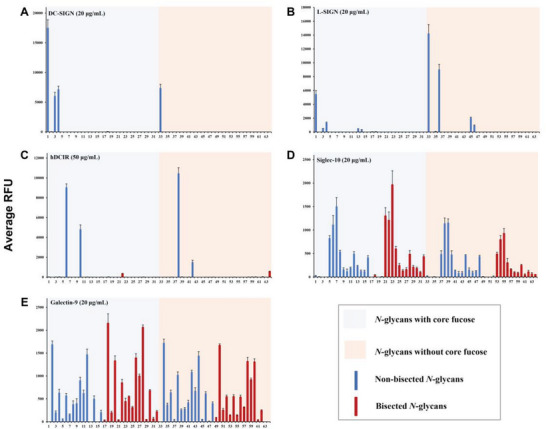
Microarray results of synthetic glycan library at 100 µm with A) DC‐SIGN (20 µg mL^−1^), B) L‐SIGN (20 µg mL^−1^), C) human DCIR (50 µg mL^−1^), D) Siglec‐10 (20 µg mL^−1^), and E) Galectin‐9 (20 µg mL^−1^). The average relative fluorescence unit (RFU) was calculated for four independent replicates on the glycoarrays after removing the highest and lowest signals. The error bars represent the standard deviation (SD) among the values of four replicate spots. The RFU and SD of data are provided in Table S1 (Supporting Information).

The bindings of five representative immune‐associated lectins with *N*‐glycans were investigated. Dendritic cell‐specific ICAM‐3‐grabbing nonintegrin (DC‐SIGN) and liver/lymph node‐specific ICAM‐3 grabbing non‐integrin (L‐SIGN), are two trans‐membrane C‐type lectin receptors respectively expressed by dendritic cells and type II alveolar cells of human lungs, which mediate immune responses and participate in viral recognition and microbial pathogens presentation.^[^ ^20^ ^]^ It is reported that DC‐SIGN binds high‐mannose and branched‐fucosylated structures,^[^ ^21^ ^]^ while the binding specificity of L‐SIGN with *N*‐glycans has not yet been systematically explored by glycoarray technology. In our arrays, DC‐SIGN and L‐SIGN both presented preference toward the *N*‐glycan core heptasaccharide structure with or without fucosylation (**Figure** **4**A,B, e.g., compounds **1** and **33**). It is worth noting that core fucosylated *N*‐glycan were preferably recognized by DC‐SIGN, whereas L‐SIGN mainly bound defucosylated glycans.

Human dendritic cell immunoreceptor (DCIR), an inhibitory receptor that is expressed on antigen‐presenting cells including B cells, monocytes, and myeloid dendritic cells (DCs), involves in vital activities in immune responses and autoimmune diseases.^[^ ^22^ ^]^ DCIR was reported to bind fucosylated and mannose‐modified oligosaccharides,^[^ ^22c^ ^]^ and has been proven to recognize pathogenic organisms and participate in the attachment for human immunodeficiency virus (HIV) in DCs,^[^ ^22b^ ^]^ while the precise structures of *N*‐glycan ligands are still ambiguous. We tested the recognition specificities of hDCIR on our 64‐membered *N*‐glycans (Figure 4C). Unlike the mouse dendritic cell inhibitory receptor 2 (mDCIR2), which is the only bisected *N*‐glycan‐specific animal lectin discovered,^[^ ^23^ ^]^ hDCIR exhibited vigorous binding to di‐α2,3‐sialylated non‐bisected glycans (e.g., compounds **6** and **38**).

To better elucidate the *N*‐glycosylation functions of IgG, we tested the bindings of four type I Fcγ receptors, and two lectins (Siglec‐10 and Galectin‐9) that mediate the activation of B cells possibly due to the interactions with Fab glycans.^[^ ^6^ ^]^ Unfortunately, all synthetic *N*‐glycans displayed no binding to four type I Fcγ receptors, indicating that the protein segment is necessary for IgG bindings to Fcγ receptors. Siglec‐10 is acknowledged to be more inclined to bind glycans containing α2,6‐sialylated LacNAc motifs.^[^ ^24^ ^]^ The binding profiles of asymmetric *N*‐glycans and isomers are sophisticated, of which *N*‐glycans with α2,6‐sialylated LacNAc on α1,3‐Man‐branch and α2,3‐sialylated LacNAc on α1,6‐Man branch represented a relative stronger recognition specificity (Figure 4D, e.g., compounds **23** and **7**). Galectin‐9 is a β‐galactoside‐binding lectin that plays essential roles in mediating B cell activation and altering the progression of infectious diseases.^[^ ^25^ ^]^ Both galactose and LacNAc moieties are the common domains that interact with Galectin‐9 and other galectins. In our tests, a broader tolerance to LacNAc‐containing *N*‐glycans of Galetin‐9 was discovered, and nearly all LacNAc‐containing glycans were bound, of which *N*‐glycans with α2,3‐Neu5Ac were preferred over α2,6‐sialylated ones (Figure 4E, e.g., compound **5** vs **6**, compounds **21** vs **22**).

All the results are shown as average relative fluorescence unit (RFU) that was calculated for four independent replicates on the glycoarrays after removing the highest and lowest signals. The error bars represent the standard deviation (SD) among the values of four replicate spots. The RFU and SD of data are provided in Table S1 (Supporting Information).

## 3 Conclusion

In summary, we developed an easy‐to‐implement approach to efficiently synthesize a comprehensive and systemic IgG *N*‐glycan library containing all possible biantennary and bisected *N*‐glycans for functional glycomics studies by glycan microarray technology. Key features include divergent enzymatic assembly of various *N*‐glycans via reprogramming biosynthetic assembly lines based on the inherent branch selectivity and substrate specificity of enzymes, as well as regioselective sialylation of *N*‐glycans by differentiating the branches with specially designed assembly sequences to generate all terminal sialic acid linkage‐isomers. The binding studies of 64 *N*‐glycans with different proteins demonstrated that the architecture and topology of complex *N*‐glycan affected recognition. This work enriches the synthesis space of *N*‐glycan, provides invaluable standards for *N*‐glycan structure identification in complex biological samples, and offers opportunities for precise functional investigations of IgG *N*‐glycosylation to optimize the performances of glycoengineered therapeutic antibodies.^[^ ^26^ ^]^

## Conflict of Interest

The authors declare no conflict of interest.

## Supporting information

Supporting Information

Supplemental Table 1

## Data Availability

The data that support the findings of this study are available in the supplementary material of this article.
