# Supplementary material for: Divergent Enzymatic Assembly of a Comprehensive 64‐Membered IgG N‐Glycan Library for Functional Glycomics
Source: Adv Sci (Weinh). 2023 Aug 26;10(30):2303832. doi: 10.1002/advs.202303832 (PMC10602528; doi:10.1002/advs.202303832)
Supplement: Supplementary file 1 — Supporting Information [file ADVS-10-2303832-s002.pdf]

ADVANCED SCIENCE
Open Access

Supporting Information

for *Adv. Sci.*, DOI 10.1002/advs.202303832

Divergent Enzymatic Assembly of a Comprehensive 64-Membered IgG *N*-Glycan Library for Functional Glycomics

*Wenjing Ma, Zhuojia Xu, Yuhan Jiang, Jialin Liu, Dandan Xu, Wei Huang and Tiehai Li**

Table of Contents

1. General materials and methods……………………………………………………….……S2

2. Glycosyltransferases expression and purification ………………………………………S2

3. General protocols for enzymatic reactions ……………………...................................S3

4. Divergent enzymatic synthesis of core-fucosylated biantennary *N*-glycans.……………....S7

5. Divergent enzymatic synthesis of bisected *N*-glycans S8

6. LC-MS analysis and purification of complex *N*-glycans S9

7. General procedure for installing functional spacer to reducing terminal of glycan……..S12

8. Microarray procedure…………………………………………………..…...............S12

9. NMR nomenclature……………………...S14

10. NMR assignments and MS data………………………..S14

11. References ……………………………………………………………………..S78

12. NMR spectra ……………………………………………..………………………S79

1. General materials and methods

Chemical reagents were purchased from J&K Scientific Ltd. and TCI Shanghai, China. Uridine 5′-diphosphogalactose (UDP-Gal), uridine 5′-diphospho-*N*-acetylglucosamine (UDP-GlcNAc), guanosine 5′-diphospho-β-*L*-fucose (GDP-Fuc), and cytidine-5′-monophospho-N-acetylneuraminic acid (CMP-Neu5Ac) were purchased from BioChemSyn. β-Galactosidase (E-BGLAN) from *Aspergillus niger* (*A. niger*) was purchased from Megazyme. *E. coli* galactosidase (G5635) was purchased from Sigma-Aldrich. Calf intestine alkaline phosphatase (CIAP) and α2-3,6,8-neuraminidase were purchased from BioLabs® Inc.

Proton nuclear magnetic resonance (^{1}H NMR) spectra were recorded on a Bruker Avance 600 (at 600 MHz) spectrometer with cryogenically cooled

probe. Samples were dissolved into 99.9% deuterium oxide (D_2O). The reducing terminal *N*-acetylglucosamine (GlcNAc-1α H-1) of glycan was set to 5.18 ppm. Spectra were assigned using ^{1}H NMR, COSY, HSQC, TOCSY, and NOESY experiments. MALDI-MS data were recorded on an Autoflex II MALDI-TOF (Bruker Daltonics) system instrument using 2,5-dihydroxybenzoic acid (DHB) as matrix. Low-resolution mass spectrometry (LRMS) was measured on ESI apparatus using a Shimadzu LC-MS2020. Compound analysis was recorded on Shimadzu LC-MS2020-ESI with XBrigde® Amide 3.5 μm, 2.1 mm x 150 mm column (Waters). Compound purification was performed by size-exclusion chromatography with Bio-Gel P-4 (45-90 μm) column and Shimadzu LC-MS2020-ESI with XBridge® Amide 5 μm, 10 mm x 250 mm column (Waters).

2. Glycosyltransferases expression and purification

Glycosyltransferases FUT8, B4GalT1, MGAT3, ST6Gal1, ST3Gal4, PNGase F, and FucA1 were expressed and purified as described previously.[1] The catalytic domains of human glycosyltransferases were expressed as soluble, secreted fusion proteins by transient transfection of HEK293 suspension cultures. The coding regions were amplified from Mammalian Gene Collection clones, human tissue cDNAs, or generated by gene synthesis by a process that appended a tobacco etch virus (TEV) protease cleavage site to the NH_2-terminal end of the coding region and attL1 and attL2 Gateway adaptor sites were extended on the 5′ and 3′ terminal ends of the coding region during transfer to pDONR221 vector backbone. The pDONR221 clones were then recombined via LR clonase reaction into a custom Gateway adapted version of the pGEn2 mammalian expression vector to assemble a recombinant coding region comprised of

a 25 amino acid NH_2-terminal signal sequence from the *T. cruzi* lysosomal *α*-mannosidase followed by an 8xHis tag, 17 amino acid AviTag, "superfolder" GFP, the nine amino acid sequence encoded by attB1 recombination site, followed by the TEV protease cleavage site and the respective glycosyltransferase catalytic domain coding region.

Suspension culture HEK293 cells (Freestyle 293-F cells, Life Technologies, Grand Island, NY) were transfected as previously described[1a] and the culture supernatant was subjected to Ni^{2+}-NTA superflow chromatography (Qiagen, Valencia, CA). Enzyme preparations were eluted with 300 mM imidazole, concentrated by ultrafiltration, and subjected to gel filtration on a Superdex 75 column (GE Healthcare) preconditioned with a buffer containing 20 mM HEPES, pH 7.0, 100 mM NaCl, 10% glycerol, 0.05% sodium azide. Peak fractions were pooled and concentrated to ~1 mg/mL using an ultrafiltration pressure cell membrane (Millipore, Billerica, MA) with a 10 kDa molecular weight cutoff.

3. General protocols for enzymatic reactions

General procedure for the removal of peptide moiety of SGP using PNGase F

To a solution of a sialoglycopeptide (SGP, 5-50 mM) in sodium phosphate buffer (50 mM, pH 7.5) was added PNGase F (10 µg/µmol acceptor), and the mixture was incubated at 37 ^oC. Reaction progress was monitored by ESI-MS, if starting material remained after 16 h, another portion of PNGase F (5 µg/µmol acceptor) were added until no starting material could be detected. The reaction mixture was centrifuged, and the resulting supernatant was loaded on Bio-Gel P4 column (eluent: 0.1 M NH_4HCO_3). Product containing fractions were combined and lyophilized to afford the desired product as a white amorphous solid.

General procedure for the removal of terminal galactose using galactosidase from *A. niger*

To a solution of *N*-glycan (5-30 mM) in sodium acetate buffer solution (100 mM, pH 4.5) was added galactosidase from *A. niger* (500 U/mL), and the mixture was incubated at 37 ^oC. Reaction progress was monitored by MALDI-TOF MS or ESI-MS, if starting material remained after 16 h, another portion of galactosidase from *A. niger* (100 U/mL) were added until no starting material could be detected. The reaction mixture was centrifuged, and the resulting supernatant was loaded on Bio-Gel P4 column (eluent: 0.1 M NH_4HCO_3). Product containing fractions were combined and lyophilized to afford the desired product as a white amorphous solid.

General procedure for the removal of terminal Neu5Ac using α2-3,6,8 neuraminidase

To a solution of *N*-glycan (5-30 mM) in sodium acetate buffer solution (50 mM, pH 5.5) containing $CaCl_2$ (5 mM) was added α2-3,6,8 neuraminidase (2500 U/mL), and the mixture was incubated at 37 °C. Reaction progress was monitored by ESI-MS, if starting material remained after 16 h, another portion of neuraminidase was added until no starting material could be detected. Once the reaction was completed, the neuraminidase was inactivated at 65 °C for 10 min. The reaction mixture was centrifuged, and the resulting supernatant was loaded on Bio-Gel P4 column (eluent: 0.1 M NH_4HCO_3) to afford the desired product as a white amorphous solid.

General procedure for the installation of α1,6 Fuc using FUT8

To a solution of *N*-glycan acceptor (5-20 mM) and GDP-Fuc (1.2 eq per Fuc) in a Tris buffer solution (100 mM, pH 7.5) containing $MnCl_2$ (10 mM) and BSA (1% total volume, stock solution = 10 mg/mL) was added calf intestinal alkaline phosphatase (CIAP, 1% total volume, 1000 U/mL) and FUT8 (50 µg/µmol acceptor). The reaction mixture was incubated at 37 °C. Reaction progress was monitored by ESI-MS, if starting material remained after 18 h, another portion of FUT8 and GDP-Fuc (0.3 eq per Fuc) were added until no starting material could be detected. The reaction mixture was centrifuged, and the resulting supernatant was loaded on Bio-Gel P4 column (eluent: 0.1 M NH_4HCO_3). Product containing fractions were combined and lyophilized to afford the desired product as a white amorphous solid.

General procedure for the installation of β1,4 Gal using B4GalT1

To a solution of *N*-glycan acceptor (5-10 mM) and UDP-Gal (1.2 eq per Gal) in a Tris buffer solution (100 mM, pH 7.5) containing $MnCl_2$ (10 mM) and BSA (1% total volume, stock solution = 10 mg/mL) was added calf intestinal alkaline phosphatase (CIAP, 1% total volume, 1000 U/mL) and B4GalT1 (40 µg/µmol acceptor). The reaction mixture was incubated at 37 °C. Reaction progress was monitored by ESI-MS, if starting material remained after 16 h, another portion of B4GalT1 and UDP-Gal (0.3 eq per Gal) were added until no starting material could be detected. The reaction mixture was centrifuged, and the resulting supernatant was loaded on Bio-Gel P4 column (eluent: 0.1 M NH_4HCO_3). Product containing fractions were combined and lyophilized to afford the desired product as a white amorphous solid.

Procedure for the selective installation of α2,6 Neu5Ac using ST6Gal1

To a solution of *N*-glycan acceptor (5-10 mM) and CMP-Neu5Ac (1.0 eq) in a sodium cacodylate buffer solution (100 mM, pH 7.2) containing BSA (1% total volume, stock solution =

10 mg/mL) was added calf intestinal alkaline phosphatase (CIAP, 1% total volume, 1000 U/mL) and ST6Gal1 (80 μg/μmol acceptor), and the reaction mixture was incubated at 37 °C for 16 h. The reaction mixture was centrifuged, and the resulting supernatant was loaded on Bio-Gel P4 column (eluent: 0.1 M NH_4HCO_3). Product containing fractions were combined and lyophilized to afford the desired product as a white amorphous solid. The product containing impure side fractions were combined and lyophilized for further purification by HPLC.

General procedure for the installation of α2,6 Neu5Ac using ST6Gal1

To a solution of *N*-glycan acceptor (5-10 mM) and CMP-Neu5Ac (1.5 eq per Neu5Ac) in a sodium cacodylate buffer solution (100 mM, pH 7.2) containing BSA (1% total volume, stock solution = 10 mg/mL) was added calf intestinal alkaline phosphatase (CIAP, 1% total volume, 1000 U/mL) and ST6Gal1 (80 μg/μmol acceptor), and the reaction mixture was incubated at 37 °C. Reaction progress was monitored by ESI-MS, if starting material remained after 16 h, another portion of ST6Gal1 and CMP-Neu5Ac (0.5 eq per Neu5Ac) were added until no starting material could be detected. The reaction mixture was centrifuged, and the resulting supernatant was loaded on Bio-Gel P4 column (eluent: 0.1 M NH_4HCO_3). Product containing fractions were combined and lyophilized to afford the desired product as a white amorphous solid.

General procedure for the installation of α2,3 Neu5Ac using ST3Gal4

To a solution of *N*-glycan acceptor (5-10 mM) and CMP-Neu5Ac (1.5 eq) in a sodium cacodylate buffer solution (100 mM, pH 7.2) containing BSA (1% total volume, stock solution = 10 mg/mL) was added calf intestinal alkaline phosphatase (CIAP, 1% total volume, 1000 U/mL) and ST3Gal4 (100 μg/μmol acceptor), and the reaction mixture was incubated at 37 °C. Reaction progress was monitored by ESI-MS, if starting material remained after 18 h, another portion of ST3Gal4 and CMP-Neu5Ac (0.5 eq per Neu5Ac) were added until no starting material could be detected. The reaction mixture was centrifuged, and the resulting supernatant was loaded on Bio-Gel P4 column (eluent: 0.1 M NH_4HCO_3). Product containing fractions were combined and lyophilized to afford the desired product as a white amorphous solid.

Procedure for the selective removal of terminal galactose using galactosidase from *E. coli*

To a solution of *N*-glycan (5-20 mM) in a Tris buffer solution (100 mM, pH 7.5) was added galactosidase from *E. coli* (250 U/mL), and the mixture was incubated at 37 °C for 12 h. The reaction mixture was centrifuged, and the resulting supernatant was loaded on Bio-Gel P4 column (eluent: 0.1 M NH_4HCO_3). Product containing fractions were combined and lyophilized

to afford the desired product as a white amorphous solid. The product containing impure side fractions were combined and lyophilized for further purification by HPLC.

General Procedure for the installation of bisecting β1,4 GlcNAc using MGAT3

To a solution of *N*-glycan acceptor (5-10 mM) and UDP-GlcNAc (1.2 eq) in a MES buffer solution (100 mM, pH 7.0) containing $MnCl_2$ (2 mM) and BSA (1% total volume, stock solution = 10 mg/mL) was added calf intestinal alkaline phosphatase (CIAP, 1% total volume, 1000 U/mL) and MGAT3 (50 μg/μmol acceptor). The reaction mixture was incubated at 37 ^{o}C. Reaction progress was monitored by MALDI-TOF MS or ESI-MS, if starting material remained after 18 h, another portion of MGAT3 and UDP-GlcNAc (0.3 eq) were added until no starting material could be detected. The reaction mixture was centrifuged, and the resulting supernatant was loaded on Bio-Gel P4 column (eluent: 0.1 M NH_4HCO_3). Product containing fractions were combined and lyophilized to afford the desired product as a white amorphous solid.

Procedure for the installation of β1,4 Gal for bisected *N*-glycan using B4GalT1

To a solution of *N*-glycan acceptor (5-10 mM) and UDP-Gal (1 eq per Gal) in a Tris buffer solution (100 mM, pH 7.5) containing $MnCl_2$ (10 mM) and BSA (1% total volume, stock solution = 10 mg/mL) was added calf intestinal alkaline phosphatase (CIAP, 1% total volume, 1000 U/mL) and B4GalT1 (40 μg/μmol acceptor). The reaction mixture was incubated at 37 ^{o}C for 16 h. The reaction mixture was centrifuged, and the resulting supernatant was loaded on Bio-Gel P4 column (eluent: 0.1 M NH_4HCO_3). Product containing fractions were combined and lyophilized to afford the desired product as a white amorphous solid. The product containing impure side fractions were combined and lyophilized for further purification by HPLC.

General procedure for the selective removal of core fucose using fucosidase FucA1

To a solution of *N*-glycan (1-10 mM) in sodium acetate buffer solution (100 mM, pH 4.5) was added fucosidase FucA1 (100 μg/μmol acceptor) containing BSA (1% total volume, stock solution = 10 mg/mL), and the mixture was incubated at 37 ^{o}C. Reaction progress was monitored by ESI-MS, if starting material remained after 16 h, another portion of fucosidase FucA1 (50 μg/μmol acceptor) were added until no starting material could be detected. The reaction mixture was centrifuged, and the resulting supernatant was loaded on Bio-Gel P4 column (eluent: 0.1 M NH_4HCO_3). Product containing fractions were combined and lyophilized to afford the desired product as a white amorphous solid.

4. Divergent enzymatic synthesis of core-fucosylated biantennary *N*-glycans

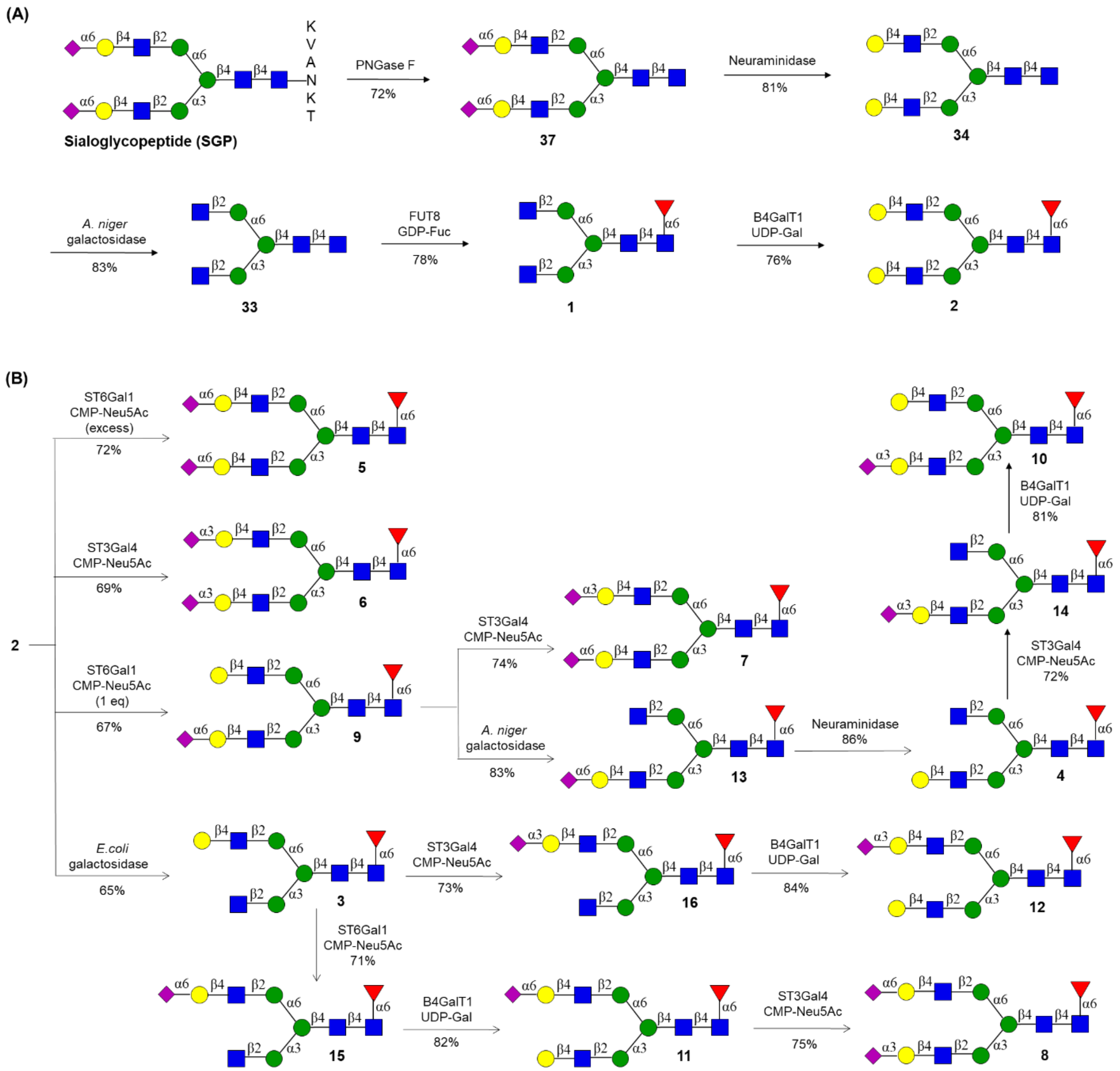

Scheme S1. (A) Enzymatic synthesis core-fucosylated *N*-glycan **2** from SGP; (B) Divergent enzymatic synthesis of core-fucosylated *N*-glycans **3-16**.

5. Divergent enzymatic synthesis of bisected *N*-glycans

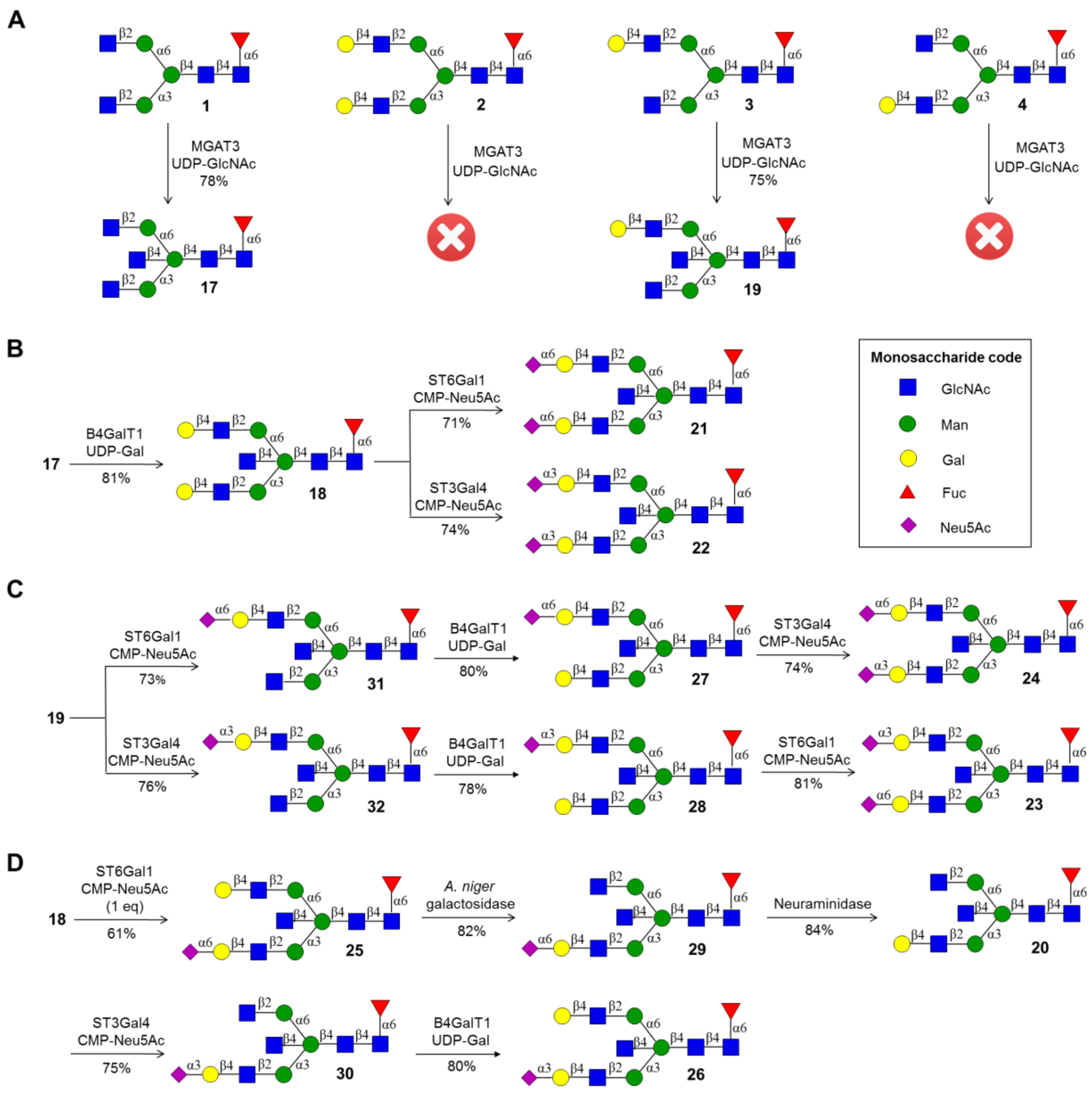

Scheme S2. (A) The substrate specificity study of MGAT3 and enzymatic synthesis bisected *N*-glycans **17** and **19**; (B) Enzymatic synthesis of symmetrical disialylated isomers **21** and **22**; (C) Enzymatic synthesis of asymmetrical disialylated isomers **23** and **24**; (D) Enzymatic synthesis of asymmetrical monosialylated isomers **25** and **26**, isomers **29** and **30**.

6. LC-MS analysis and purification of complex *N*-glycans

Table S1. The procedure for analysis of *N*-glycans

Time (min)	A%
0	35
35	50
40	60
50	50
60	35

Analysis was performed on Shimadzu LC-MS2020 with XBridge® Amide 3.5 μm, 2.1 mm x 150 mm column (Waters) at a flow rate of 0.16 mL/min using ESI-MS for compound detection. Mobile phase A consisted of consisted of 100 mM ammonium formate adjusted to pH 3.5 using formic acid; mobile phase B consisted of 100% acetonitrile.

Table S2. The semi-preparative procedure for purification of *N*-glycans

Time (min)	A%
0	35
35	50
40	60
50	50
60	35

Purification by semi-preparative HPLC was performed on Shimadzu LC-MS2020 using XBridge® Amide 5 μm, 10 mm x 250 mm column (Waters) at a flow rate of 3.8 mL/min, and 1% of the flow was diverted to ESI-MS detector using a splitter. Mobile phase A consisted of 10 mM ammonium formate adjusted to pH 3.5 using formic acid; mobile phase B consisted of 100% acetonitrile.

LC-MS profiles of *N*-glycan isomers 21-24

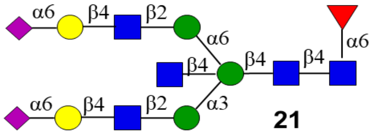

21

ESI-MS: $[M\text{-}2H]^{2-}$ calcd for $C_{98}H_{159}N_7O_{71}^{2-}$,1284.95; found: 1284.95; T = 25.27 min

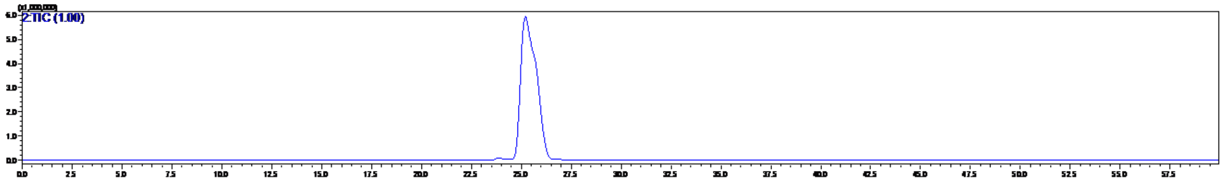

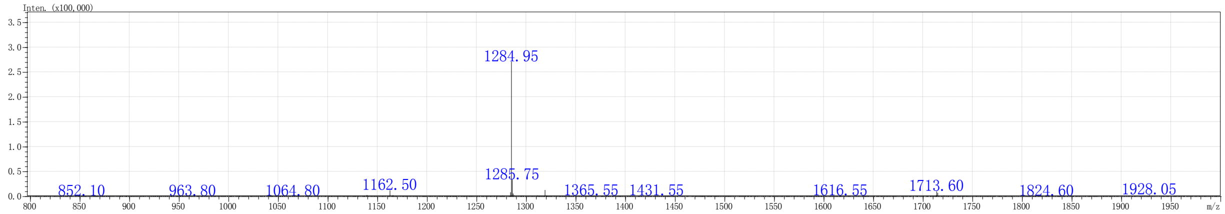

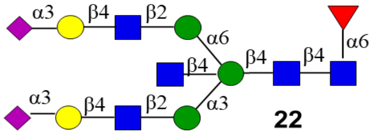

22

ESI-MS: $[M\text{-}2H]^{2-}$ calcd for $C_{98}H_{159}N_7O_{71}^{2-}$,1284.95; found: 1285.00; T = 22.23 min

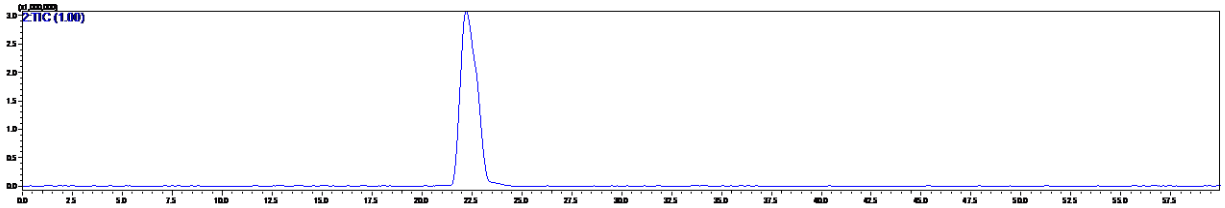

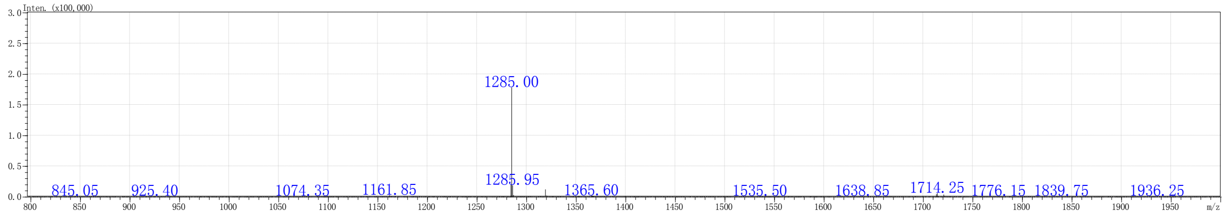

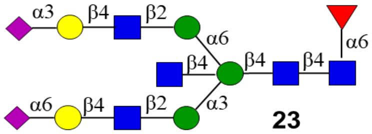

23

ESI-MS: $[M-2H]^{2-}$ calcd for $C_{98}H_{159}N_7O_{71}^{2-}$,1284.95; found: 1285.10; T = 23.63 min

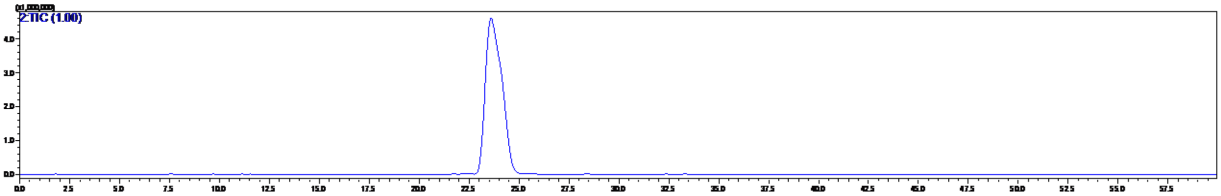

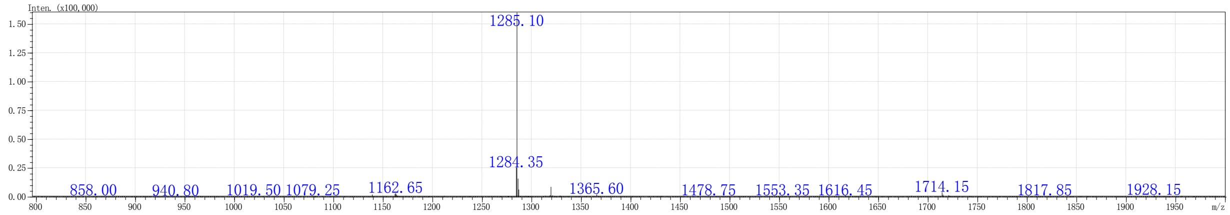

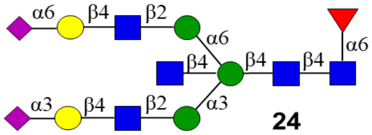

24

ESI-MS: $[M-2H]^{2-}$ calcd for $C_{98}H_{159}N_7O_{71}^{2-}$,1284.95; found: 1285.05; T = 23.43 min

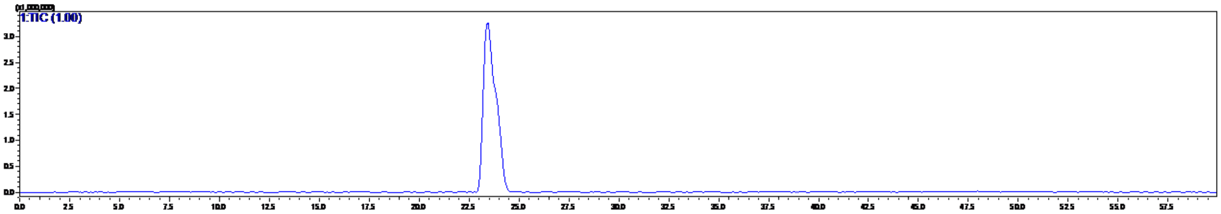

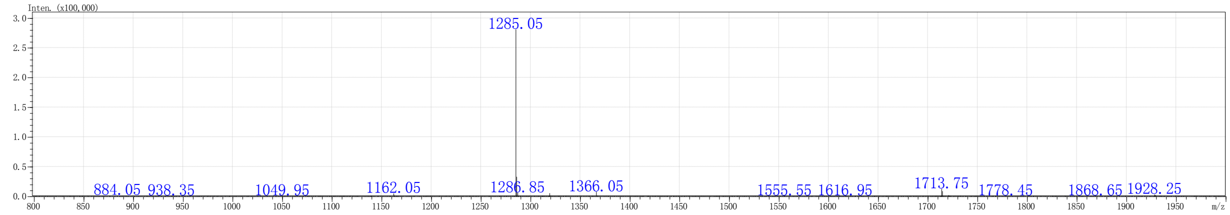

7. General procedure for installing functional spacer to reducing terminal of glycan

Glycans **1-64** (10-20 mM) and the spacer 2-[(methylamino)oxy]ethanamine (100 eq) were dissolved in aqueous NaOAc buffer (100 mM, 50-100 μL), and the final pH of reaction mixture was adjusted to 4.0-5.0. The reaction was incubated at 37 ^{o}C for 2 days. The resulting mixture was loaded on a Carbograph cartridge for rapid solid phase extraction (SPE) to remove excess spacer and salt (eluent: 10 mM NH_4HCO_3), followed by releasing product with a mixture solution of CH_3CN and 10 mM NH_4HCO_3 (v/v 1:1). Product containing fractions were combined and lyophilized to afford glycans with an amino-containing linker at reducing terminal.[2]

8. Microarray procedure[3]

All compounds were printed on NHS-activated Nexterion® slides purchased from Schott using a Scienion sciCLEAN 8 non-contact microarray printer. Biotinylated plant lectins *Phaseolus vulgaris* leucoagglutinin (PHA-L), Wheat germ agglutinin (WGA), *Aleuria Aurantia* lectin (AAL), *Ricinus communis* agglutinin (RCA-I), *Erythrina cristagalli* lectin (ECL), *Maackia amurensis* lectin II (MAL-II) and *Sambucus Nigra* Agglutinin (SNA) were purchased from Vector Labs. Biotinylated Fcγ receptors: biotinylated human CD64 protein, biotinylated human CD32a (H167) protein, biotinylated human CD16a (V176) protein and biotinylated human FcRn were purchased from Acro Biosystems. Human DC-SIGN protein (Fc-tag), human L-SIGN protein (Fc-tag), and human DCIR/CLEC4A protein (Fc-tag) were purchased from Sino Biological. Human Siglec-10 protein (Fc-tag) and human Galectin-9 protein (His-tag) were purchased from Acro Biosystems. Biotinylated goat anti-Human IgG Fc Antibody was purchased from Thermo Fisher Scientific. Human Galectin-9 Biotinylated Antibody was purchased from R&D Systems. Streptavidin-AlexaFluor® 635 conjugate was purchased from Thermo Fisher Scientific. Stained slides were analyzed using InnoScan 710 Microarray Scanner from Innopsys.

All functionalized compounds (100 μM) were dissolved in a sodium phosphate buffer (pH 8.5, 250 mM) and were printed at 55% relative humidity in replicates of six with spot volume ~400 pL. Slides were printed with 16 subarrays (2 x 8) containing 529 spots (23 x 23). After printing, slides were incubated in a saturated NaCl chamber (providing an environment with enough humidity) for 24 h and then quenched for 1 h with 50 mM ethanolamine in a Tris buffer (pH 9.0, 100 mM). The blocked slides were then rinsed with DI water, dried by centrifugation, and kept in a desiccator at room temperature for future use.

Subarrays were incubated with 100 μL biotinylated proteins (PHA-L, WGA, AAL, ECL,

RCA-I, MAL-II, SNA, biotinylated human CD64 protein, biotinylated human CD32a (H167) protein, biotinylated human CD16a (V176) protein and biotinylated human FcRn) at the indicated concentrations in TSM binding buffer (20 mM Tris Cl, pH 7.4, 150 mM NaCl, 2 mM $CaCl_2$, 2 mM $MgCl_2$, 0.05% Tween, 1% BSA) on the rotating shaker at 60 rpm for 1 h at room temperature. After 1 h incubation, the samples were removed from each chamber by pipetting, followed by four repeating washes with 200 μL TSM washing buffer (20 mM Tris Cl, 150 mM NaCl, 2 mM $CaCl_2$, 2 mM $MgCl_2$, 0.05% Tween) and four repeating washes with 200 μL TSM buffer (20 mM Tris Cl, pH 7.4, 150 mM NaCl, 2 mM $CaCl_2$ and 2 mM $MgCl_2$). Subarrays were then incubated with 100 μL Streptavidin-AlexaFluor® 635 (5 μg/mL) in TSM binding buffer on the rotating shaker at 60 rpm for 1 h at room temperature, followed by the above washes with TSM washing buffer and TSM buffer. The slides were then washed twice with DI water, dried by a slight centrifugation and stored in the dark before analysis.

Recombinant human DC-SIGN protein (Fc-tag), human L-SIGN protein (Fc-tag), and human DCIR/CLEC4A protein (Fc-tag) were assayed at the indicated concentrations premixed with biotinylated anti-IgG Fc (5 μg/mL) and Streptavidin-AlexaFluor® 635 (5 μg/mL) for 1 h incubation on the rotating shaker at 60 rpm at room temperature. After the incubation, the similar washing steps were performed as described above.

Human Siglec-10 protein (Fc-tag) at indicated concentration in TSM binding buffer (100 μL) was added to a subarray and incubated at room temperature on the rotating shaker at 60 rpm for 1 h, followed by four subsequent washes with TSM washing buffer and TSM buffer. Then biotinylated anti-IgG Fc (5 μg/mL, 100 μL) was added and incubated, and then washed with the same protocol. Streptavidin-AlexaFluor® 635 (5 μg/mL, 100 μL) was finally added at incubated for 1 h, with subsequent washing procedures and centrifugation as described above.

Human Galectin-9 protein (His-tag) at indicated concentration in TSM binding buffer (100 μL) was added to a subarray and incubated at room temperature on the rotating shaker at 60 rpm for 1 h, followed by four subsequent washes with TSM washing buffer and TSM buffer. Then human Galectin-9 Biotinylated antibody (5 μg/mL, 100 μL) was added and incubated, and then washed with the same protocol. Streptavidin-AlexaFluor® 635 (5 μg/mL, 100 μL) was finally added and incubated for 1 h, with subsequent washing procedures and centrifugation as described above.

The pre-prepared slides were scanned for fluorescence on an InnoScan 710 Microarray

Scanner. The detection gain was adjusted to avoid saturation of the signal. The data were processed with Mapix software and further analyzed using Microsoft Excel. After removal of the lowest and highest value of the six replicates, the mean background-subtracted fluorescent intensities were calculated (n=4), and the collected data were analyzed and graphed by using Microsoft Excel.

9. NMR nomenclature

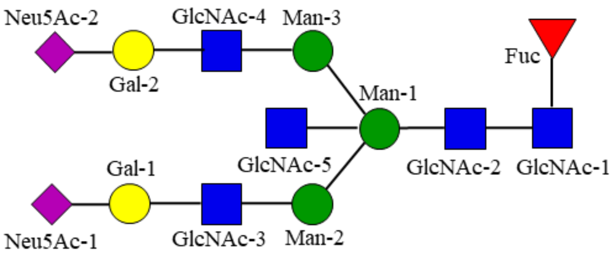

10. NMR assignments and MS data

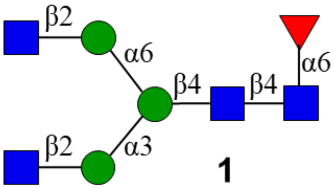

1

	H1	H2	H3	H4	H5	H6
GlcNAc-1α	5.18 (d, J = 3.1 Hz)	3.89	3.87	3.79	NR	NR
GlcNAc-1β	4.70 (d, J = 8.1 Hz)	3.70	3.67	3.66	NR	NR
GlcNAc-2	4.67	3.78	3.60	3.74	NR	NR
GlcNAc-3	4.56 (d, J = 7.8 Hz)	3.71	3.55	3.45	NR	NR
GlcNAc-4	4.56 (d, J = 7.8 Hz)	3.71	3.55	3.45	NR	NR
Man-1	4.78	4.25	3.78	3.63	NR	NR
Man-2	5.12	4.19	3.90	3.74	NR	NR
Man-3	4.92	4.10	3.89	3.62	NR	NR
Fuc	4.90	3.79	3.90	3.80	4.12	1.21

ESI-MS: $[M+H]^+$ calcd for $C_{56}H_{95}N_4O_{40}^+$, 1463.55; found: 1463.15.

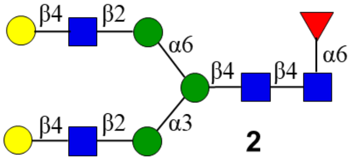

2

	H1	H2	H3	H4	H5	H6
GlcNAc-1α	5.18 (d, J = 3.1 Hz)	3.90	3.87	3.79	NR	NR
GlcNAc-1β	4.70 (d, J = 8.1 Hz)	3.71	3.68	3.66	NR	NR
GlcNAc-2	4.67	3.79	3.60	3.75	NR	NR
GlcNAc-3	4.58 (d, J = 7.8 Hz)	3.75	3.58	3.73	NR	NR
GlcNAc-4	4.58 (d, J = 7.8 Hz)	3.75	3.58	3.73	NR	NR
Man-1	4.77	4.26	3.78	3.63	NR	NR
Man-2	5.12	4.20	3.90	3.75	NR	NR
Man-3	4.93	4.11	3.89	3.62	NR	NR
Gal-1	4.47 (d, J = 8.0 Hz)	3.55	3.67	3.93	NR	NR
Gal-2	4.47 (d, J = 8.0 Hz)	3.55	3.67	3.93	NR	NR
Fuc	4.90	3.79	3.90	3.80	4.12	1.21

ESI-MS: $[M+H]^+$ calcd for $C_{68}H_{115}N_4O_{50}^+$, 1787.66; found: 1787.25.

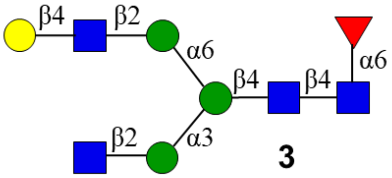

3

	H1	H2	H3	H4	H5	H6
GlcNAc-1α	5.18<br>(d, *J* = 3.2 Hz)	3.89	3.87	3.79	NR	NR
GlcNAc-1β	4.70<br>(d, *J* = 8.1 Hz)	3.71	3.68	3.66	NR	NR
GlcNAc-2	4.67	3.80	3.60	3.75	NR	NR
GlcNAc-3	4.56<br>(d, *J* = 8.5 Hz)	3.70	3.55	3.44	NR	NR
GlcNAc-4	4.58<br>(d, *J* = 8.2 Hz)	3.75	3.58	3.75	NR	NR
Man-1	4.77	4.25	3.78	3.63	NR	NR
Man-2	5.12	4.19	3.90	3.75	NR	NR
Man-3	4.93	4.11	3.90	3.62	NR	NR
Gal	4.48<br>(d, *J* = 7.9 Hz)	3.55	3.67	3.93	NR	NR
Fuc	4.90	3.79	3.90	3.80	4.12	1.21

ESI-MS: $[M+H]^+$ calcd for $C_{62}H_{105}N_4O_{45}^+$, 1625.61; found: 1625.25.

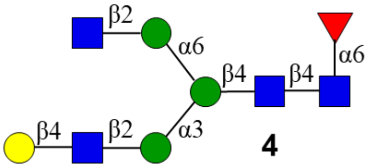

4

	H1	H2	H3	H4	H5	H6
GlcNAc-1α	5.18 (d, J = 3.8 Hz)	3.89	3.87	3.79	NR	NR
GlcNAc-1β	4.70 (d, J = 8.2 Hz)	3.70	3.68	3.66	NR	NR
GlcNAc-2	4.67	3.79	3.60	3.74	NR	NR
GlcNAc-3	4.58 (d, J = 8.5 Hz)	3.74	3.58	3.73	NR	NR
GlcNAc-4	4.56 (d, J = 8.6 Hz)	3.69	3.54	3.45	NR	NR
Man-1	4.77	4.25	3.78	3.63	NR	NR
Man-2	5.12	4.19	3.91	3.75	NR	NR
Man-3	4.92	4.11	3.89	3.62	NR	NR
Gal	4.47 (d, J = 7.7 Hz)	3.55	3.67	3.93	NR	NR
Fuc	4.90	3.79	3.90	3.80	4.11	1.21

ESI-MS: $[M+H]^+$ calcd for $C_{62}H_{105}N_4O_{45}^+$, 1625.61; found: 1625.20.

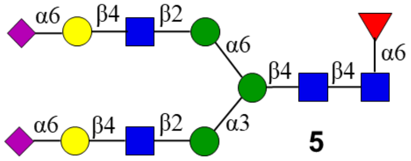

5

	H1	H2	H3	H4	H5	H6
GlcNAc-1α	5.18 (d, J = 3.2 Hz)	3.90	3.87	3.79	NR	NR
GlcNAc-1β	4.69 (d, J = 8.5 Hz)	3.71	3.69	3.67	NR	NR
GlcNAc-2	4.67	3.80	3.60	3.74	NR	NR
GlcNAc-3	4.61 (d, J = 7.3 Hz)	3.76	3.60	3.66	NR	NR
GlcNAc-4	4.61 (d, J = 7.3 Hz)	3.76	3.60	3.66	NR	NR
Man-1	4.78	4.26	3.78	3.65	NR	NR
Man-2	5.13	4.20	3.90	3.75	NR	NR
Man-3	4.94	4.11	3.90	3.62	NR	NR
Gal-1	4.45 (d, J = 8.0 Hz)	3.54	3.67	3.93	NR	NR
Gal-2	4.45 (d, J = 8.0 Hz)	3.54	3.67	3.93	NR	NR
Fuc	4.89	3.79	3.90	3.80	4.12	1.21
Neu5Ac-1	-	-	H_{ax}: 1.72 (t, J = 12.2 Hz) H_{eq}: 2.67	3.65	3.81	3.70
Neu5Ac-2	-	-	H_{ax}: 1.72 (t, J = 12.2 Hz) H_{eq}: 2.67	3.65	3.81	3.70

ESI-MS: $[M+2H]^{2+}$ calcd for $C_{90}H_{150}N_6O_{66}^{2+}$, 1185.43; found: 1185.30.

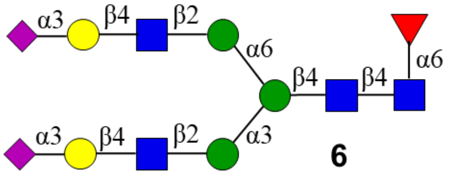

6

	H1	H2	H3	H4	H5	H6
GlcNAc-1α	5.18 (d, J = 3.1 Hz)	3.90	3.87	3.79	NR	NR
GlcNAc-1β	4.69 (d, J = 8.0 Hz)	3.71	3.69	3.66	NR	NR
GlcNAc-2	4.67	3.80	3.60	3.75	NR	NR
GlcNAc-3	4.57 (d, J = 8.0 Hz)	3.75	3.57	3.71	NR	NR
GlcNAc-4	4.57 (d, J = 8.0 Hz)	3.75	3.57	3.71	NR	NR
Man-1	4.77	4.25	3.78	3.62	NR	NR
Man-2	5.12	4.19	3.90	3.75	NR	NR
Man-3	4.92	4.11	3.89	3.62	NR	NR
Gal-1	4.54 (d, J = 7.8 Hz)	3.57	4.12	3.96	NR	NR
Gal-2	4.55 (d, J = 7.8 Hz)	3.57	4.12	3.96	NR	NR
Fuc	4.90	3.79	3.90	3.81	4.12	1.22
Neu5Ac-1	-	-	H_{ax}: 1.82 (t, J = 12.1 Hz) H_{eq}: 2.76	3.69	3.85	3.64
Neu5Ac-2	-	-	H_{ax}: 1.81 (t, J = 12.1 Hz) H_{eq}: 2.76	3.69	3.85	3.64

ESI-MS: $[M+2H]^{2+}$ calcd for $C_{90}H_{150}N_6O_{66}^{2+}$, 1185.43; found: 1185.60.

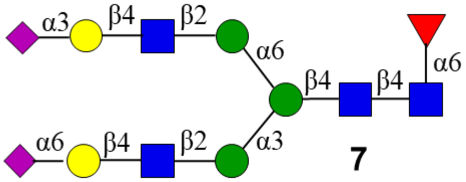

7

	H1	H2	H3	H4	H5	H6
GlcNAc-1α	5.18 (d, J = 3.0 Hz)	3.90	3.87	3.79	NR	NR
GlcNAc-1β	4.69 (d, J = 8.0 Hz)	3.71	3.69	3.66	NR	NR
GlcNAc-2	4.66	3.79	3.60	3.73	NR	NR
GlcNAc-3	4.60 (d, J = 7.5 Hz)	3.76	3.60	3.65	NR	NR
GlcNAc-4	4.57 (d, J = 8.3 Hz)	3.75	3.56	3.69	NR	NR
Man-1	4.77	4.26	3.79	3.62	NR	NR
Man-2	5.13	4.20	3.90	3.75	NR	NR
Man-3	4.92	4.11	3.89	3.62	NR	NR
Gal-1	4.44 (d, J = 8.3 Hz)	3.54	3.67	3.92	NR	NR
Gal-2	4.55 (d, J = 8.1 Hz)	3.57	4.12	3.96	NR	NR
Fuc	4.90	3.79	3.90	3.81	4.12	1.23
Neu5Ac-1	-	-	H_{ax}: 1.72 (t, J = 12.2 Hz) H_{eq}: 2.66	3.65	3.80	3.70
Neu5Ac-2	-	-	H_{ax}: 1.80 (t, J = 12.1 Hz) H_{eq}: 2.76	3.69	3.85	3.64

ESI-MS: $[M+2H]^{2+}$ calcd for $C_{90}H_{150}N_6O_{66}^{2+}$, 1185.43; found: 1185.45.

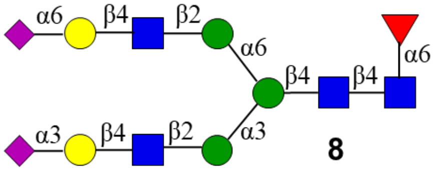

8

	H1	H2	H3	H4	H5	H6
GlcNAc-1α	5.18<br>(d, J = 2.9 Hz)	3.90	3.87	3.79	NR	NR
GlcNAc-1β	4.69<br>(d, J = 8.4 Hz)	3.71	3.69	3.65	NR	NR
GlcNAc-2	4.66	3.78	3.60	3.73	NR	NR
GlcNAc-3	4.57<br>(d, J = 7.6 Hz)	3.75	3.58	3.72	NR	NR
GlcNAc-4	4.60<br>(d, J = 6.5 Hz)	3.76	3.59	3.66	NR	NR
Man-1	4.77	4.26	3.77	3.64	NR	NR
Man-2	5.11	4.20	3.91	3.75	NR	NR
Man-3	4.94	4.11	3.90	3.62	NR	NR
Gal-1	4.54<br>(d, J = 7.8 Hz)	3.57	4.12	3.96	NR	NR
Gal-2	4.45<br>(d, J = 7.8 Hz)	3.54	3.67	3.93	NR	NR
Fuc	4.90	3.79	3.90	3.81	4.12	1.22
Neu5Ac-1	-	-	H_{ax}: 1.80<br>(t, J = 12.1 Hz)<br>H_{eq}: 2.76	3.69	3.84	3.64
Neu5Ac-2	-	-	H_{ax}: 1.72<br>(t, J = 12.2 Hz)<br>H_{eq}: 2.67	3.66	3.80	3.70

ESI-MS: $[M+2H]^{2+}$ calcd for $C_{90}H_{150}N_6O_{66}{}^{2+}$, 1185.43; found: 1185.65.

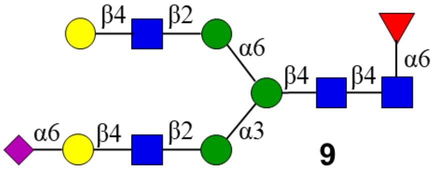

9

	H1	H2	H3	H4	H5	H6
GlcNAc-1α	5.18<br>(d, J = 3.1 Hz)	3.90	3.87	3.79	NR	NR
GlcNAc-1β	4.69<br>(d, J = 8.2 Hz)	3.71	3.69	3.65	NR	NR
GlcNAc-2	4.67	3.78	3.60	3.73	NR	NR
GlcNAc-3	4.61<br>(d, J = 7.7 Hz)	3.75	3.60	3.65	NR	NR
GlcNAc-4	4.58<br>(d, J = 8.2 Hz)	3.75	3.57	3.71	NR	NR
Man-1	4.77	4.26	3.79	3.64	NR	NR
Man-2	5.14	4.20	3.90	3.75	NR	NR
Man-3	4.93	4.11	3.90	3.62	NR	NR
Gal-1	4.45<br>(d, J = 7.9 Hz)	3.54	3.67	3.93	NR	NR
Gal-2	4.47<br>(d, J = 7.8 Hz)	3.55	3.67	3.93	NR	NR
Fuc	4.90	3.79	3.90	3.81	4.12	1.22
Neu5Ac	-	-	H_{ax}: 1.72<br>(t, J = 12.2 Hz)<br>H_{eq}: 2.67	3.66	3.80	3.70

ESI-MS: $[M+2H]^{2+}$ calcd for $C_{79}H_{133}N_5O_{58}{}^{2+}$, 1039.88; found: 1039.95.

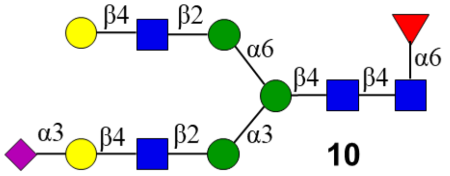

10

	H1	H2	H3	H4	H5	H6
GlcNAc-1α	5.18 (d, J = 3.1 Hz)	3.90	3.88	3.79	NR	NR
GlcNAc-1β	4.69 (d, J = 8.2 Hz)	3.71	3.69	3.65	NR	NR
GlcNAc-2	4.66	3.78	3.60	3.73	NR	NR
GlcNAc-3	4.59 (d, J = 7.7 Hz)	3.75	3.57	3.71	NR	NR
GlcNAc-4	4.58 (d, J = 8.2 Hz)	3.75	3.57	3.71	NR	NR
Man-1	4.77	4.26	3.78	3.63	NR	NR
Man-2	5.12	4.20	3.89	3.74	NR	NR
Man-3	4.93	4.11	3.90	3.62	NR	NR
Gal-1	4.54 (d, J = 7.9 Hz)	3.57	4.12	3.95	NR	NR
Gal-2	4.47 (d, J = 7.9 Hz)	3.54	3.67	3.93	NR	NR
Fuc	4.90	3.79	3.90	3.81	4.12	1.22
Neu5Ac	-	-	H_{ax}: 1.80 (t, J = 12.2 Hz) H_{eq}: 2.75	3.69	3.85	3.63

ESI-MS: $[M+2H]^{2+}$ calcd for $C_{79}H_{133}N_5O_{58}^{2+}$, 1039.88; found: 1040.00.

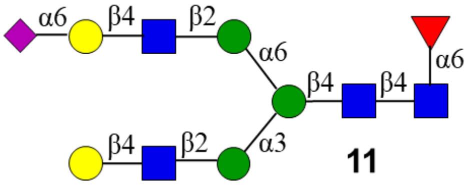

11

	H1	H2	H3	H4	H5	H6
GlcNAc-1α	5.18 (d, J = 3.2 Hz)	3.90	3.87	3.79	NR	NR
GlcNAc-1β	4.69 (d, J = 8.1 Hz)	3.71	3.69	3.65	NR	NR
GlcNAc-2	4.67	3.78	3.60	3.73	NR	NR
GlcNAc-3	4.58 (d, J = 7.4 Hz)	3.75	3.58	3.73	NR	NR
GlcNAc-4	4.61 (d, J = 7.6 Hz)	3.76	3.60	3.65	NR	NR
Man-1	4.77	4.26	3.79	3.64	NR	NR
Man-2	5.12	4.20	3.91	3.75	NR	NR
Man-3	4.94	4.11	3.90	3.62	NR	NR
Gal-1	4.47 (d, J = 7.9 Hz)	3.55	3.67	3.93	NR	NR
Gal-2	4.45 (d, J = 7.8 Hz)	3.54	3.67	3.93	NR	NR
Fuc	4.90	3.79	3.90	3.81	4.12	1.22
Neu5Ac	-	-	H_{ax}: 1.72 (t, J = 12.2 Hz) H_{eq}: 2.67	3.65	3.80	3.70

ESI-MS: $[M+2H]^{2+}$ calcd for $C_{79}H_{133}N_5O_{58}{}^{2+}$, 1039.88; found: 1039.75.

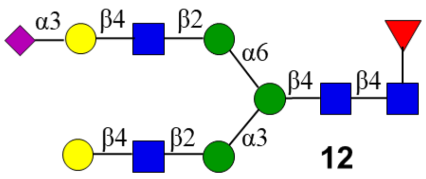

12

	H1	H2	H3	H4	H5	H6
GlcNAc-1α	5.18<br>(d, J = 3.1 Hz)	3.89	3.87	3.79	NR	NR
GlcNAc-1β	4.69<br>(d, J = 8.1 Hz)	3.71	3.68	3.65	NR	NR
GlcNAc-2	4.66	3.78	3.60	3.74	NR	NR
GlcNAc-3	4.58<br>(d, J = 8.3 Hz)	3.75	3.57	3.71	NR	NR
GlcNAc-4	4.59<br>(d, J = 8.2 Hz)	3.75	3.57	3.71	NR	NR
Man-1	4.77	4.25	3.79	3.62	NR	NR
Man-2	5.12	4.19	3.90	3.75	NR	NR
Man-3	4.92	4.11	3.89	3.62	NR	NR
Gal-1	4.47<br>(d, J = 7.8 Hz)	3.54	3.66	3.92	NR	NR
Gal-2	4.54<br>(d, J = 7.9 Hz)	3.57	4.12	3.96	NR	NR
Fuc	4.90	3.79	3.90	3.81	4.12	1.22
Neu5Ac	-	-	H_{ax}: 1.80<br>(t, J = 12.1 Hz)<br>H_{eq}: 2.75	3.69	3.84	3.64

ESI-MS: $[M+2H]^{2+}$ calcd for $C_{79}H_{133}N_5O_{58}{}^{2+}$, 1039.88; found: 1039.80.

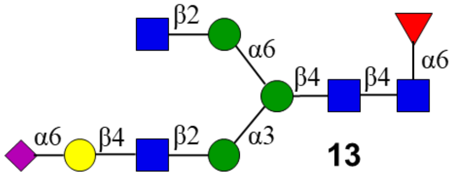

13

	H1	H2	H3	H4	H5	H6
GlcNAc-1α	5.18 (d, J = 3.1 Hz)	3.90	3.88	3.79	NR	NR
GlcNAc-1β	4.69 (d, J = 8.4 Hz)	3.71	3.68	3.65	NR	NR
GlcNAc-2	4.66	3.77	3.60	3.74	NR	NR
GlcNAc-3	4.60 (d, J = 7.5 Hz)	3.76	3.60	3.65	NR	NR
GlcNAc-4	4.55 (d, J = 8.4 Hz)	3.71	3.55	3.46	NR	NR
Man-1	4.78	4.26	3.78	3.64	NR	NR
Man-2	5.14	4.20	3.90	3.74	NR	NR
Man-3	4.92	4.11	3.90	3.62	NR	NR
Gal	4.44 (d, J = 8.0 Hz)	3.54	3.67	3.92	NR	NR
Fuc	4.90	3.79	3.90	3.81	4.12	1.22
Neu5Ac	-	-	H_{ax}: 1.72 (t, J = 12.1 Hz) H_{eq}: 2.67	3.65	3.80	3.70

ESI-MS: $[M+2H]^{2+}$ calcd for $C_{73}H_{123}N_5O_{53}^{2+}$, 958.85; found: 958.95.

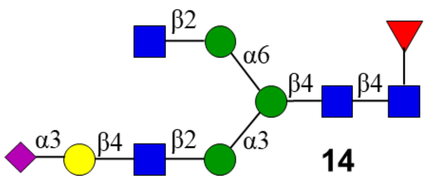

14

	H1	H2	H3	H4	H5	H6
GlcNAc-1α	5.18 (d, J = 3.1 Hz)	3.90	3.88	3.79	NR	NR
GlcNAc-1β	4.69 (d, J = 8.1 Hz)	3.71	3.69	3.65	NR	NR
GlcNAc-2	4.66	3.77	3.60	3.74	NR	NR
GlcNAc-3	4.57	3.74	3.58	3.72	NR	NR
GlcNAc-4	4.55	3.71	3.55	3.45	NR	NR
Man-1	4.77	4.25	3.78	3.64	NR	NR
Man-2	5.12	4.19	3.90	3.74	NR	NR
Man-3	4.92	4.11	3.89	3.63	NR	NR
Gal	4.54	3.57	4.12	3.96	NR	NR
Fuc	4.90	3.79	3.90	3.81	4.12	1.22
Neu5Ac	-	-	H_{ax}: 1.80 (t, J = 12.0 Hz) H_{eq}: 2.75	3.69	3.85	3.63

ESI-MS: $[M+2H]^{2+}$ calcd for $C_{73}H_{123}N_5O_{53}^{2+}$, 958.85; found: 959.00.

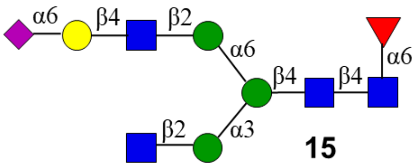

15

	H1	H2	H3	H4	H5	H6
GlcNAc-1α	5.18 (d, J = 3.2 Hz)	3.90	3.88	3.79	NR	NR
GlcNAc-1β	4.69 (d, J = 8.1 Hz)	3.71	3.68	3.65	NR	NR
GlcNAc-2	4.66	3.78	3.60	3.73	NR	NR
GlcNAc-3	4.55 (d, J = 8.4 Hz)	3.71	3.56	3.45	NR	NR
GlcNAc-4	4.61 (d, J = 7.2 Hz)	3.76	3.59	3.66	NR	NR
Man-1	4.78	4.25	3.77	3.64	NR	NR
Man-2	5.12	4.19	3.91	3.75	NR	NR
Man-3	4.94	4.11	3.89	3.62	NR	NR
Gal	4.45 (d, J = 7.9 Hz)	3.54	3.67	3.93	NR	NR
Fuc	4.90	3.79	3.90	3.81	4.12	1.22
Neu5Ac	-	-	H_{ax}: 1.73 (t, J = 12.2 Hz) H_{eq}: 2.67	3.66	3.80	3.70

ESI-MS: $[M+2H]^{2+}$ calcd for $C_{73}H_{123}N_5O_{53}^{2+}$, 958.85; found: 958.90.

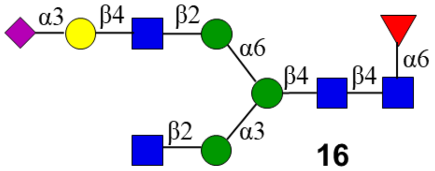

16

	H1	H2	H3	H4	H5	H6
GlcNAc-1α	5.18 (d, J = 3.2 Hz)	3.89	3.86	3.79	NR	NR
GlcNAc-1β	4.70 (d, J = 8.1 Hz)	3.72	3.68	3.66	NR	NR
GlcNAc-2	4.67	3.79	3.60	3.74	NR	NR
GlcNAc-3	4.56	3.70	3.56	3.45	NR	NR
GlcNAc-4	4.58 (d, J = 8.4 Hz)	3.75	3.56	3.70	NR	NR
Man-1	4.77	4.25	3.78	3.62	NR	NR
Man-2	5.12	4.19	3.90	3.75	NR	NR
Man-3	4.93	4.11	3.89	3.62	NR	NR
Gal	4.56	3.57	4.12	3.96	NR	NR
Fuc	4.90	3.79	3.90	3.80	4.12	1.22
Neu5Ac-1	-	-	H_{ax}: 1.80 (t, J = 12.1 Hz) H_{eq}: 2.75	3.69	3.85	3.63

ESI-MS: $[M+2H]^{2+}$ calcd for $C_{73}H_{123}N_5O_{53}^{2+}$, 958.85; found: 958.95.

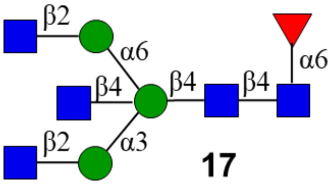

17

	H1	H2	H3	H4	H5	H6
GlcNAc-1α	5.18 (d, J = 3.2 Hz)	3.90	3.88	3.79	NR	NR
GlcNAc-1β	4.69 (d, J = 7.8 Hz)	3.70	3.67	3.66	NR	NR
GlcNAc-2	4.66	3.80	3.58	3.75	NR	NR
GlcNAc-3	4.56 (d, J = 7.9 Hz)	3.71	3.61	3.48	NR	NR
GlcNAc-4	4.55 (d, J = 8.2 Hz)	3.70	3.49	3.41	NR	NR
GlcNAc-5	4.47 (d, J = 8.2 Hz)	3.70	3.58	3.42	NR	NR
Man-1	4.70	4.18	3.87	3.56	NR	NR
Man-2	5.07	4.25	3.90	3.71	NR	NR
Man-3	5.00	4.15	3.85	3.63	NR	NR
Fuc	4.90	3.79	3.90	3.81	4.12	1.22

ESI-MS: $[M+H]^+$ calcd for $C_{64}H_{108}N_5O_{45}^+$, 1666.63; found: 1666.20.

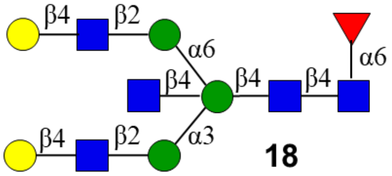

18

	H1	H2	H3	H4	H5	H6
GlcNAc-1α	5.18 (d, J = 2.8 Hz)	3.89	3.87	3.79	NR	NR
GlcNAc-1β	4.70	3.71	3.68	3.66	NR	NR
GlcNAc-2	4.66	3.81	3.59	3.74	NR	NR
GlcNAc-3	4.59	3.76	3.62	3.76	NR	NR
GlcNAc-4	4.58	3.74	3.54	3.64	NR	NR
GlcNAc-5	4.46	3.69	3.57	3.41	NR	NR
Man-1	4.69	4.18	3.87	3.54	NR	NR
Man-2	5.06	4.26	3.91	3.71	NR	NR
Man-3	5.01	4.14	3.84	3.63	NR	NR
Gal-1	4.47	3.54	3.67	3.93	NR	NR
Gal-2	4.47	3.54	3.67	3.93	NR	NR
Fuc	4.91	3.79	3.90	3.80	4.12	1.20

ESI-MS: $[M+2H]^{2+}$ calcd for $C_{76}H_{129}N_5O_{55}^{2+}$, 995.87; found: 995.95.

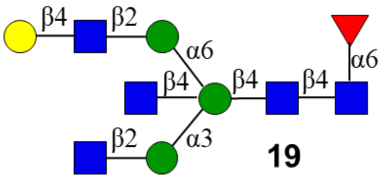

19

	H1	H2	H3	H4	H5	H6
GlcNAc-1α	5.18 (d, *J* = 3.2 Hz)	3.90	3.86	3.79	NR	NR
GlcNAc-1β	4.69	3.71	3.67	3.65	NR	NR
GlcNAc-2	4.66	3.82	3.59	3.74	NR	NR
GlcNAc-3	4.56 (d, *J* = 8.4 Hz)	3.72	3.60	3.47	NR	NR
GlcNAc-4	4.58 (d, *J* = 8.4 Hz)	3.74	3.54	3.64	NR	NR
GlcNAc-5	4.47 (d, *J* = 8.4 Hz)	3.70	3.57	3.40	NR	NR
Man-1	4.69	4.18	3.87	3.53	NR	NR
Man-2	5.06	4.25	3.90	3.71	NR	NR
Man-3	5.01	4.15	3.83	3.64	NR	NR
Gal	4.48 (d, *J* = 7.9 Hz)	3.55	3.67	3.93	NR	NR
Fuc	4.91	3.79	3.90	3.80	4.12	1.20

ESI-MS: $[M+2H]^{2+}$ calcd for $C_{70}H_{119}N_5O_{50}^{2+}$, 914.85; found: 914.95.

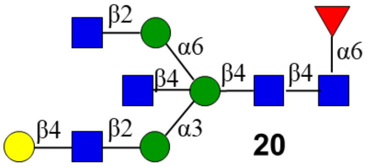

20

	H1	H2	H3	H4	H5	H6
GlcNAc-1α	5.18<br>(d, J = 2.8 Hz)	3.90	3.87	3.78	NR	NR
GlcNAc-1β	4.70	3.72	3.67	3.66	NR	NR
GlcNAc-2	4.66	3.80	3.58	3.74	NR	NR
GlcNAc-3	4.59<br>(d, J = 7.2 Hz)	3.76	3.62	3.76	NR	NR
GlcNAc-4	4.55<br>(d, J = 8.1 Hz)	3.70	3.49	3.41	NR	NR
GlcNAc-5	4.46	3.70	3.57	3.41	NR	NR
Man-1	4.70	4.18	3.86	3.55	NR	NR
Man-2	5.06	4.26	3.90	3.71	NR	NR
Man-3	5.00	4.15	3.84	3.62	NR	NR
Gal	4.47	3.54	3.67	3.93	NR	NR
Fuc	4.90	3.79	3.90	3.80	4.12	1.20

ESI-MS: $[M+2H]^{2+}$ calcd for $C_{70}H_{119}N_5O_{50}{}^{2+}$, 914.85; found: 914.90.

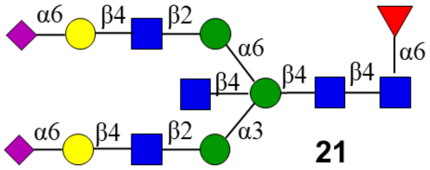

21

	H1	H2	H3	H4	H5	H6
GlcNAc-1α	5.18 (d, J = 2.8 Hz)	3.89	3.87	3.78	NR	NR
GlcNAc-1β	4.69 (d, J = 8.1 Hz)	3.71	3.68	3.65	NR	NR
GlcNAc-2	4.66	3.81	3.59	3.74	NR	NR
GlcNAc-3	4.61 (d, J = 8.8 Hz)	3.77	3.69	3.76	NR	NR
GlcNAc-4	4.59 (d, J = 8.6 Hz)	3.74	3.56	3.69	NR	NR
GlcNAc-5	4.47	3.70	3.58	3.42	NR	NR
Man-1	4.71	4.19	3.87	3.58	NR	NR
Man-2	5.07	4.26	3.90	3.72	NR	NR
Man-3	5.01	4.15	3.85	3.62	NR	NR
Gal-1	4.46	3.54	3.67	3.93	NR	NR
Gal-2	4.46	3.54	3.67	3.93	NR	NR
Fuc	4.90	3.78	3.90	3.81	4.12	1.21
Neu5Ac-1	-	-	H_{ax}: 1.72 (t, J = 12.1 Hz) H_{eq}: 2.67	3.65	3.81	3.70
Neu5Ac-2	-	-	H_{ax}: 1.71 (t, J = 12.2 Hz) H_{eq}: 2.67	3.65	3.81	3.70

ESI-MS: $[M+2H]^{2+}$ calcd for $C_{98}H_{163}N_7O_{71}^{2+}$, 1286.97; found: 1287.15.

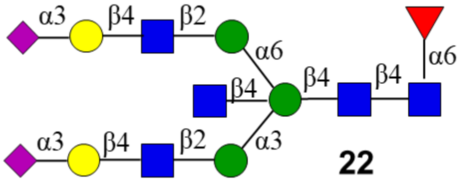

22

	H1	H2	H3	H4	H5	H6
GlcNAc-1α	5.18 (d, J = 2.7 Hz)	3.89	3.86	3.78	NR	NR
GlcNAc-1β	4.69	3.71	3.67	3.65	NR	NR
GlcNAc-2	4.65	3.82	3.58	3.74	NR	NR
GlcNAc-3	4.59	3.77	3.61	3.76	NR	NR
GlcNAc-4	4.56	3.74	3.53	3.63	NR	NR
GlcNAc-5	4.45 (d, J = 8.2 Hz)	3.69	3.57	3.42	NR	NR
Man-1	4.68	4.17	3.86	3.52	NR	NR
Man-2	5.06	4.25	3.90	3.71	NR	NR
Man-3	5.00	4.15	3.83	3.62	NR	NR
Gal-1	4.55	3.57	4.12	3.96	NR	NR
Gal-2	4.55	3.57	4.12	3.96	NR	NR
Fuc	4.91	3.79	3.90	3.81	4.12	1.22
Neu5Ac-1	-	-	H_{ax}: 1.81 (t, J = 12.1 Hz) H_{eq}: 2.76	3.69	3.84	3.63
Neu5Ac-2	-	-	H_{ax}: 1.80 (t, J = 12.2 Hz) H_{eq}: 2.76	3.68	3.84	3.62

ESI-MS: $[M+2H]^{2+}$ calcd for $C_{98}H_{163}N_7O_{71}^{2+}$, 1286.97; found: 1287.10.

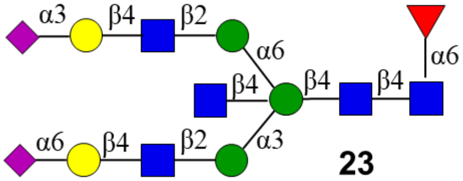

23

	H1	H2	H3	H4	H5	H6
GlcNAc-1α	5.18<br>(d, J = 2.7 Hz)	3.89	3.86	3.78	NR	NR
GlcNAc-1β	4.69	3.70	3.67	3.65	NR	NR
GlcNAc-2	4.65	3.81	3.58	3.73	NR	NR
GlcNAc-3	4.62<br>(d, J = 8.1 Hz)	3.77	3.69	3.76	NR	NR
GlcNAc-4	4.56	3.75	3.52	3.62	NR	NR
GlcNAc-5	4.46	3.69	3.58	3.40	NR	NR
Man-1	4.69	4.18	3.87	3.53	NR	NR
Man-2	5.07	4.26	3.91	3.71	NR	NR
Man-3	5.00	4.15	3.83	3.62	NR	NR
Gal-1	4.44	3.53	3.67	3.93	NR	NR
Gal-2	4.55	3.57	4.12	3.96	NR	NR
Fuc	4.91	3.78	3.89	3.80	4.12	1.20
Neu5Ac-1	-	-	H_{ax}: 1.72<br>(t, J = 12.1 Hz)<br>H_{eq}: 2.67	3.65	3.81	3.70
Neu5Ac-2	-	-	H_{ax}: 1.81<br>(t, J = 12.1 Hz)<br>H_{eq}: 2.76	3.68	3.85	3.63

ESI-MS: $[M+2H]^{2+}$ calcd for $C_{98}H_{163}N_7O_{71}{}^{2+}$, 1286.97; found: 1287.05.

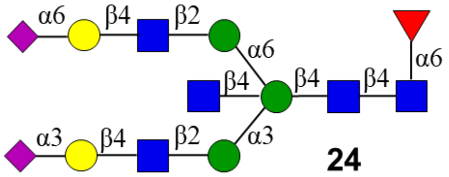

24

	H1	H2	H3	H4	H5	H6
GlcNAc-1α	5.18 (d, J = 2.9 Hz)	3.90	3.86	3.78	NR	NR
GlcNAc-1β	4.69	3.71	3.67	3.65	NR	NR
GlcNAc-2	4.66	3.81	3.58	3.73	NR	NR
GlcNAc-3	4.56	3.77	3.61	3.76	NR	NR
GlcNAc-4	4.62	3.74	3.56	3.69	NR	NR
GlcNAc-5	4.46	3.70	3.57	3.42	NR	NR
Man-1	4.70	4.18	3.87	3.57	NR	NR
Man-2	5.06	4.25	3.90	3.70	NR	NR
Man-3	5.01	4.15	3.85	3.63	NR	NR
Gal-1	4.55 (d, J = 7.8 Hz)	3.57	4.12	3.96	NR	NR
Gal-2	4.45	3.54	3.67	3.94	NR	NR
Fuc	4.90	3.78	3.89	3.80	4.12	1.21
Neu5Ac-1	-	-	H_{ax}: 1.81 (t, J = 12.1 Hz) H_{eq}: 2.75	3.68	3.85	3.63
Neu5Ac-2	-	-	H_{ax}: 1.72 (t, J = 12.1 Hz) H_{eq}: 2.68	3.65	3.81	3.71

ESI-MS: $[M+2H]^{2+}$ calcd for $C_{98}H_{163}N_7O_{71}{}^{2+}$, 1286.97; found: 1287.10.

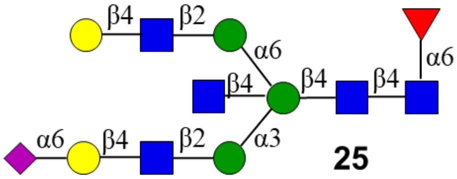

25

	H1	H2	H3	H4	H5	H6
GlcNAc-1α	5.18 (d, J = 3.0 Hz)	3.90	3.86	3.77	NR	NR
GlcNAc-1β	4.69	3.71	3.68	3.66	NR	NR
GlcNAc-2	4.66	3.81	3.59	3.74	NR	NR
GlcNAc-3	4.62 (d, J = 8.0 Hz)	3.77	3.67	3.76	NR	NR
GlcNAc-4	4.58 (d, J = 8.4 Hz)	3.74	3.54	3.64	NR	NR
GlcNAc-5	4.46	3.69	3.58	3.40	NR	NR
Man-1	4.69	4.19	3.87	3.54	NR	NR
Man-2	5.08	4.27	3.90	3.71	NR	NR
Man-3	5.01	4.14	3.84	3.63	NR	NR
Gal-1	4.44	3.54	3.67	3.93	NR	NR
Gal-2	4.48	3.55	3.68	3.94	NR	NR
Fuc	4.90	3.78	3.90	3.81	4.12	1.21
Neu5Ac	-	-	H_{ax}: 1.73 (t, J = 12.1 Hz) H_{eq}: 2.67	3.66	3.81	3.71

ESI-MS: $[M+2H]^{2+}$ calcd for $C_{87}H_{146}N_6O_{63}^{2+}$, 1141.42; found: 1141.55.

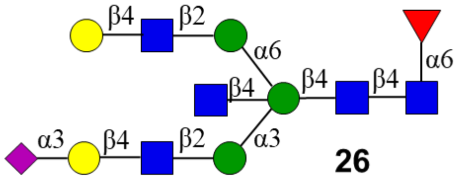

26

	H1	H2	H3	H4	H5	H6
GlcNAc-1α	5.18<br>(d, J = 2.9 Hz)	3.90	3.86	3.78	NR	NR
GlcNAc-1β	4.69	3.71	3.69	3.67	NR	NR
GlcNAc-2	4.66	3.82	3.58	3.74	NR	NR
GlcNAc-3	4.58	3.77	3.61	3.76	NR	NR
GlcNAc-4	4.58	3.74	3.54	3.65	NR	NR
GlcNAc-5	4.46	3.69	3.57	3.41	NR	NR
Man-1	4.69	4.18	3.87	3.54	NR	NR
Man-2	5.06	4.26	3.90	3.71	NR	NR
Man-3	5.01	4.14	3.84	3.63	NR	NR
Gal-1	4.55<br>(d, J = 7.8 Hz)	3.57	4.12	3.96	NR	NR
Gal-2	4.48	3.55	3.67	3.93	NR	NR
Fuc	4.91	3.79	3.90	3.80	4.12	1.20
Neu5Ac	-	-	H_{ax}: 1.79<br>(t, J = 12.1 Hz)<br>H_{eq}: 2.75	3.68	3.85	3.63

ESI-MS: $[M+2H]^{2+}$ calcd for $C_{87}H_{146}N_6O_{63}^{2+}$, 1141.42; found: 1141.60.

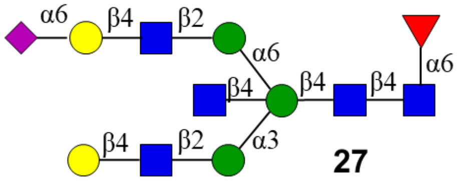

27

	H1	H2	H3	H4	H5	H6
GlcNAc-1α	5.18<br>(d, J = 3.0 Hz)	3.90	3.88	3.78	NR	NR
GlcNAc-1β	4.69	3.70	3.68	3.67	NR	NR
GlcNAc-2	4.66	3.81	3.59	3.74	NR	NR
GlcNAc-3	4.59	3.77	3.62	3.76	NR	NR
GlcNAc-4	4.60	3.73	3.56	3.69	NR	NR
GlcNAc-5	4.46	3.70	3.56	3.42	NR	NR
Man-1	4.70	4.19	3.87	3.57	NR	NR
Man-2	5.06	4.26	3.91	3.71	NR	NR
Man-3	5.02	4.15	3.85	3.62	NR	NR
Gal-1	4.46	3.54	3.67	3.94	NR	NR
Gal-2	4.46	3.54	3.66	3.92	NR	NR
Fuc	4.91	3.80	3.90	3.79	4.12	1.22
Neu5Ac	-	-	H_{ax}: 1.72<br>(t, J = 12.2 Hz)<br>H_{eq}: 2.68	3.66	3.80	3.70

ESI-MS: $[M+2H]^{2+}$ calcd for $C_{87}H_{146}N_6O_{63}^{2+}$, 1141.42; found: 1141.50.

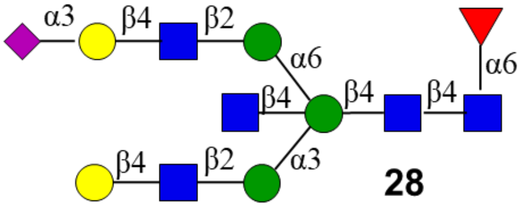

28

	H1	H2	H3	H4	H5	H6
GlcNAc-1α	5.18 (d, J = 3.1 Hz)	3.90	3.88	3.78	NR	NR
GlcNAc-1β	4.69	3.70	3.68	3.66	NR	NR
GlcNAc-2	4.65	3.82	3.58	3.74	NR	NR
GlcNAc-3	4.59	3.77	3.55	3.76	NR	NR
GlcNAc-4	4.56	3.74	3.53	3.63	NR	NR
GlcNAc-5	4.46	3.69	3.57	3.41	NR	NR
Man-1	4.69	4.12	3.86	3.53	NR	NR
Man-2	5.06	4.26	3.91	3.71	NR	NR
Man-3	5.00	4.15	3.82	3.62	NR	NR
Gal-1	4.46	3.54	3.67	3.92	NR	NR
Gal-2	4.55	3.58	4.12	3.97	NR	NR
Fuc	4.91	3.80	3.89	3.78	4.12	1.20
Neu5Ac	-	-	H_{ax}: 1.81 (t, J = 12.2 Hz) H_{eq}: 2.76	3.69	3.85	3.64

ESI-MS: $[M+2H]^{2+}$ calcd for $C_{87}H_{146}N_6O_{63}{}^{2+}$, 1141.42; found: 1141.60.

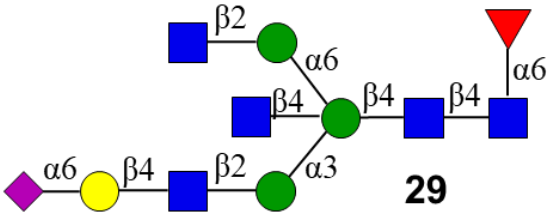

29

	H1	H2	H3	H4	H5	H6
GlcNAc-1α	5.18<br>(d, J = 2.8 Hz)	3.90	3.86	3.78	NR	NR
GlcNAc-1β	4.69	3.71	3.69	3.66	NR	NR
GlcNAc-2	4.66	3.81	3.59	3.74	NR	NR
GlcNAc-3	4.62<br>(d, J = 8.1 Hz)	3.77	3.66	3.78	NR	NR
GlcNAc-4	4.55<br>(d, J = 8.4 Hz)	3.70	3.49	3.41	NR	NR
GlcNAc-5	4.46<br>(d, J = 8.5 Hz)	3.69	3.57	3.41	NR	NR
Man-1	4.70	4.19	3.88	3.56	NR	NR
Man-2	5.08	4.26	3.91	3.71	NR	NR
Man-3	5.00	4.15	3.85	3.63	NR	NR
Gal	4.44<br>(d, J = 8.1 Hz)	3.54	3.67	3.93	NR	NR
Fuc	4.90	3.79	3.90	3.81	4.12	1.21
Neu5Ac	-	-	H_{ax}: 1.72<br>(t, J = 12.1 Hz)<br>H_{eq}: 2.67	3.65	3.82	3.70

ESI-MS: $[M+2H]^{2+}$ calcd for $C_{81}H_{136}N_6O_{58}^{2+}$, 1060.39; found: 1060.50.

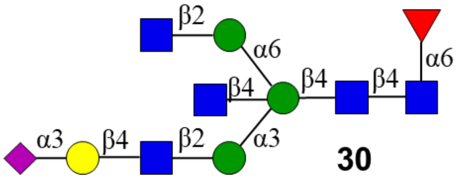

30

	H1	H2	H3	H4	H5	H6
GlcNAc-1α	5.18 (d, J = 3.1 Hz)	3.90	3.86	3.79	NR	NR
GlcNAc-1β	4.69	3.70	3.68	3.66	NR	NR
GlcNAc-2	4.66	3.81	3.58	3.74	NR	NR
GlcNAc-3	4.59 (d, J = 8.1 Hz)	3.77	3.61	3.76	NR	NR
GlcNAc-4	4.55 (d, J = 8.1 Hz)	3.70	3.49	3.41	NR	NR
GlcNAc-5	4.46 (d, J = 8.2 Hz)	3.70	3.57	3.41	NR	NR
Man-1	4.70	4.18	3.87	3.55	NR	NR
Man-2	5.06	4.26	3.90	3.70	NR	NR
Man-3	5.00	4.15	3.85	3.63	NR	NR
Gal	4.55 (d, J = 8.1 Hz)	3.56	4.11	3.96	NR	NR
Fuc	4.90	3.79	3.90	3.81	4.12	1.20
Neu5Ac	-	-	H_{ax}: 1.79 (t, J = 12.1 Hz) H_{eq}: 2.75	3.68	3.84	3.63

ESI-MS: $[M+2H]^{2+}$ calcd for $C_{81}H_{136}N_6O_{58}^{2+}$, 1060.39; found: 1060.55.

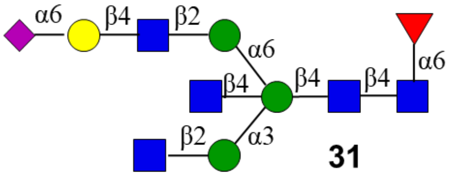

31

	H1	H2	H3	H4	H5	H6
GlcNAc-1α	5.18 (d, J = 3.1 Hz)	3.90	3.86	3.79	NR	NR
GlcNAc-1β	4.70	3.70	3.68	3.66	NR	NR
GlcNAc-2	4.67	3.81	3.58	3.74	NR	NR
GlcNAc-3	4.56 (d, J = 8.3 Hz)	3.72	3.61	3.47	NR	NR
GlcNAc-4	4.60 (d, J = 8.1 Hz)	3.74	3.56	3.69	NR	NR
GlcNAc-5	4.46	3.70	3.58	3.43	NR	NR
Man-1	4.71	4.18	3.87	3.58	NR	NR
Man-2	5.06	4.25	3.91	3.71	NR	NR
Man-3	5.02	4.14	3.85	3.63	NR	NR
Gal	4.45	3.54	3.67	3.94	NR	NR
Fuc	4.90	3.79	3.90	3.81	4.12	1.20
Neu5Ac	-	-	H_{ax}: 1.72 (t, J = 12.2 Hz) H_{eq}: 2.68	3.66	3.80	3.70

ESI-MS: $[M+2H]^{2+}$ calcd for $C_{81}H_{136}N_6O_{58}^{2+}$, 1060.39; found: 1060.30.

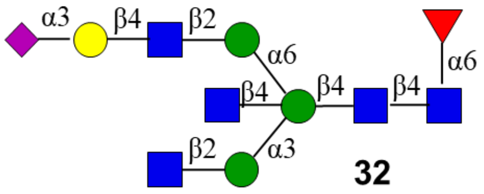

32

	H1	H2	H3	H4	H5	H6
GlcNAc-1α	5.18 (d, J = 3.2 Hz)	3.90	3.87	3.78	NR	NR
GlcNAc-1β	4.70	3.71	3.68	3.65	NR	NR
GlcNAc-2	4.66	3.82	3.58	3.74	NR	NR
GlcNAc-3	4.55	3.74	3.60	3.47	NR	NR
GlcNAc-4	4.56	3.72	3.53	3.63	NR	NR
GlcNAc-5	4.46 (d, J = 8.3 Hz)	3.70	3.57	3.41	NR	NR
Man-1	4.69	4.17	3.86	3.53	NR	NR
Man-2	5.06	4.25	3.91	3.71	NR	NR
Man-3	5.00	4.15	3.83	3.62	NR	NR
Gal	4.55	3.58	4.12	3.96	NR	NR
Fuc	4.90	3.80	3.90	3.81	4.12	1.20
Neu5Ac	-	-	H_{ax}: 1.81 (t, J = 12.1 Hz) H_{eq}: 2.76	3.69	3.85	3.64

ESI-MS: $[M+2H]^{2+}$ calcd for $C_{81}H_{136}N_6O_{58}{}^{2+}$, 1060.39; found: 1060.55.

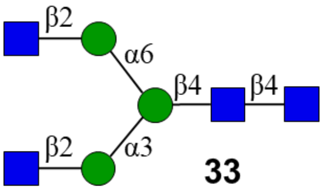

33

	H1	H2	H3	H4	H5	H6
GlcNAc-1α	5.18 (d, J = 2.4 Hz)	3.87	3.87	3.63	NR	NR
GlcNAc-1β	4.69 (d, J = 7.9 Hz)	3.68	3.63	3.51	NR	NR
GlcNAc-2	4.60	3.78	3.60	3.74	NR	NR
GlcNAc-3	4.55 (d, J = 8.4 Hz)	3.70	3.54	3.44	NR	NR
GlcNAc-4	4.55 (d, J = 8.4 Hz)	3.70	3.54	3.44	NR	NR
Man-1	4.77	4.25	3.78	3.63	NR	NR
Man-2	5.11	4.18	3.90	3.74	NR	NR
Man-3	4.92	4.10	3.89	3.62	NR	NR

ESI-MS: $[M+H]^+$ calcd for $C_{50}H_{85}N_4O_{36}^+$, 1317.49; found: 1317.10.

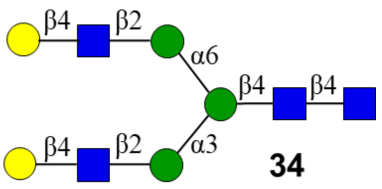

34

	H1	H2	H3	H4	H5	H6
GlcNAc-1α	5.18 (d, J = 2.6 Hz)	3.87	3.87	3.63	NR	NR
GlcNAc-1β	4.69 (d, J = 7.5 Hz)	3.68	3.63	3.51	NR	NR
GlcNAc-2	4.60	3.79	3.60	3.74	NR	NR
GlcNAc-3	4.57 (d, J = 7.9 Hz)	3.74	3.57	3.70	NR	NR
GlcNAc-4	4.57 (d, J = 7.9 Hz)	3.74	3.57	3.70	NR	NR
Man-1	4.76	4.24	3.77	3.62	NR	NR
Man-2	5.11	4.19	3.90	3.74	NR	NR
Man-3	4.92	4.10	3.89	3.61	NR	NR
Gal-1	4.47	3.54	3.67	3.92	NR	NR
Gal-2	4.47	3.54	3.67	3.92	NR	NR

ESI-MS: $[M+H]^+$ calcd for $C_{62}H_{105}N_4O_{46}^+$, 1641.60; found: 1641.15.

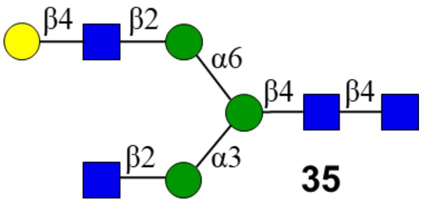

35

	H1	H2	H3	H4	H5	H6
GlcNAc-1α	5.18<br>(d, J = 2.5 Hz)	3.87	3.87	3.63	NR	NR
GlcNAc-1β	4.69<br>(d, J = 7.7 Hz)	3.69	3.63	3.52	NR	NR
GlcNAc-2	4.60	3.79	3.61	3.74	NR	NR
GlcNAc-3	4.55<br>(d, J = 8.4 Hz)	3.69	3.55	3.69	NR	NR
GlcNAc-4	4.58<br>(d, J = 8.2 Hz)	3.74	3.57	3.72	NR	NR
Man-1	4.76	4.24	3.77	3.61	NR	NR
Man-2	5.11	4.19	3.90	3.74	NR	NR
Man-3	4.92	4.11	3.88	3.61	NR	NR
Gal	4.47<br>(d, J = 7.8 Hz)	3.54	3.67	3.92	NR	NR

ESI-MS: $[M+H]^+$ calcd for $C_{56}H_{95}N_4O_{41}^+$, 1479.55; found: 1479.15.

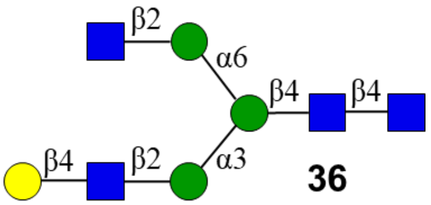

36

	H1	H2	H3	H4	H5	H6
GlcNAc-1α	5.18 (d, J = 2.5 Hz)	3.87	3.87	3.63	NR	NR
GlcNAc-1β	4.69 (d, J = 7.8 Hz)	3.69	3.62	3.52	NR	NR
GlcNAc-2	4.60	3.80	3.61	3.74	NR	NR
GlcNAc-3	4.57 (d, J = 7.3 Hz)	3.74	3.57	3.73	NR	NR
GlcNAc-4	4.55 (d, J = 8.4 Hz)	3.70	3.53	3.46	NR	NR
Man-1	4.77	4.25	3.77	3.63	NR	NR
Man-2	5.11	4.19	3.90	3.74	NR	NR
Man-3	4.91	4.10	3.89	3.61	NR	NR
Gal	4.46 (d, J = 7.8 Hz)	3.54	3.66	3.92	NR	NR

ESI-MS: $[M+H]^+$ calcd for $C_{56}H_{95}N_4O_{41}{}^+$, 1479.55; found: 1479.10.

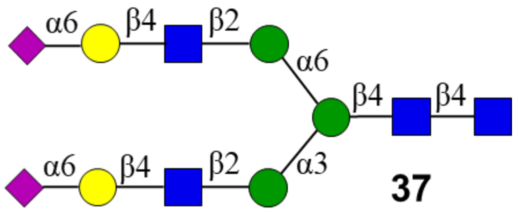

37

	H1	H2	H3	H4	H5	H6
GlcNAc-1α	5.18<br>(d, J = 2.8 Hz)	3.87	3.86	3.62	NR	NR
GlcNAc-1β	4.69<br>(d, J = 7.5 Hz)	3.68	3.62	3.51	NR	NR
GlcNAc-2	4.60	3.79	3.59	3.72	NR	NR
GlcNAc-3	4.59	3.75	3.59	3.65	NR	NR
GlcNAc-4	4.59	3.75	3.59	3.65	NR	NR
Man-1	4.77	4.25	3.79	3.63	NR	NR
Man-2	5.13	4.19	3.89	3.74	NR	NR
Man-3	4.94	4.12	3.89	3.61	NR	NR
Gal-1	4.44<br>(d, J = 7.9 Hz)	3.54	3.66	3.92	NR	NR
Gal-2	4.44<br>(d, J = 7.9 Hz)	3.54	3.66	3.92	NR	NR
Neu5Ac-1	-	-	H_{ax}: 1.71<br>(t, J = 12.1 Hz)<br>H_{eq}: 2.66	3.65	3.79	3.69
Neu5Ac-2	-	-	H_{ax}: 1.71<br>(t, J = 12.1 Hz)<br>H_{eq}: 2.66	3.65	3.79	3.69

ESI-MS: $[M+2H]^{2+}$ calcd for $C_{84}H_{140}N_6O_{62}{}^{2+}$, 1112.40; found: 1112.50.

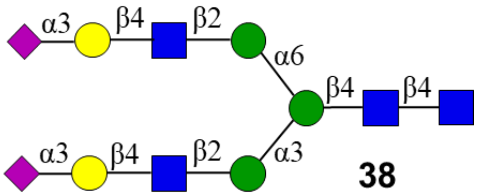

38

	H1	H2	H3	H4	H5	H6
GlcNAc-1α	5.18 (d, J = 2.7 Hz)	3.87	3.86	3.63	NR	NR
GlcNAc-1β	4.69 (d, J = 7.4 Hz)	3.69	3.63	3.51	NR	NR
GlcNAc-2	4.60	3.79	3.60	3.74	NR	NR
GlcNAc-3	4.57 (d, J = 8.0 Hz)	3.75	3.56	3.69	NR	NR
GlcNAc-4	4.57 (d, J = 8.0 Hz)	3.75	3.56	3.69	NR	NR
Man-1	4.76	4.24	3.78	3.61	NR	NR
Man-2	5.11	4.19	3.89	3.75	NR	NR
Man-3	4.92	4.11	3.87	3.61	NR	NR
Gal-1	4.54	3.56	4.11	3.95	NR	NR
Gal-2	4.54	3.56	4.11	3.95	NR	NR
Neu5Ac-1	-	-	H_{ax}: 1.79 (t, J = 12.1 Hz) H_{eq}: 2.75	3.68	3.84	3.62
Neu5Ac-2	-	-	H_{ax}: 1.79 (t, J = 12.1 Hz) H_{eq}: 2.75	3.68	3.84	3.62

ESI-MS: $[M+2H]^{2+}$ calcd for $C_{84}H_{140}N_6O_{62}^{2+}$, 1112.40; found: 1112.60.

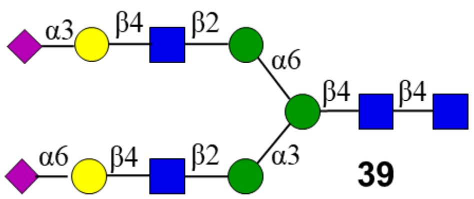

39

	H1	H2	H3	H4	H5	H6
GlcNAc-1α	5.18<br>(d, J = 2.7 Hz)	3.87	3.87	3.62	NR	NR
GlcNAc-1β	4.69<br>(d, J = 7.4 Hz)	3.69	3.62	3.51	NR	NR
GlcNAc-2	4.60	3.79	3.60	3.75	NR	NR
GlcNAc-3	4.60	3.76	3.60	3.64	NR	NR
GlcNAc-4	4.57<br>(d, J = 8.4 Hz)	3.75	3.55	3.68	NR	NR
Man-1	4.76	4.25	3.78	3.61	NR	NR
Man-2	5.13	4.19	3.89	3.74	NR	NR
Man-3	4.92	4.12	3.88	3.61	NR	NR
Gal-1	4.44<br>(d, J = 7.9 Hz)	3.51	3.66	3.92	NR	NR
Gal-2	4.54<br>(d, J = 8.1 Hz)	3.55	4.11	3.95	NR	NR
Neu5Ac-1	-	-	H_{ax}: 1.71<br>(t, J = 12.2 Hz)<br>H_{ex}: 2.66	3.64	3.79	3.69
Neu5Ac-2	-	-	H_{ax}: 1.79<br>(t, J = 12.2 Hz)<br>H_{eq}: 2.75	3.67	3.84	3.63

ESI-MS: $[M+2H]^{2+}$ calcd for $C_{84}H_{140}N_6O_{62}^{2+}$, 1112.40; found: 1112.45.

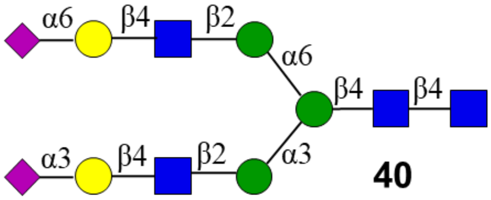

40

	H1	H2	H3	H4	H5	H6
GlcNAc-1α	5.18 (d, J = 2.6 Hz)	3.87	3.87	3.62	NR	NR
GlcNAc-1β	4.69 (d, J = 7.4 Hz)	3.69	3.61	3.51	NR	NR
GlcNAc-2	4.60	3.78	3.59	3.75	NR	NR
GlcNAc-3	4.57 (d, J = 7.6 Hz)	3.74	3.56	3.69	NR	NR
GlcNAc-4	4.60	3.75	3.61	3.65	NR	NR
Man-1	4.77	4.25	3.80	3.63	NR	NR
Man-2	5.11	4.19	3.89	3.74	NR	NR
Man-3	4.94	4.12	3.88	3.61	NR	NR
Gal-1	4.54 (d, J = 7.7 Hz)	3.56	4.11	3.95	NR	NR
Gal-2	4.44 (d, J = 7.9 Hz)	3.53	3.67	3.92	NR	NR
Neu5Ac-1	-	-	H_{ax}: 1.80 (t, J = 12.1 Hz) H_{eq}: 2.75	3.69	3.85	3.64
Neu5Ac-2	-	-	H_{ax}: 1.73 (t, J = 12.2 Hz) H_{eq}: 2.66	3.66	3.80	3.71

ESI-MS: $[M+2H]^{2+}$ calcd for $C_{84}H_{140}N_6O_{62}^{2+}$, 1112.40; found: 1112.50.

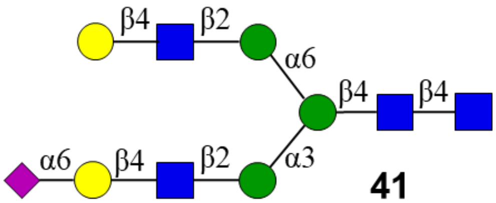

41

	H1	H2	H3	H4	H5	H6
GlcNAc-1α	5.18 (d, J = 2.7 Hz)	3.87	3.86	3.63	NR	NR
GlcNAc-1β	4.69 (d, J = 8.1 Hz)	3.69	3.62	3.52	NR	NR
GlcNAc-2	4.60	3.80	3.60	3.74	NR	NR
GlcNAc-3	4.60	3.74	3.60	3.64	NR	NR
GlcNAc-4	4.58 (d, J = 8.2 Hz)	3.74	3.57	3.71	NR	NR
Man-1	4.76	4.25	3.78	3.62	NR	NR
Man-2	5.13	4.19	3.89	3.74	NR	NR
Man-3	4.92	4.11	3.89	3.61	NR	NR
Gal-1	4.44 (d, J = 7.9 Hz)	3.53	3.66	3.92	NR	NR
Gal-2	4.47 (d, J = 7.8 Hz)	3.54	3.66	3.92	NR	NR
Neu5Ac	-	-	H_{ax}: 1.72 (t, J = 12.2 Hz) H_{eq}: 2.66	3.65	3.80	3.69

ESI-MS: $[M+2H]^{2+}$ calcd for $C_{73}H_{123}N_5O_{54}^{2+}$, 966.85; found: 966.90.

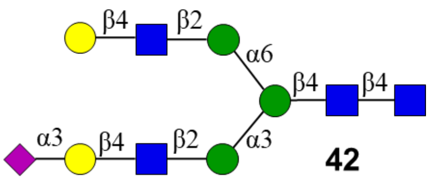

42

	H1	H2	H3	H4	H5	H6
GlcNAc-1α	5.18<br>(d, J = 3.3 Hz)	3.87	3.86	3.62	NR	NR
GlcNAc-1β	4.69<br>(d, J = 7.4 Hz)	3.69	3.62	3.51	NR	NR
GlcNAc-2	4.60	3.79	3.61	3.74	NR	NR
GlcNAc-3	4.57	3.74	3.56	3.69	NR	NR
GlcNAc-4	4.57	3.74	3.56	3.71	NR	NR
Man-1	4.76	4.24	3.77	3.62	NR	NR
Man-2	5.11	4.18	3.89	3.74	NR	NR
Man-3	4.92	4.10	3.89	3.61	NR	NR
Gal-1	4.54<br>(d, J = 7.9 Hz)	3.56	4.11	3.95	NR	NR
Gal-2	4.46<br>(d, J = 7.8 Hz)	3.54	3.66	3.92	NR	NR
Neu5Ac	-	-	H_{ax}: 1.79<br>(t, J = 12.1 Hz)<br>H_{eq}: 2.75	3.68	3.84	3.63

ESI-MS: $[M+2H]^{2+}$ calcd for $C_{73}H_{123}N_5O_{54}^{2+}$, 966.85; found: 966.95.

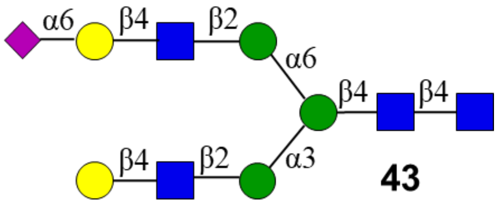

43

	H1	H2	H3	H4	H5	H6
GlcNAc-1α	5.18 (d, J = 3.1 Hz)	3.87	3.87	3.63	NR	NR
GlcNAc-1β	4.69 (d, J = 7.5 Hz)	3.69	3.62	3.51	NR	NR
GlcNAc-2	4.60	3.79	3.59	3.75	NR	NR
GlcNAc-3	4.57 (d, J = 7.5 Hz)	3.74	3.58	3.72	NR	NR
GlcNAc-4	4.60	3.75	3.61	3.65	NR	NR
Man-1	4.76	4.24	3.77	3.63	NR	NR
Man-2	5.11	4.19	3.90	3.74	NR	NR
Man-3	4.94	4.11	3.89	3.61	NR	NR
Gal-1	4.46 (d, J = 8.1 Hz)	3.54	3.66	3.92	NR	NR
Gal-2	4.44 (d, J = 7.9 Hz)	3.54	3.66	3.92	NR	NR
Neu5Ac	-	-	H_{ax}: 1.71 (t, J = 12.1 Hz) H_{eq}: 2.66	3.65	3.80	3.70

ESI-MS: $[M+2H]^{2+}$ calcd for $C_{73}H_{123}N_5O_{54}{}^{2+}$, 966.85; found: 966.70.

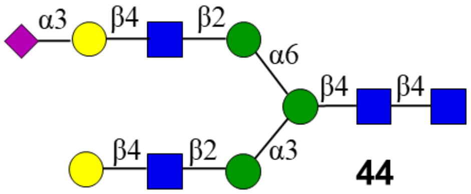

44

	H1	H2	H3	H4	H5	H6
GlcNAc-1α	5.18 (d, J = 3.1 Hz)	3.87	3.87	3.63	NR	NR
GlcNAc-1β	4.69 (d, J = 7.5 Hz)	3.69	3.62	3.51	NR	NR
GlcNAc-2	4.60	3.79	3.60	3.74	NR	NR
GlcNAc-3	4.57	3.74	3.56	3.68	NR	NR
GlcNAc-4	4.57	3.74	3.56	3.68	NR	NR
Man-1	4.76	4.24	3.78	3.61	NR	NR
Man-2	5.11	4.18	3.90	3.74	NR	NR
Man-3	4.92	4.11	3.88	3.61	NR	NR
Gal-1	4.46 (d, J = 7.8 Hz)	3.53	3.66	3.92	NR	NR
Gal-2	4.54 (d, J = 8.1 Hz)	3.57	4.11	3.96	NR	NR
Neu5Ac	-	-	H_{ax}: 1.79 (t, J = 12.1 Hz) H_{eq}: 2.75	3.68	3.84	3.63

ESI-MS: $[M+2H]^{2+}$ calcd for $C_{73}H_{123}N_5O_{54}^{2+}$, 966.85; found: 966.95.

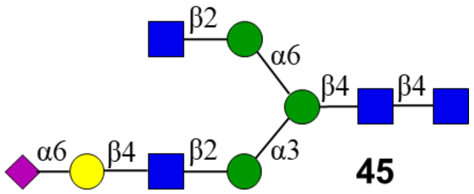

45

	H1	H2	H3	H4	H5	H6
GlcNAc-1α	5.18 (d, J = 2.4 Hz)	3.87	3.87	3.63	NR	NR
GlcNAc-1β	4.68 (d, J = 8.1 Hz)	3.69	3.62	3.51	NR	NR
GlcNAc-2	4.60	3.79	3.60	3.75	NR	NR
GlcNAc-3	4.59	3.75	3.60	3.64	NR	NR
GlcNAc-4	4.54 (d, J = 8.6 Hz)	3.70	3.53	3.44	NR	NR
Man-1	4.77	4.25	3.77	3.63	NR	NR
Man-2	5.13	4.19	3.89	3.74	NR	NR
Man-3	4.91	4.10	3.89	3.61	NR	NR
Gal	4.43 (d, J = 7.9 Hz)	3.53	3.66	3.92	NR	NR
Neu5Ac	-	-	H_{ax}: 1.72 (t, J = 12.1 Hz) H_{eq}: 2.66	3.65	3.79	3.69

ESI-MS: $[M+H]^+$ calcd for $C_{67}H_{112}N_5O_{49}^+$, 1770.64; found: 1770.20.

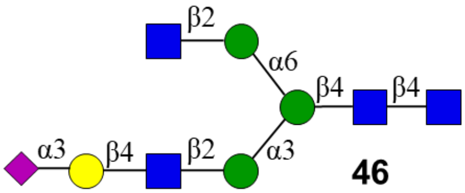

46

	H1	H2	H3	H4	H5	H6
GlcNAc-1α	5.18 (d, J = 2.7 Hz)	3.87	3.87	3.63	NR	NR
GlcNAc-1β	4.69 (d, J = 7.2 Hz)	3.69	3.62	3.51	NR	NR
GlcNAc-2	4.60	3.78	3.60	3.73	NR	NR
GlcNAc-3	4.56	3.73	3.56	3.72	NR	NR
GlcNAc-4	4.54	3.70	3.53	3.44	NR	NR
Man-1	4.76	4.24	3.77	3.62	NR	NR
Man-2	5.11	4.18	3.89	3.74	NR	NR
Man-3	4.91	4.10	3.89	3.61	NR	NR
Gal	4.54	3.56	4.11	3.95	NR	NR
Neu5Ac	-	-	H_{ax}: 1.79 (t, J = 12.1 Hz) H_{eq}: 2.75	3.68	3.84	3.63

ESI-MS: $[M+H]^+$ calcd for $C_{67}H_{112}N_5O_{49}^+$, 1770.64; found: 1770.15.

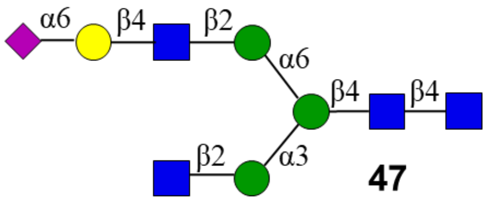

47

	H1	H2	H3	H4	H5	H6
GlcNAc-1α	5.18 (d, J = 2.6 Hz)	3.87	3.87	3.62	NR	NR
GlcNAc-1β	4.69 (d, J = 7.5 Hz)	3.69	3.62	3.51	NR	NR
GlcNAc-2	4.60	3.79	3.61	3.75	NR	NR
GlcNAc-3	4.55 (d, J = 8.4 Hz)	3.69	3.55	3.44	NR	NR
GlcNAc-4	4.60	3.75	3.58	3.65	NR	NR
Man-1	4.76	4.24	3.77	3.63	NR	NR
Man-2	5.11	4.18	3.90	3.74	NR	NR
Man-3	4.94	4.11	3.88	3.61	NR	NR
Gal	4.54 (d, J = 8.1 Hz)	3.57	4.11	3.96	NR	NR
Neu5Ac	-	-	H_{ax}: 1.71 (t, J = 12.2 Hz) H_{eq}: 2.66	3.65	3.79	3.69

ESI-MS: $[M+H]^+$ calcd for $C_{67}H_{112}N_5O_{49}^+$, 1770.64; found: 1770.15.

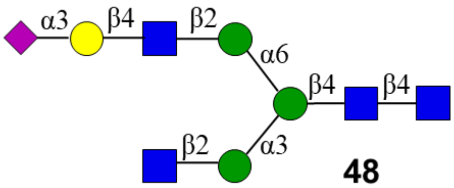

48

	H1	H2	H3	H4	H5	H6
GlcNAc-1α	5.18<br>(d, J = 2.4 Hz)	3.87	3.87	3.62	NR	NR
GlcNAc-1β	4.69<br>(d, J = 8.2 Hz)	3.68	3.62	3.51	NR	NR
GlcNAc-2	4.60	3.78	3.61	3.74	NR	NR
GlcNAc-3	4.54<br>(d, J = 8.4 Hz)	3.69	3.55	3.44	NR	NR
GlcNAc-4	4.56<br>(d, J = 8.5 Hz)	3.72	3.56	3.68	NR	NR
Man-1	4.76	4.24	3.77	3.60	NR	NR
Man-2	5.11	4.18	3.90	3.74	NR	NR
Man-3	4.92	4.11	3.88	3.61	NR	NR
Gal	4.54<br>(d, J = 8.4 Hz)	3.56	4.11	3.96	NR	NR
Neu5Ac	-	-	H_{ax}: 1.79<br>(t, J = 12.1 Hz)<br>H_{eq}: 2.75	3.68	3.85	3.63

ESI-MS: $[M+H]^+$ calcd for $C_{67}H_{112}N_5O_{49}^+$, 1770.64; found: 1770.20.

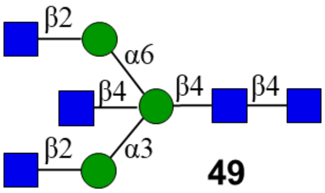

49

	H1	H2	H3	H4	H5	H6
GlcNAc-1α	5.18 (d, J = 2.5 Hz)	3.86	3.86	3.62	NR	NR
GlcNAc-1β	4.69	3.68	3.65	3.50	NR	NR
GlcNAc-2	4.60	3.80	3.58	3.73	NR	NR
GlcNAc-3	4.55 (d, J = 8.3 Hz)	3.69	3.59	3.47	NR	NR
GlcNAc-4	4.54 (d, J = 8.2 Hz)	3.69	3.47	3.40	NR	NR
GlcNAc-5	4.46 (d, J = 8.4 Hz)	3.69	3.56	3.41	NR	NR
Man-1	4.69	4.17	3.87	3.54	NR	NR
Man-2	5.05	4.24	3.90	3.69	NR	NR
Man-3	5.00	4.14	3.83	3.62	NR	NR

ESI-MS: $[M+H]^+$ calcd for $C_{58}H_{98}N_5O_{41}{}^+$, 1520.57; found: 1520.15.

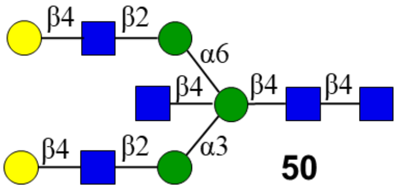

50

	H1	H2	H3	H4	H5	H6
GlcNAc-1α	5.18<br>(d, J = 2.6 Hz)	3.88	3.86	3.64	NR	NR
GlcNAc-1β	4.69	3.68	3.65	3.50	NR	NR
GlcNAc-2	4.59	3.83	3.58	3.71	NR	NR
GlcNAc-3	4.57	3.74	3.62	3.70	NR	NR
GlcNAc-4	4.57	3.74	3.51	3.62	NR	NR
GlcNAc-5	4.45	3.68	3.54	3.40	NR	NR
Man-1	4.68	4.17	3.85	3.52	NR	NR
Man-2	5.05	4.25	3.89	3.68	NR	NR
Man-3	5.01	4.14	3.82	3.61	NR	NR
Gal-1	4.46	3.52	3.67	3.92	NR	NR
Gal-2	4.46	3.52	3.67	3.92	NR	NR

ESI-MS: $[M+2H]^{2+}$ calcd for $C_{70}H_{119}N_5O_{51}{}^{2+}$, 922.84; found: 922.90.

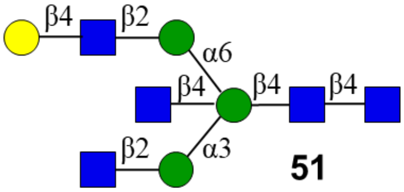

51

	H1	H2	H3	H4	H5	H6
GlcNAc-1α	5.18<br>(d, J = 2.6 Hz)	3.87	3.86	3.64	NR	NR
GlcNAc-1β	4.69	3.68	3.66	3.53	NR	NR
GlcNAc-2	4.60	3.80	3.59	3.73	NR	NR
GlcNAc-3	4.55	3.70	3.54	3.46	NR	NR
GlcNAc-4	4.58	3.74	3.51	3.63	NR	NR
GlcNAc-5	4.46	3.68	3.56	3.40	NR	NR
Man-1	4.68	4.17	3.86	3.53	NR	NR
Man-2	5.05	4.24	3.91	3.68	NR	NR
Man-3	5.01	4.14	3.82	3.63	NR	NR
Gal	4.47	3.53	3.67	3.93	NR	NR

ESI-MS: $[M+H]^+$ calcd for $C_{64}H_{108}N_5O_{46}^+$, 1682.63; found: 1682.15.

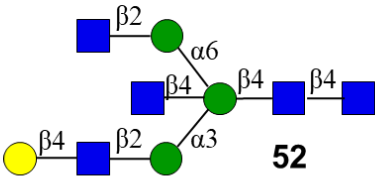

52

	H1	H2	H3	H4	H5	H6
GlcNAc-1α	5.18<br>(d, *J* = 2.5 Hz)	3.87	3.86	3.65	NR	NR
GlcNAc-1β	4.69	3.68	3.65	3.51	NR	NR
GlcNAc-2	4.60	3.80	3.59	3.73	NR	NR
GlcNAc-3	4.58	3.75	3.60	3.73	NR	NR
GlcNAc-4	4.54<br>(d, *J* = 8.4 Hz)	3.68	3.48	3.40	NR	NR
GlcNAc-5	4.46	3.68	3.53	3.40	NR	NR
Man-1	4.69	4.17	3.86	3.54	NR	NR
Man-2	5.05	4.25	3.91	3.70	NR	NR
Man-3	5.00	4.14	3.83	3.62	NR	NR
Gal	4.46	3.52	3.67	3.92	NR	NR

ESI-MS: $[M+H]^+$ calcd for $C_{64}H_{108}N_5O_{46}^+$, 1682.63; found: 1682.10.

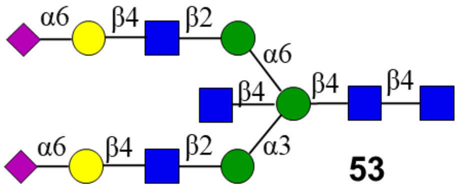

53

	H1	H2	H3	H4	H5	H6
GlcNAc-1α	5.18 (d, J = 2.7 Hz)	3.88	3.85	3.64	NR	NR
GlcNAc-1β	4.68	3.69	3.66	3.50	NR	NR
GlcNAc-2	4.60	3.83	3.60	3.73	NR	NR
GlcNAc-3	4.61	3.75	3.63	3.76	NR	NR
GlcNAc-4	4.58	3.73	3.56	3.68	NR	NR
GlcNAc-5	4.46	3.69	3.56	3.41	NR	NR
Man-1	4.70	4.18	3.87	3.55	NR	NR
Man-2	5.07	4.26	3.91	3.65	NR	NR
Man-3	5.02	4.15	3.83	3.62	NR	NR
Gal-1	4.44	3.54	3.66	3.93	NR	NR
Gal-2	4.44	3.54	3.66	3.93	NR	NR
Neu5Ac-1	-	-	H_{ax}: 1.71 (t, J = 12.2 Hz) H_{eq}: 2.67	3.64	3.80	3.70
Neu5Ac-2	-	-	H_{ax}: 1.71 (t, J = 12.2 Hz) H_{eq}: 2.67	3.64	3.80	3.70

ESI-MS: $[M+2H]^{2+}$ calcd for $C_{92}H_{153}N_7O_{67}^{2+}$, 1213.94; found: 1213.90.

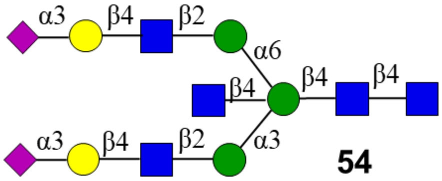

54

	H1	H2	H3	H4	H5	H6
GlcNAc-1α	5.18 (d, J = 2.5 Hz)	3.87	3.85	3.64	NR	NR
GlcNAc-1β	4.69	3.69	3.65	3.50	NR	NR
GlcNAc-2	4.59	3.82	3.61	3.71	NR	NR
GlcNAc-3	4.58	3.73	3.56	3.72	NR	NR
GlcNAc-4	4.55	3.73	3.56	3.71	NR	NR
GlcNAc-5	4.45 (d, J = 8.2 Hz)	3.68	3.56	3.41	NR	NR
Man-1	4.68	4.16	3.85	3.51	NR	NR
Man-2	5.05	4.25	3.91	3.69	NR	NR
Man-3	4.99	4.15	3.81	3.62	NR	NR
Gal-1	4.54	3.56	4.11	3.96	NR	NR
Gal-2	4.54	3.56	4.11	3.96	NR	NR
Neu5Ac-1	-	-	H_{ax}: 1.79 (t, J = 12.1 Hz) H_{eq}: 2.75	3.67	3.84	3.63
Neu5Ac-2	-	-	H_{ax}: 1.79 (t, J = 12.1 Hz) H_{eq}: 2.75	3.67	3.84	3.63

ESI-MS: $[M+2H]^{2+}$ calcd for $C_{92}H_{153}N_7O_{67}^{2+}$, 1213.94; found: 1213.80.

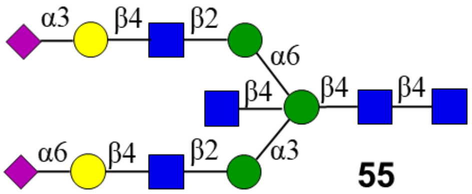

55

	H1	H2	H3	H4	H5	H6
GlcNAc-1α	5.18 (d, J = 2.5 Hz)	3.87	3.85	3.64	NR	NR
GlcNAc-1β	4.69	3.69	3.65	3.51	NR	NR
GlcNAc-2	4.60	3.81	3.58	3.73	NR	NR
GlcNAc-3	4.61	3.76	3.63	3.73	NR	NR
GlcNAc-4	4.55	3.73	3.57	3.72	NR	NR
GlcNAc-5	4.45 (d, J = 8.5 Hz)	3.68	3.56	3.39	NR	NR
Man-1	4.68	4.17	3.87	3.51	NR	NR
Man-2	5.07	4.25	3.90	3.65	NR	NR
Man-3	4.99	4.15	3.82	3.61	NR	NR
Gal-1	4.43 (d, J = 7.9 Hz)	3.53	3.65	3.92	NR	NR
Gal-2	4.54	3.57	4.11	3.95	NR	NR
Neu5Ac-1	-	-	H_{ax}: 1.72 (t, J = 11.9 Hz) H_{eq}: 2.67	3.65	3.80	3.70
Neu5Ac-2	-	-	H_{ax}: 1.79 (t, J = 12.0 Hz) H_{eq}: 2.75	3.68	3.84	3.63

ESI-MS: $[M+2H]^{2+}$ calcd for $C_{92}H_{153}N_7O_{67}^{2+}$, 1213.94; found: 1214.00.

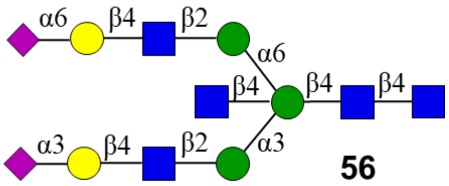

56

	H1	H2	H3	H4	H5	H6
GlcNAc-1α	5.18 (d, J = 2.6 Hz)	3.88	3.86	3.63	NR	NR
GlcNAc-1β	4.68	3.69	3.65	3.51	NR	NR
GlcNAc-2	4.60	3.83	3.60	3.75	NR	NR
GlcNAc-3	4.58	3.74	3.59	3.72	NR	NR
GlcNAc-4	4.59	3.71	3.57	3.67	NR	NR
GlcNAc-5	4.45	3.69	3.53	3.40	NR	NR
Man-1	4.69	4.17	3.86	3.54	NR	NR
Man-2	5.05	4.25	3.91	3.64	NR	NR
Man-3	5.02	4.15	3.83	3.62	NR	NR
Gal-1	4.54 (d, J = 7.8 Hz)	3.55	4.11	3.95	NR	NR
Gal-2	4.44	3.54	3.65	3.93	NR	NR
Neu5Ac-1	-	-	H_{ax}: 1.79 (t, J = 12.1 Hz) H_{eq}: 2.75	3.67	3.83	3.62
Neu5Ac-2	-	-	H_{ax}: 1.71 (t, J = 12.2 Hz) H_{eq}: 2.68	3.65	3.79	3.70

ESI-MS: $[M+2H]^{2+}$ calcd for $C_{92}H_{153}N_7O_{67}{}^{2+}$, 1213.94; found: 1213.75.

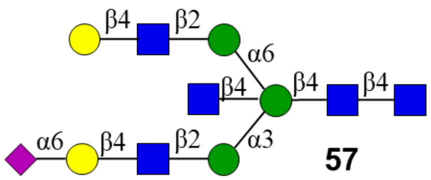

57

	H1	H2	H3	H4	H5	H6
GlcNAc-1α	5.18 (d, J = 2.7 Hz)	3.88	3.87	3.64	NR	NR
GlcNAc-1β	4.69	3.70	3.66	3.51	NR	NR
GlcNAc-2	4.60	3.80	3.58	3.73	NR	NR
GlcNAc-3	4.61	3.78	3.64	3.72	NR	NR
GlcNAc-4	4.57 (d, J = 8.4 Hz)	3.74	3.53	3.63	NR	NR
GlcNAc-5	4.46 (d, J = 7.8 Hz)	3.68	3.57	3.39	NR	NR
Man-1	4.69	4.18	3.86	3.54	NR	NR
Man-2	5.07	4.26	3.91	3.59	NR	NR
Man-3	5.01	4.14	3.82	3.62	NR	NR
Gal-1	4.44 (d, J = 7.9 Hz)	3.53	3.65	3.92	NR	NR
Gal-2	4.47 (d, J = 7.7 Hz)	3.54	3.67	3.93	NR	NR
Neu5Ac	-	-	H_{ax}: 1.72 (t, J = 12.1 Hz) H_{eq}: 2.67	3.65	3.80	3.70

ESI-MS: $[M+2H]^{2+}$ calcd for $C_{81}H_{136}N_6O_{59}^{2+}$, 1068.39; found: 1068.50.

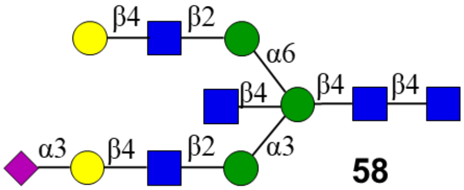

58

	H1	H2	H3	H4	H5	H6
GlcNAc-1α	5.18<br>(d, J = 2.7 Hz)	3.87	3.87	3.64	NR	NR
GlcNAc-1β	4.69	3.70	3.67	3.51	NR	NR
GlcNAc-2	4.61	3.82	3.59	3.74	NR	NR
GlcNAc-3	4.58	3.74	3.61	3.73	NR	NR
GlcNAc-4	4.57	3.74	3.53	3.65	NR	NR
GlcNAc-5	4.45<br>(d, J = 8.6 Hz)	3.69	3.54	3.39	NR	NR
Man-1	4.68	4.17	3.85	3.53	NR	NR
Man-2	5.05	4.25	3.89	3.59	NR	NR
Man-3	5.01	4.14	3.83	3.62	NR	NR
Gal-1	4.54<br>(d, J = 7.8 Hz)	3.56	4.11	3.95	NR	NR
Gal-2	4.47<br>(d, J = 7.9 Hz)	3.55	3.66	3.93	NR	NR
Neu5Ac	-	-	H_{ax}: 1.79<br>(t, J = 12.1 Hz)<br>H_{eq}: 2.75	3.66	3.83	3.62

ESI-MS: $[M+2H]^{2+}$ calcd for $C_{81}H_{136}N_6O_{59}^{2+}$, 1068.39; found: 1068.45.

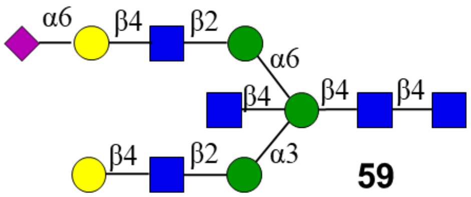

59

	H1	H2	H3	H4	H5	H6
GlcNAc-1α	5.18<br>(d, J = 2.5 Hz)	3.88	3.87	3.65	NR	NR
GlcNAc-1β	4.68	3.69	3.67	3.52	NR	NR
GlcNAc-2	4.60	3.82	3.58	3.73	NR	NR
GlcNAc-3	4.57	3.75	3.61	3.72	NR	NR
GlcNAc-4	4.59	3.72	3.57	3.66	NR	NR
GlcNAc-5	4.46	3.69	3.54	3.42	NR	NR
Man-1	4.69	4.17	3.86	3.55	NR	NR
Man-2	5.05	4.25	3.91	3.71	NR	NR
Man-3	5.02	4.14	3.83	3.62	NR	NR
Gal-1	4.46	3.53	3.67	3.93	NR	NR
Gal-2	4.44	3.53	3.67	3.93	NR	NR
Neu5Ac	-	-	H_{ax}: 1.71<br>(t, J = 12.2 Hz)<br>H_{eq}: 2.67	3.65	3.79	3.70

ESI-MS: $[M+2H]^{2+}$ calcd for $C_{81}H_{136}N_6O_{59}{}^{2+}$, 1068.39; found: 1068.55.

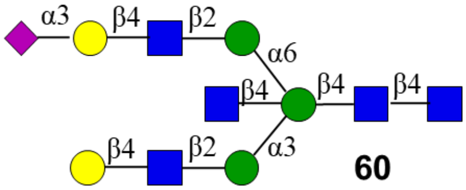

60

	H1	H2	H3	H4	H5	H6
GlcNAc-1α	5.18 (d, J = 2.5 Hz)	3.86	3.87	3.65	NR	NR
GlcNAc-1β	4.69	3.68	3.67	3.53	NR	NR
GlcNAc-2	4.59	3.83	3.59	3.76	NR	NR
GlcNAc-3	4.58	3.77	3.63	3.75	NR	NR
GlcNAc-4	4.55	3.72	3.56	3.75	NR	NR
GlcNAc-5	4.45	3.68	3.54	3.40	NR	NR
Man-1	4.68	4.17	3.85	3.52	NR	NR
Man-2	5.05	4.25	3.92	3.71	NR	NR
Man-3	5.00	4.15	3.82	3.62	NR	NR
Gal-1	4.46	3.53	3.67	3.93	NR	NR
Gal-2	4.55	3.56	4.11	3.95	NR	NR
Neu5Ac	-	-	H_{ax}: 1.79 (t, J = 12.1Hz) H_{eq}: 2.75	3.68	3.84	3.63

ESI-MS: $[M+2H]^{2+}$ calcd for $C_{81}H_{136}N_6O_{59}{}^{2+}$, 1068.39; found: 1068.20.

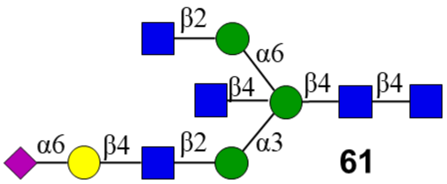

61

	H1	H2	H3	H4	H5	H6
GlcNAc-1α	5.18 (d, J = 2.7 Hz)	3.87	3.87	3.65	NR	NR
GlcNAc-1β	4.69	3.69	3.66	3.51	NR	NR
GlcNAc-2	4.60	3.81	3.58	3.73	NR	NR
GlcNAc-3	4.61	3.77	3.63	3.75	NR	NR
GlcNAc-4	4.54 (d, J = 8.4 Hz)	3.70	3.49	3.40	NR	NR
GlcNAc-5	4.46 (d, J = 8.3 Hz)	3.69	3.56	3.41	NR	NR
Man-1	4.69	4.18	3.87	3.55	NR	NR
Man-2	5.07	4.26	3.91	3.59	NR	NR
Man-3	5.00	4.14	3.83	3.62	NR	NR
Gal	4.44 (d, J = 8.0 Hz)	3.53	3.66	3.92	NR	NR
Neu5Ac	-	-	H_{ax}: 1.72 (t, J = 12.1 Hz) H_{eq}: 2.67	3.65	3.80	3.70

ESI-MS: $[M+2H]^{2+}$ calcd for $C_{75}H_{126}N_6O_{54}{}^{2+}$, 987.36; found: 987.45.

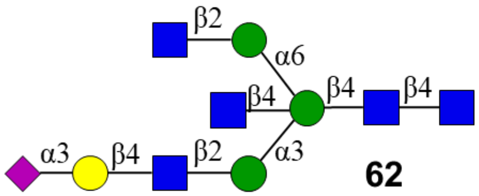

62

	H1	H2	H3	H4	H5	H6
GlcNAc-1α	5.18 (d, J = 2.6 Hz)	3.86	3.87	3.64	NR	NR
GlcNAc-1β	4.69	3.68	3.66	3.48	NR	NR
GlcNAc-2	4.60	3.80	3.59	3.74	NR	NR
GlcNAc-3	4.58	3.76	3.60	3.75	NR	NR
GlcNAc-4	4.54	3.68	3.47	3.39	NR	NR
GlcNAc-5	4.45 (d, J = 8.3 Hz)	3.68	3.56	3.40	NR	NR
Man-1	4.69	4.17	3.86	3.54	NR	NR
Man-2	5.05	4.25	3.90	3.59	NR	NR
Man-3	4.99	4.14	3.83	3.62	NR	NR
Gal	4.54	3.55	4.11	3.94	NR	NR
Neu5Ac	-	-	H_{ax}: 1.79 (t, J = 12.2 Hz) H_{eq}: 2.75	3.68	3.83	3.62

ESI-MS: $[M+2H]^{2+}$ calcd for $C_{75}H_{126}N_6O_{54}^{2+}$, 987.36; found: 987.40.

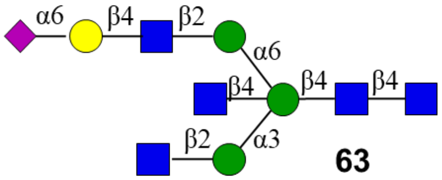

63

	H1	H2	H3	H4	H5	H6
GlcNAc-1α	5.18 (d, J = 2.5 Hz)	3.86	3.86	3.64	NR	NR
GlcNAc-1β	4.69	3.69	3.67	3.51	NR	NR
GlcNAc-2	4.60	3.82	3.59	3.73	NR	NR
GlcNAc-3	4.55 (d, J = 8.3 Hz)	3.70	3.59	3.71	NR	NR
GlcNAc-4	4.59	3.71	3.56	3.69	NR	NR
GlcNAc-5	4.46	3.68	3.57	3.41	NR	NR
Man-1	4.69	4.17	3.86	3.55	NR	NR
Man-2	5.05	4.24	3.91	3.58	NR	NR
Man-3	5.02	4.14	3.83	3.62	NR	NR
Gal	4.45	3.52	3.67	3.94	NR	NR
Neu5Ac	-	-	H_{ax}: 1.71 (t, J = 12.2 Hz) H_{eq}: 2.67	3.65	3.80	3.69

ESI-MS: $[M+2H]^{2+}$ calcd for $C_{75}H_{126}N_6O_{54}{}^{2+}$, 987.36; found: 987.45.

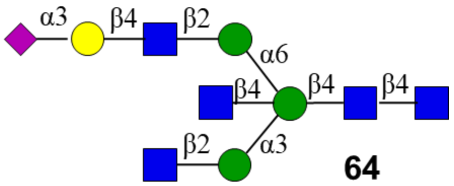

64

	H1	H2	H3	H4	H5	H6
GlcNAc-1α	5.18<br>(d, J = 2.9 Hz)	3.86	3.87	3.65	NR	NR
GlcNAc-1β	4.69	3.68	3.67	3.51	NR	NR
GlcNAc-2	4.59	3.80	3.58	3.75	NR	NR
GlcNAc-3	4.55	3.71	3.60	3.44	NR	NR
GlcNAc-4	4.55	3.71	3.57	3.74	NR	NR
GlcNAc-5	4.45<br>(d, J = 8.3 Hz)	3.68	3.56	3.39	NR	NR
Man-1	4.68	4.16	3.85	3.51	NR	NR
Man-2	5.05	4.24	3.91	3.58	NR	NR
Man-3	5.00	4.14	3.81	3.62	NR	NR
Gal-2	4.55	3.56	4.11	3.94	NR	NR
Neu5Ac	-	-	H_{ax}: 1.80<br>(t, J = 12.1Hz)<br>H_{eq}: 2.75	3.67	3.84	3.63

ESI-MS: $[M+2H]^{2+}$ calcd for $C_{75}H_{126}N_6O_{54}^{2+}$, 987.36; found: 987.40.

11. References

[1] a) K. W. Moremen, A. Ramiah, M. Stuart, J. Steel, L. Meng, F. Forouhar, H. A. Moniz, G. Gahlay, Z. Gao, D. Chapla, S. Wang, J.-Y. Yang, P. K. Prabhakar, R. Johnson, M. d. Rosa, C. Geisler, A. V. Nairn, J. Seetharaman, S.-C. Wu, L. Tong, H. J. Gilbert, J. LaBaer, D. L. Jarvis, *Nat. Chem. Biol.* **2018**, *14*, 156-162; b) L. Liu, A. R. Prudden, C. J. Capicciotti, G. P. Bosman, J.-Y. Yang, D. G. Chapla, K. W. Moremen, G.-J. Boons, *Nat. Chem.* **2019**, *11*, 161-169; c) A. Y. Mehta, R. K. H. Veeraiah, S. Dutta, C. K. Goth, M. S. Hanes, C. Gao, K. Stavenhagen, R. Kardish, Y. Matsumoto, J. Heimburg-Molinaro, M. Boyce, N. L. B. Pohl, R. D. Cummings, *Cell Chem. Biol.* **2020**, *27*, 1207-1219; d) Y. Ihara, A. Nishikawa, T. Tohma, H. Soejima, N. Niikawa, N. Taniguchi, *J. Biochem.* **1993**, *113*, 692-698; e) T. Li, M. Huang, L. Liu, S. Wang, K. W. Moremen, G. J. Boons, *Chem. Eur. J.* **2016**, *22*, 18742-18746.

[2] O. Bohorov, H. Andersson-Sand, J. Hoffmann, O. Blixt, *Glycobiology* **2006**, *16*, 21C-27C.

[3] T. Li, M. A. Wolfert, N. Wei, R. Huizinga, B. C. Jacobs, G. J. Boons, *J. Am. Chem. Soc.* **2020**, *142*, 19611-19621.

12. NMR spectra

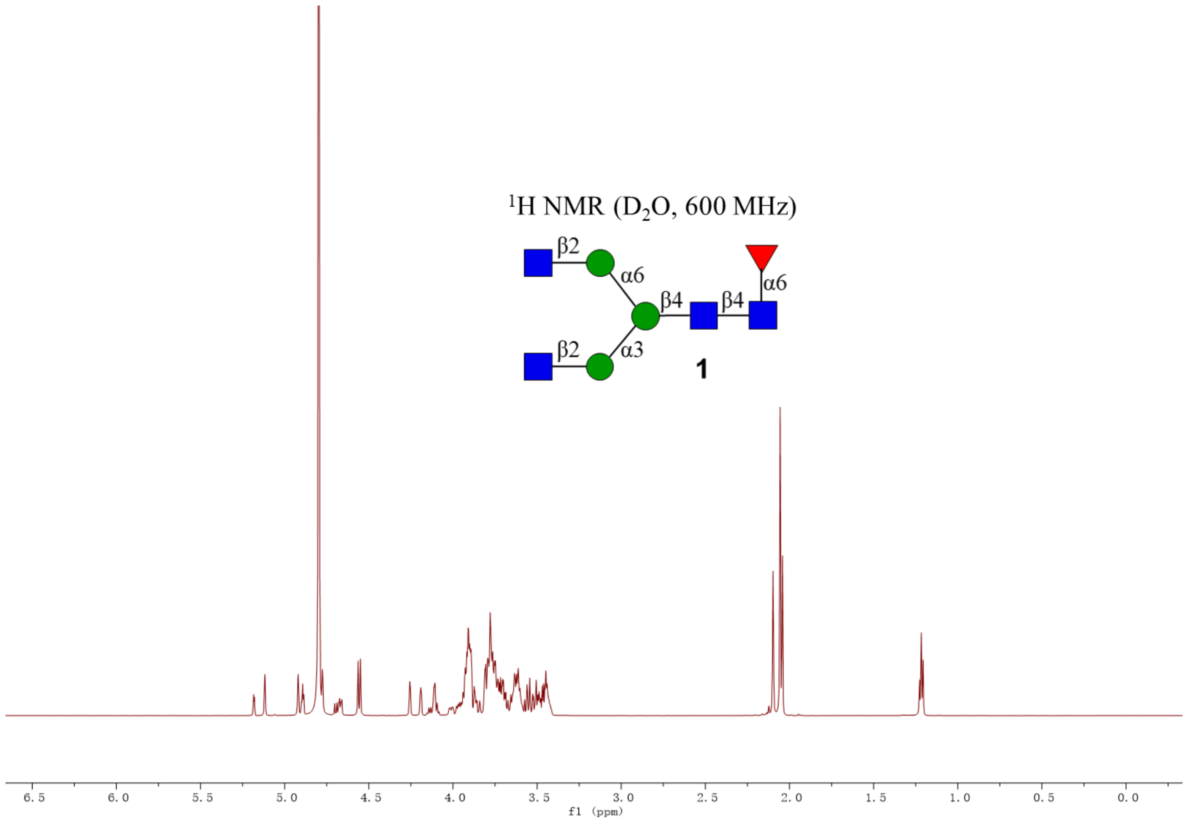

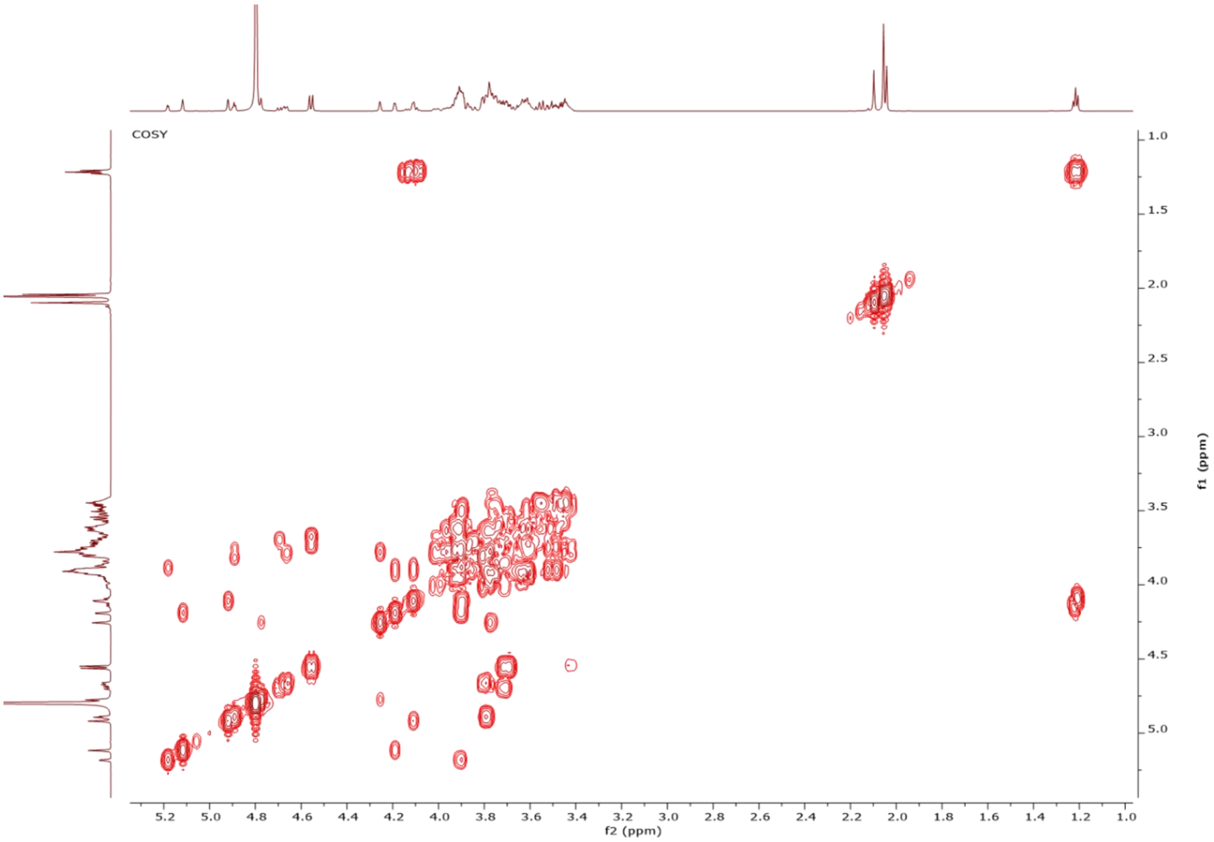

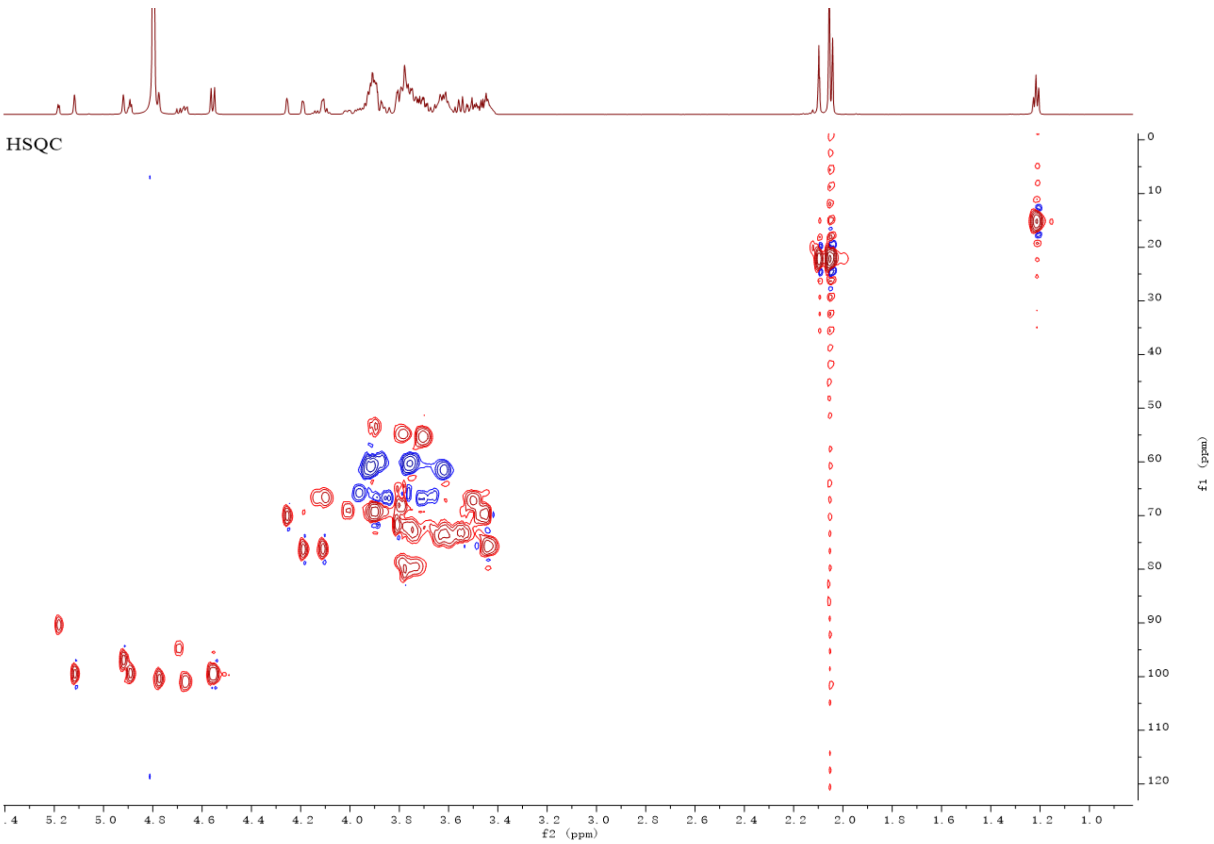
HSQC
f2 (ppm)
f1 (ppm)

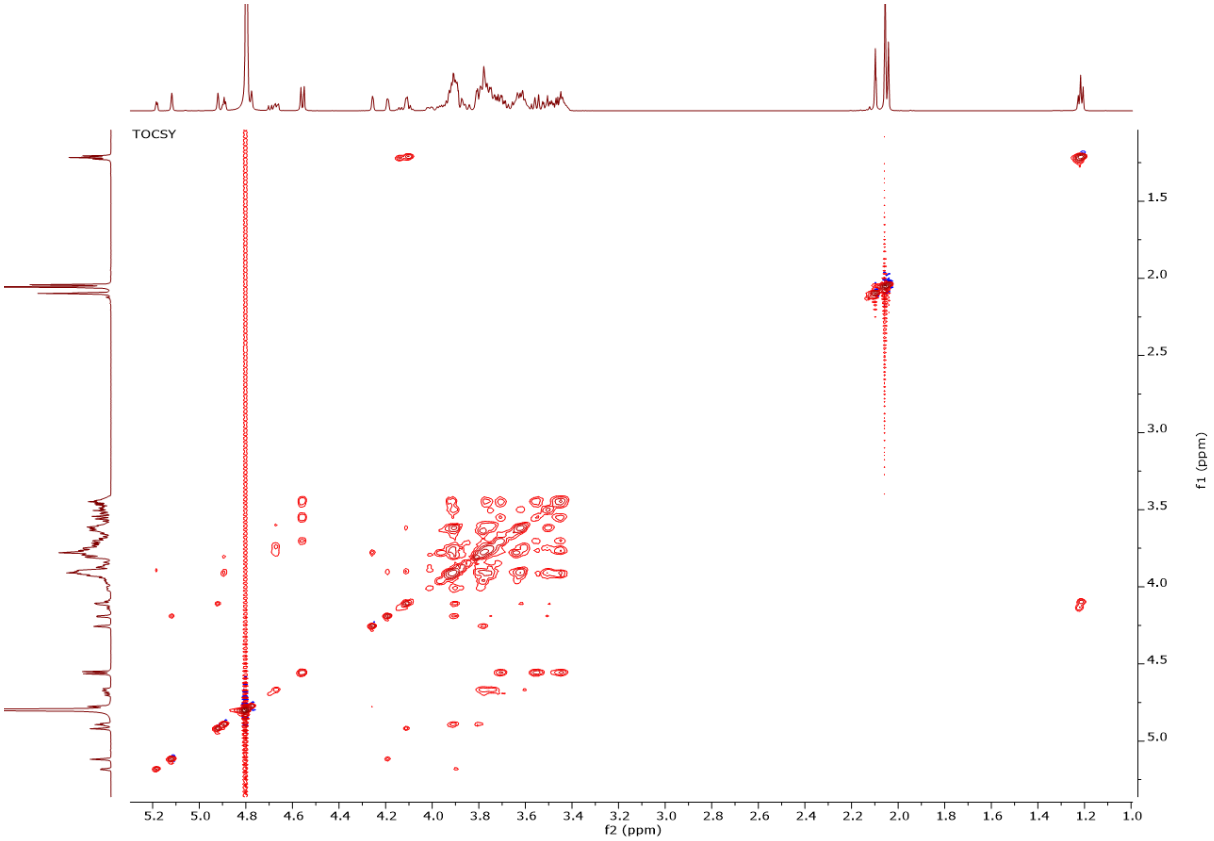
TOCSY
f2 (ppm)
f1 (ppm)

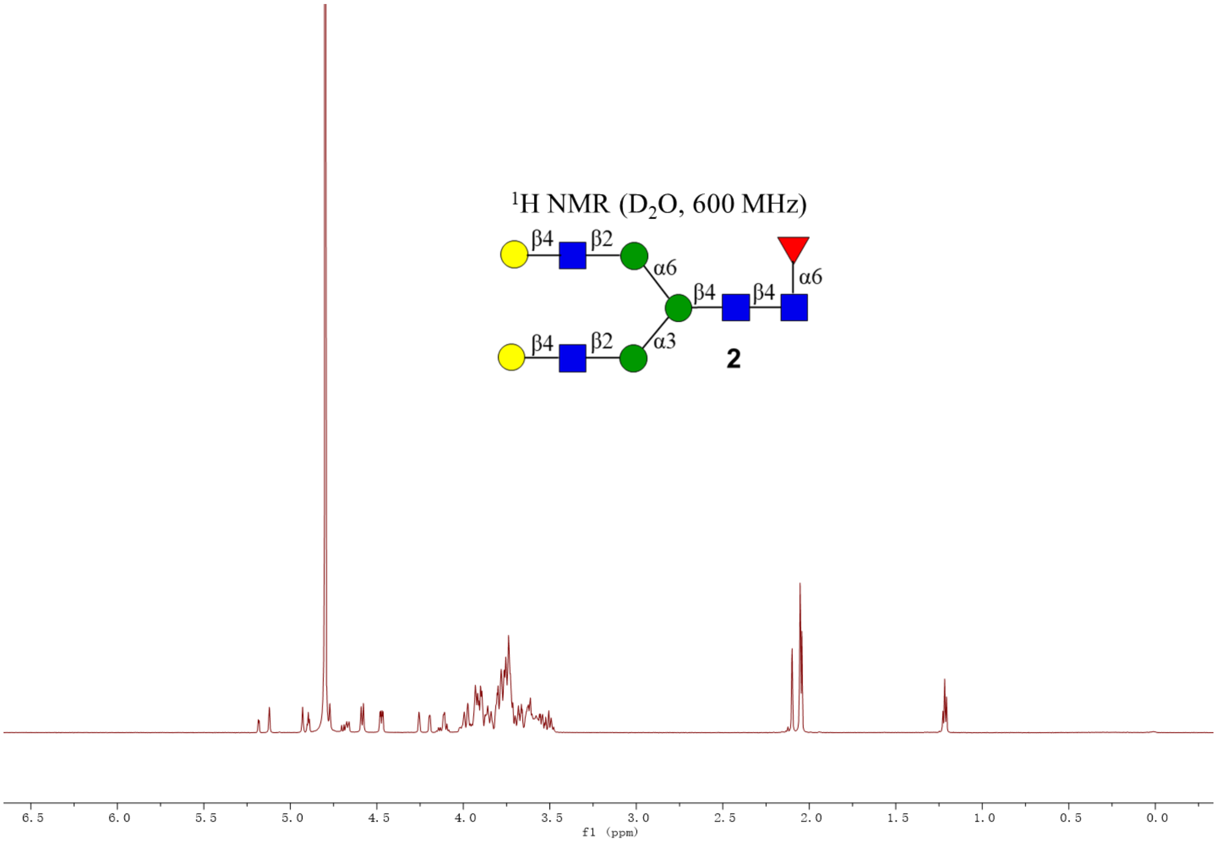
^{1}H NMR (D_2O, 600 MHz)
β4
β2
α6
β4
β4
α6
β4
β2
α3
2
6.5 6.0 5.5 5.0 4.5 4.0 3.5 3.0 2.5 2.0 1.5 1.0 0.5 0.0
f1 (ppm)

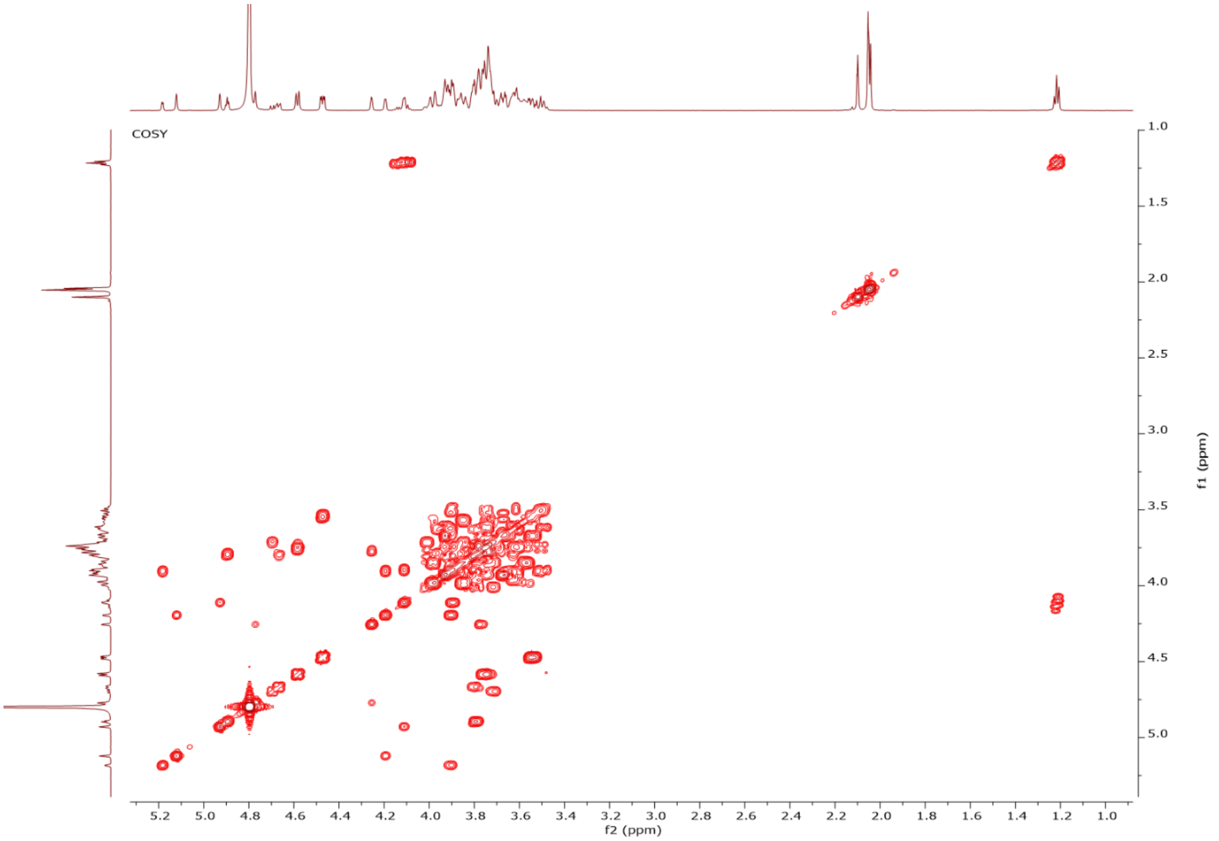
COSY
f1 (ppm)
f2 (ppm)
5.2 5.0 4.8 4.6 4.4 4.2 4.0 3.8 3.6 3.4 3.2 3.0 2.8 2.6 2.4 2.2 2.0 1.8 1.6 1.4 1.2 1.0
1.0 1.5 2.0 2.5 3.0 3.5 4.0 4.5 5.0

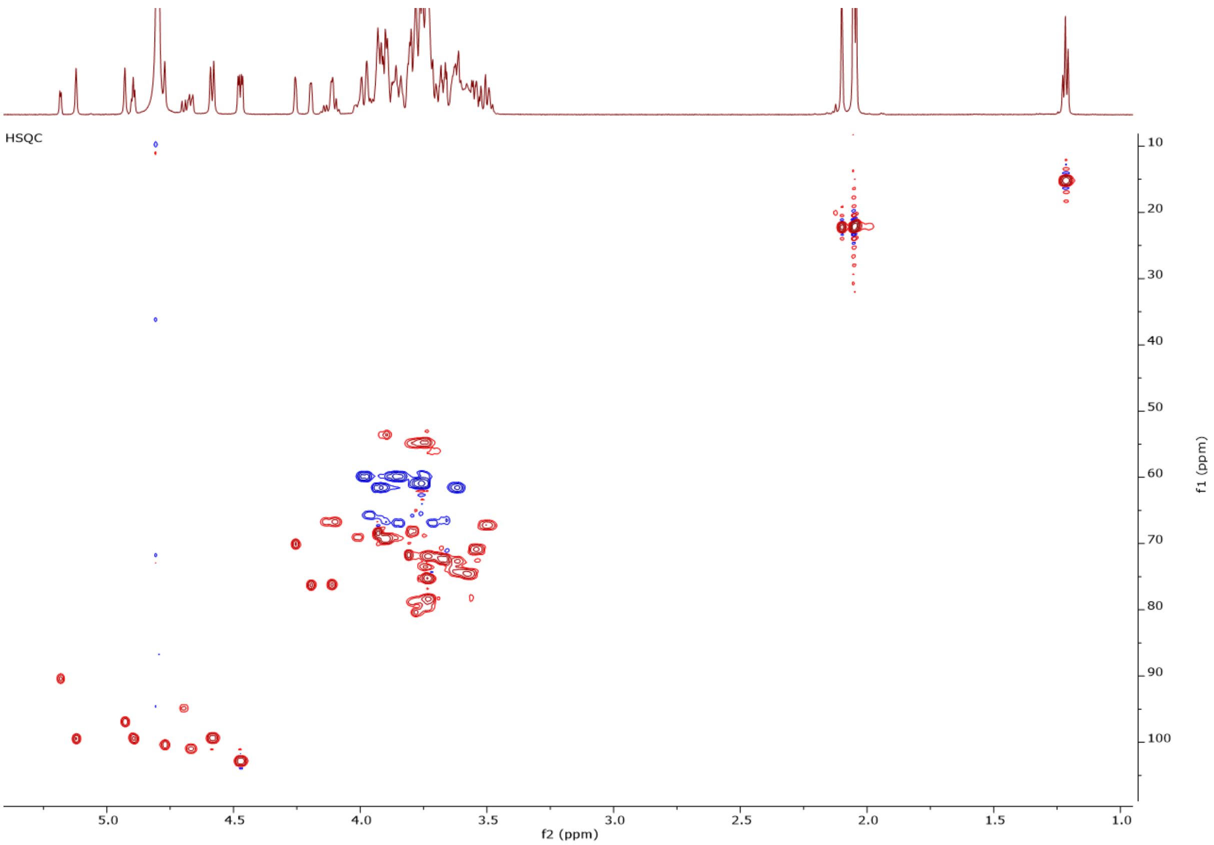

HSQC
f2 (ppm)
f1 (ppm)

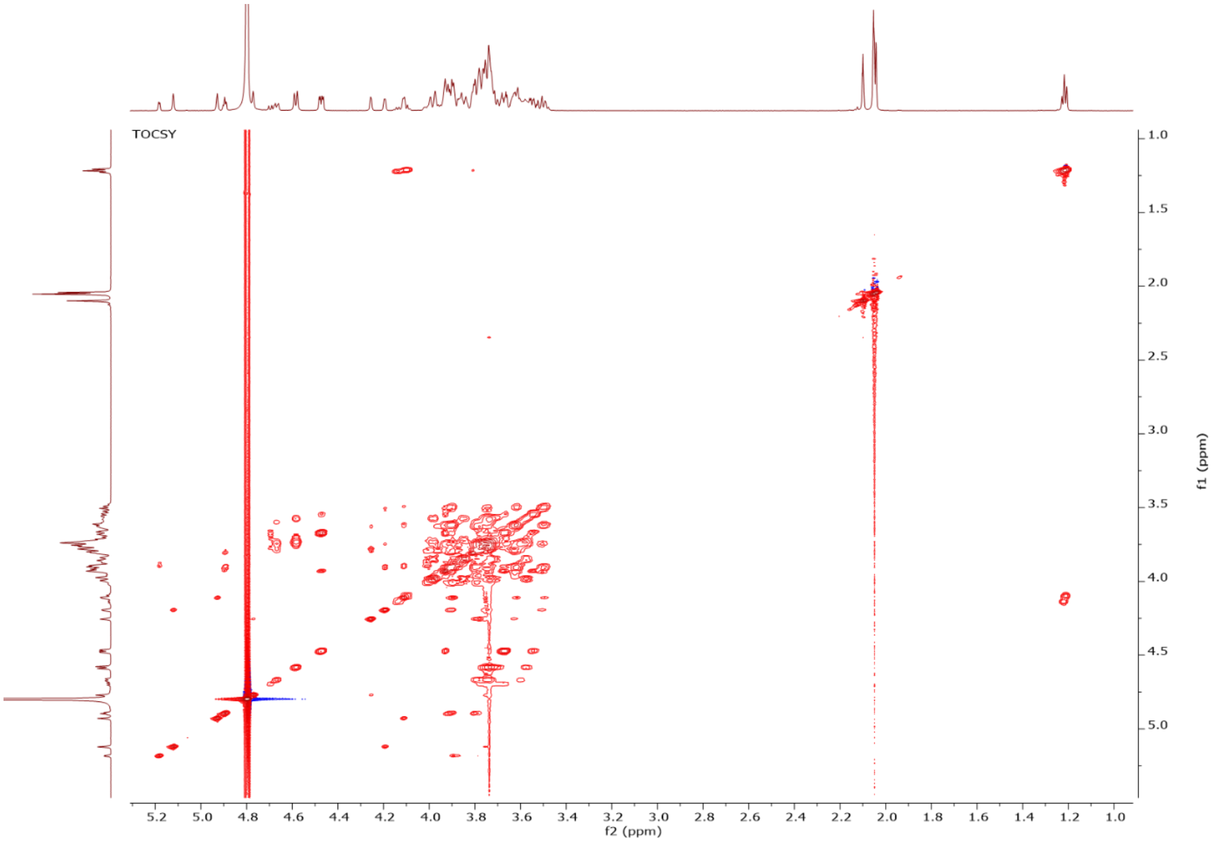

TOCSY
f2 (ppm)
f1 (ppm)

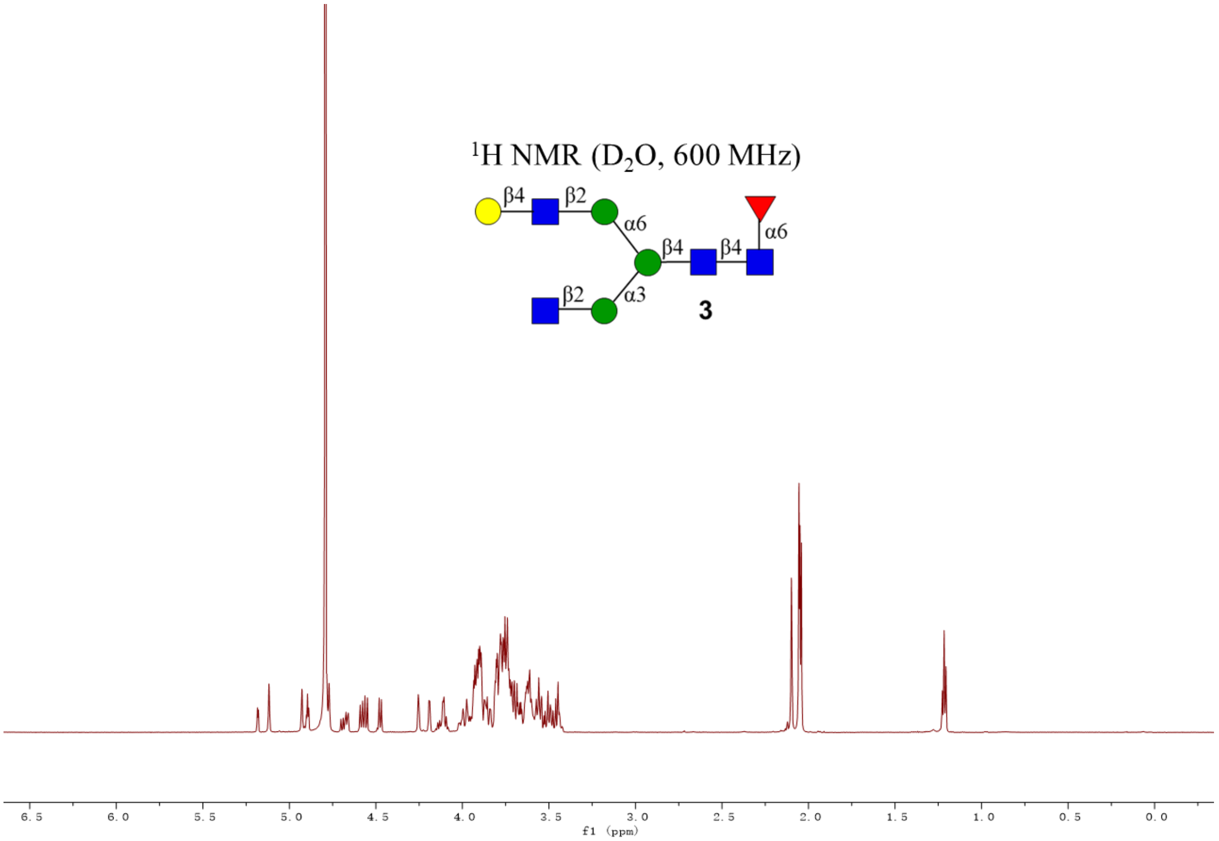

^{1}H NMR (D_2O, 600 MHz)
β4
β2
α6
β4
β4
α6
β2
α3
3
f1 (ppm)

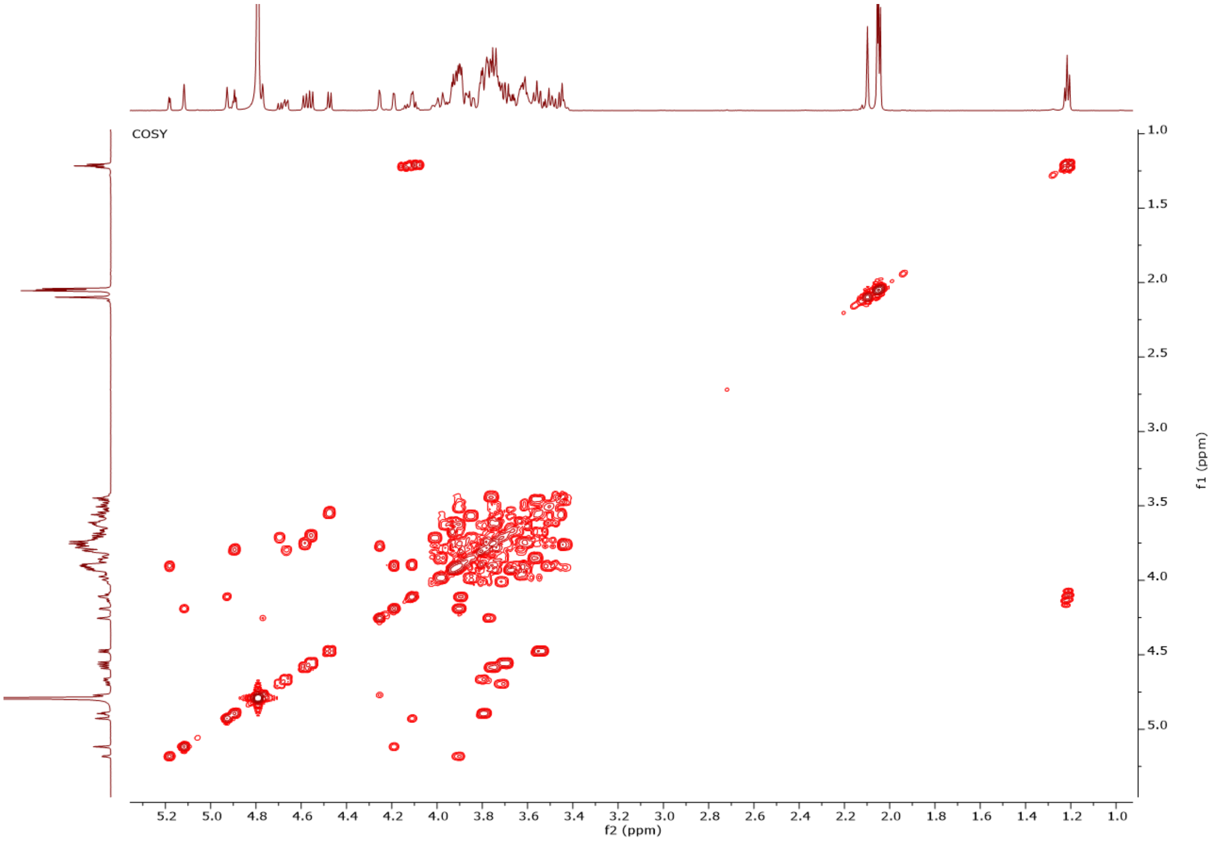

COSY
f2 (ppm)
f1 (ppm)

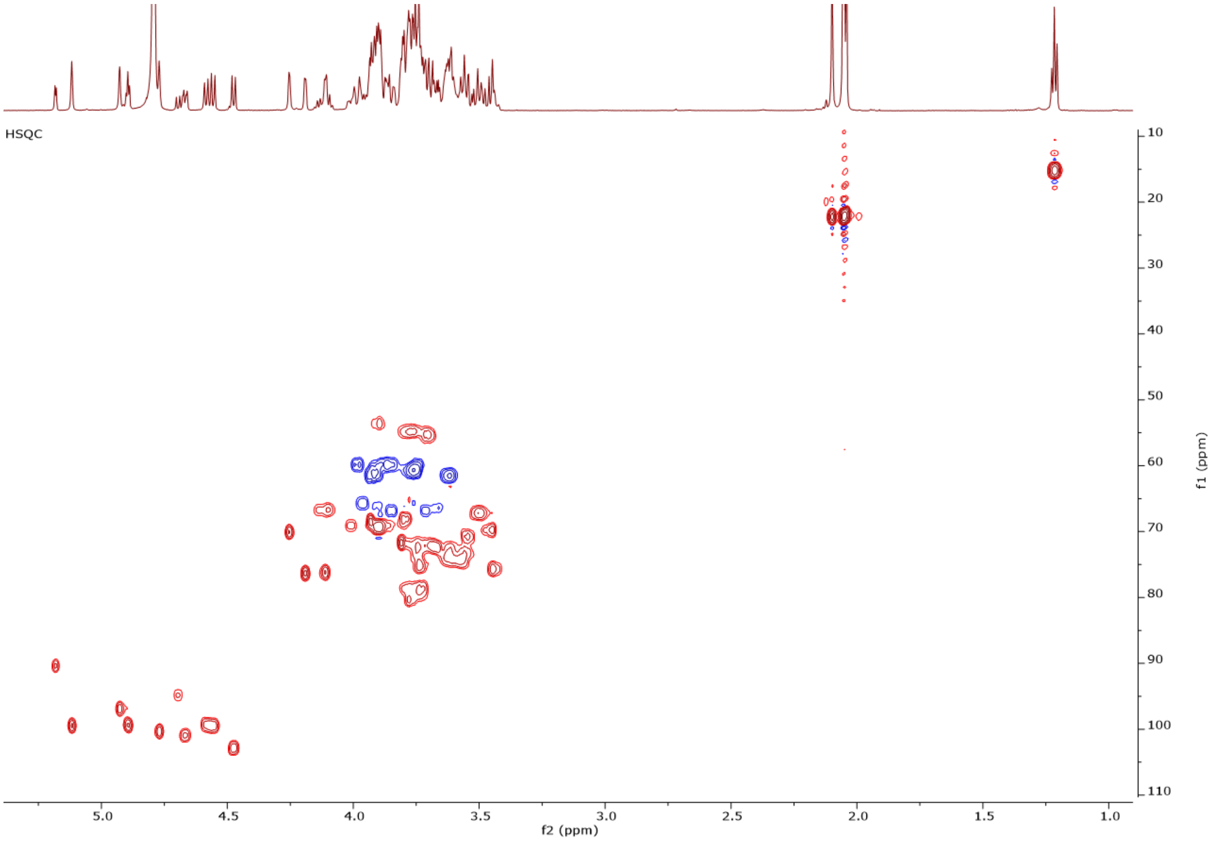
HSQC
f2 (ppm)
f1 (ppm)

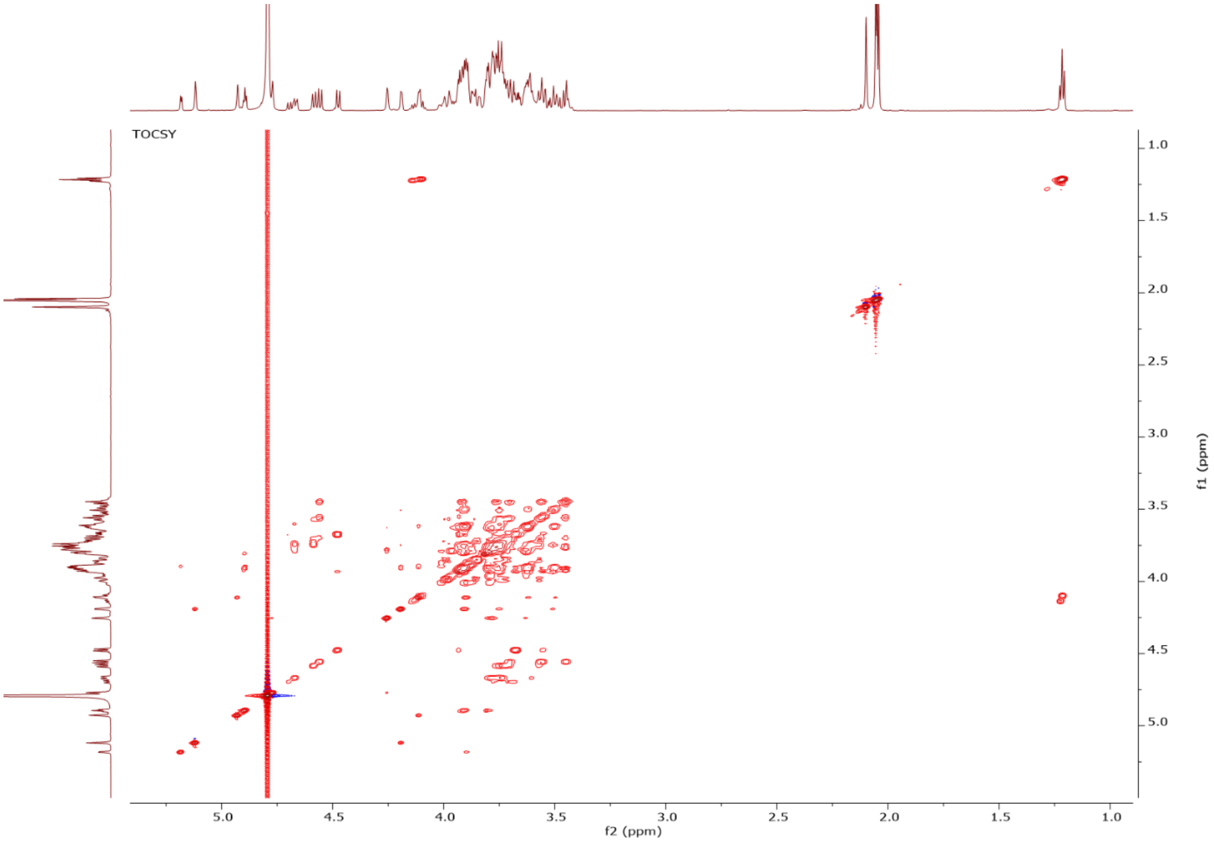
TOCSY
f2 (ppm)
f1 (ppm)

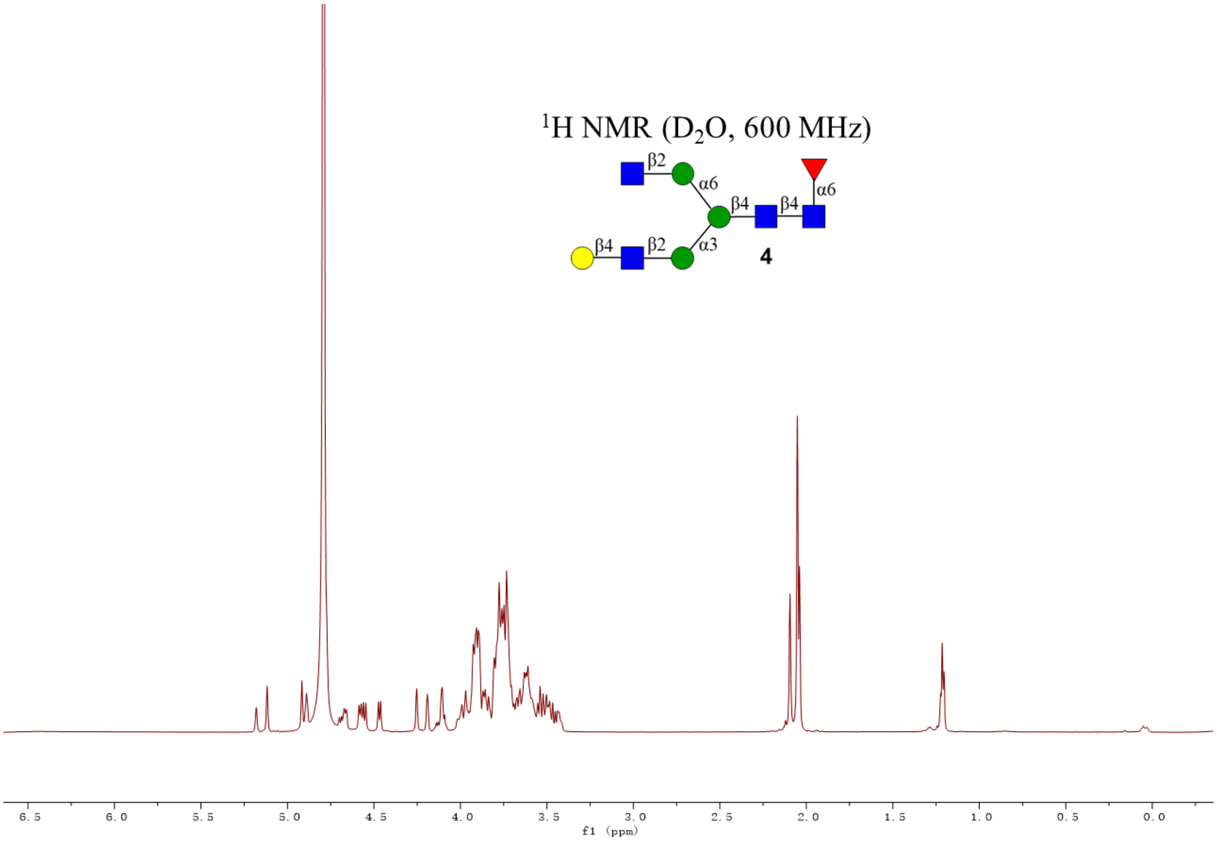
¹H NMR (D₂O, 600 MHz)
β2
α6
β4
β4
α6
β4
β2
α3
4
6.5
6.0
5.5
5.0
4.5
4.0
3.5
3.0
2.5
2.0
1.5
1.0
0.5
0.0
f1 (ppm)

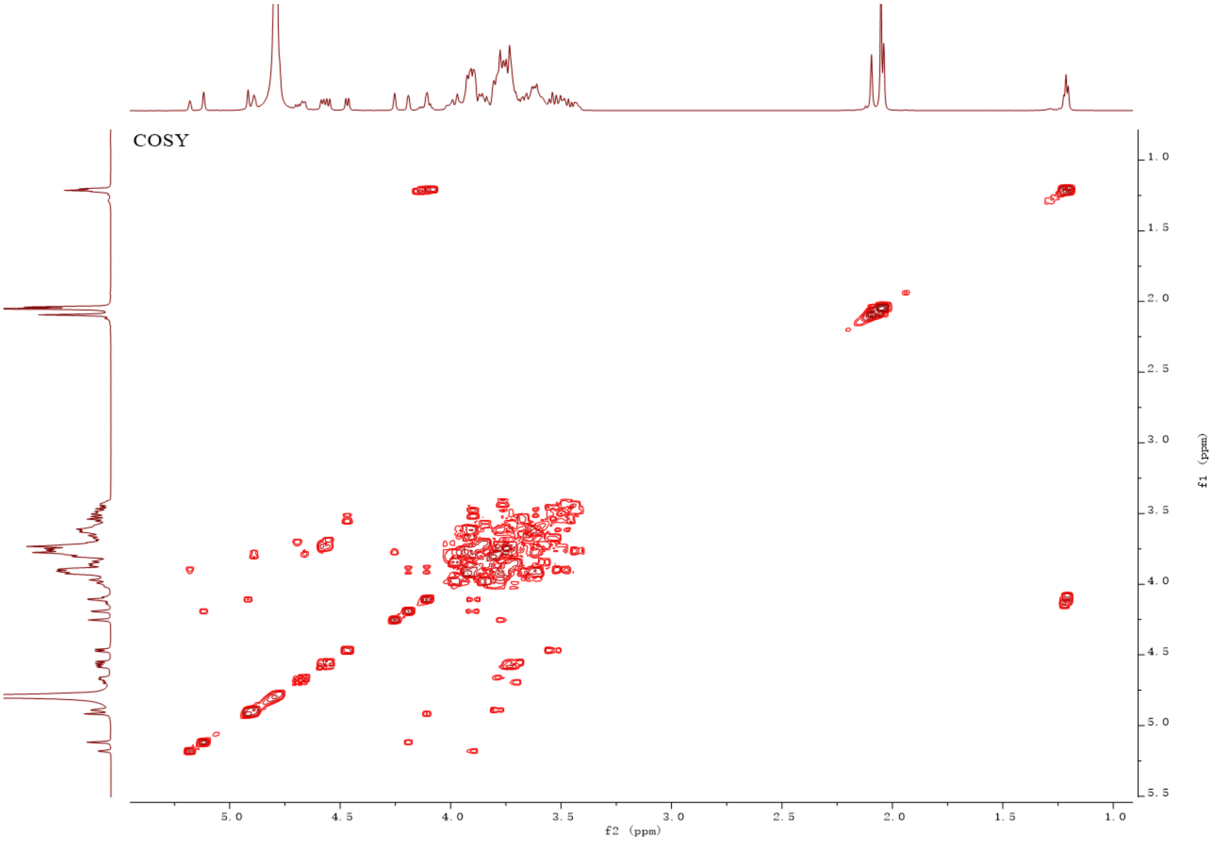
COSY
5.0
4.5
4.0
3.5
3.0
2.5
2.0
1.5
1.0
f2 (ppm)
f1 (ppm)
5.5

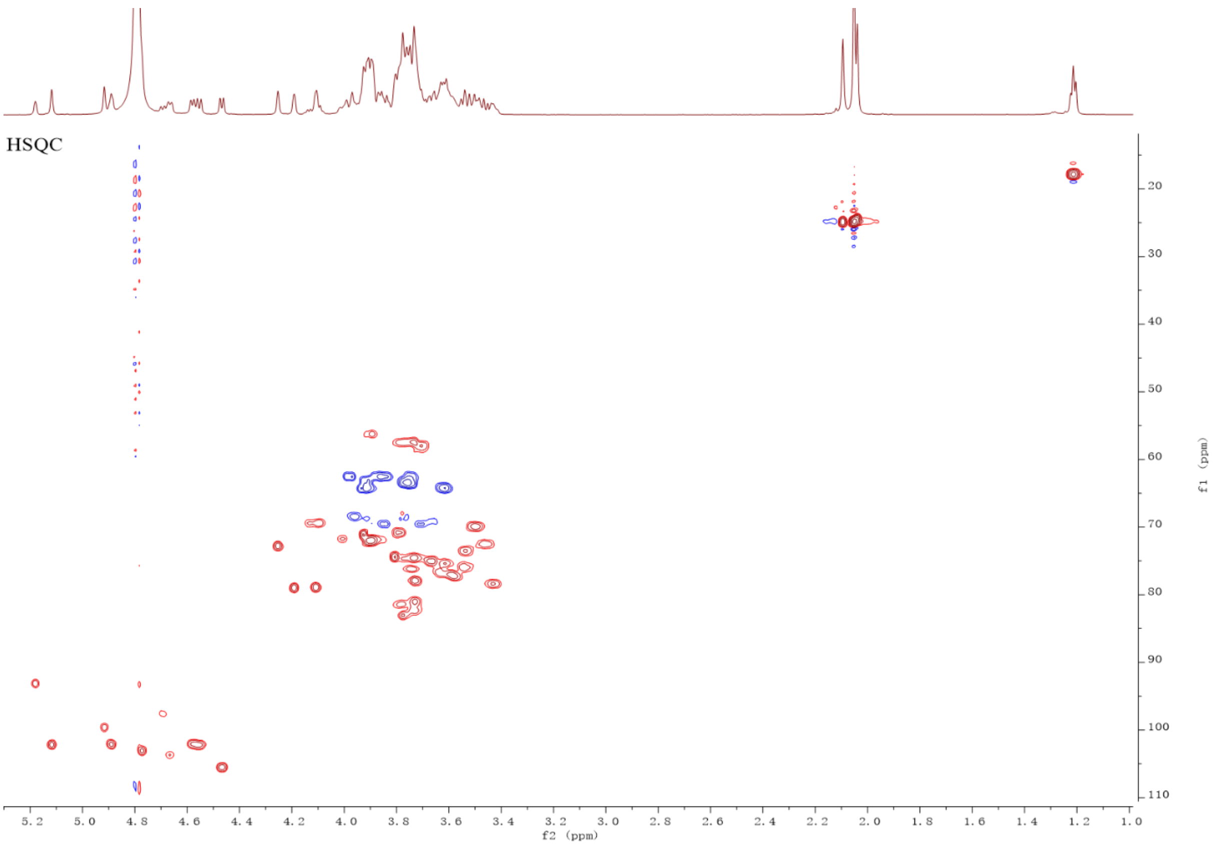
HSQC
f2 (ppm)
f1 (ppm)

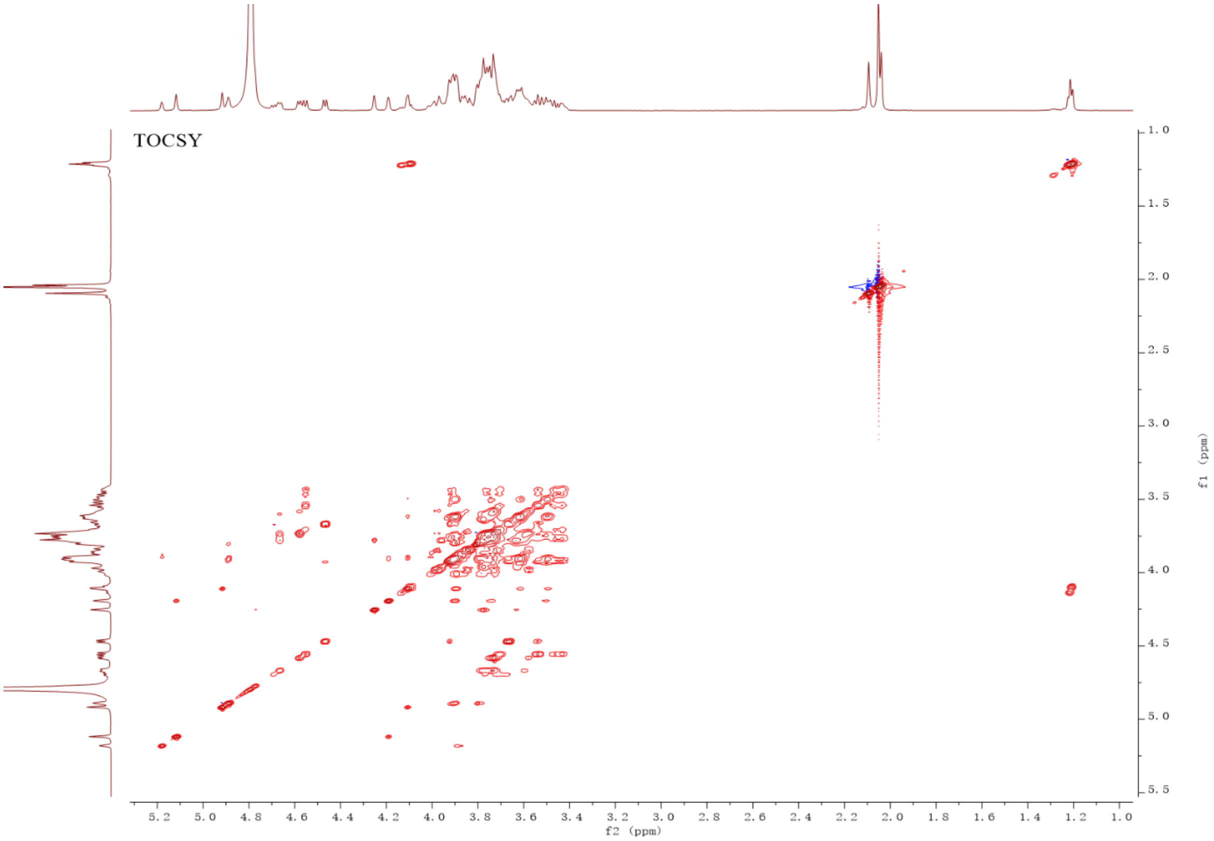
TOCSY
f2 (ppm)
f1 (ppm)

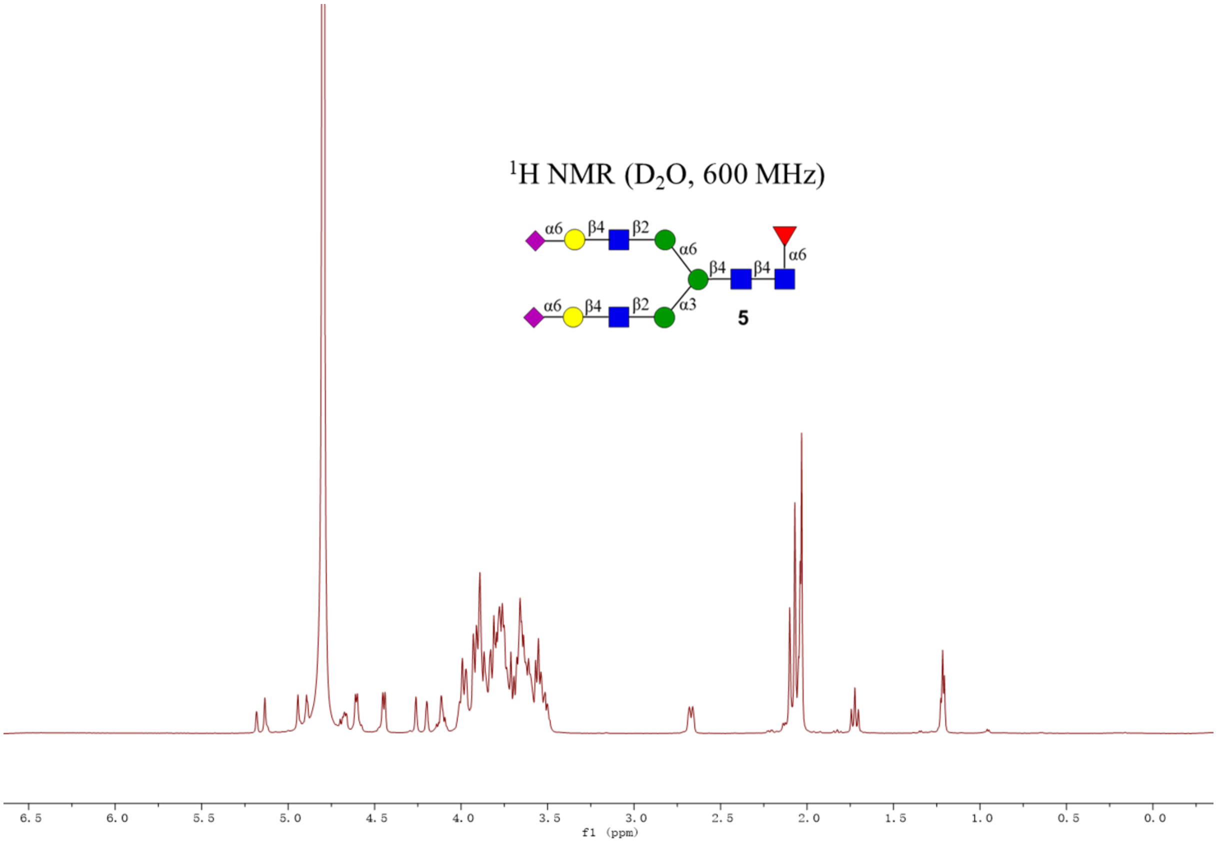

^{1}H NMR (D_2O, 600 MHz)
α6
β4
β2
α6
β4
β4
α6
α6
β4
β2
α3
5
f1 (ppm)

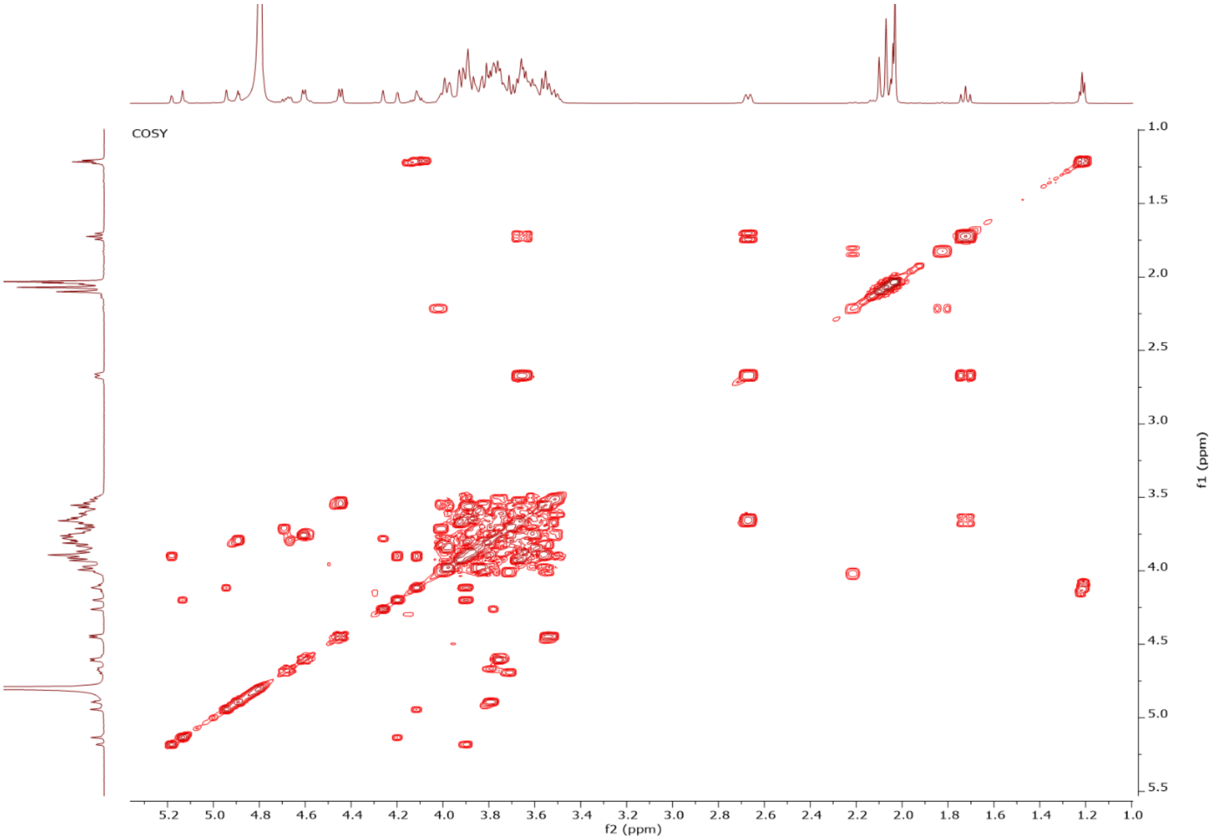

COSY
f2 (ppm)
f1 (ppm)

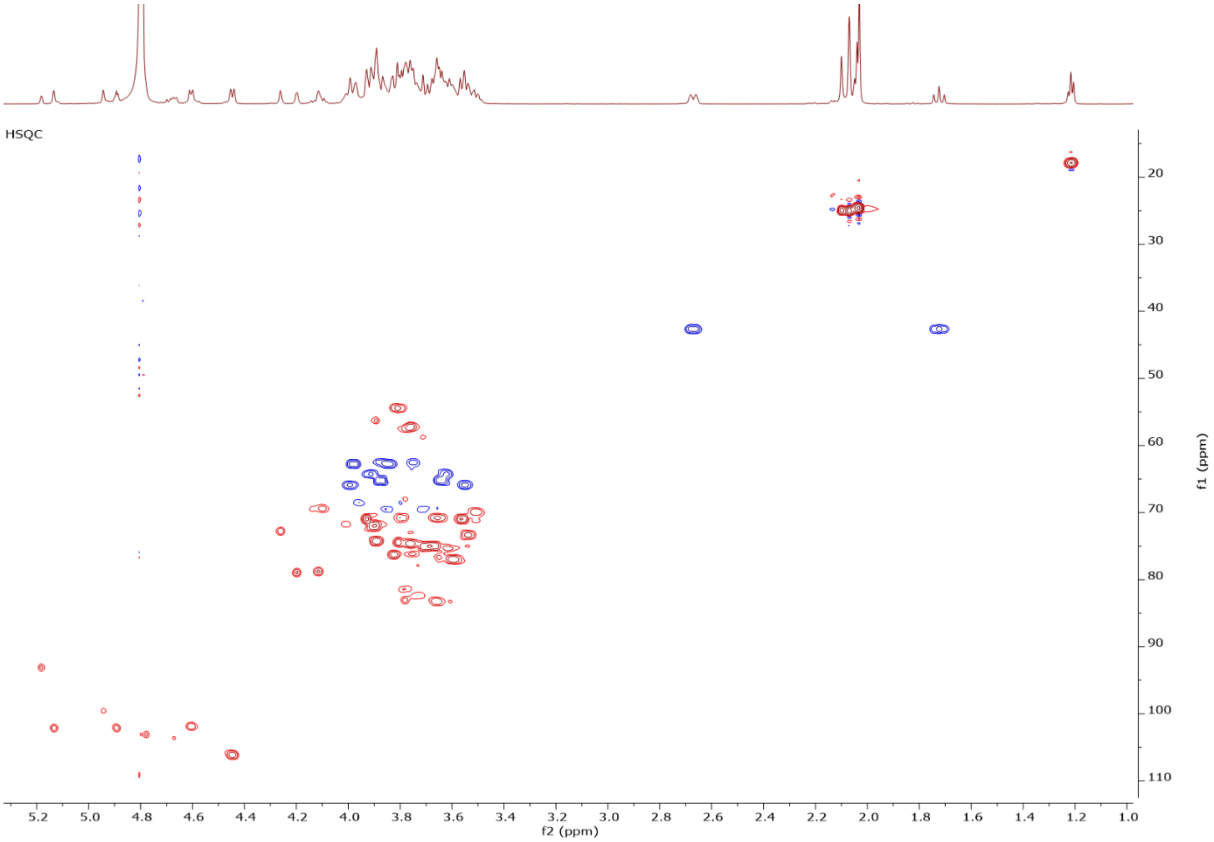
HSQC
f2 (ppm)
f1 (ppm)

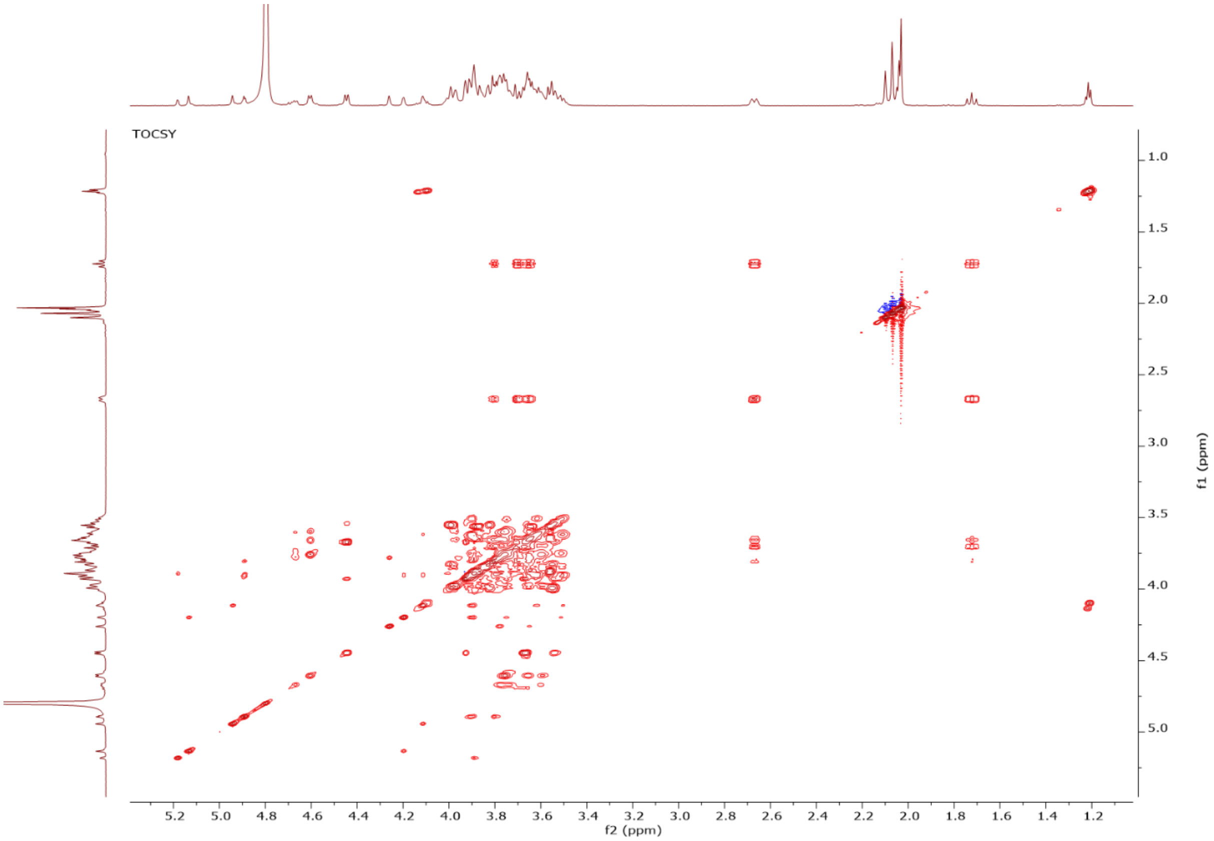
TOCSY
f2 (ppm)
f1 (ppm)

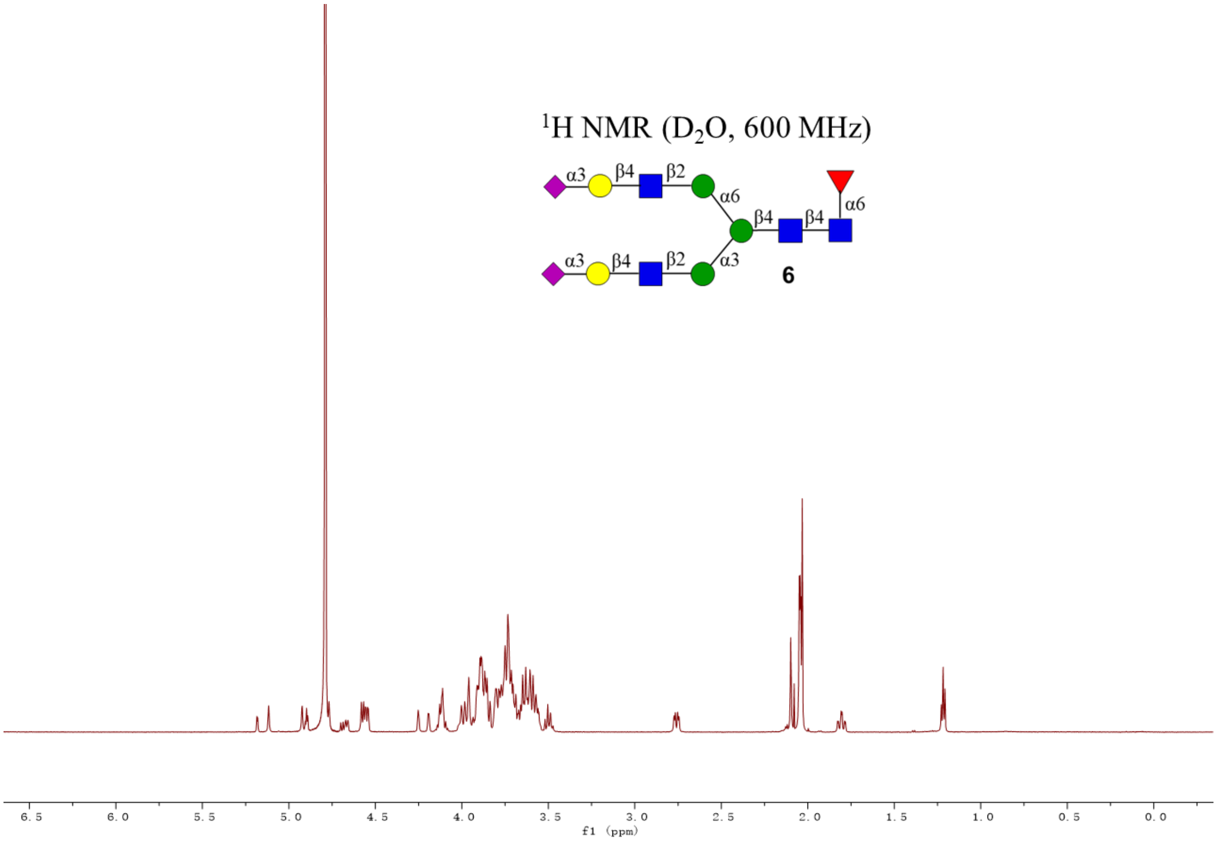

^{1}H NMR (D_2O, 600 MHz)
α3
β4
β2
α6
β4
β4
α6
α3
β4
β2
α3
6
f1 (ppm)

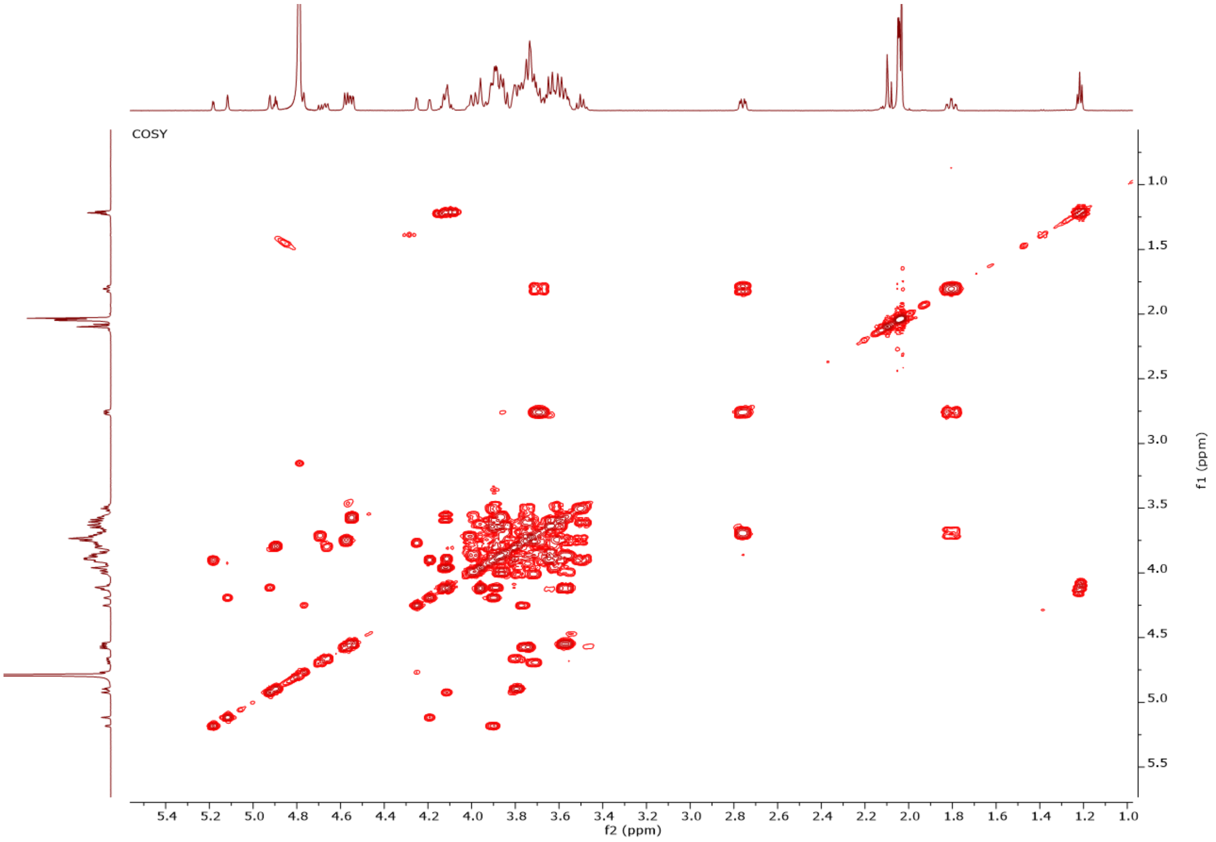

COSY
f2 (ppm)
f1 (ppm)

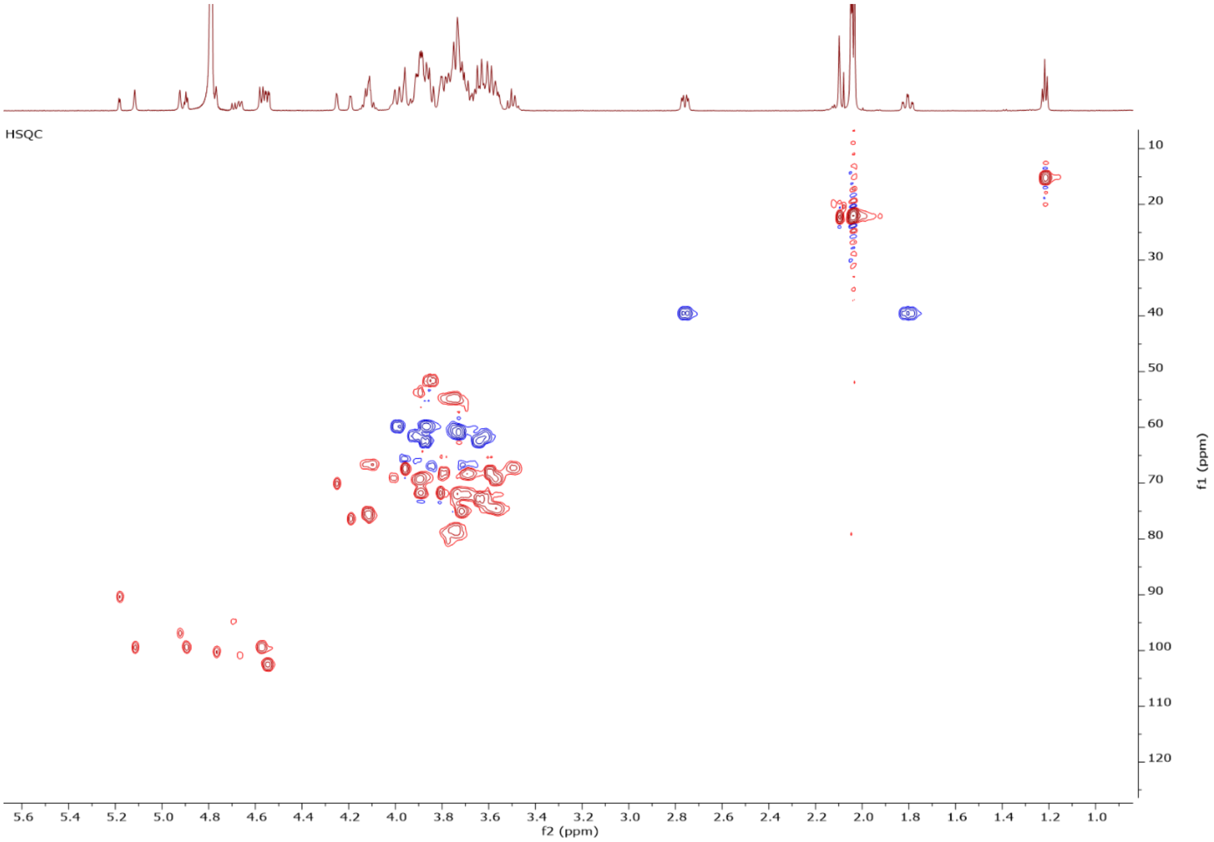
HSQC
f2 (ppm)
f1 (ppm)

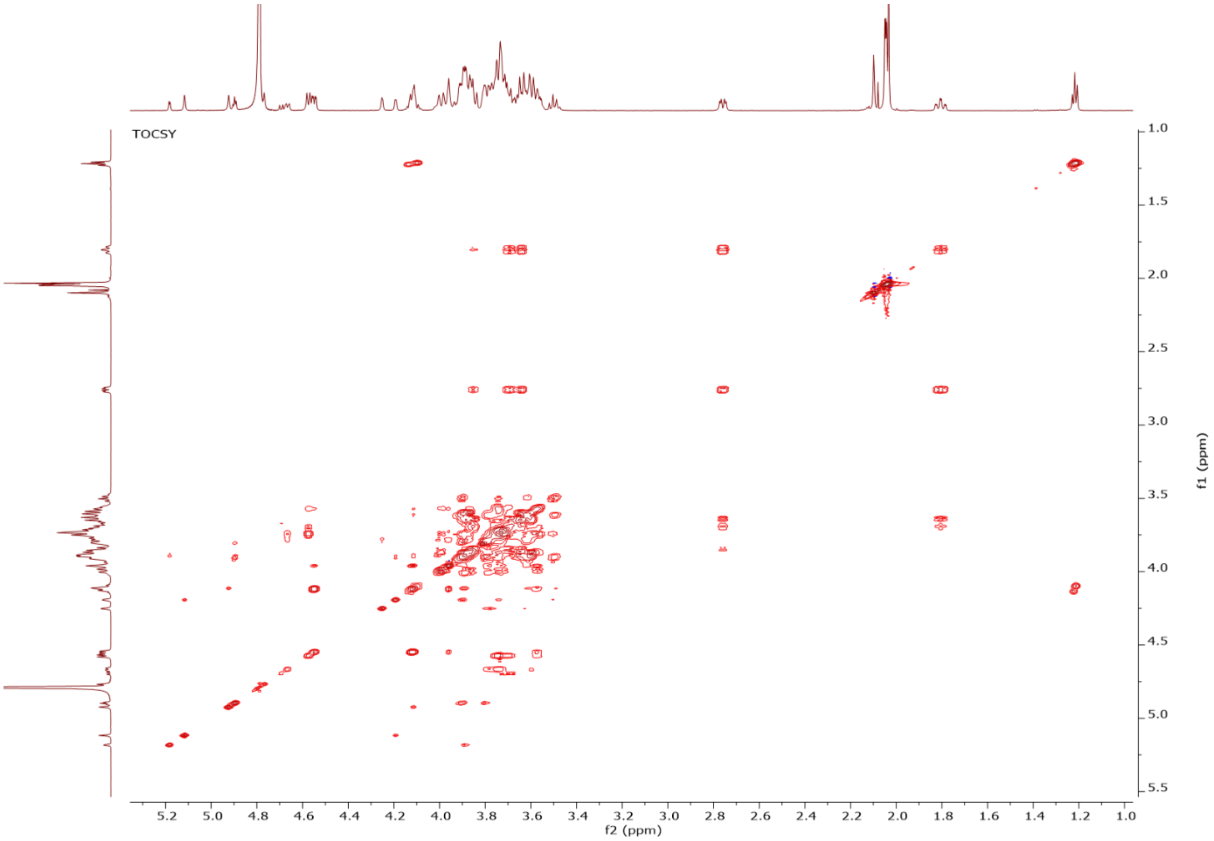
TOCSY
f2 (ppm)
f1 (ppm)

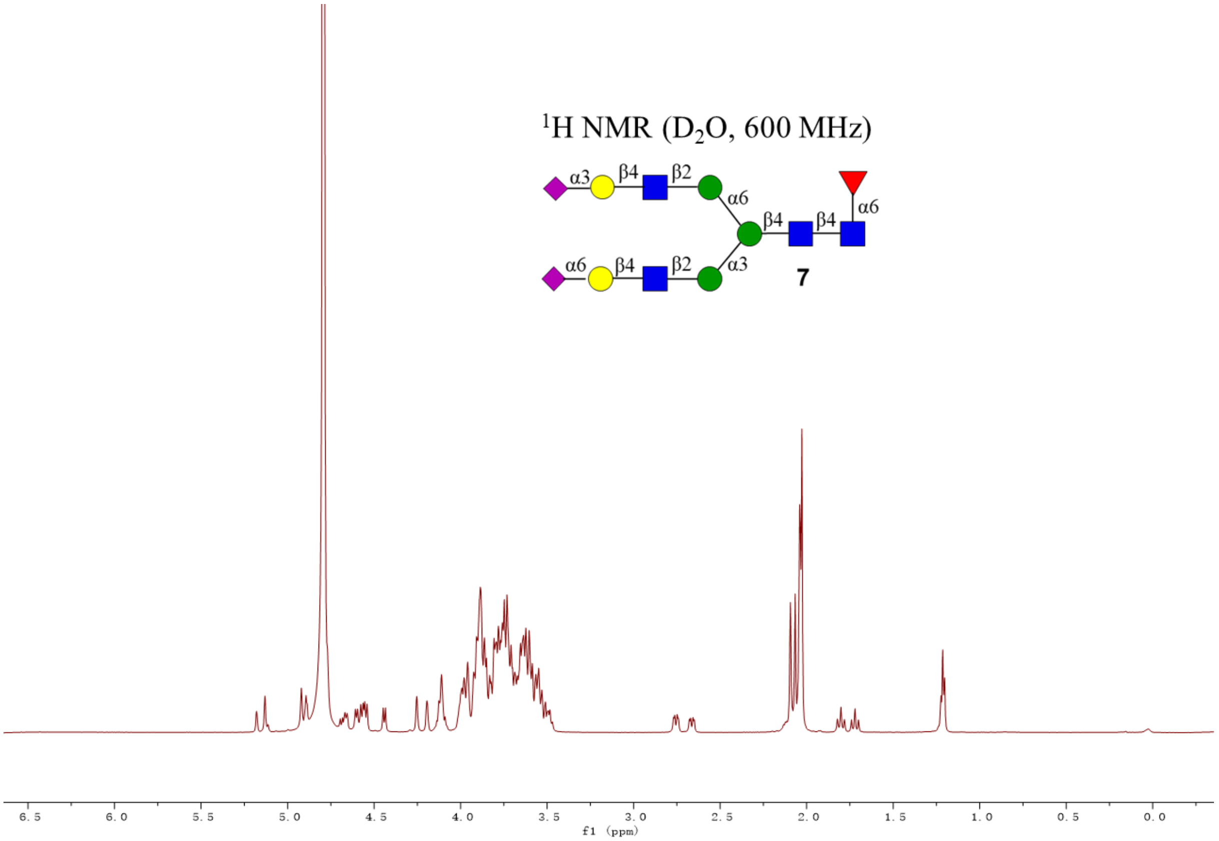

^{1}H NMR (D$_2$O, 600 MHz)
α3 β4 β2 α6 β4 β4 α6
α6 β4 β2 α3
7
f1 (ppm)

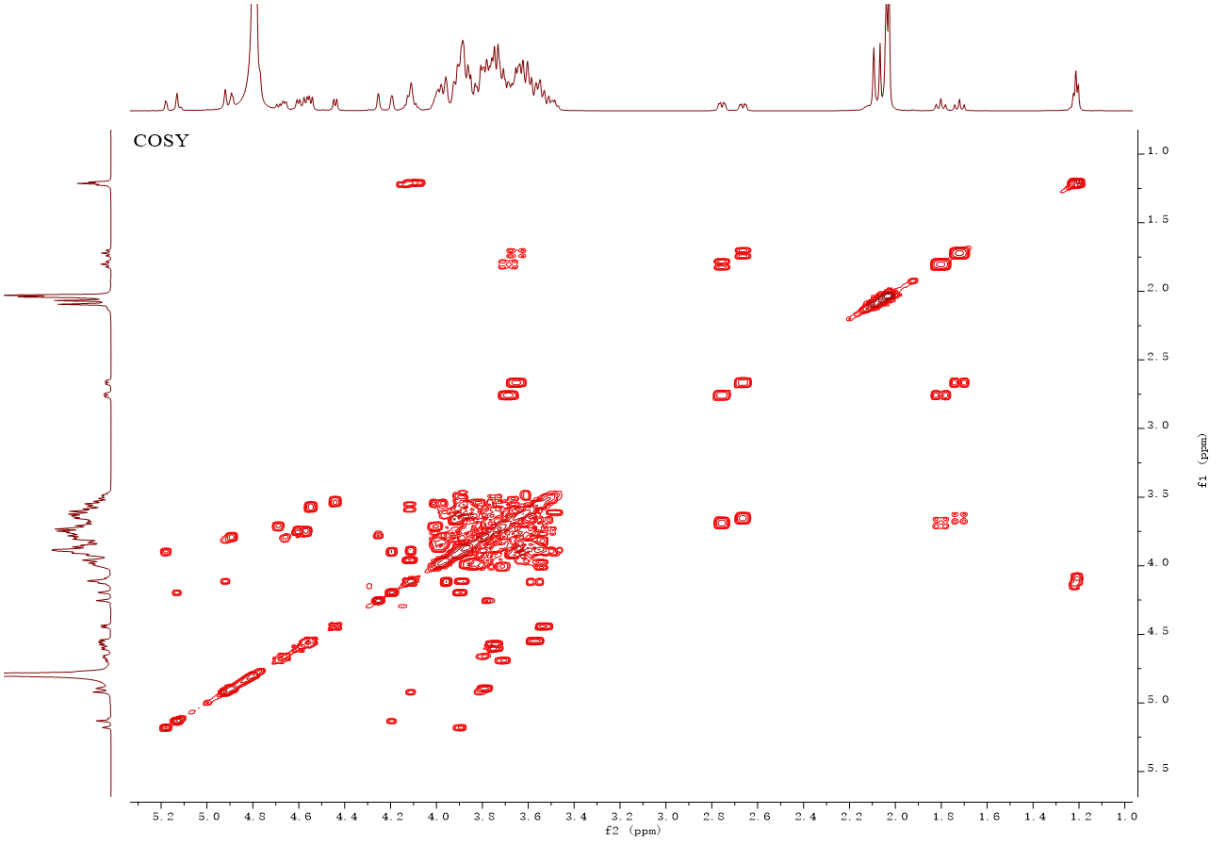

COSY
f2 (ppm)
f1 (ppm)

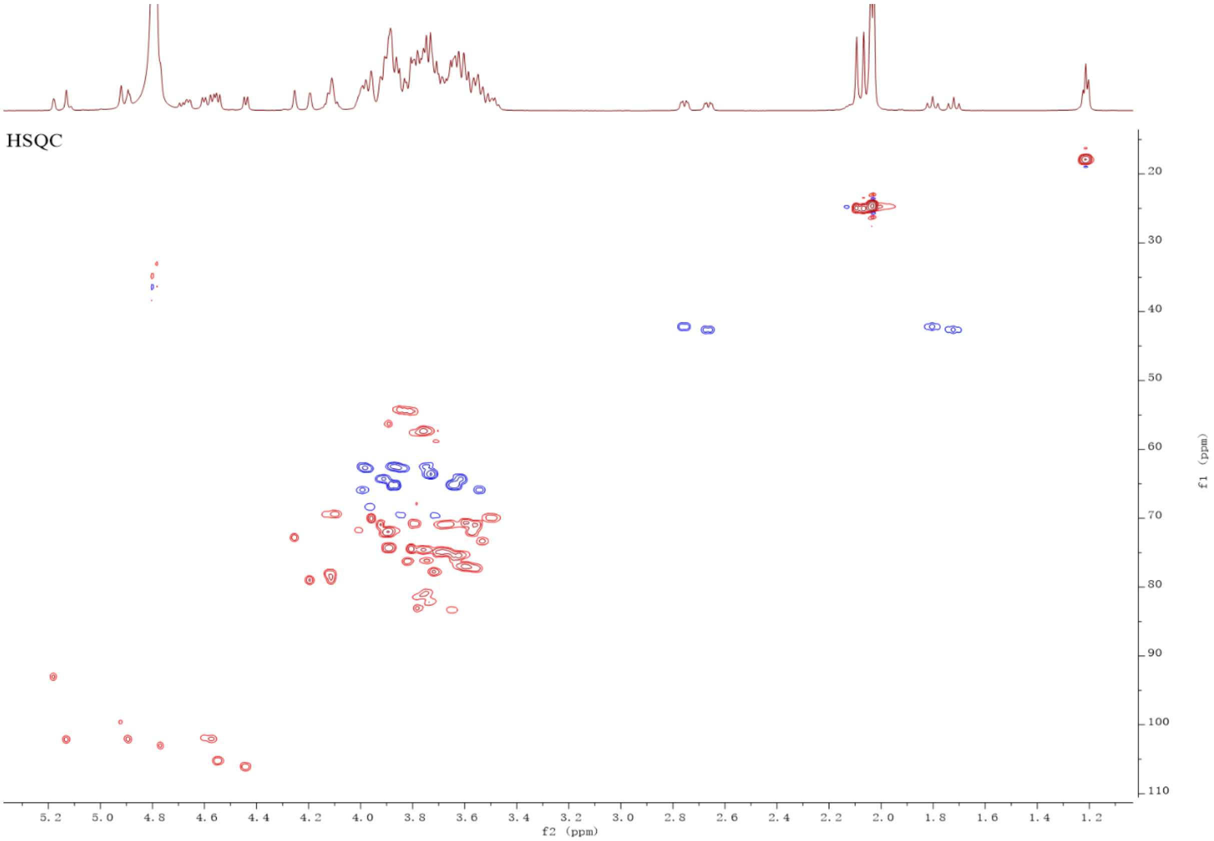
HSQC
f2 (ppm)
f1 (ppm)

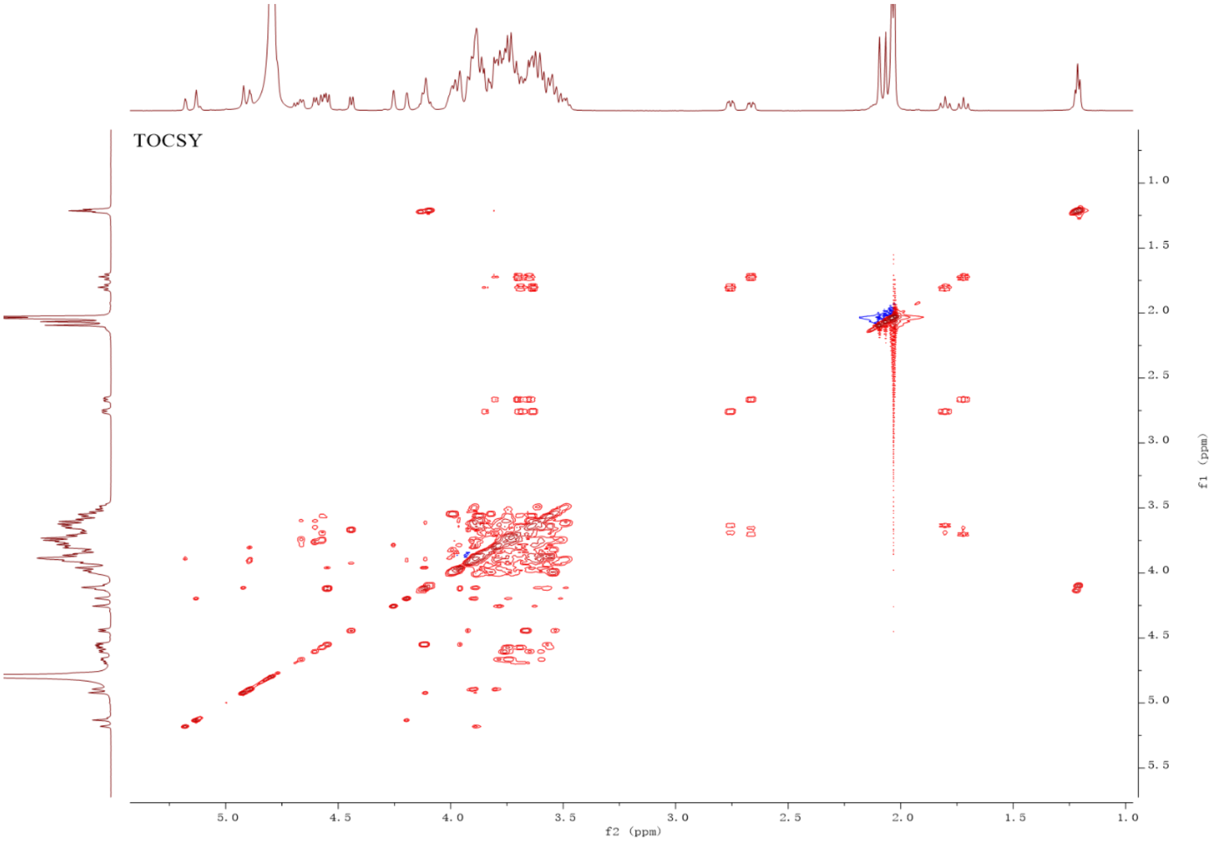
TOCSY
f2 (ppm)
f1 (ppm)

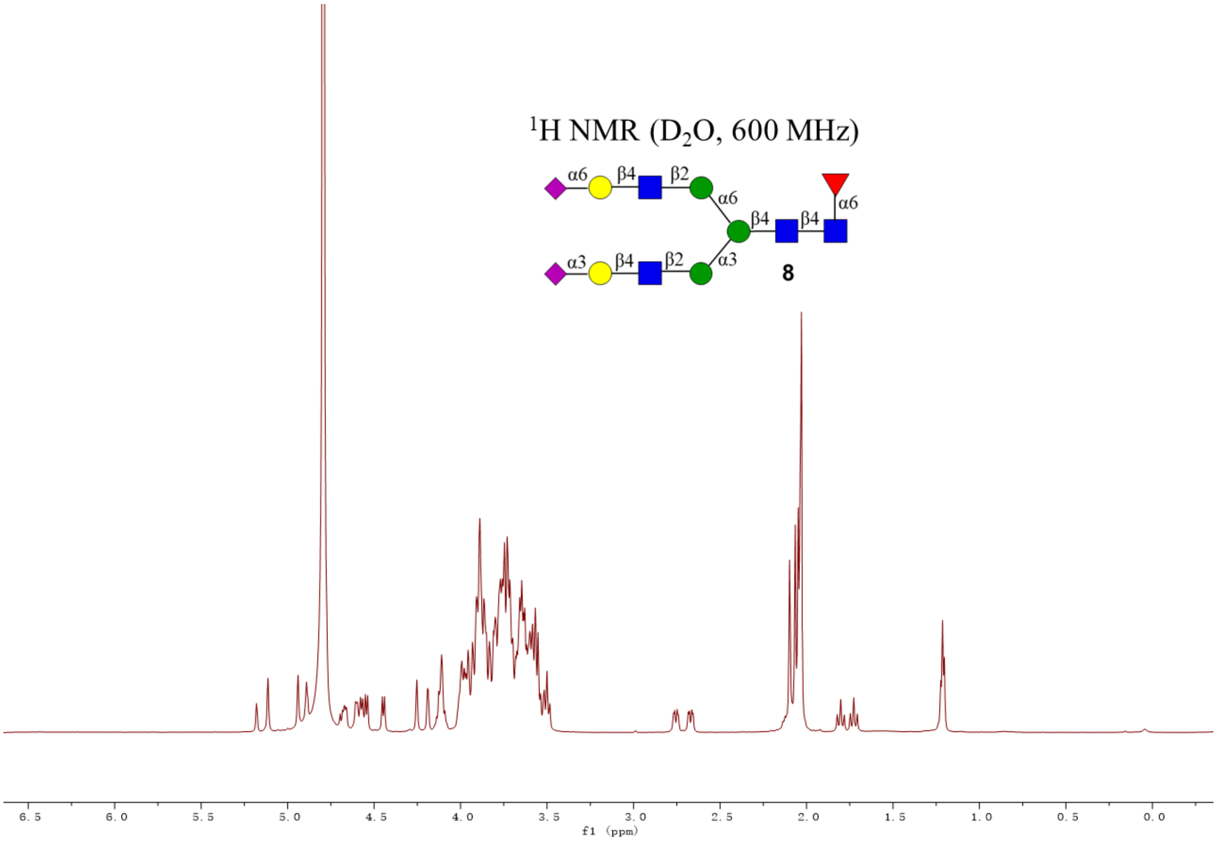

^{1}H NMR (D_2O, 600 MHz)
α6
β4
β2
α6
β4
β4
α6
α3
β4
β2
α3
8
f1 (ppm)

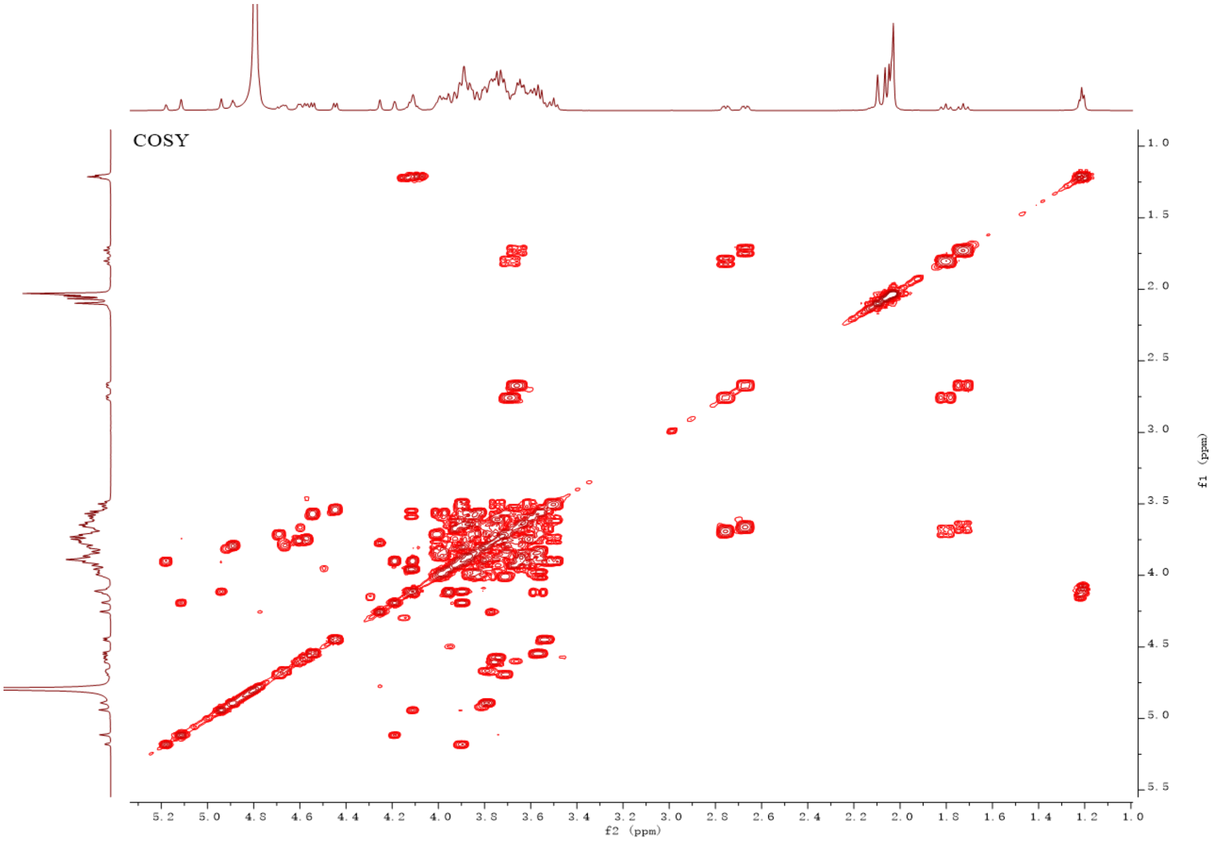

COSY
f2 (ppm)
f1 (ppm)

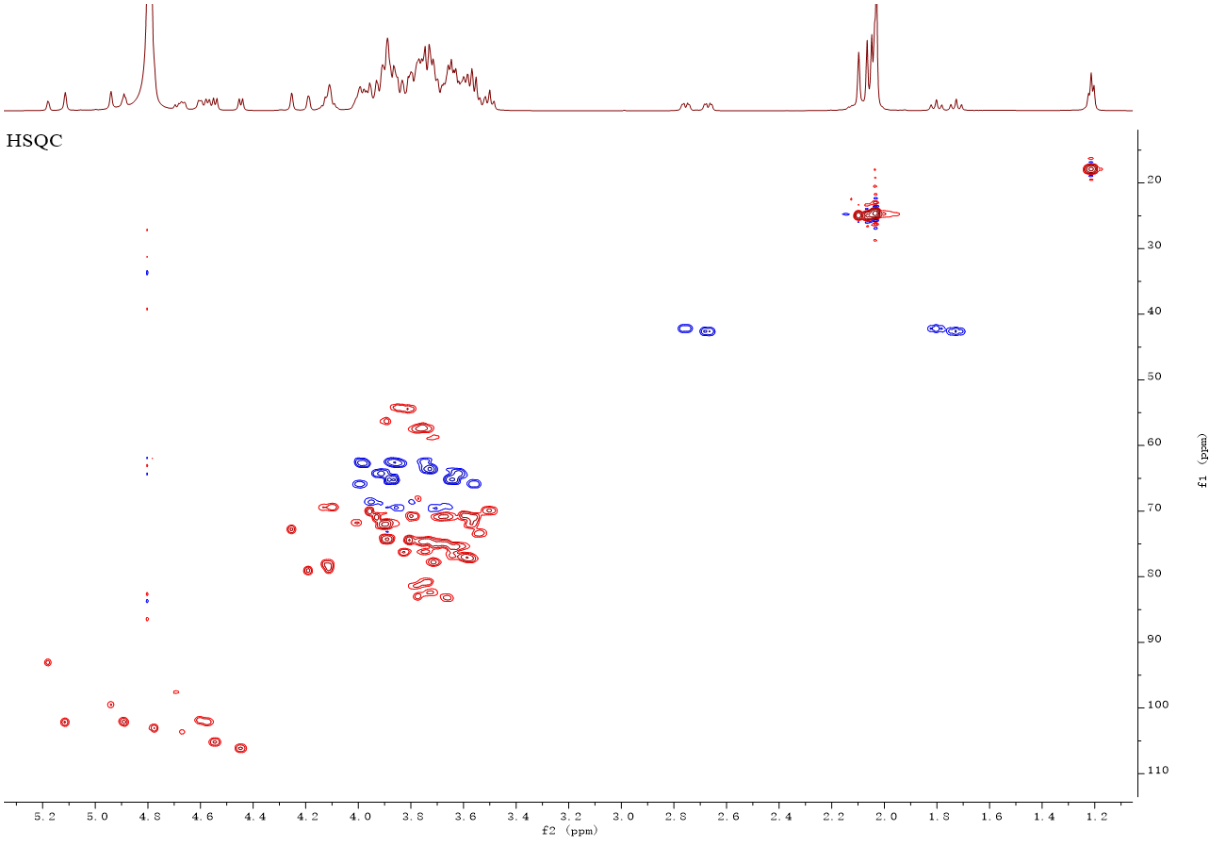
HSQC
f2 (ppm)
f1 (ppm)

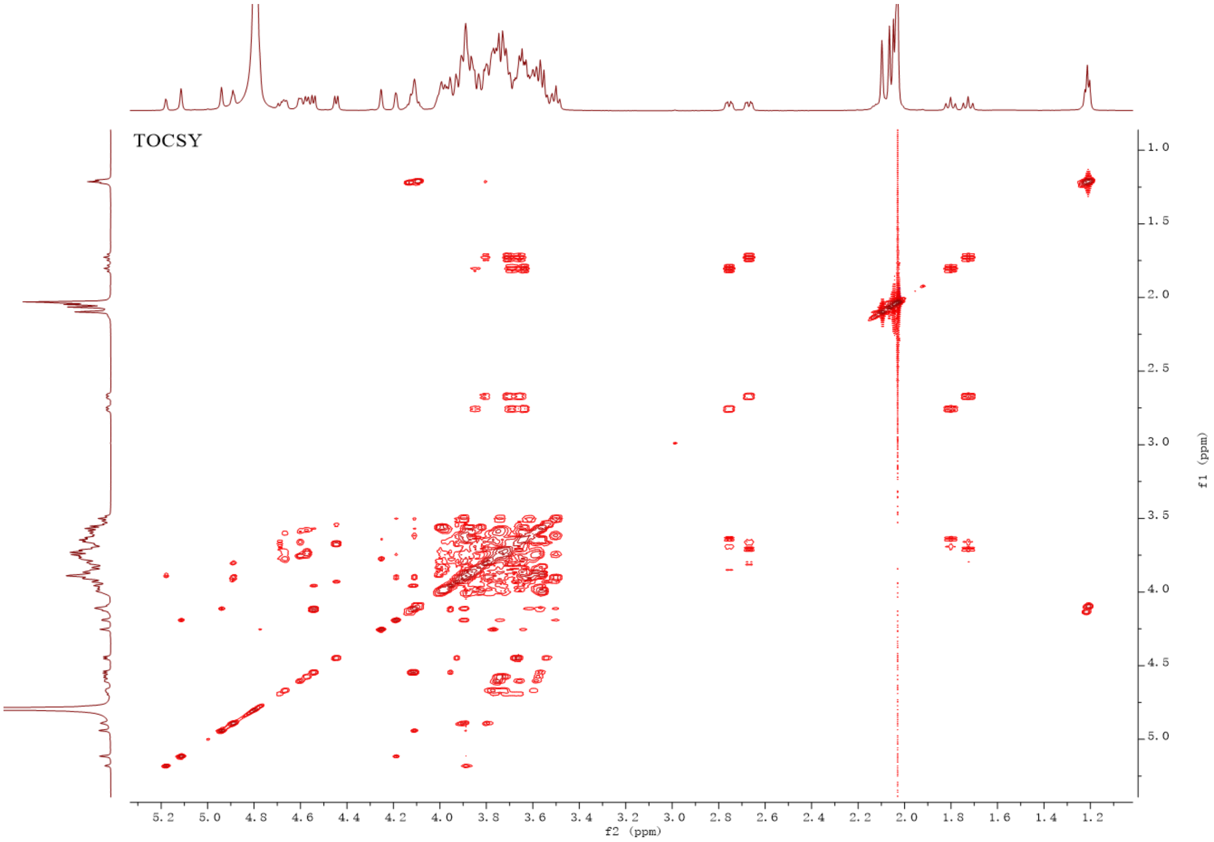
TOCSY
f2 (ppm)
f1 (ppm)

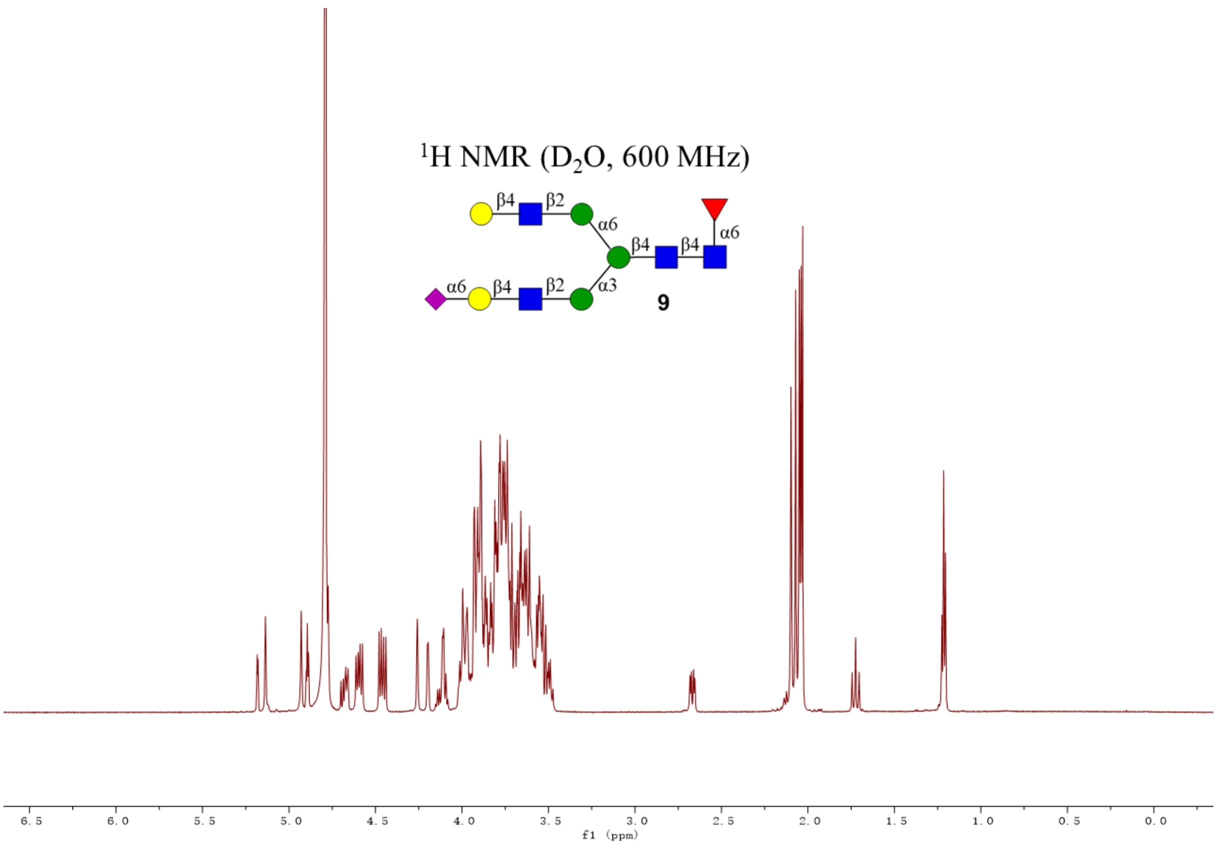

^{1}H NMR (D_2O, 600 MHz)
9
f1 (ppm)

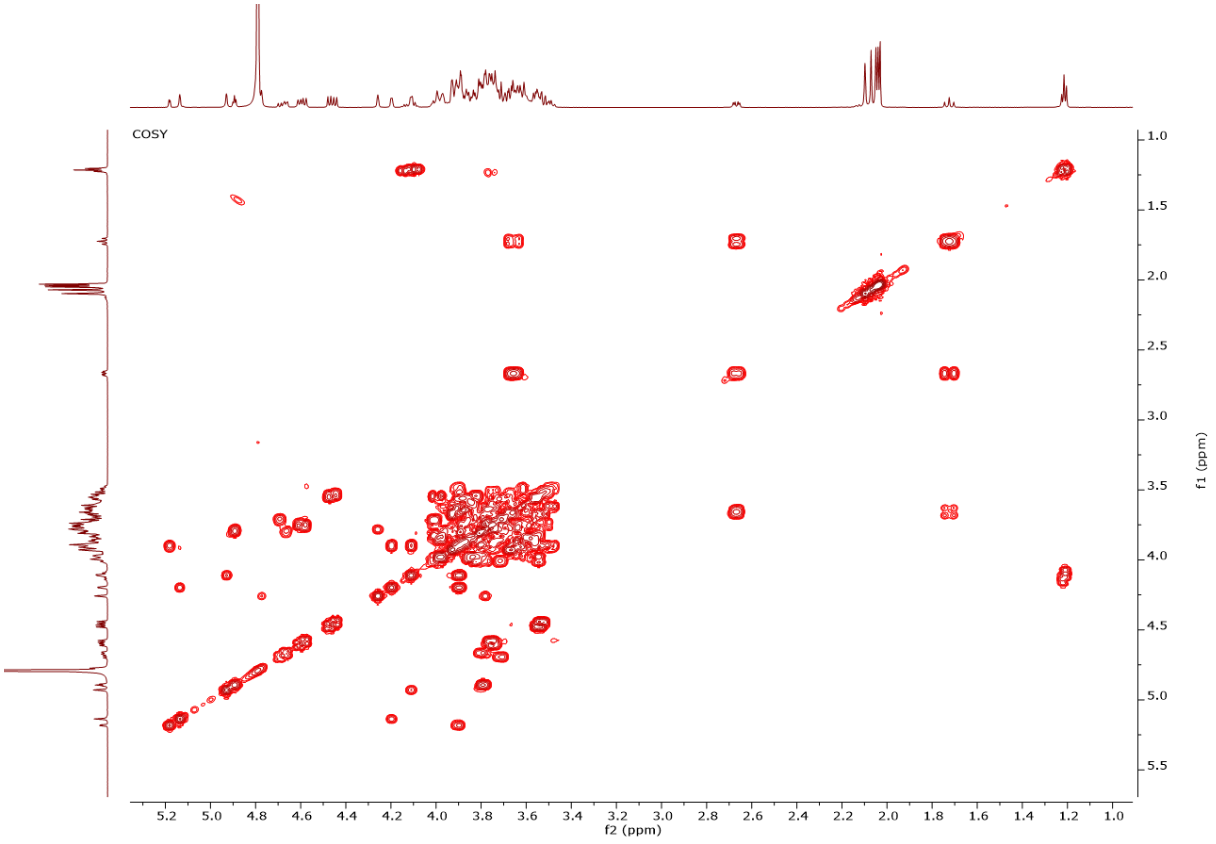

COSY
f2 (ppm)
f1 (ppm)

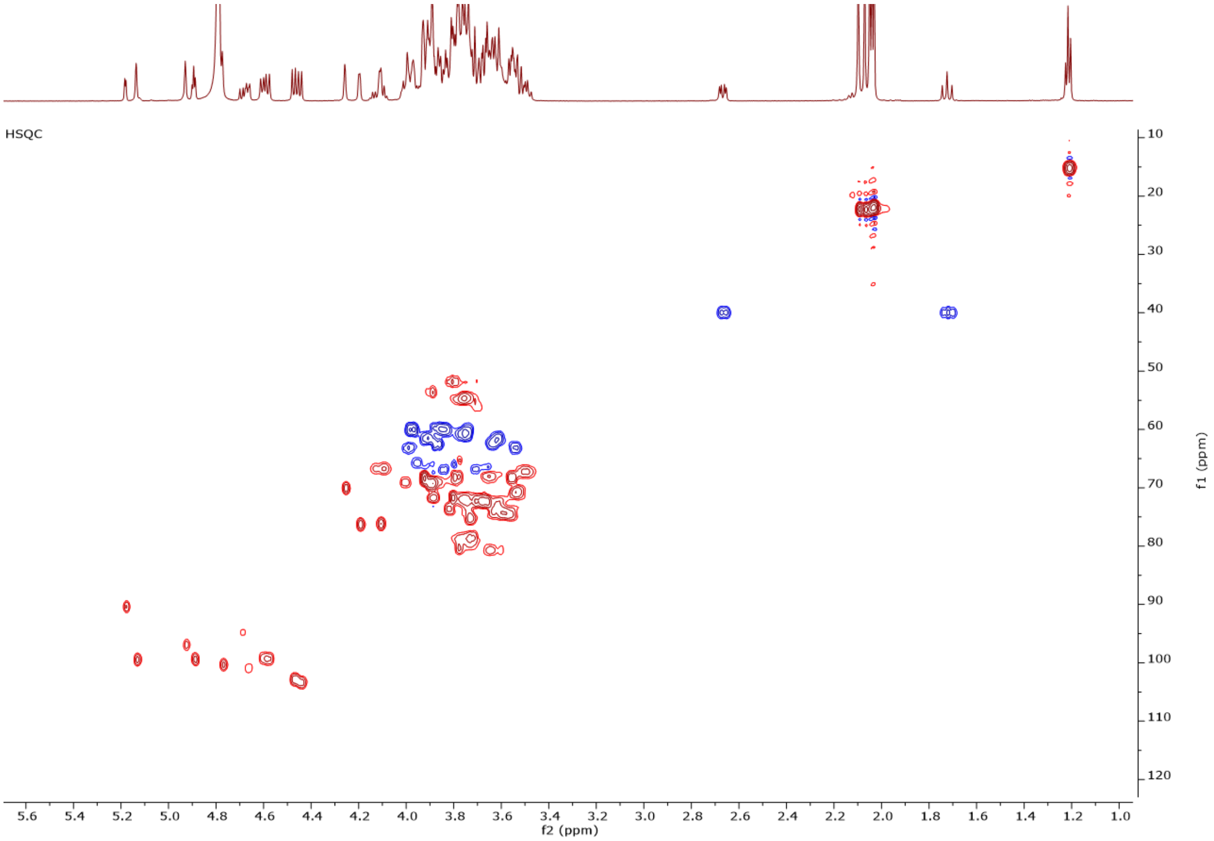
HSQC
f2 (ppm)
f1 (ppm)

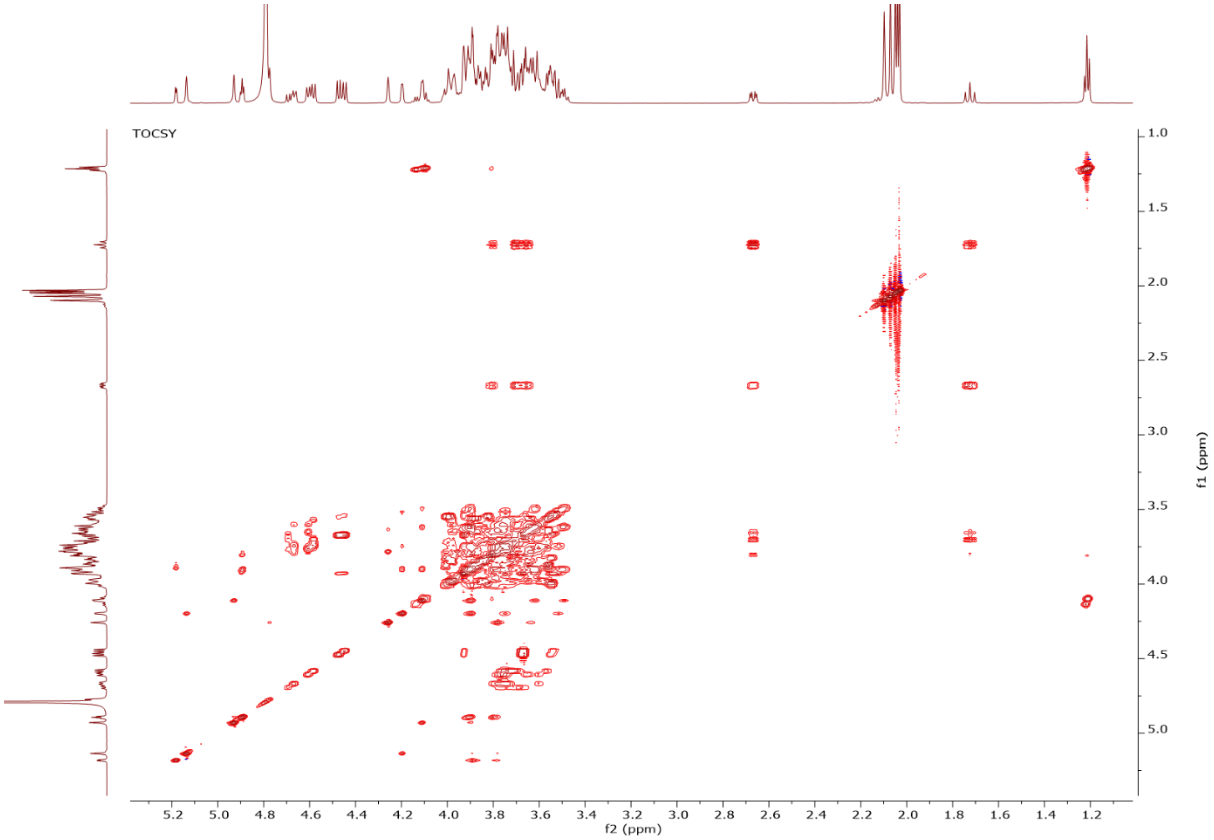
TOCSY
f2 (ppm)
f1 (ppm)

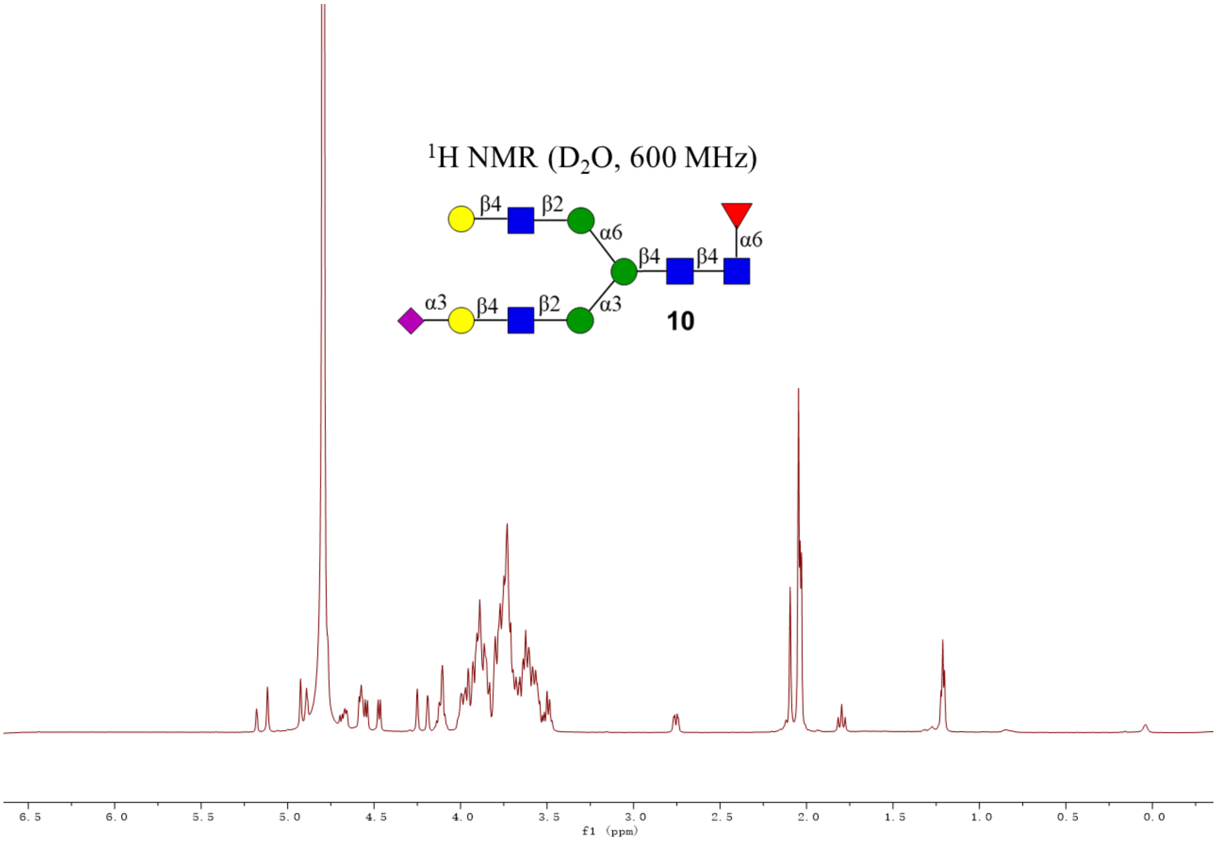
1H NMR (D2O, 600 MHz)
β4
β2
α6
β4
β4
α6
α3
β4
β2
α3
10
f1 (ppm)

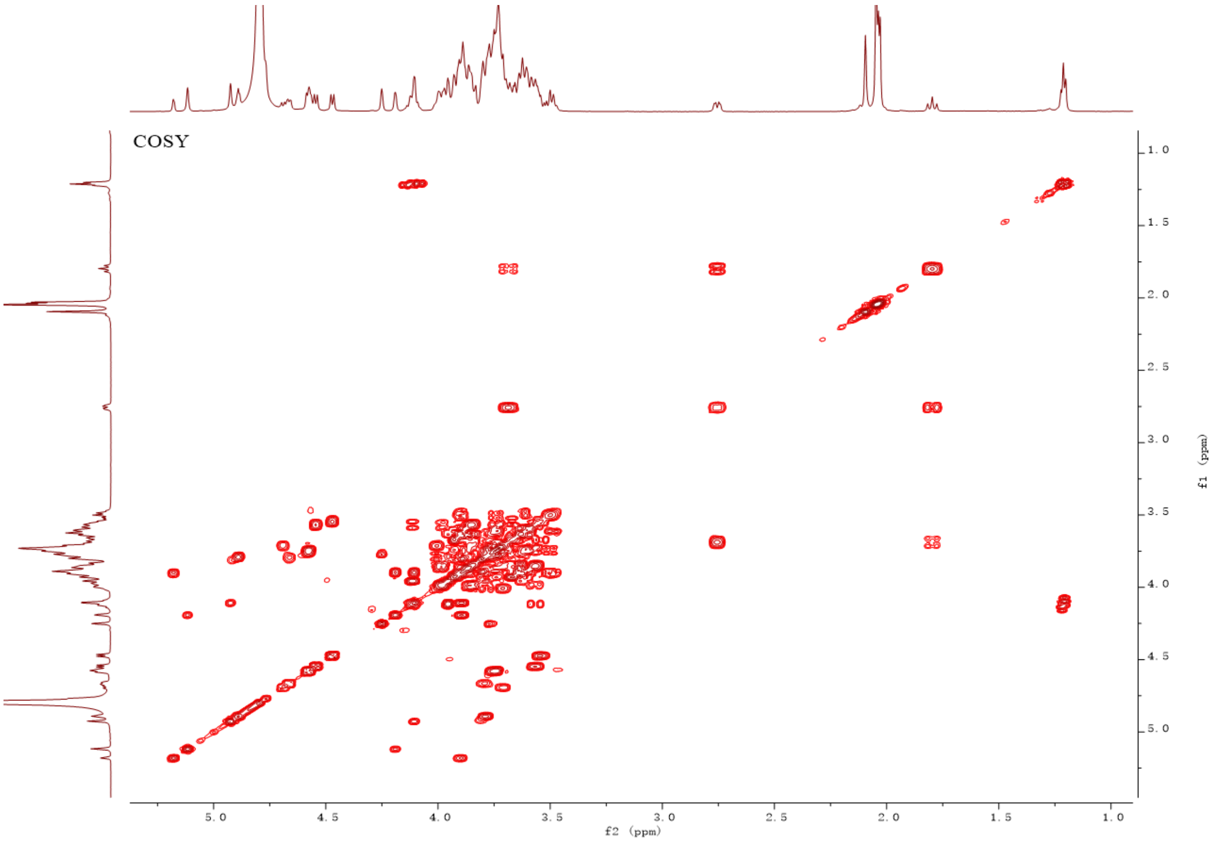
COSY
f2 (ppm)
f1 (ppm)

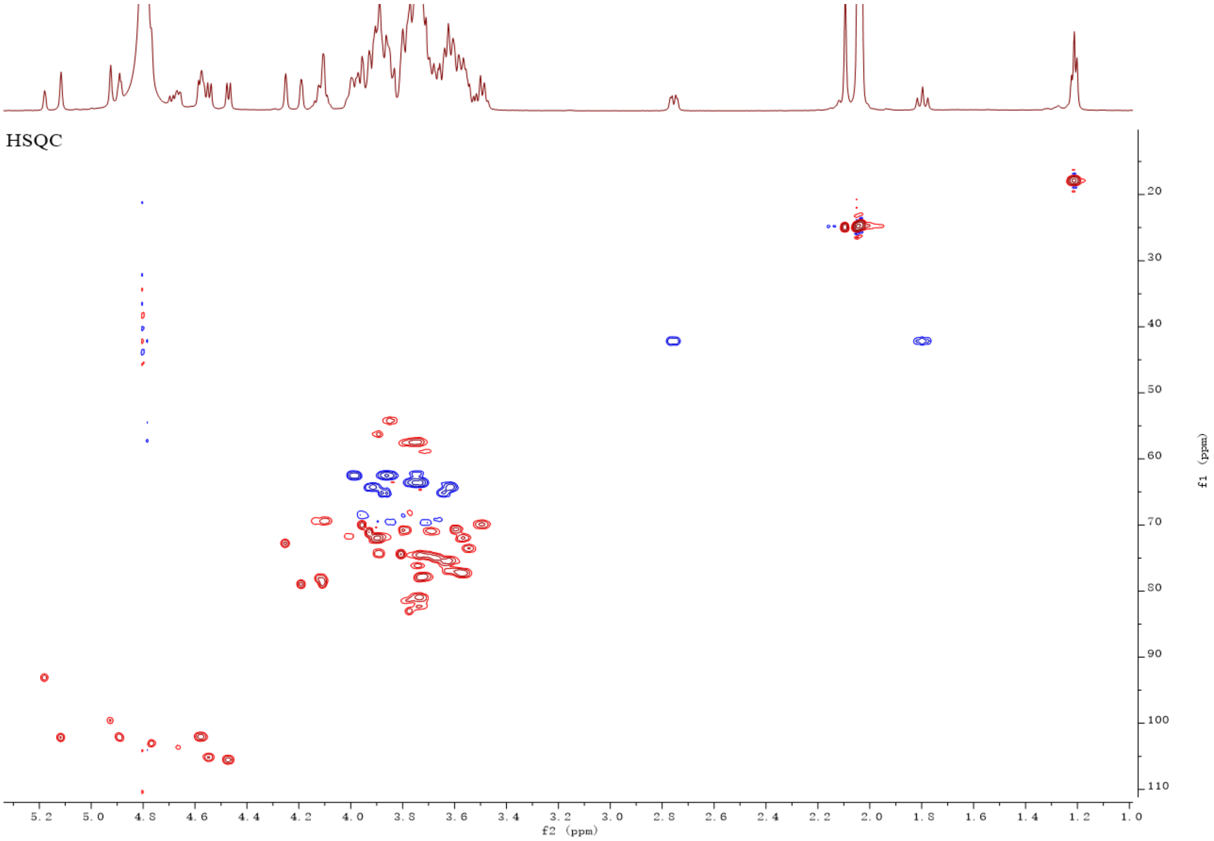
HSQC
f2 (ppm)
f1 (ppm)

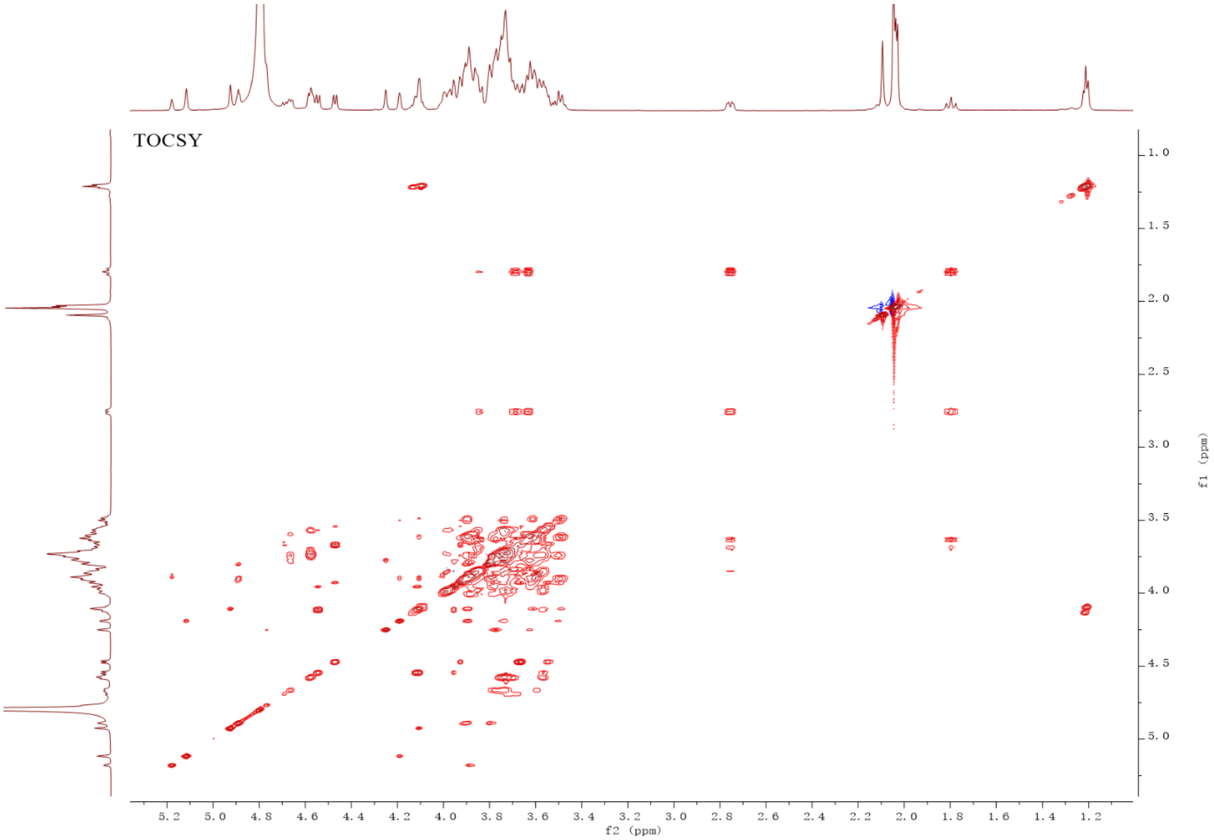
TOCSY
f2 (ppm)
f1 (ppm)

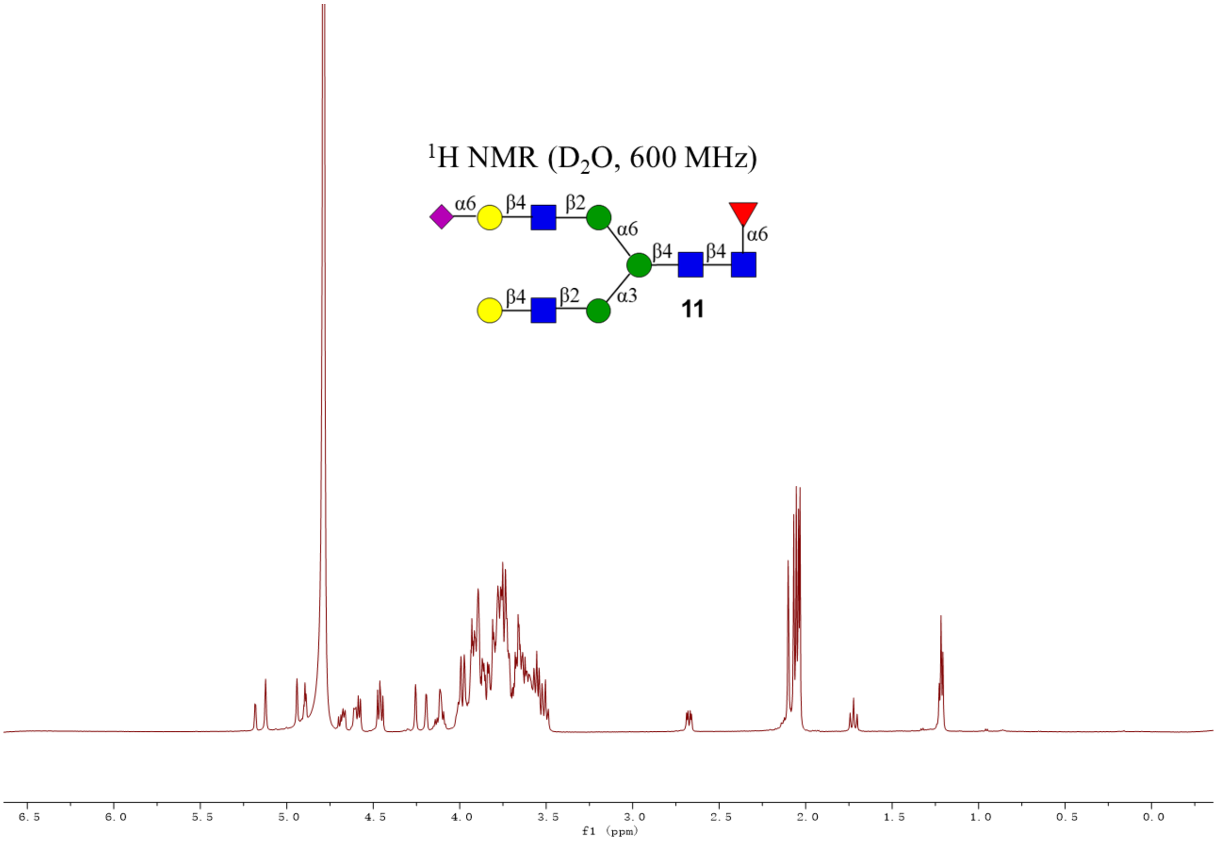

1H NMR (D2O, 600 MHz)
α6
β4
β2
α6
β4
β4
α6
β4
β2
α3
11
f1 (ppm)

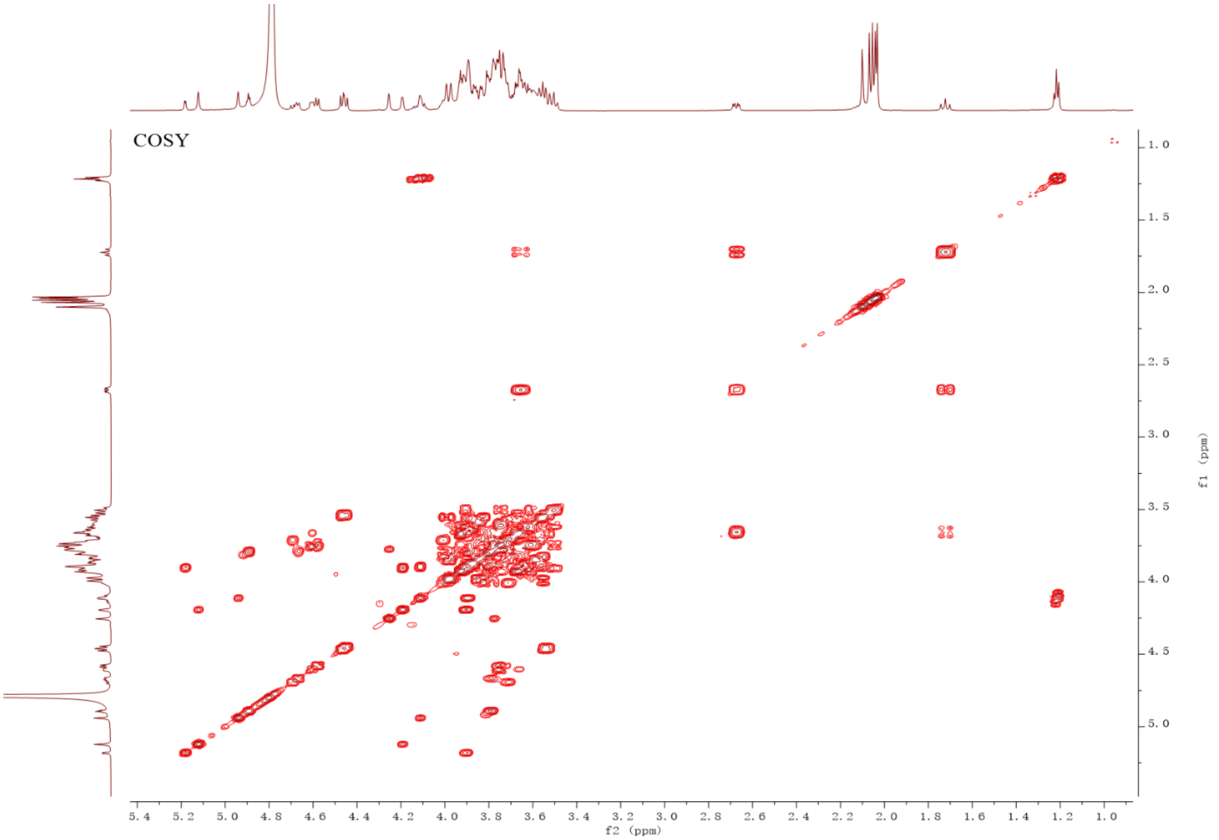

COSY
f2 (ppm)
f1 (ppm)

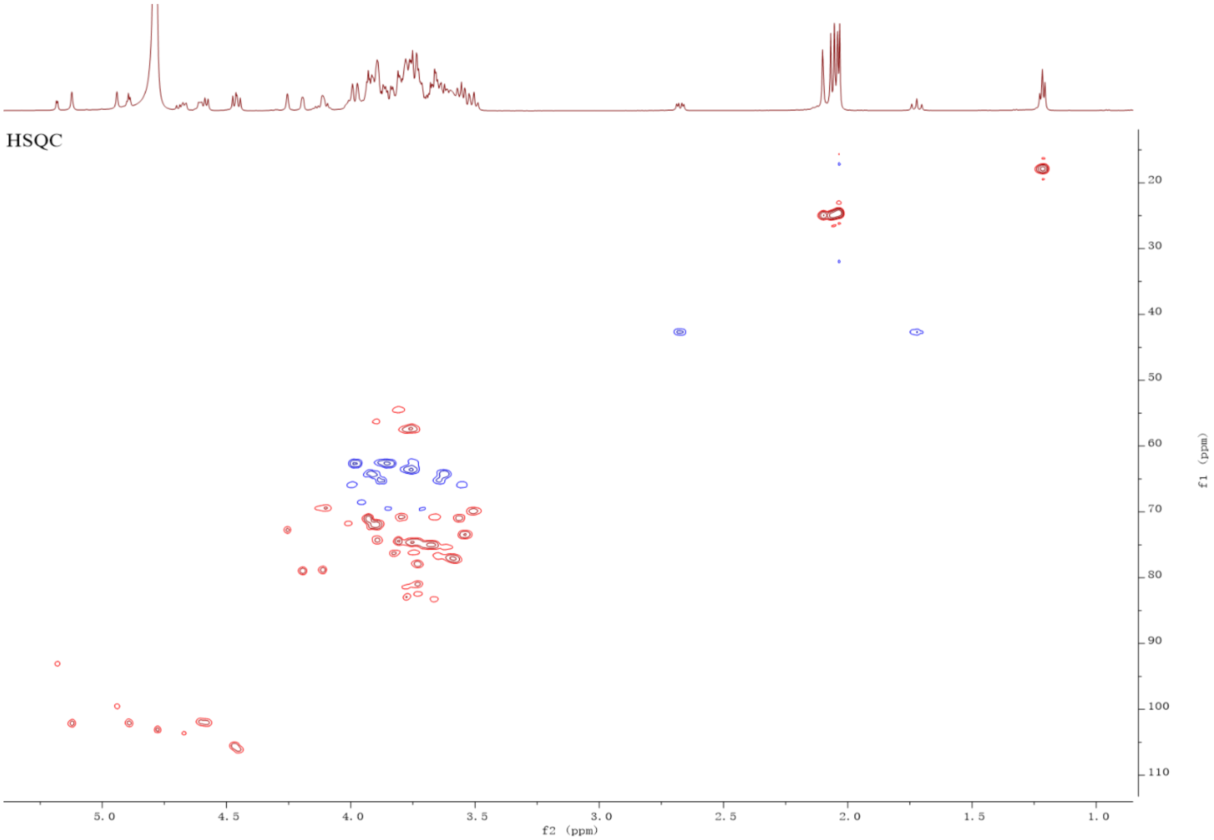
HSQC
f2 (ppm)
f1 (ppm)

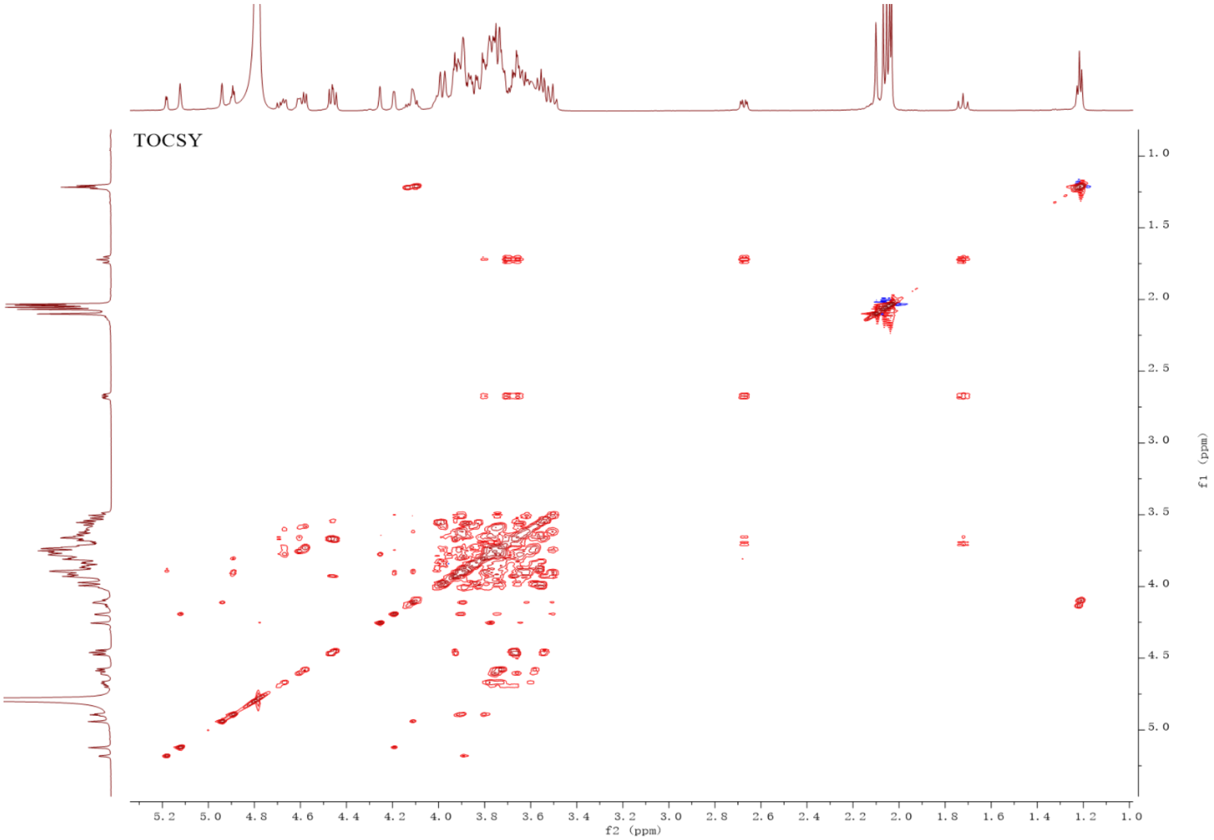
TOCSY
f2 (ppm)
f1 (ppm)

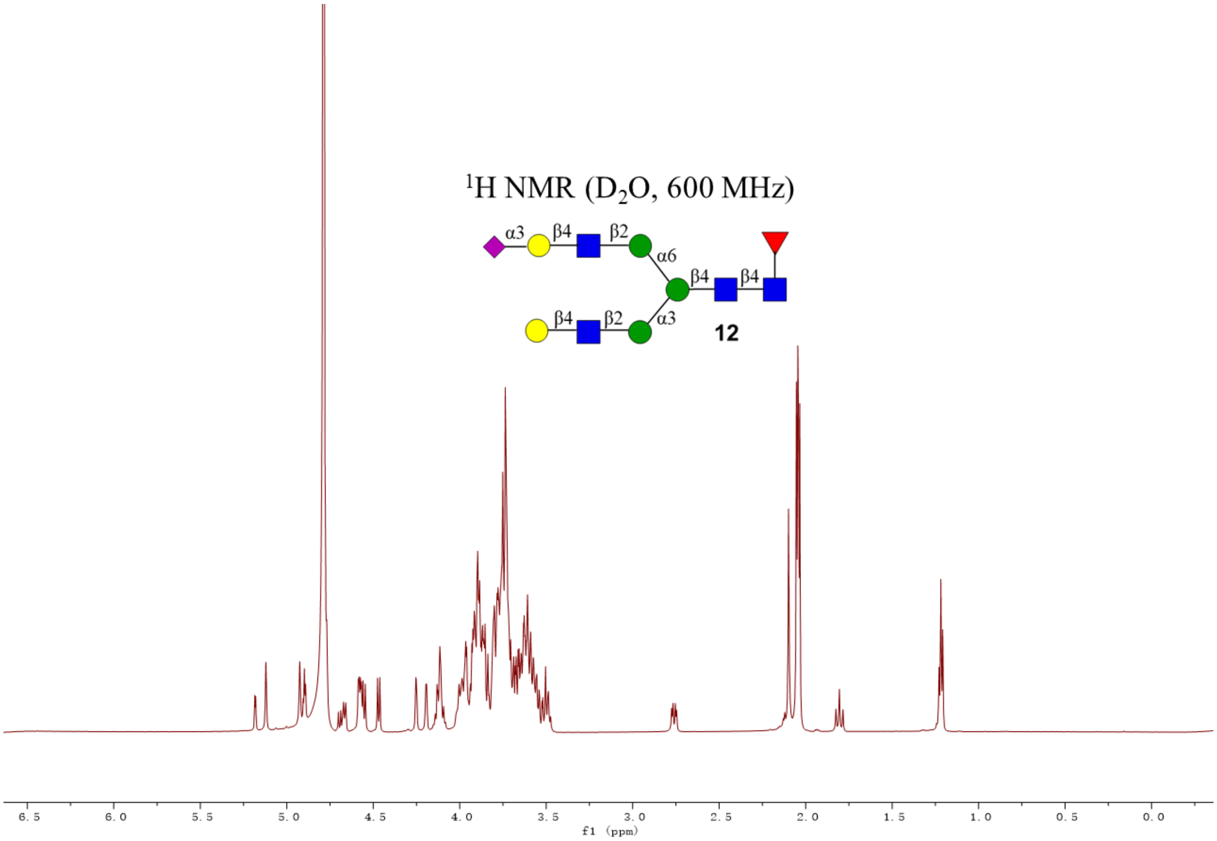

^{1}H NMR (D_2O, 600 MHz)
α3
β4
β2
α6
β4
β4
β4
β2
α3
12
6.5
6.0
5.5
5.0
4.5
4.0
3.5
3.0
2.5
2.0
1.5
1.0
0.5
0.0
f1 (ppm)

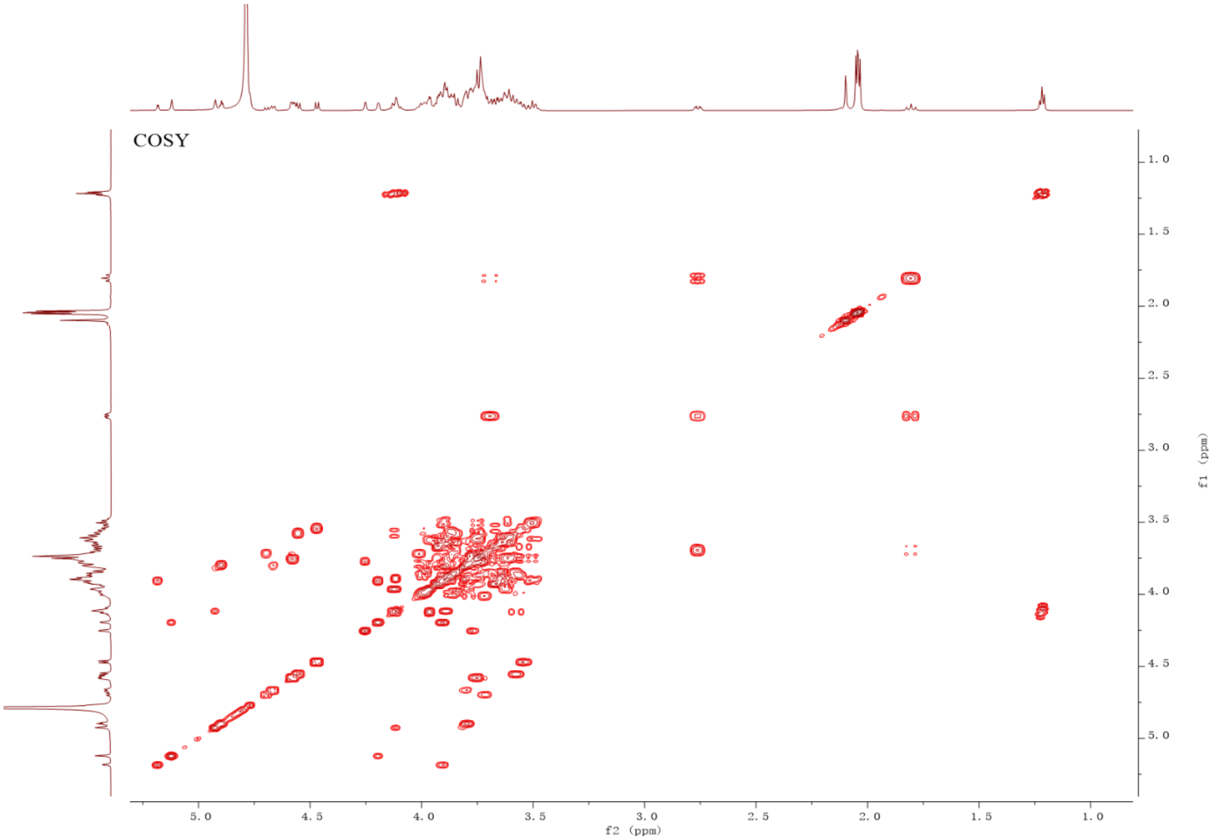

COSY
5.0
4.5
4.0
3.5
3.0
2.5
2.0
1.5
1.0
f2 (ppm)
f1 (ppm)

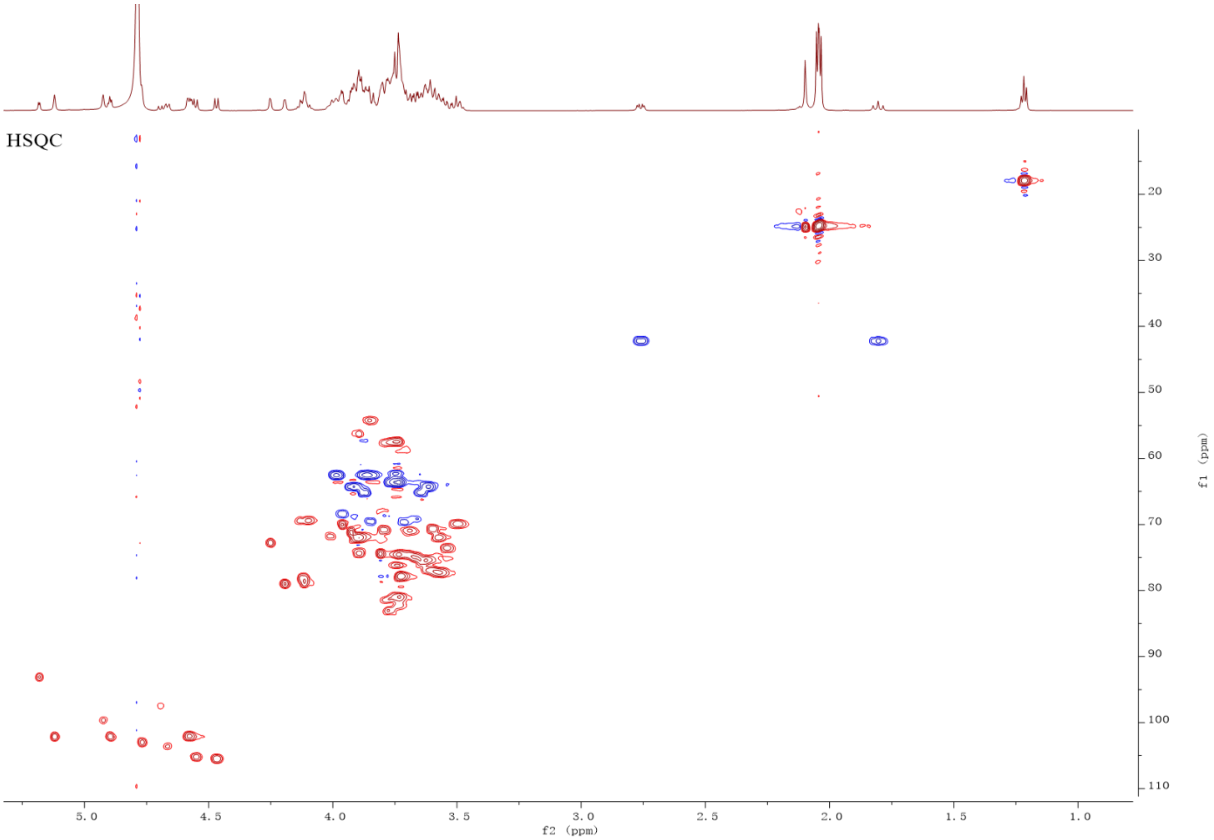
HSQC
f2 (ppm)
f1 (ppm)

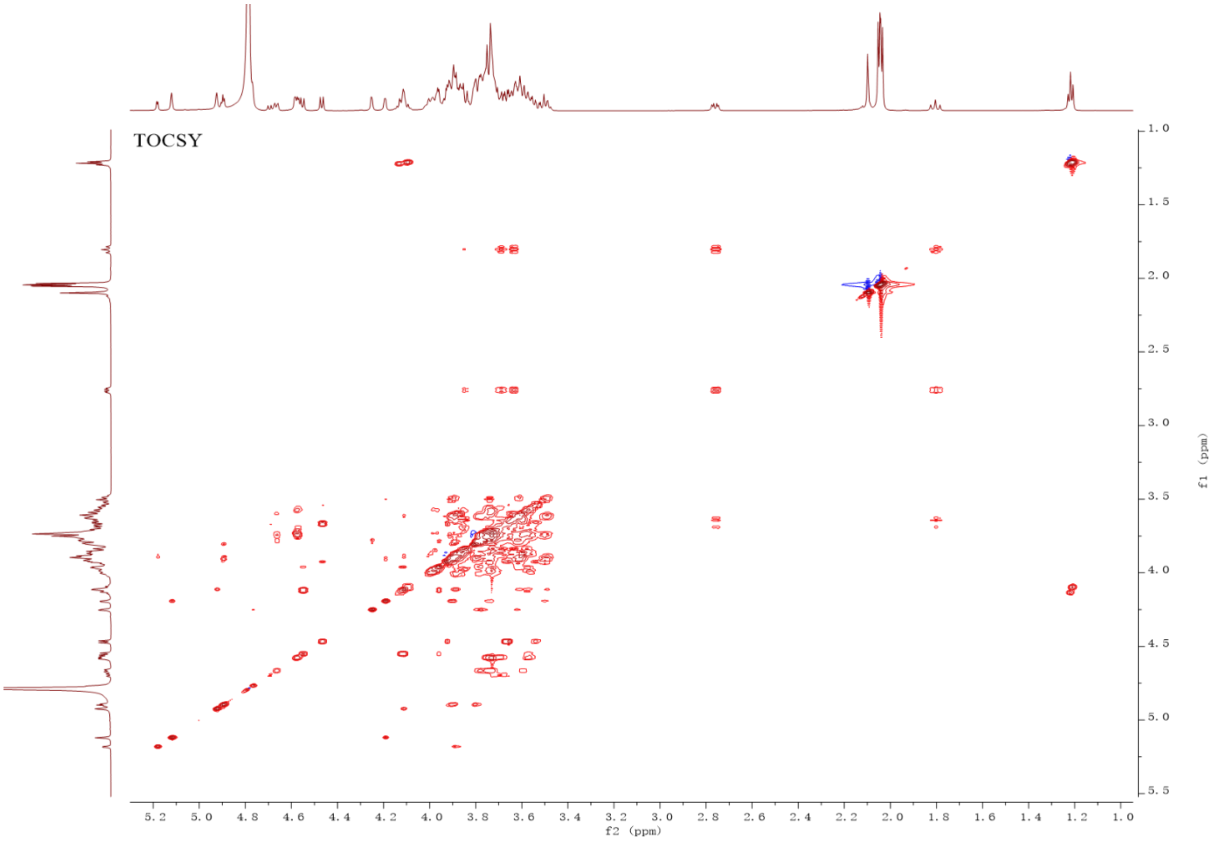
TOCSY
f2 (ppm)
f1 (ppm)

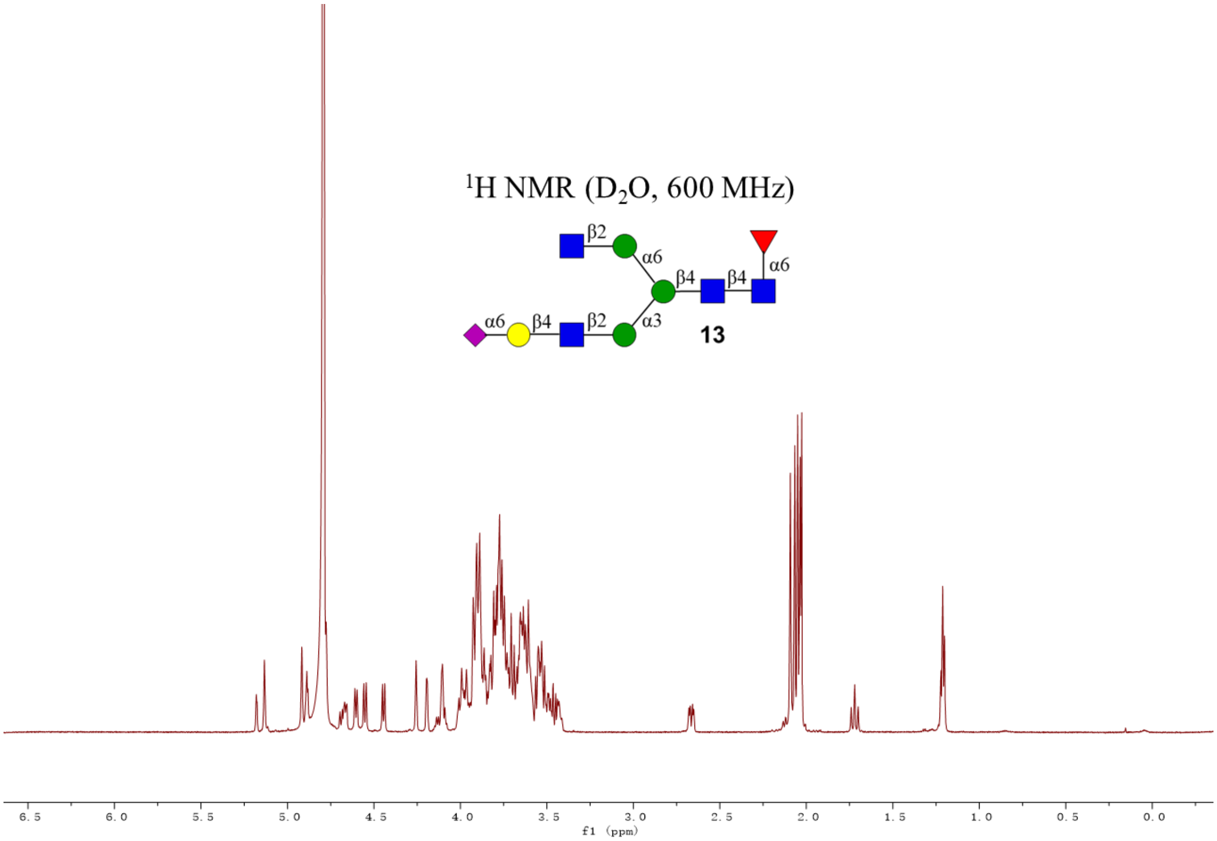
^{1}H NMR (D_2O, 600 MHz)
β2
α6
β4
β4
α6
α6
β4
β2
α3
13
f1 (ppm)

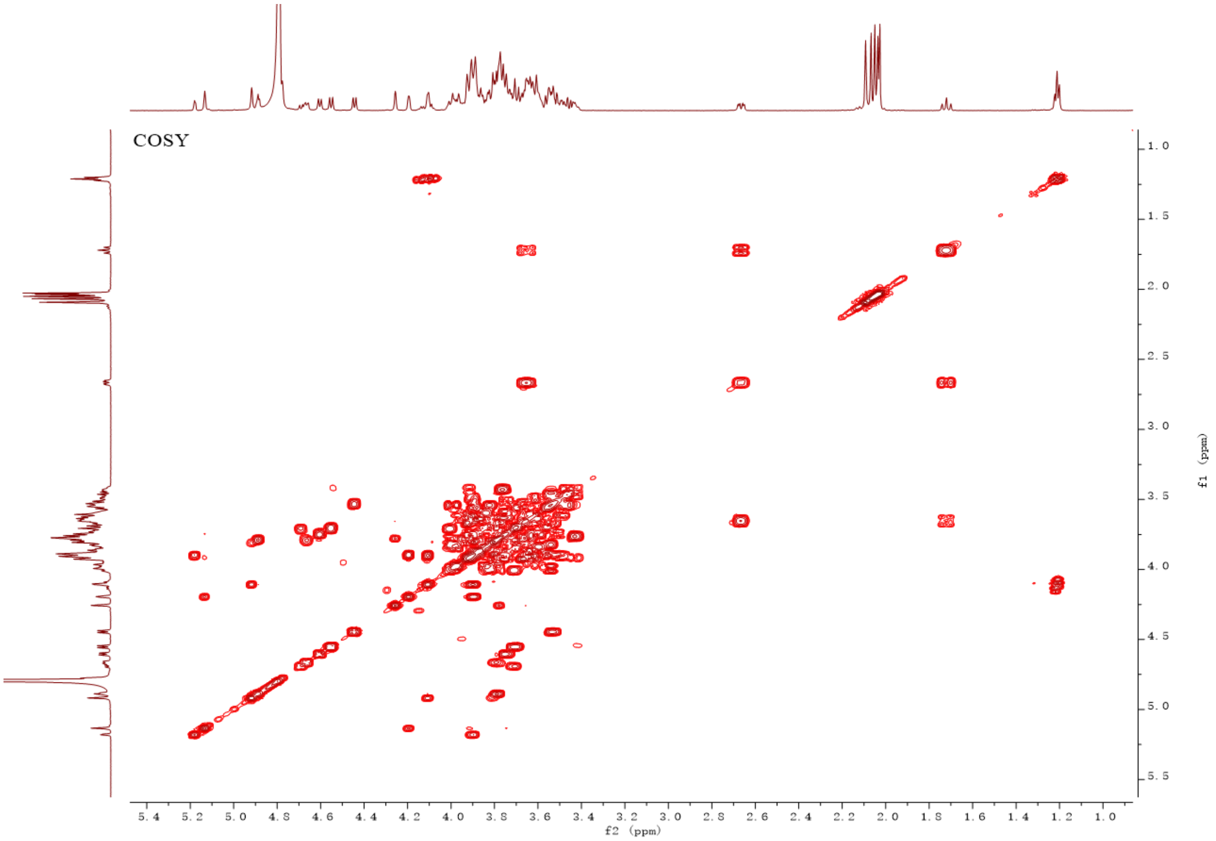
COSY
f2 (ppm)
f1 (ppm)

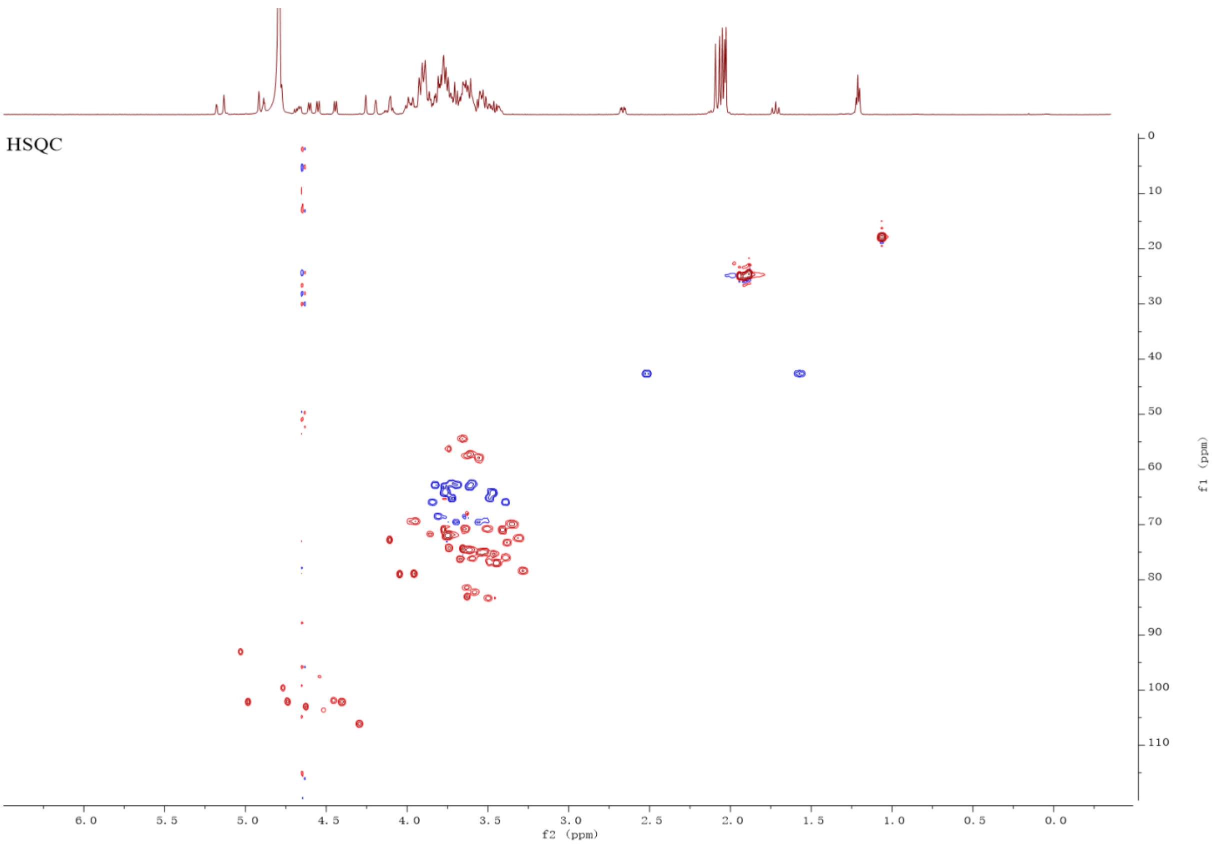
HSQC
f2 (ppm)
f1 (ppm)

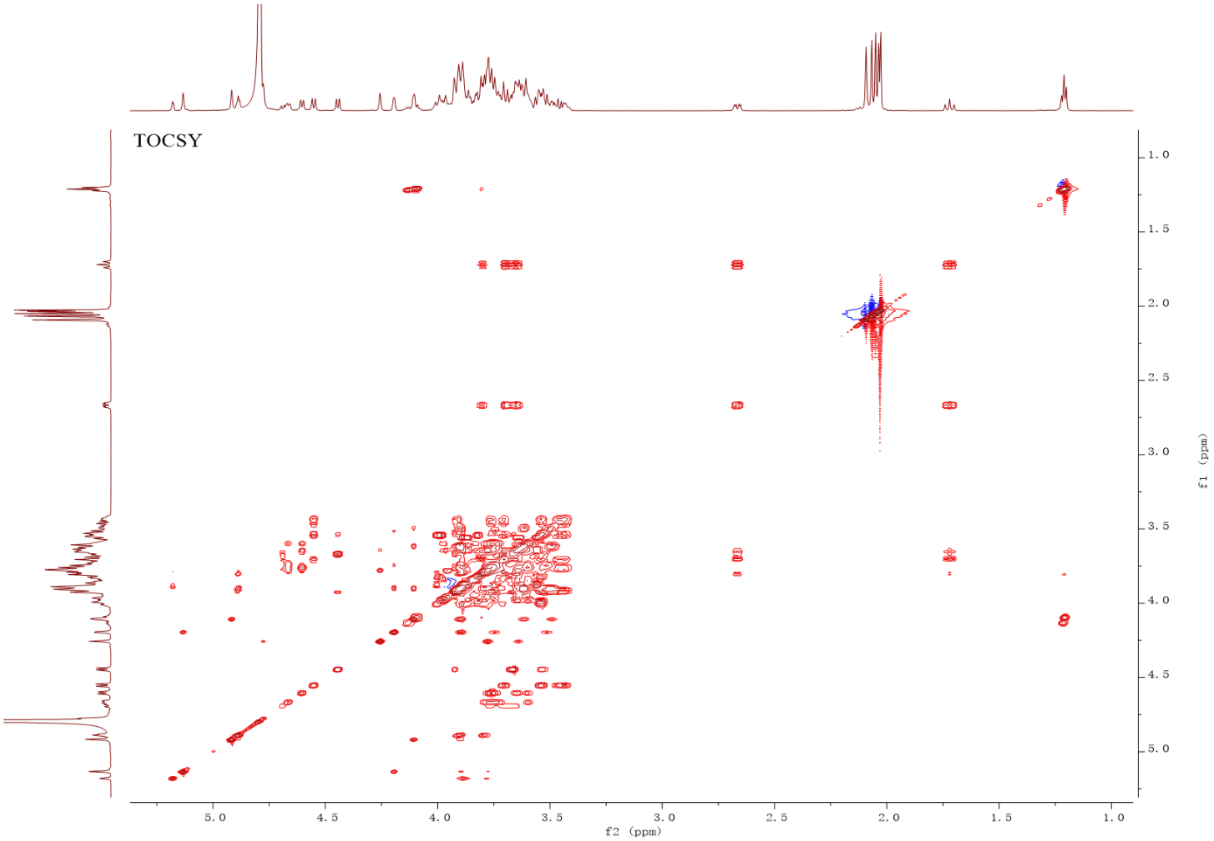
TOCSY
f2 (ppm)
f1 (ppm)

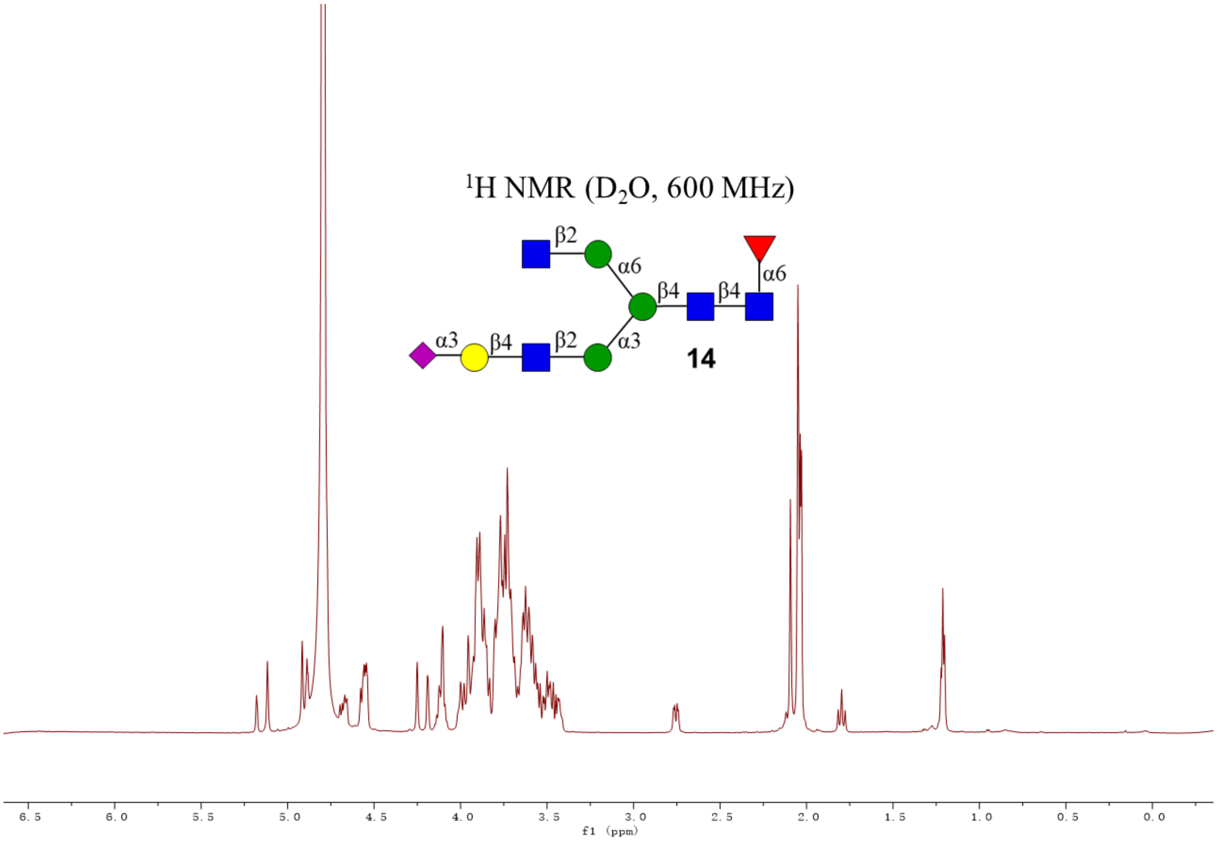

^{1}H NMR (D_2O, 600 MHz)
β2
α6
β4
β4
α6
α3
β4
β2
α3
14
6.5 6.0 5.5 5.0 4.5 4.0 3.5 3.0 2.5 2.0 1.5 1.0 0.5 0.0
f1 (ppm)

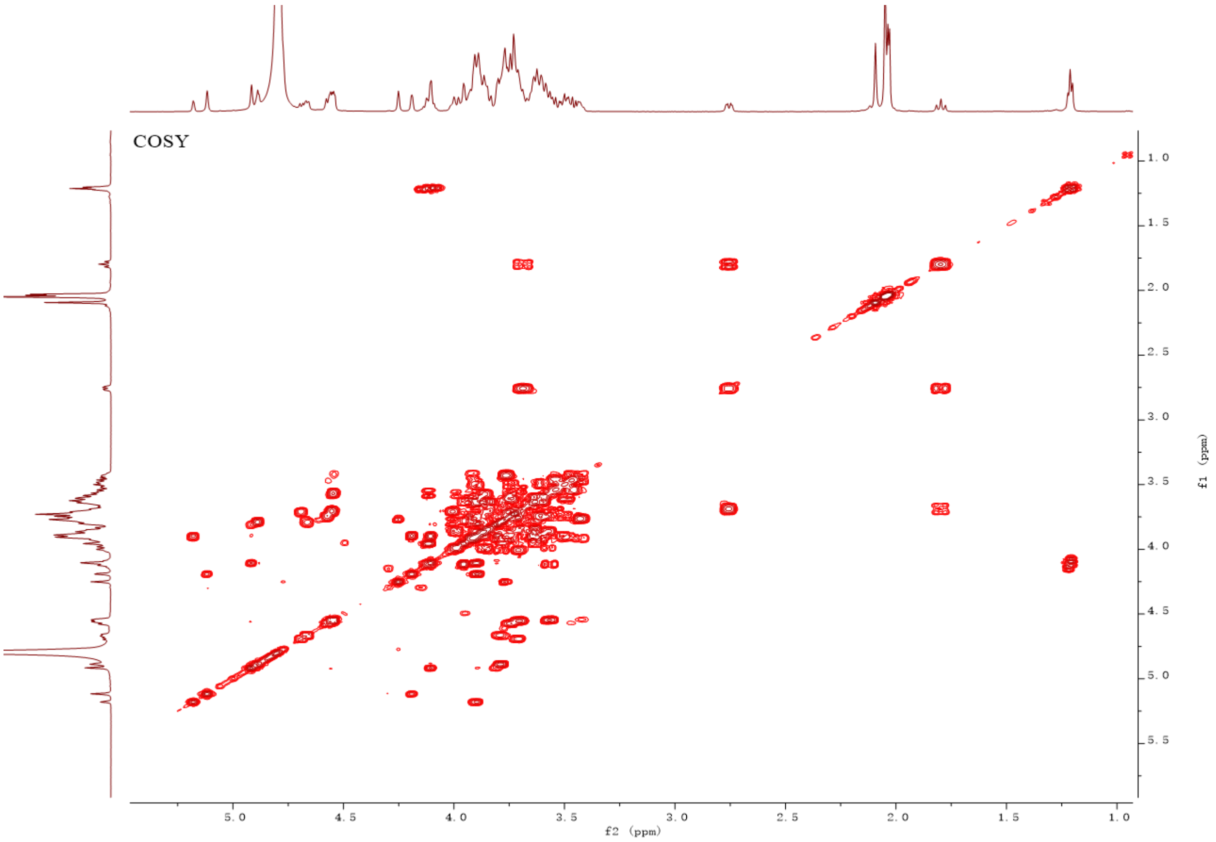

COSY
5.0 4.5 4.0 3.5 3.0 2.5 2.0 1.5 1.0
f2 (ppm)
1.0 1.5 2.0 2.5 3.0 3.5 4.0 4.5 5.0 5.5
f1 (ppm)

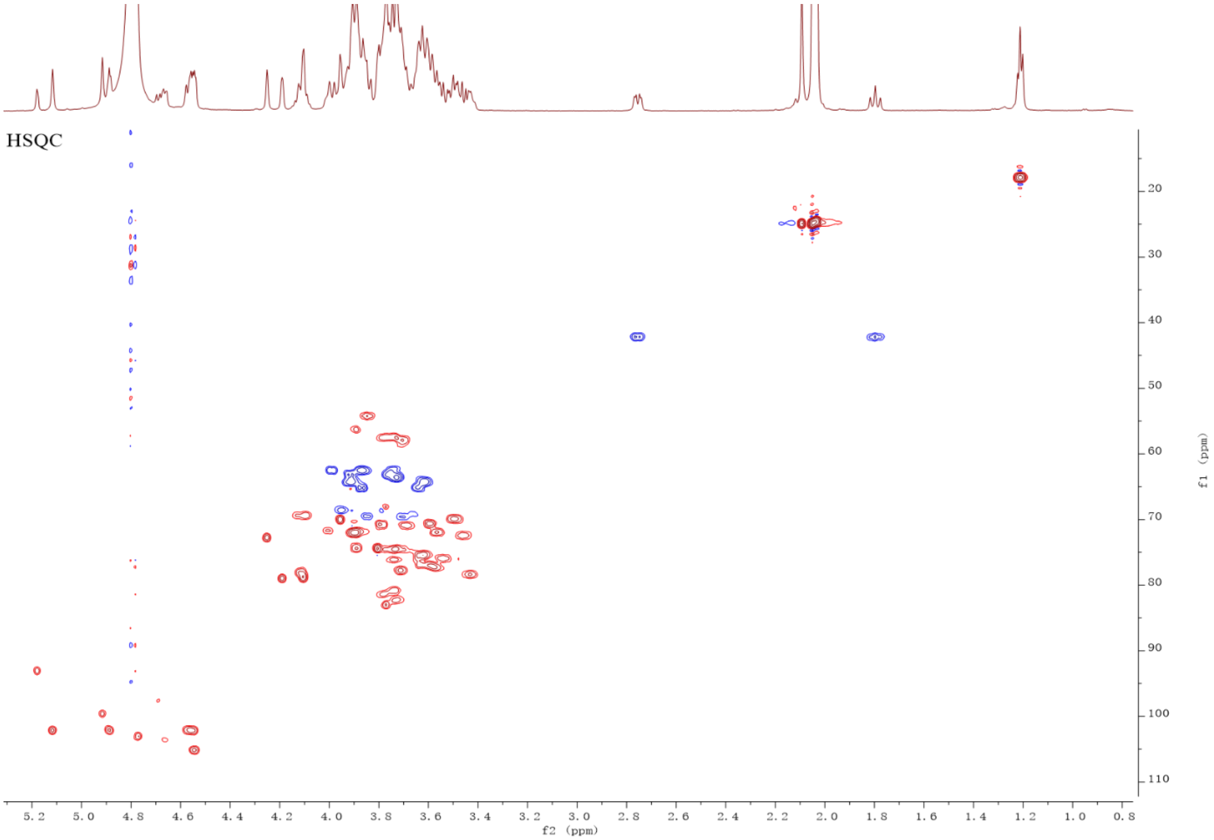
HSQC
f2 (ppm)
f1 (ppm)

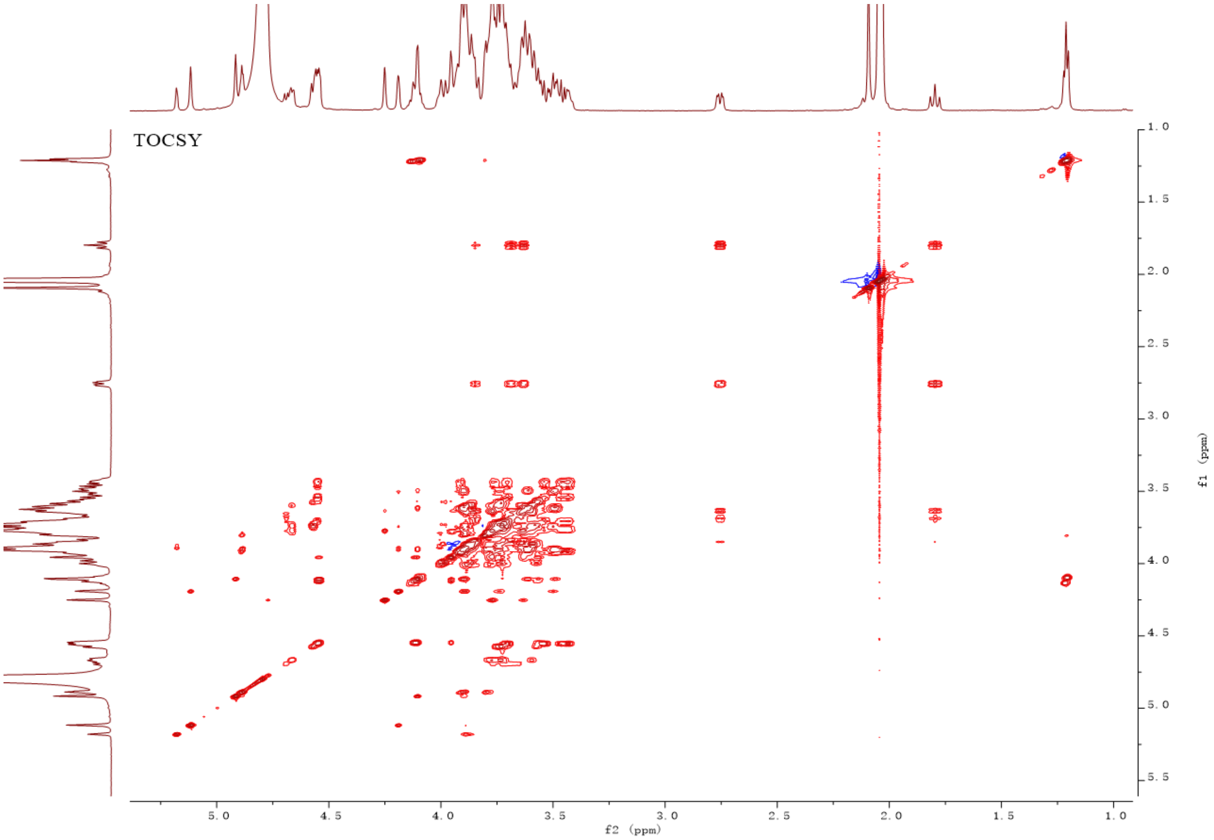
TOCSY
f2 (ppm)
f1 (ppm)

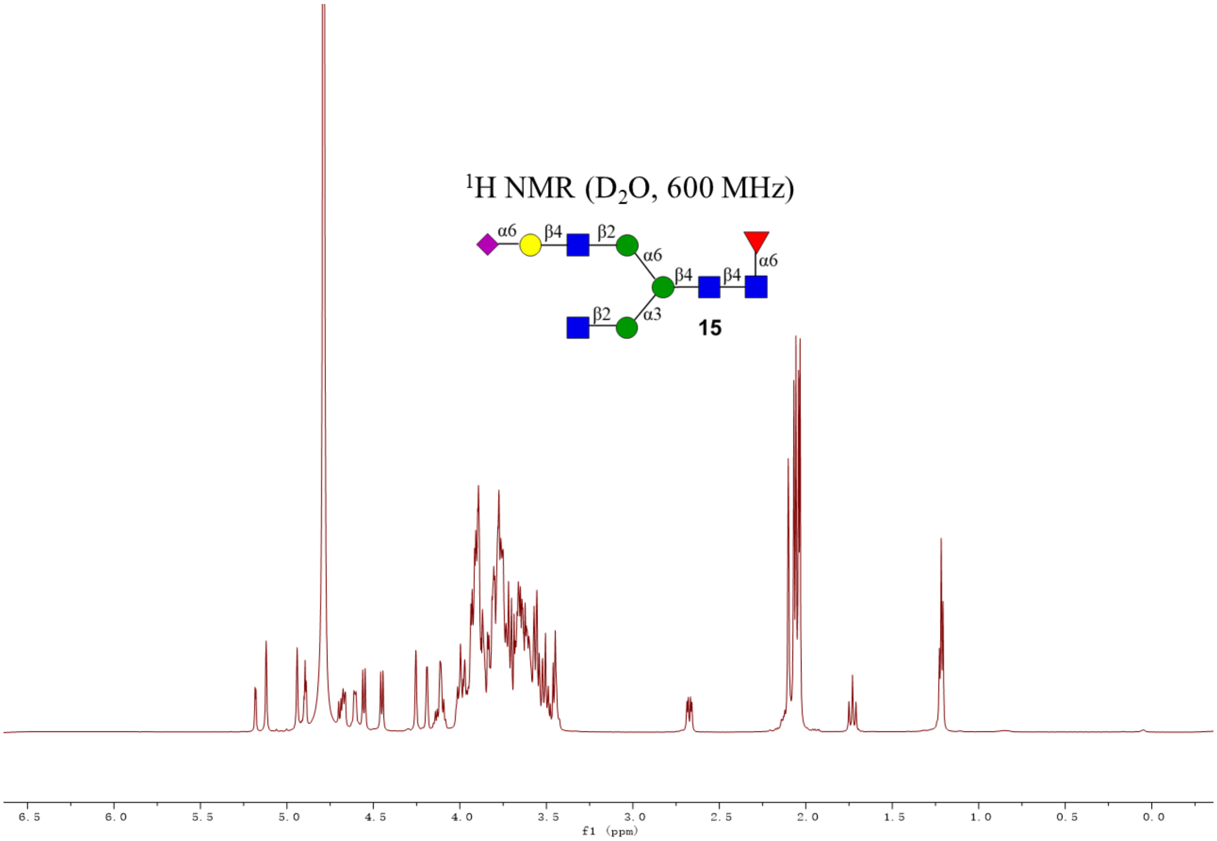

^{1}H NMR (D_2O, 600 MHz)
α6
β4
β2
α6
β4
β4
α6
β2
α3
15
f1 (ppm)

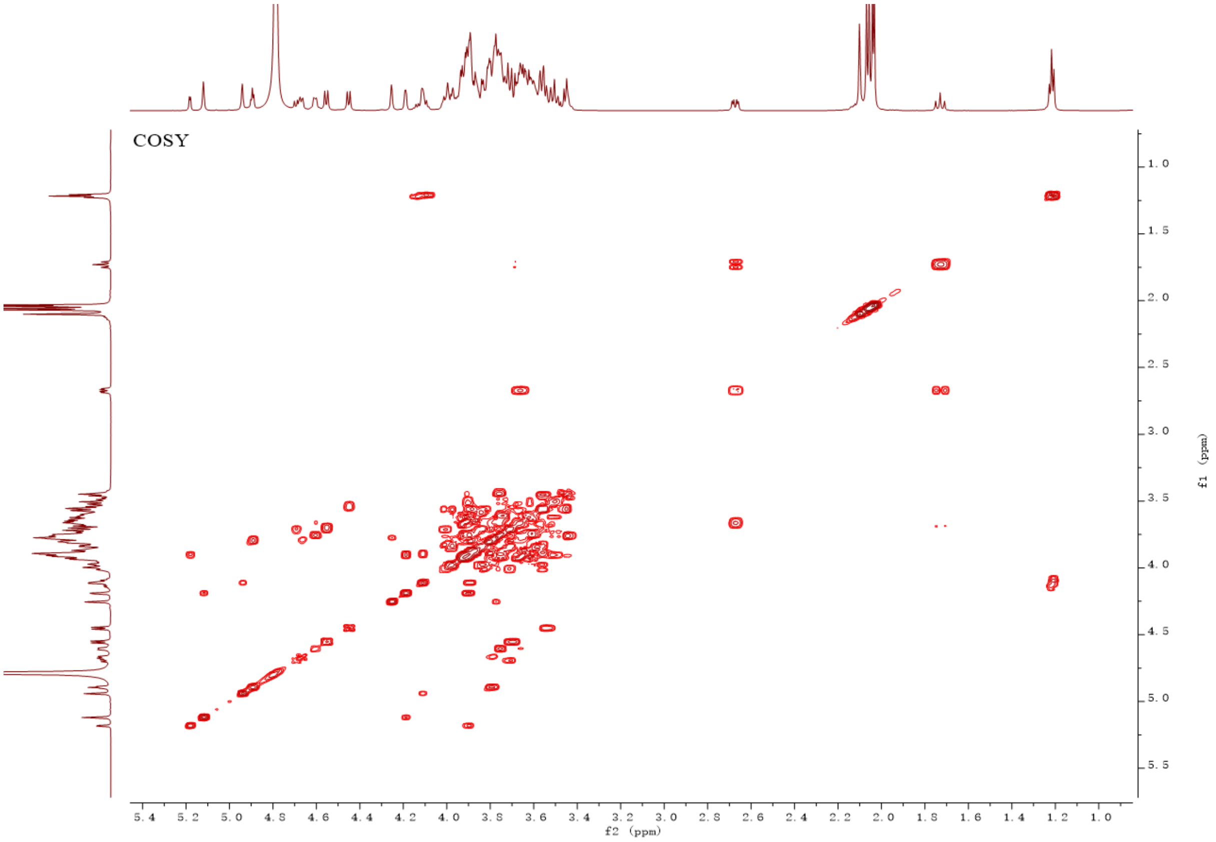

COSY
f2 (ppm)
f1 (ppm)

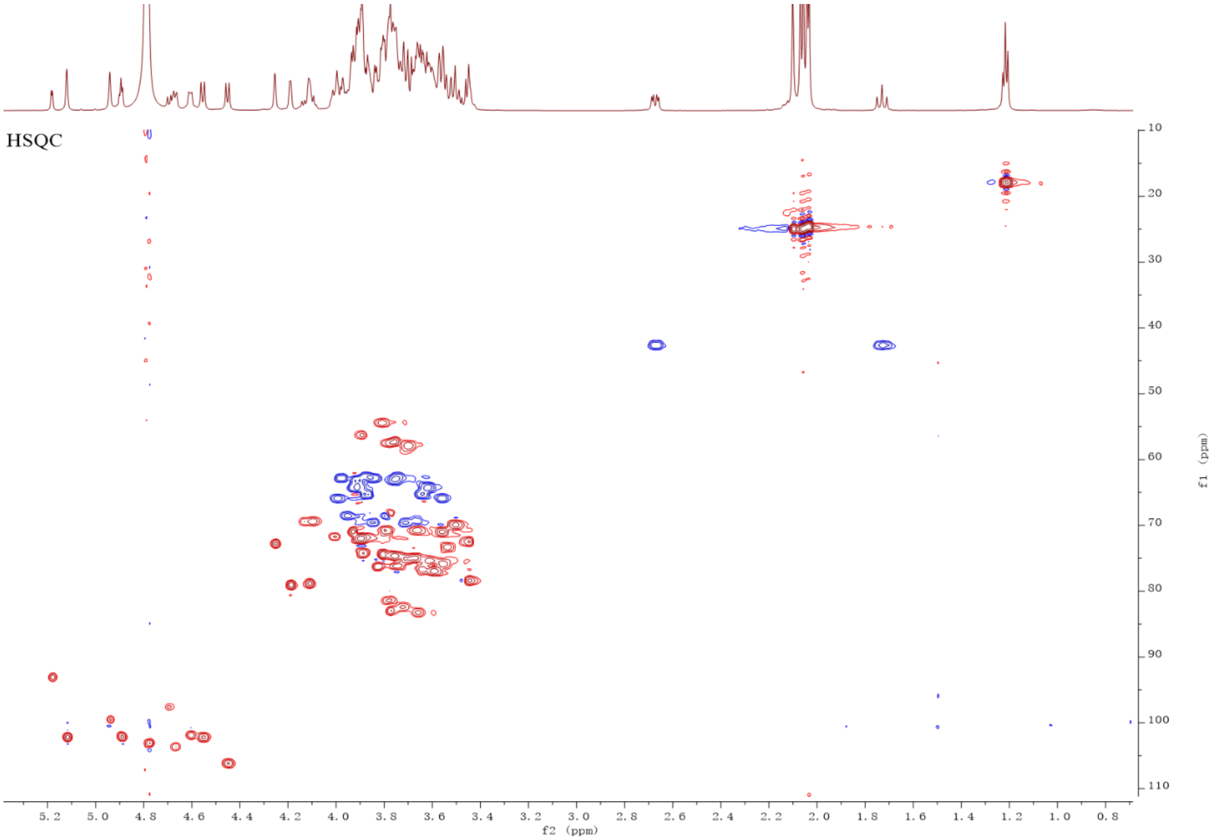
HSQC
f2 (ppm)
f1 (ppm)

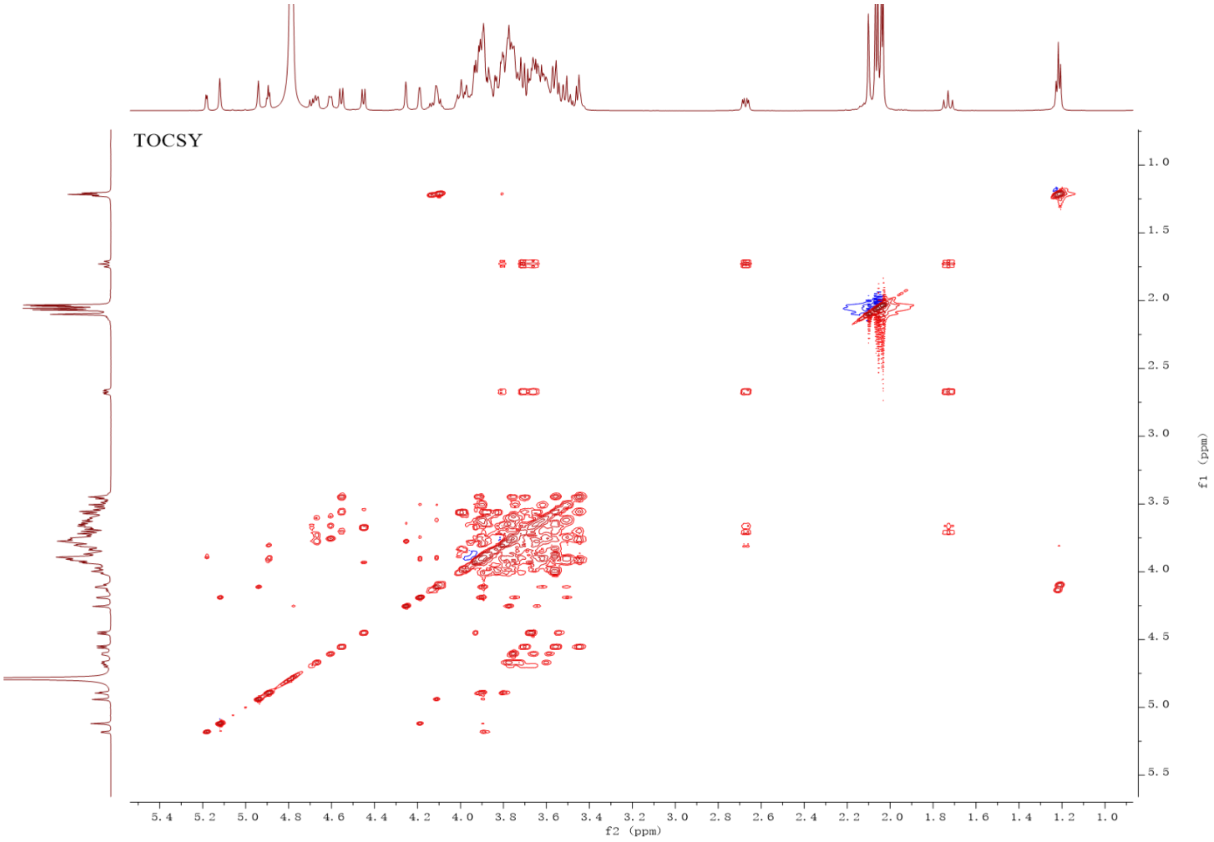
TOCSY
f2 (ppm)
f1 (ppm)

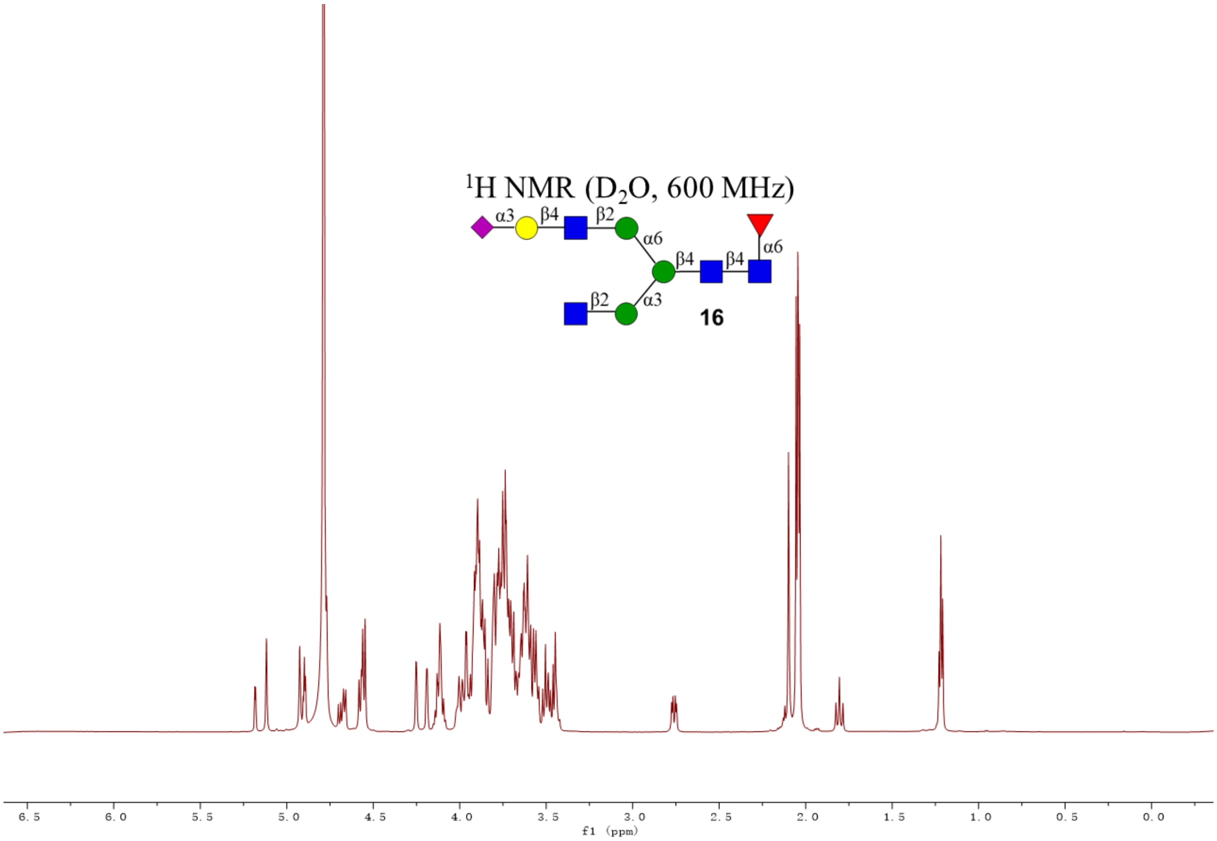

^{1}H NMR (D_2O, 600 MHz)
16
f1 (ppm)

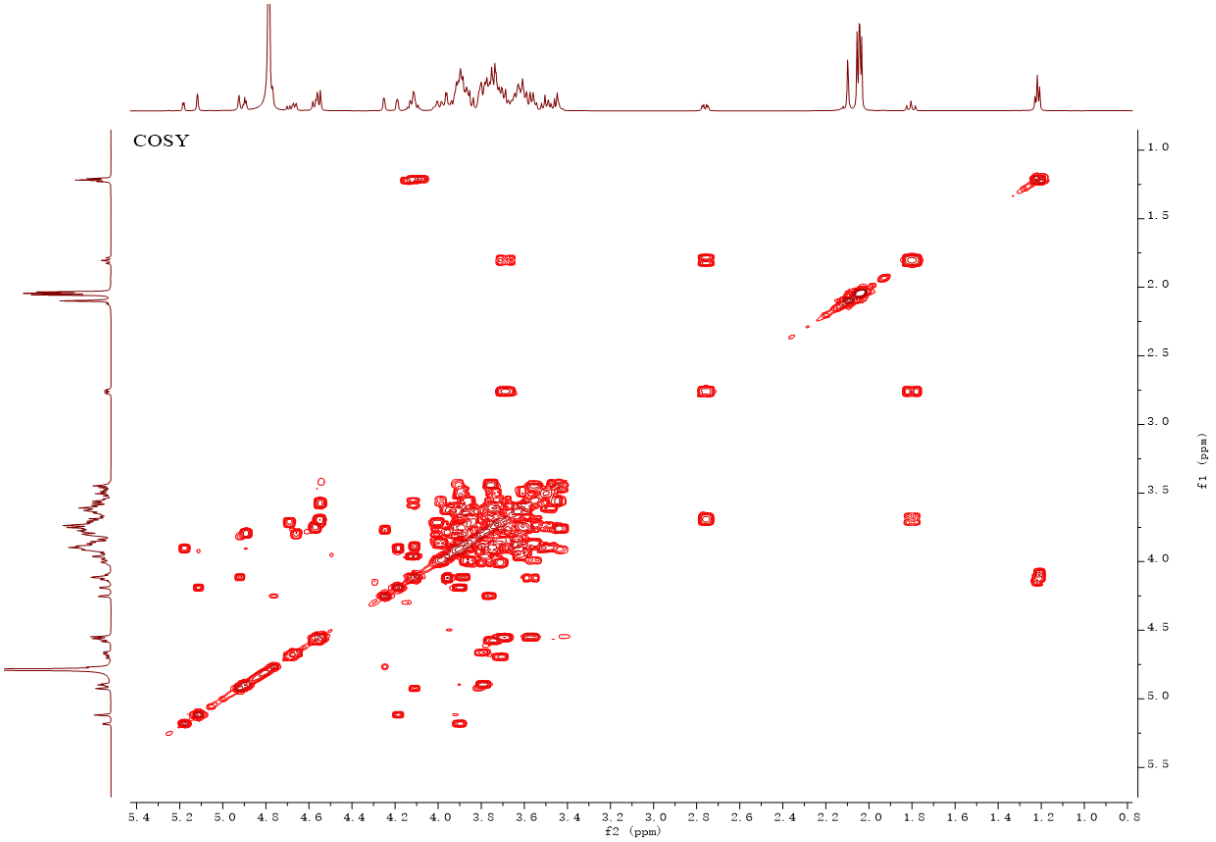

COSY
f2 (ppm)
f1 (ppm)

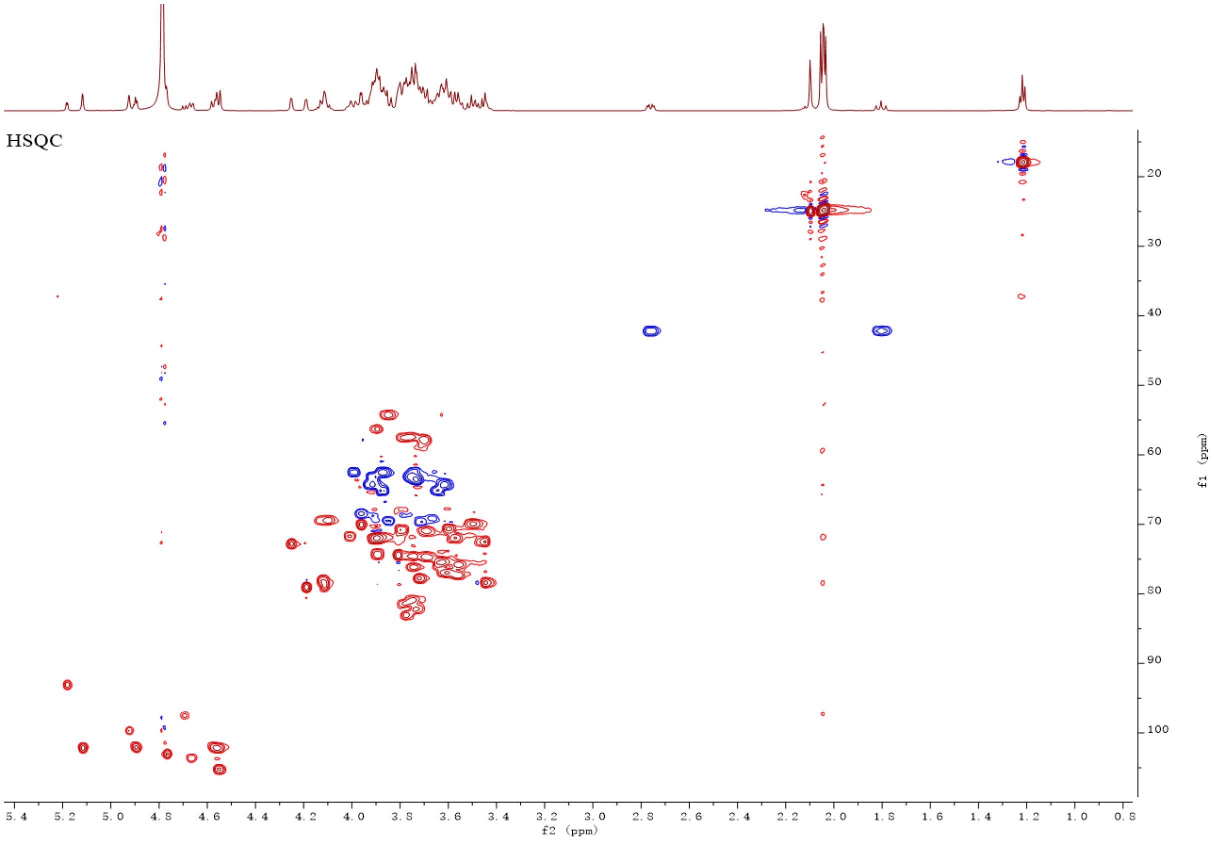
HSQC
f2 (ppm)
f1 (ppm)

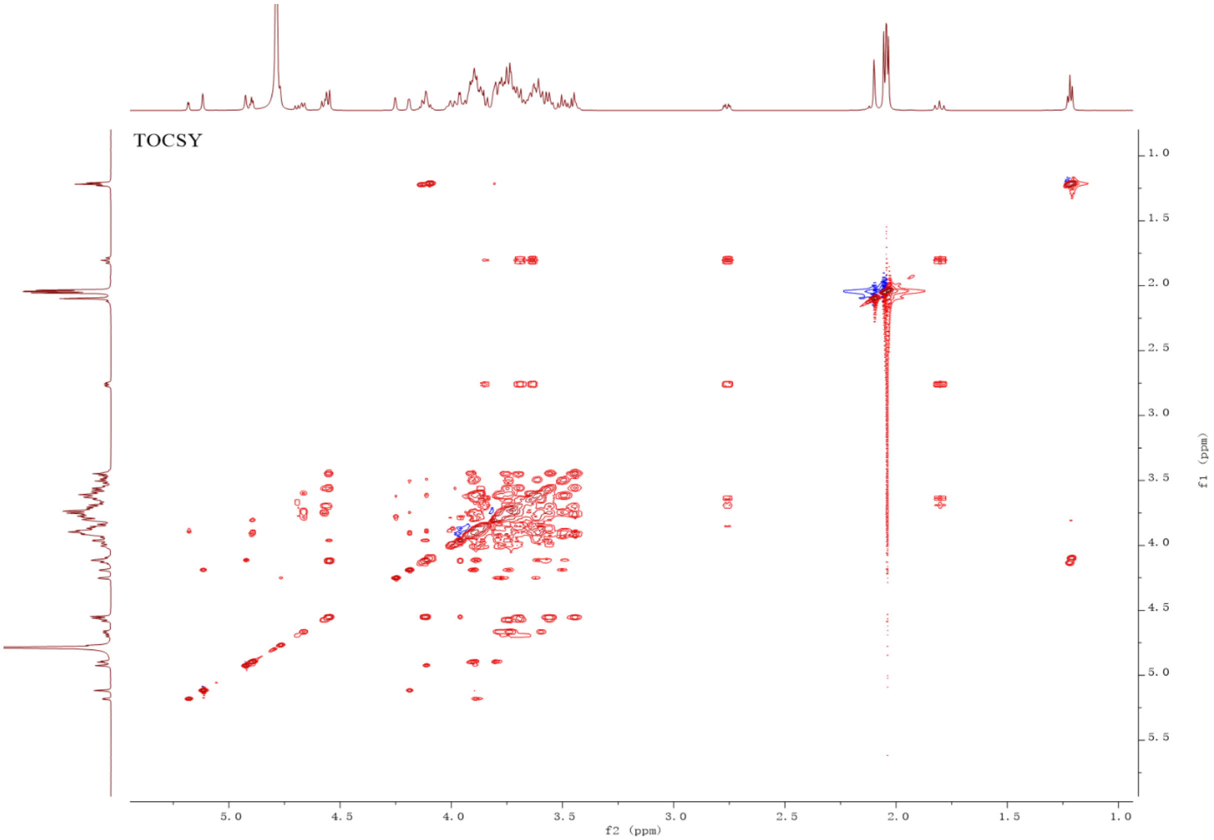
TOCSY
f2 (ppm)
f1 (ppm)

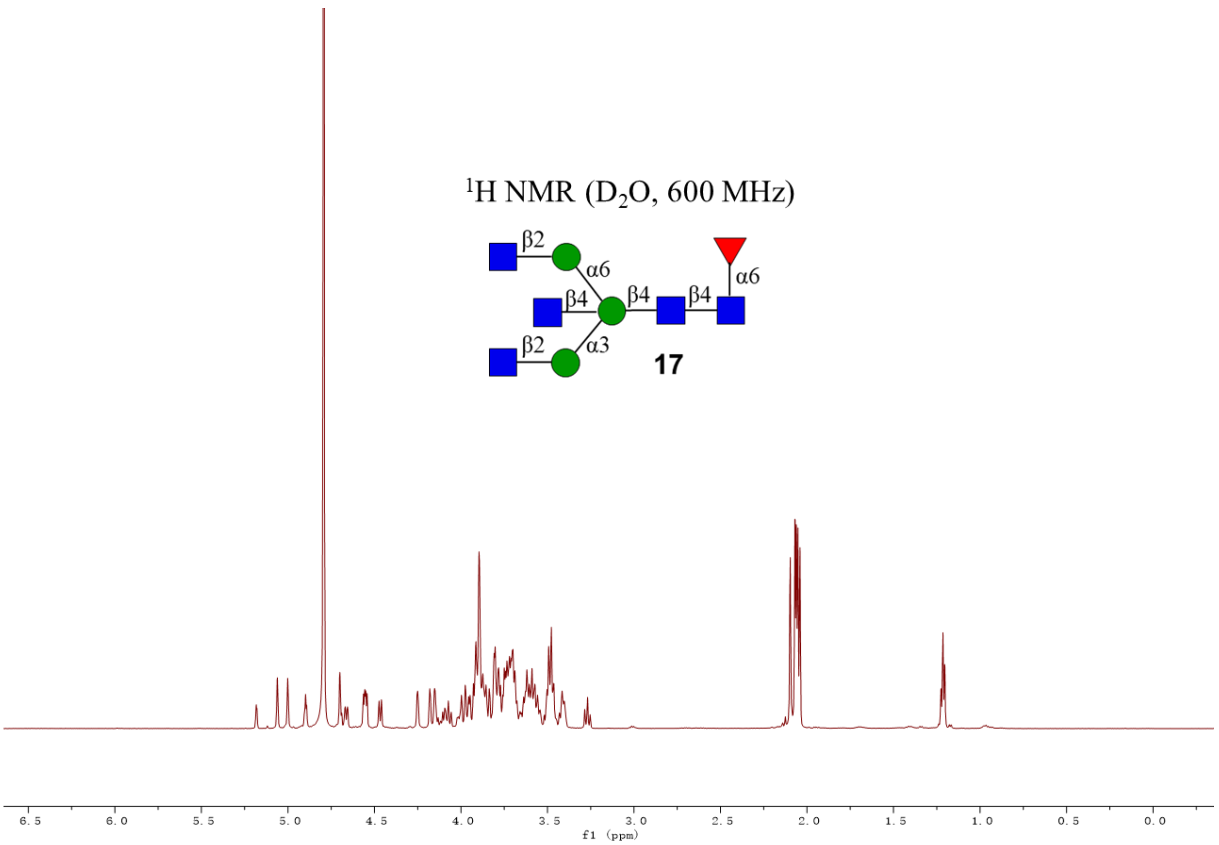

^{1}H NMR (D_2O, 600 MHz)
β2
α6
β4
β4
β4
α6
β2
α3
17
f1 (ppm)

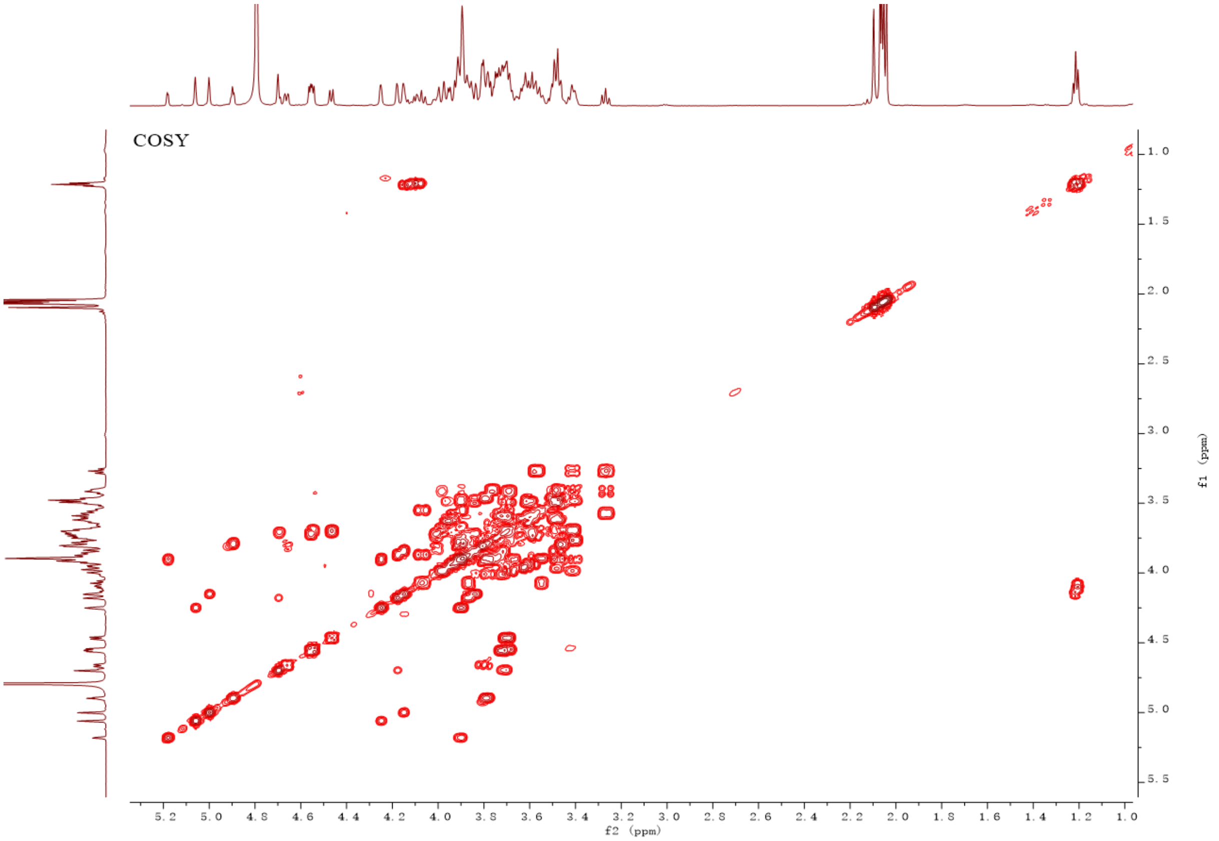

COSY
f2 (ppm)
f1 (ppm)

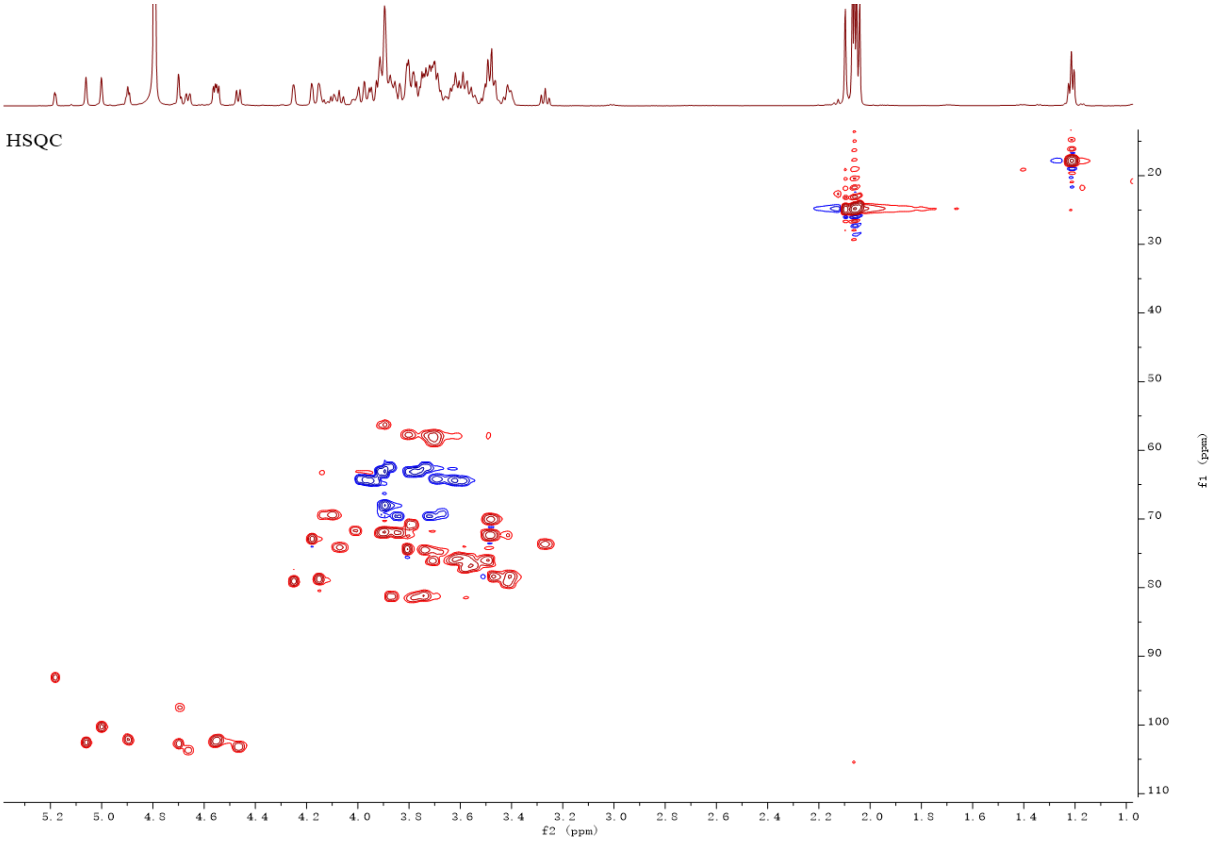
HSQC
f2 (ppm)
f1 (ppm)

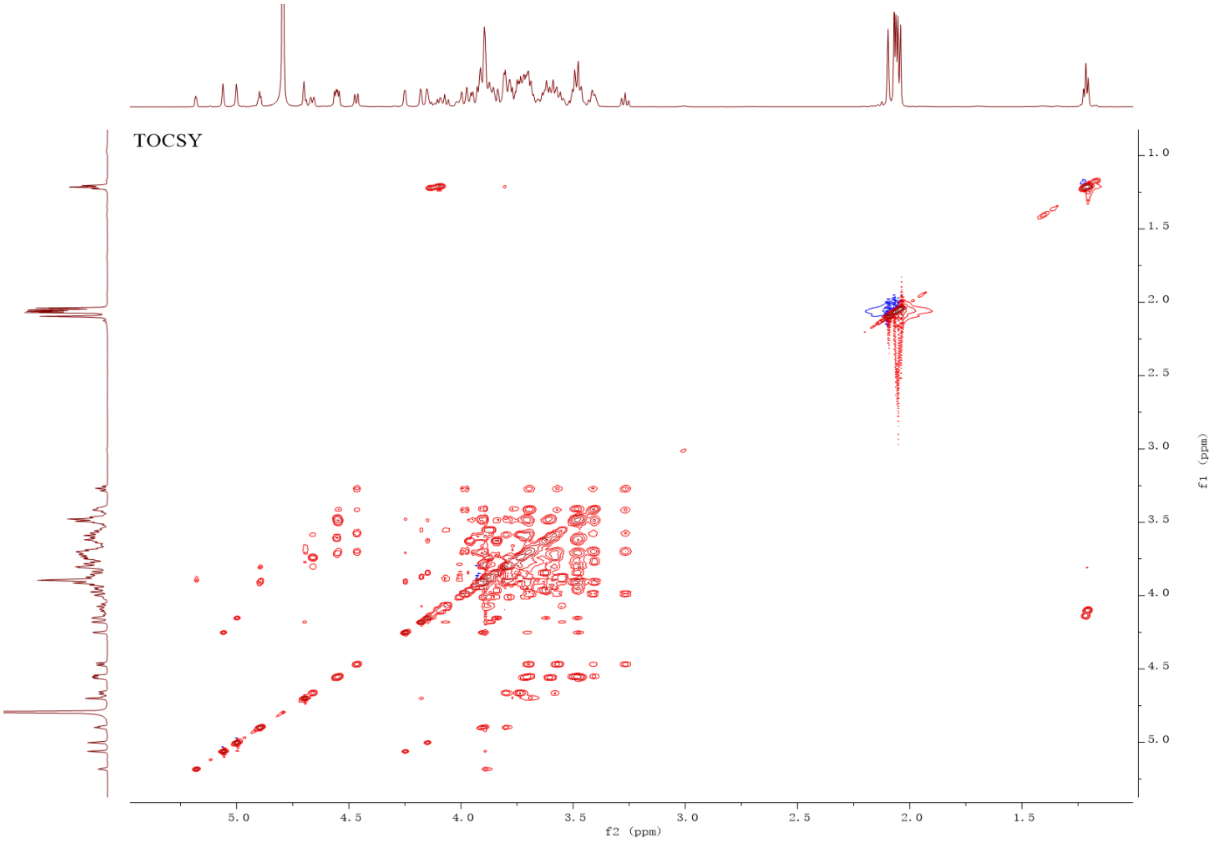
TOCSY
f2 (ppm)
f1 (ppm)

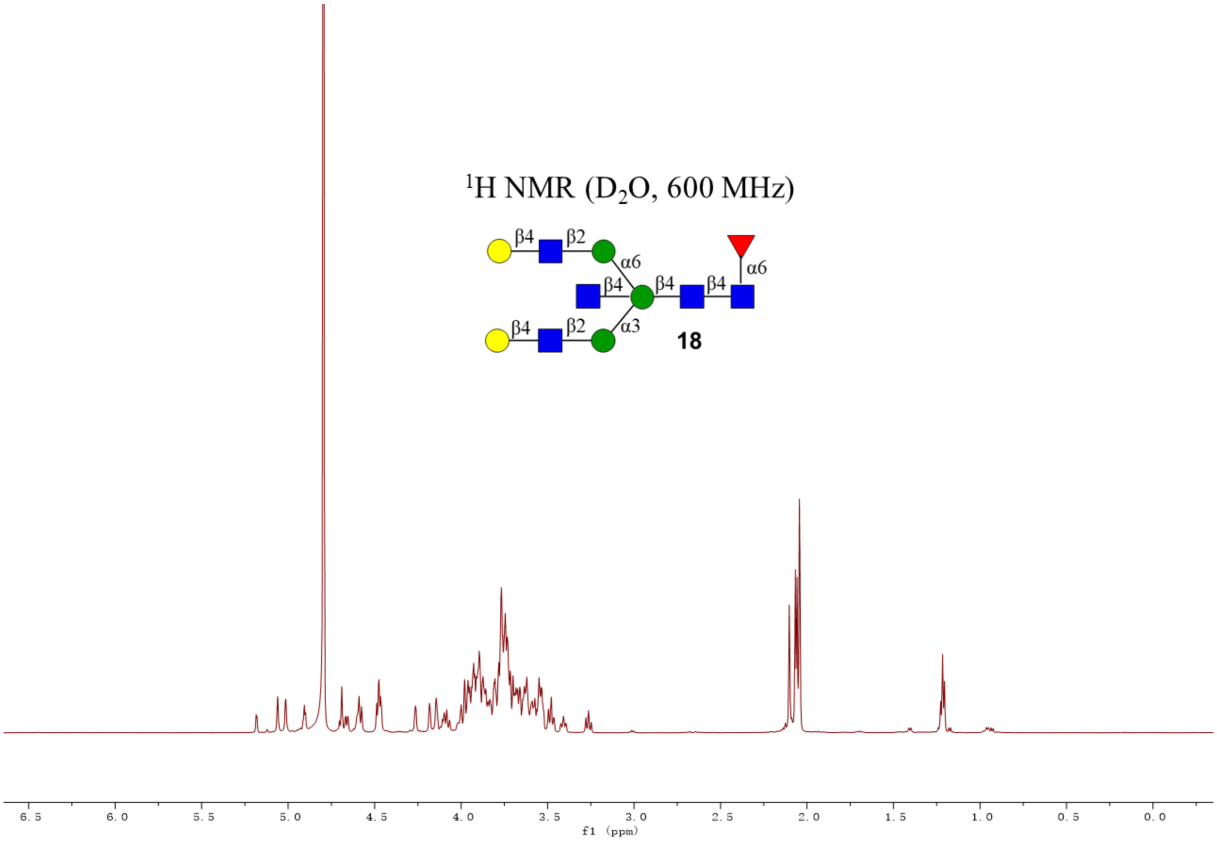
^{1}H NMR (D_2O, 600 MHz)
β4
β2
α6
β4
β4
β4
α6
β4
β2
α3
18
f1 (ppm)

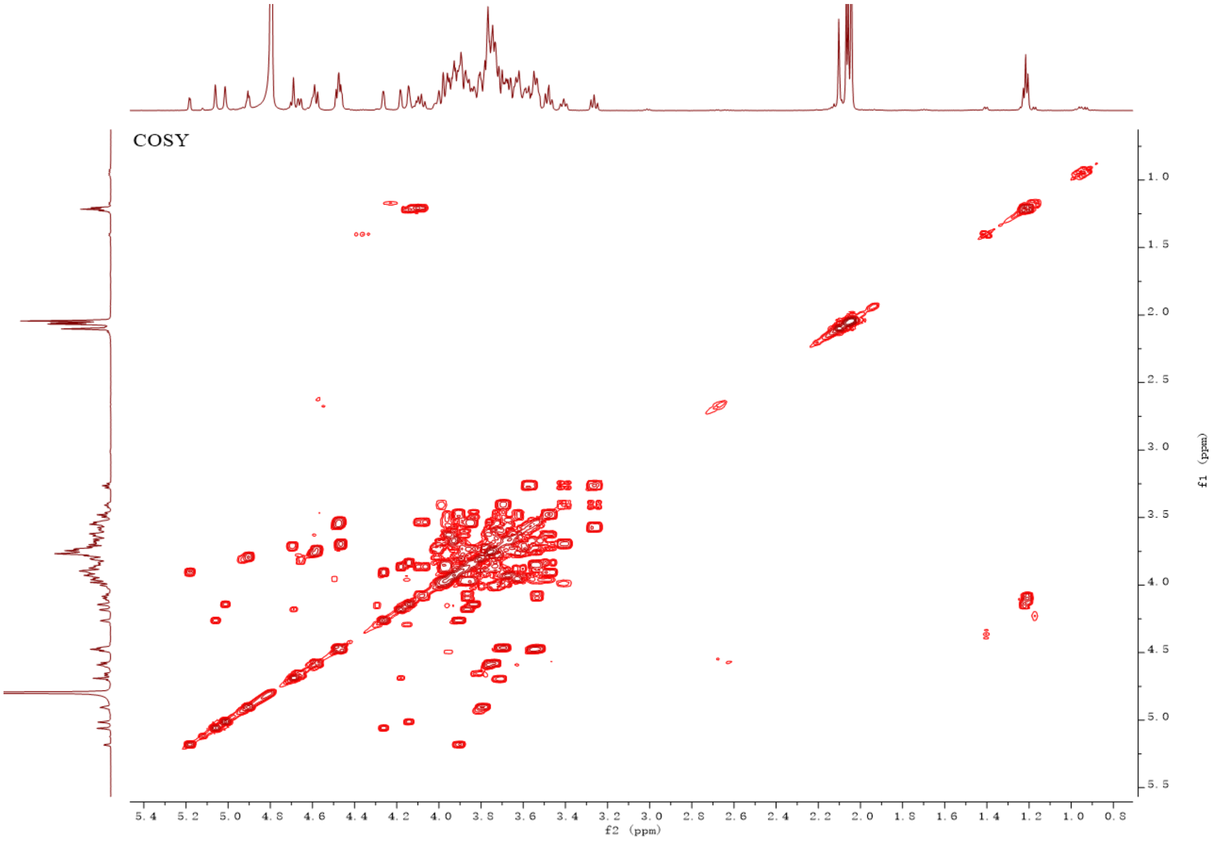
COSY
f2 (ppm)
f1 (ppm)

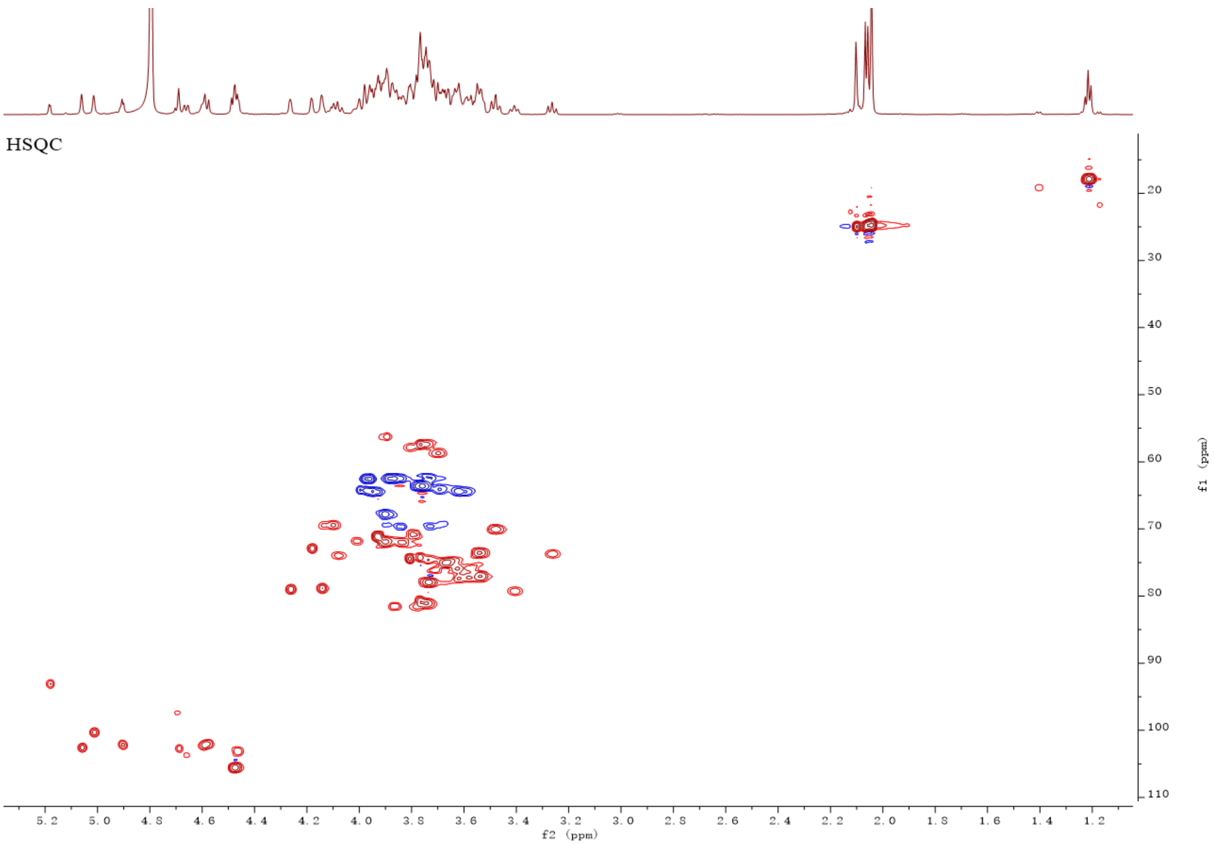
HSQC
f2 (ppm)
f1 (ppm)

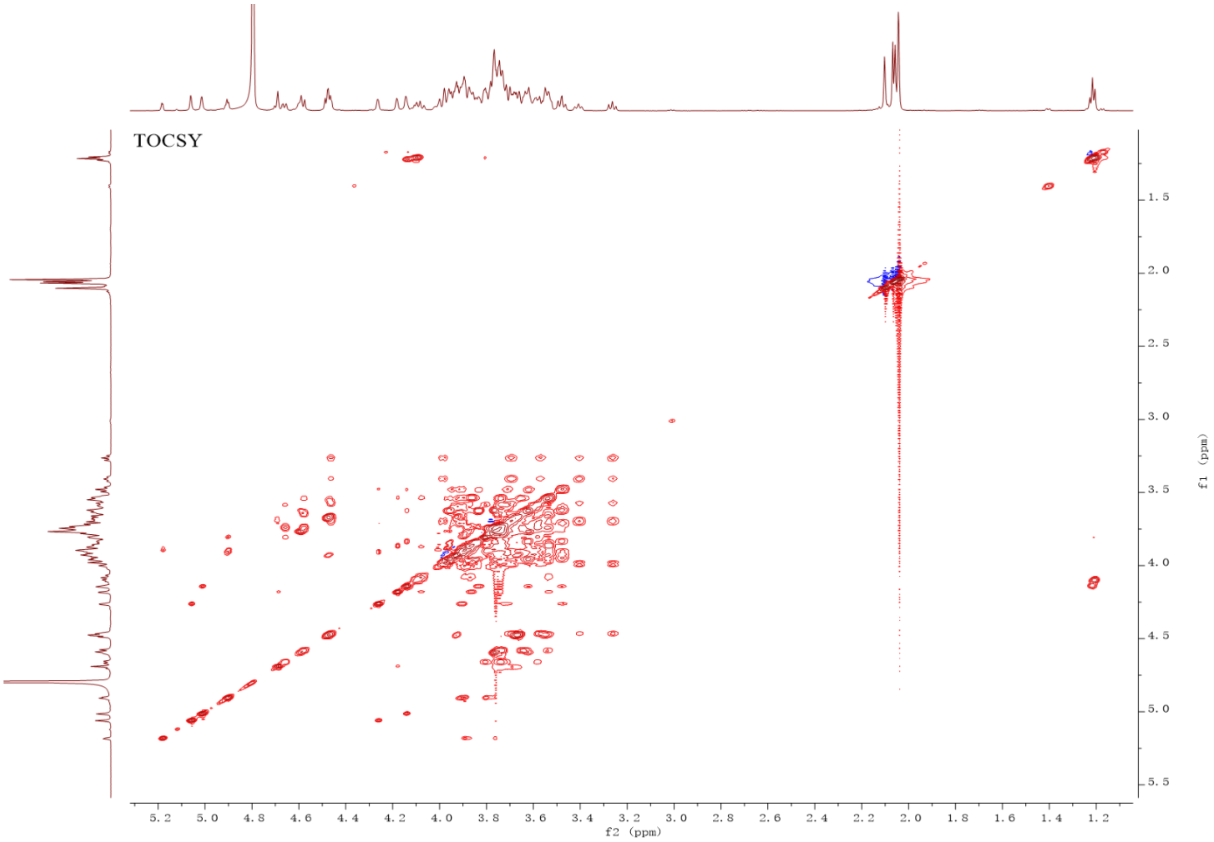
TOCSY
f2 (ppm)
f1 (ppm)

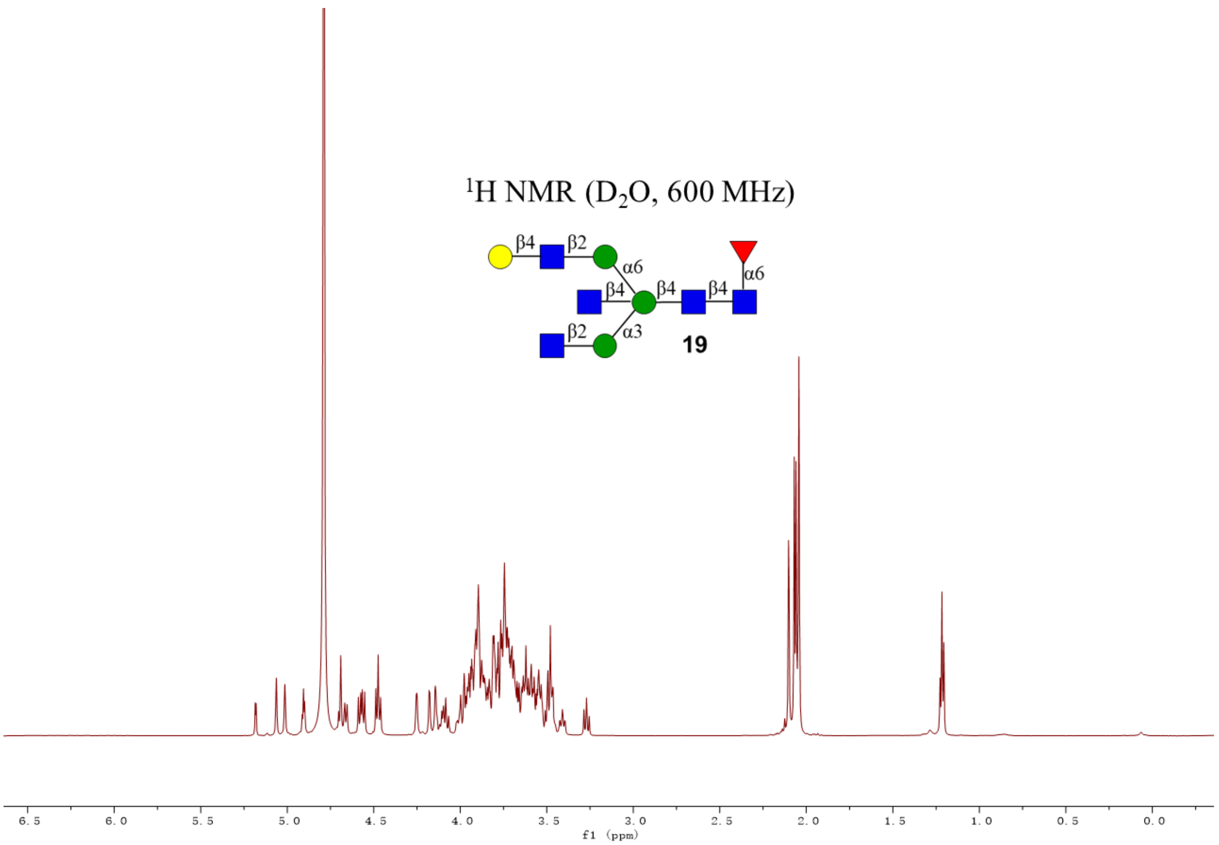

^{1}H NMR (D_2O, 600 MHz)
β4
β2
α6
β4
β4
β4
α6
β2
α3
19
f1 (ppm)

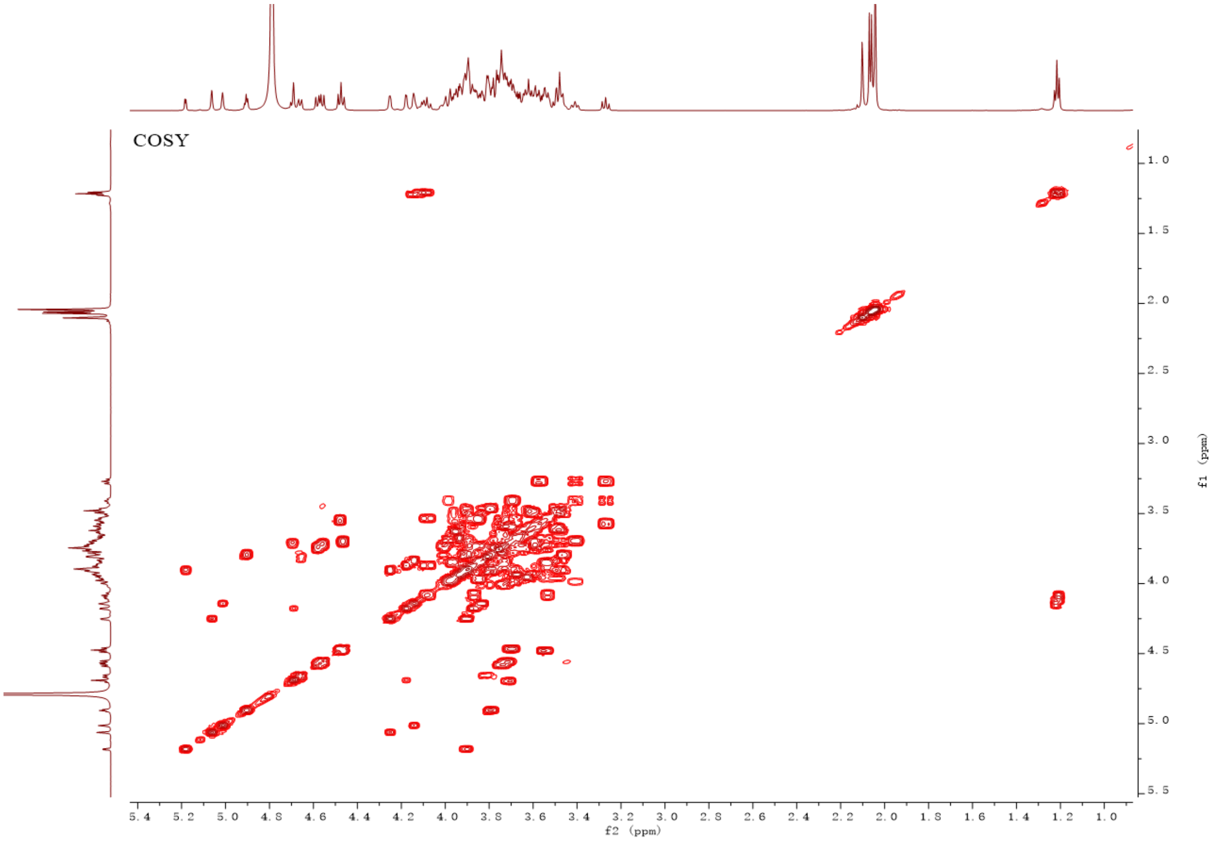

COSY
f2 (ppm)
f1 (ppm)

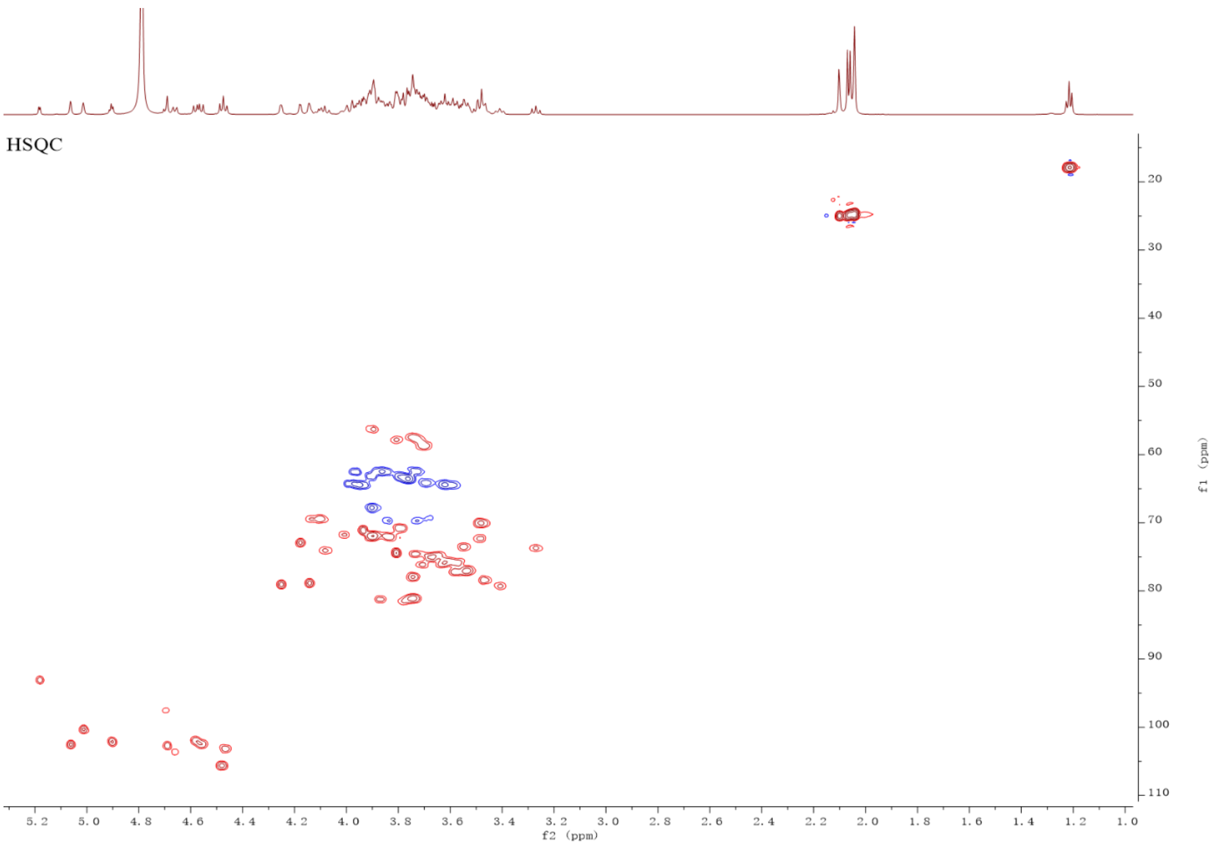
HSQC
f2 (ppm)
f1 (ppm)

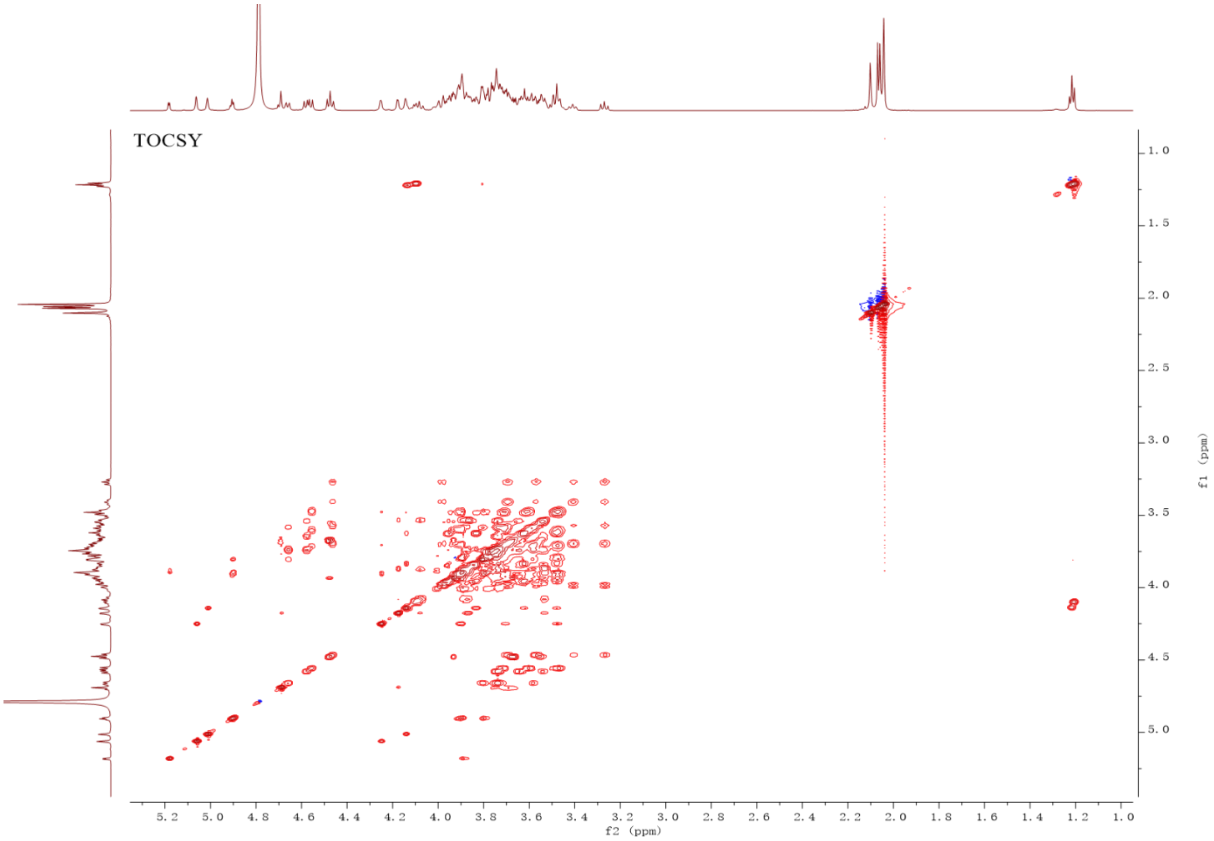
TOCSY
f2 (ppm)
f1 (ppm)

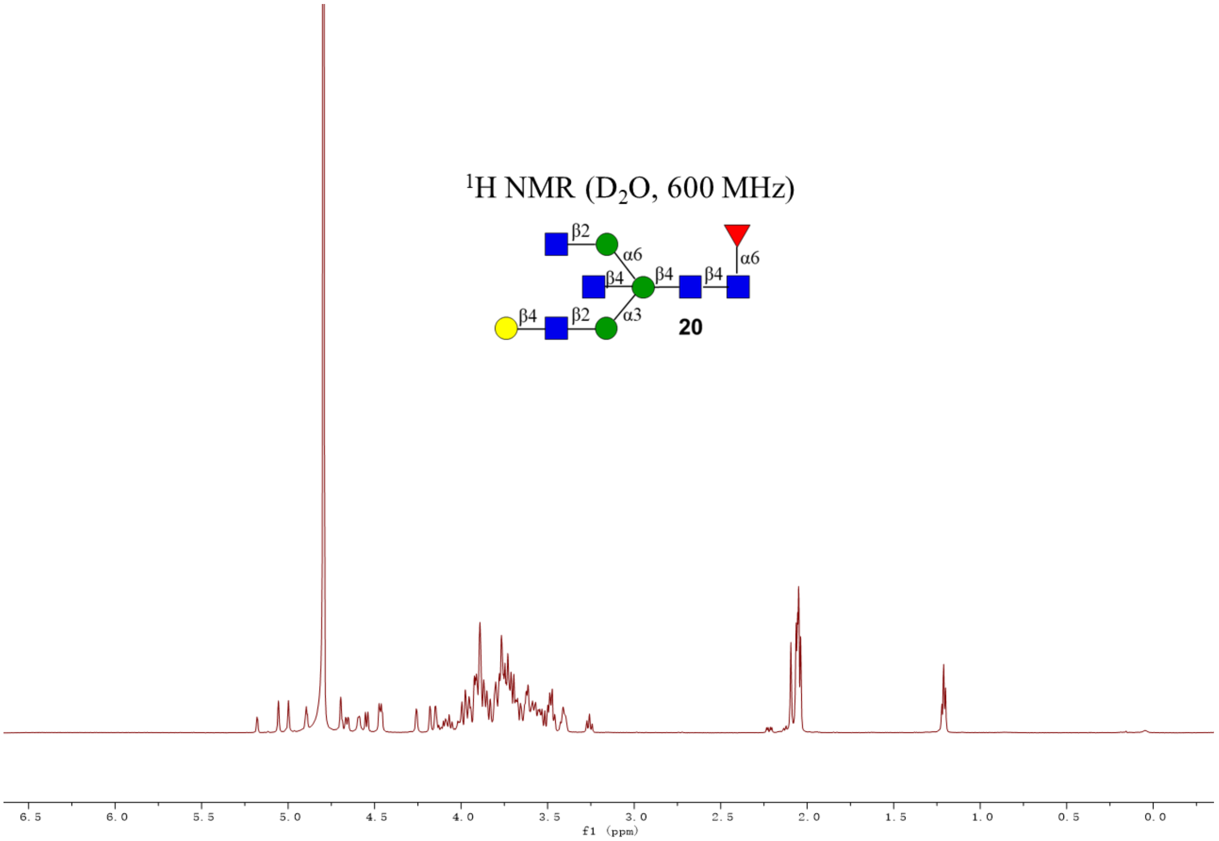

^{1}H NMR (D_2O, 600 MHz)
β2
α6
β4
β4
β4
α6
β4
β2
α3
20
f1 (ppm)

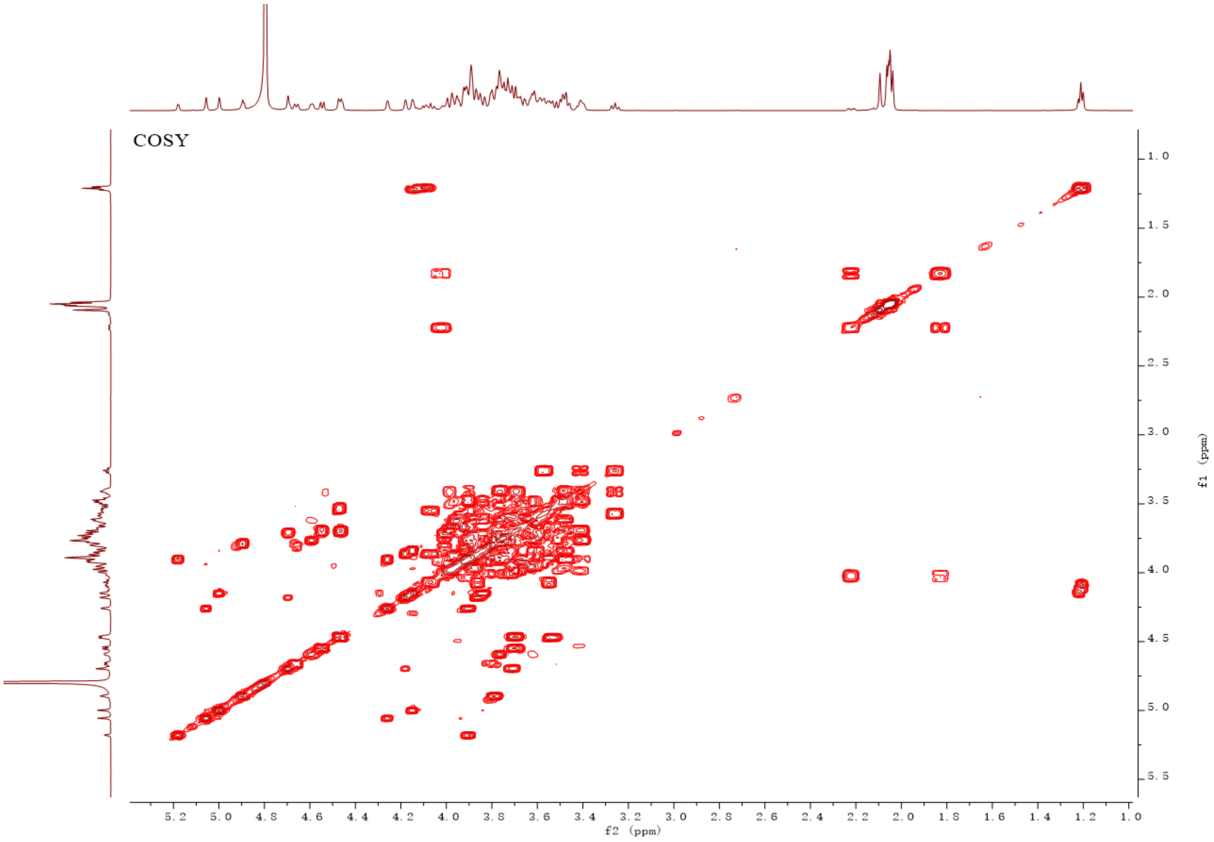

COSY
f2 (ppm)
f1 (ppm)

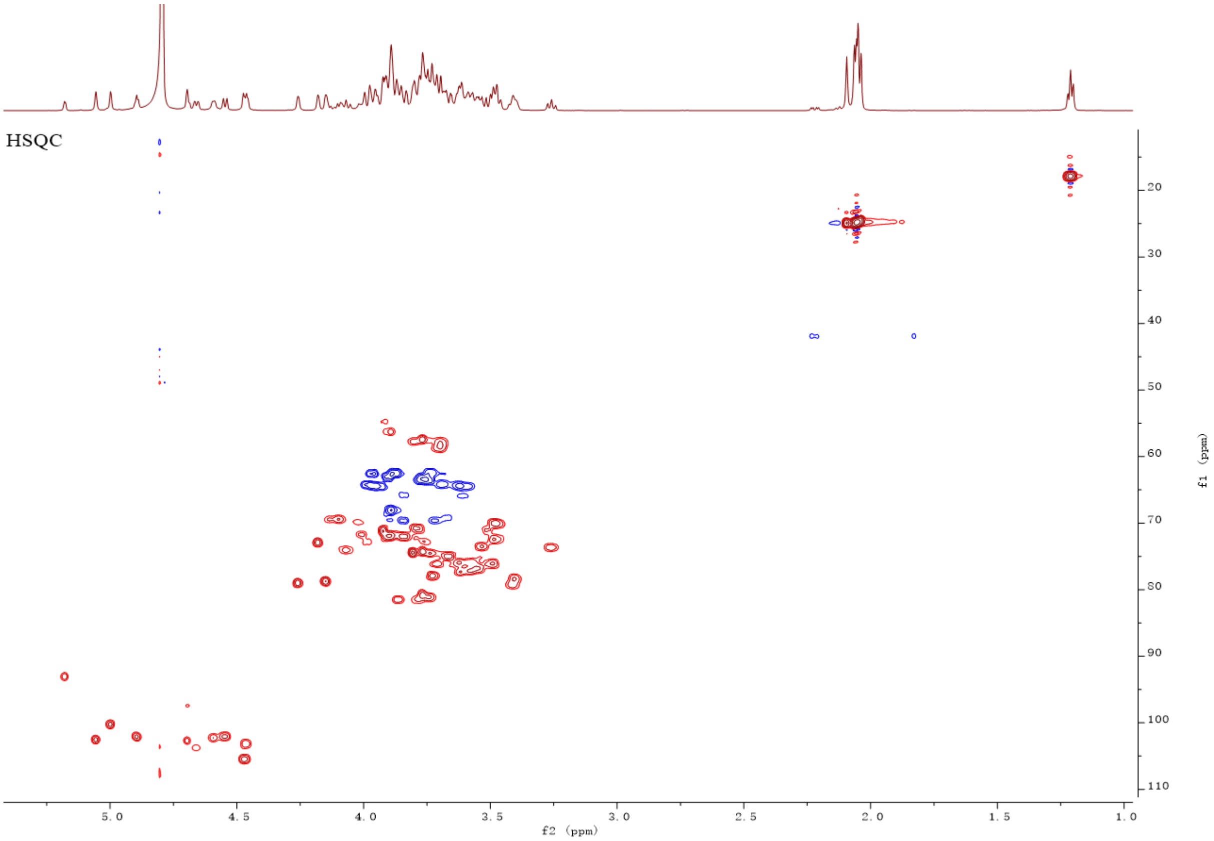
HSQC
f2 (ppm)
f1 (ppm)

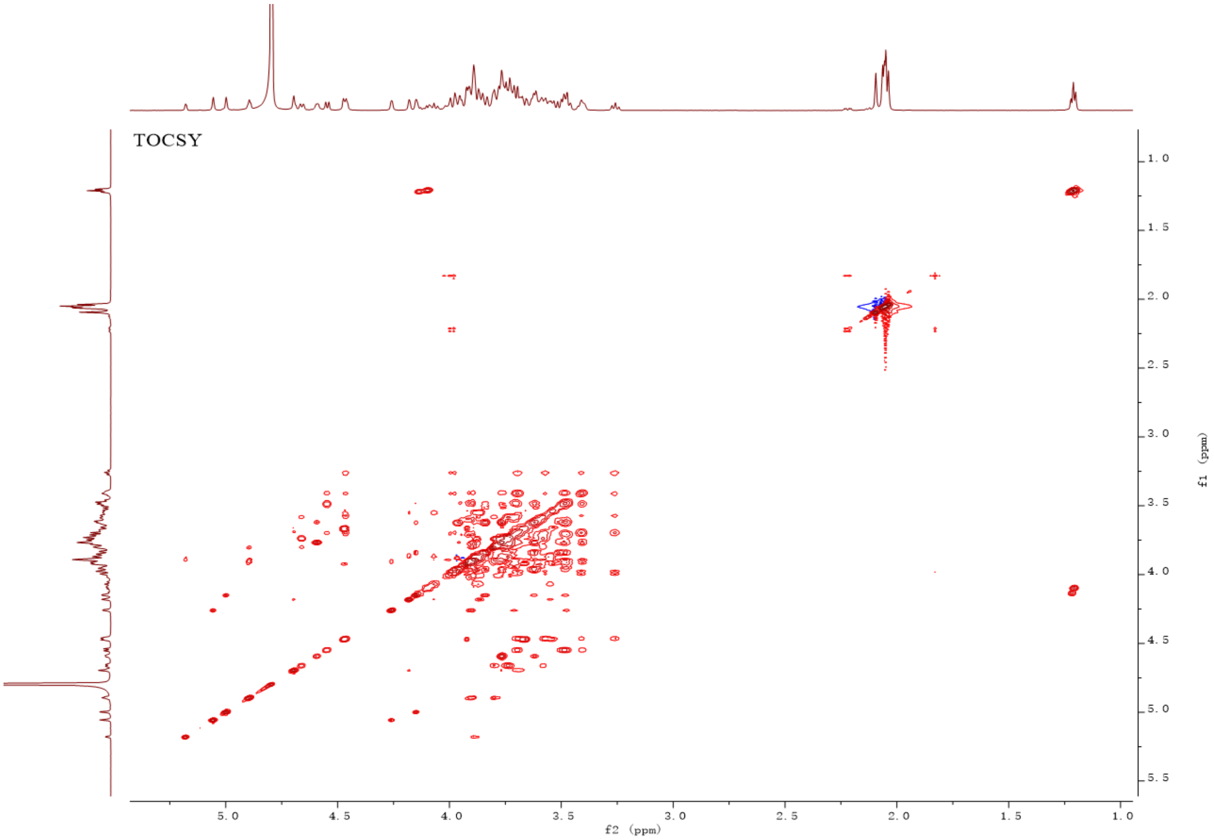
TOCSY
f2 (ppm)
f1 (ppm)

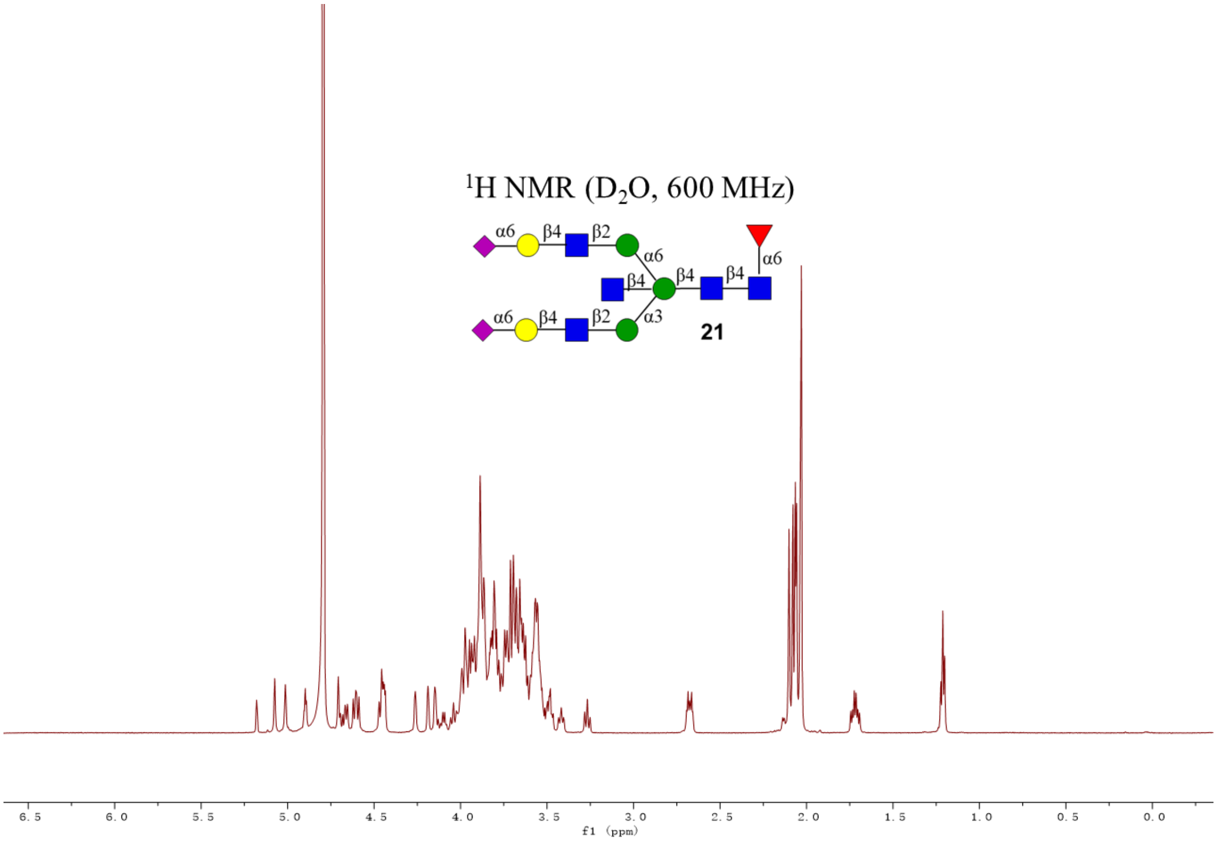

1H NMR (D2O, 600 MHz)
α6
β4
β2
α6
β4
β4
α6
β4
α6
β4
β2
α3
21
f1 (ppm)

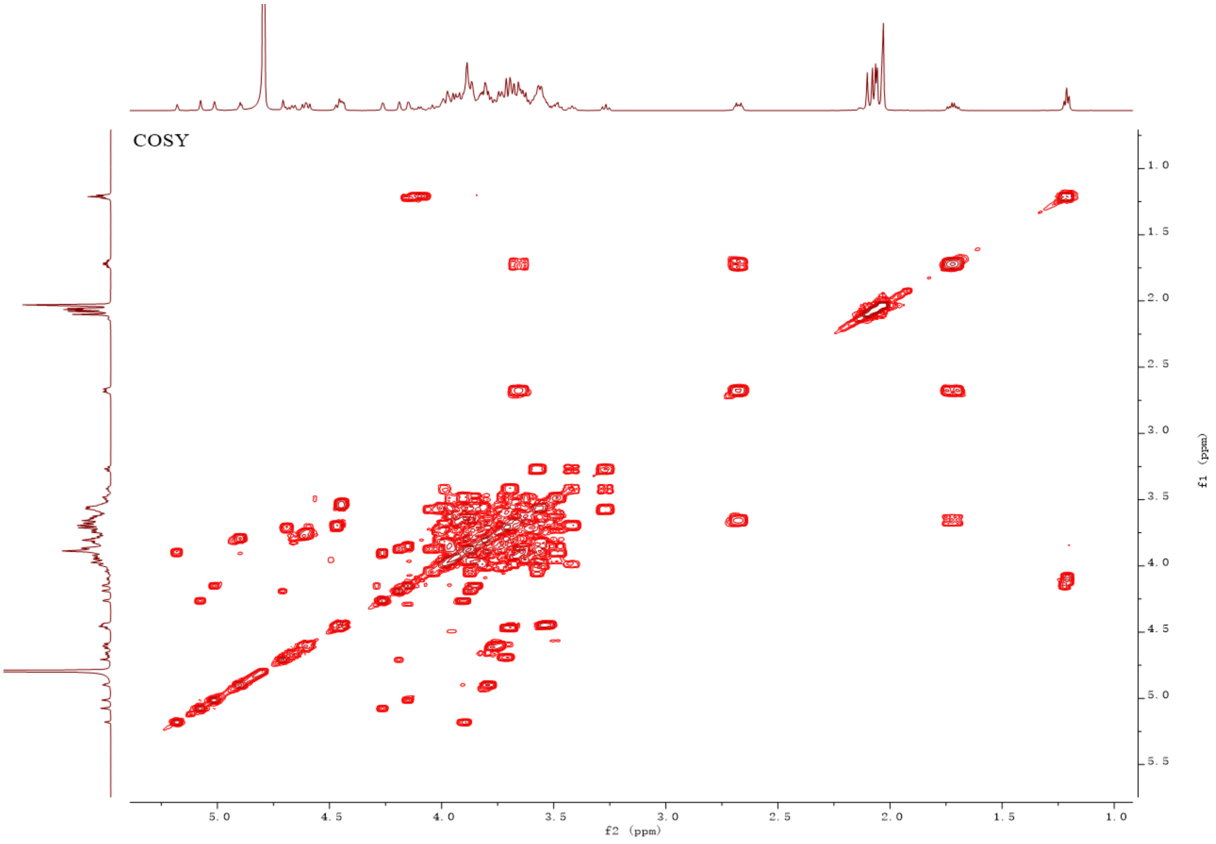

COSY
f2 (ppm)
f1 (ppm)

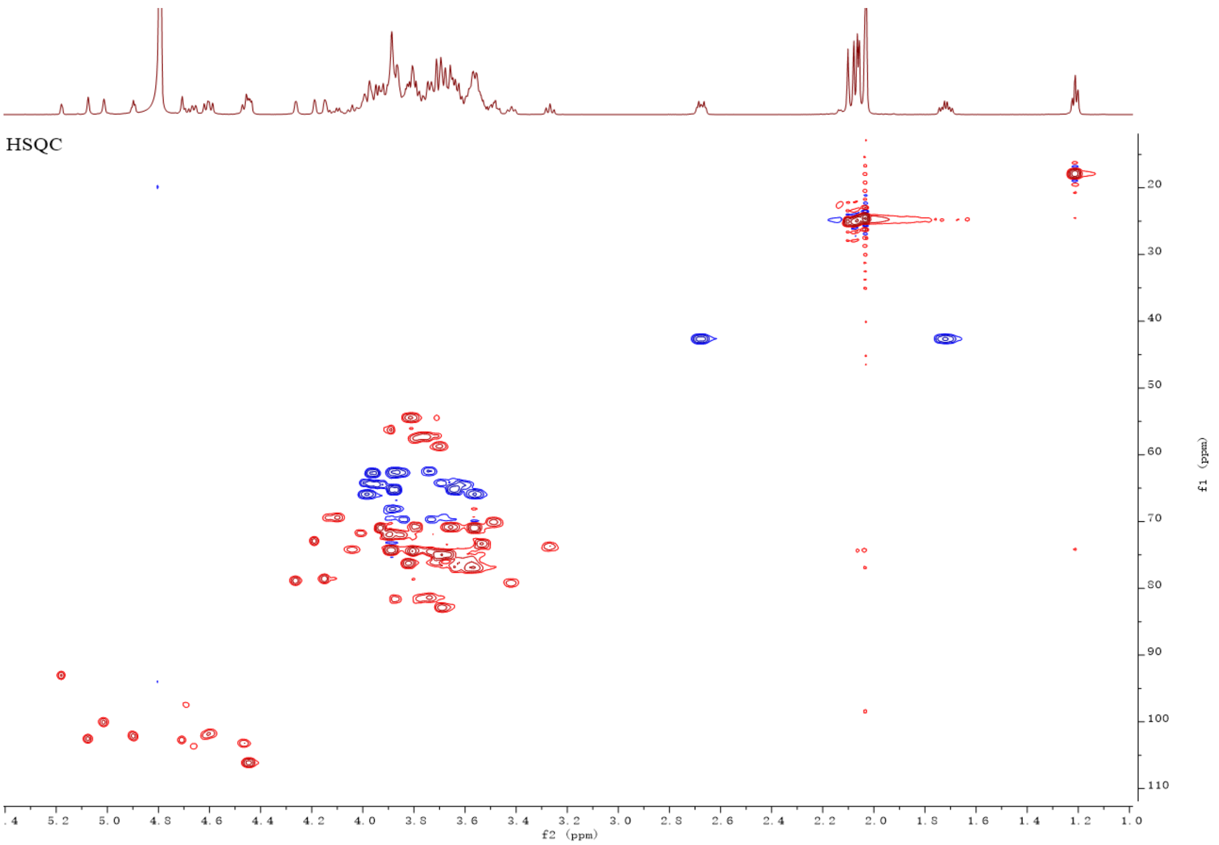
HSQC
f2 (ppm)
f1 (ppm)

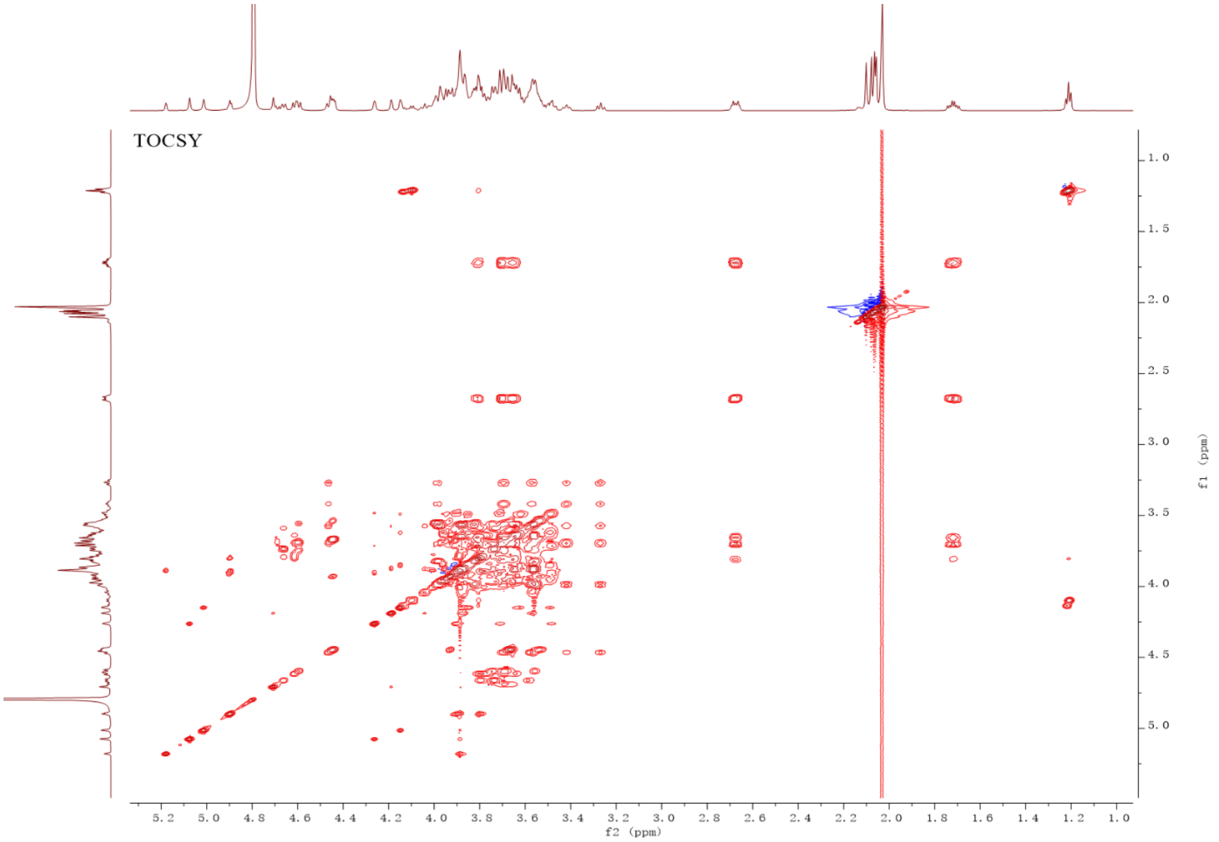
TOCSY
f2 (ppm)
f1 (ppm)

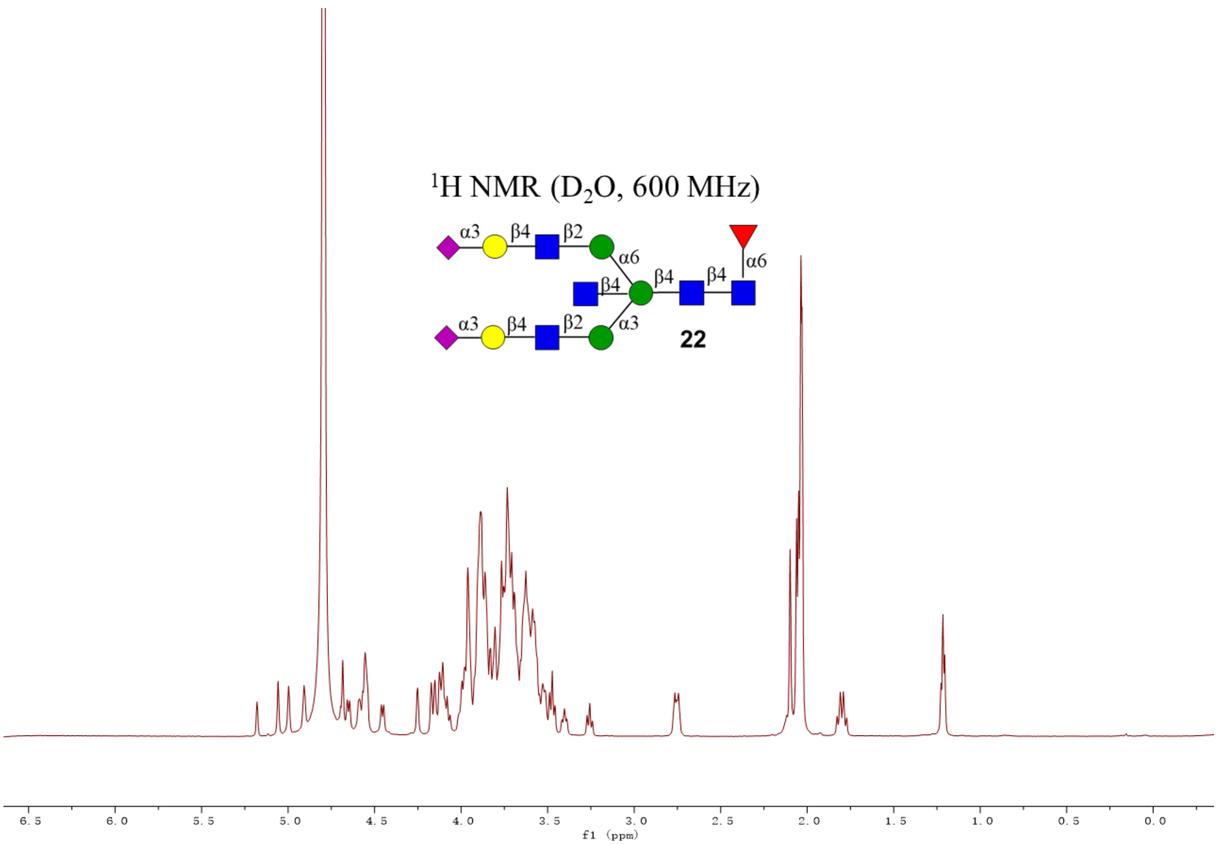

1H NMR (D2O, 600 MHz)
α3
β4
β2
α6
β4
β4
β4
α6
α3
β4
β2
α3
22
f1 (ppm)

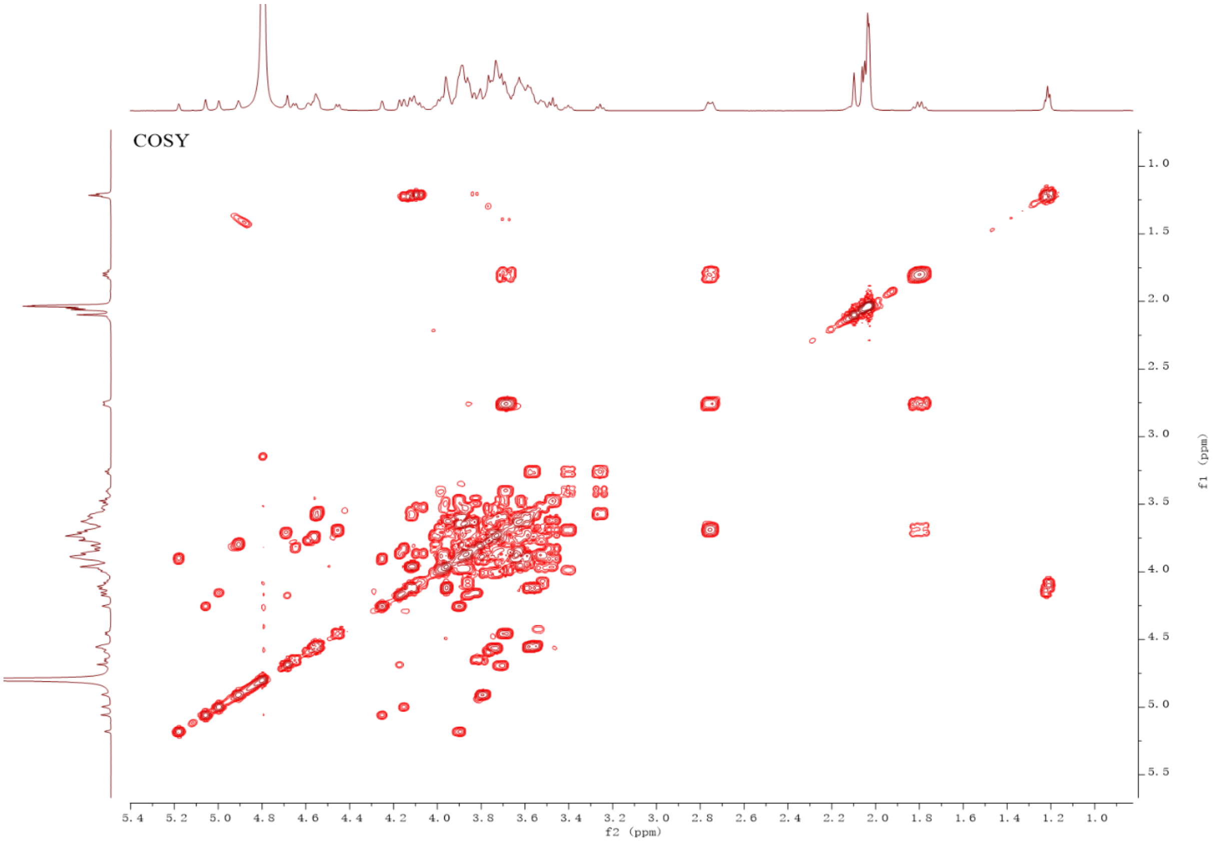

COSY
f2 (ppm)
f1 (ppm)

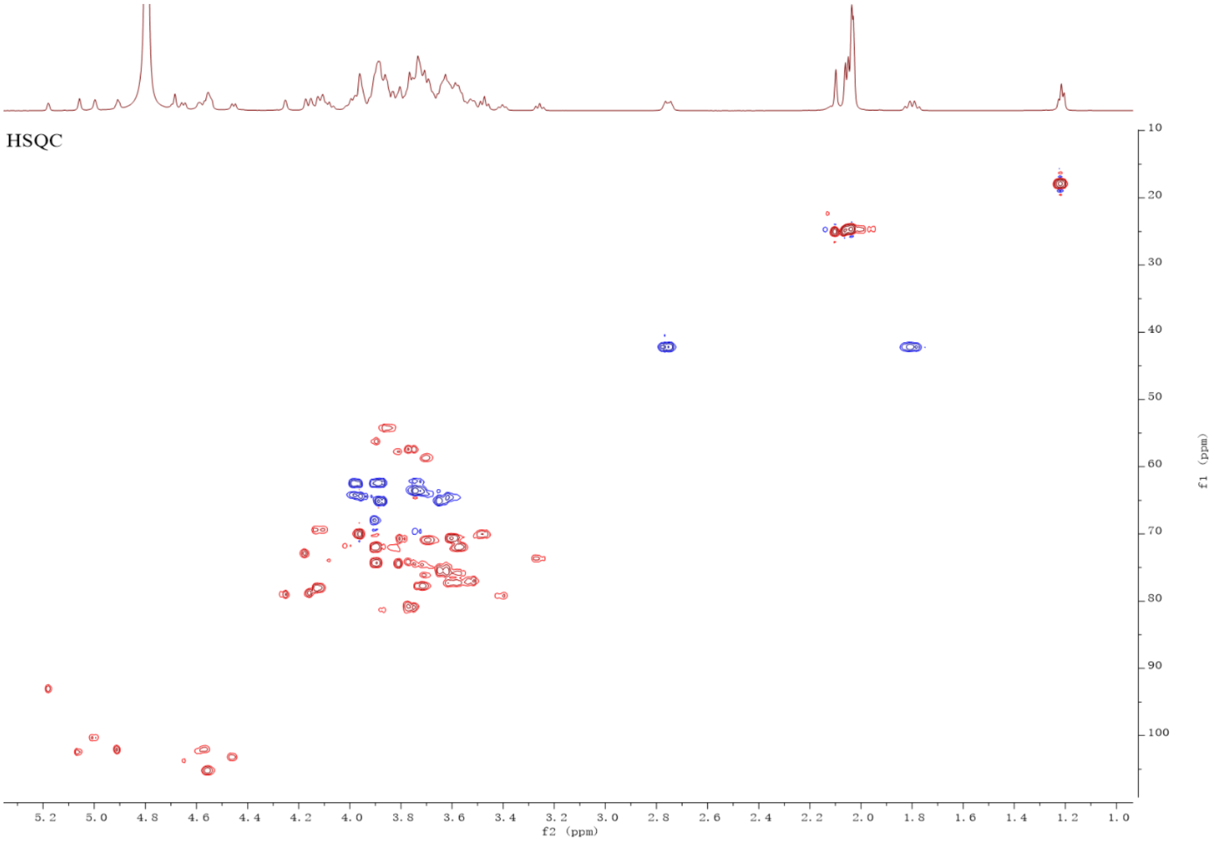
HSQC
f2 (ppm)
f1 (ppm)

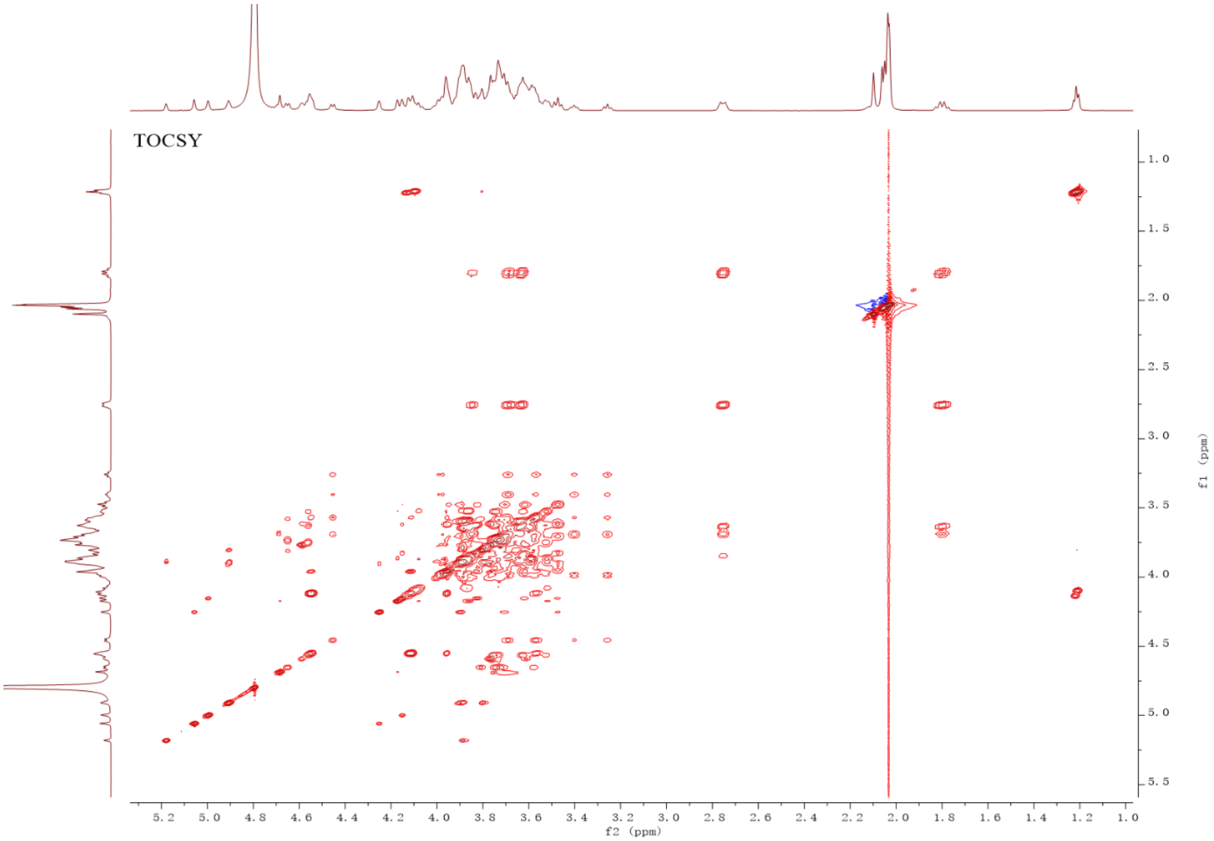
TOCSY
f2 (ppm)
f1 (ppm)

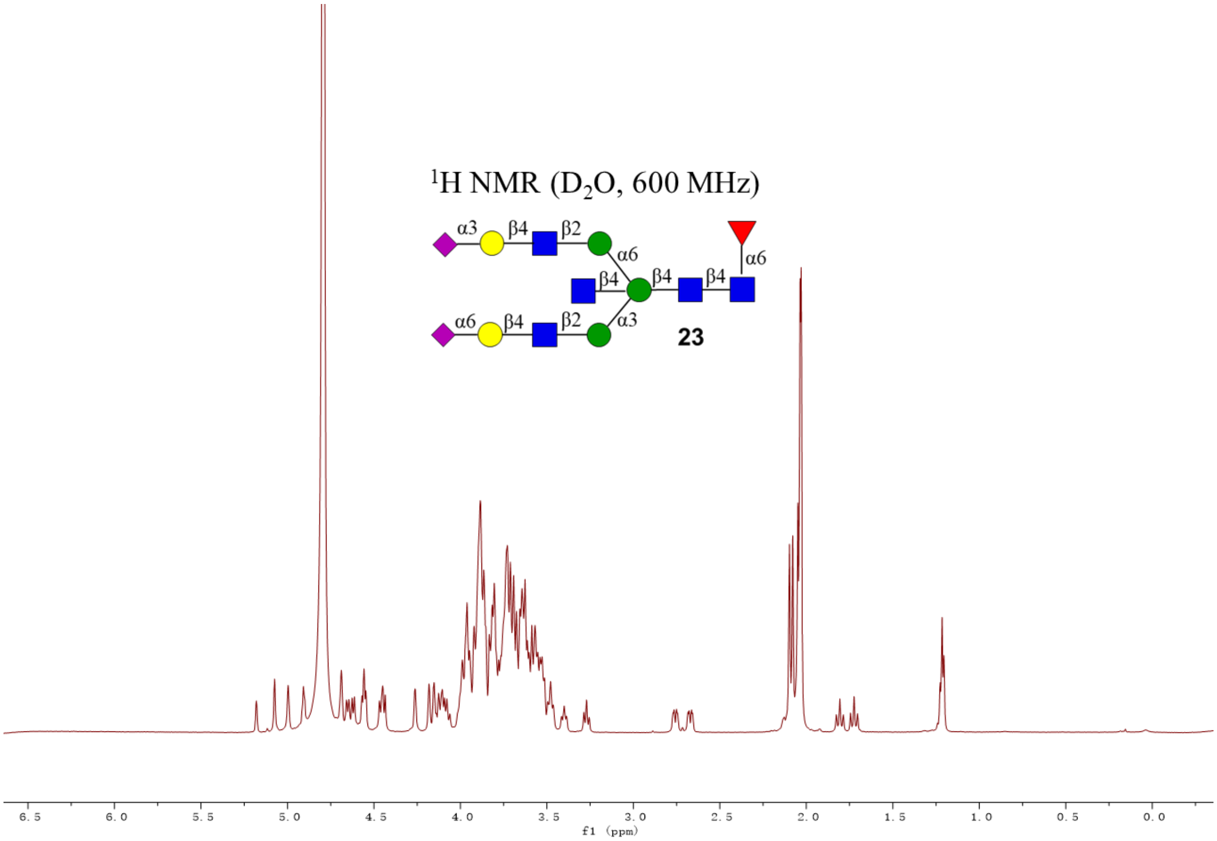

^{1}H NMR (D_2O, 600 MHz)
α3
β4
β2
α6
β4
β4
β4
α6
α6
β4
β2
α3
23
f1 (ppm)

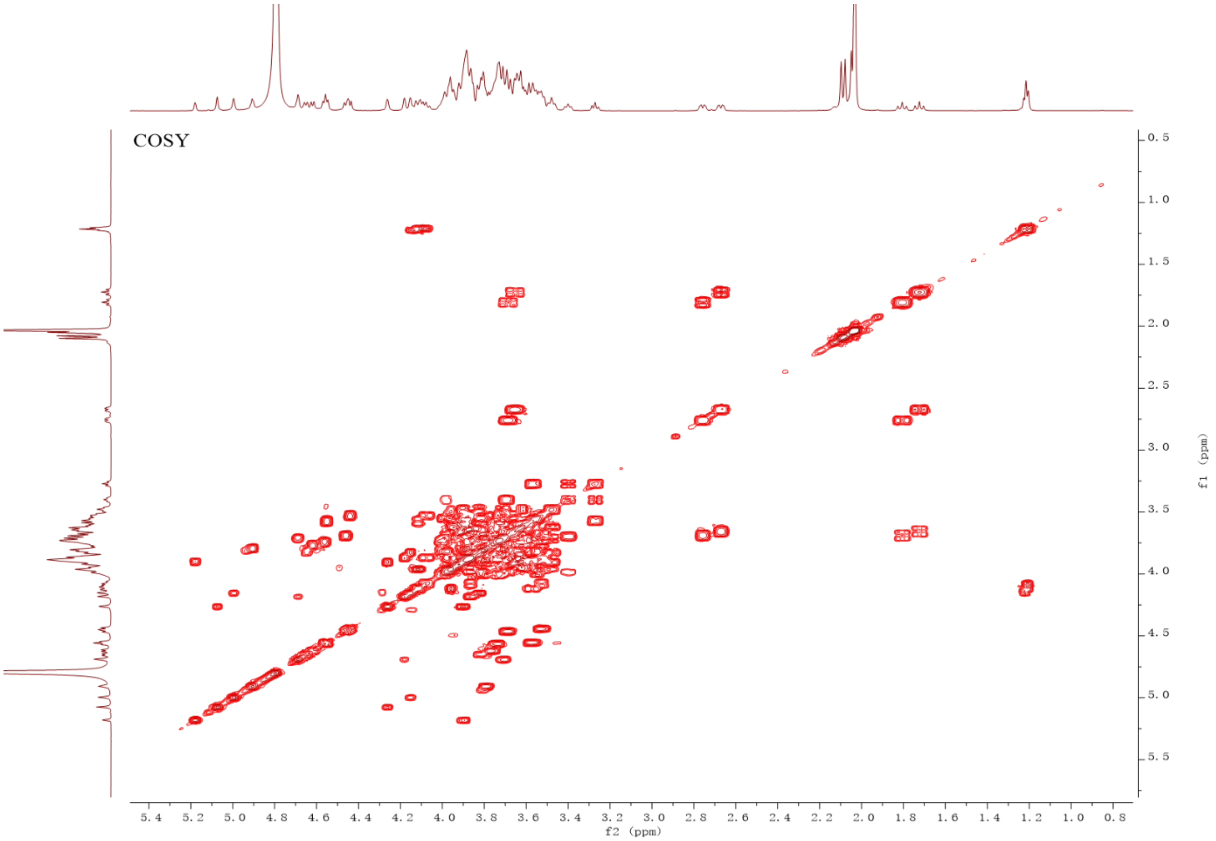

COSY
f2 (ppm)
f1 (ppm)

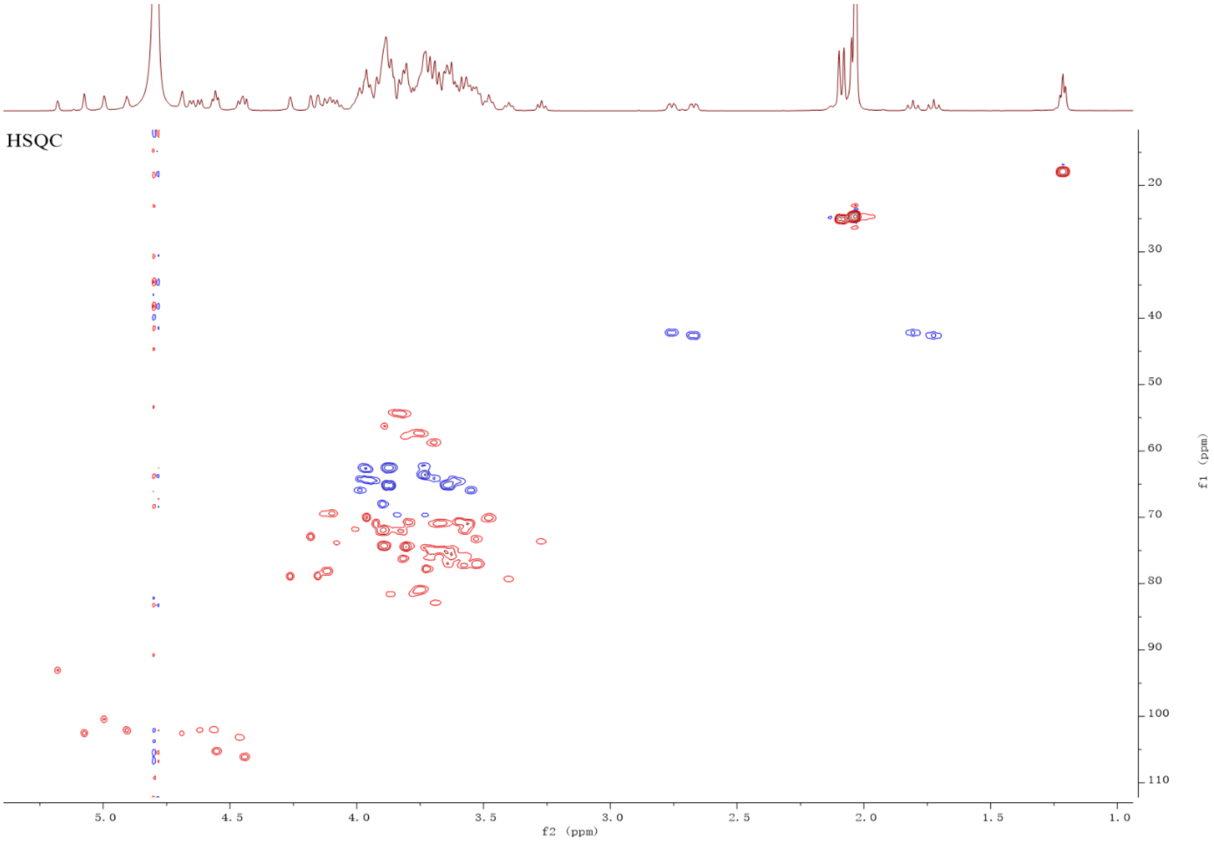
HSQC
f2 (ppm)
f1 (ppm)

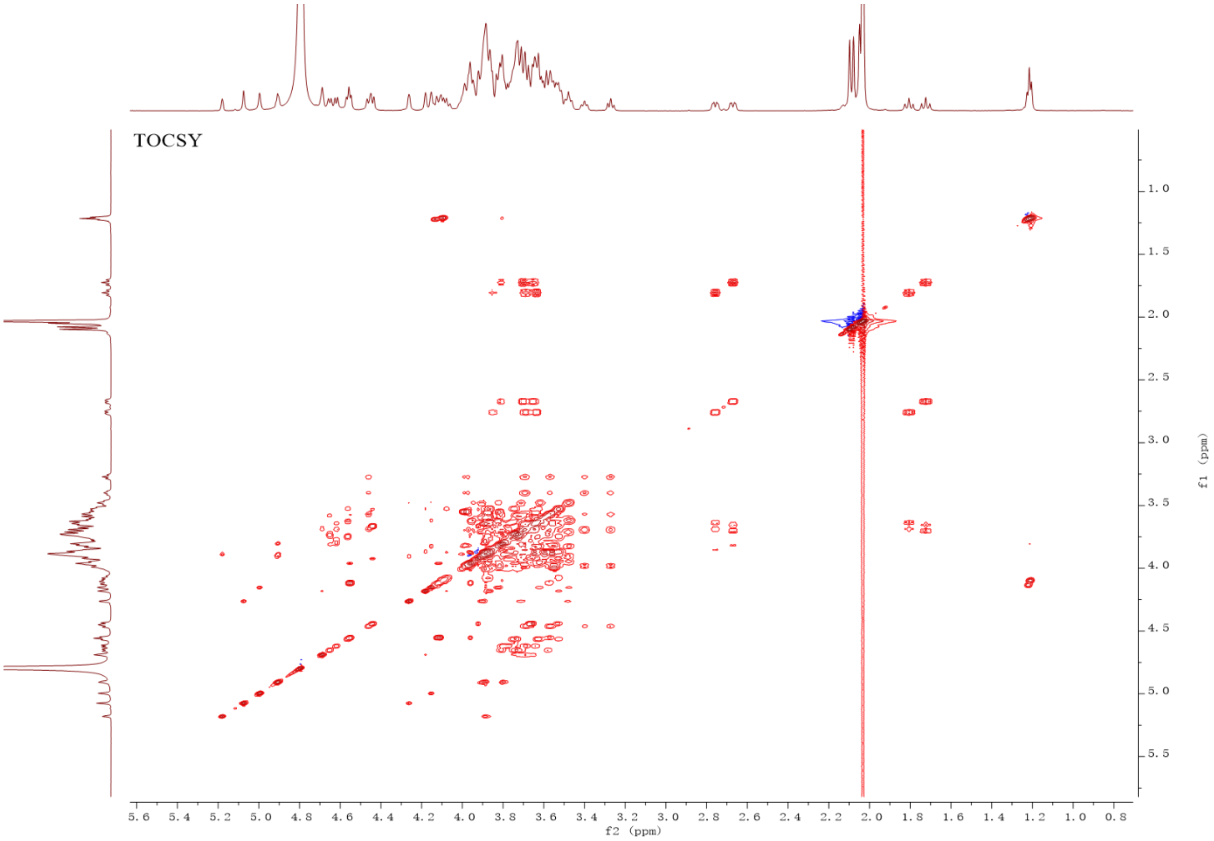
TOCSY
f2 (ppm)
f1 (ppm)

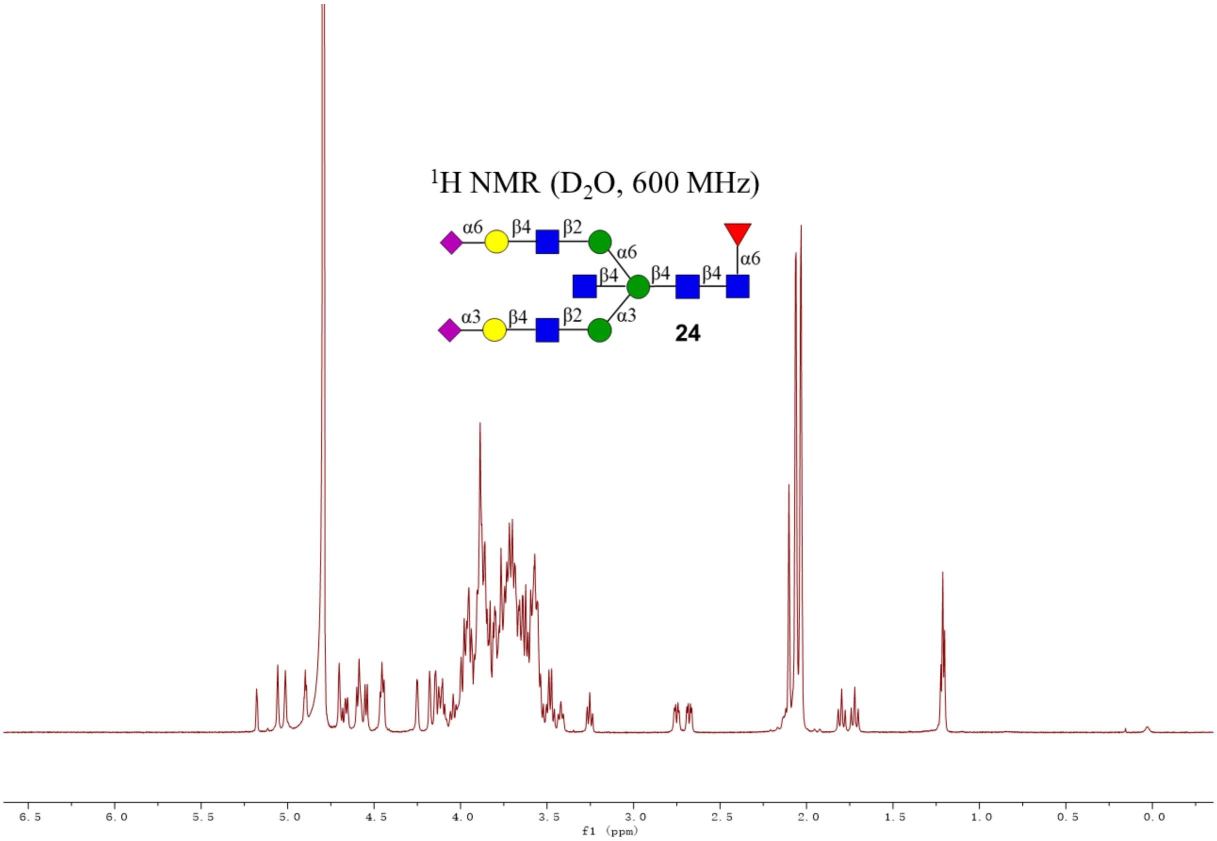

^{1}H NMR (D_2O, 600 MHz)
α6
β4
β2
α6
β4
β4
β4
α6
α3
β4
β2
α3
24
f1 (ppm)

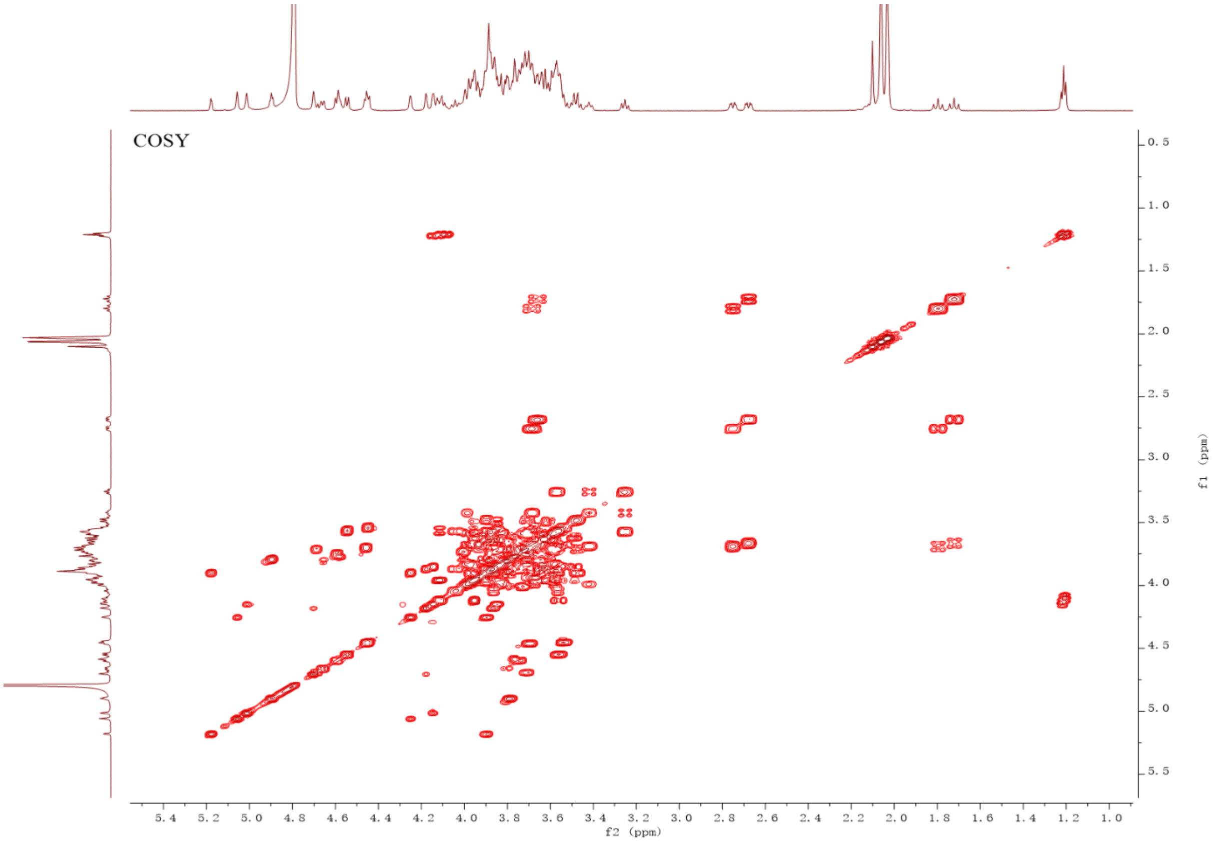

COSY
f2 (ppm)
f1 (ppm)

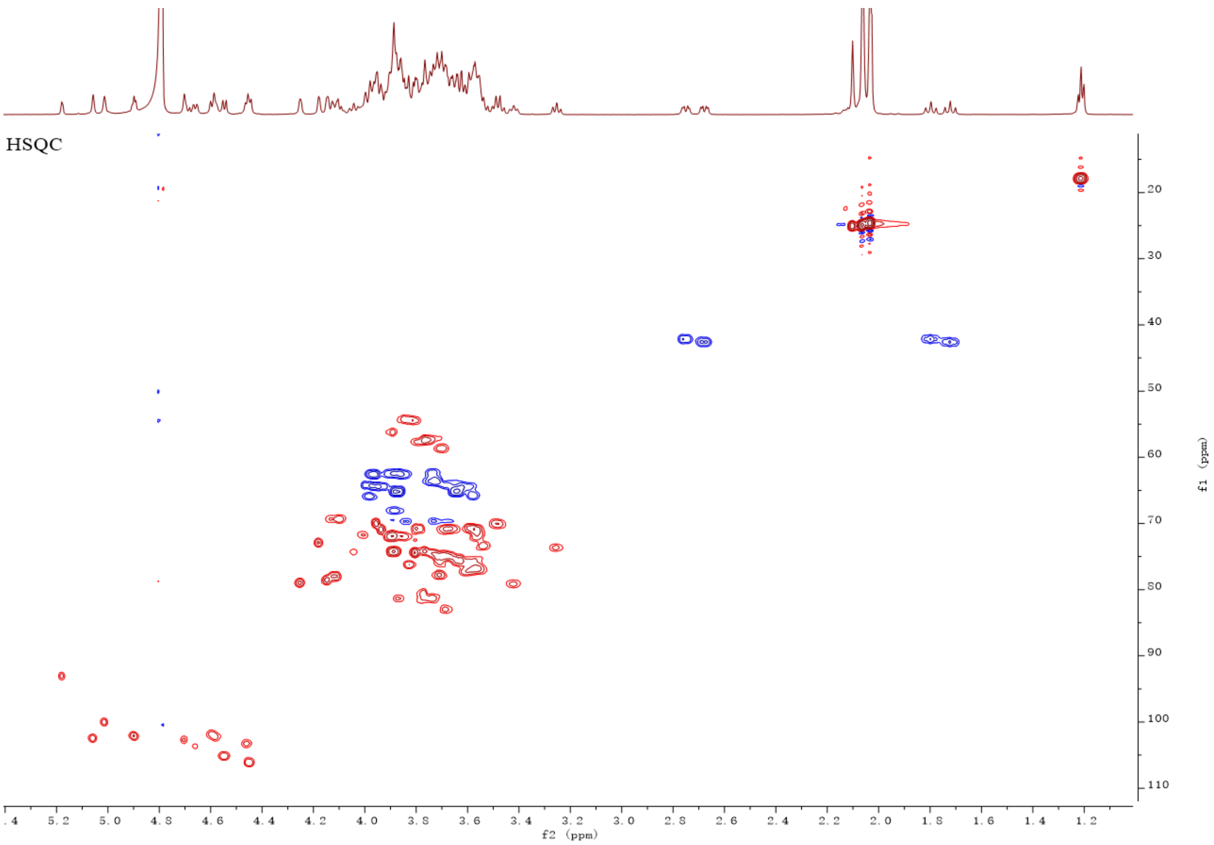
HSQC
f2 (ppm)
f1 (ppm)

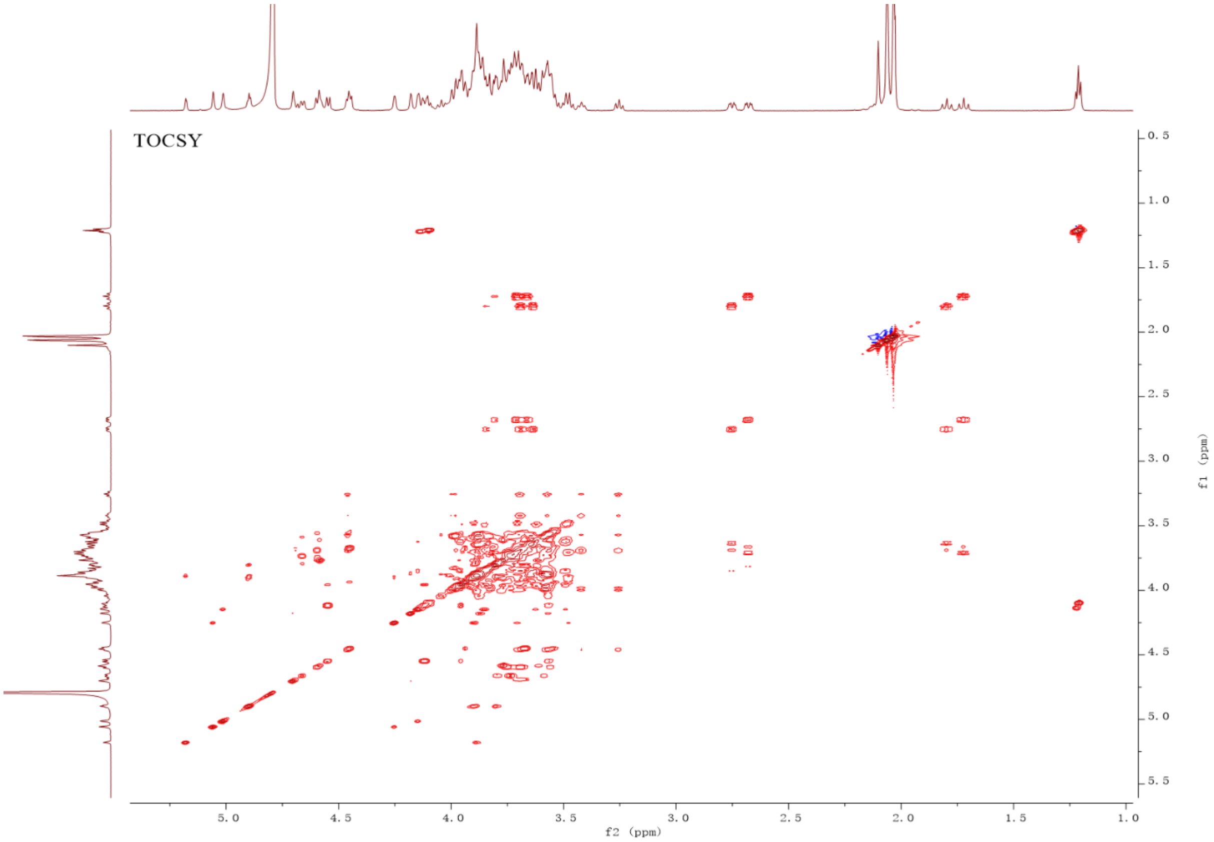
TOCSY
f2 (ppm)
f1 (ppm)

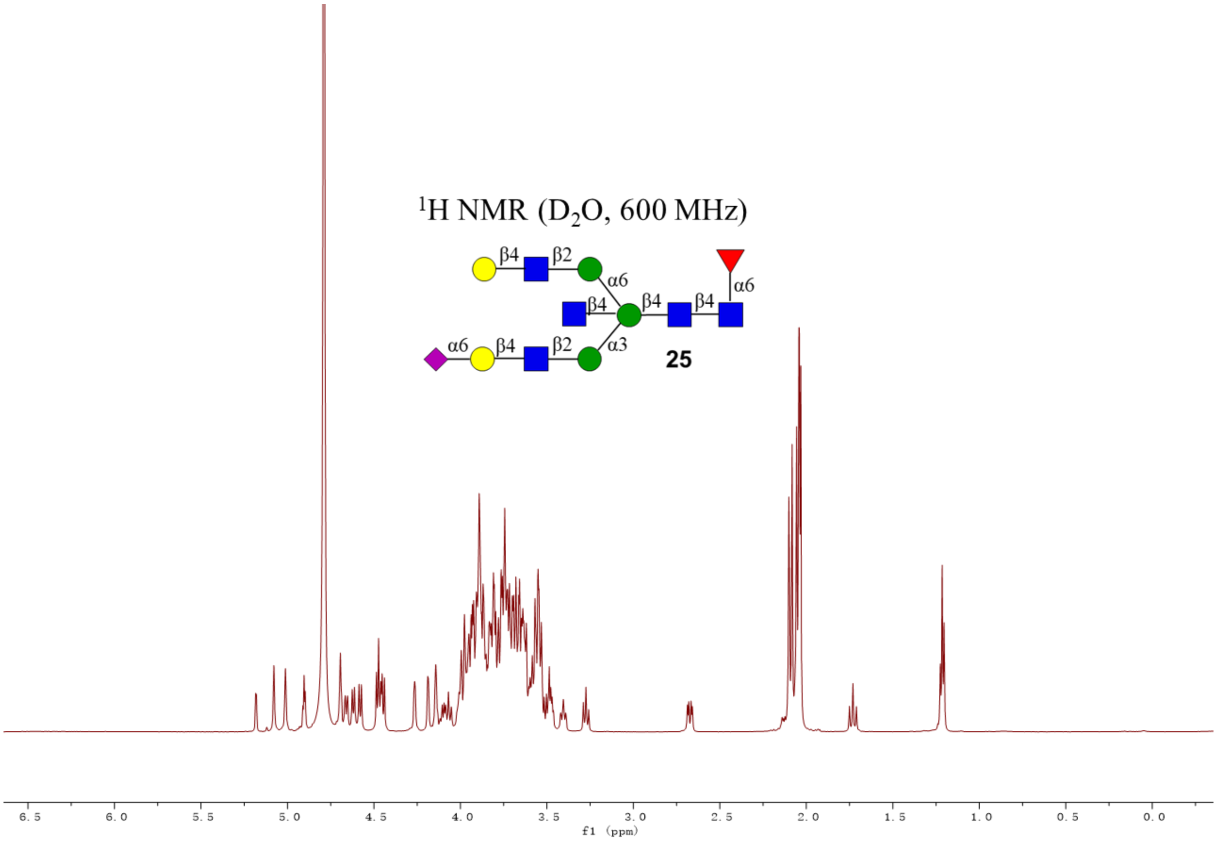

^1H NMR (D_2O, 600 MHz)
β4
β2
α6
β4
β4
β4
α6
α6
β4
β2
α3
25
f1 (ppm)

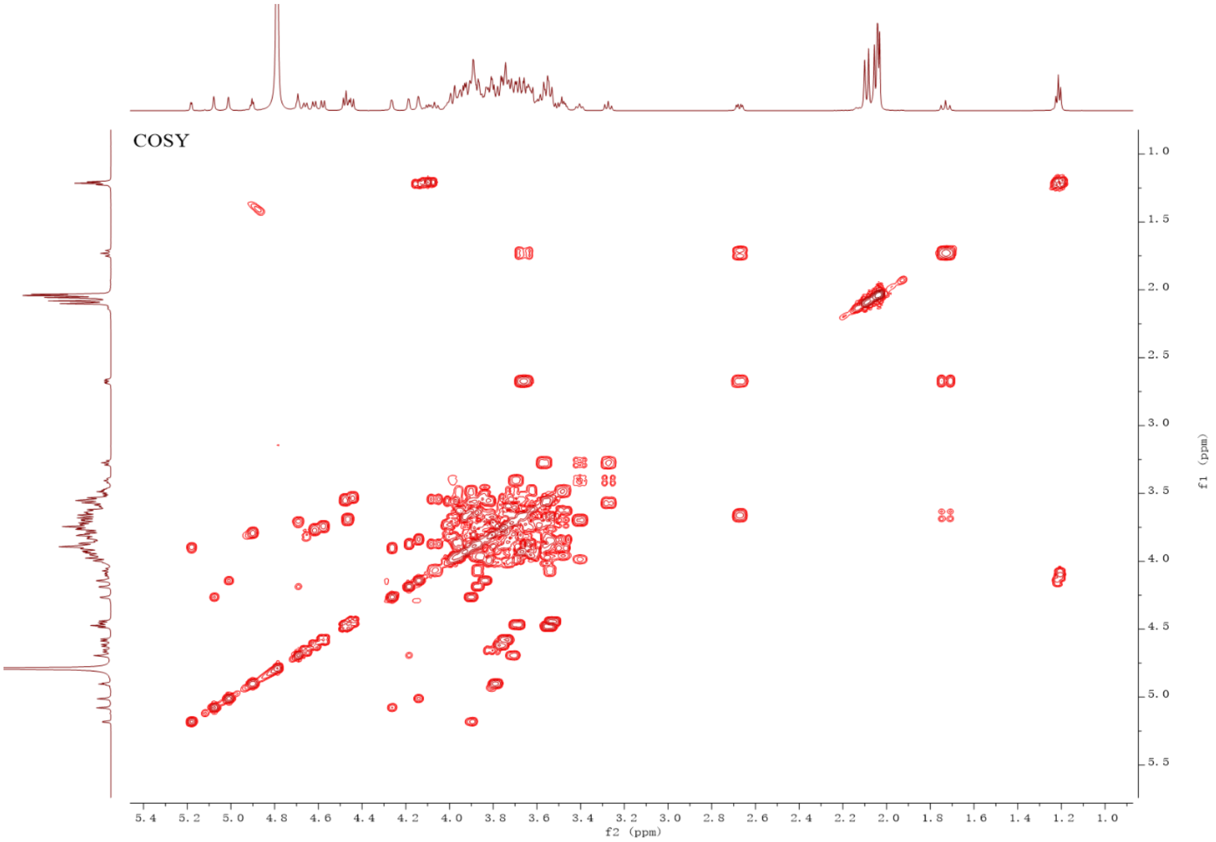

COSY
f2 (ppm)
f1 (ppm)

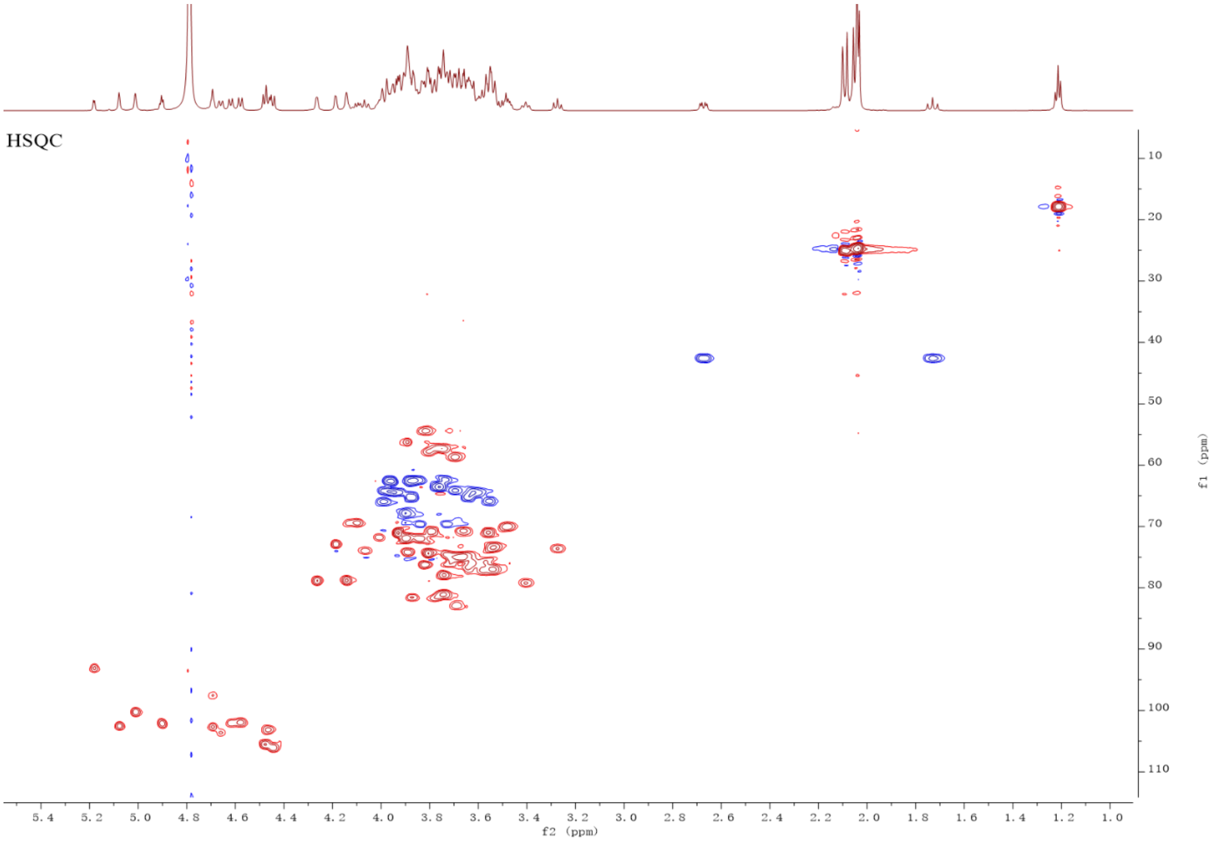
HSQC
f2 (ppm)
f1 (ppm)

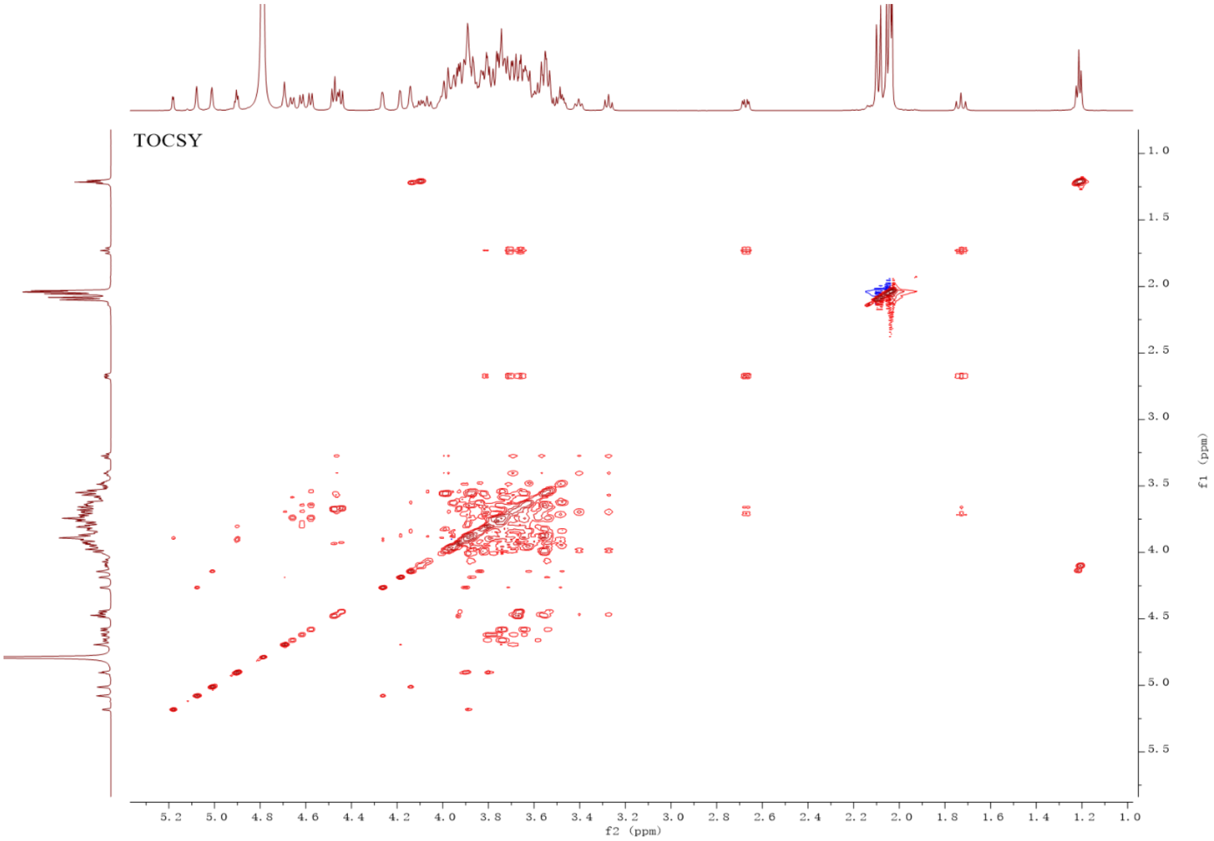
TOCSY
f2 (ppm)
f1 (ppm)

^{1}H NMR (D_2O, 600 MHz)

26

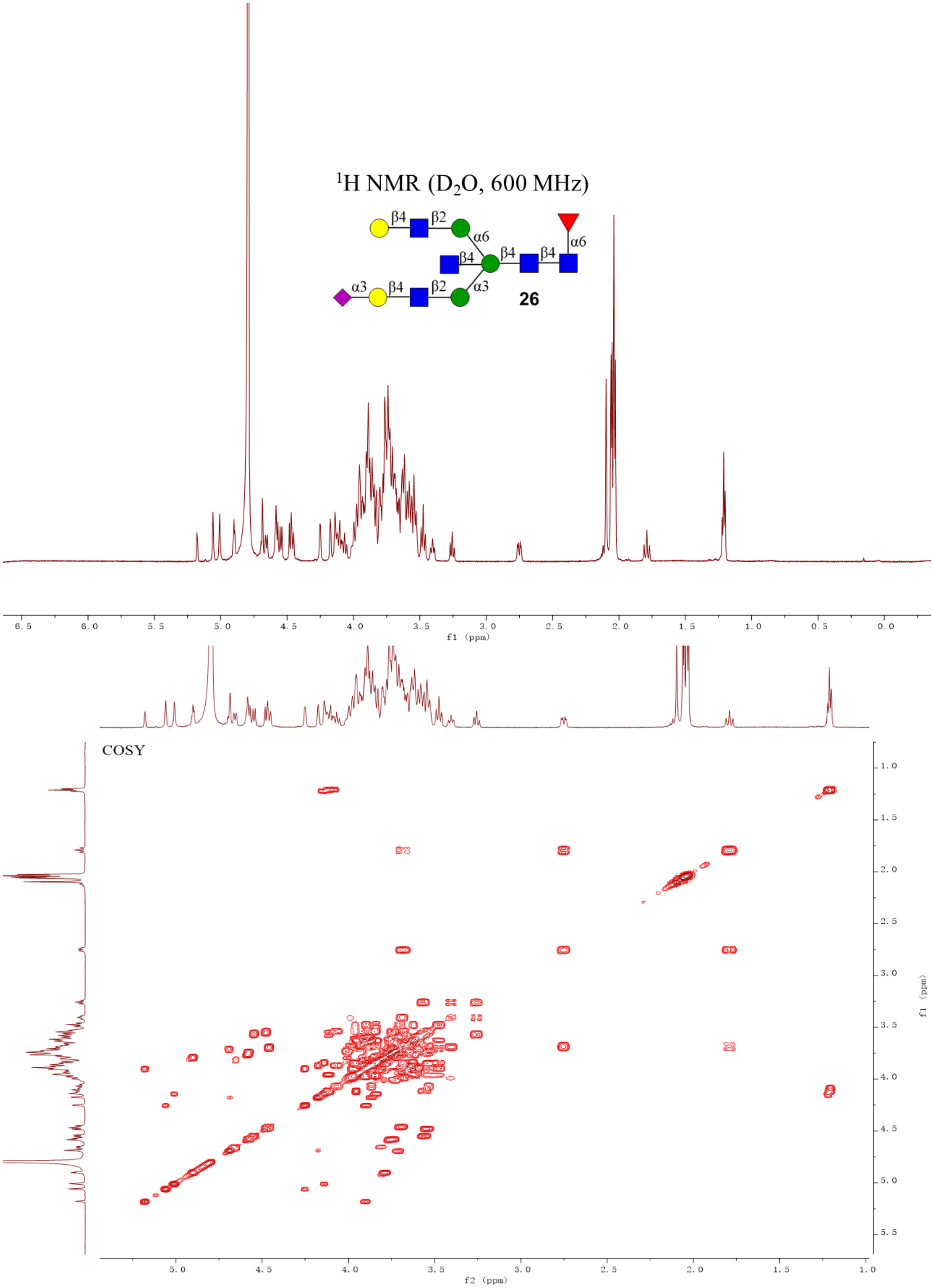

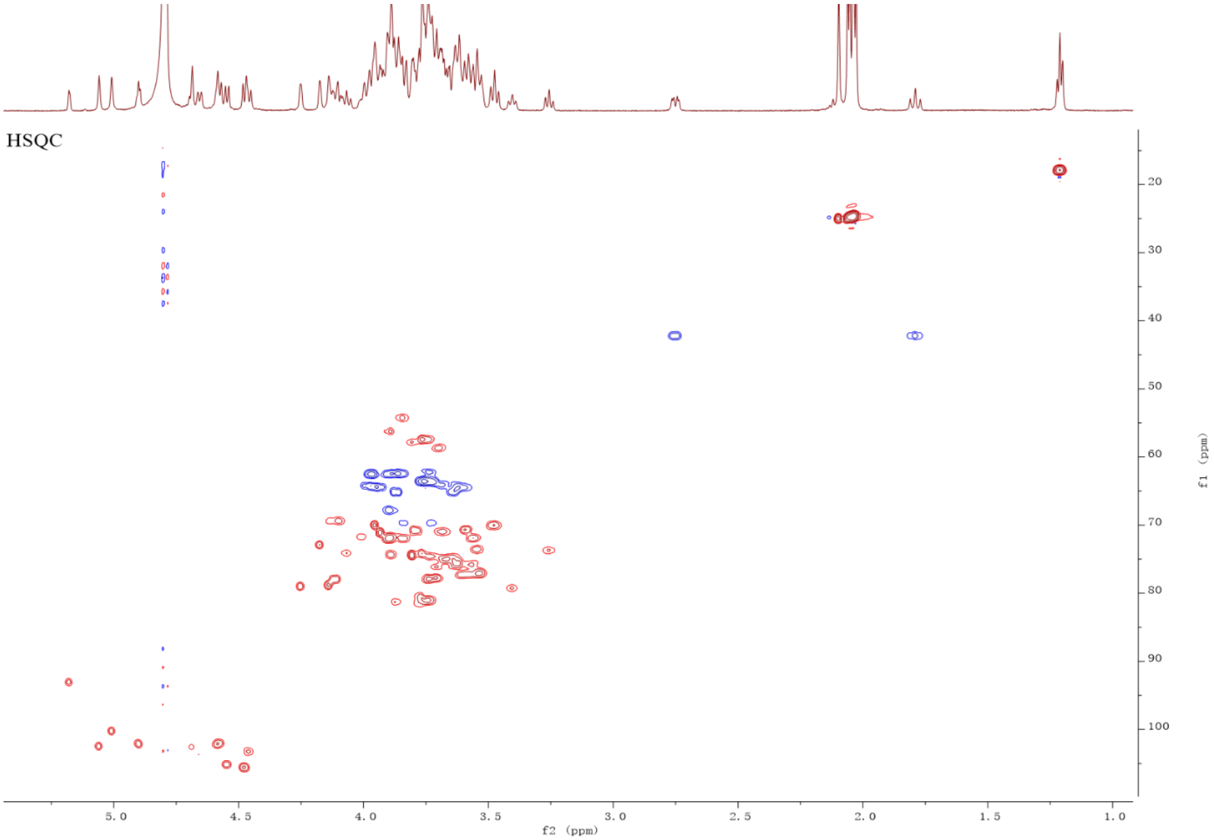
HSQC
f2 (ppm)
f1 (ppm)

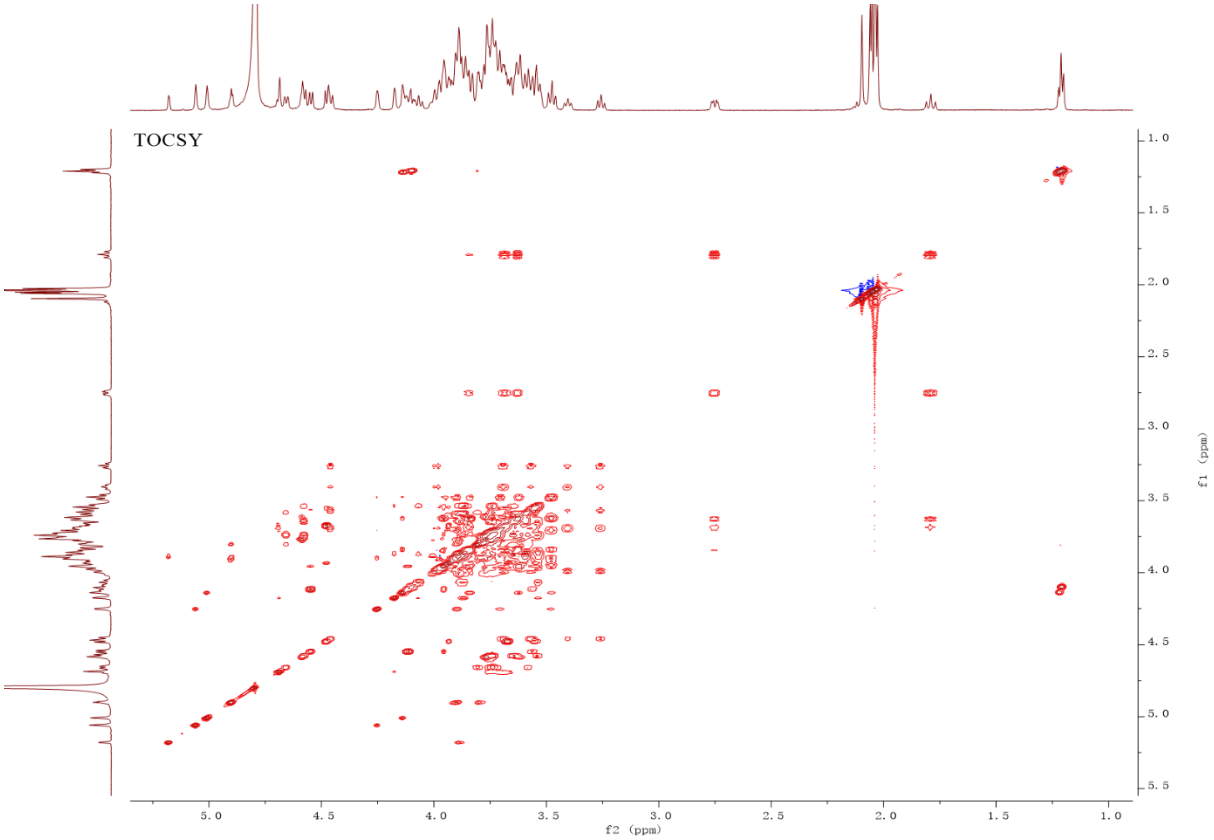
TOCSY
f2 (ppm)
f1 (ppm)

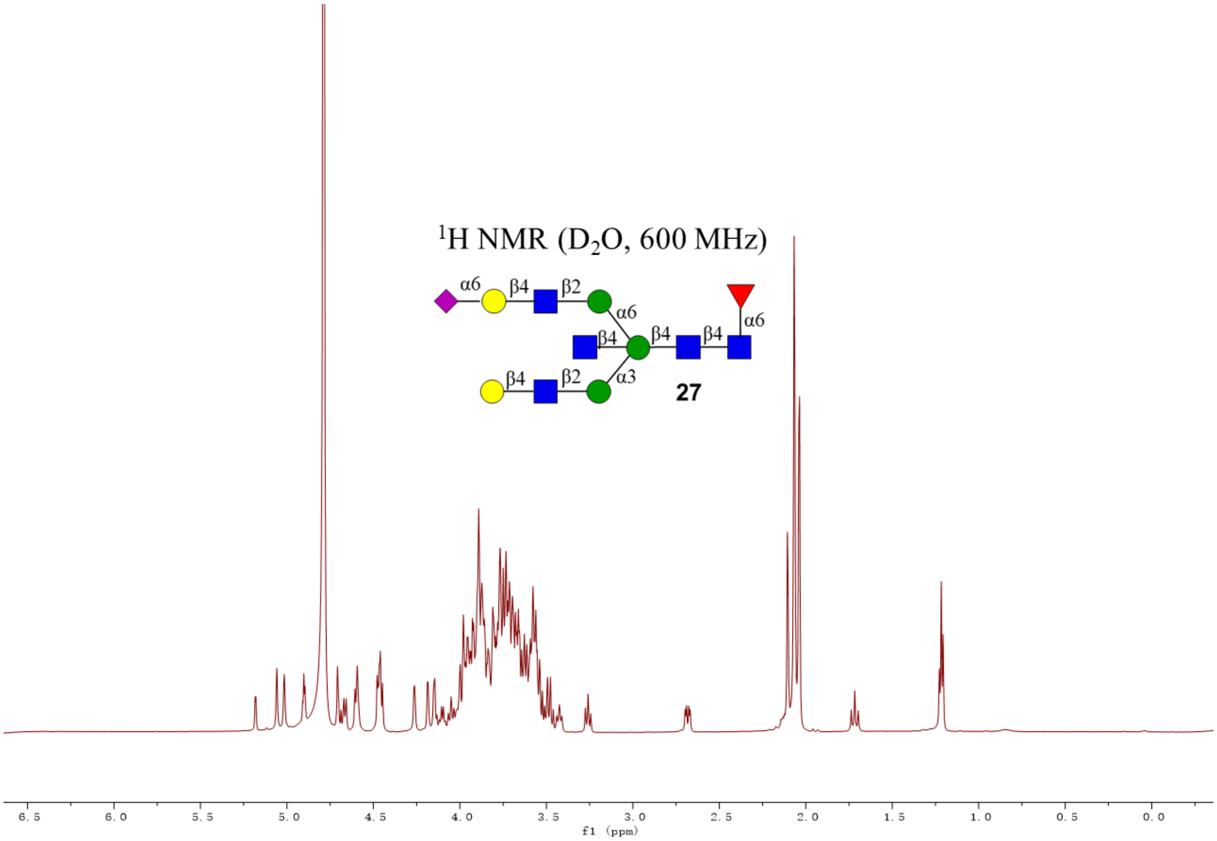
^{1}H NMR (D_2O, 600 MHz)
α6
β4
β2
α6
β4
β4
β4
α6
β4
β2
α3
27
f1 (ppm)

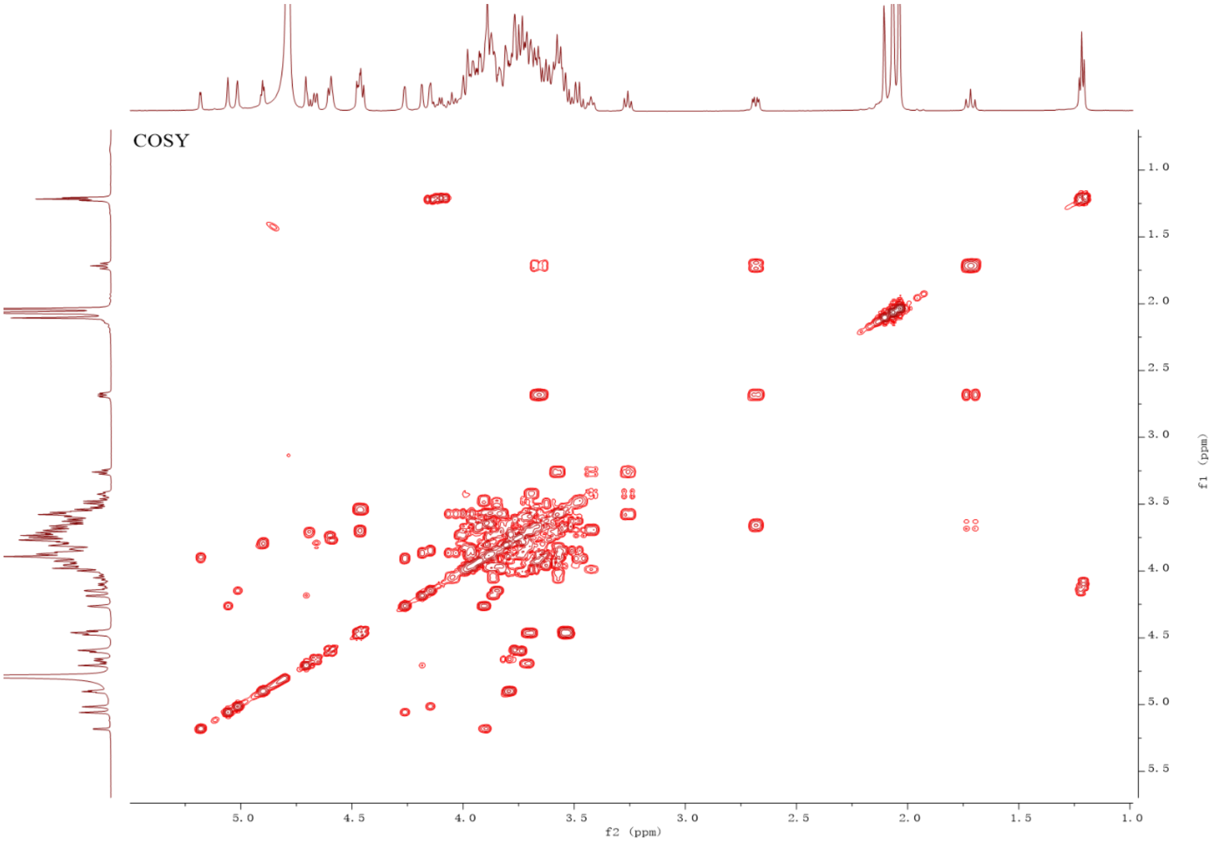
COSY
f2 (ppm)
f1 (ppm)

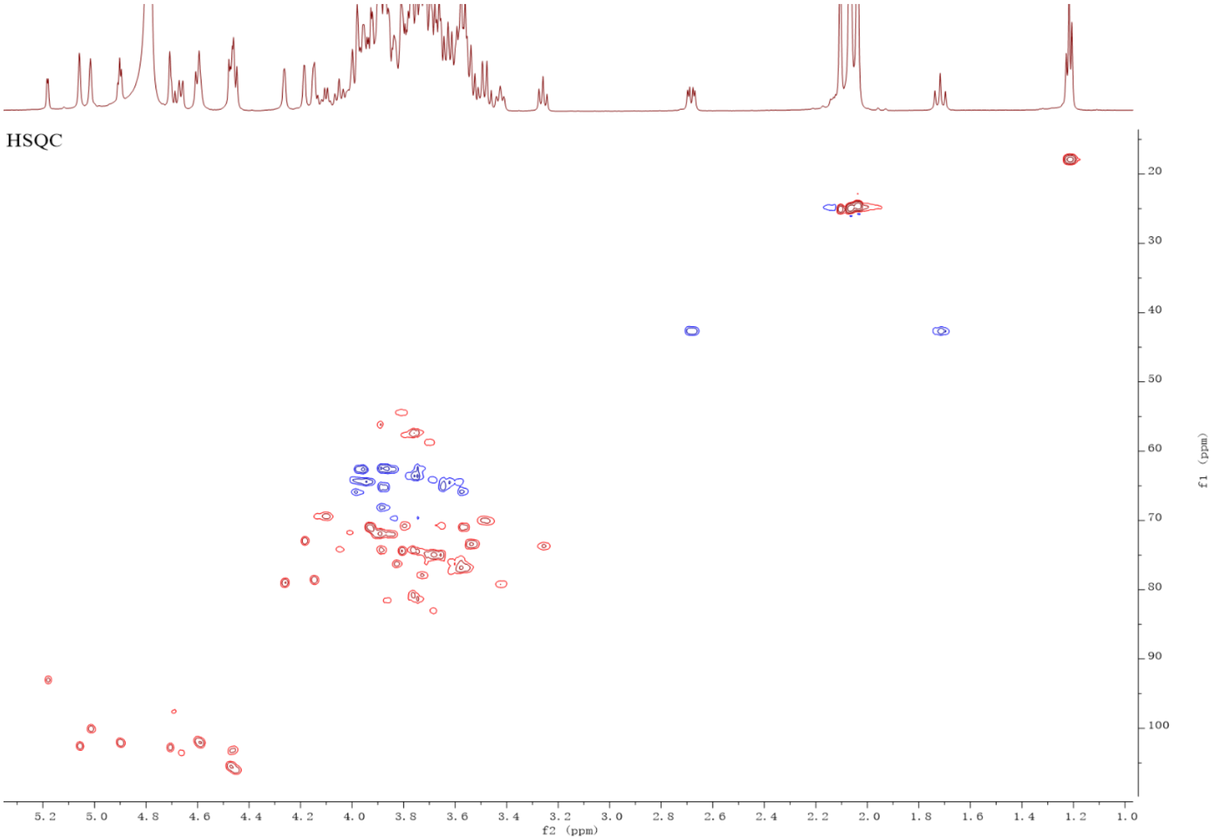
HSQC
f2 (ppm)
f1 (ppm)

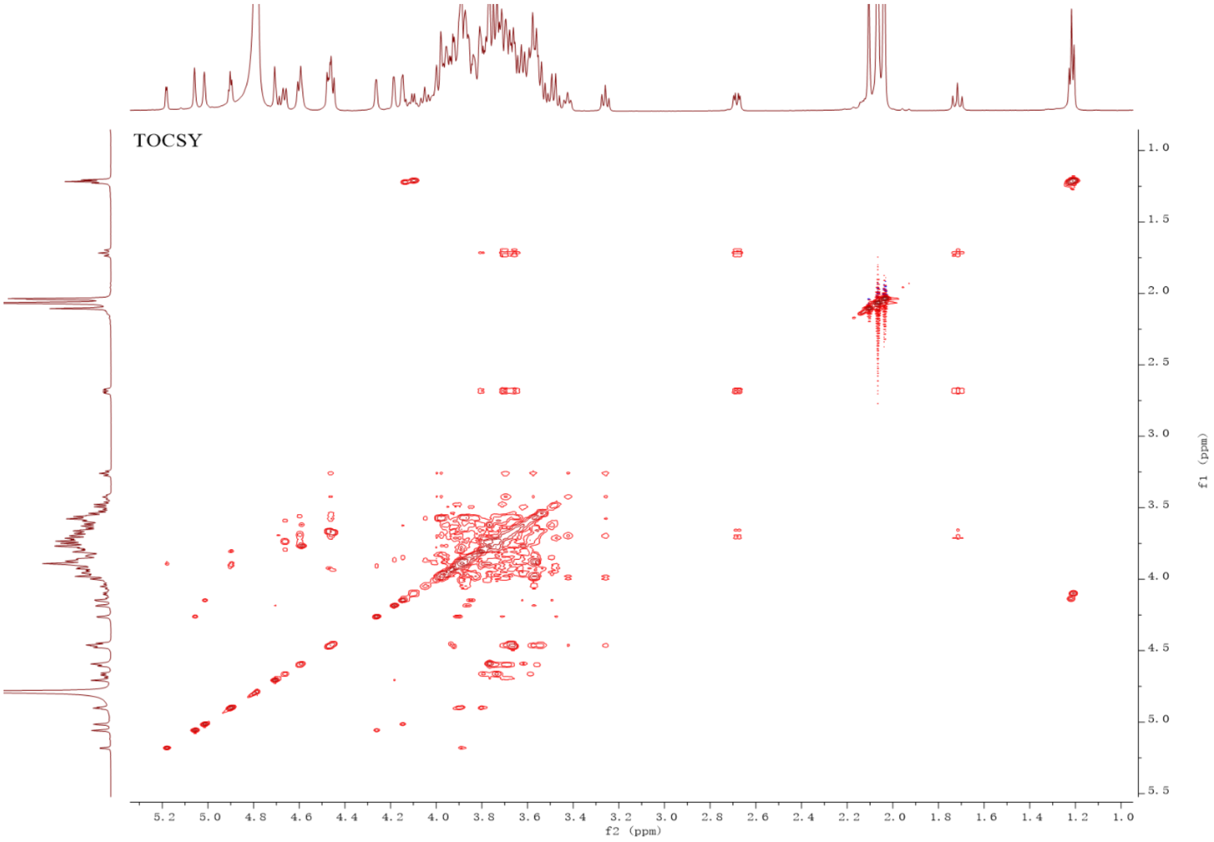
TOCSY
f2 (ppm)
f1 (ppm)

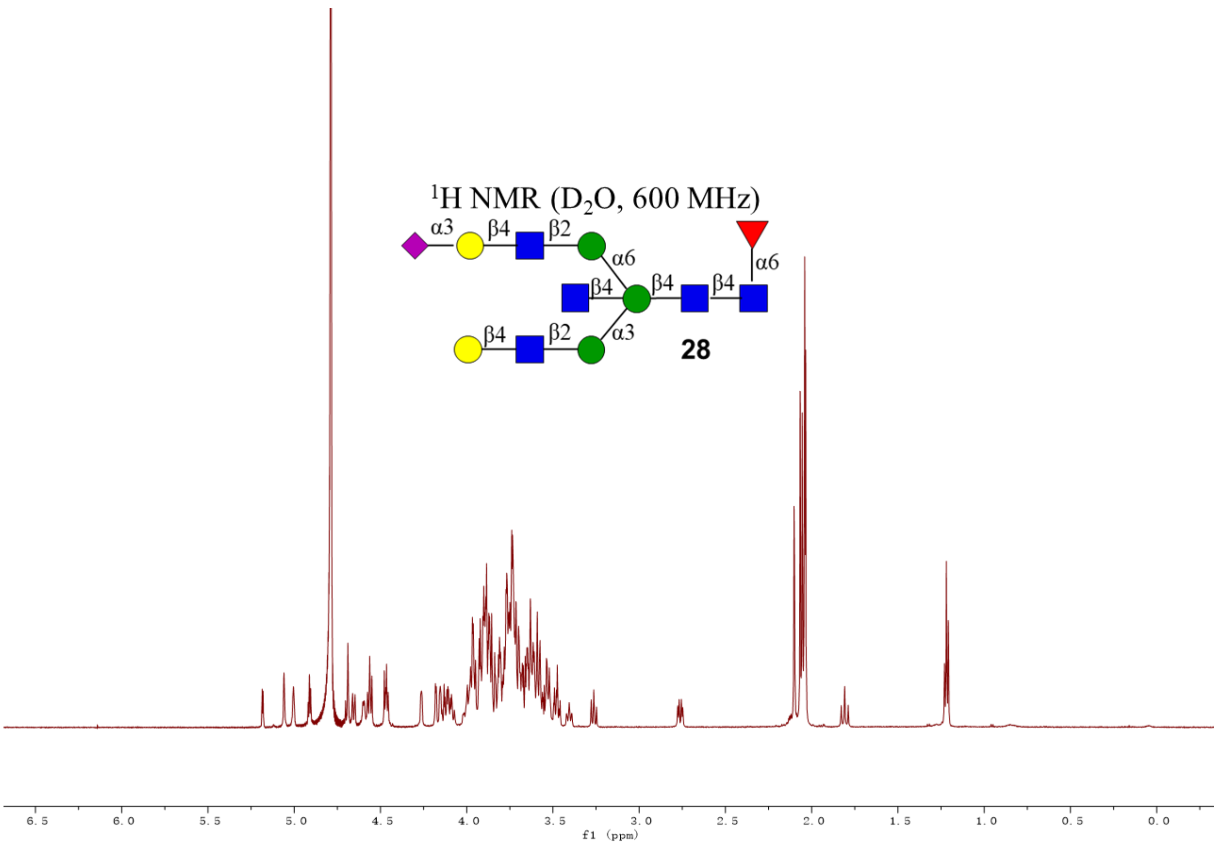

^{1}H NMR (D_2O, 600 MHz)
α3
β4
β2
α6
β4
β4
β4
α6
α3
β4
β2
28
f1 (ppm)

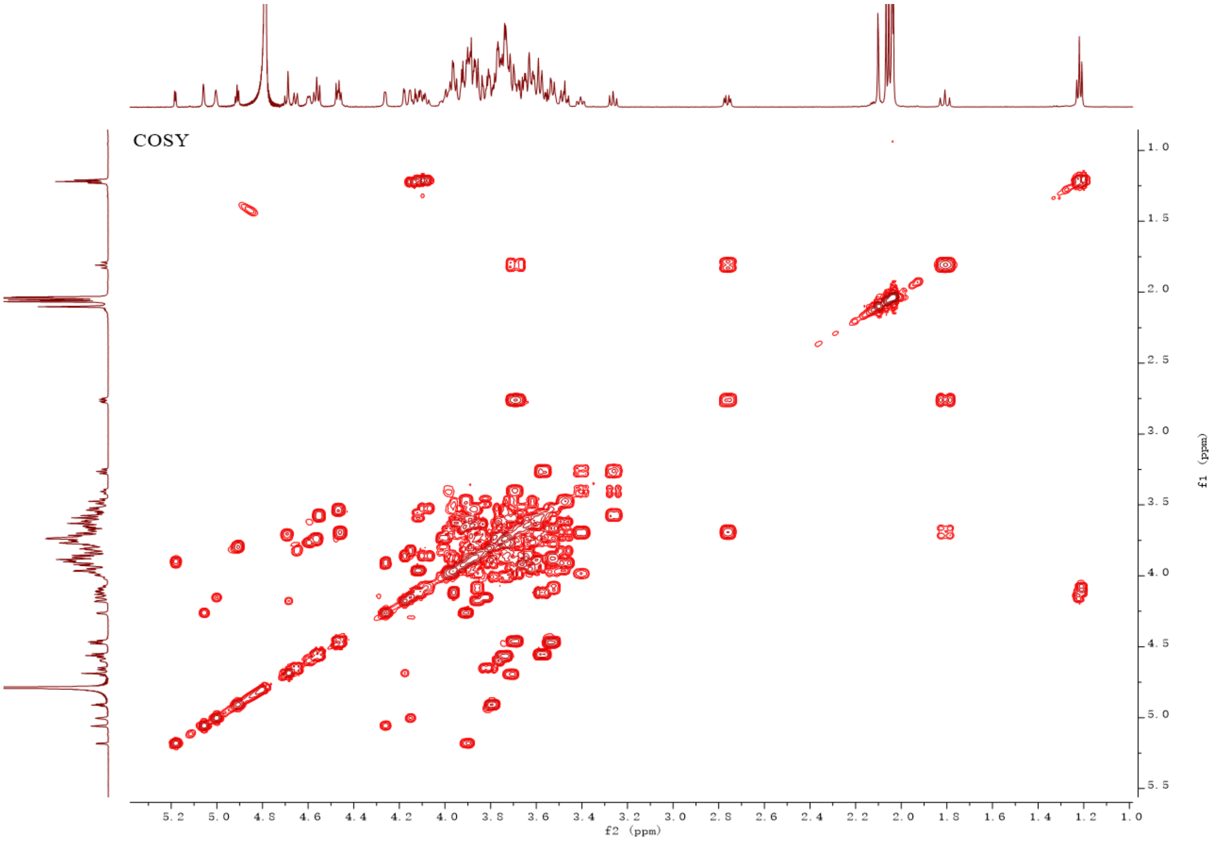

COSY
f2 (ppm)
f1 (ppm)

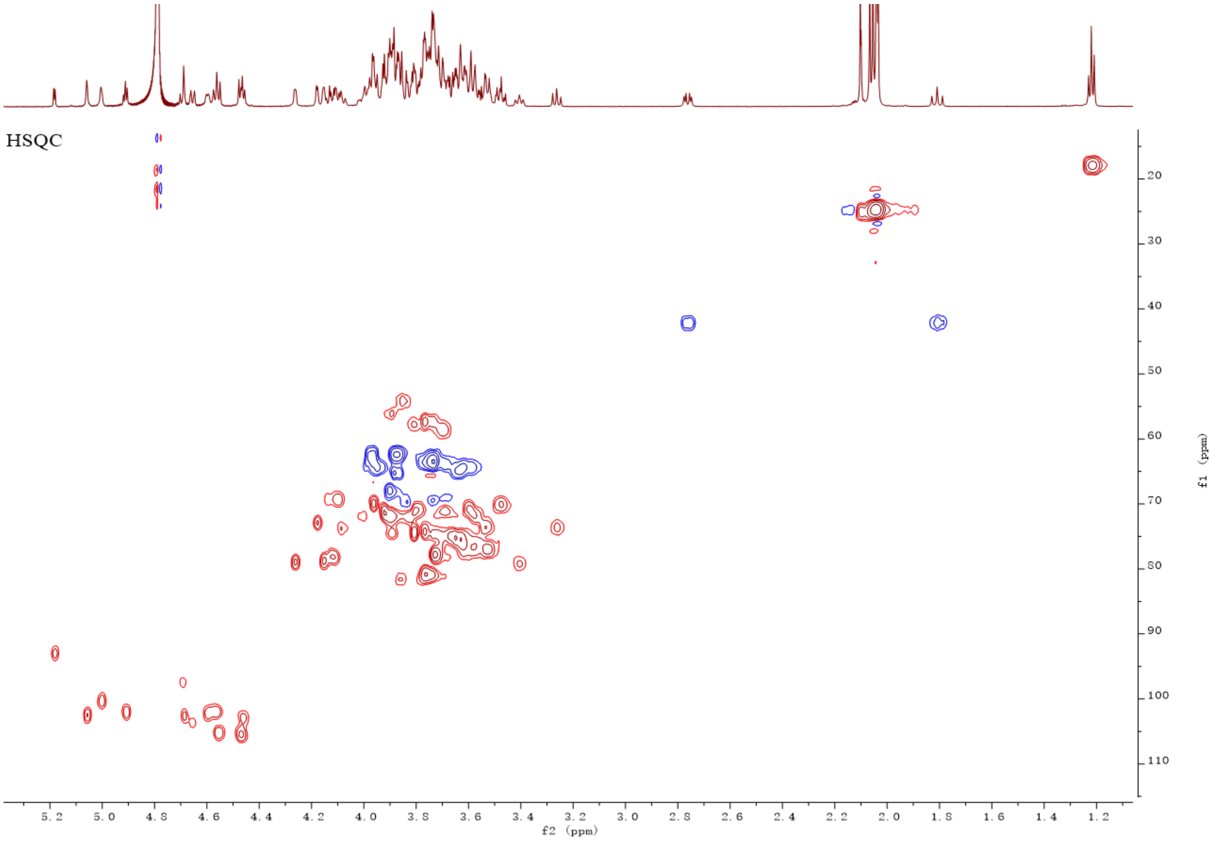
HSQC
f1 (ppm)
f2 (ppm)

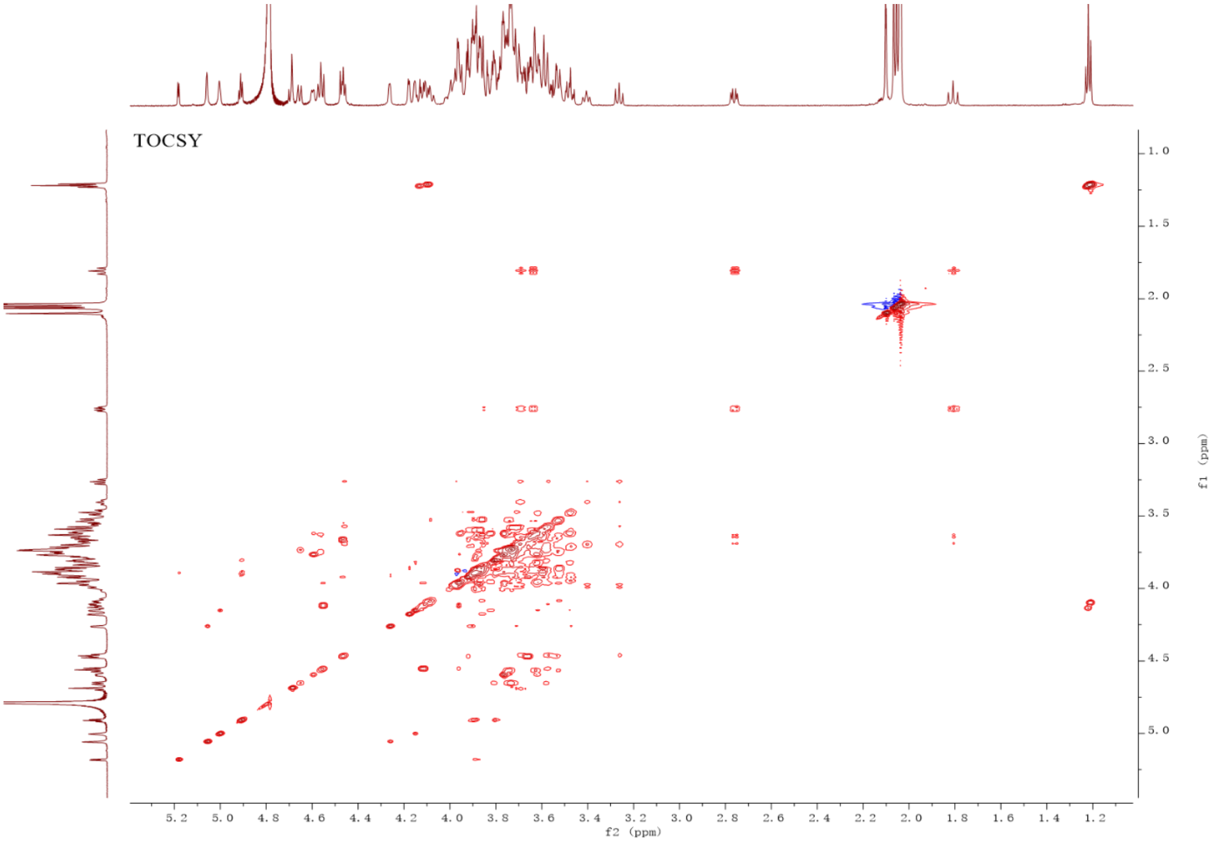
TOCSY
f1 (ppm)
f2 (ppm)

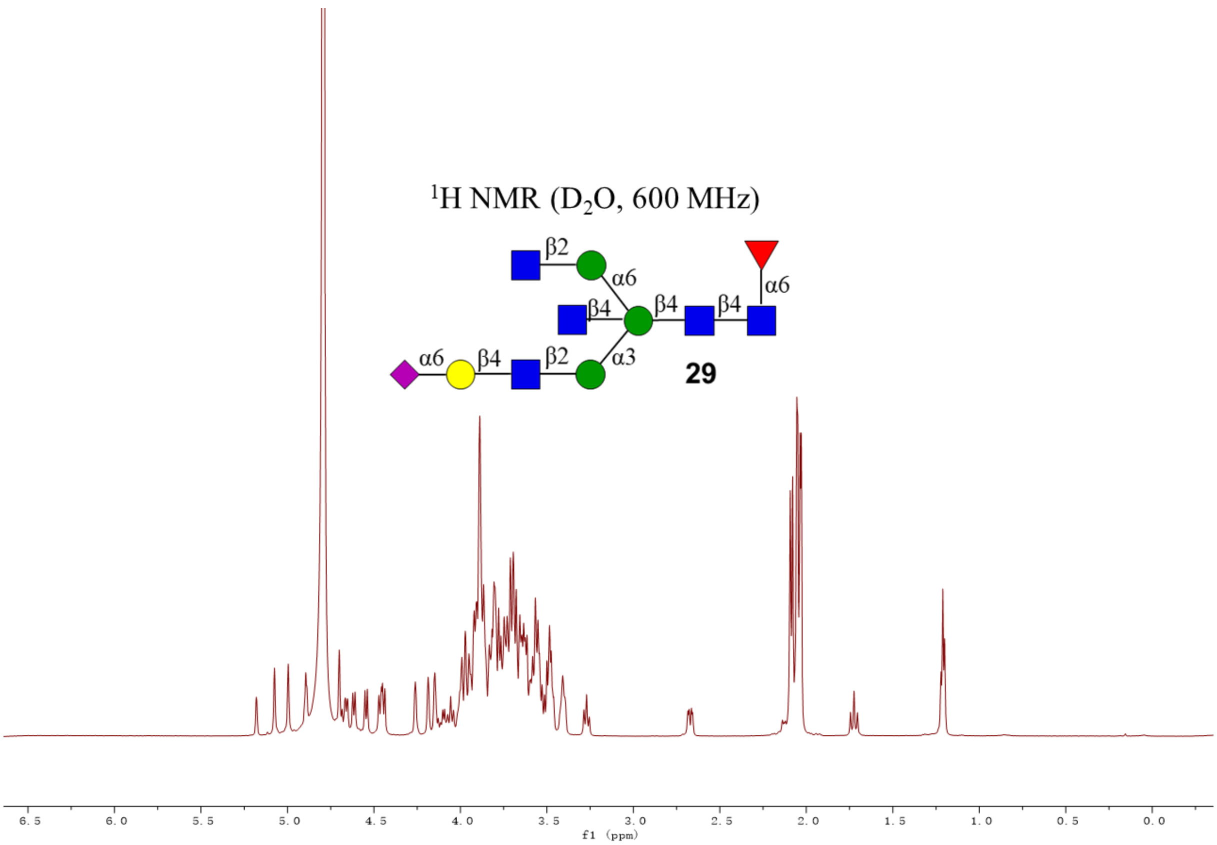

^{1}H NMR (D_2O, 600 MHz)
β2
α6
β4
β4
β4
α6
α6
β4
β2
α3
29
6.5 6.0 5.5 5.0 4.5 4.0 3.5 3.0 2.5 2.0 1.5 1.0 0.5 0.0
f1 (ppm)

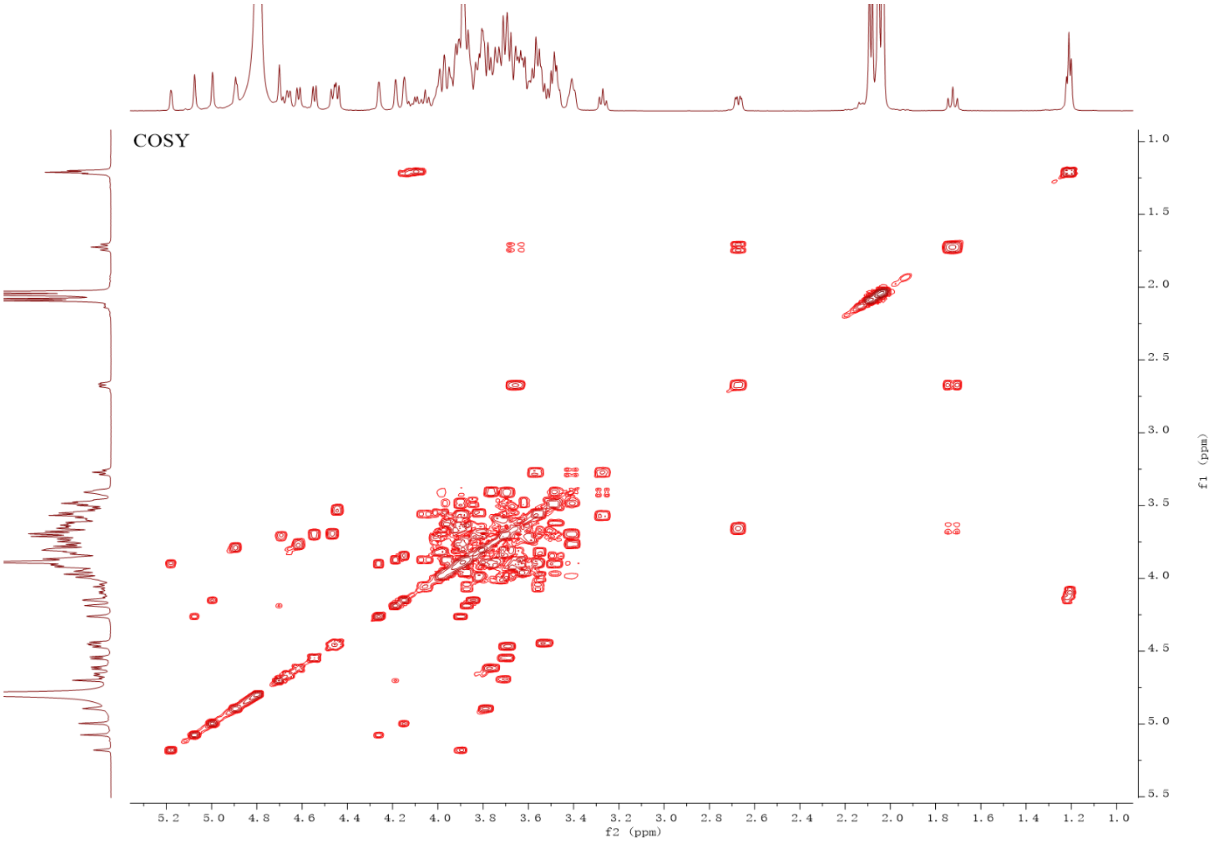

COSY
5.2 5.0 4.8 4.6 4.4 4.2 4.0 3.8 3.6 3.4 3.2 3.0 2.8 2.6 2.4 2.2 2.0 1.8 1.6 1.4 1.2 1.0
f2 (ppm)
1.0 1.5 2.0 2.5 3.0 3.5 4.0 4.5 5.0 5.5
f1 (ppm)

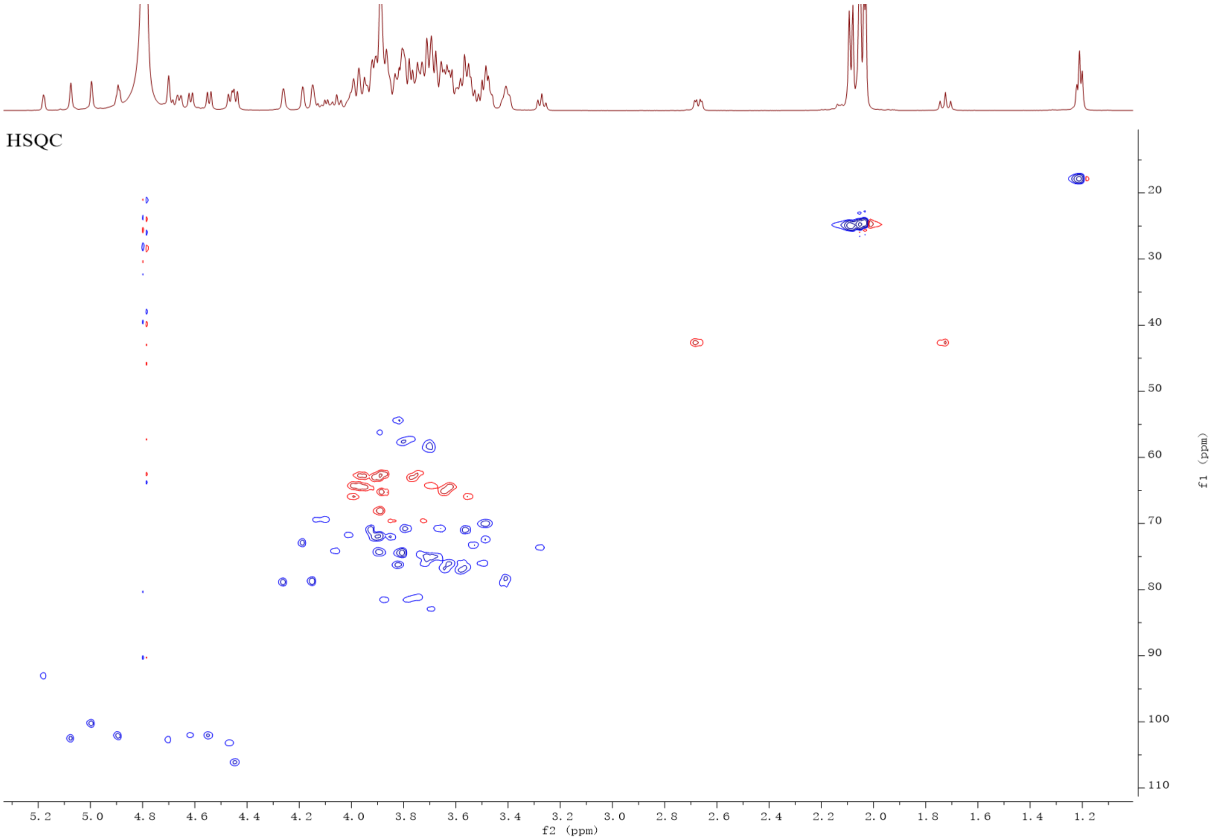
HSQC
f2 (ppm)
f1 (ppm)

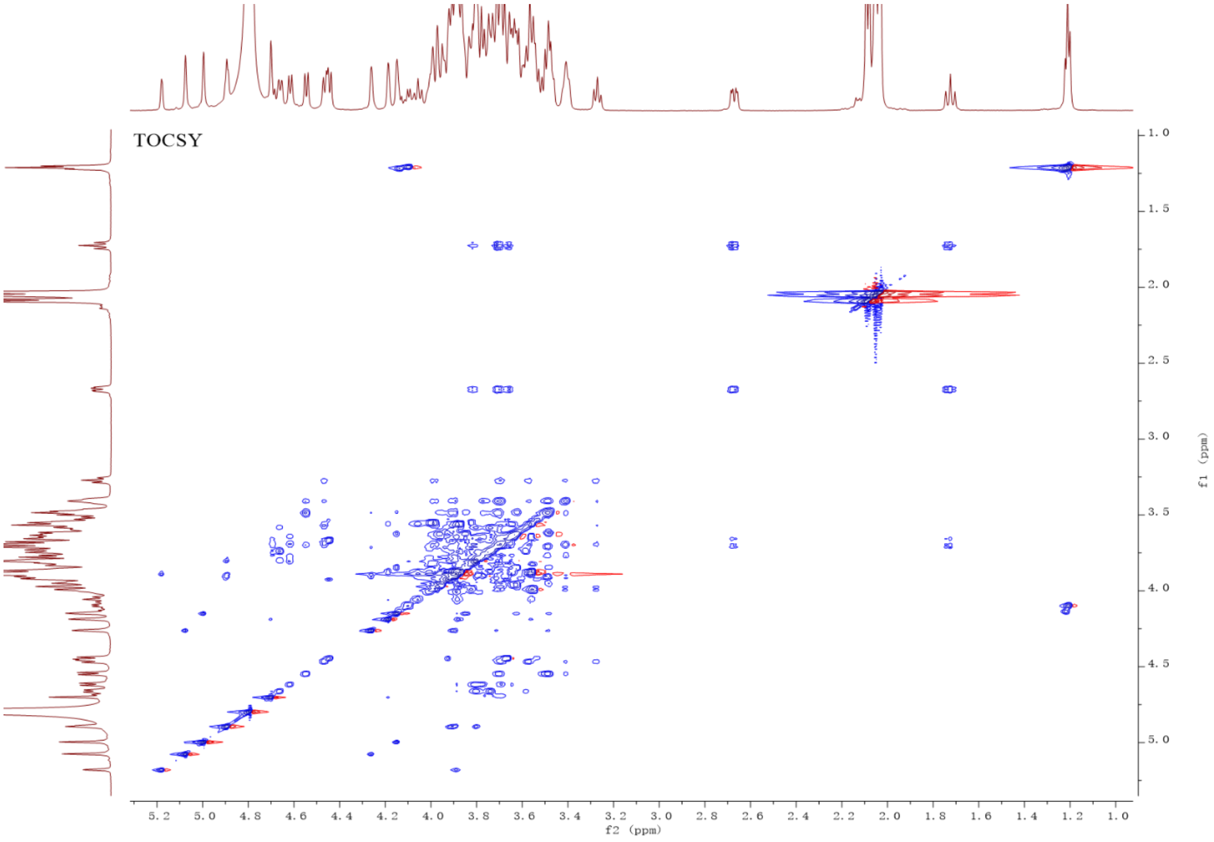
TOCSY
f2 (ppm)
f1 (ppm)

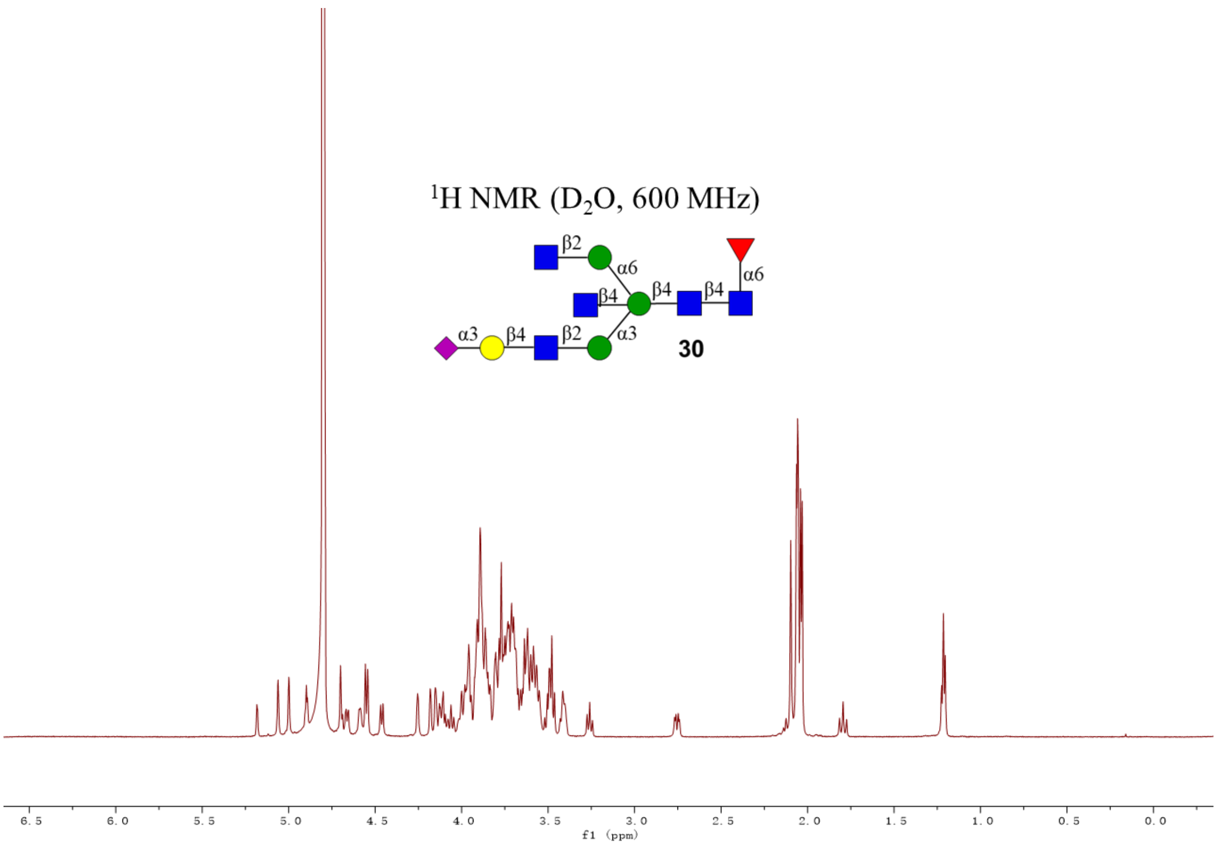

1H NMR (D2O, 600 MHz)
β2
α6
β4
β4
β4
α6
α3
β4
β2
α3
30
f1 (ppm)

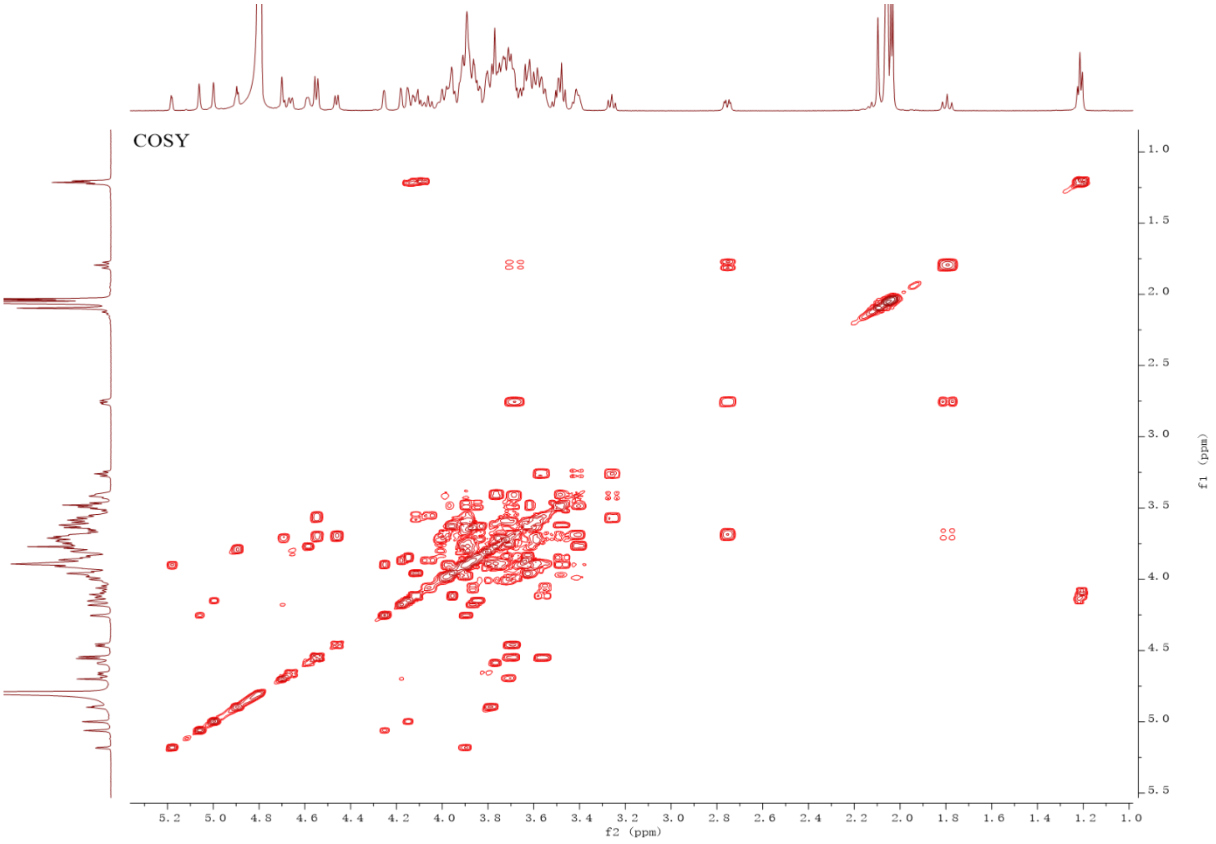

COSY
f2 (ppm)
f1 (ppm)

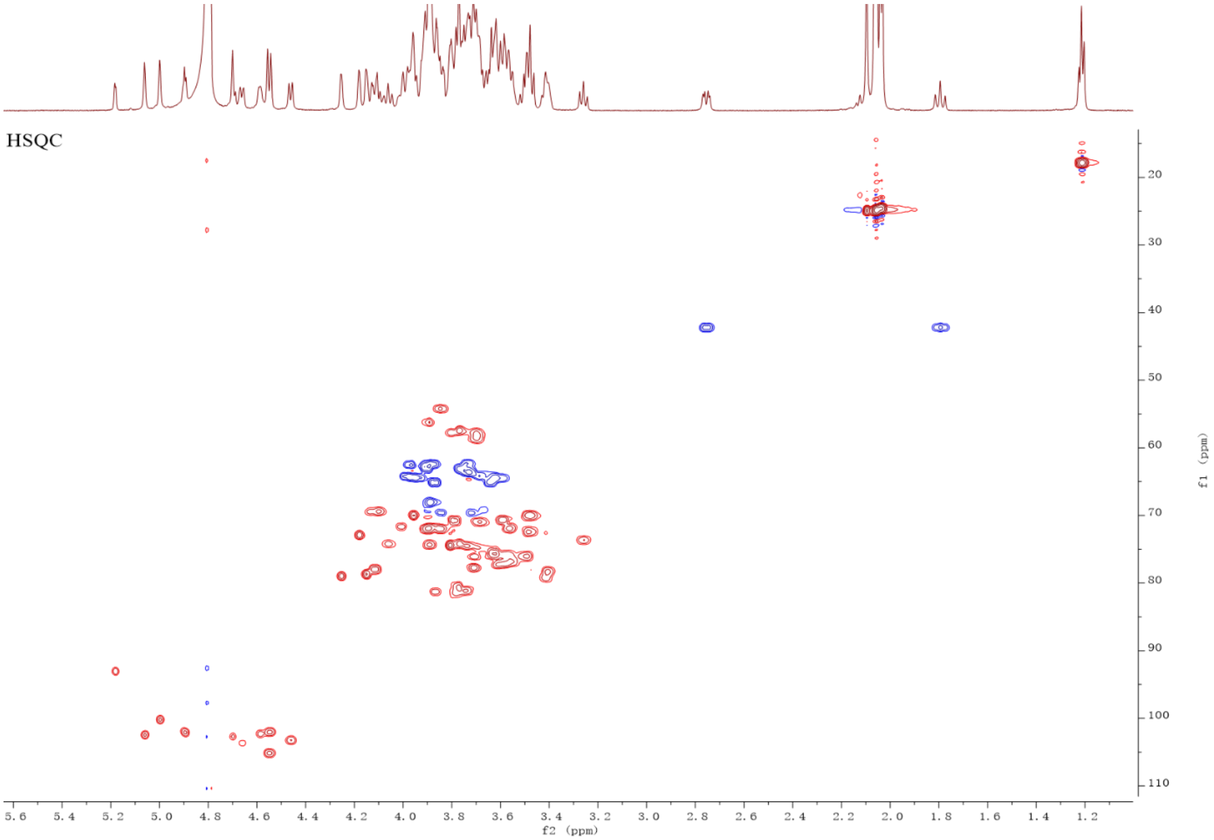
HSQC
f2 (ppm)
f1 (ppm)

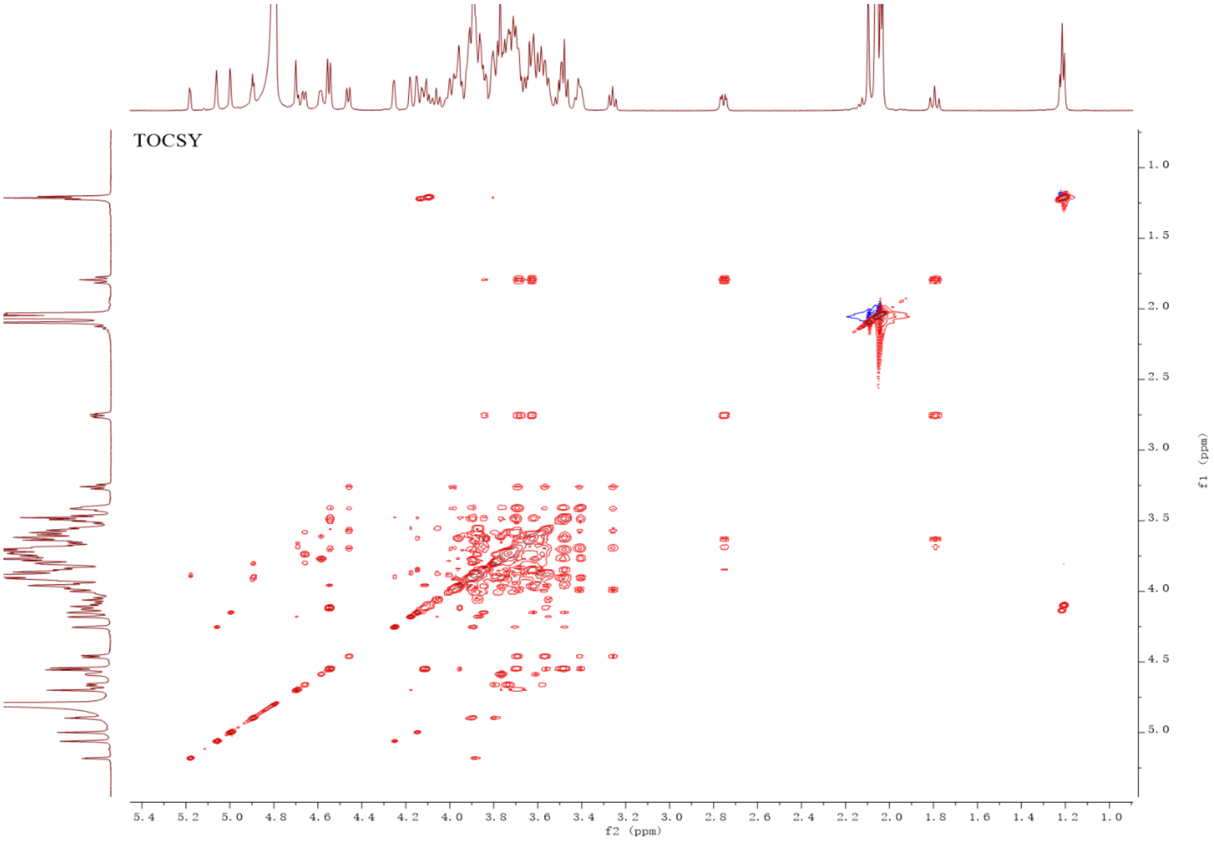
TOCSY
f2 (ppm)
f1 (ppm)

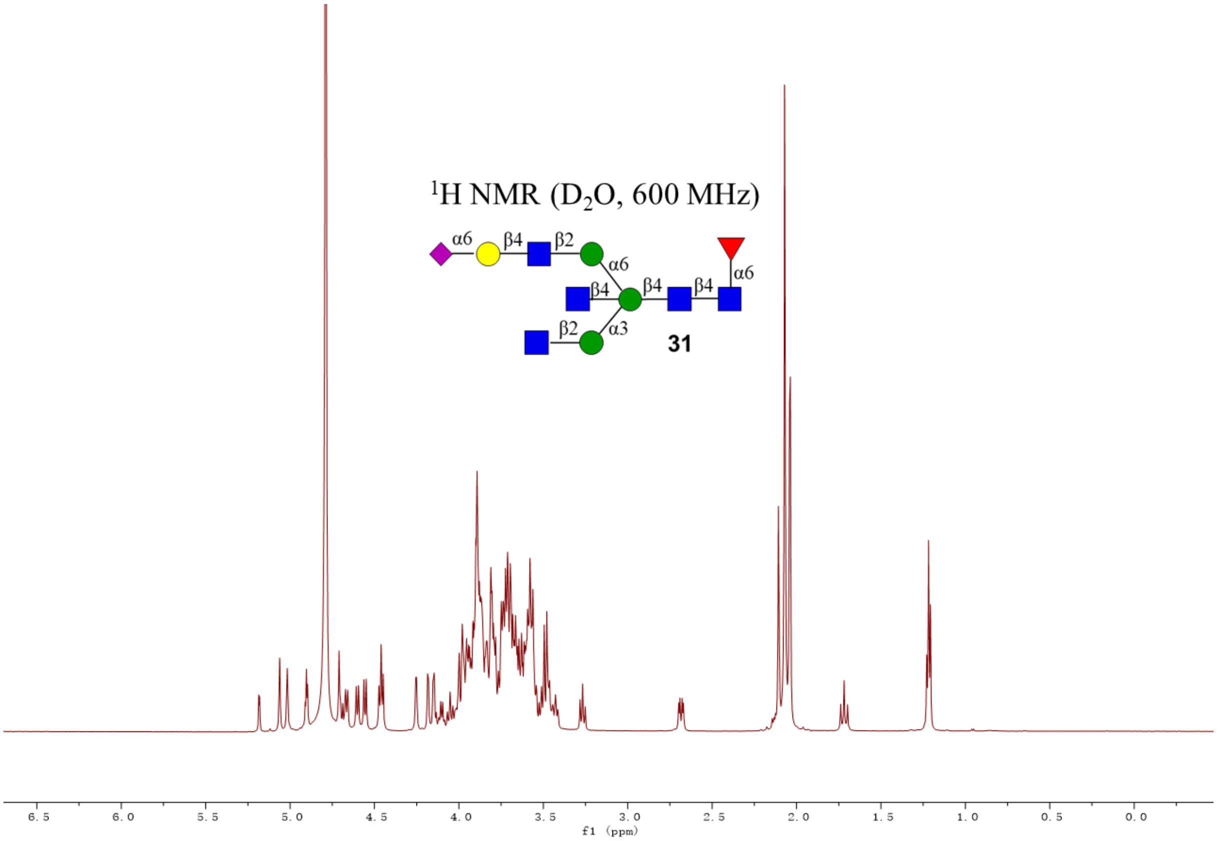
1H NMR (D2O, 600 MHz)
α6
β4
β2
α6
β4
β4
β4
α6
β2
α3
31
f1 (ppm)

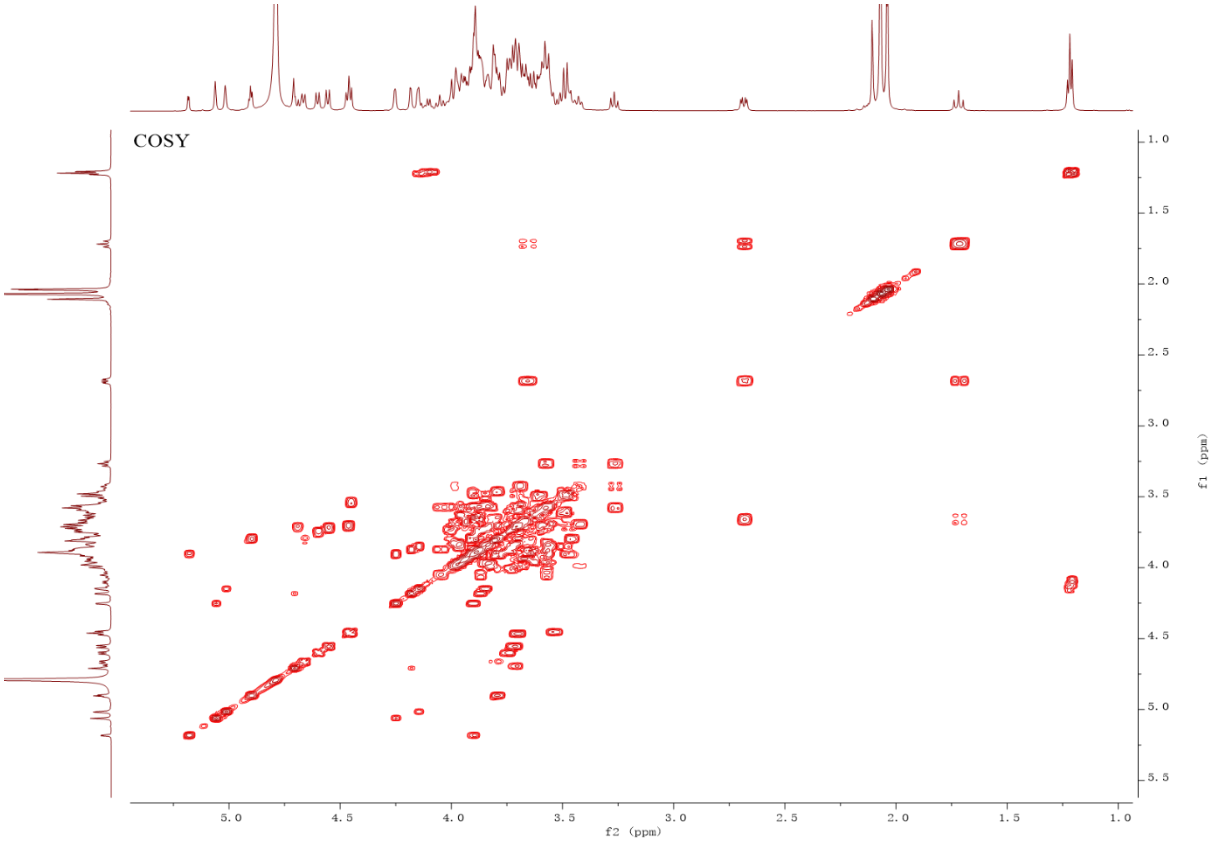
COSY
f2 (ppm)
f1 (ppm)

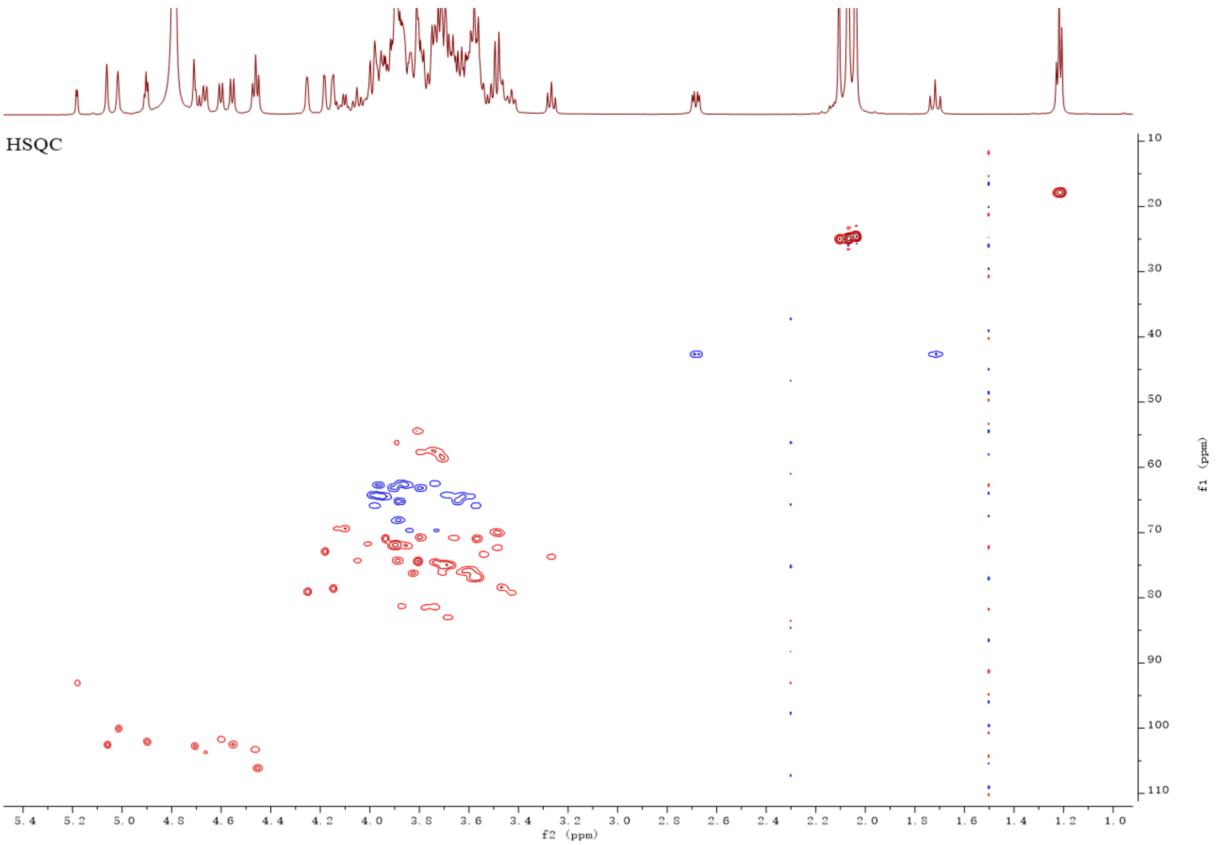
HSQC
f2 (ppm)
f1 (ppm)

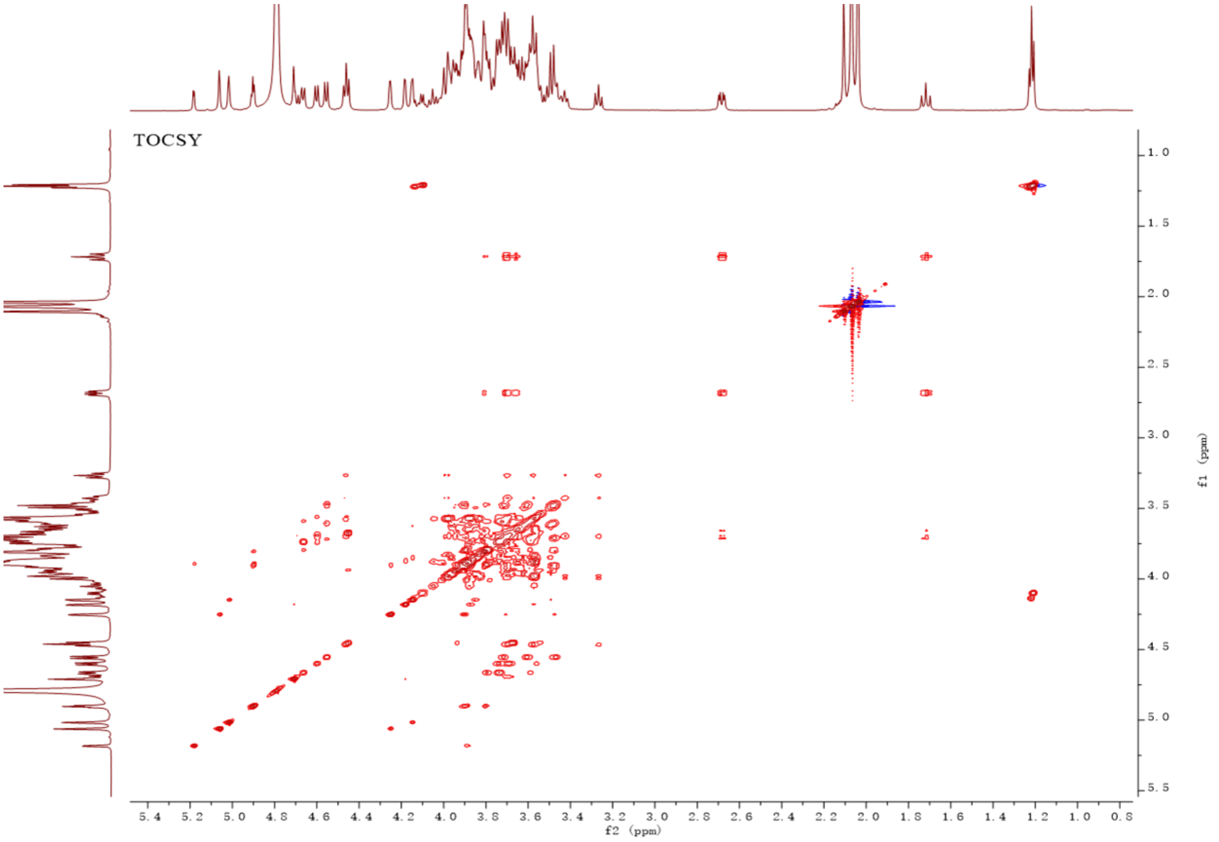
TOCSY
f2 (ppm)
f1 (ppm)

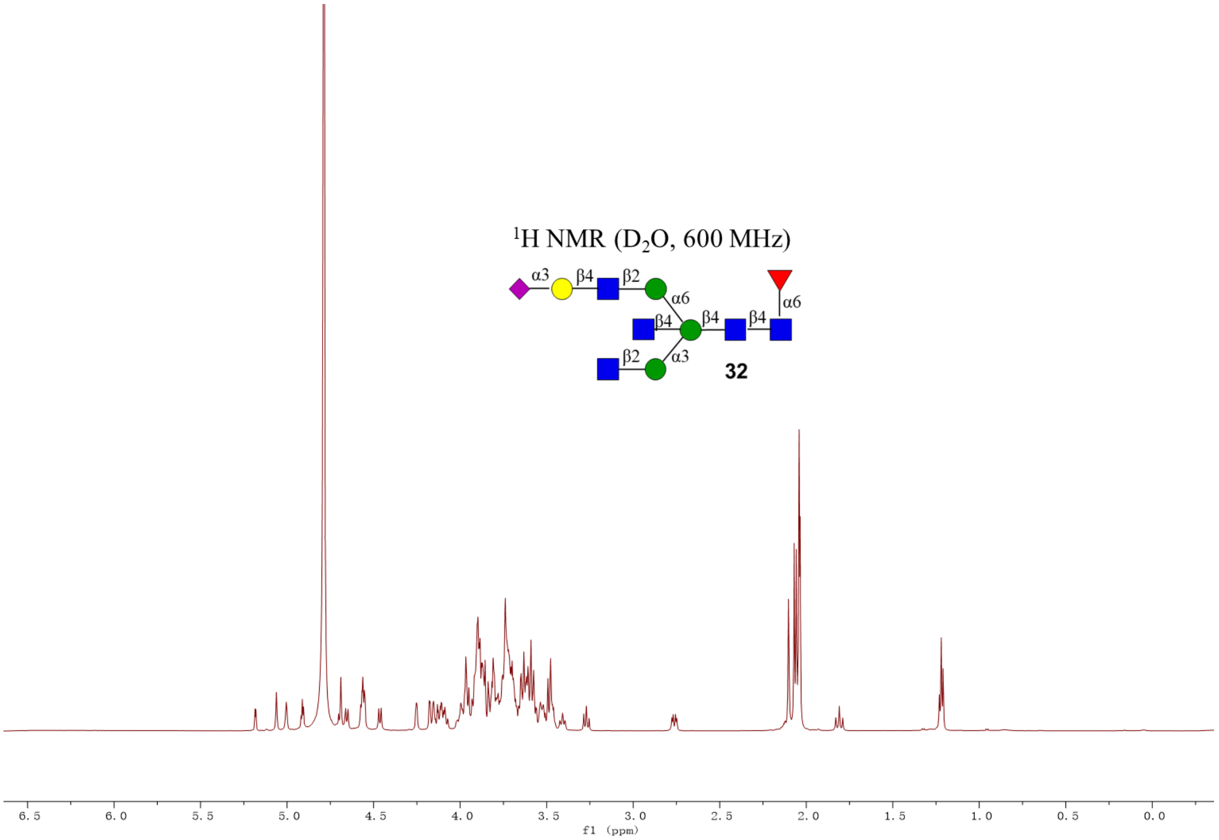

^{1}H NMR (D_2O, 600 MHz)
α3
β4
β2
α6
β4
β4
β4
α6
β2
α3
32
6.5 6.0 5.5 5.0 4.5 4.0 3.5 3.0 2.5 2.0 1.5 1.0 0.5 0.0
f1 (ppm)

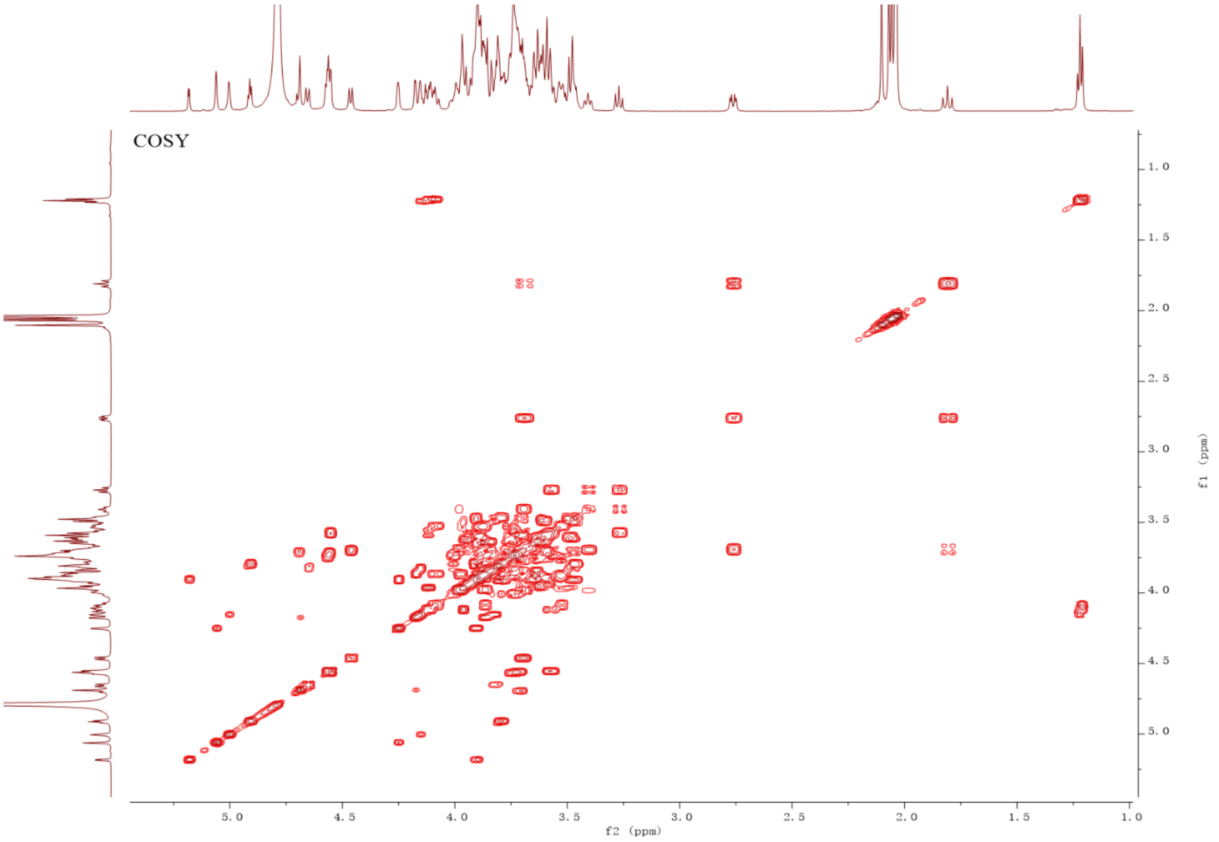

COSY
5.0 4.5 4.0 3.5 3.0 2.5 2.0 1.5 1.0
f2 (ppm)
1.0 1.5 2.0 2.5 3.0 3.5 4.0 4.5 5.0
f1 (ppm)

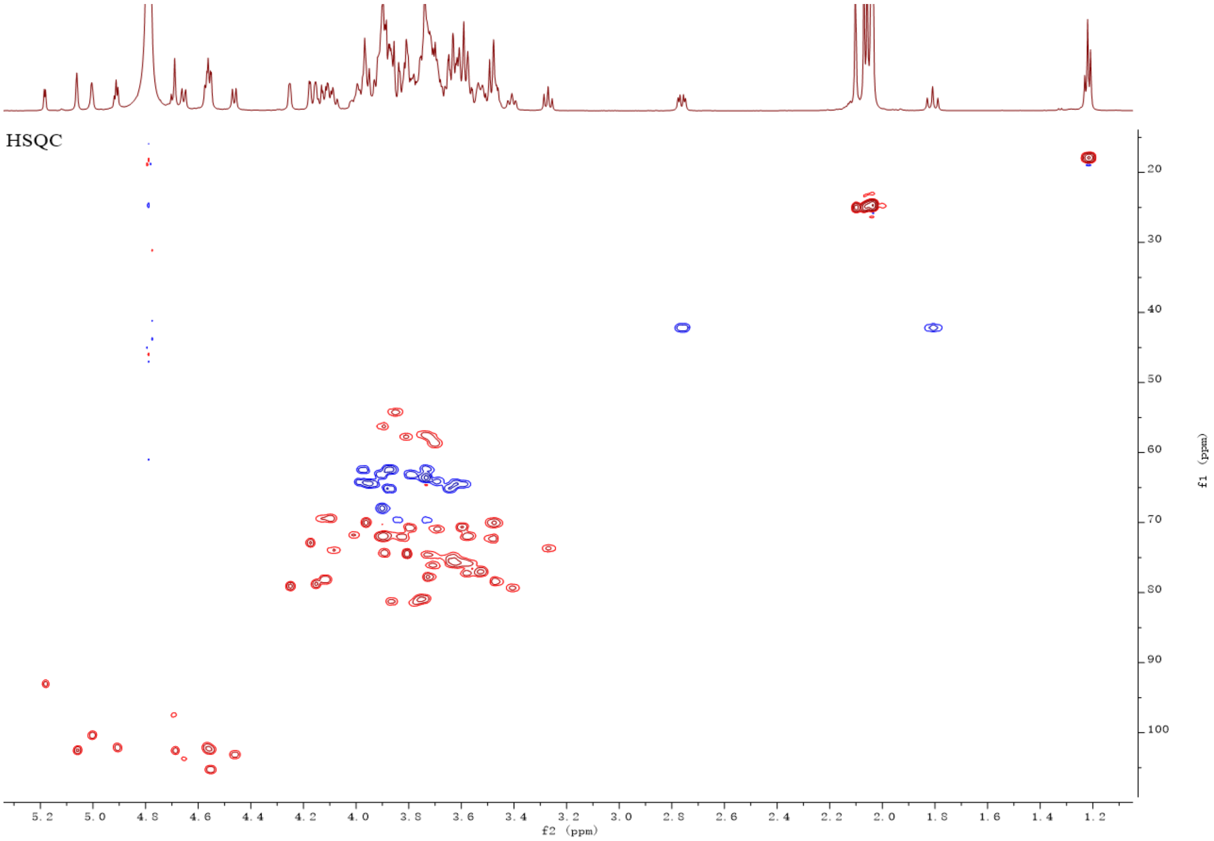
HSQC
f2 (ppm)
f1 (ppm)

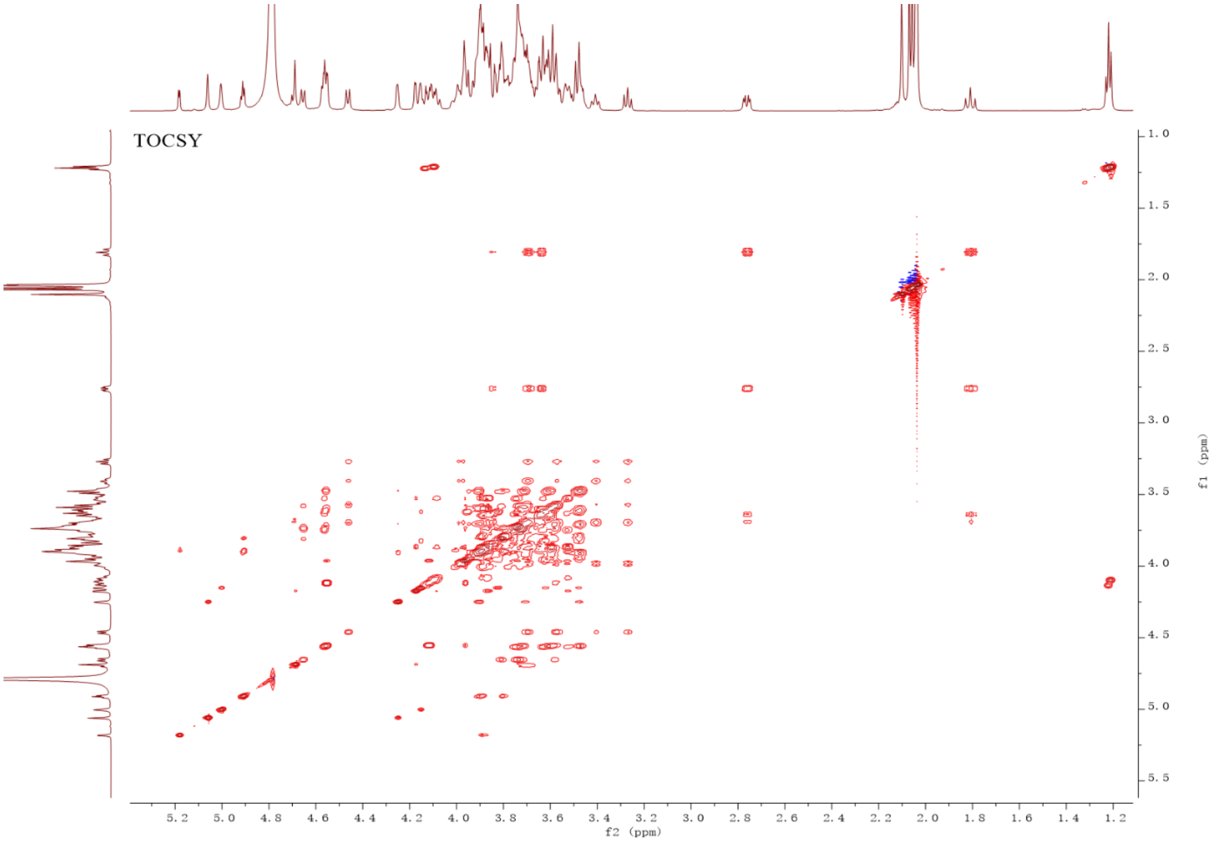
TOCSY
f2 (ppm)
f1 (ppm)

^{1}H NMR (D_2O, 600 MHz)

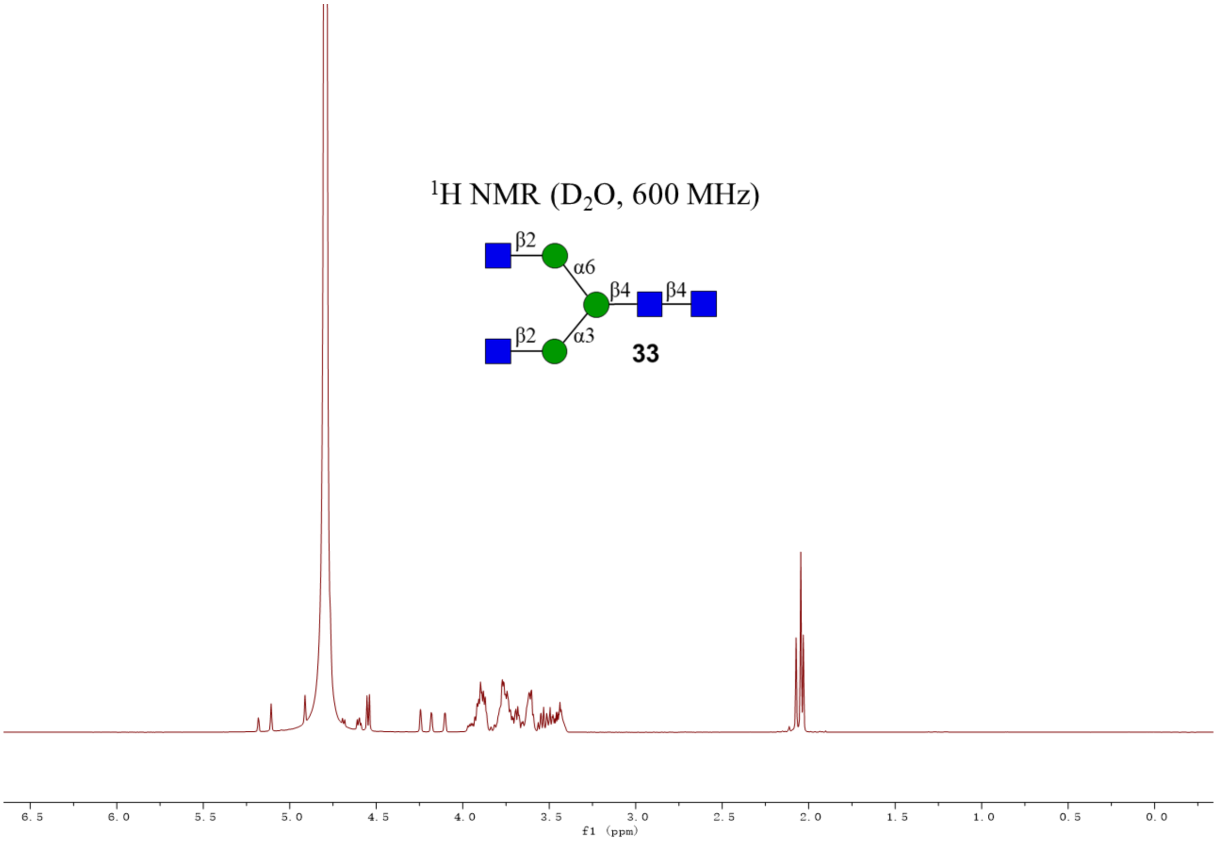

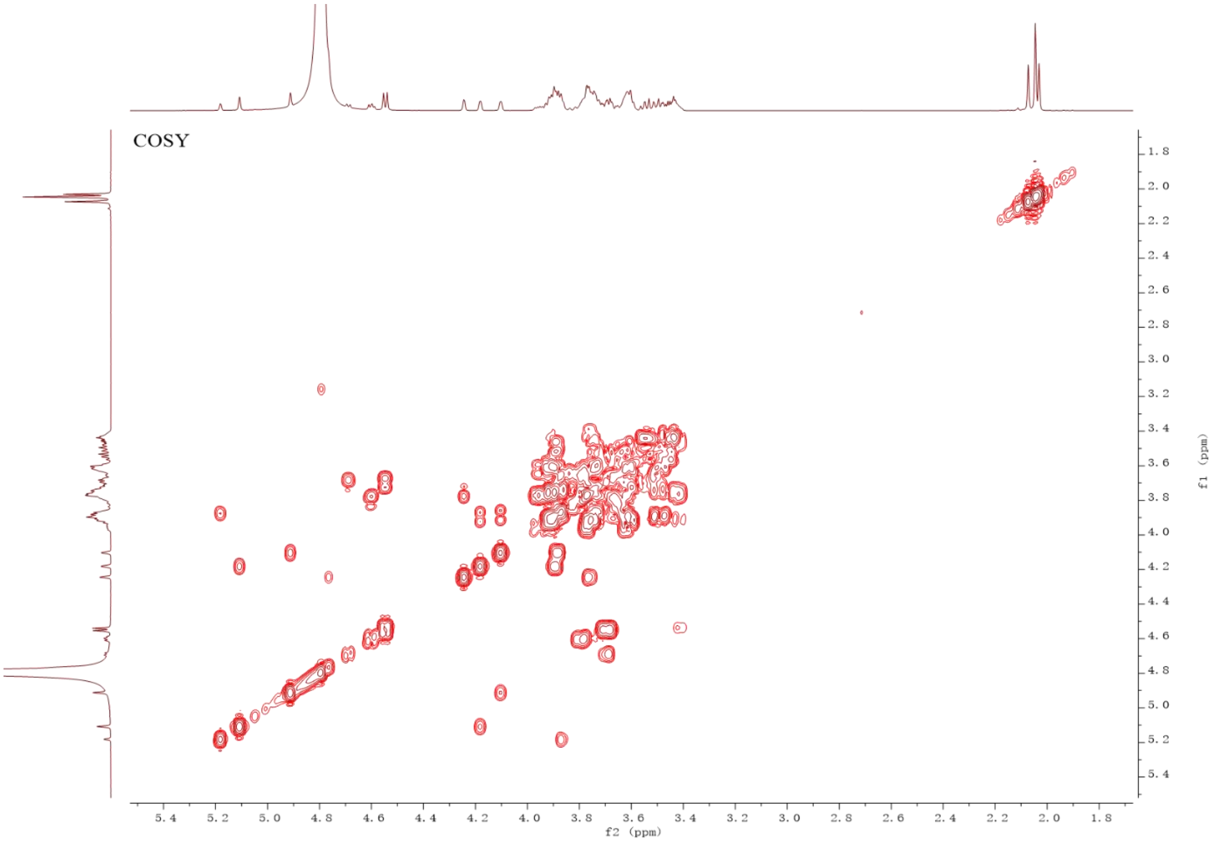

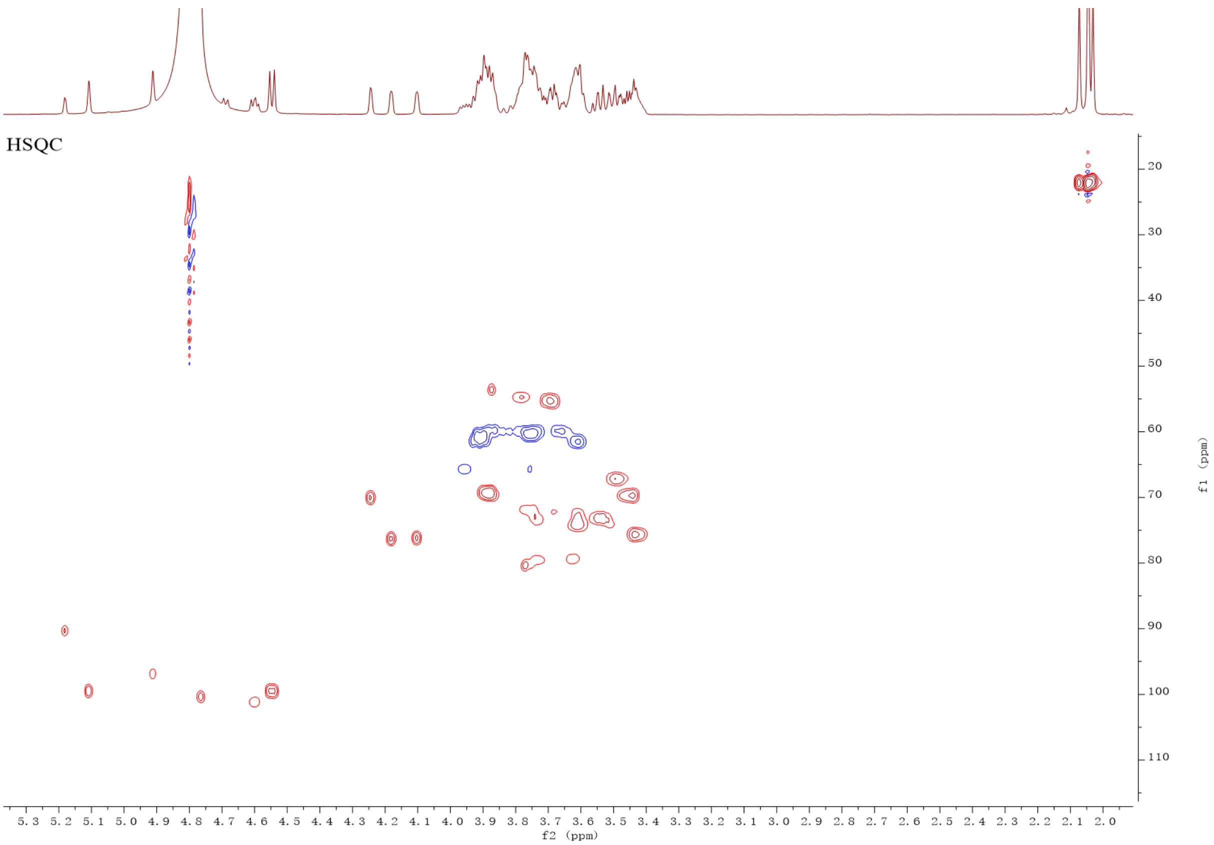
HSQC
f2 (ppm)
f1 (ppm)

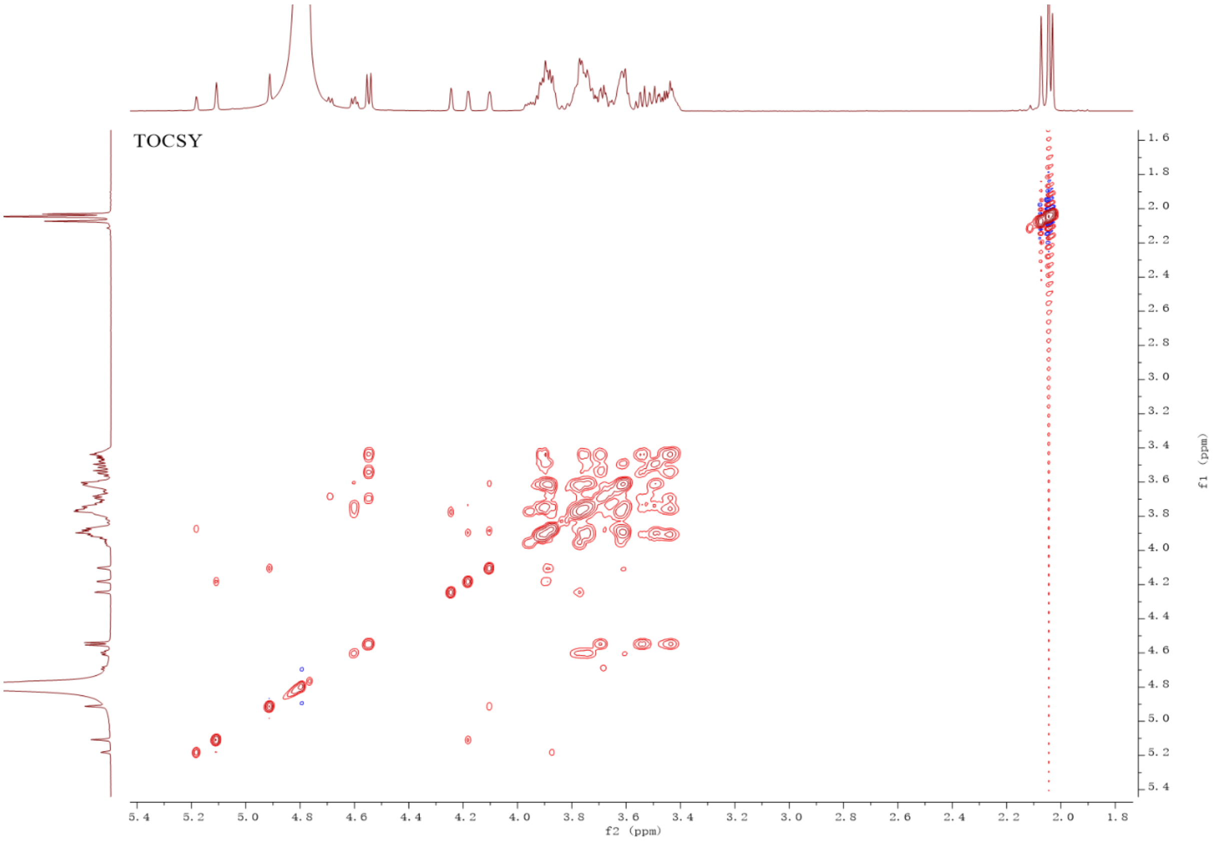
TOCSY
f2 (ppm)
f1 (ppm)

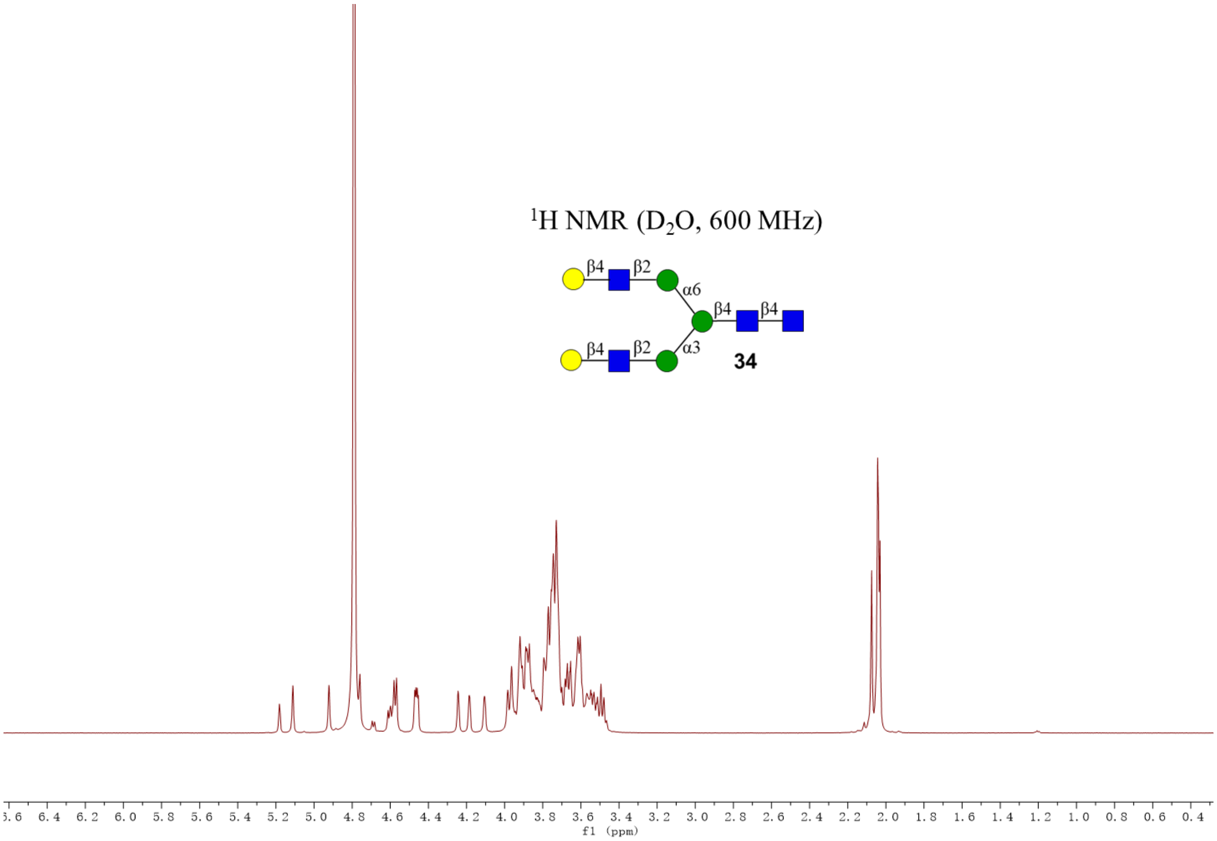
^{1}H NMR (D_2O, 600 MHz)
β4
β2
α6
β4
β4
β4
β2
α3
34
f1 (ppm)

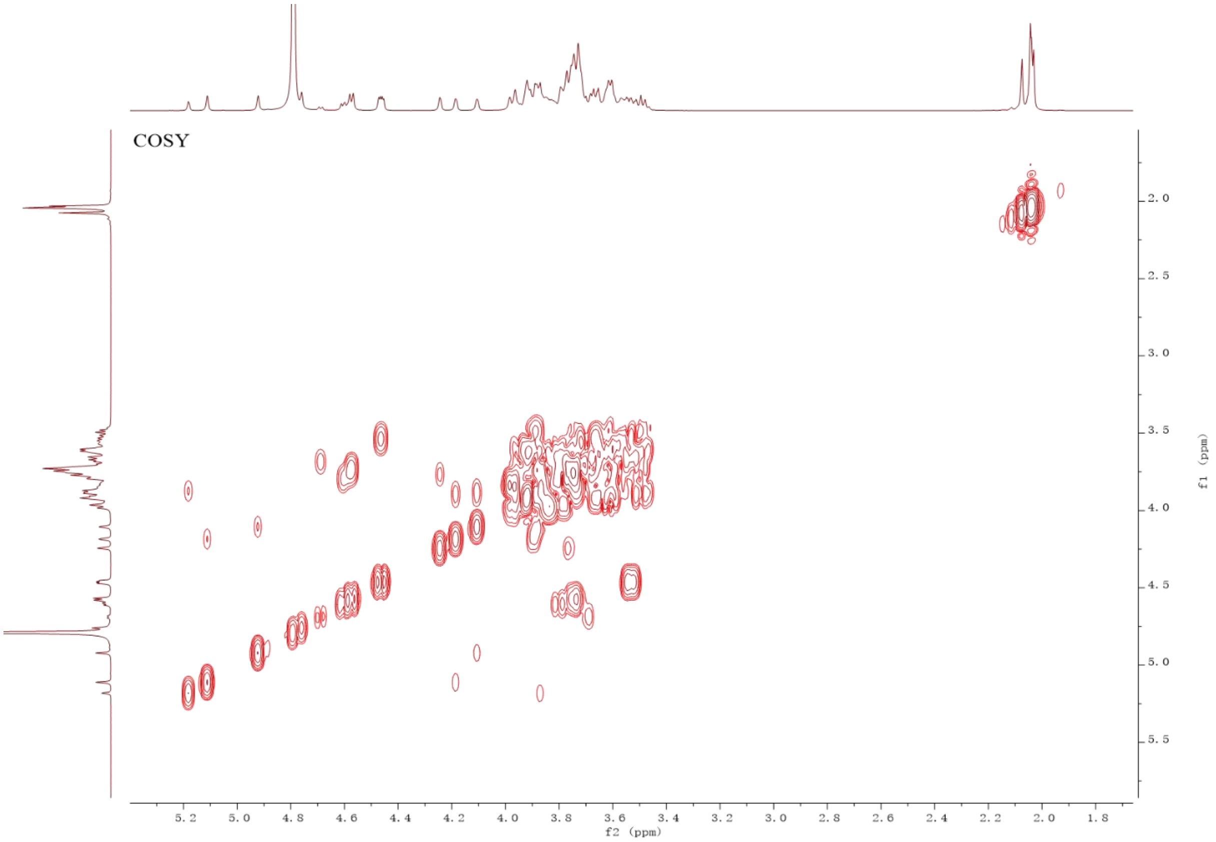
COSY
f2 (ppm)
f1 (ppm)

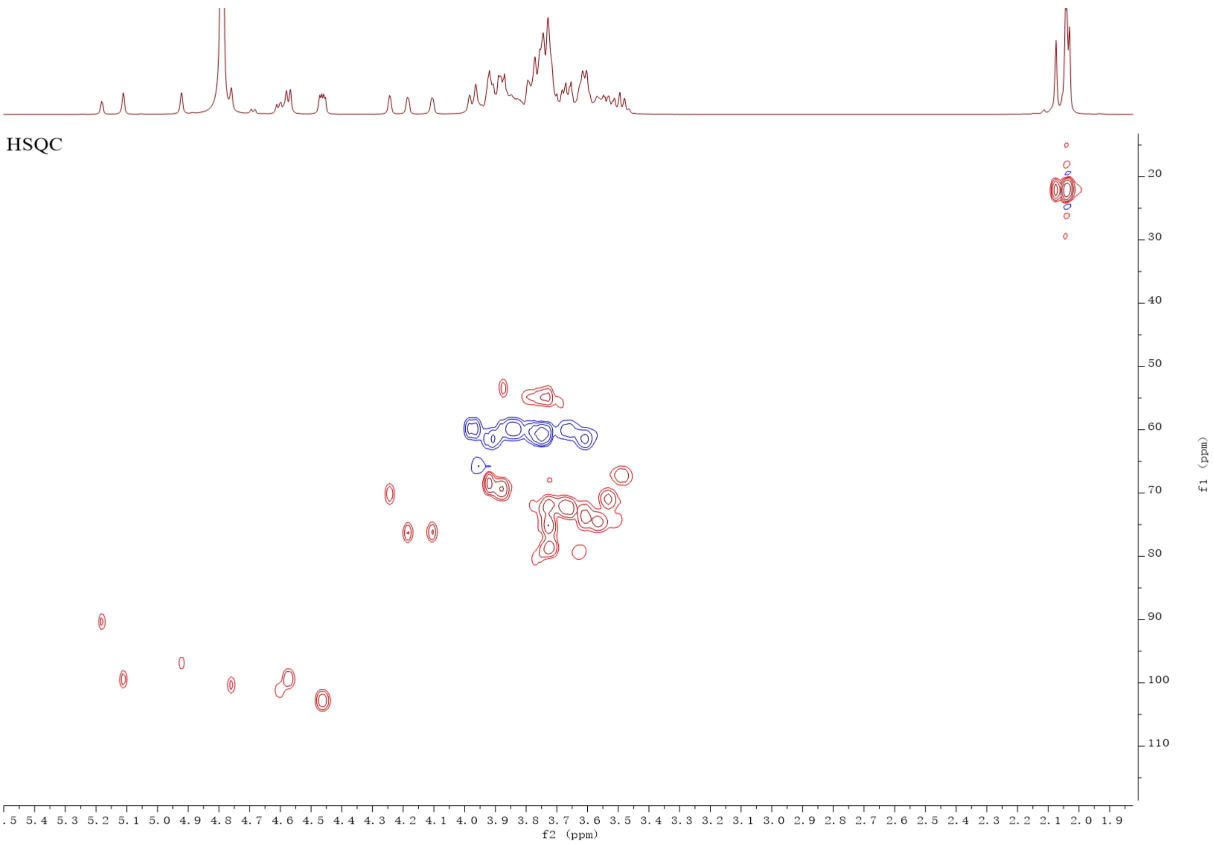
HSQC
f2 (ppm)
f1 (ppm)

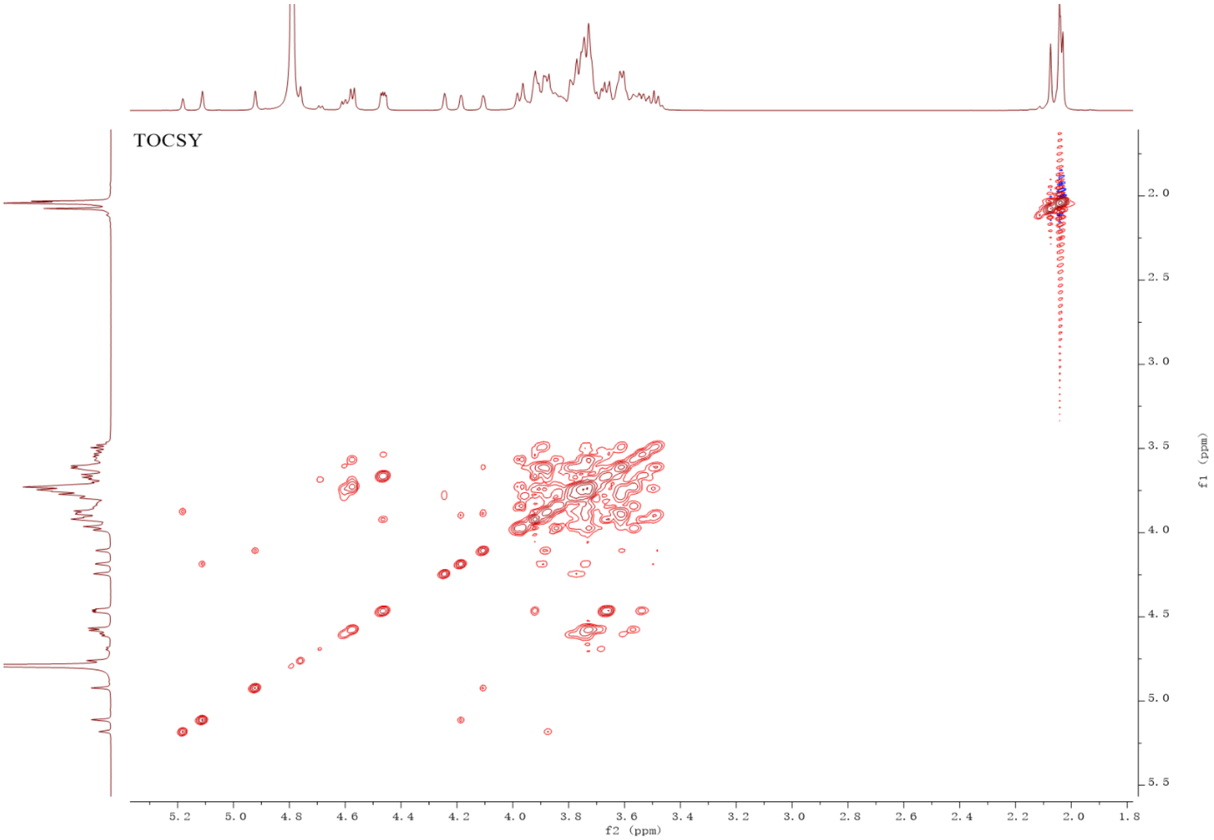
TOCSY
f2 (ppm)
f1 (ppm)

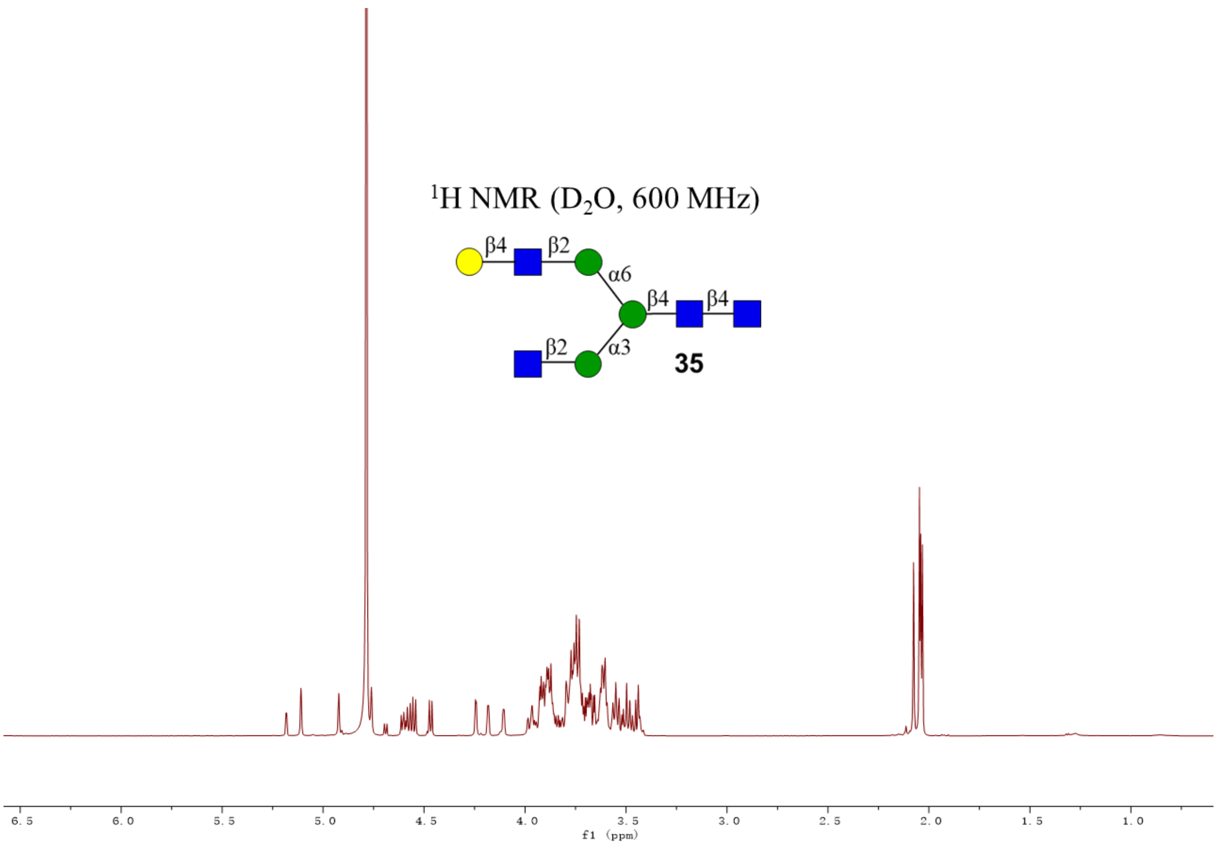
1H NMR (D2O, 600 MHz)
β4
β2
α6
β4
β4
β2
α3
35
f1 (ppm)

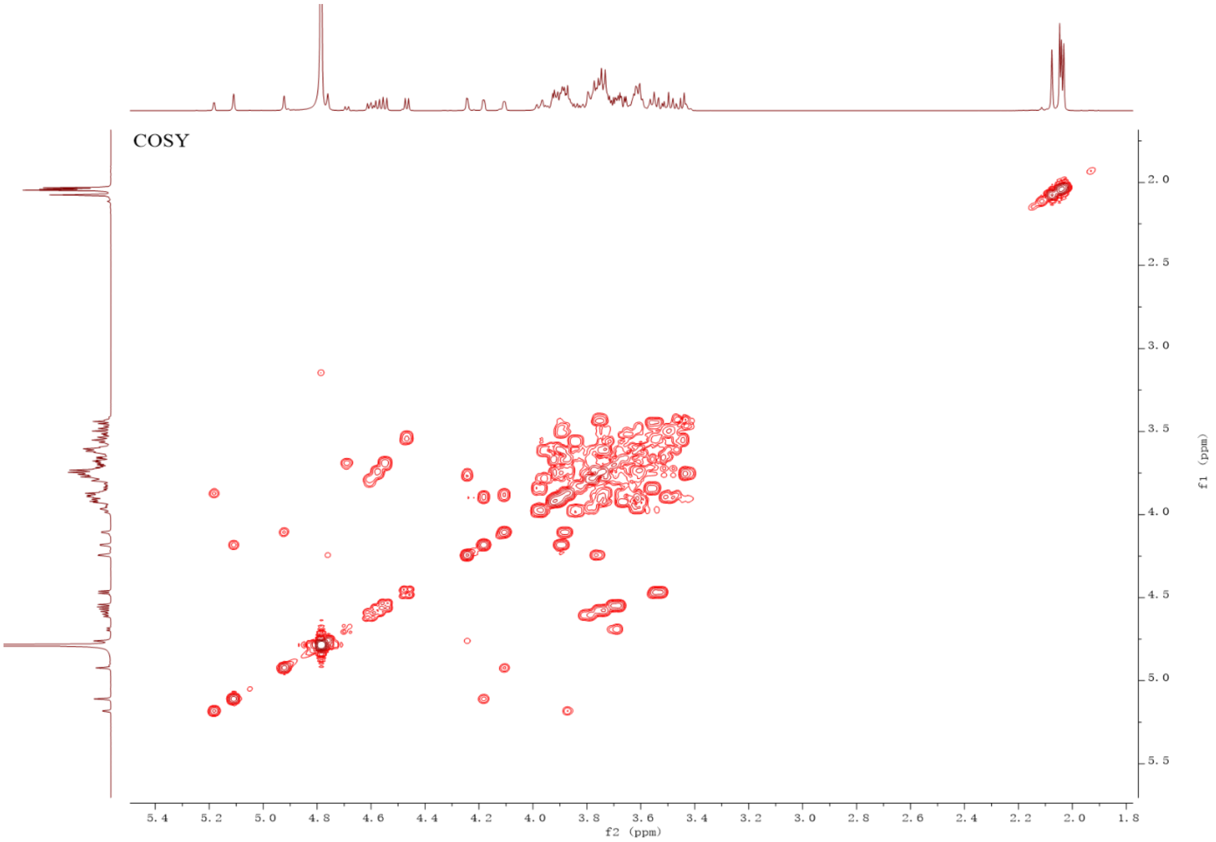
COSY
f2 (ppm)
f1 (ppm)

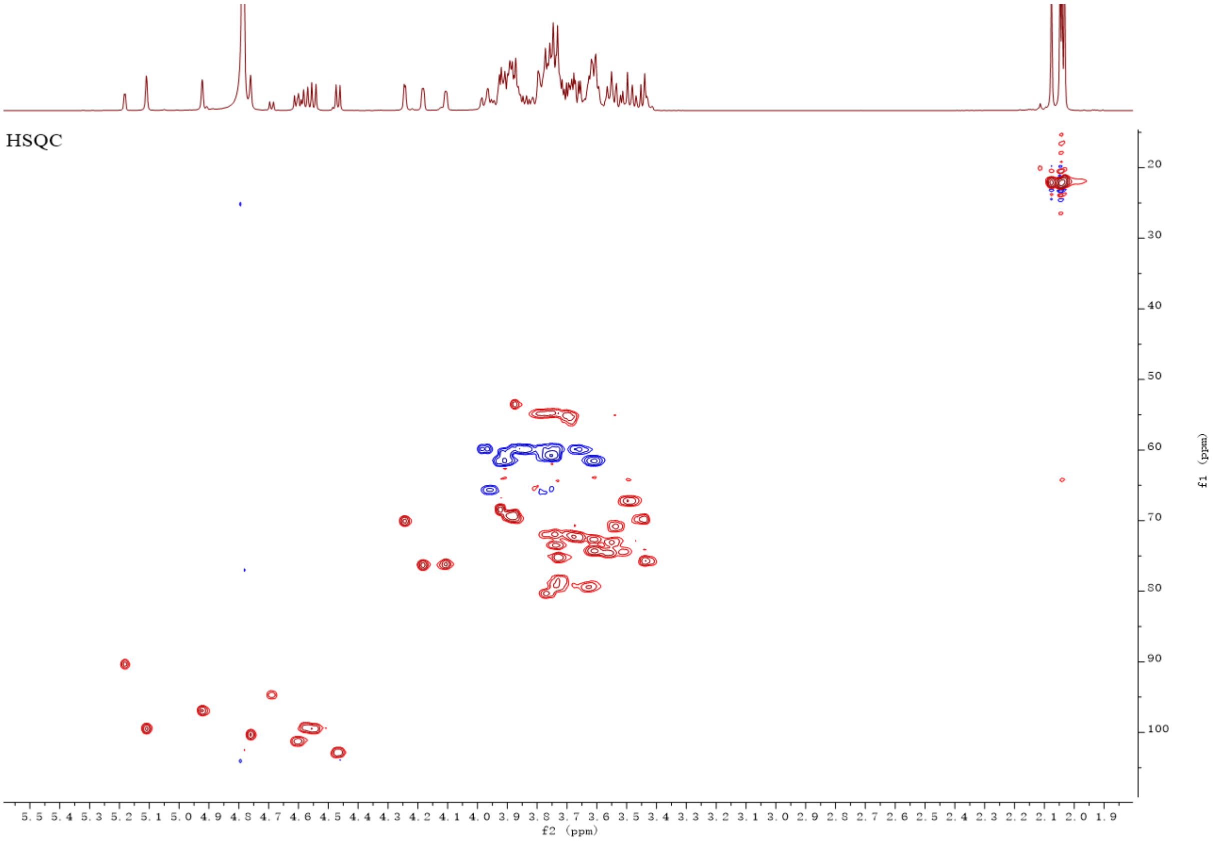
HSQC
f2 (ppm)
f1 (ppm)

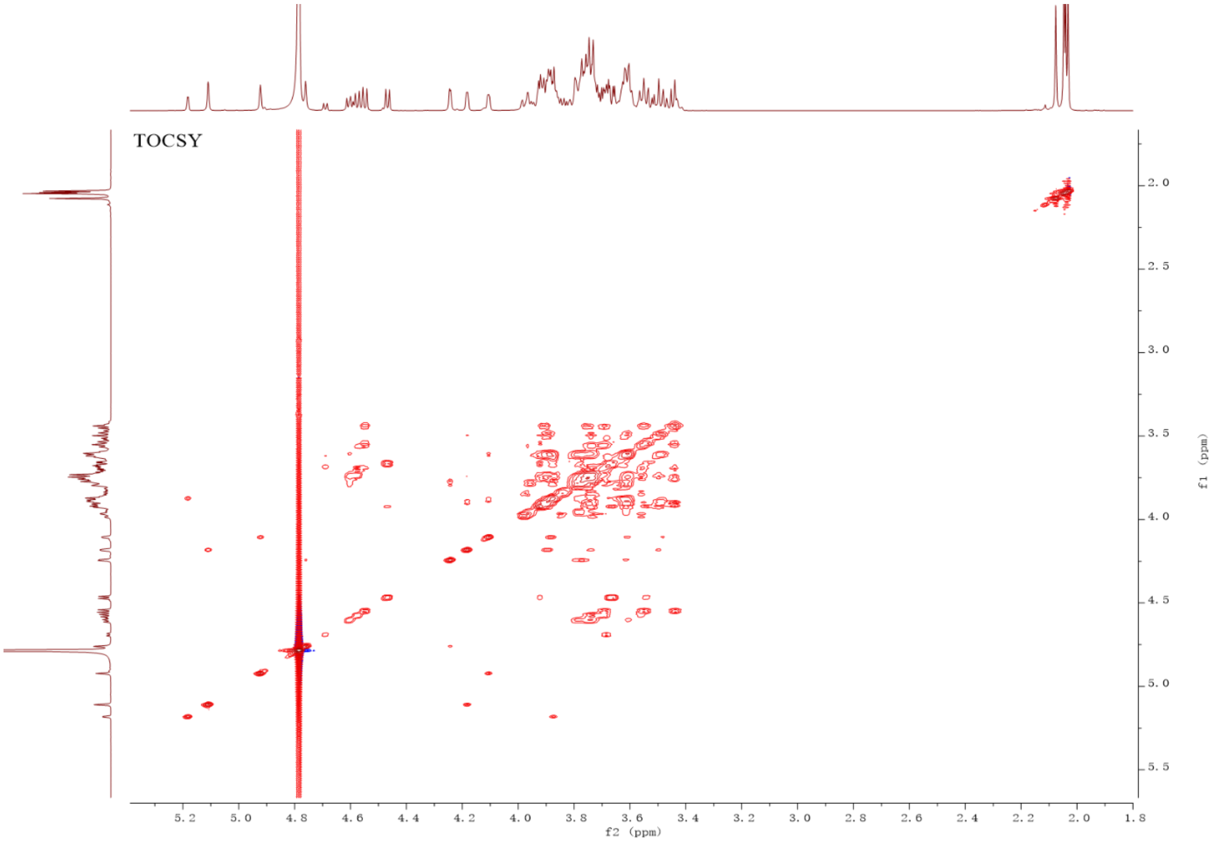
TOCSY
f2 (ppm)
f1 (ppm)

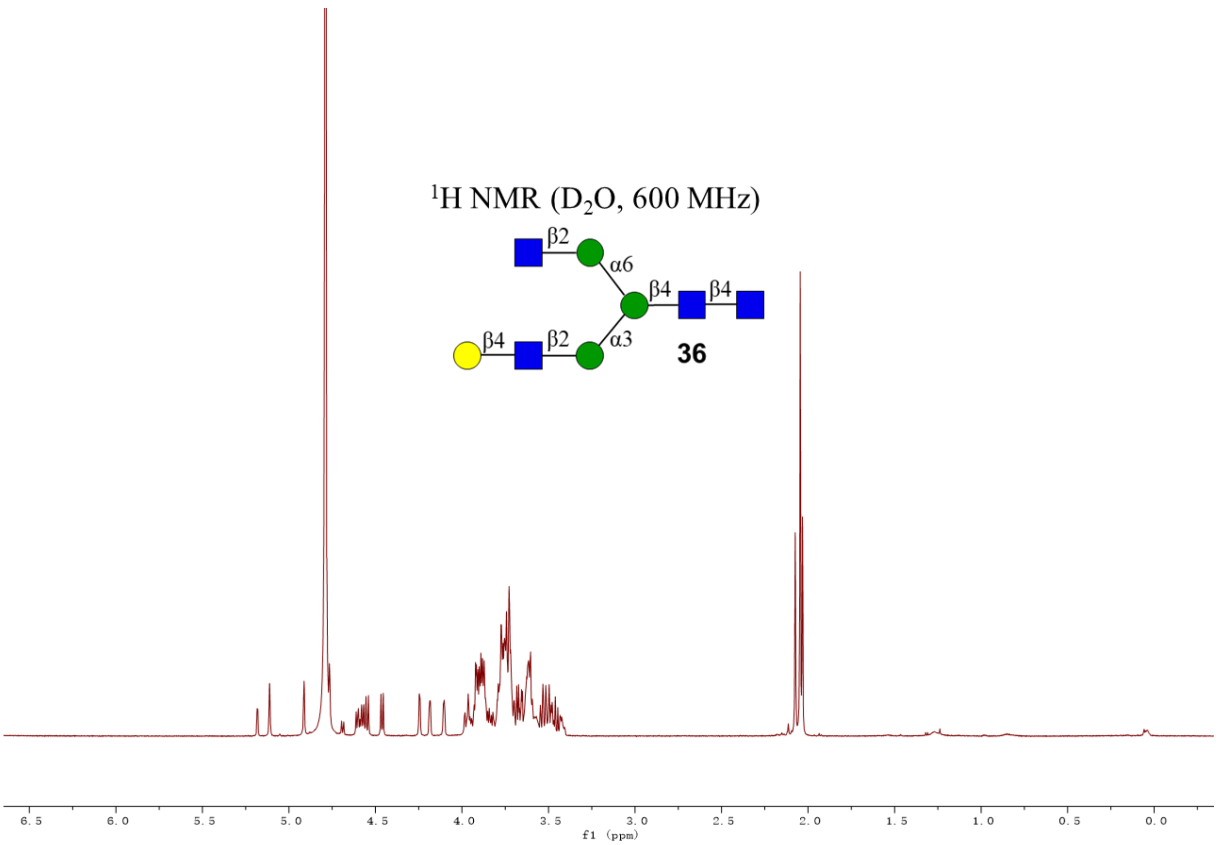

^{1}H NMR (D_2O, 600 MHz)
β2
α6
β4
β4
β4
β2
α3
36
f1 (ppm)

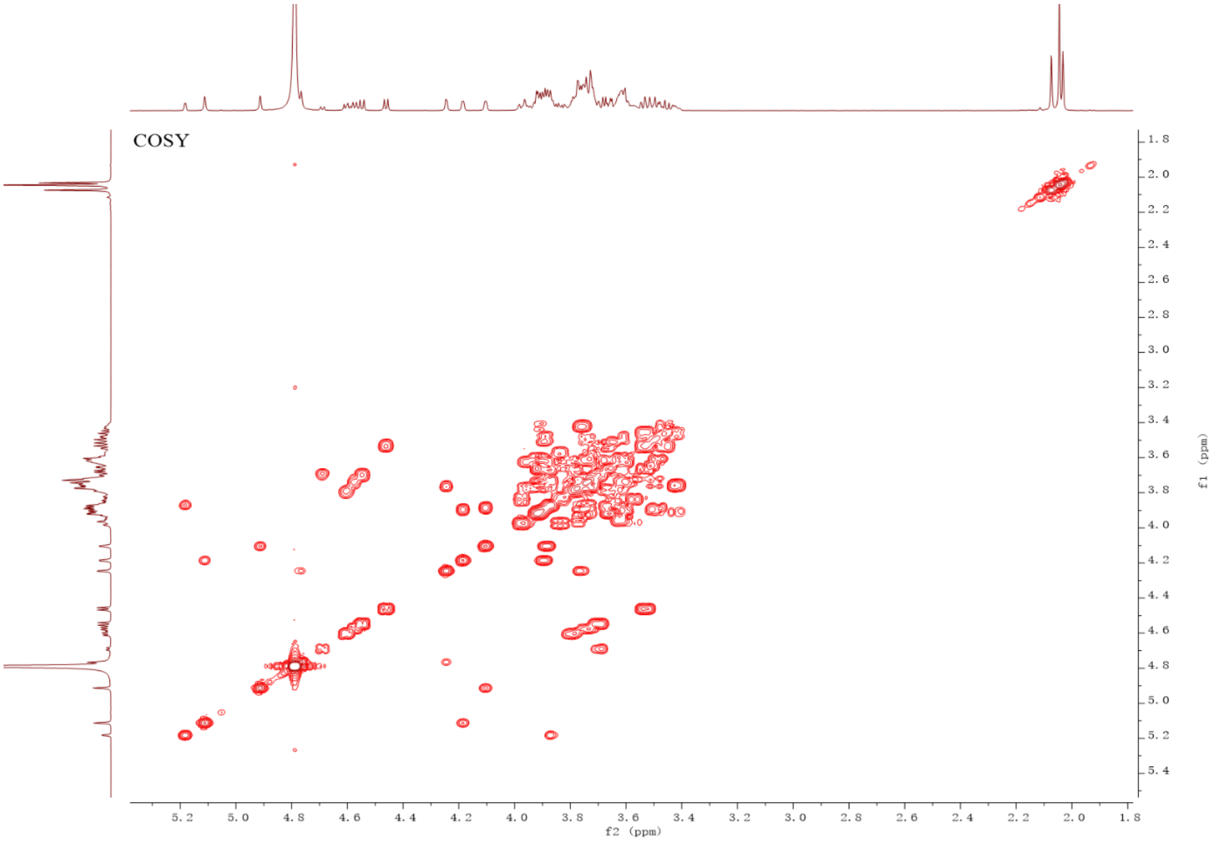

COSY
f2 (ppm)
f1 (ppm)

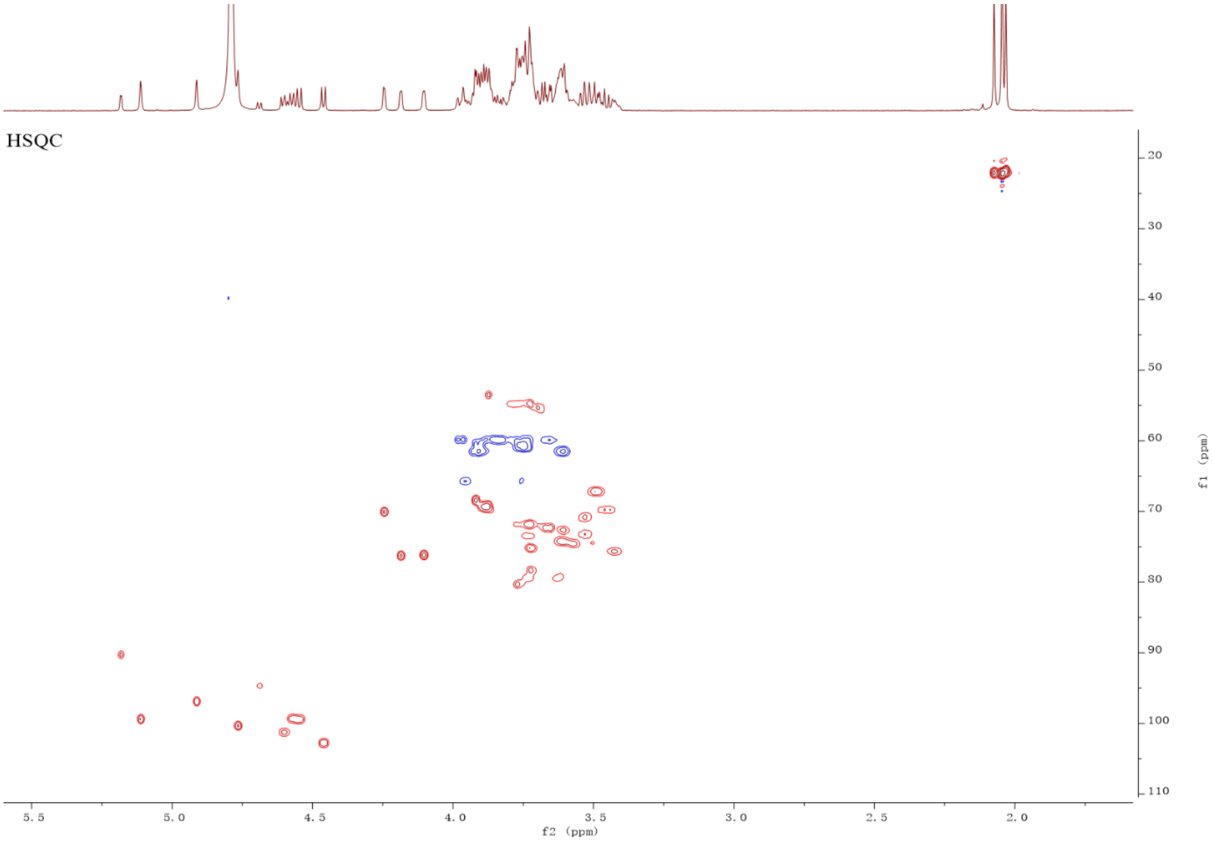
HSQC
f2 (ppm)
f1 (ppm)

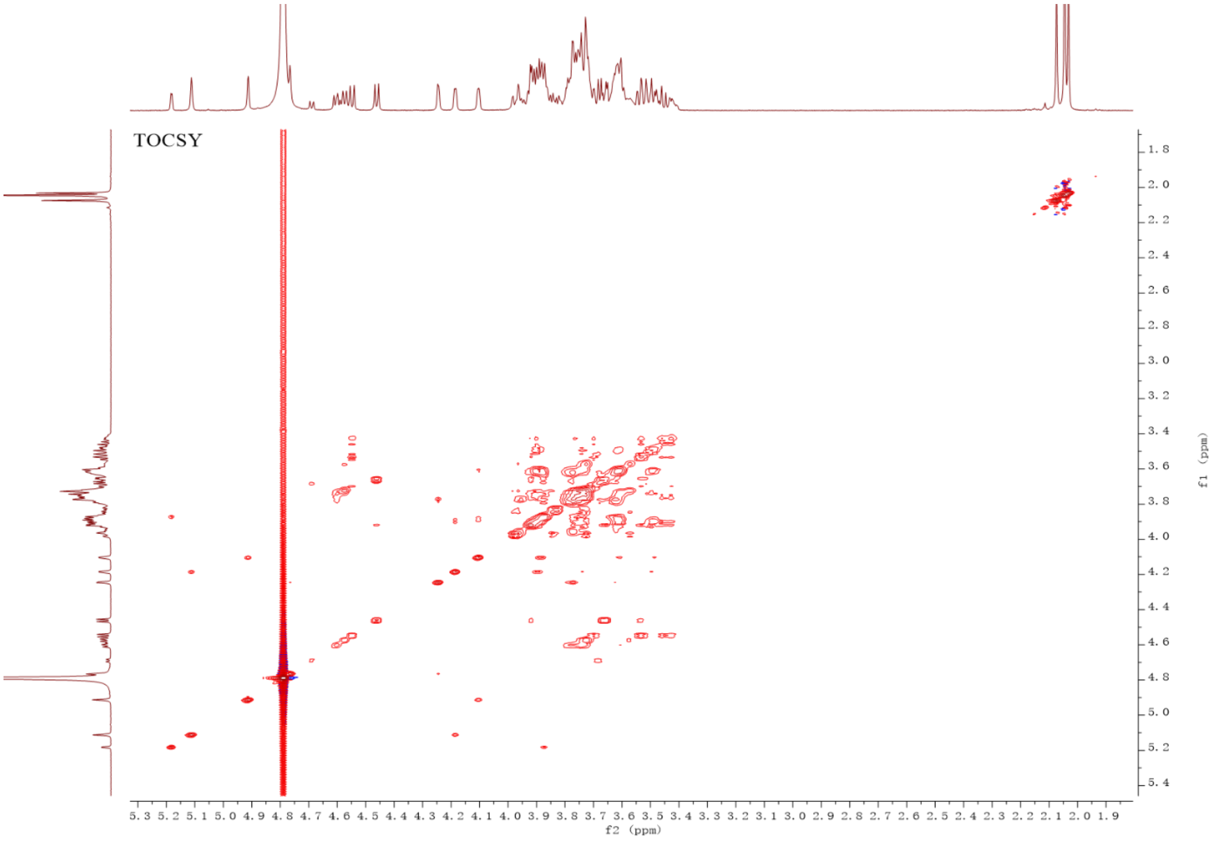
TOCSY
f2 (ppm)
f1 (ppm)

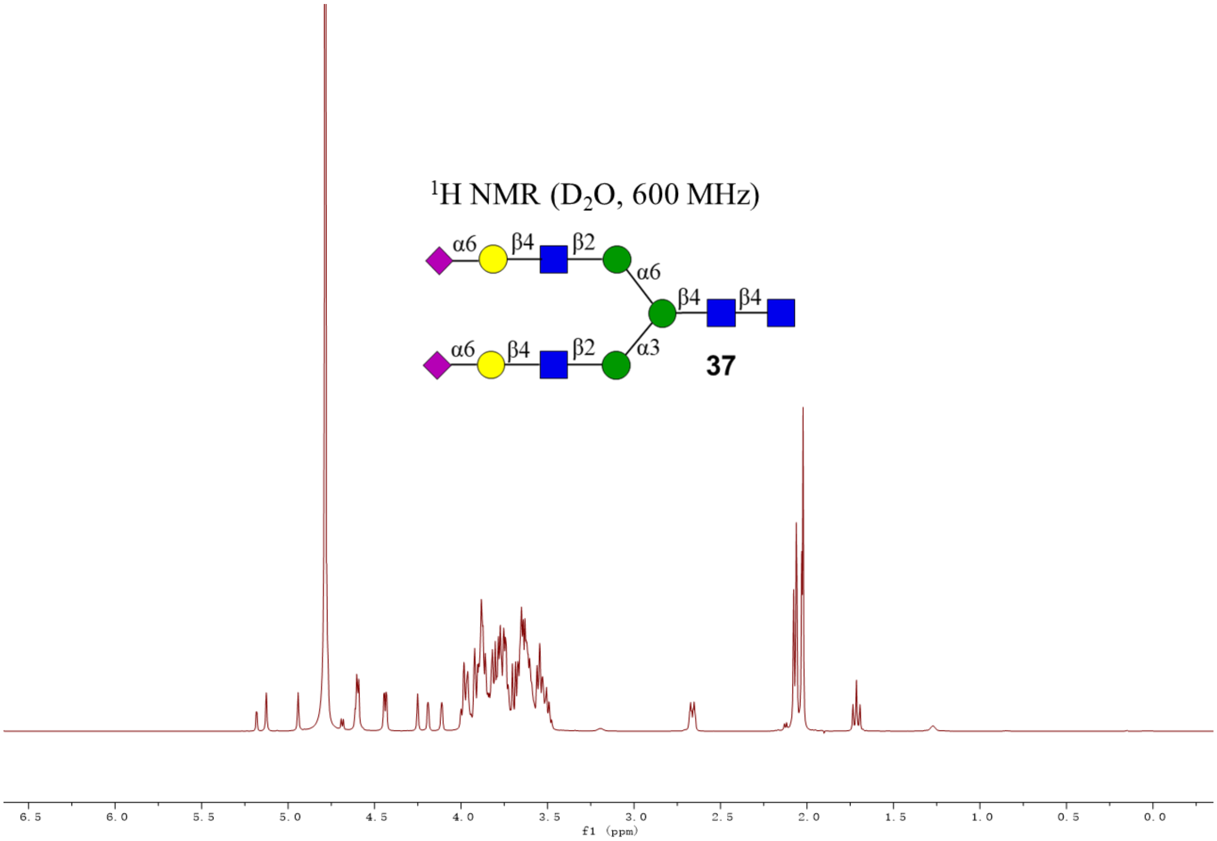

^{1}H NMR (D_2O, 600 MHz)
α6
β4
β2
α6
β4
β4
α6
β4
β2
α3
37
f1 (ppm)

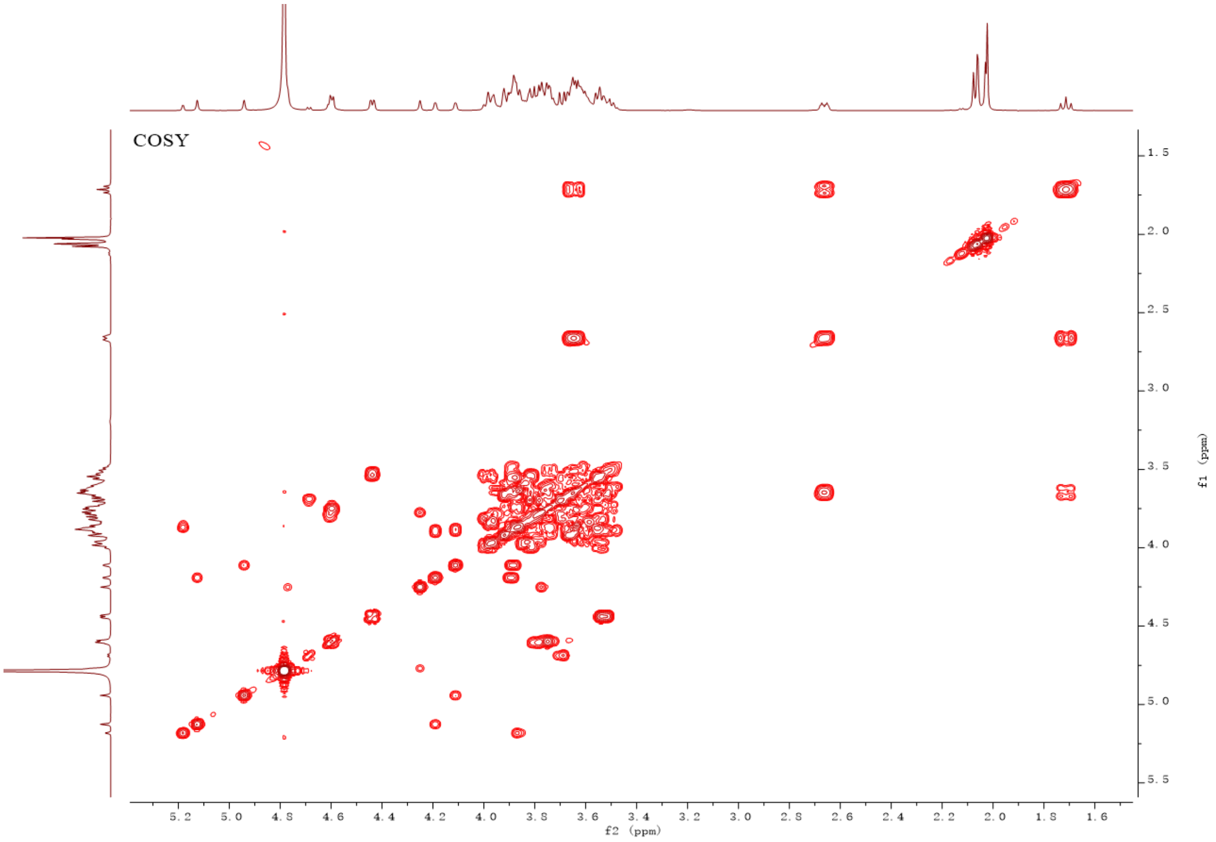

COSY
f2 (ppm)
f1 (ppm)

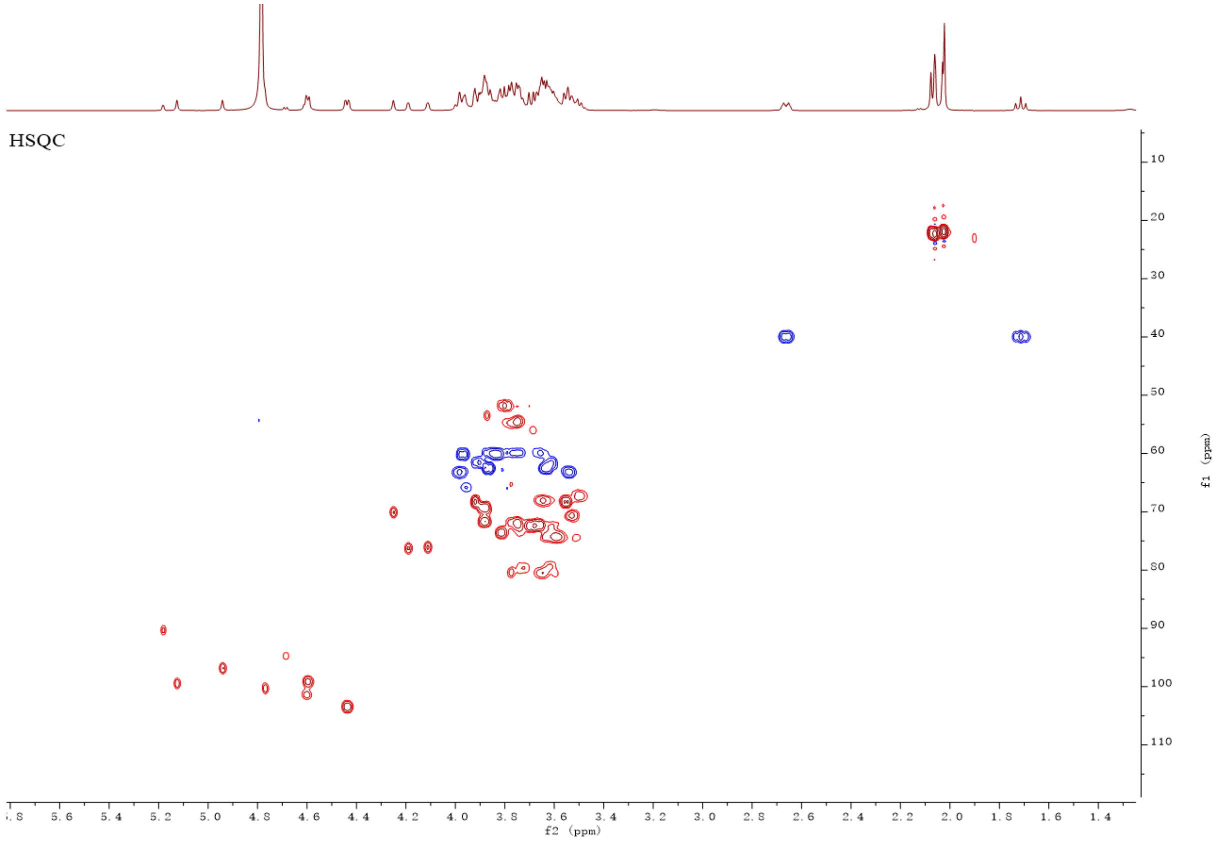
HSQC
f1 (ppm)
f2 (ppm)

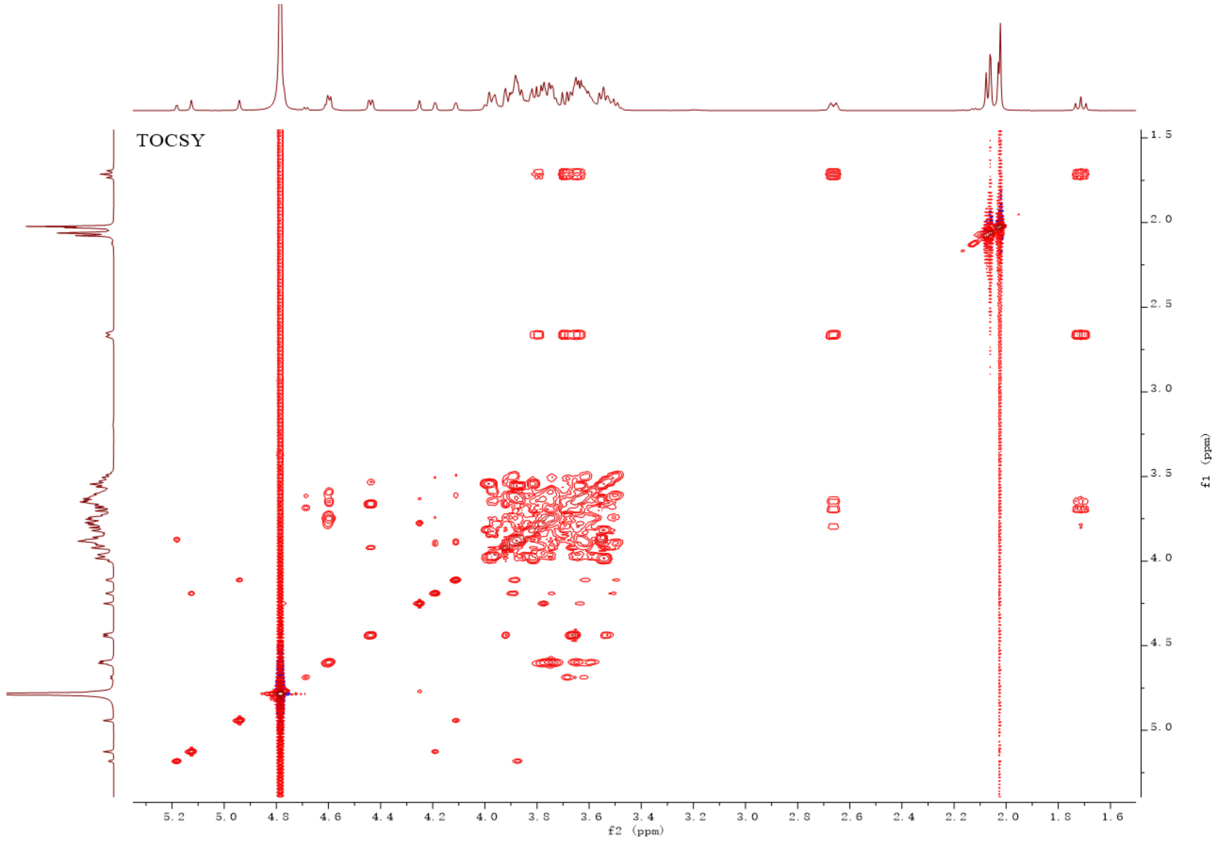
TOCSY
f1 (ppm)
f2 (ppm)

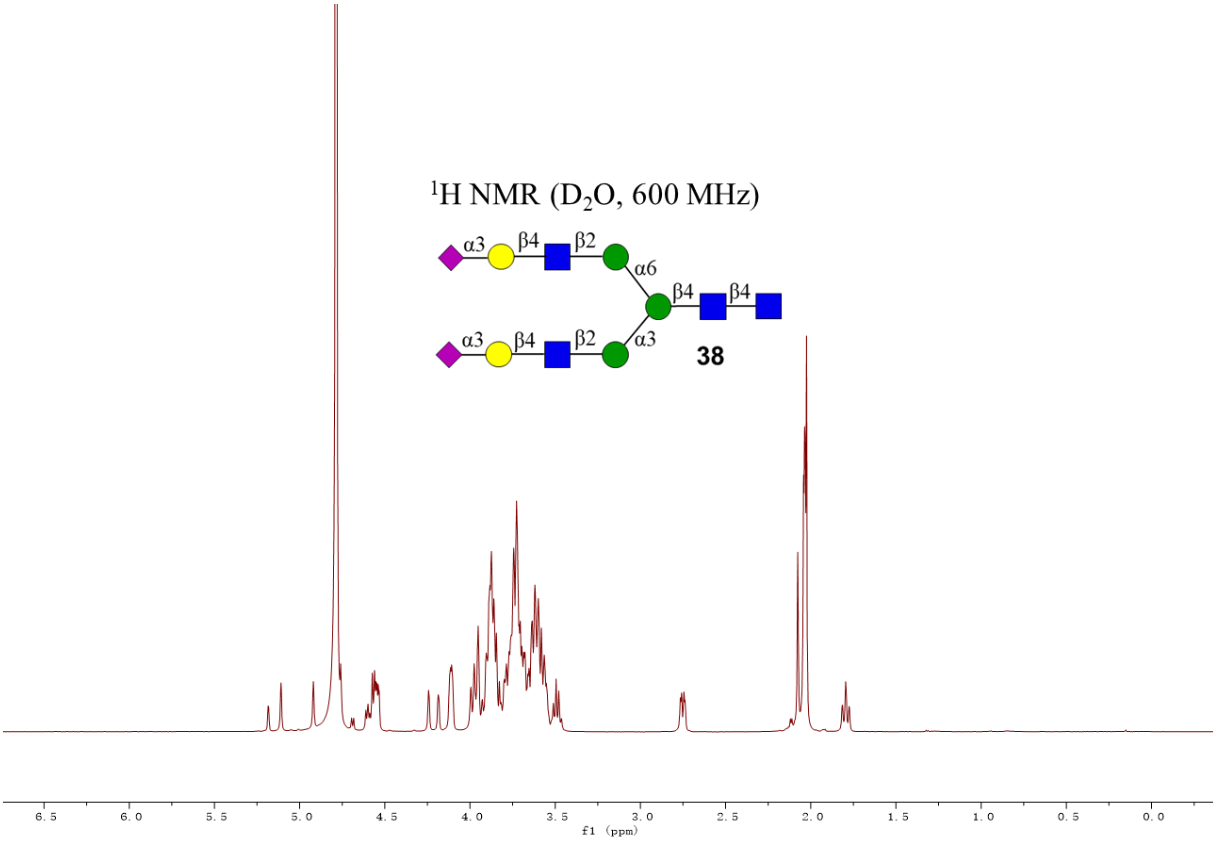

¹H NMR (D₂O, 600 MHz)
α3
β4
β2
α6
β4
β4
α3
β4
β2
α3
38
f1 (ppm)

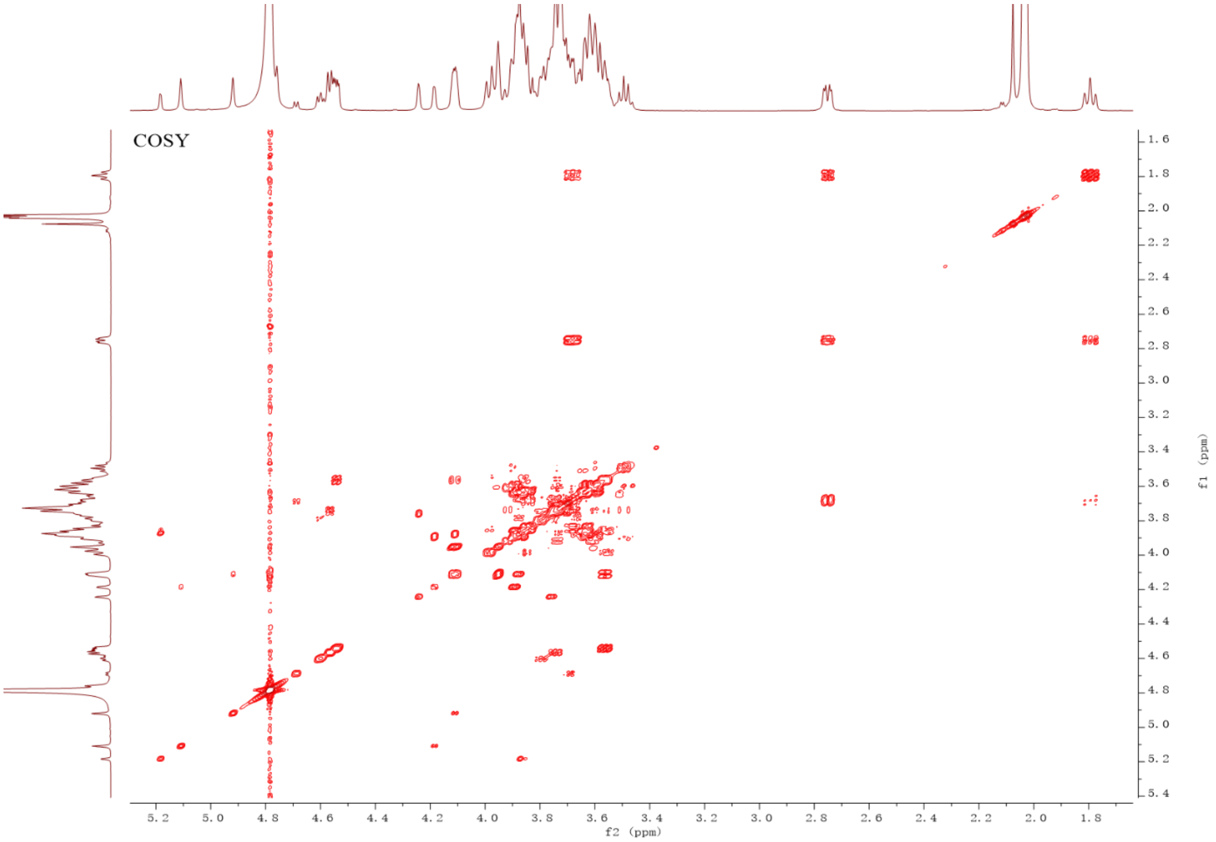

COSY
f2 (ppm)
f1 (ppm)

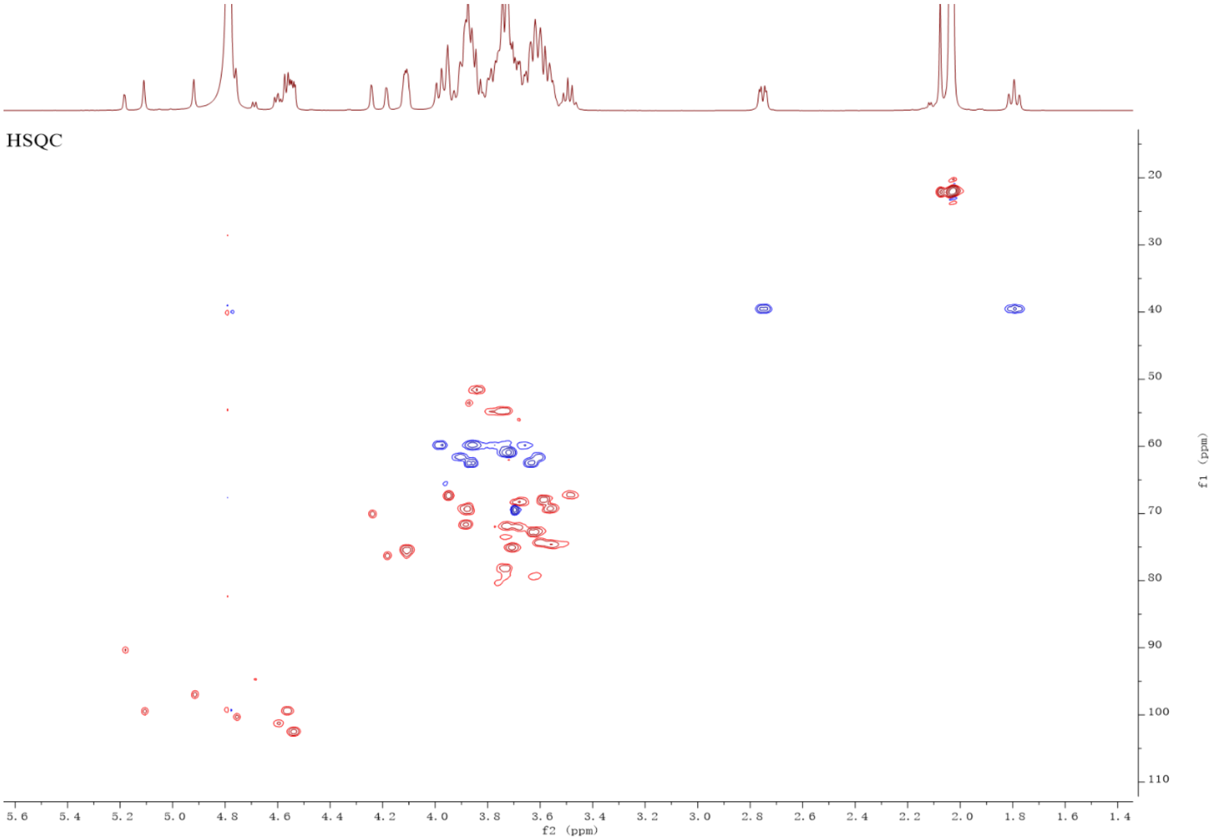
HSQC
f2 (ppm)
f1 (ppm)

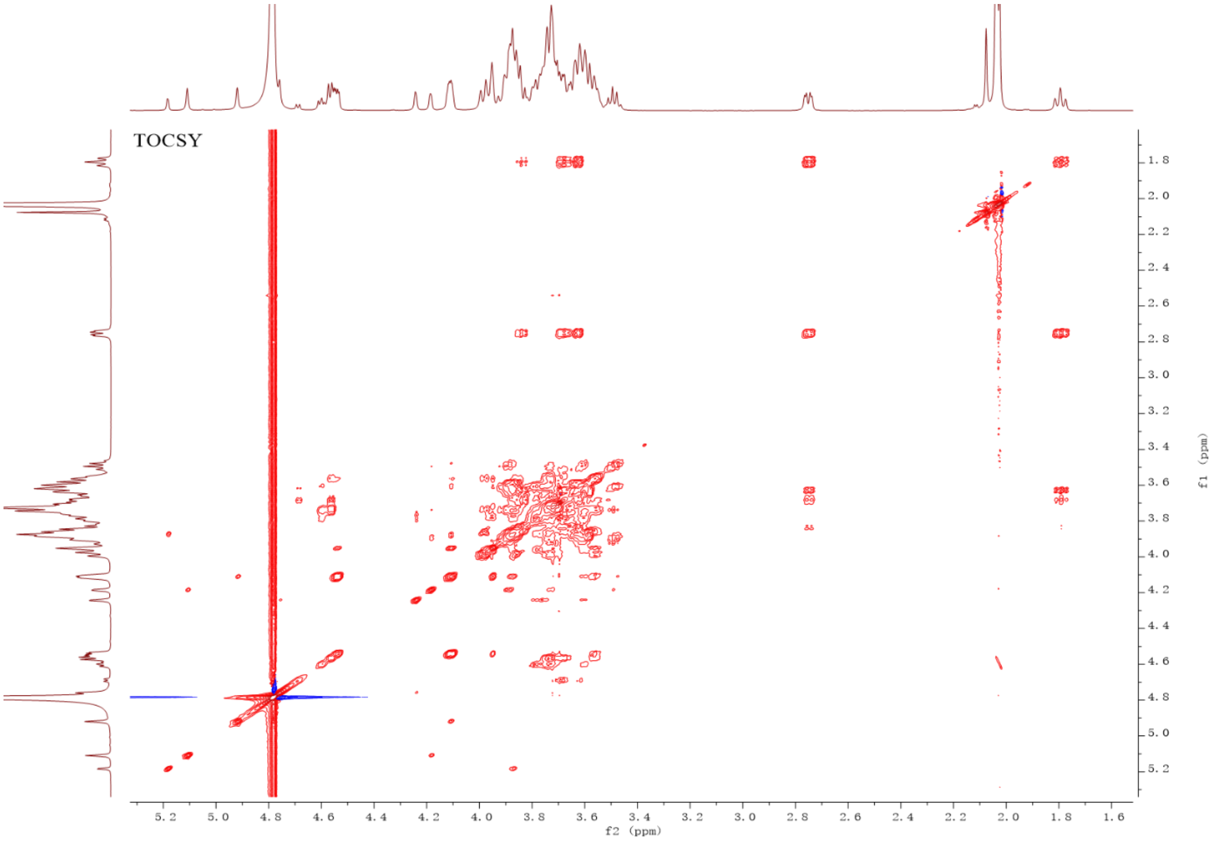
TOCSY
f2 (ppm)
f1 (ppm)

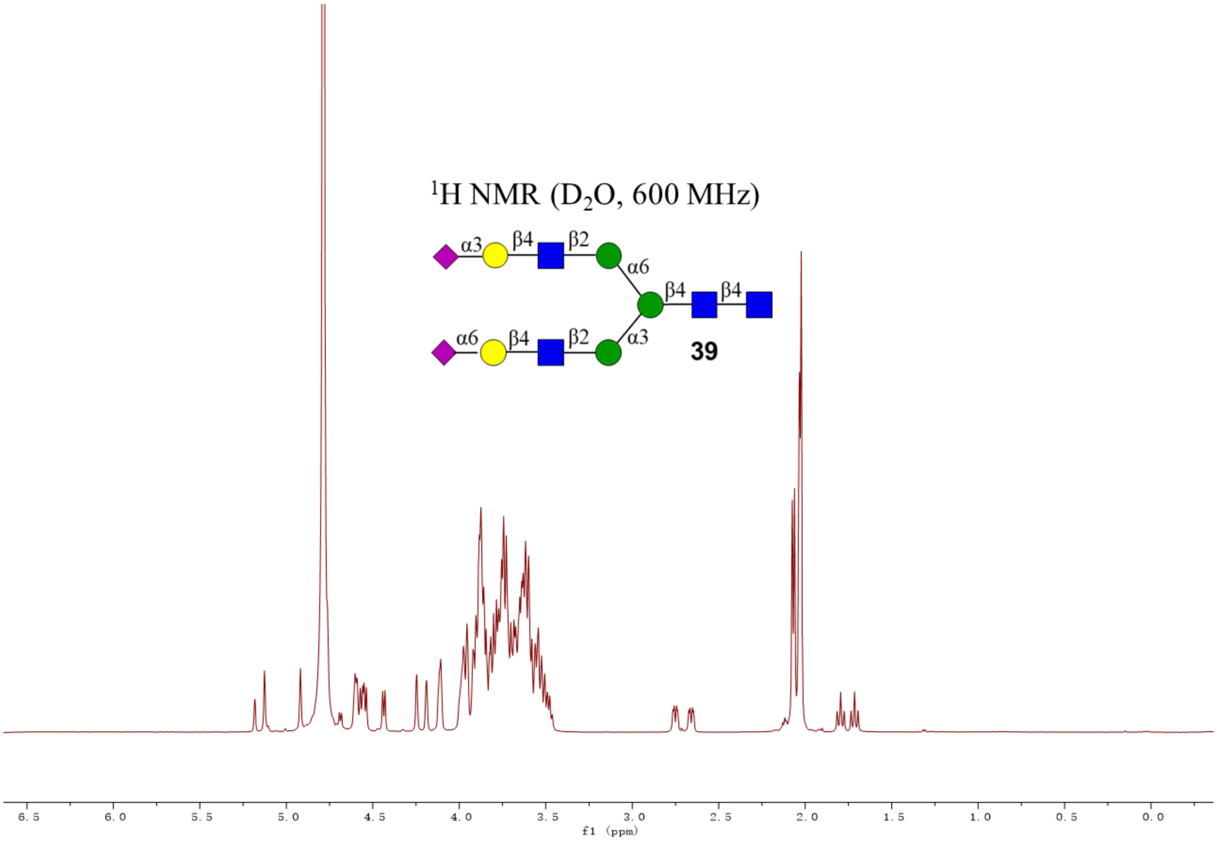
^{1}H NMR (D_2O, 600 MHz)
α3
β4
β2
α6
β4
β4
α6
β4
β2
α3
39
f1 (ppm)

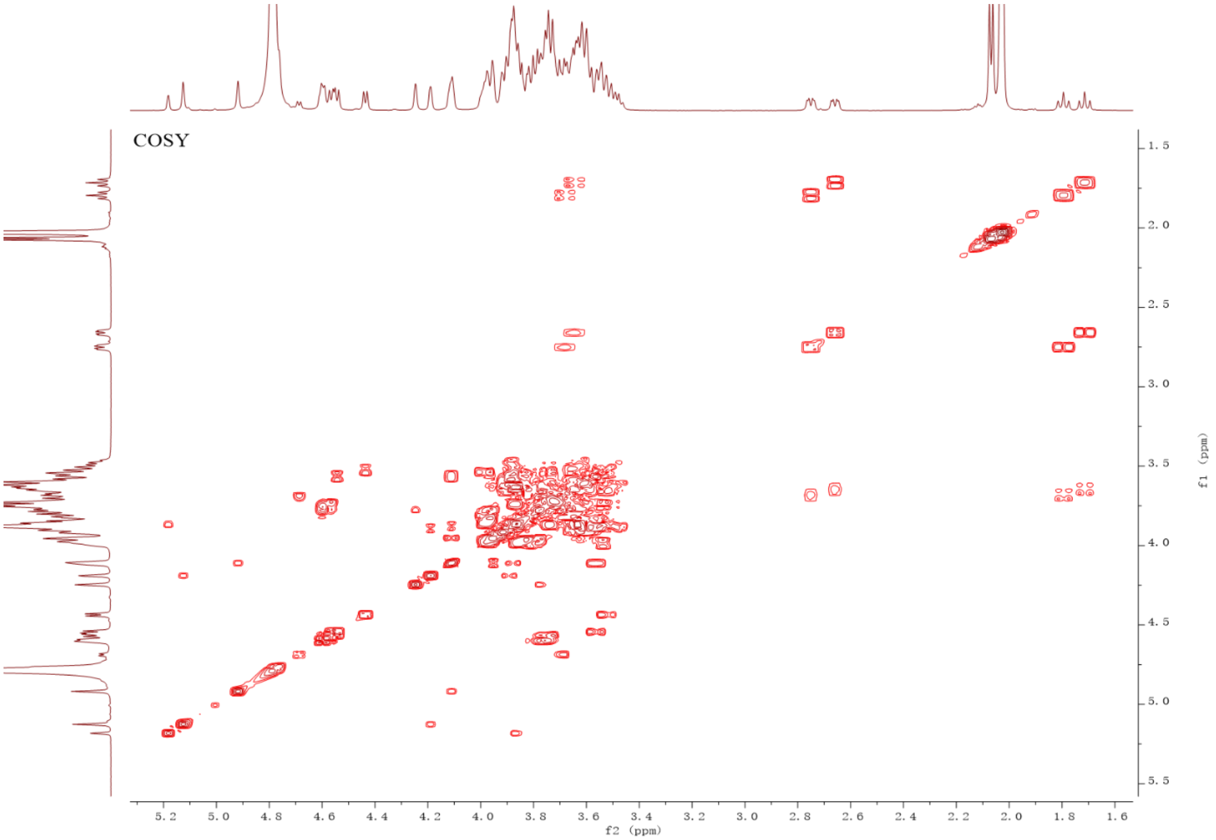
COSY
f2 (ppm)
f1 (ppm)

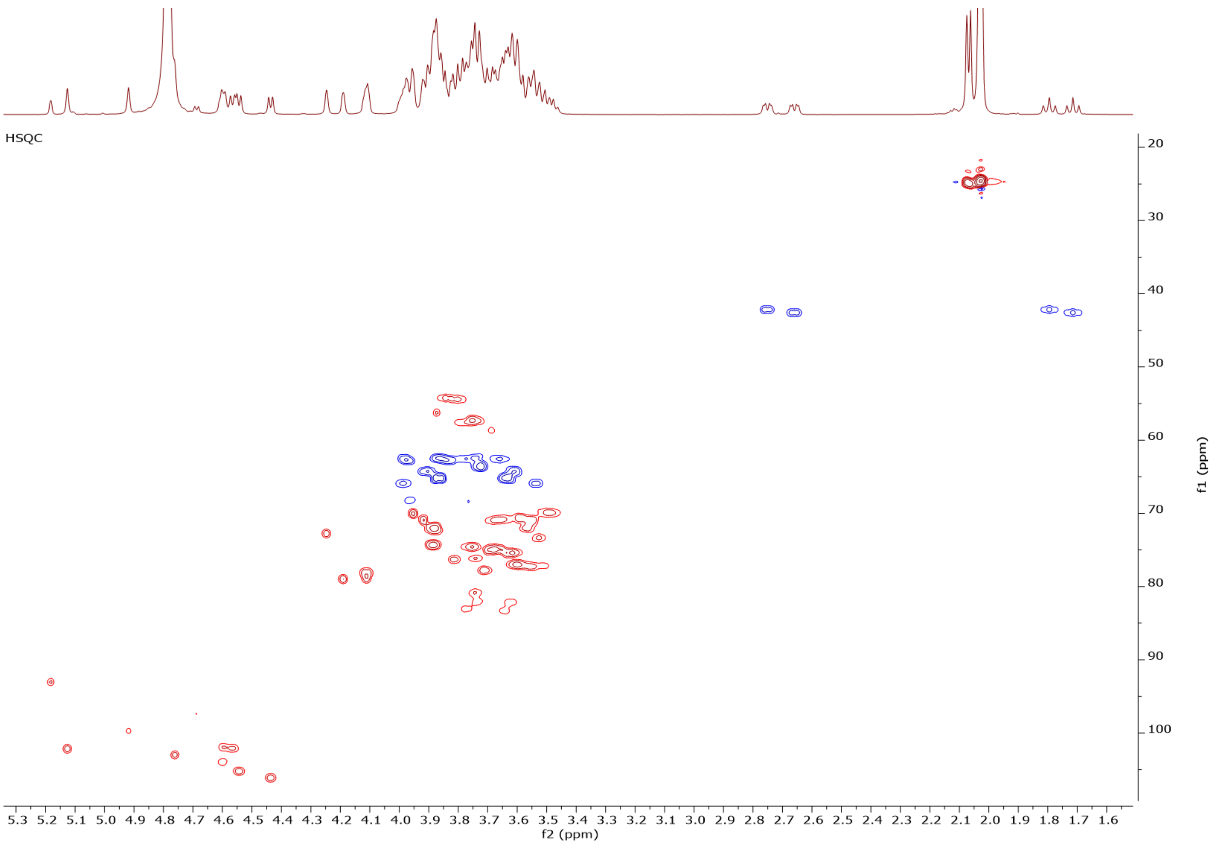
HSQC
f2 (ppm)
f1 (ppm)

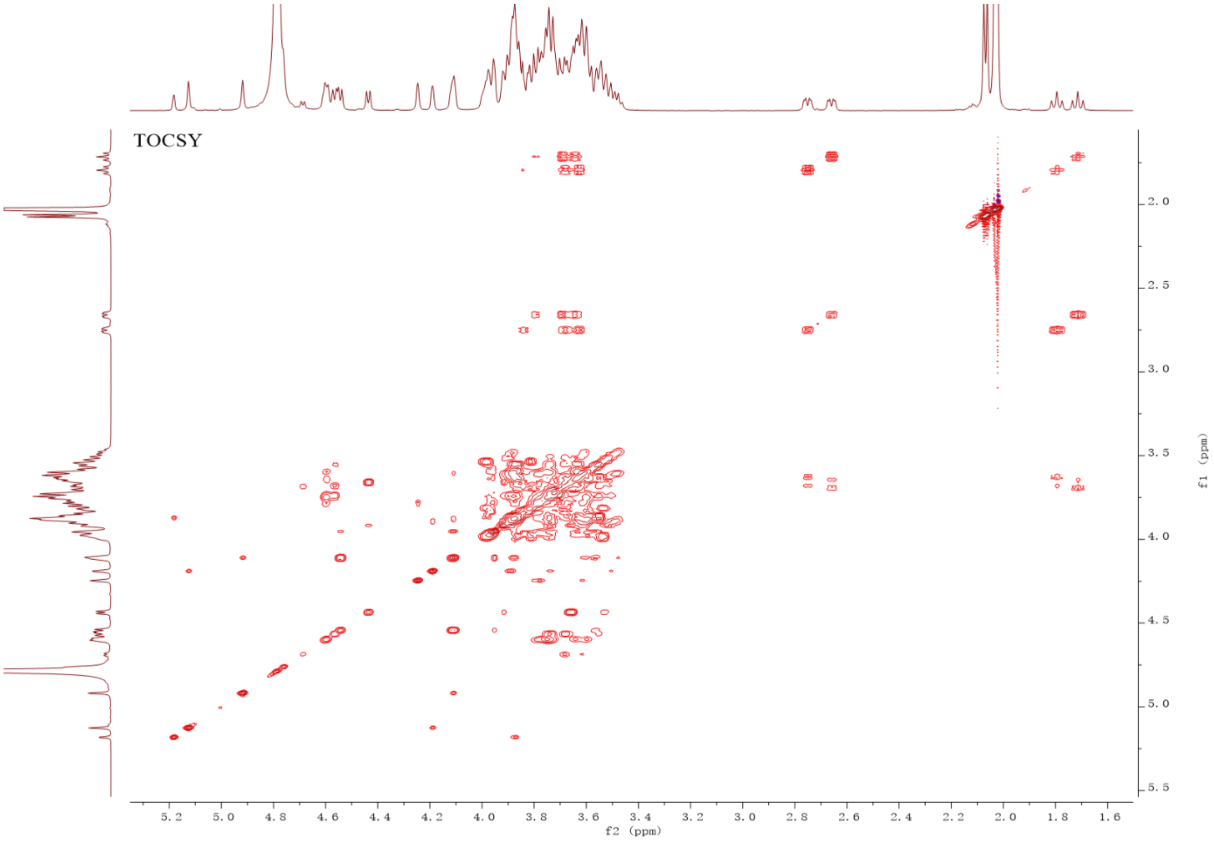
TOCSY
f2 (ppm)
f1 (ppm)

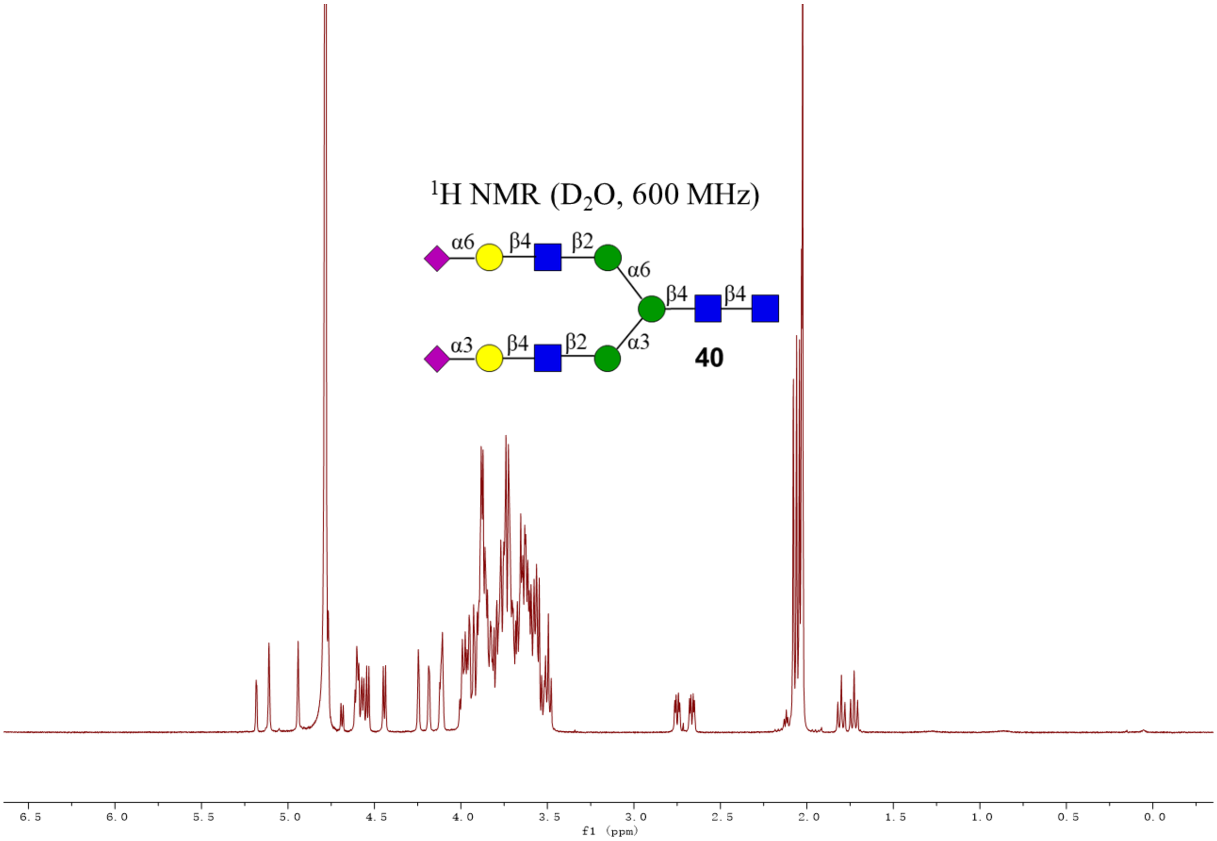
^{1}H NMR (D_2O, 600 MHz)
α6
β4
β2
α6
β4
β4
α3
β4
β2
α3
40
f1 (ppm)

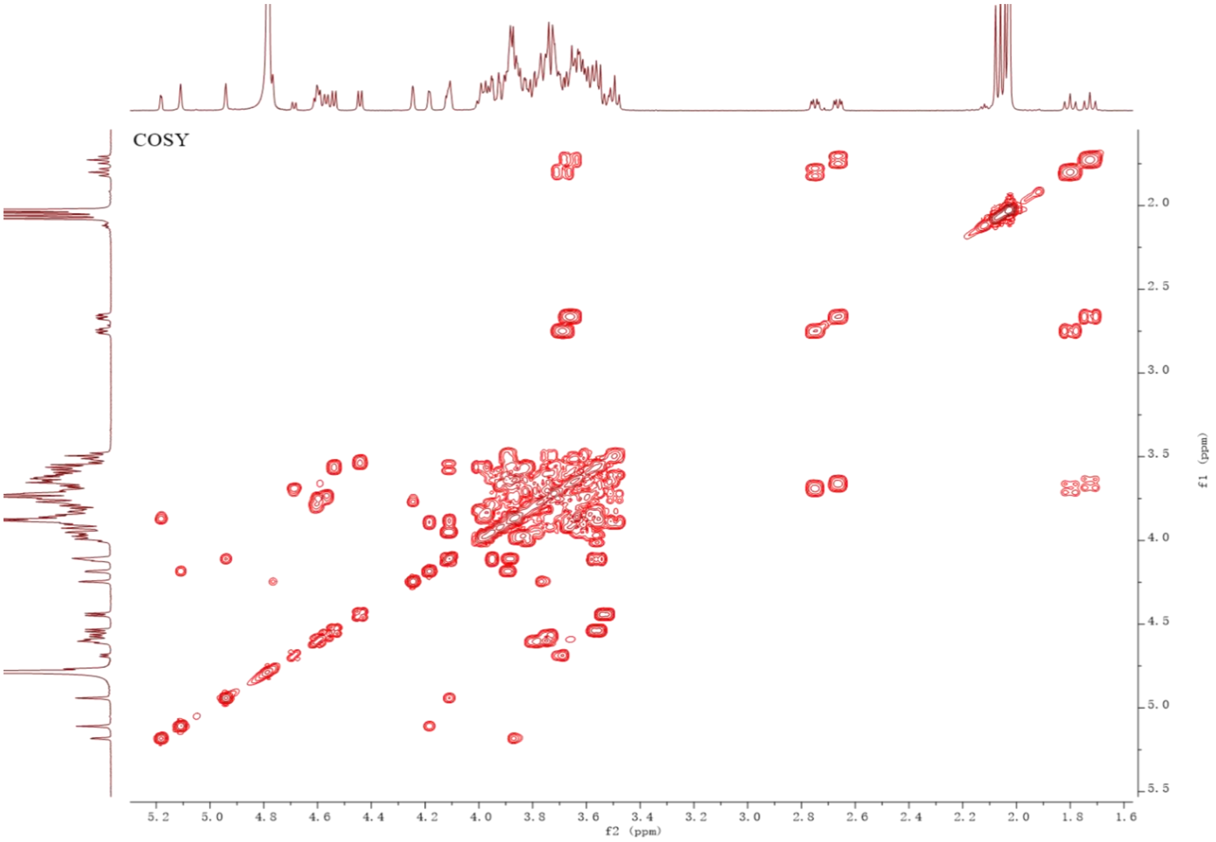
COSY
f2 (ppm)
f1 (ppm)

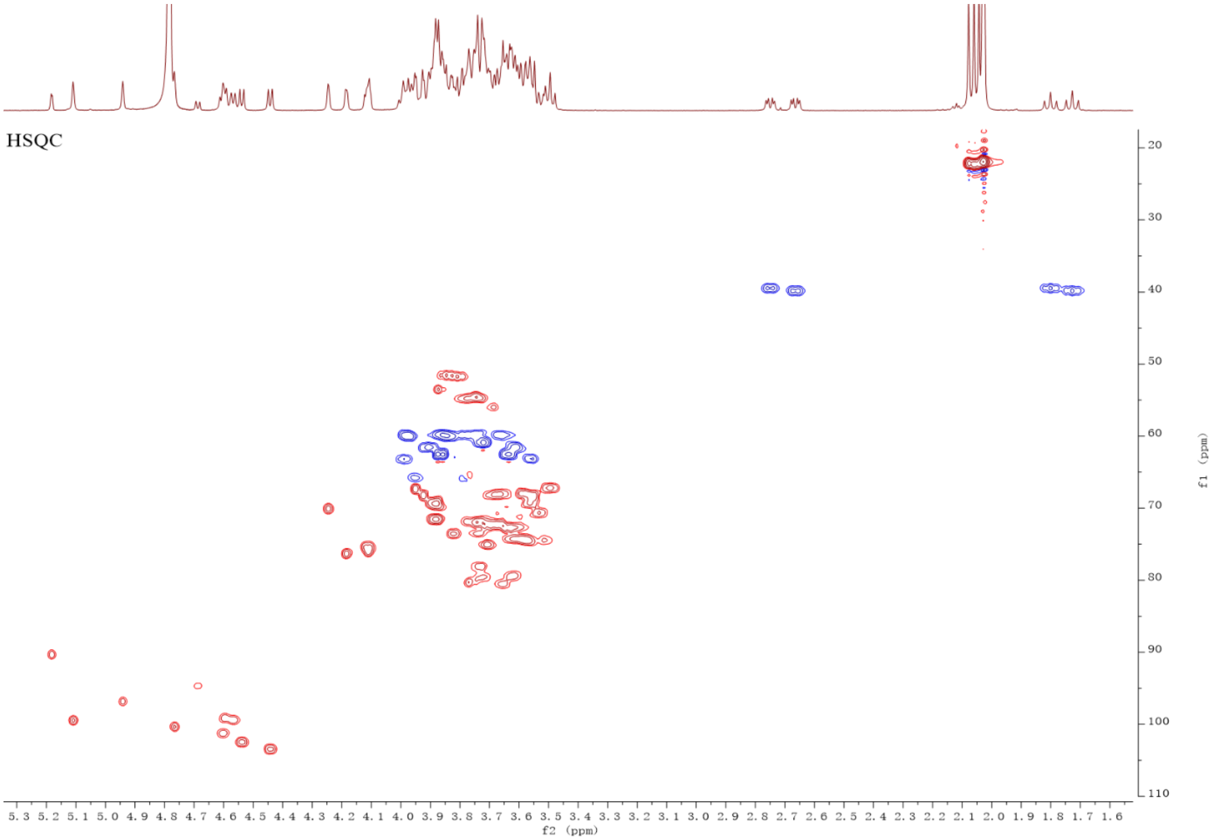
HSQC
f2 (ppm)
f1 (ppm)

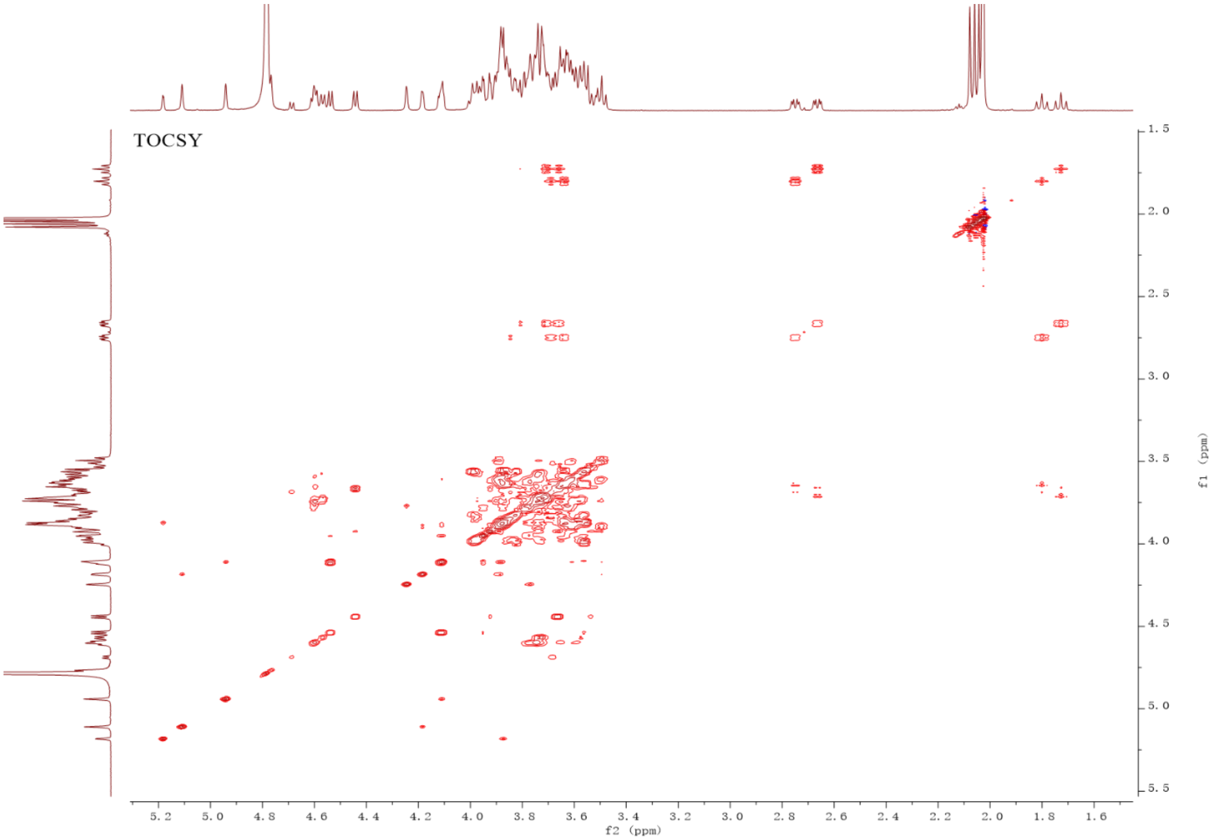
TOCSY
f2 (ppm)
f1 (ppm)

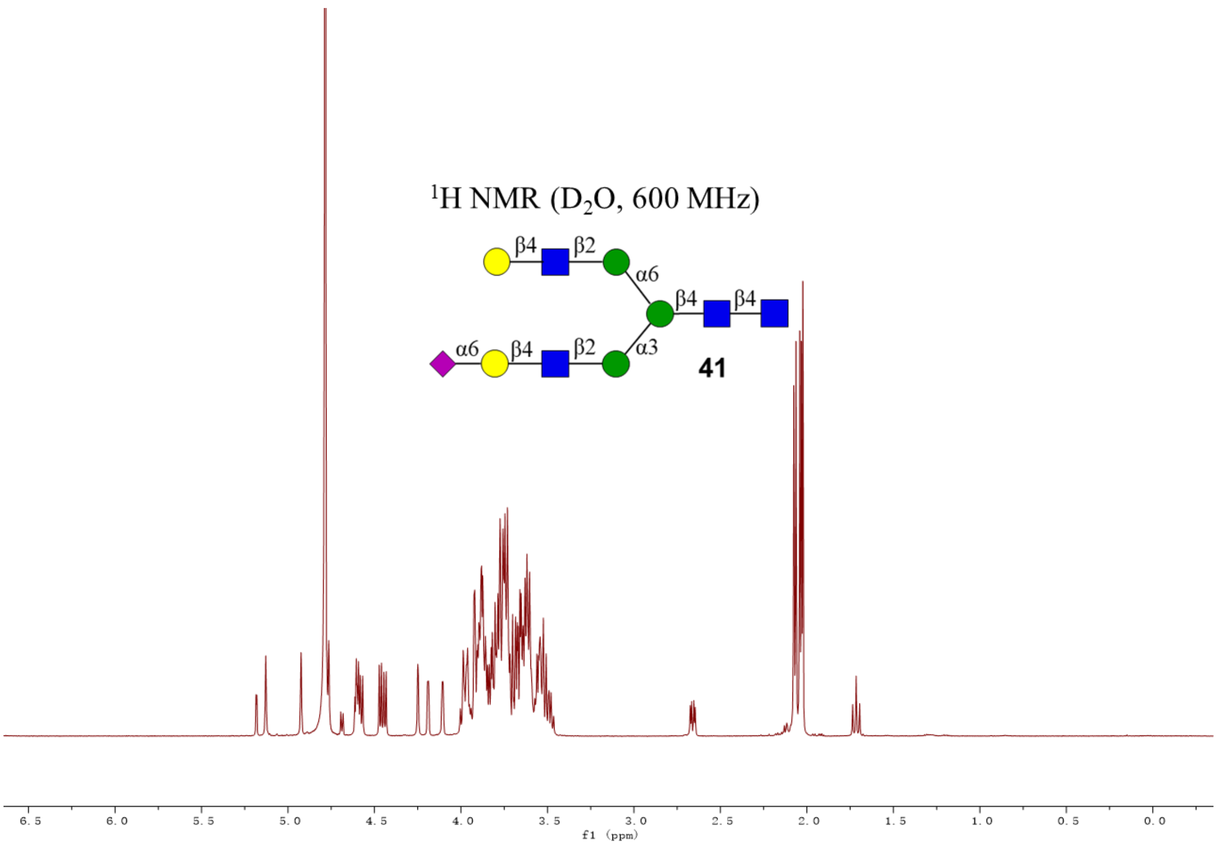

^{1}H NMR (D_2O, 600 MHz)
β4
β2
α6
β4
β4
α6
β4
β2
α3
41
f1 (ppm)

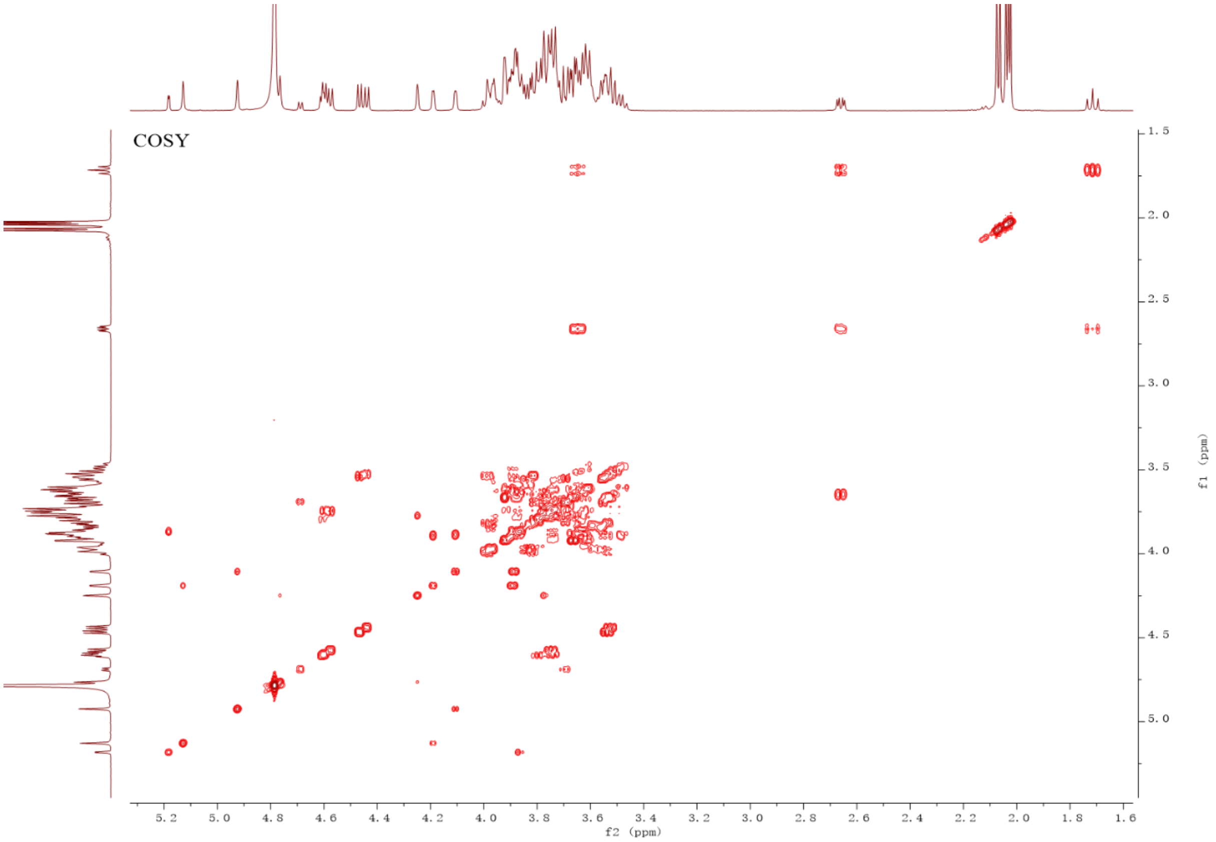

COSY
f2 (ppm)
f1 (ppm)

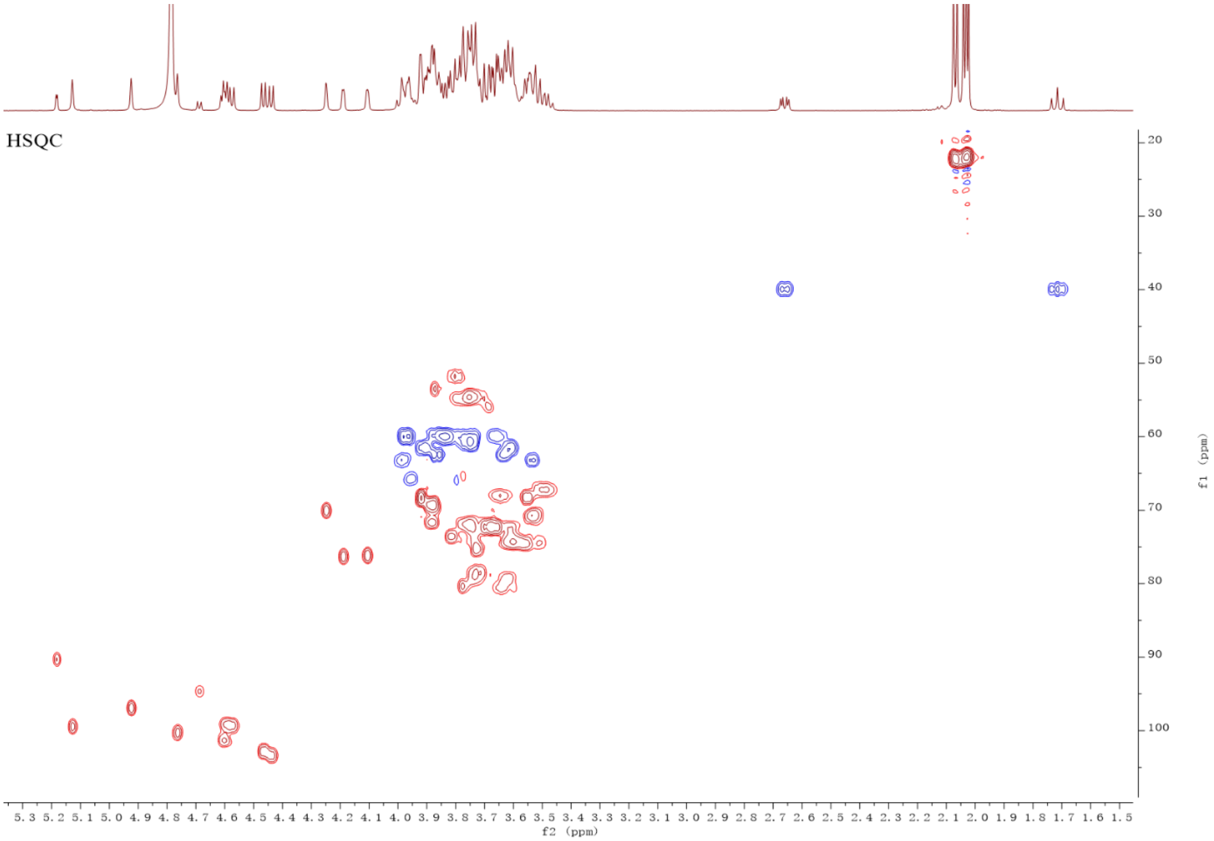
HSQC
f2 (ppm)
f1 (ppm)

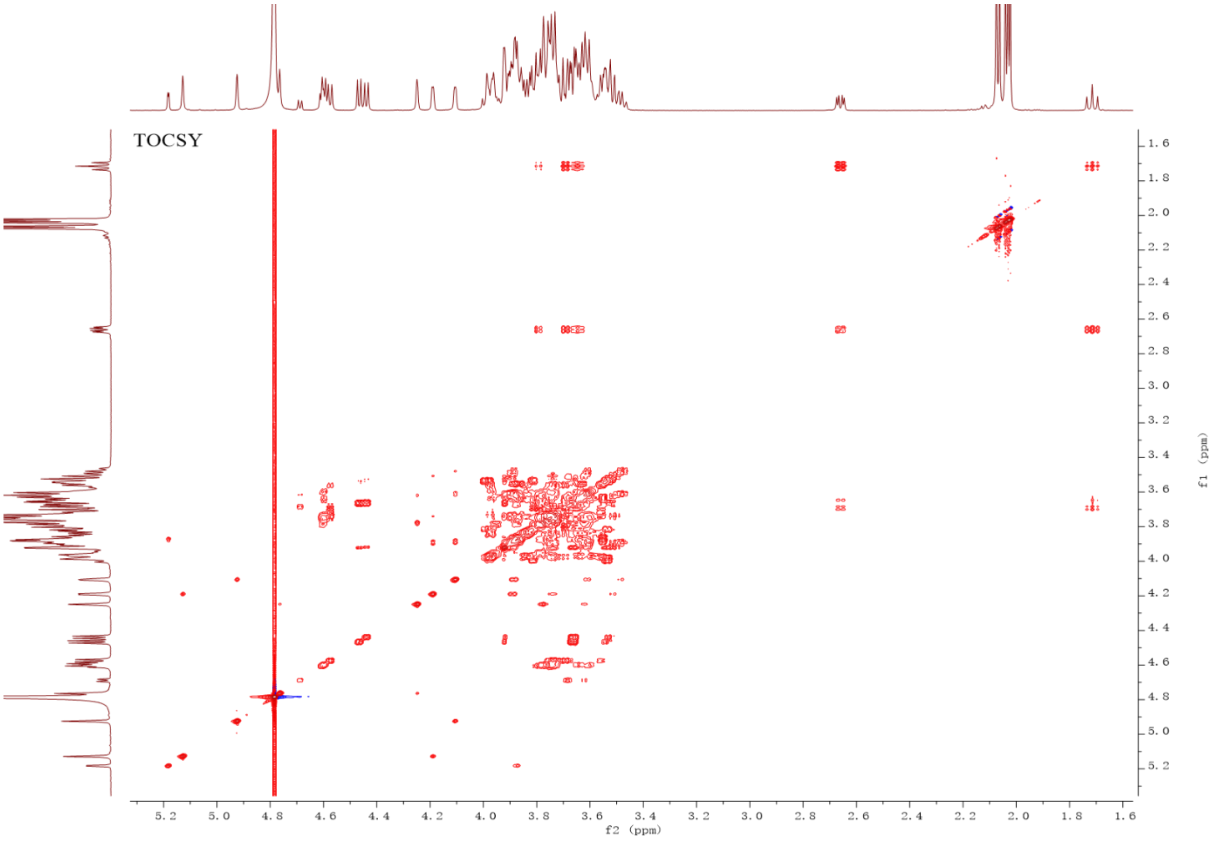
TOCSY
f2 (ppm)
f1 (ppm)

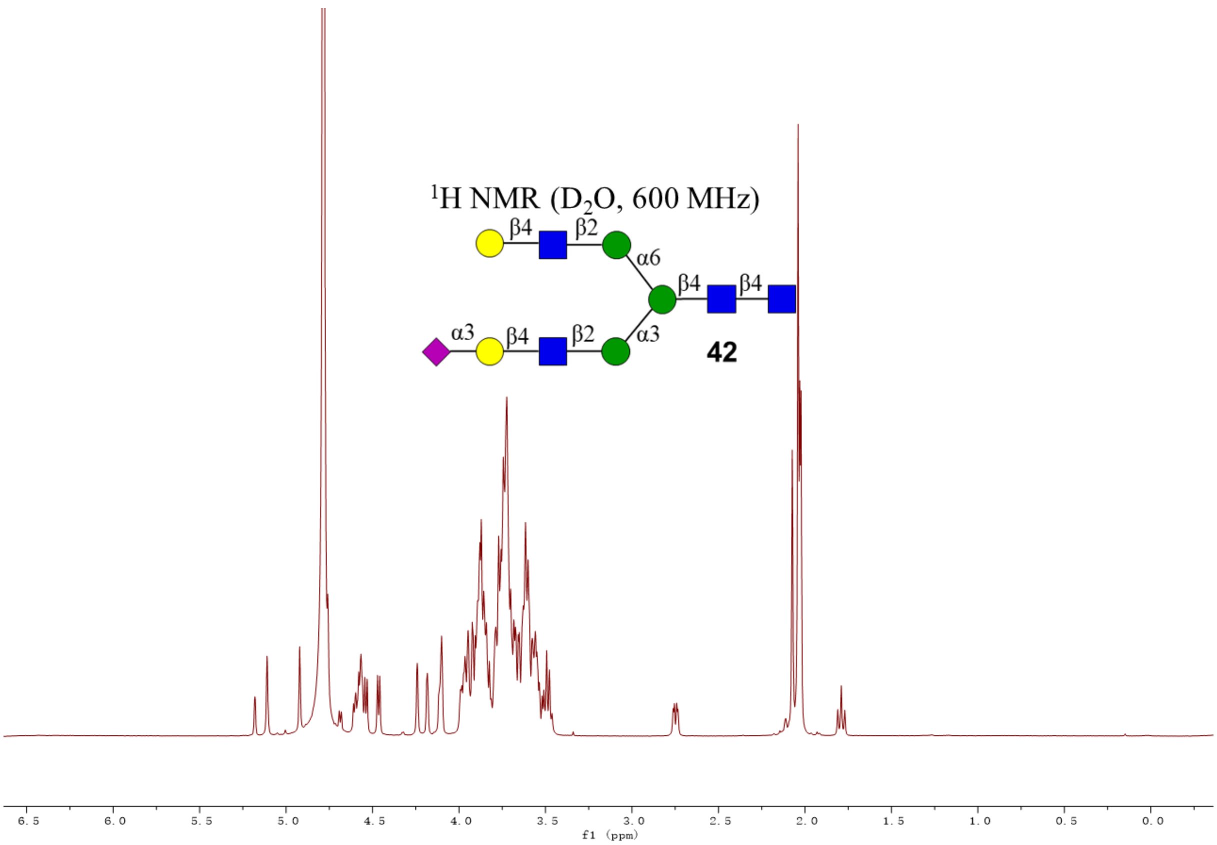

^{1}H NMR (D_2O, 600 MHz)
β4
β2
α6
β4
β4
α3
β4
β2
α3
42
6.5
6.0
5.5
5.0
4.5
4.0
3.5
3.0
2.5
2.0
1.5
1.0
0.5
0.0
f1 (ppm)

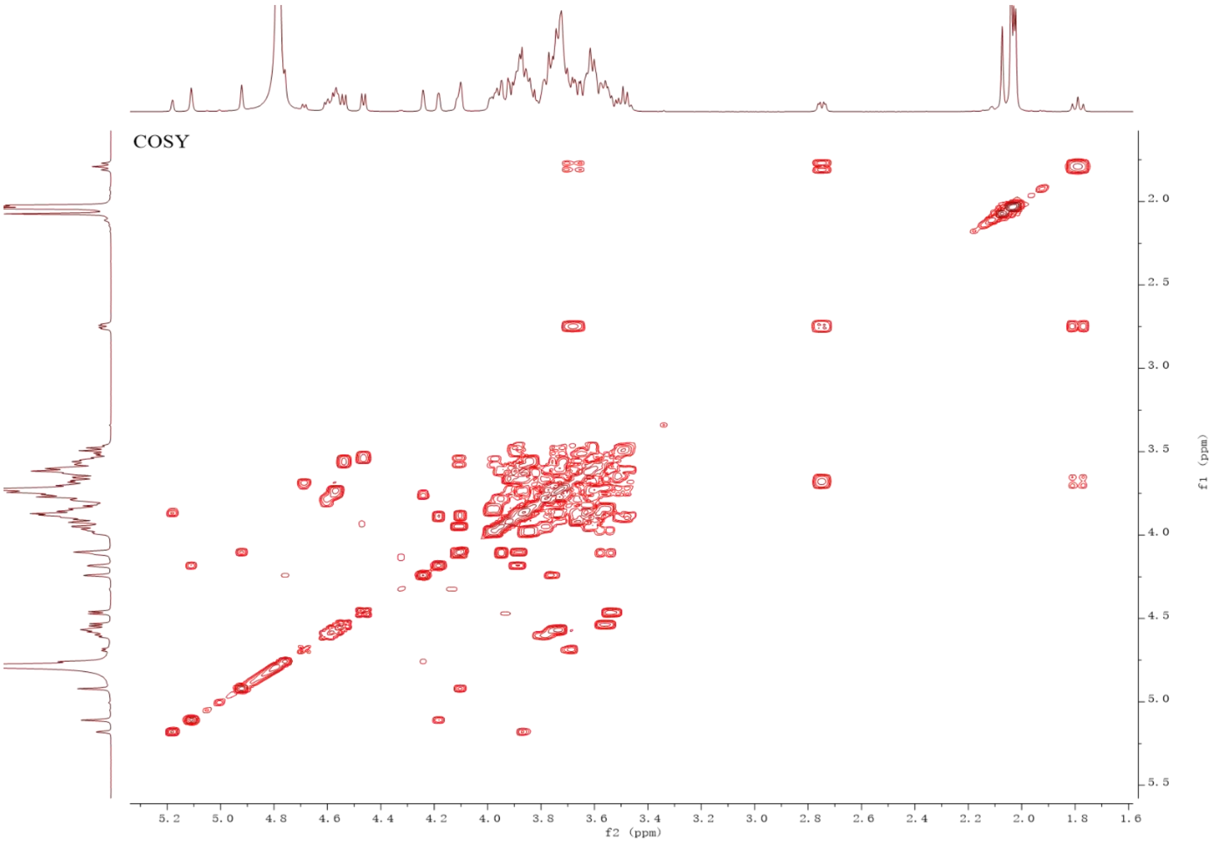

COSY
5.2
5.0
4.8
4.6
4.4
4.2
4.0
3.8
3.6
3.4
3.2
3.0
2.8
2.6
2.4
2.2
2.0
1.8
1.6
f2 (ppm)
f1 (ppm)

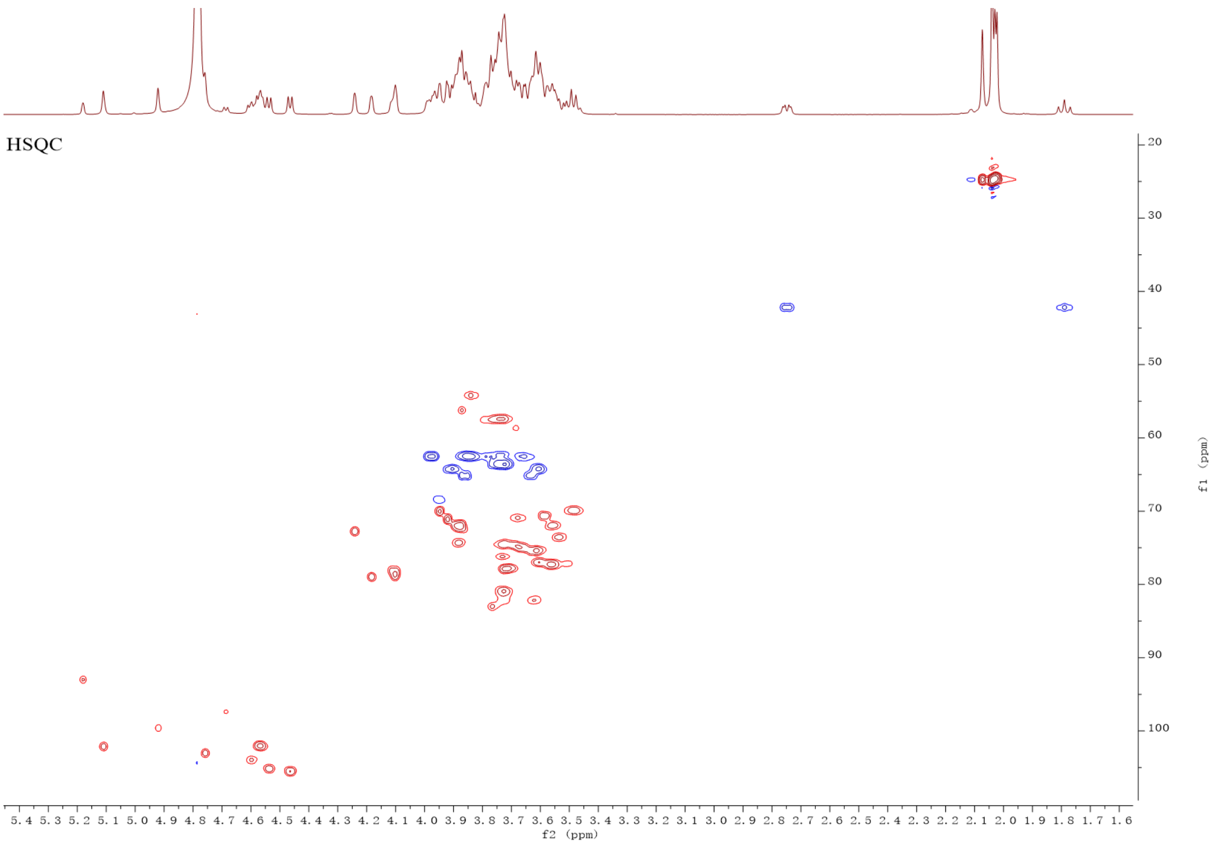
HSQC
f2 (ppm)
f1 (ppm)

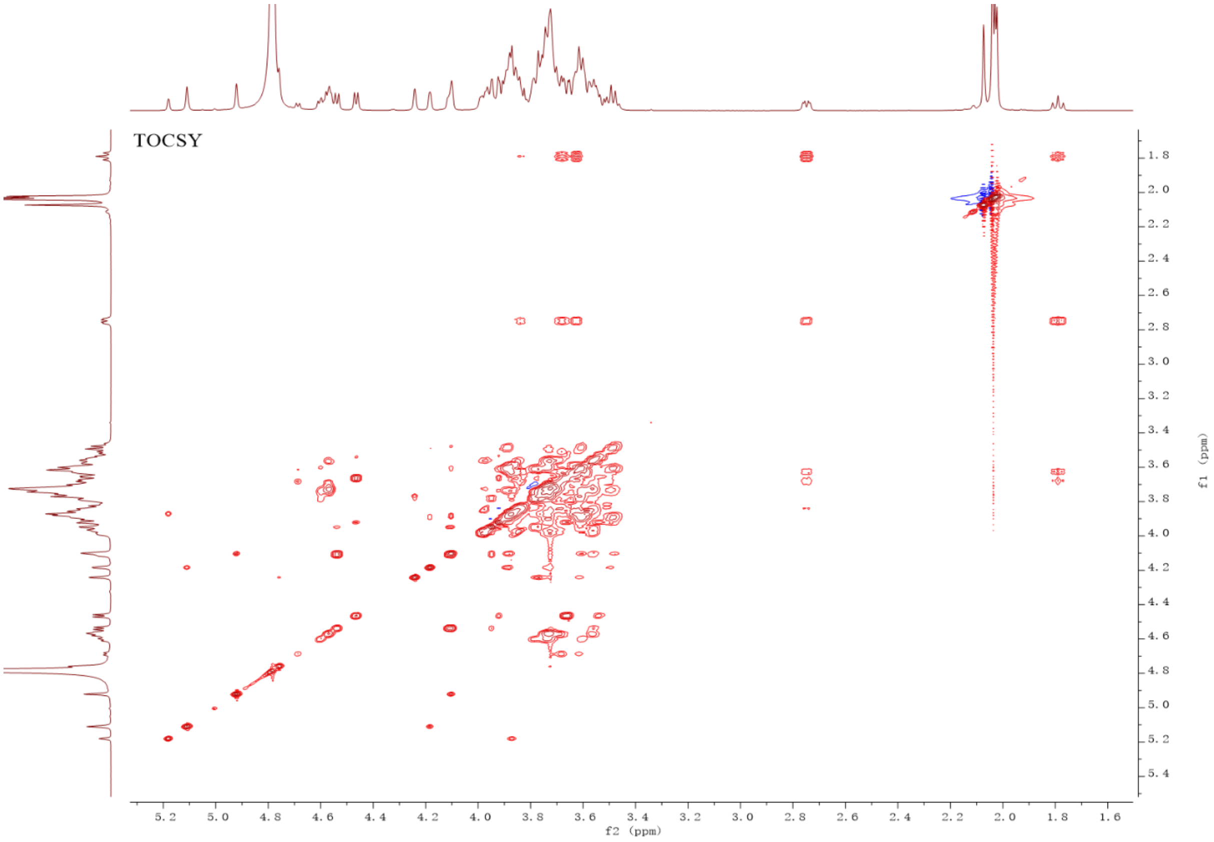
TOCSY
f2 (ppm)
f1 (ppm)

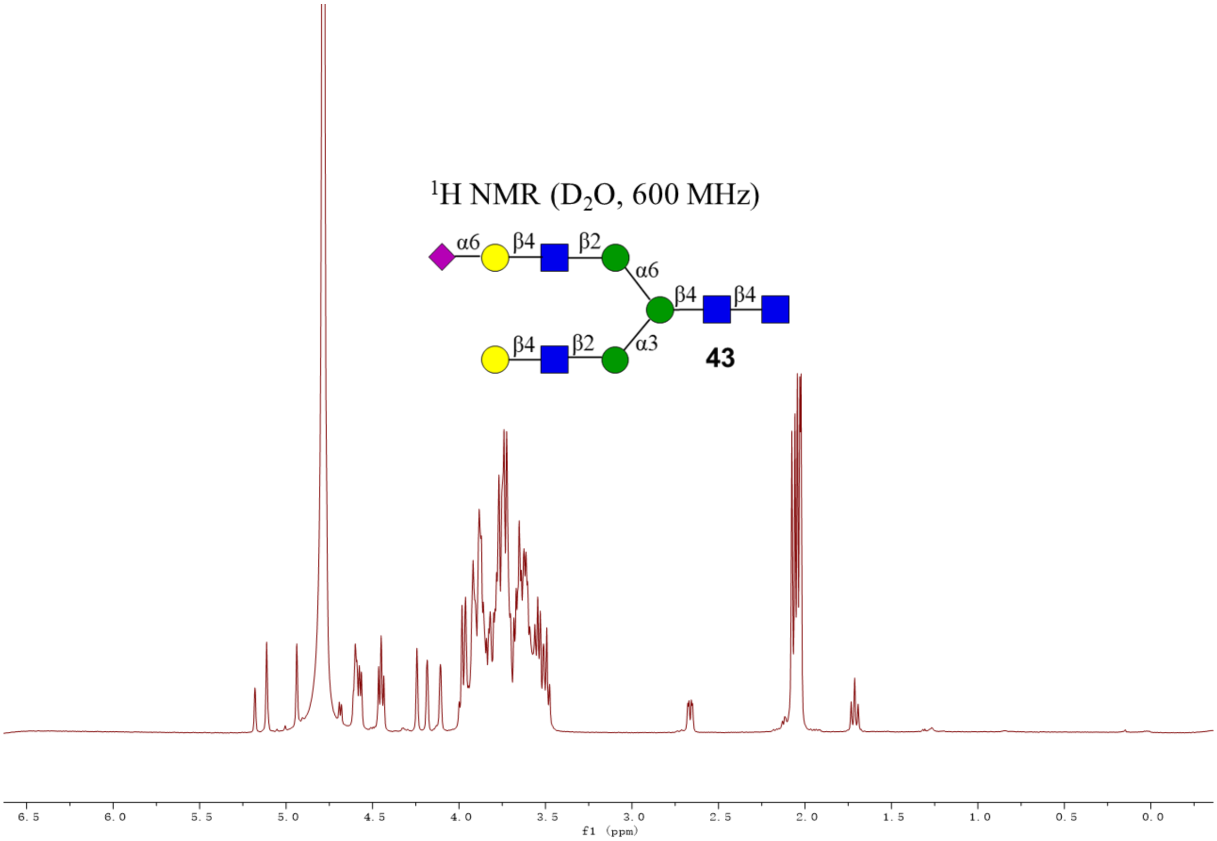

^{1}H NMR (D2O, 600 MHz)
α6
β4
β2
α6
β4
β4
β4
β2
α3
43
f1 (ppm)

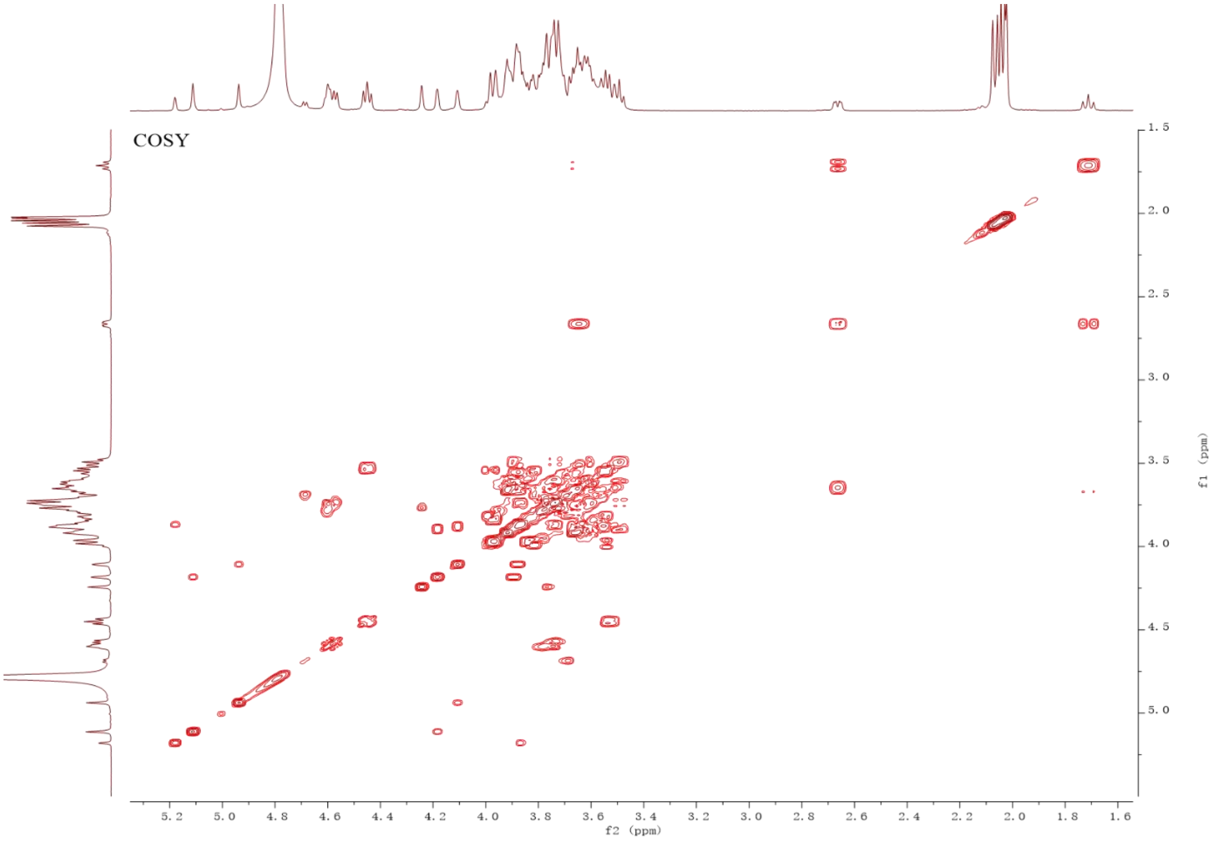

COSY
f2 (ppm)
f1 (ppm)

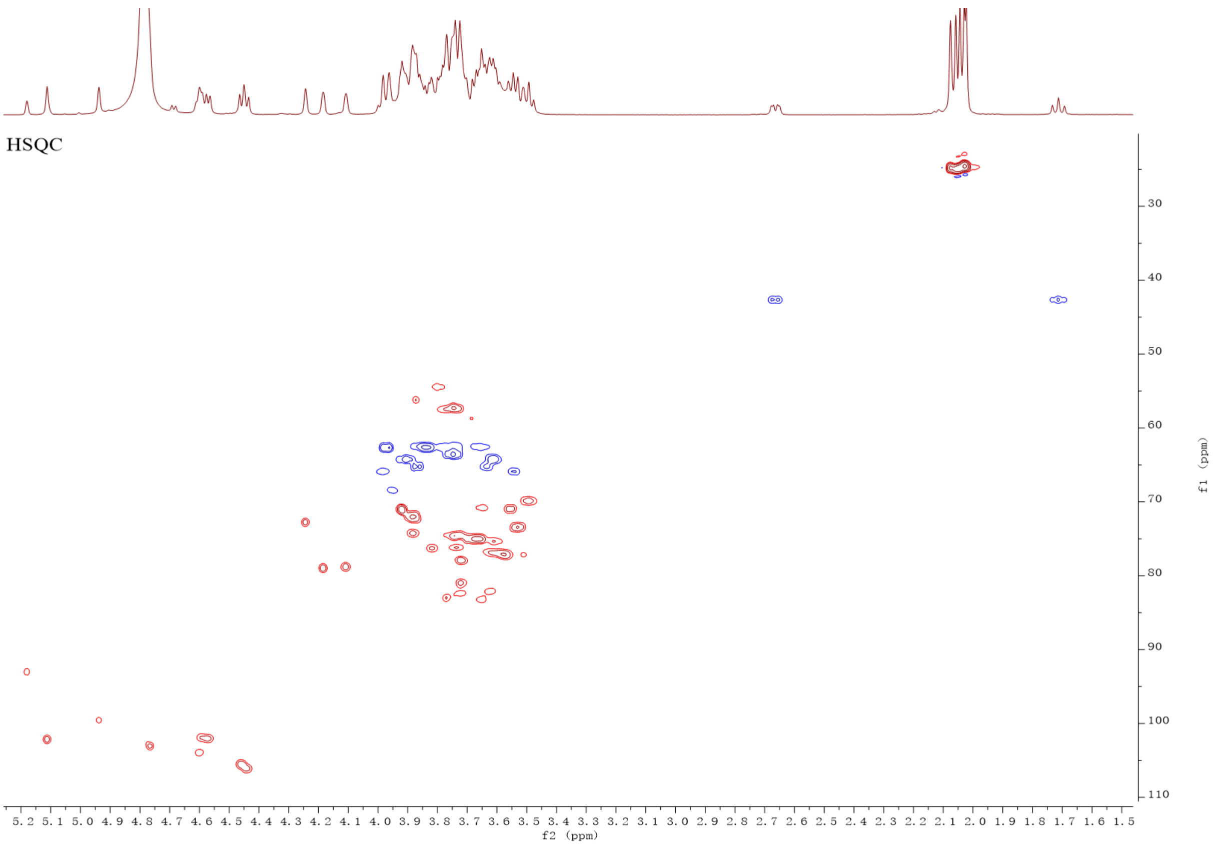
HSQC
f2 (ppm)
f1 (ppm)

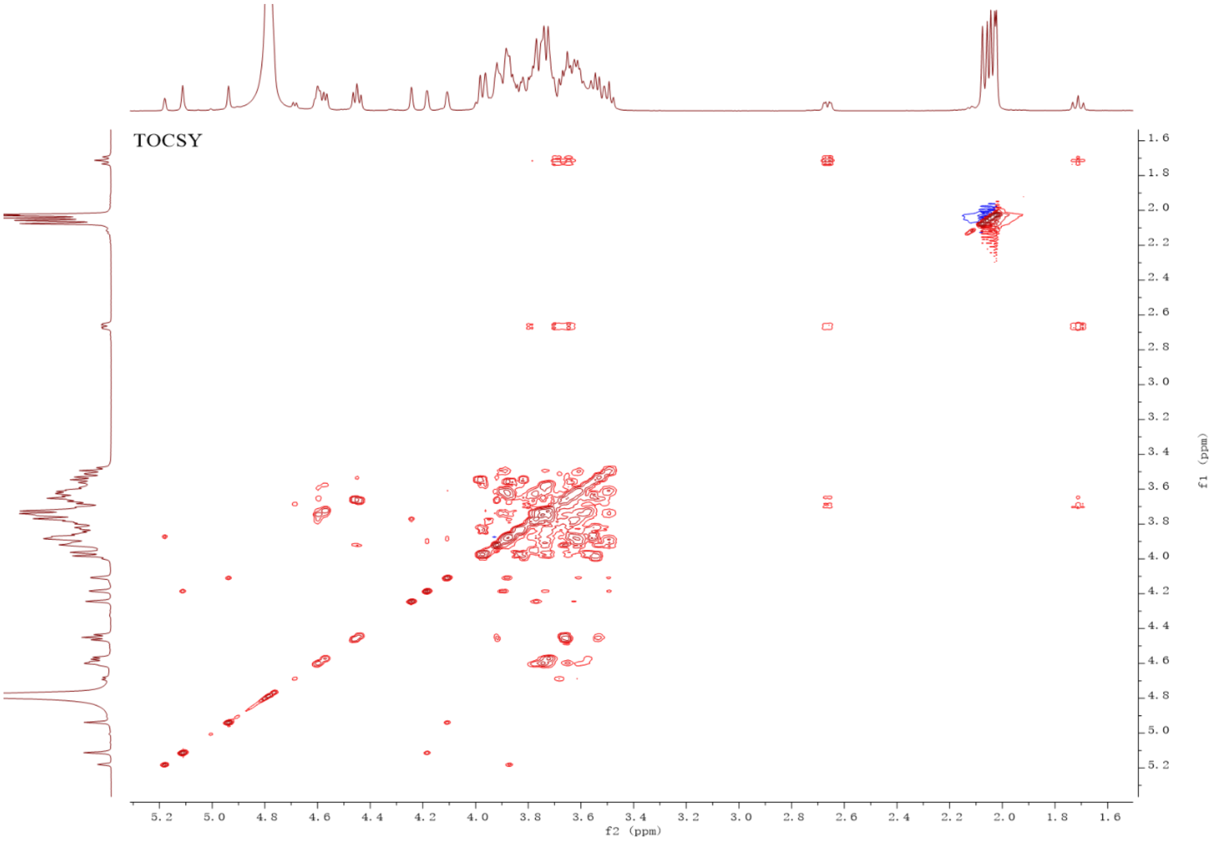
TOCSY
f2 (ppm)
f1 (ppm)

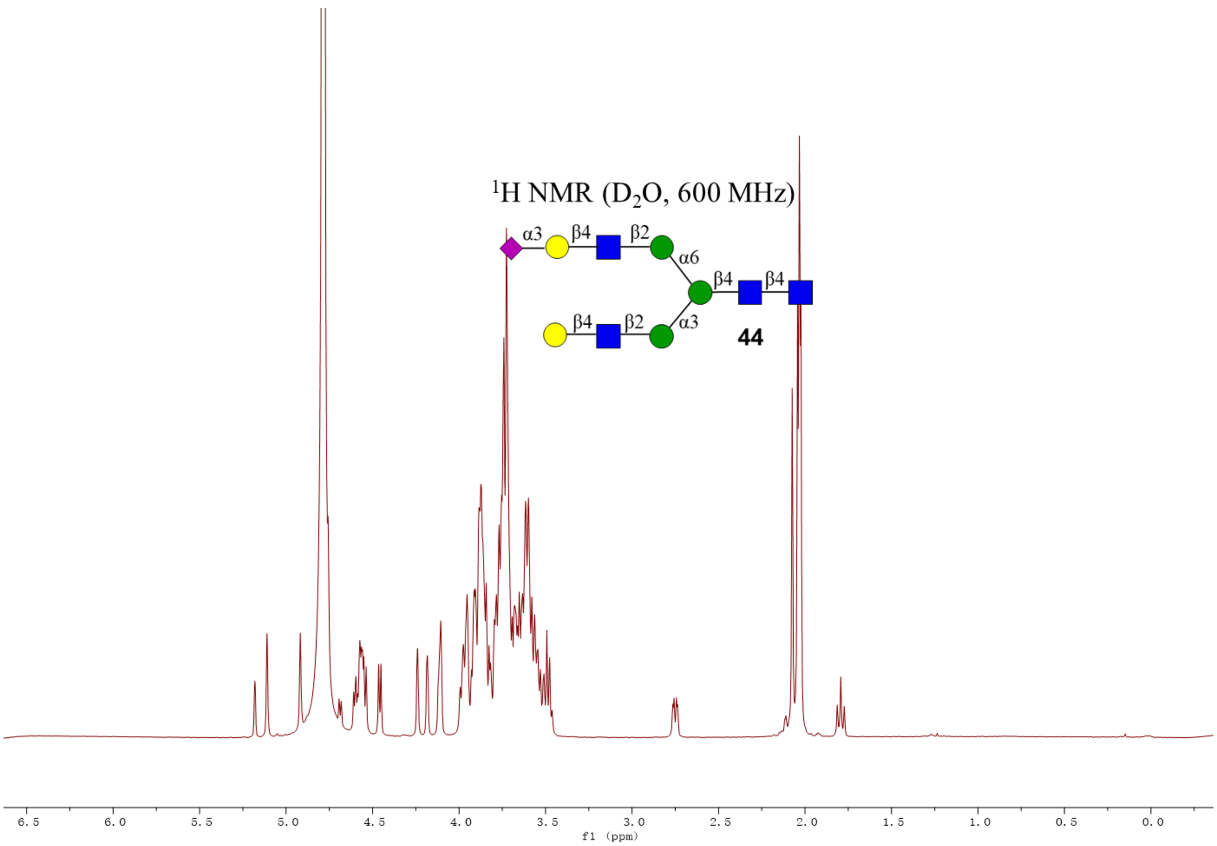

^{1}H NMR (D_2O, 600 MHz)
α3
β4
β2
α6
β4
β4
β4
β2
α3
44
f1 (ppm)

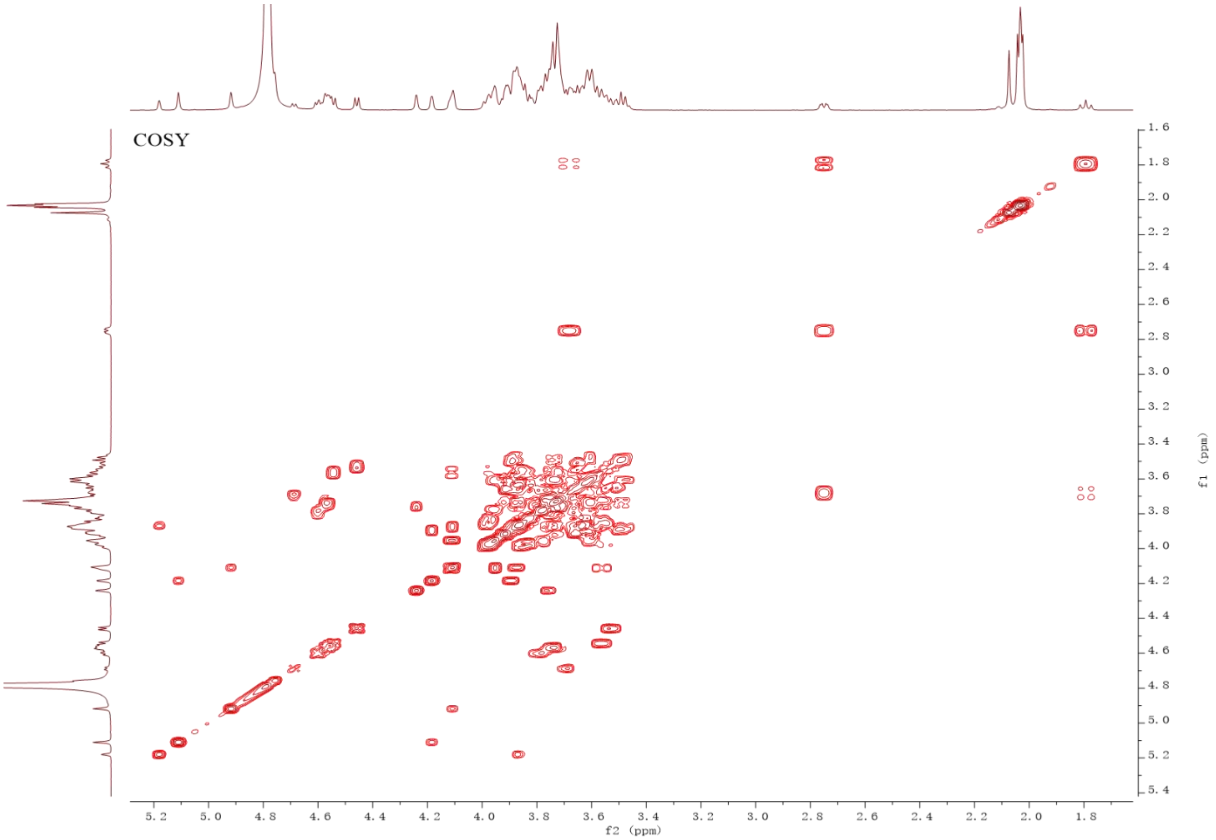

COSY
f2 (ppm)
f1 (ppm)

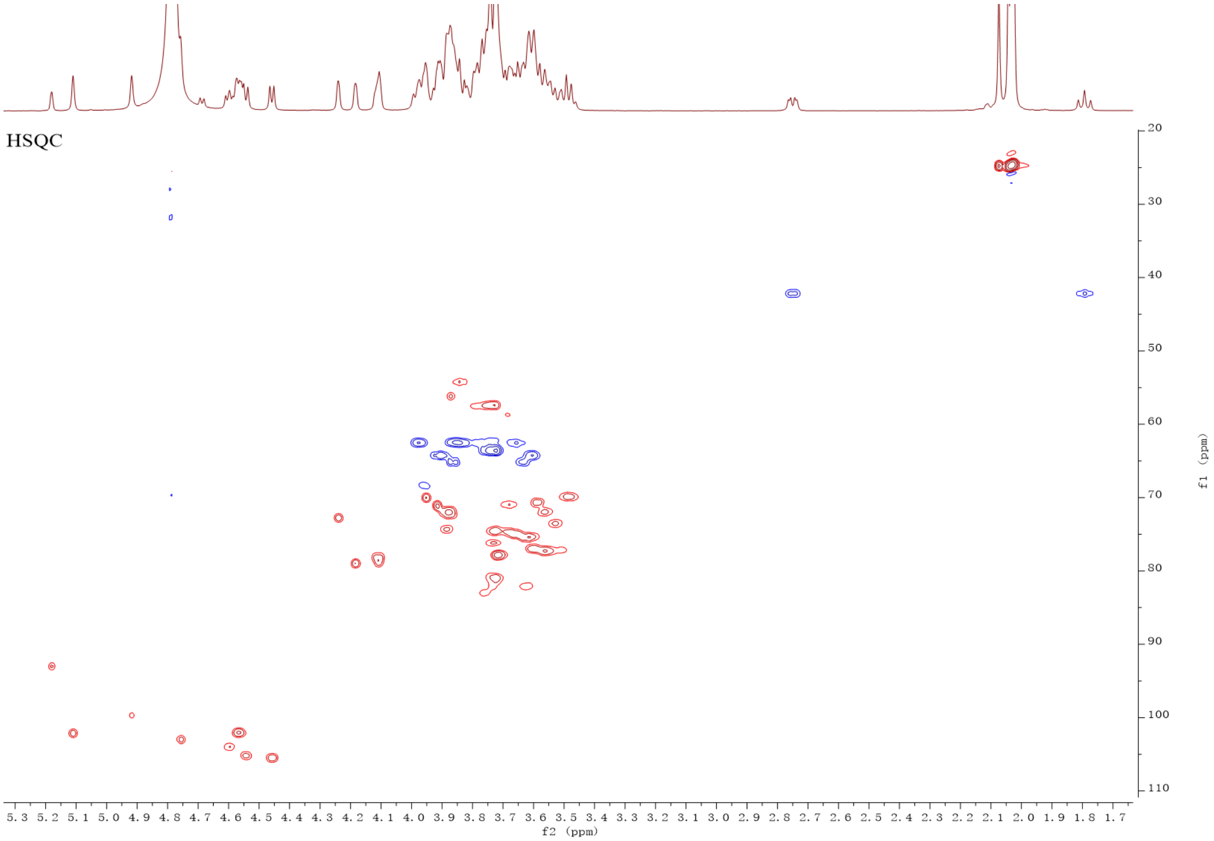
HSQC
f2 (ppm)
f1 (ppm)

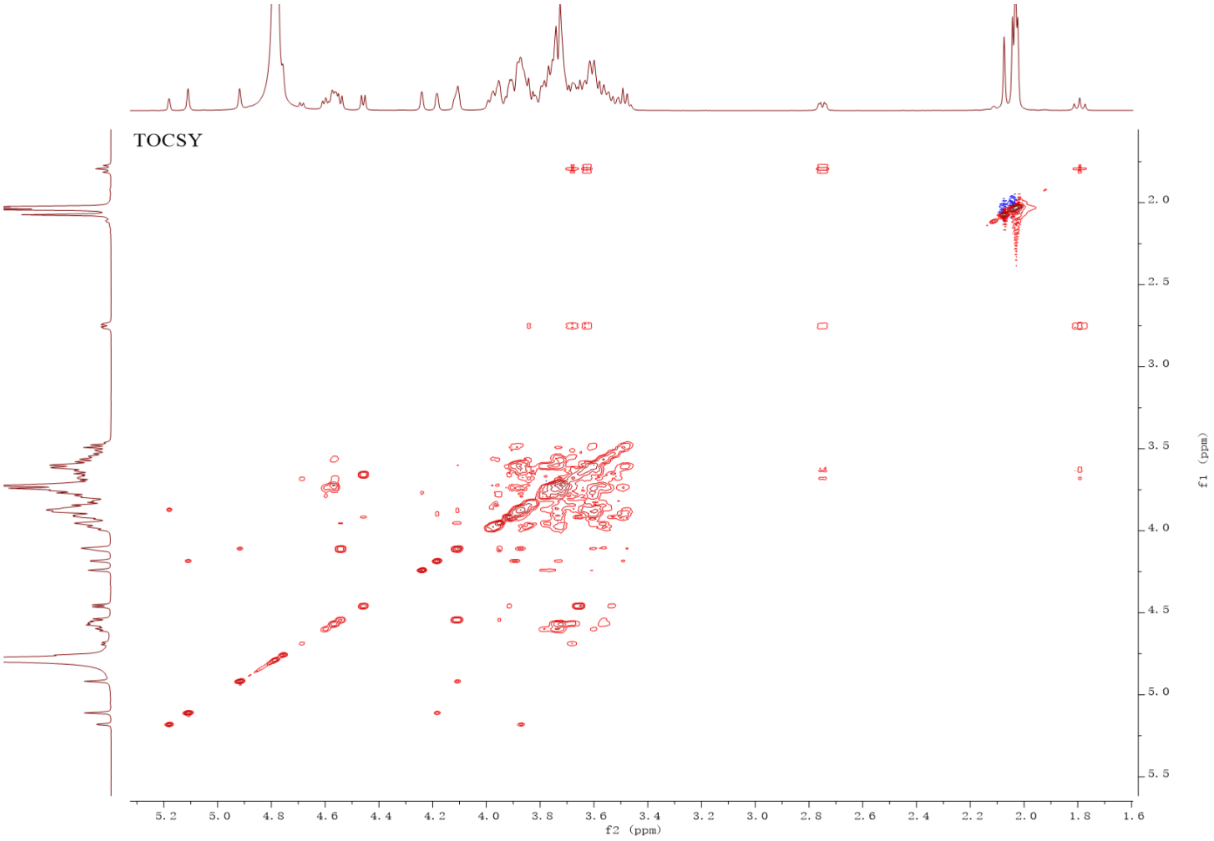
TOCSY
f2 (ppm)
f1 (ppm)

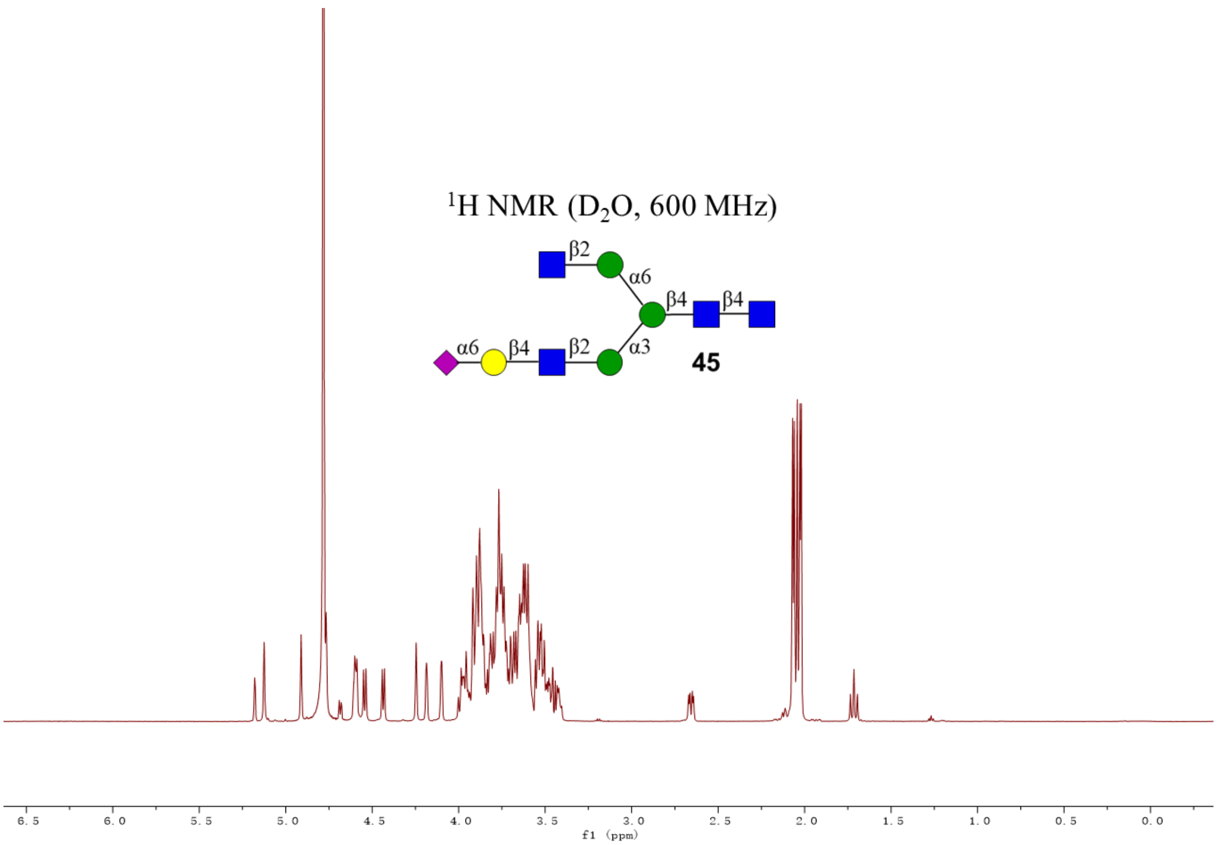

^{1}H NMR (D_2O, 600 MHz)
β2
α6
β4
β4
α6
β4
β2
α3
45
6.5 6.0 5.5 5.0 4.5 4.0 3.5 3.0 2.5 2.0 1.5 1.0 0.5 0.0
f1 (ppm)

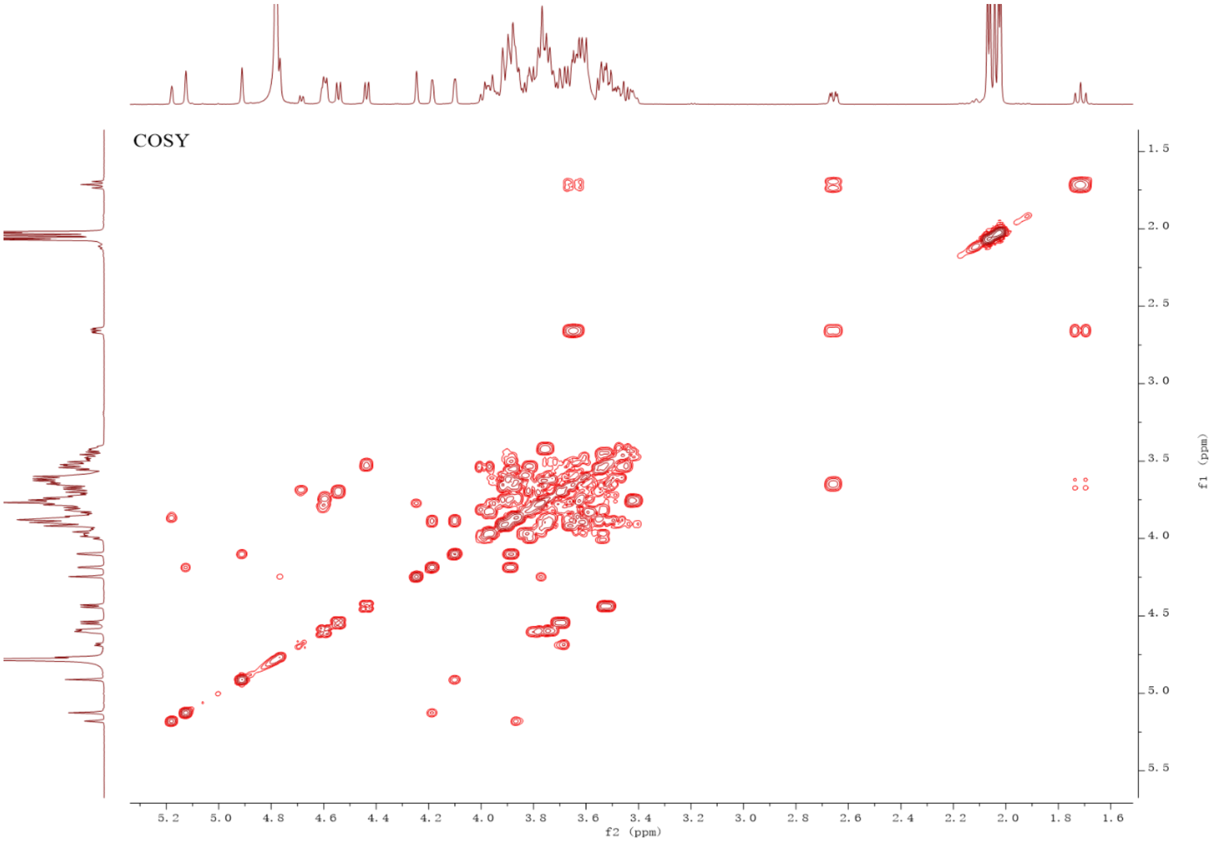

COSY
5.2 5.0 4.8 4.6 4.4 4.2 4.0 3.8 3.6 3.4 3.2 3.0 2.8 2.6 2.4 2.2 2.0 1.8 1.6
f2 (ppm)
1.5 2.0 2.5 3.0 3.5 4.0 4.5 5.0 5.5
f1 (ppm)

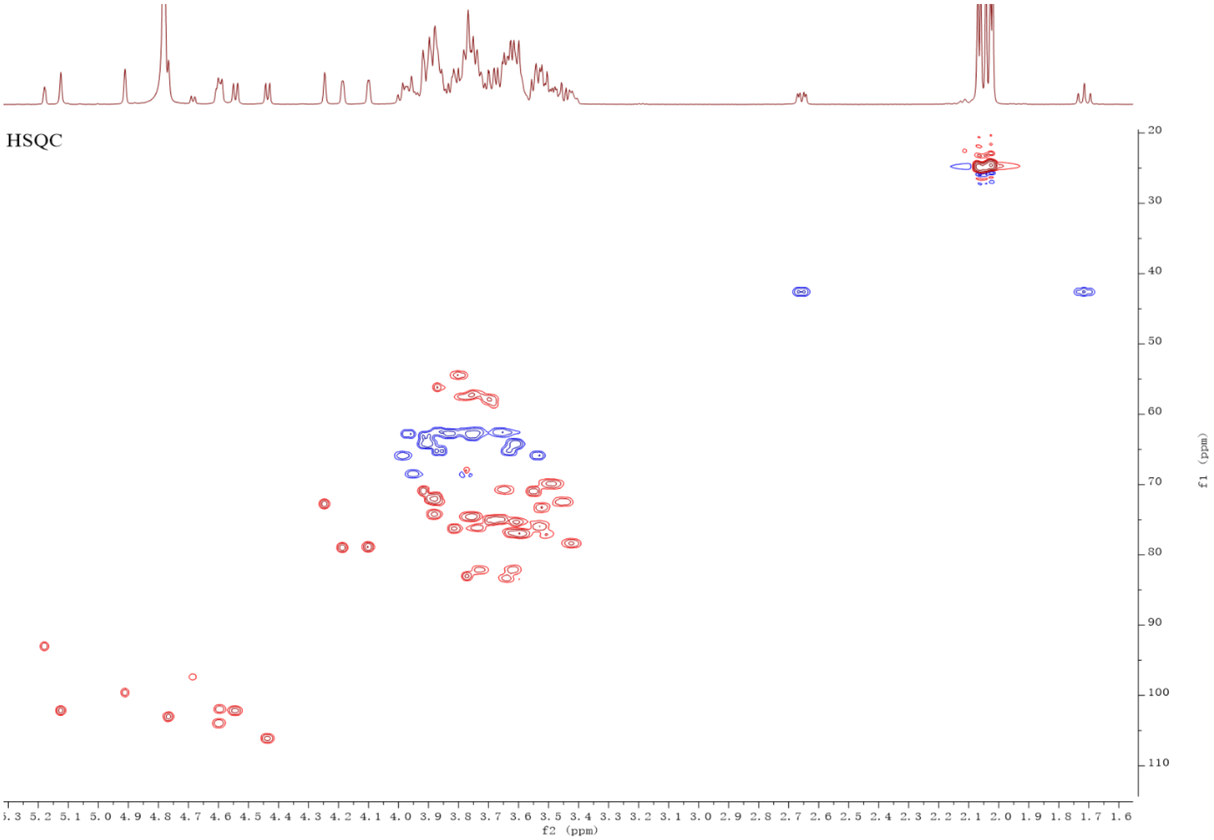
HSQC
f2 (ppm)
f1 (ppm)

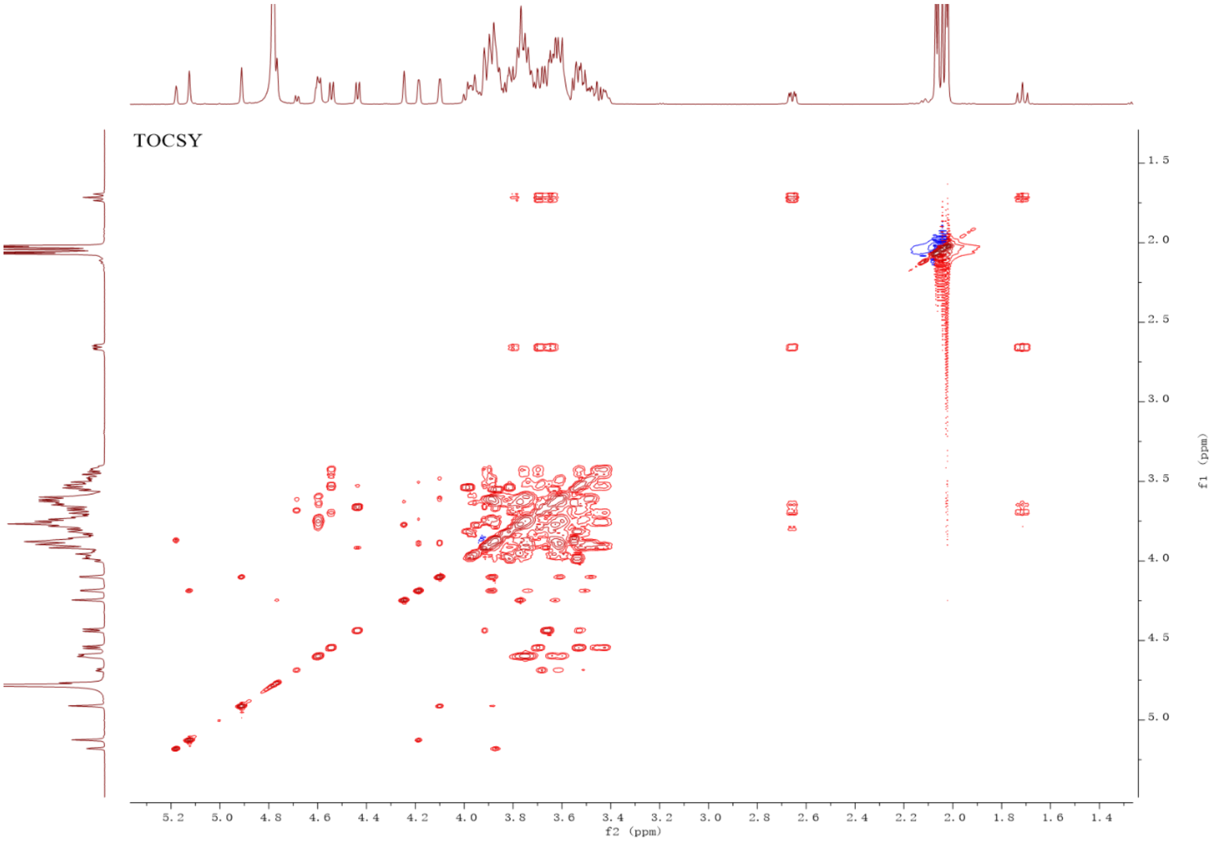
TOCSY
f2 (ppm)
f1 (ppm)

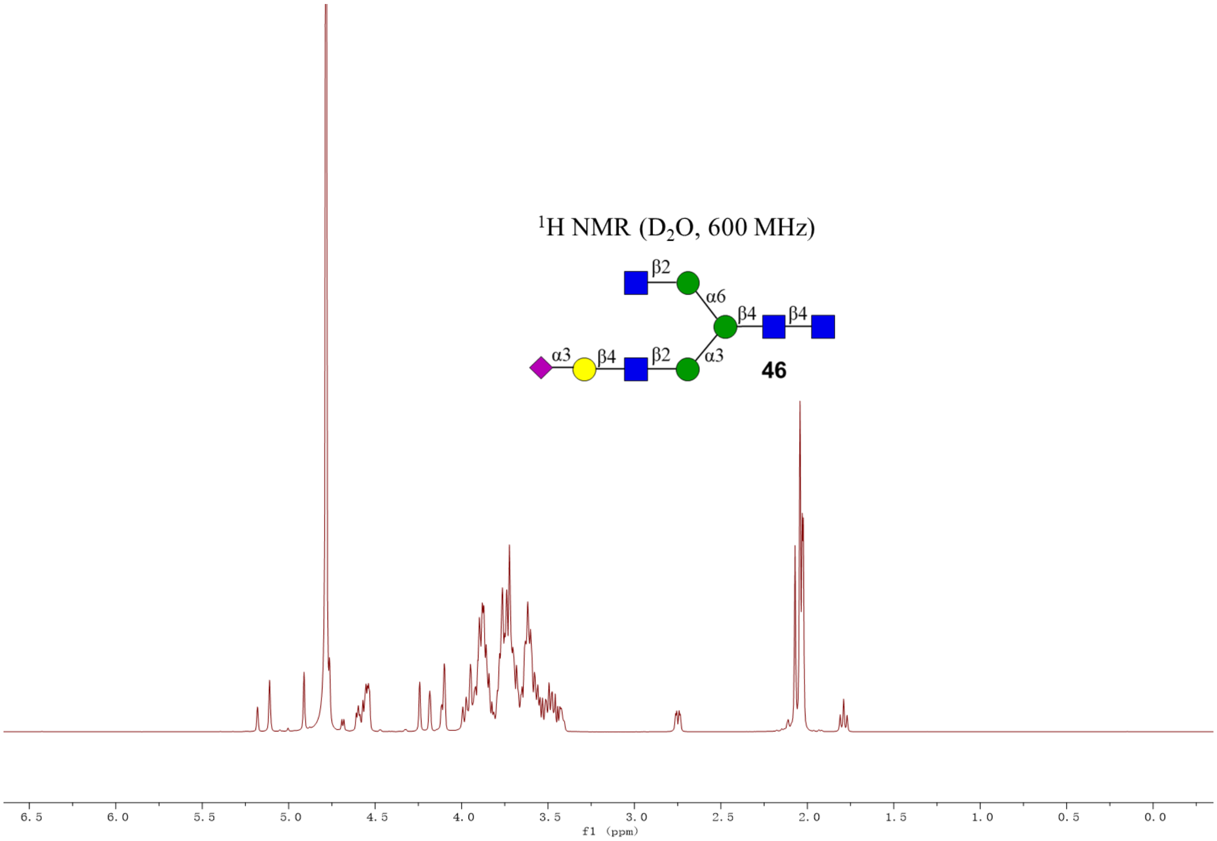
^{1}H NMR (D_2O, 600 MHz)
β2
α6
β4
β4
α3
β4
β2
α3
46
6.5 6.0 5.5 5.0 4.5 4.0 3.5 3.0 2.5 2.0 1.5 1.0 0.5 0.0
f1 (ppm)

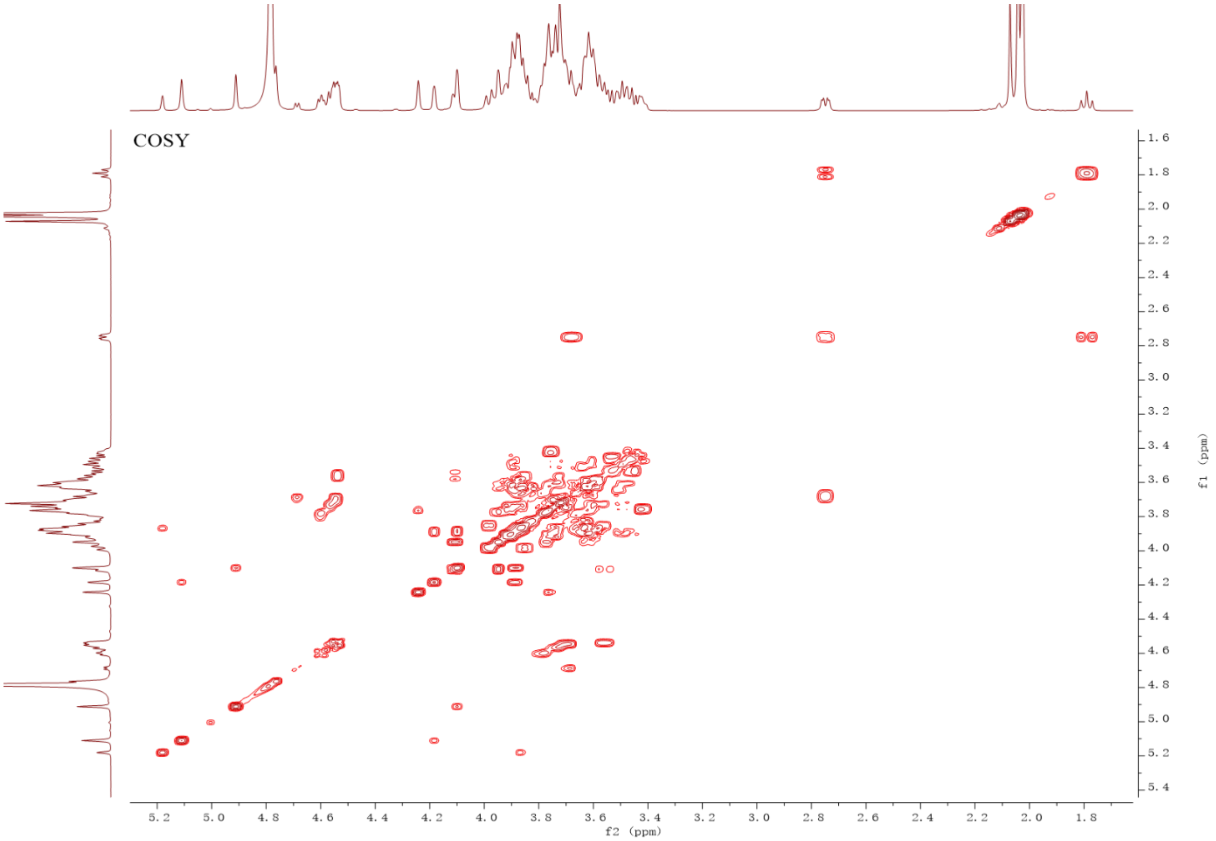
COSY
f1 (ppm)
f2 (ppm)

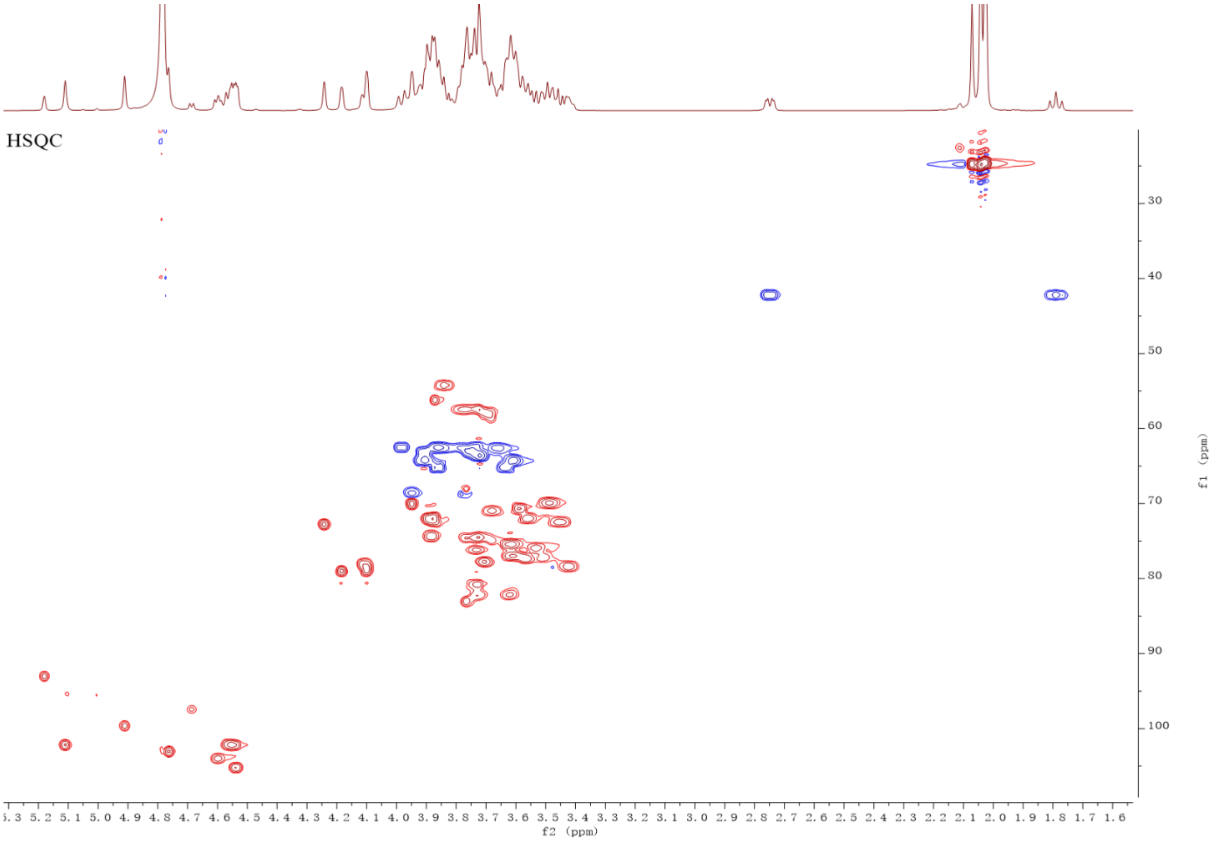
HSQC
f2 (ppm)
f1 (ppm)

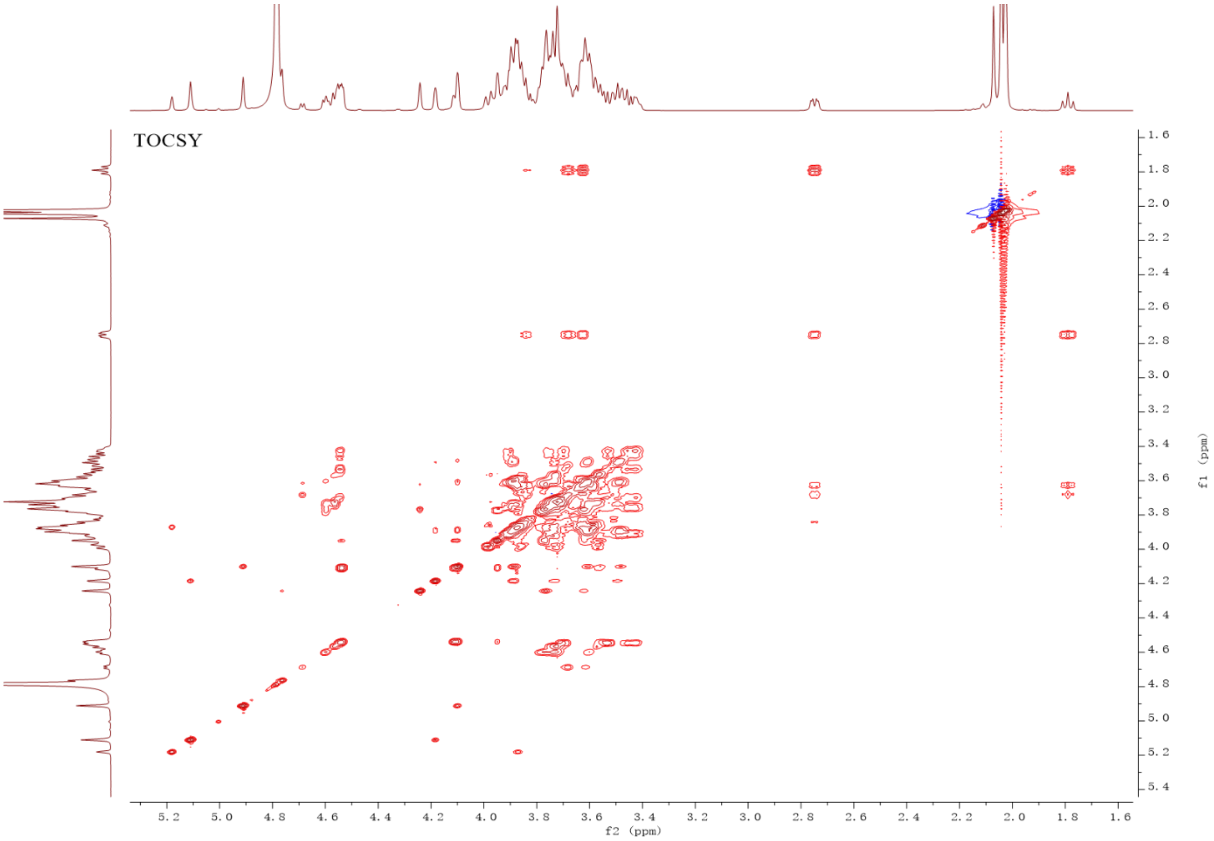
TOCSY
f2 (ppm)
f1 (ppm)

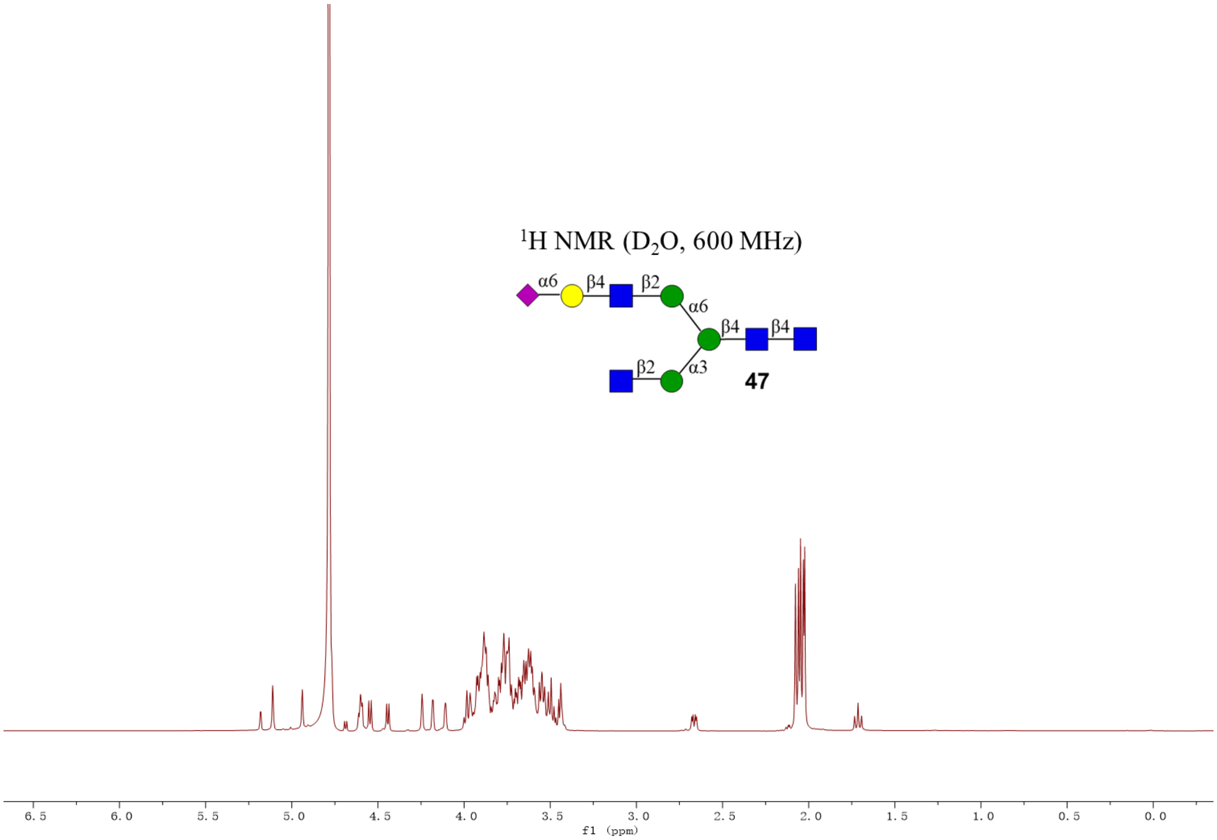
^{1}H NMR (D_2O, 600 MHz)
α6
β4
β2
α6
β4
β4
β2
α3
47
f1 (ppm)

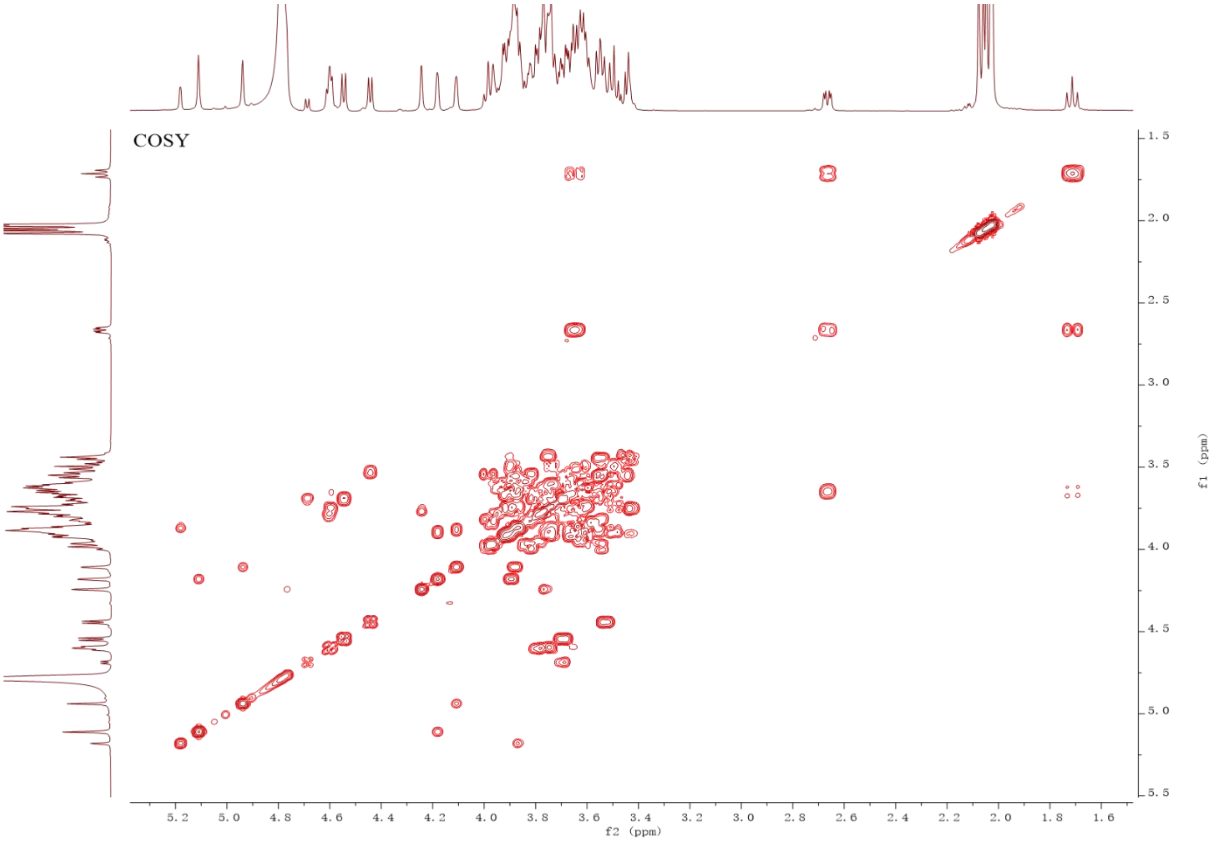
COSY
f2 (ppm)
f1 (ppm)

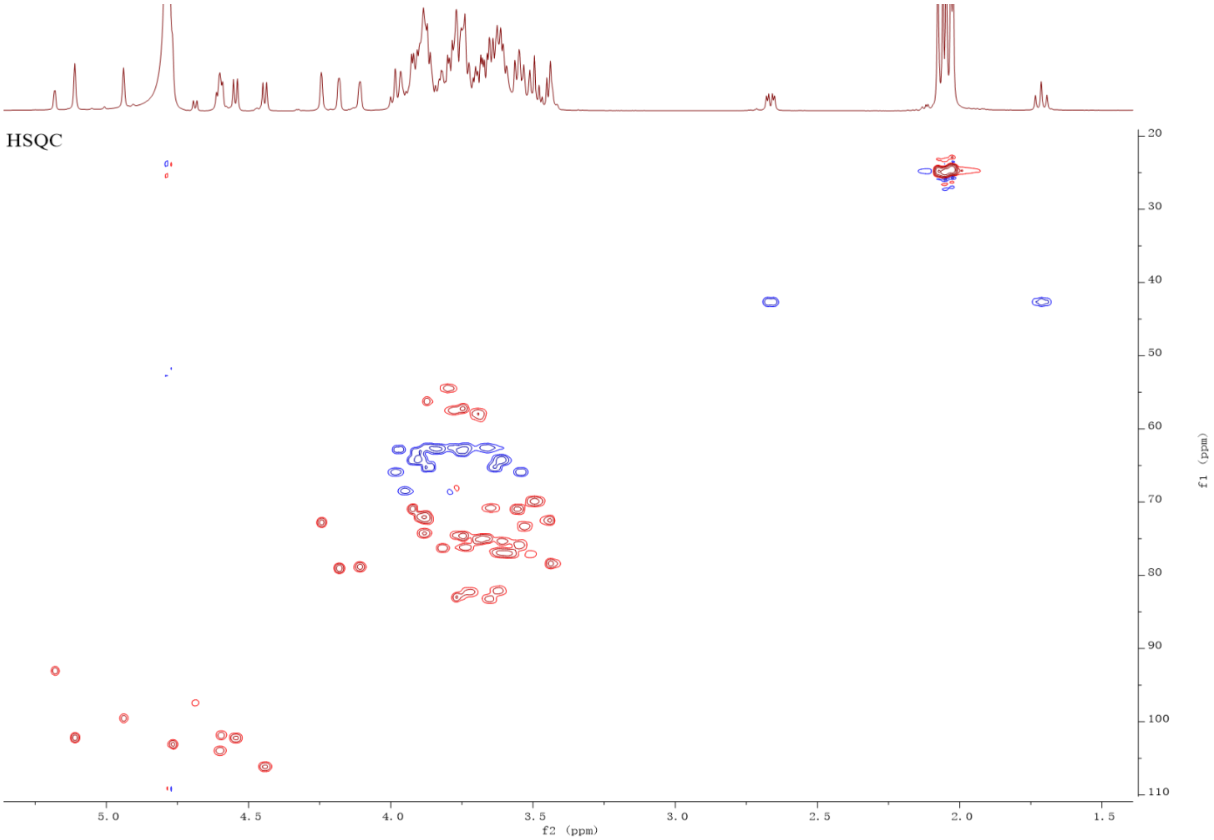
HSQC
f2 (ppm)
f1 (ppm)

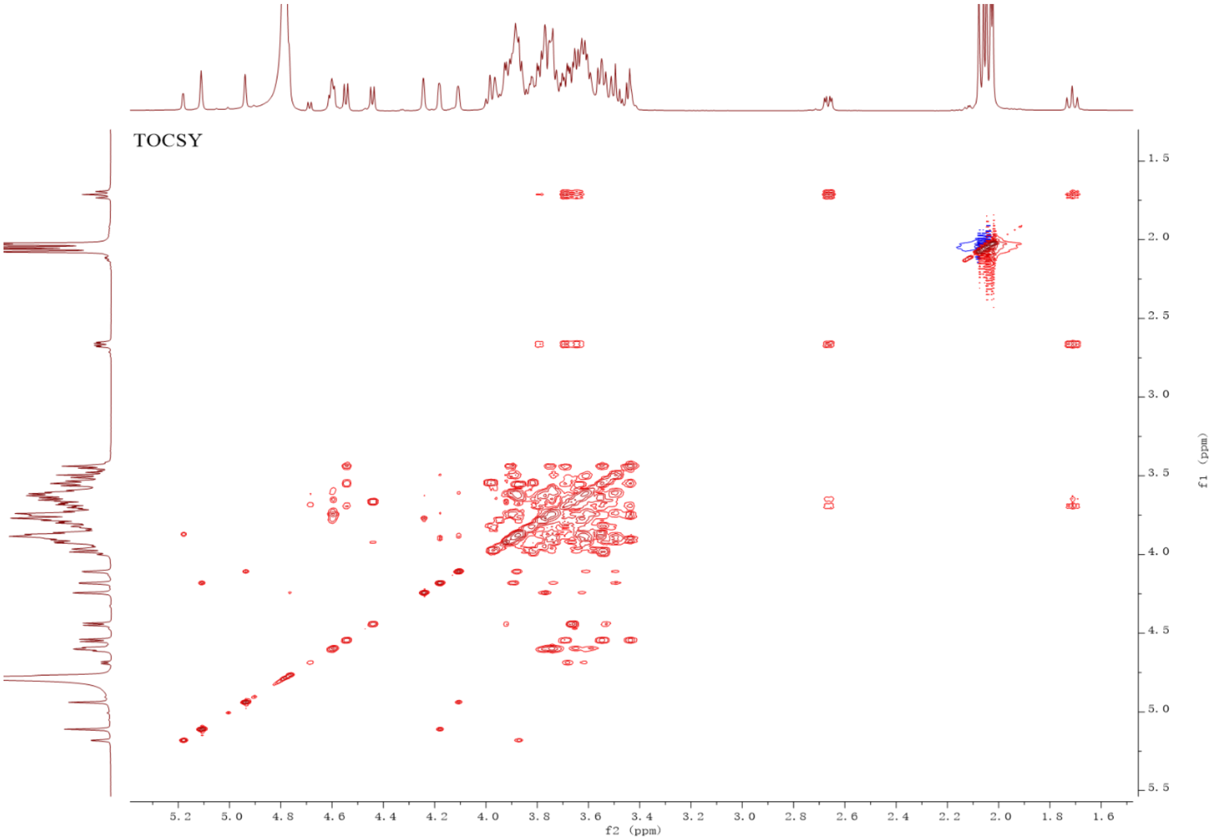
TOCSY
f2 (ppm)
f1 (ppm)

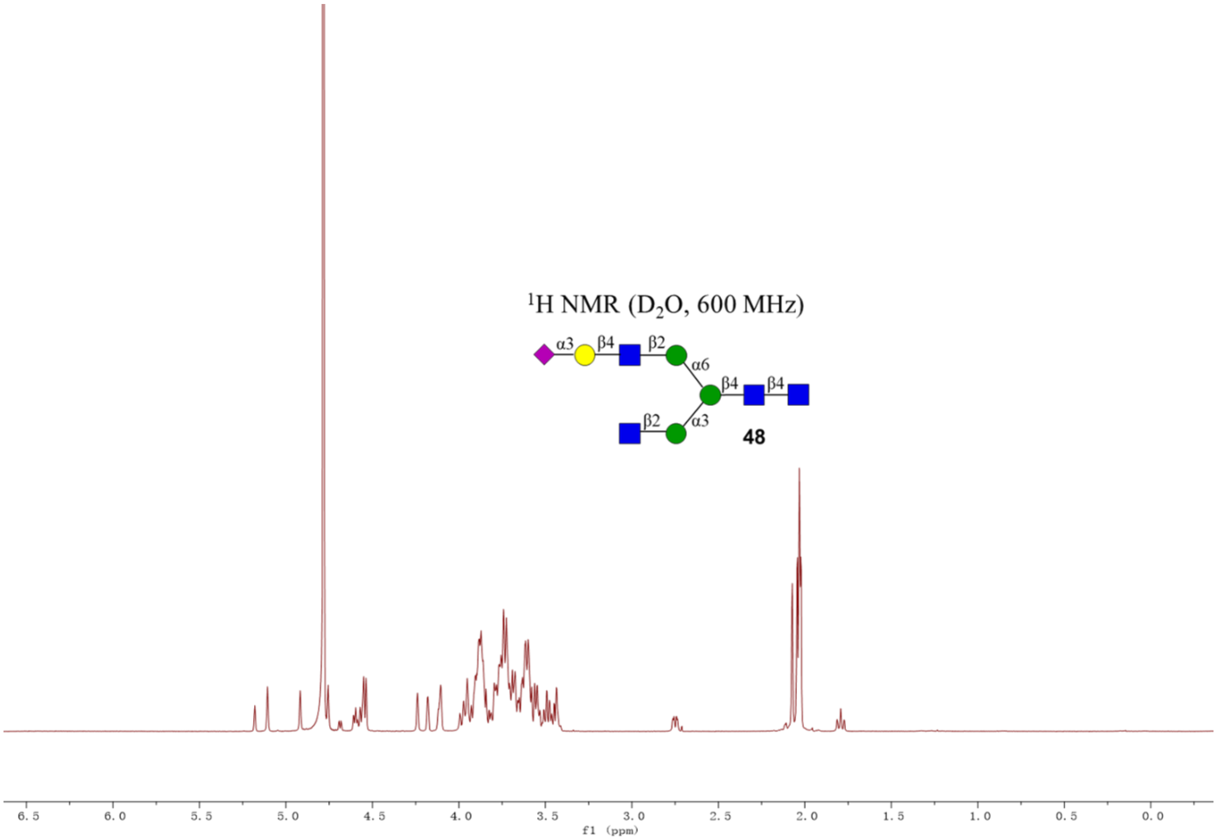

1H NMR (D2O, 600 MHz)
α3
β4
β2
α6
β4
β4
β2
α3
48
6.5
6.0
5.5
5.0
4.5
4.0
3.5
3.0
2.5
2.0
1.5
1.0
0.5
0.0
f1 (ppm)

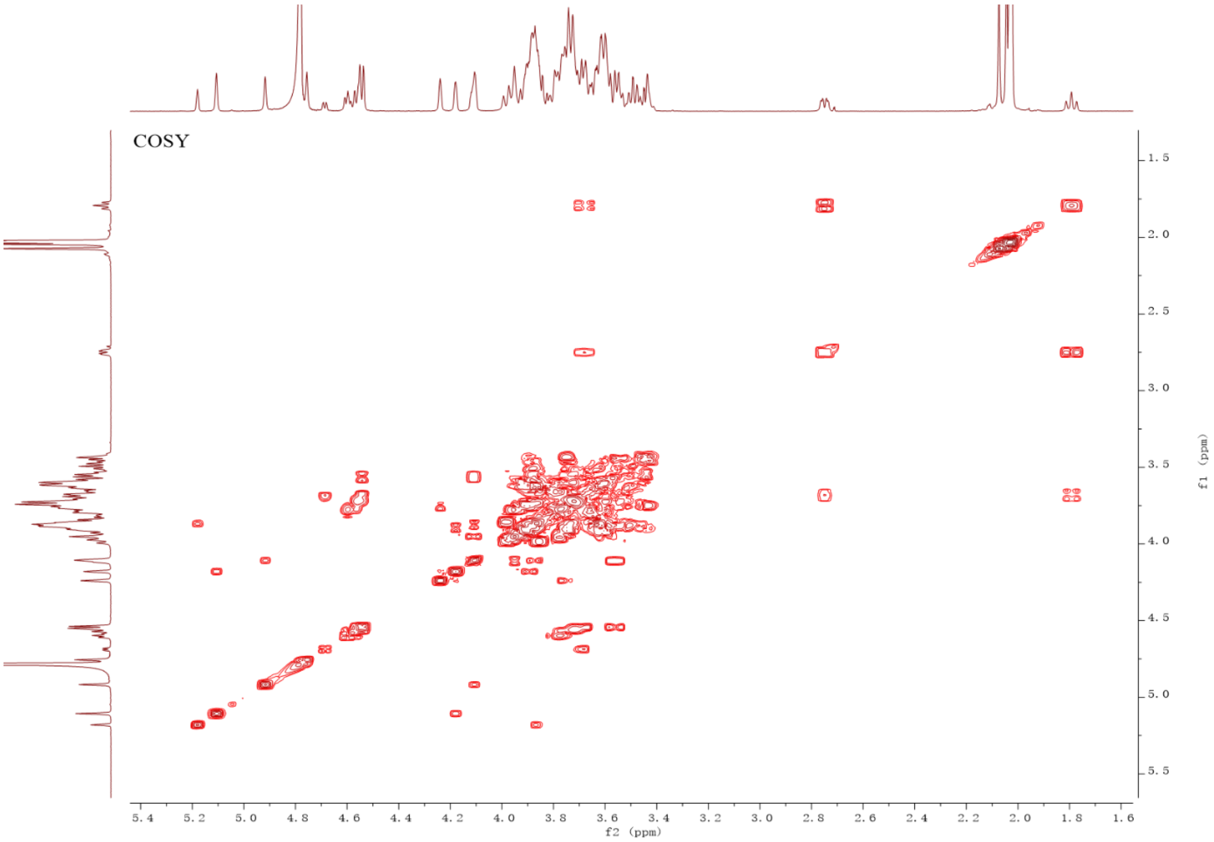

COSY
f2 (ppm)
f1 (ppm)

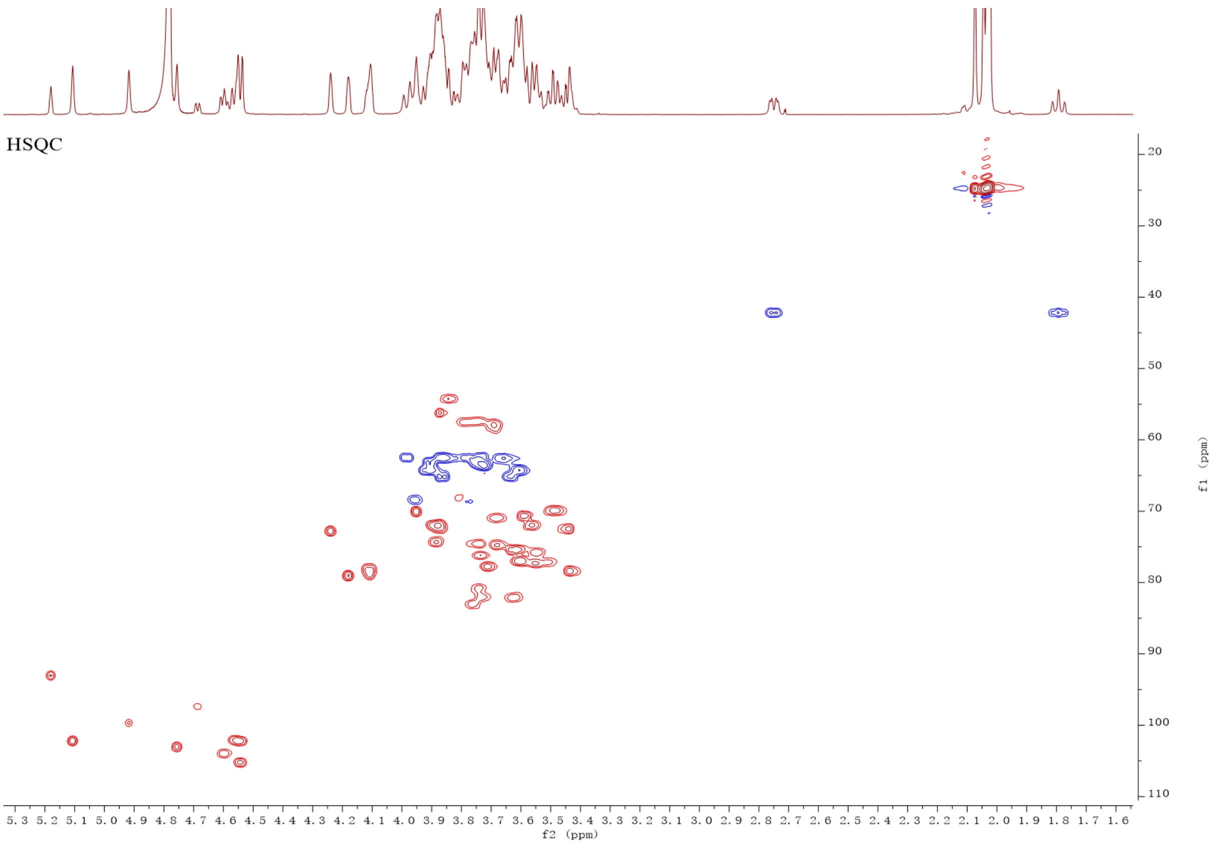
HSQC
f2 (ppm)
f1 (ppm)

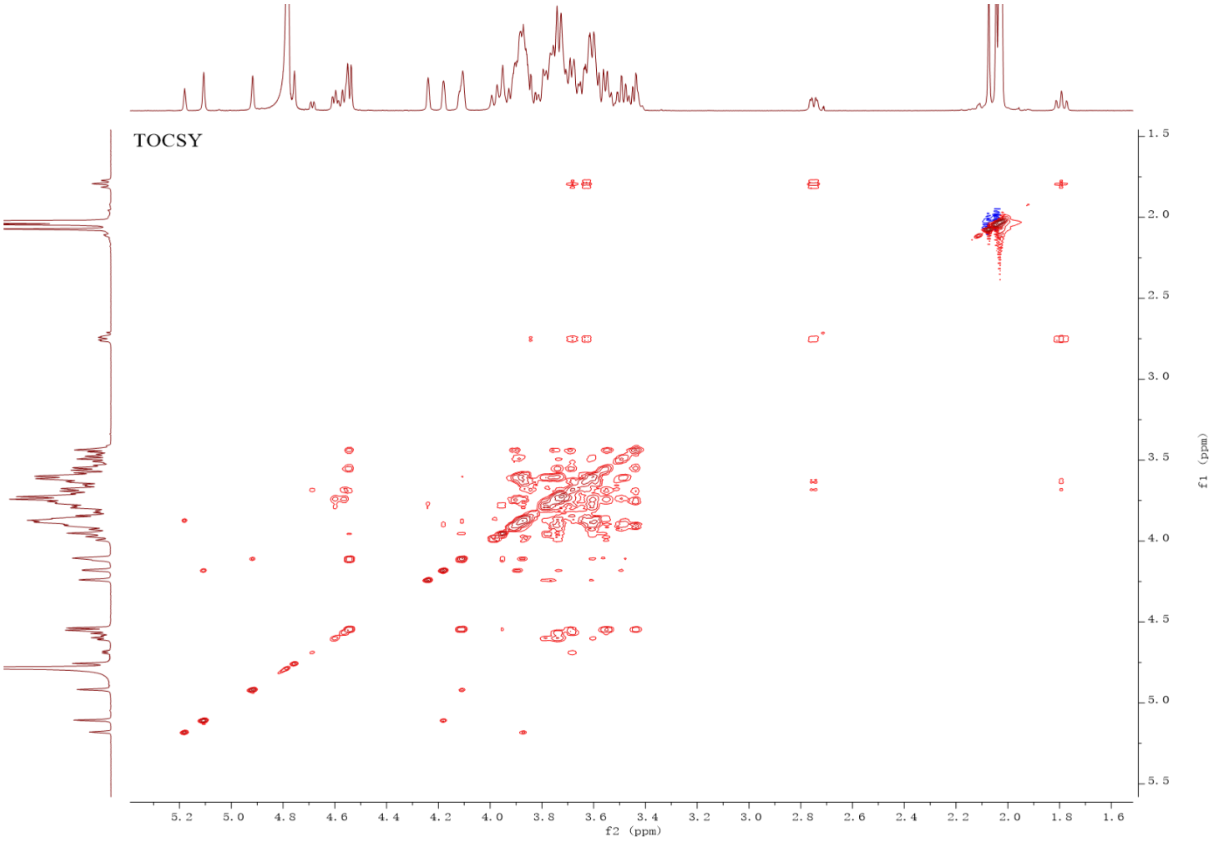
TOCSY
f2 (ppm)
f1 (ppm)

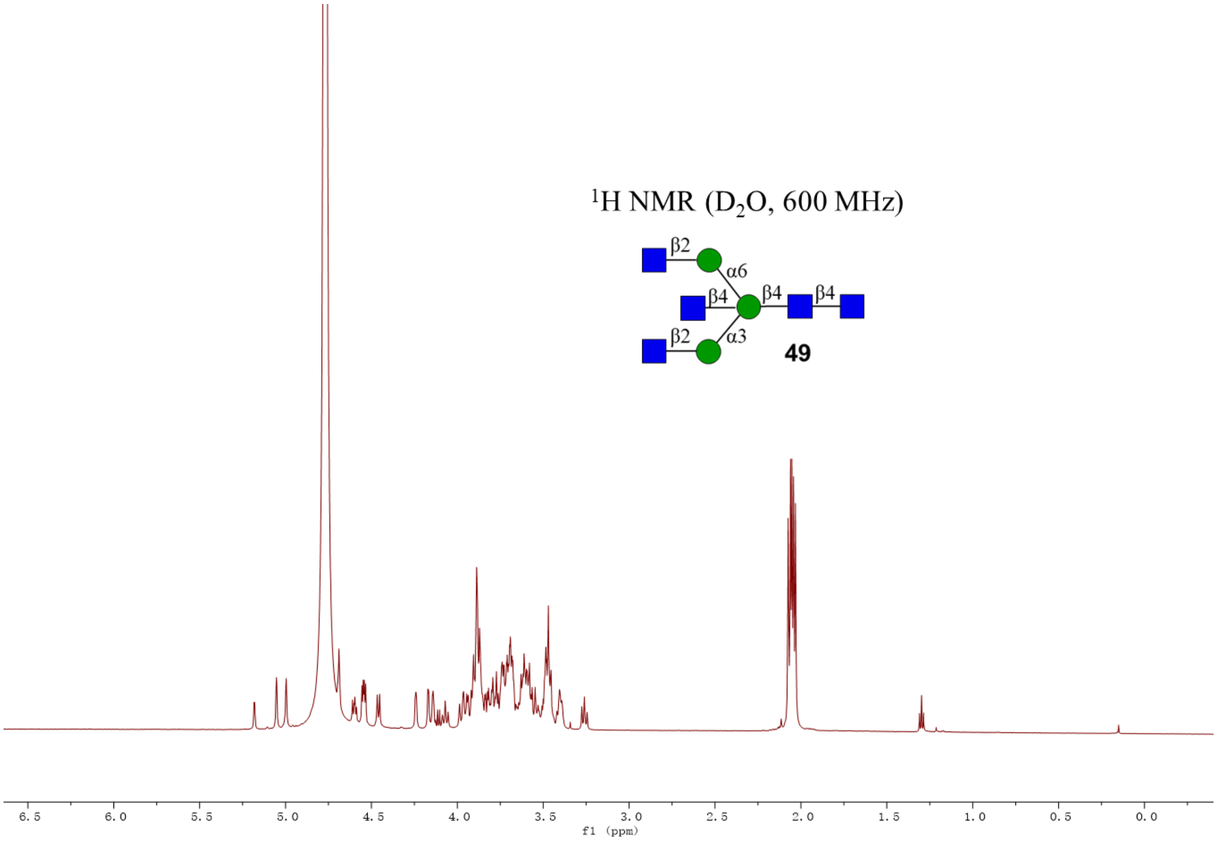
^{1}H NMR (D_2O, 600 MHz)
β2
α6
β4
β4
β4
β2
α3
49
6.5 6.0 5.5 5.0 4.5 4.0 3.5 3.0 2.5 2.0 1.5 1.0 0.5 0.0
f1 (ppm)

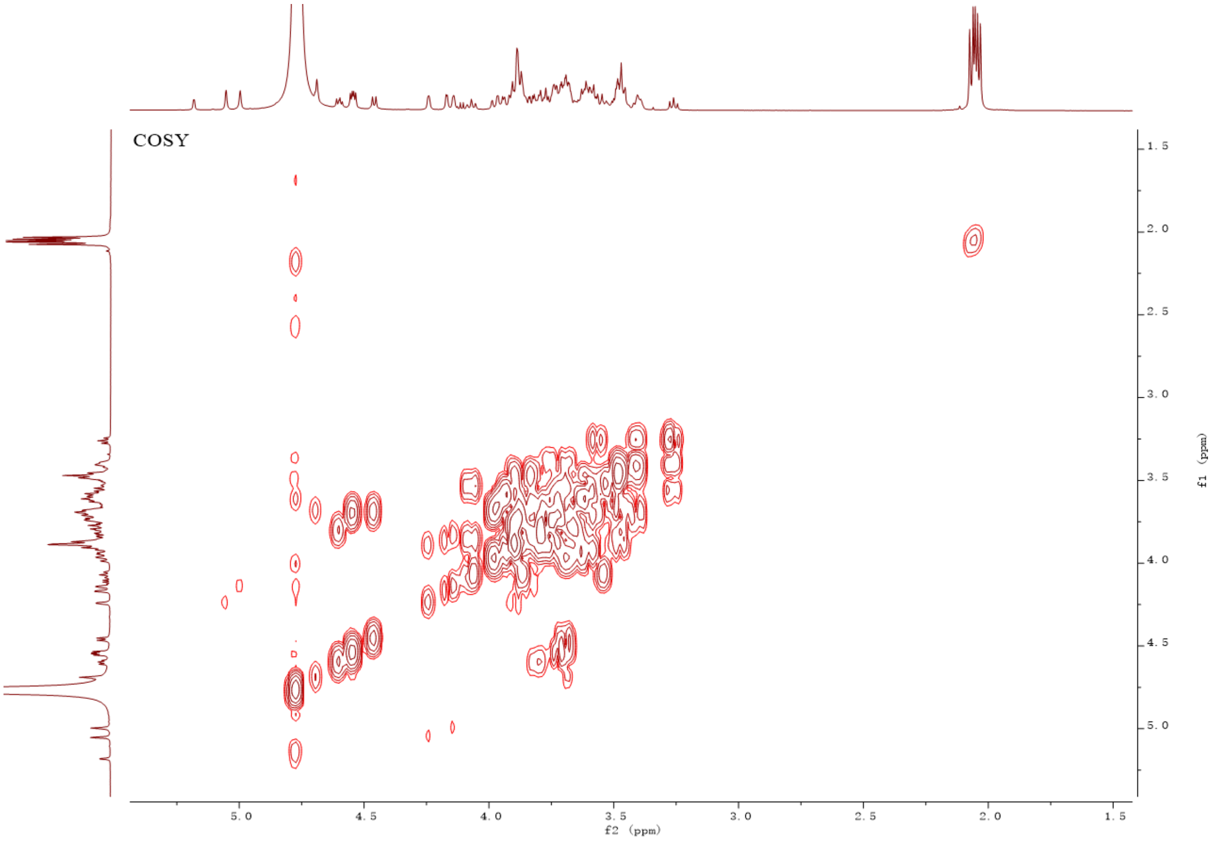
COSY
5.0 4.5 4.0 3.5 3.0 2.5 2.0 1.5
f2 (ppm)
1.5 2.0 2.5 3.0 3.5 4.0 4.5 5.0
f1 (ppm)

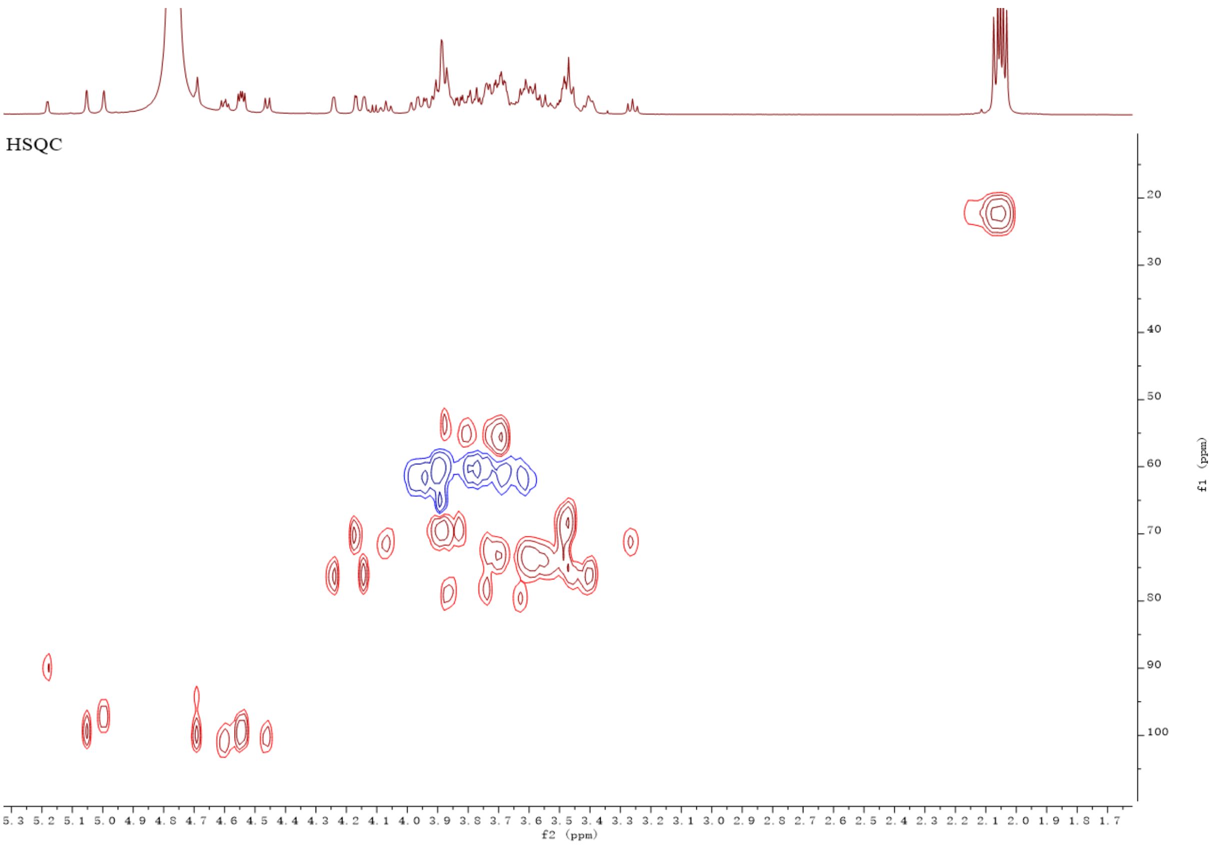
HSQC
f2 (ppm)
f1 (ppm)

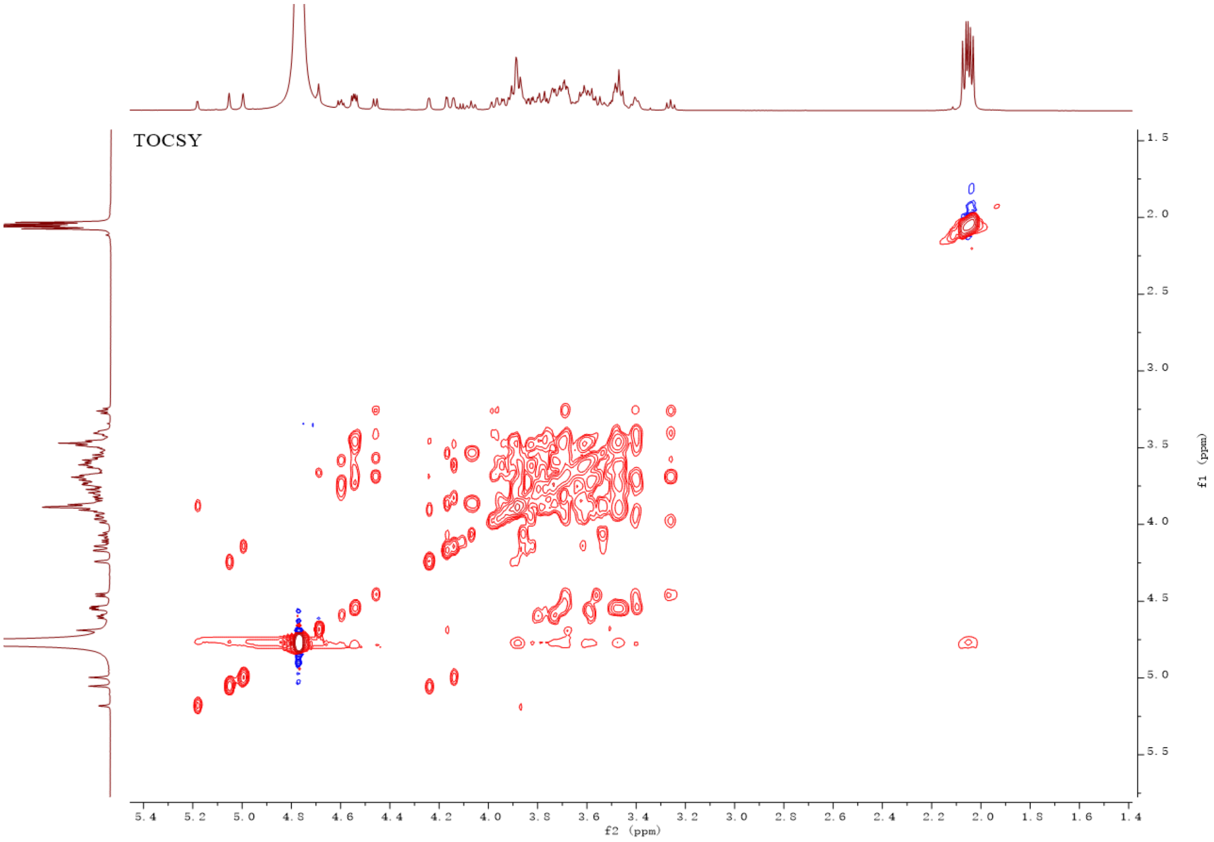
TOCSY
f2 (ppm)
f1 (ppm)

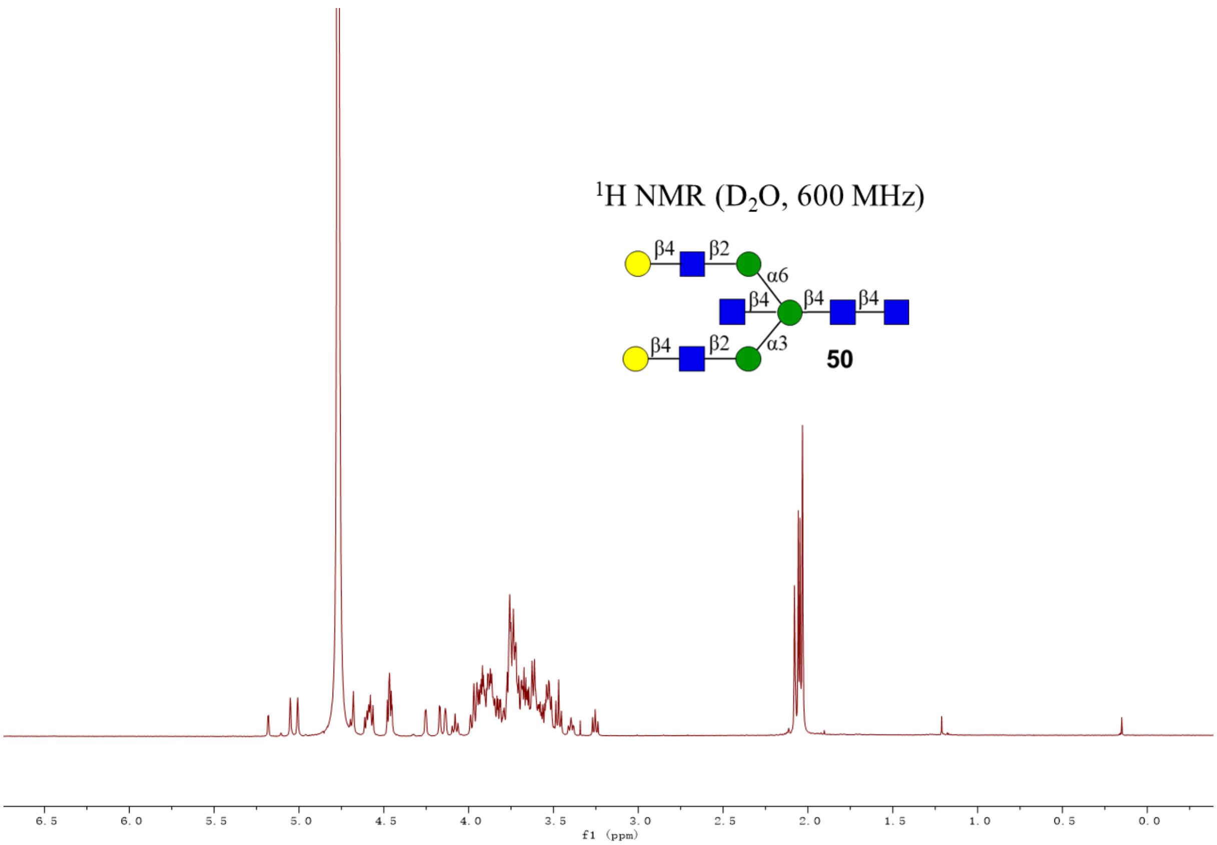
^{1}H NMR (D_2O, 600 MHz)
β4
β2
α6
β4
β4
β4
β4
β2
α3
50
f1 (ppm)

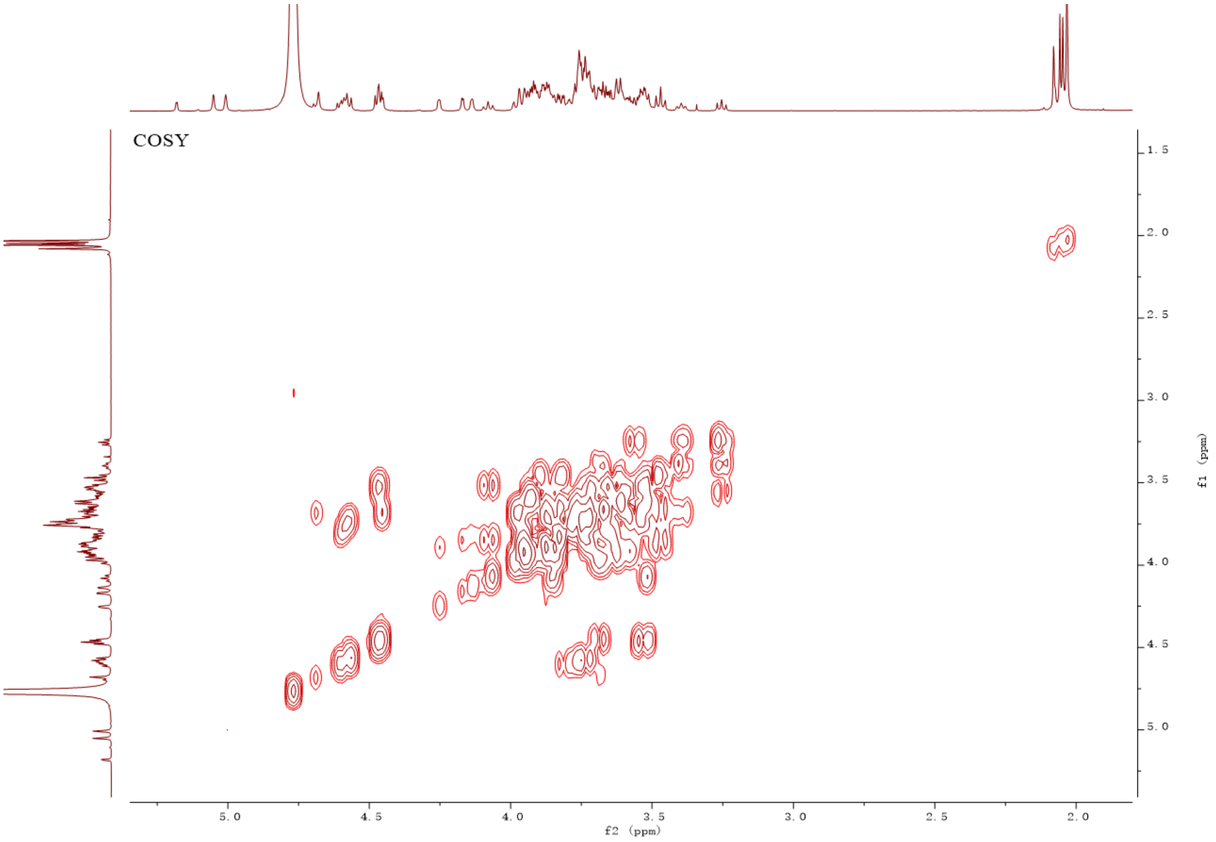
COSY
f2 (ppm)
f1 (ppm)

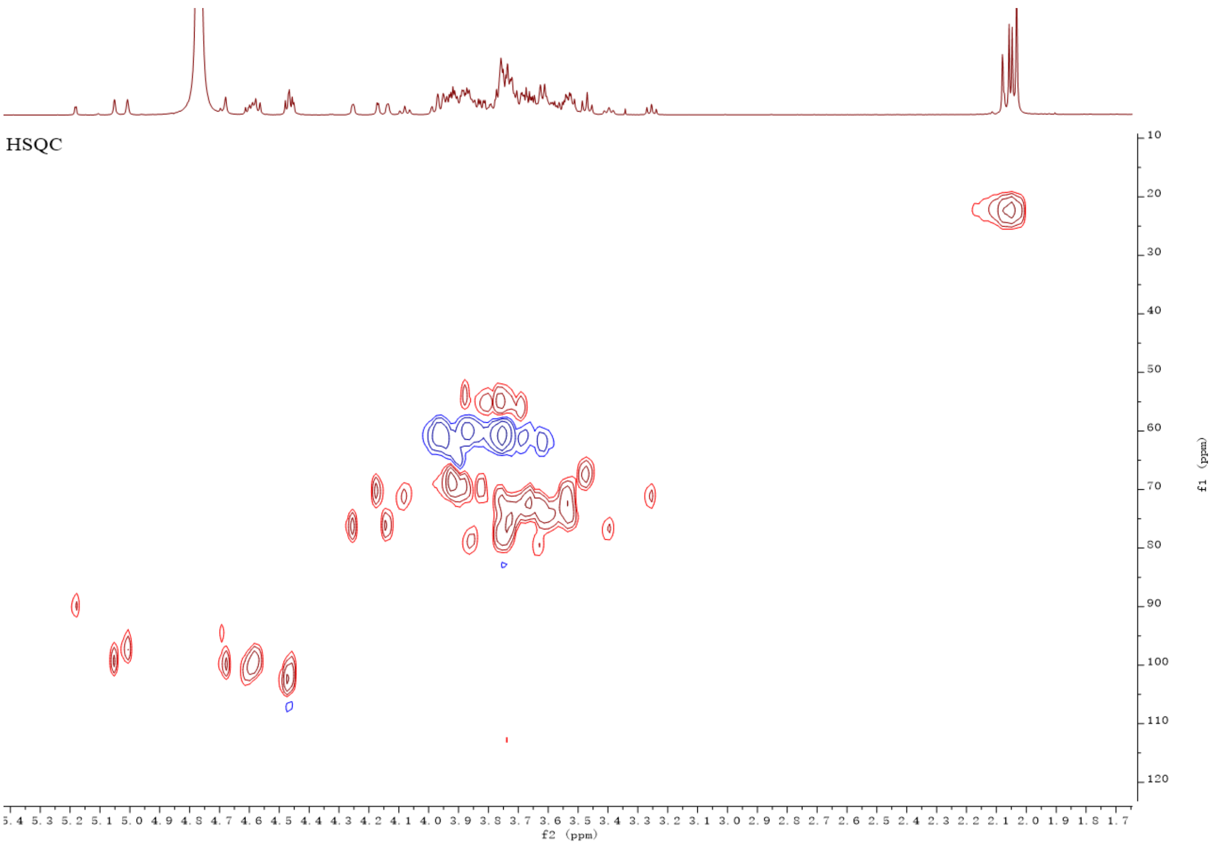
HSQC
f2 (ppm)
f1 (ppm)

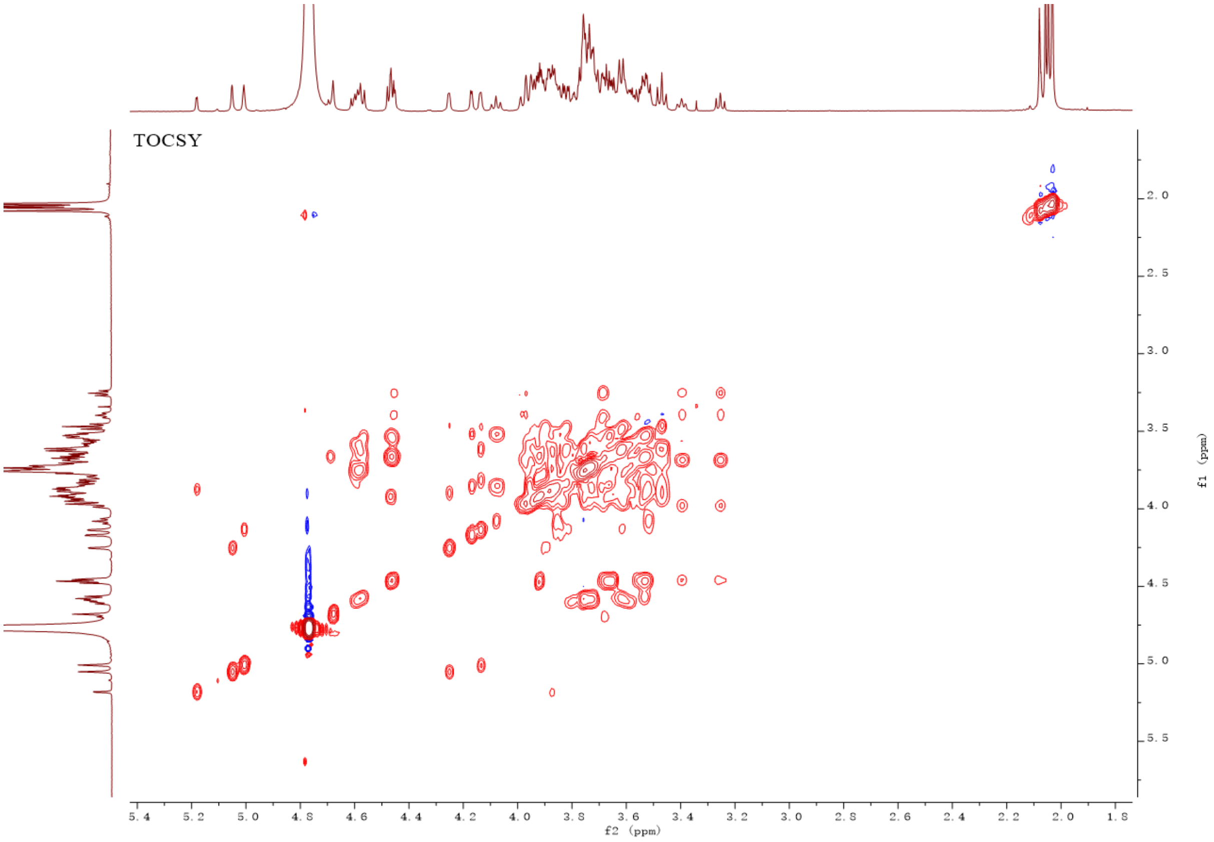
TOCSY
f2 (ppm)
f1 (ppm)

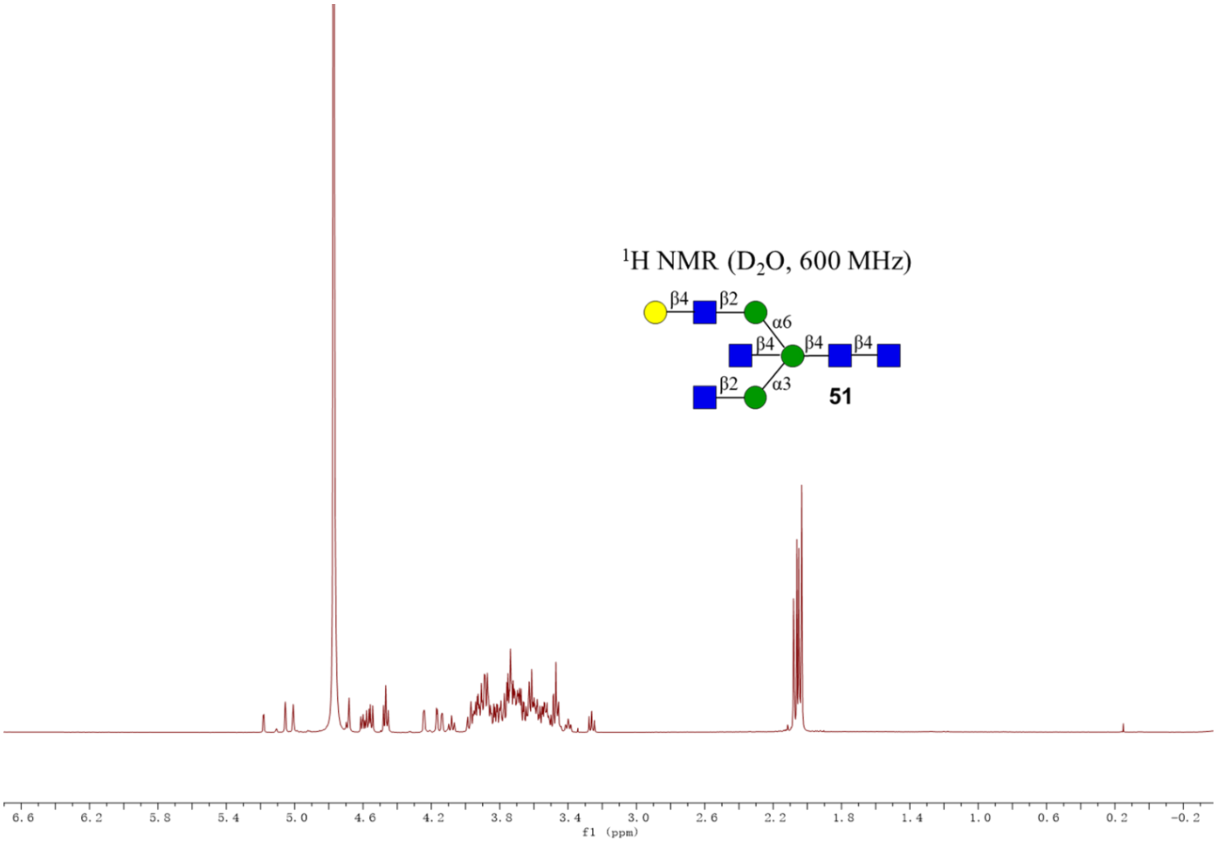

^{1}H NMR (D$_2$O, 600 MHz)
β4
β2
α6
β4
β4
β4
β2
α3
51
f1 (ppm)

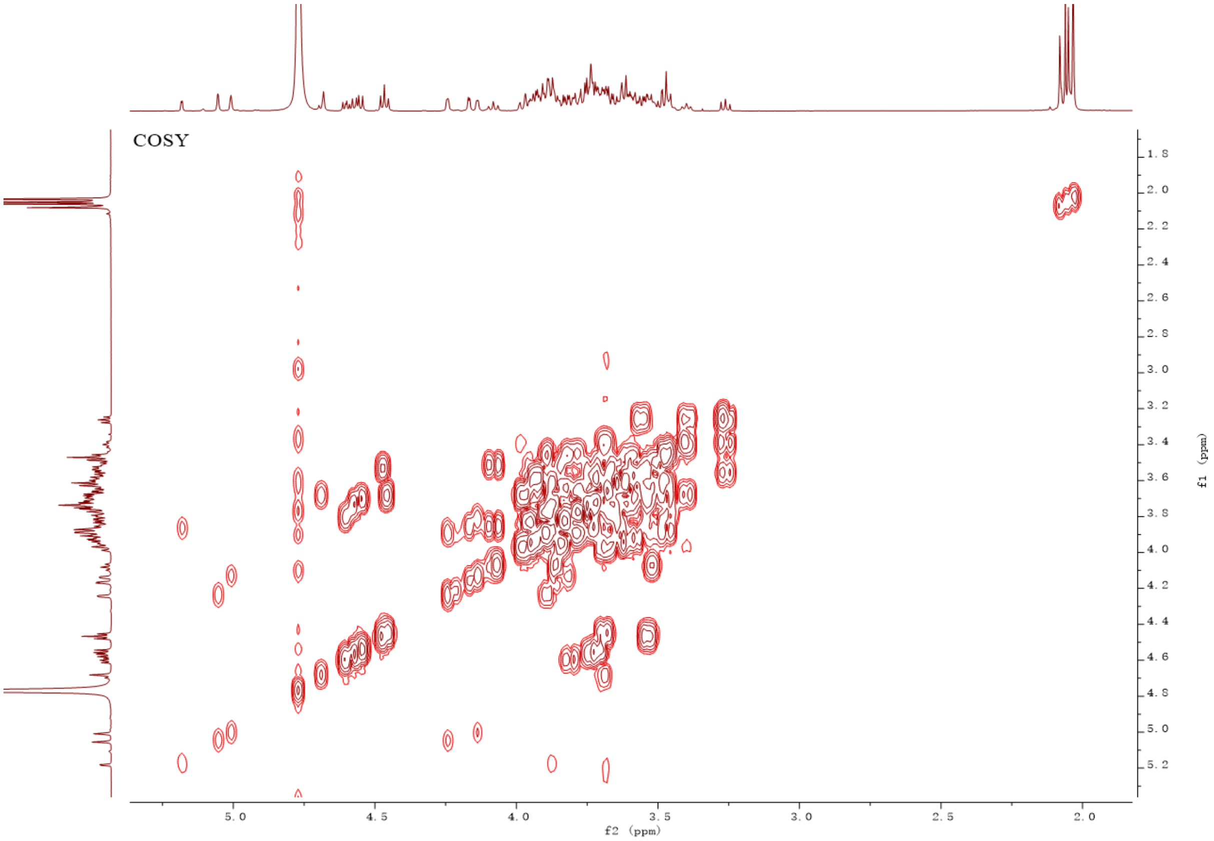

COSY
f2 (ppm)
f1 (ppm)

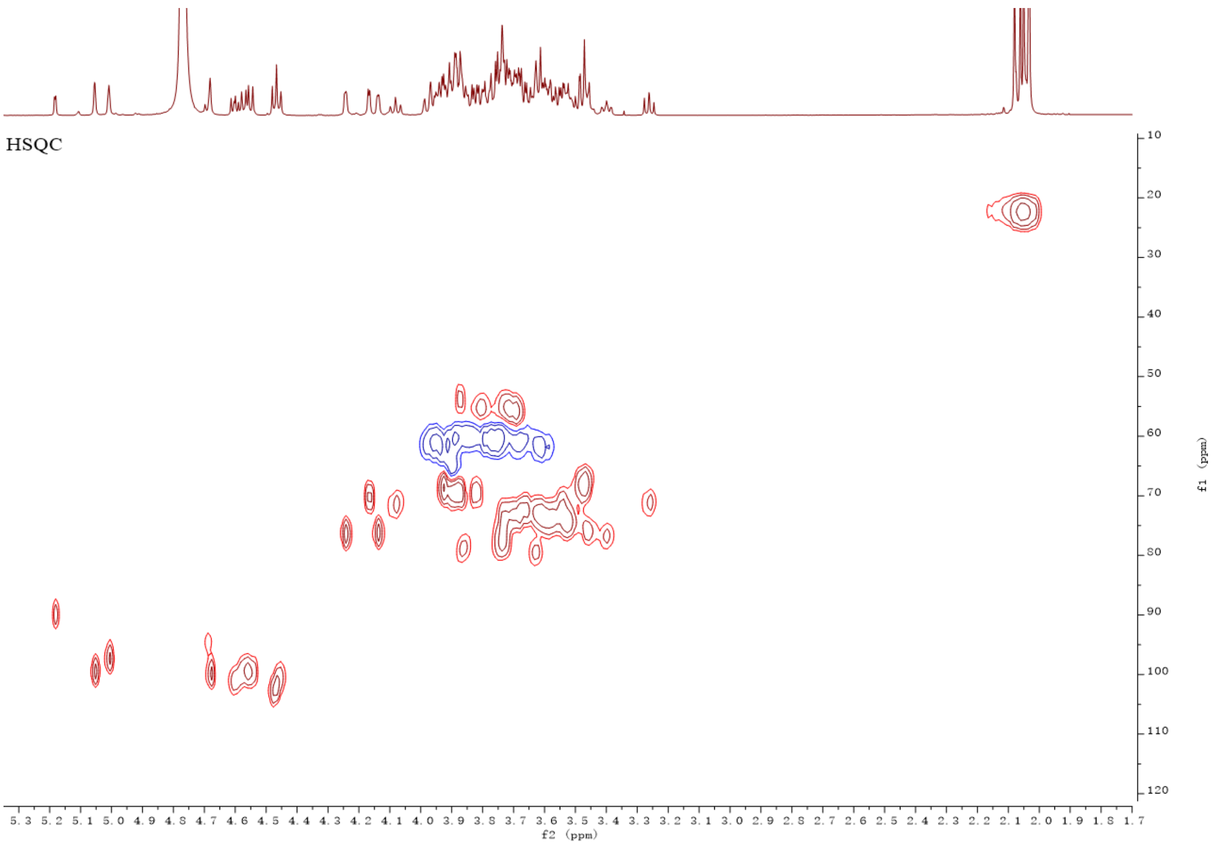
HSQC
f1 (ppm)
10 20 30 40 50 60 70 80 90 100 110 120
5.3 5.2 5.1 5.0 4.9 4.8 4.7 4.6 4.5 4.4 4.3 4.2 4.1 4.0 3.9 3.8 3.7 3.6 3.5 3.4 3.3 3.2 3.1 3.0 2.9 2.8 2.7 2.6 2.5 2.4 2.3 2.2 2.1 2.0 1.9 1.8 1.7
f2 (ppm)

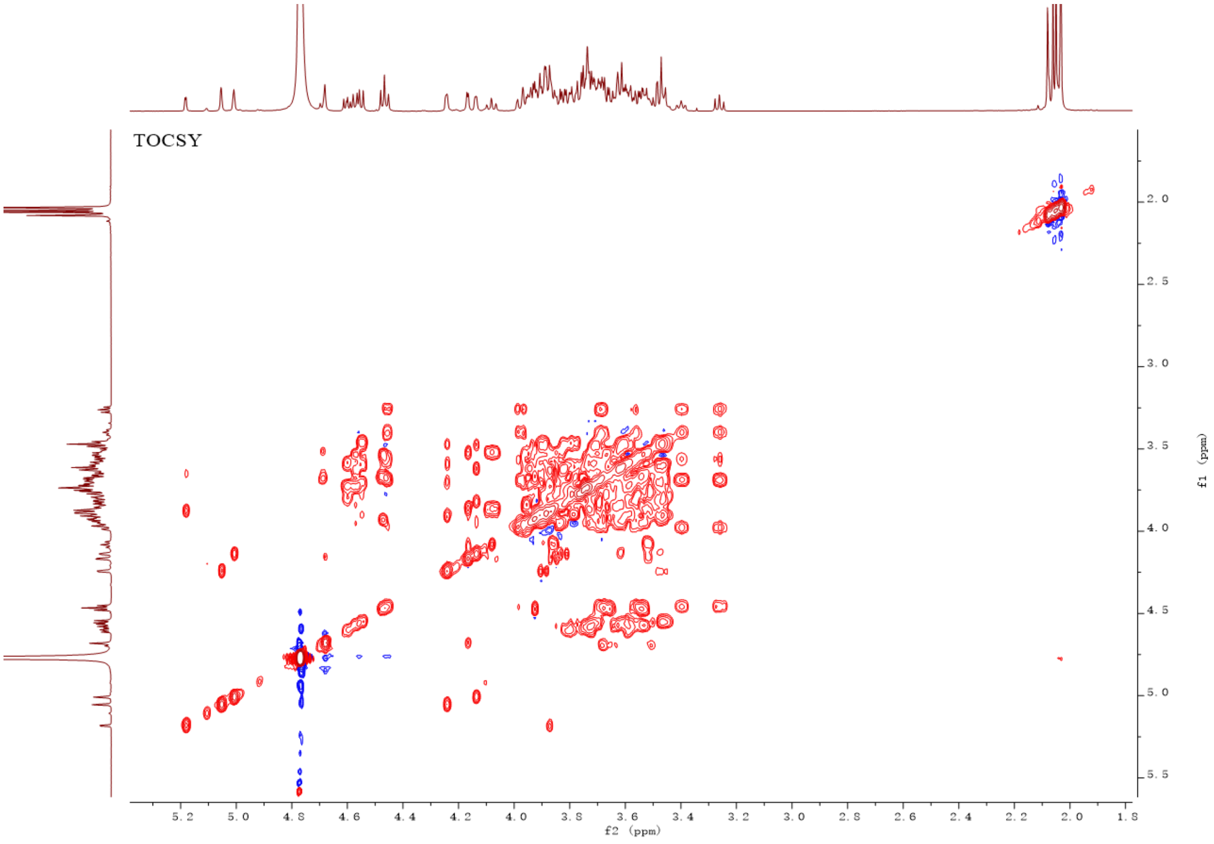
TOCSY
f1 (ppm)
2.0 2.5 3.0 3.5 4.0 4.5 5.0 5.5
5.2 5.0 4.8 4.6 4.4 4.2 4.0 3.8 3.6 3.4 3.2 3.0 2.8 2.6 2.4 2.2 2.0 1.8
f2 (ppm)

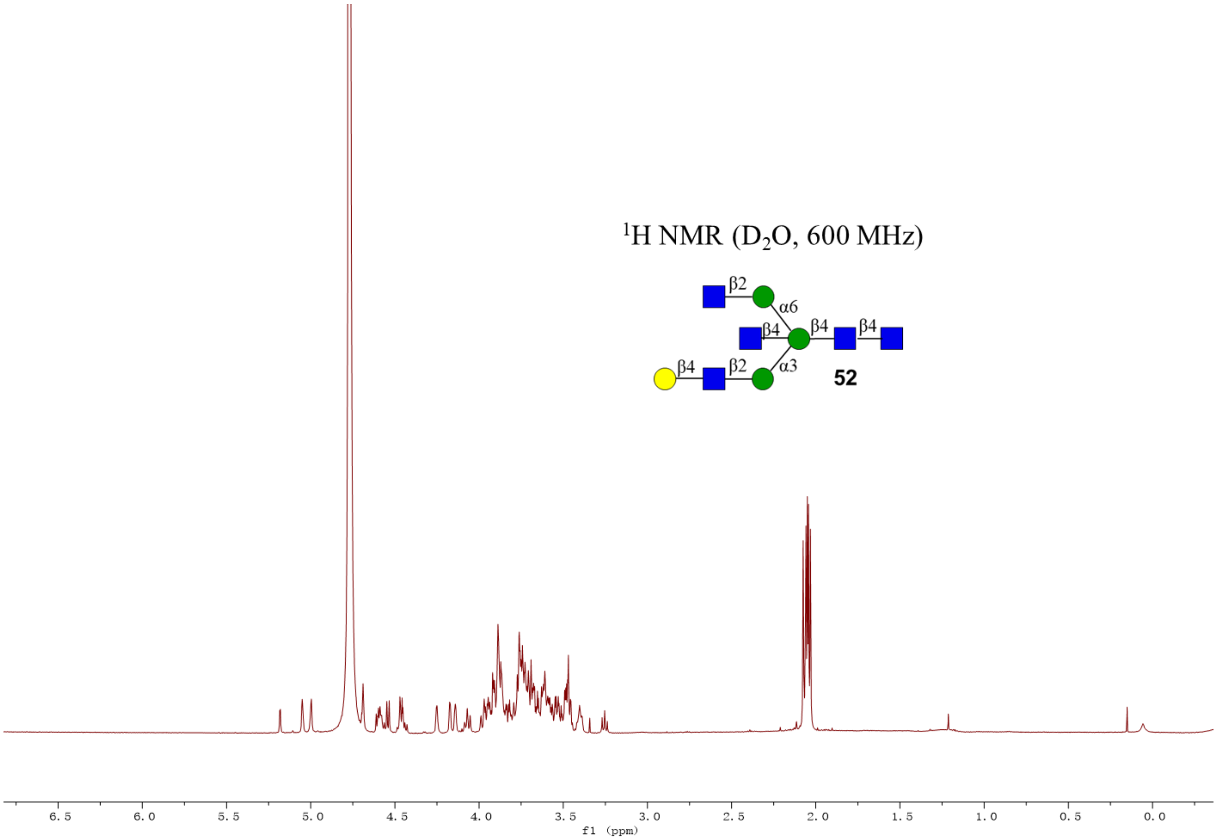

^{1}H NMR (D_2O, 600 MHz)
β2
α6
β4
β4
β4
β4
β2
α3
52
f1 (ppm)

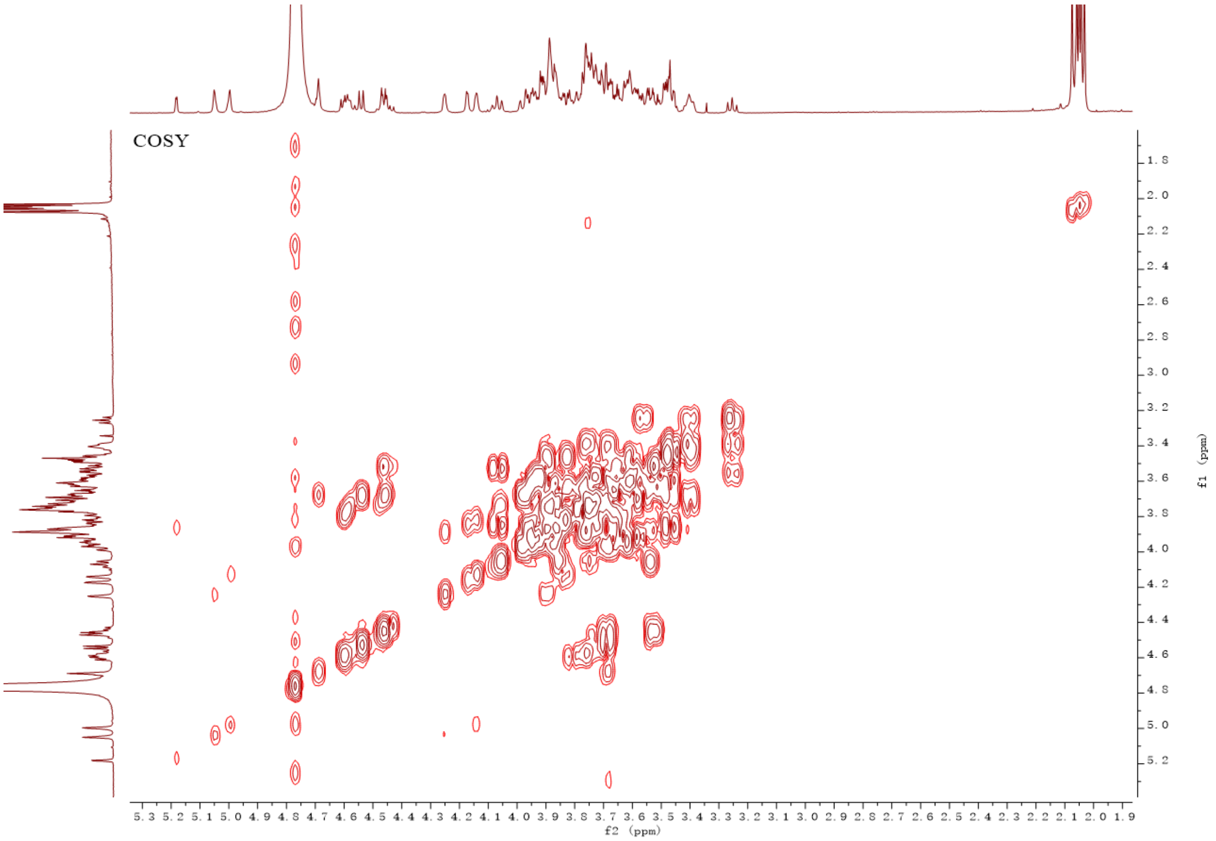

COSY
f2 (ppm)
f1 (ppm)

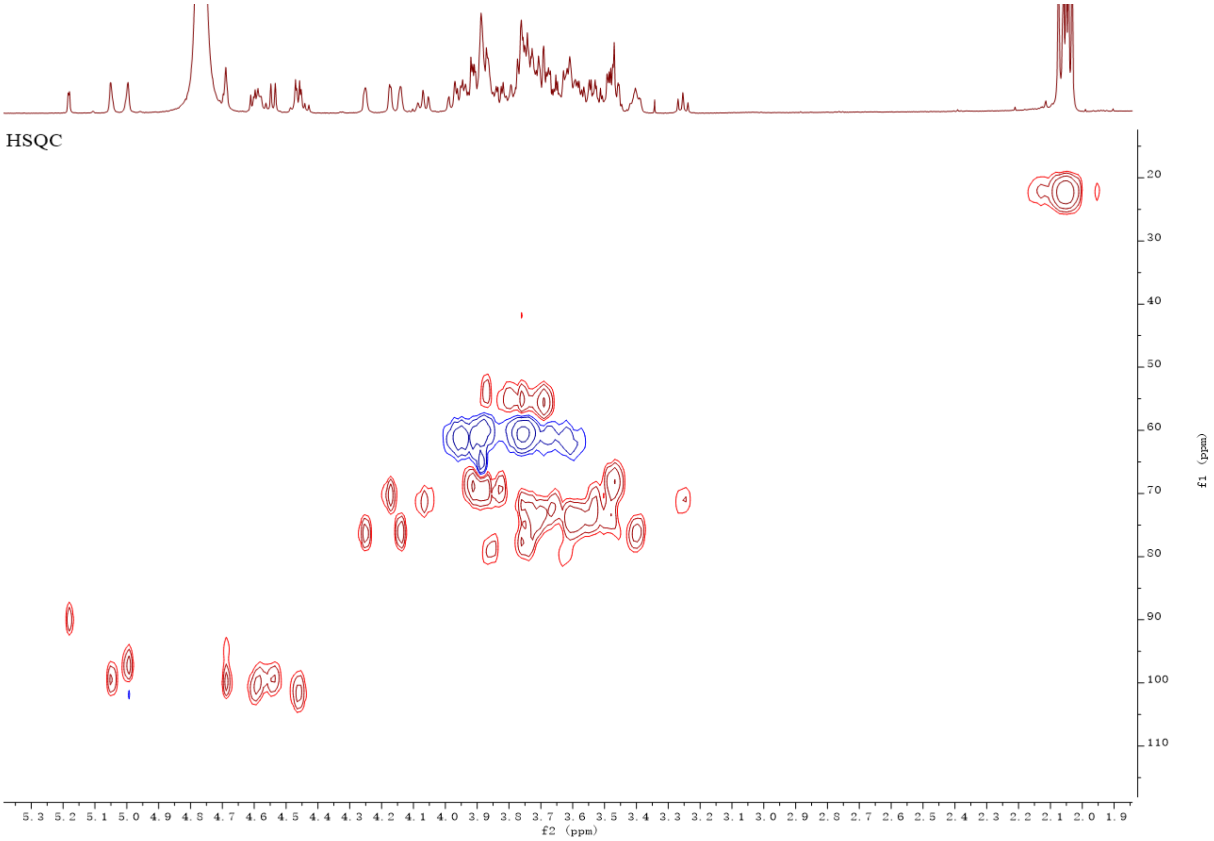
HSQC
f2 (ppm)
f1 (ppm)

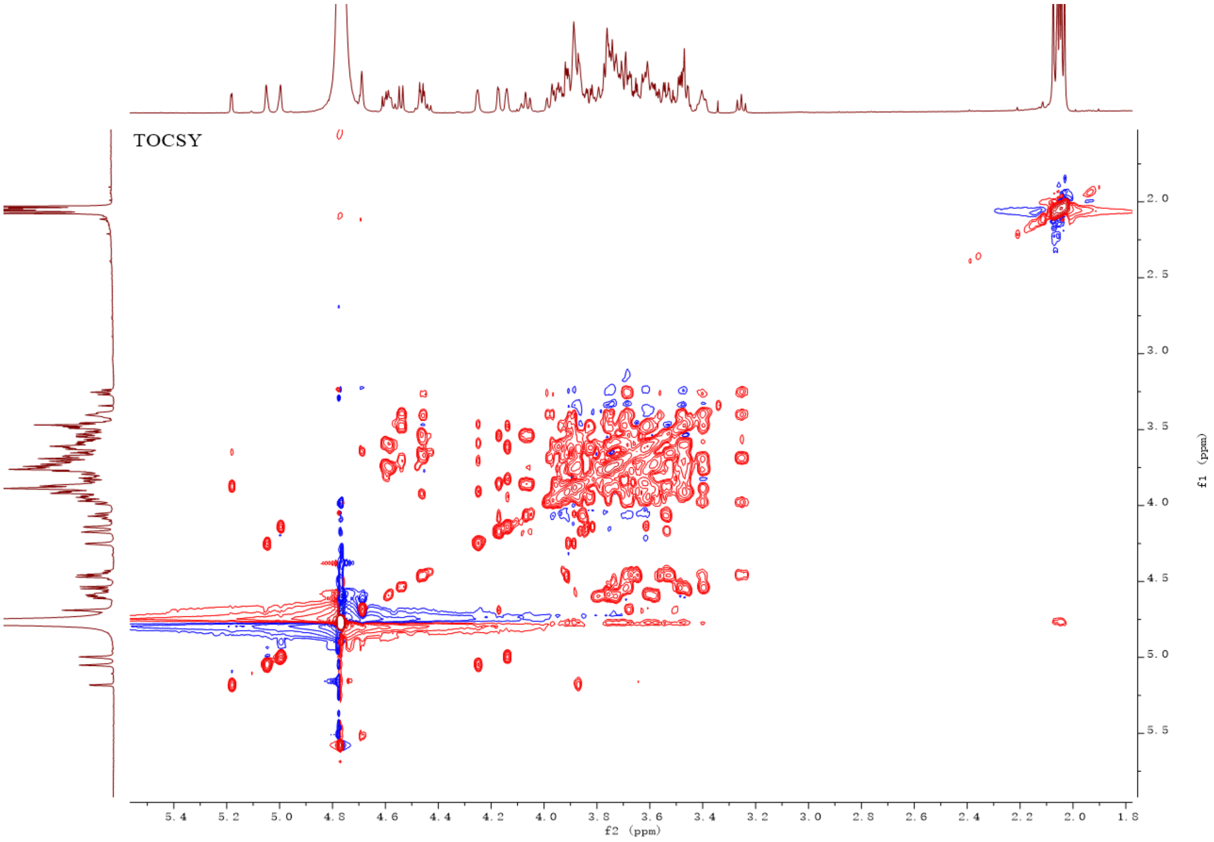
TOCSY
f2 (ppm)
f1 (ppm)

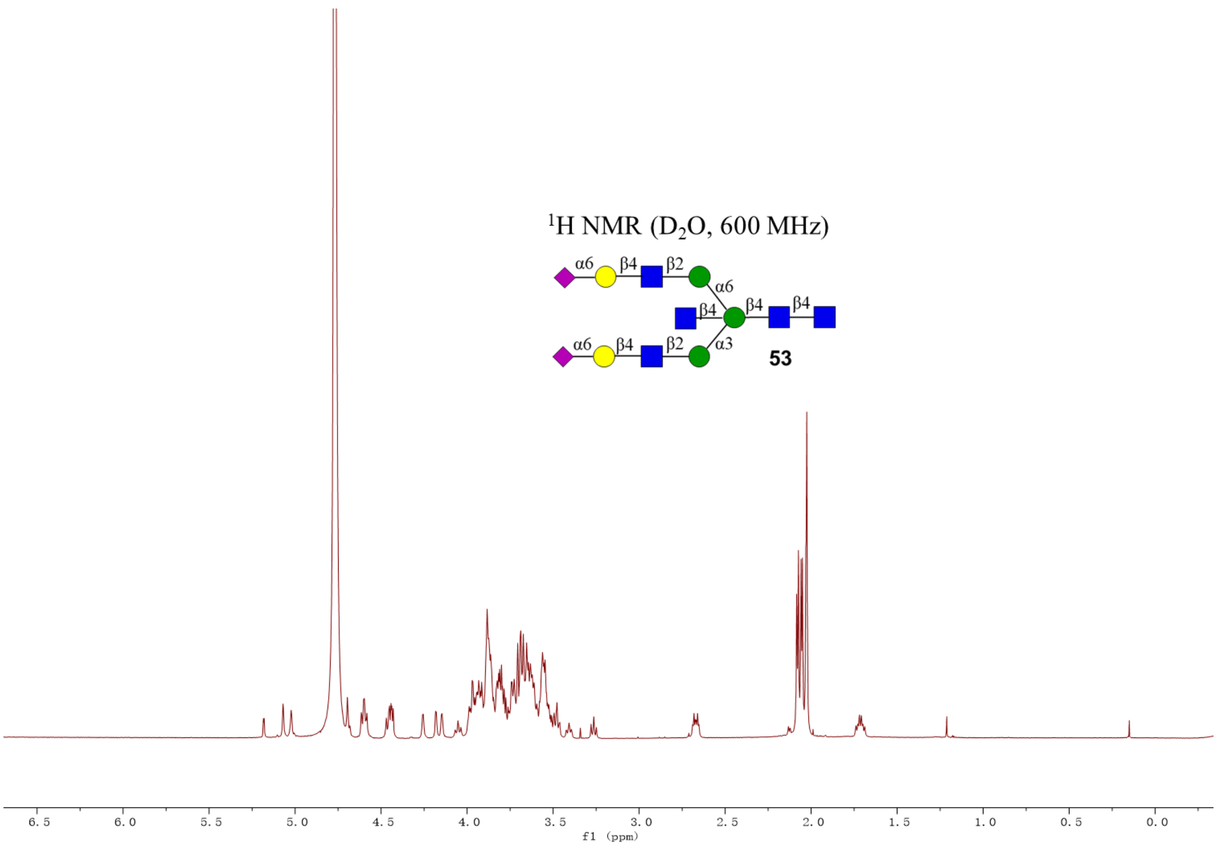
^{1}H NMR (D_2O, 600 MHz)
α6
β4
β2
α6
β4
β4
β4
α6
β4
β2
α3
53
f1 (ppm)

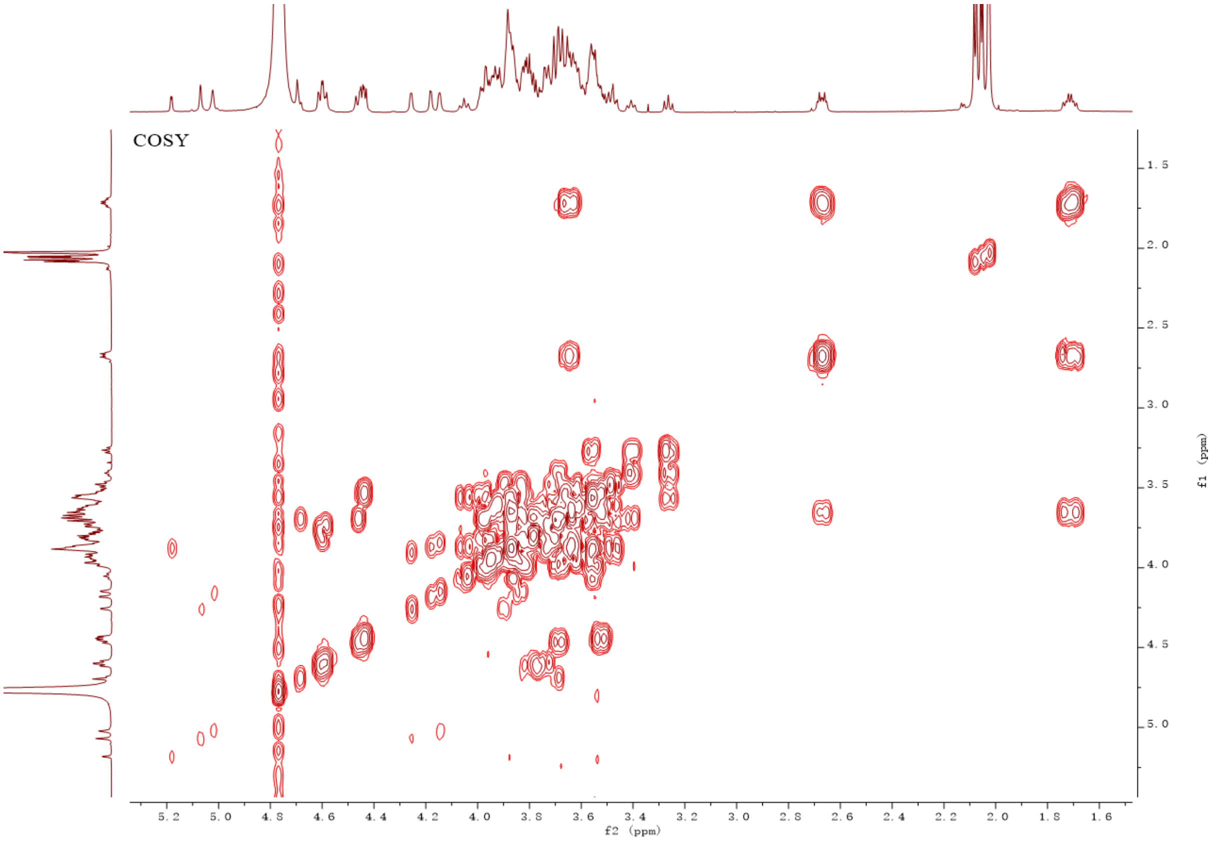
COSY
f2 (ppm)
f1 (ppm)

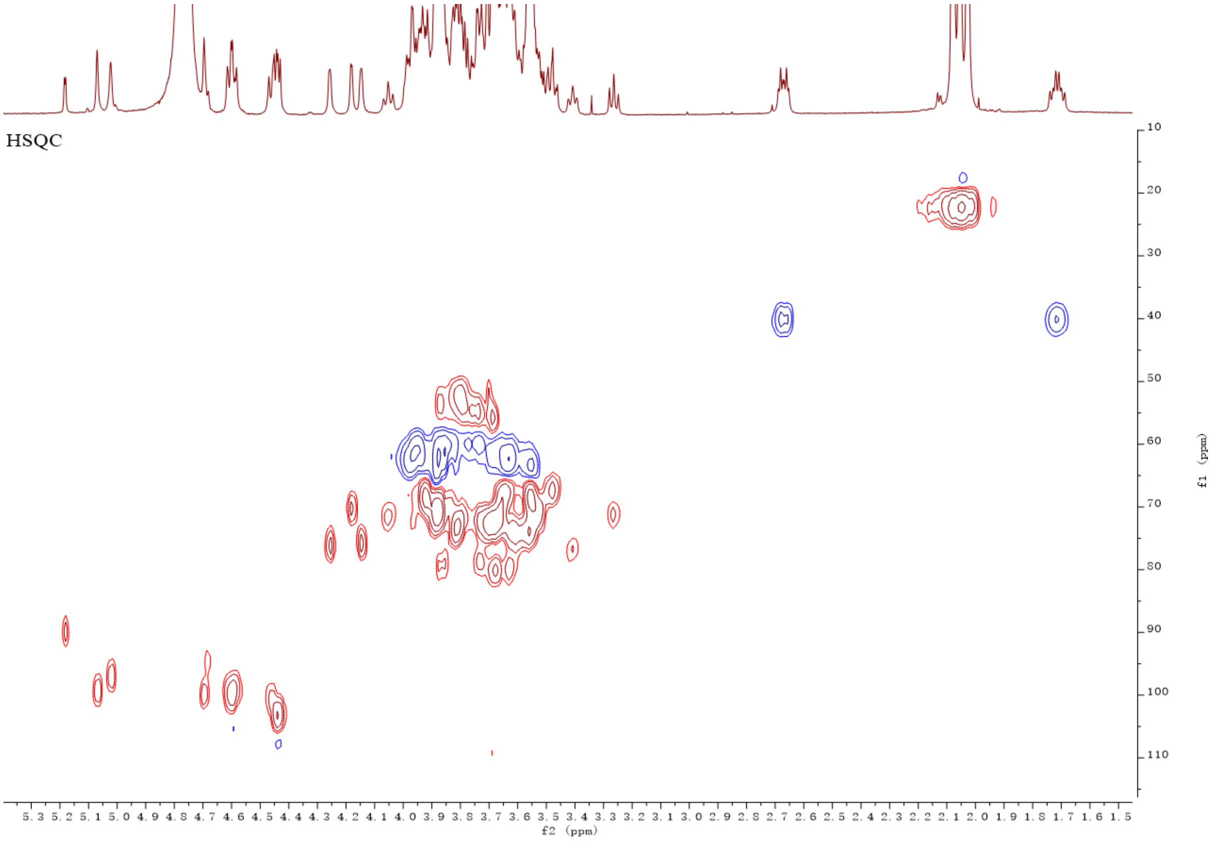
HSQC
f2 (ppm)
f1 (ppm)

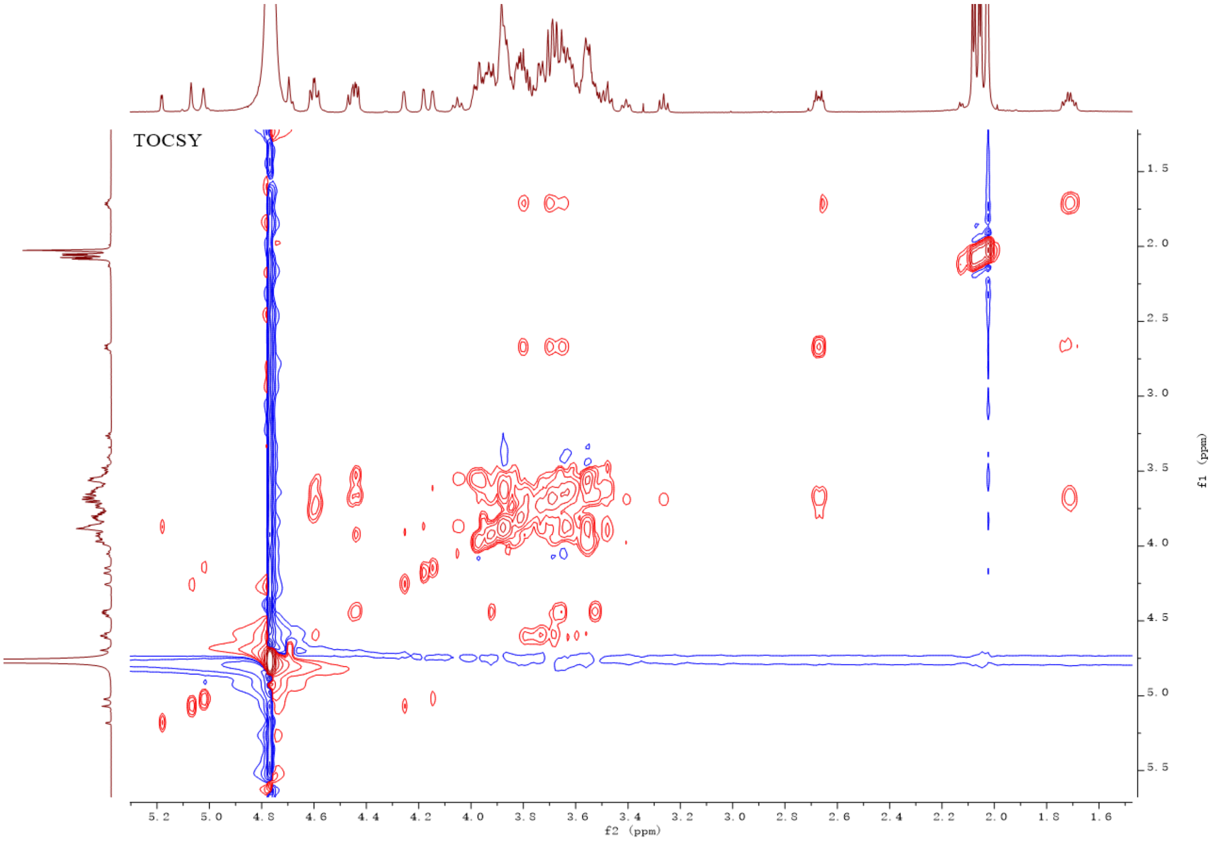
TOCSY
f2 (ppm)
f1 (ppm)

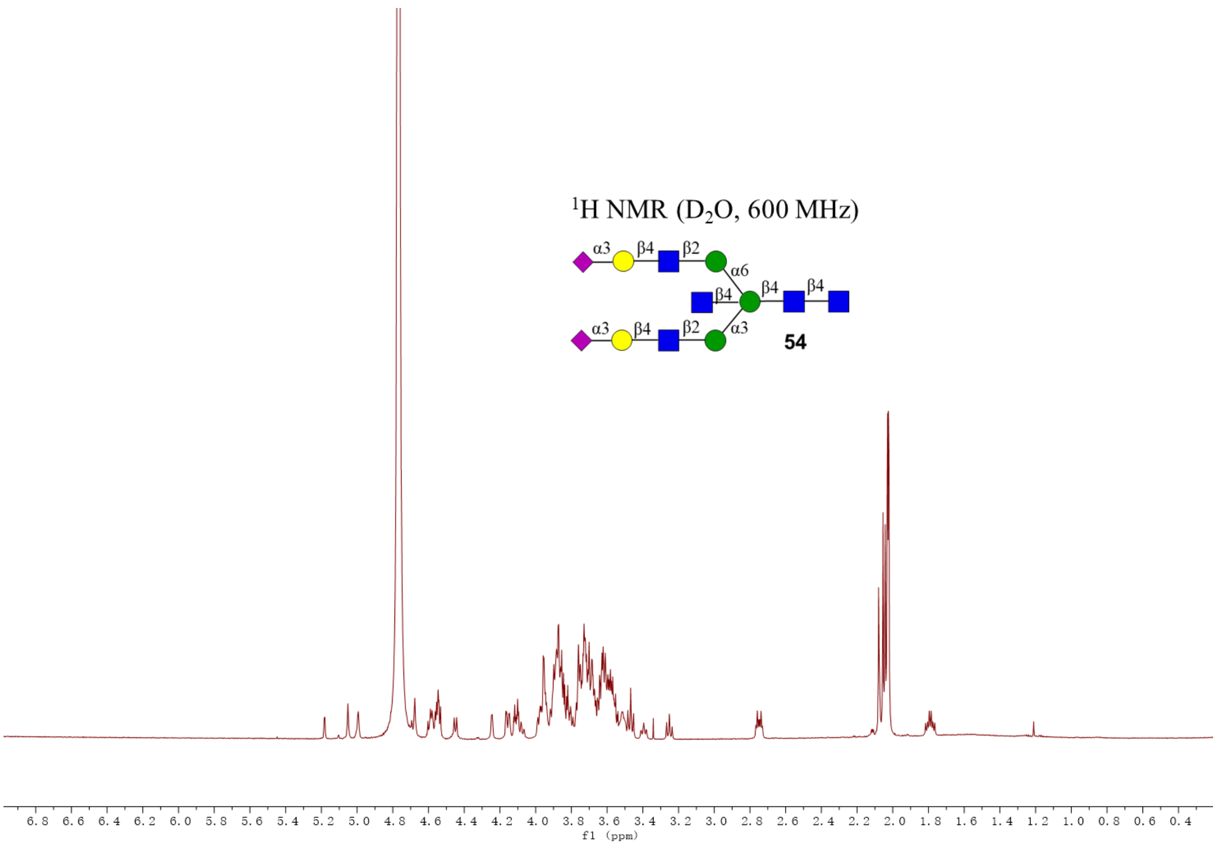
^{1}H NMR (D_2O, 600 MHz)
α3
β4
β2
α6
β4
β4
β4
α3
β4
β2
α3
54
f1 (ppm)

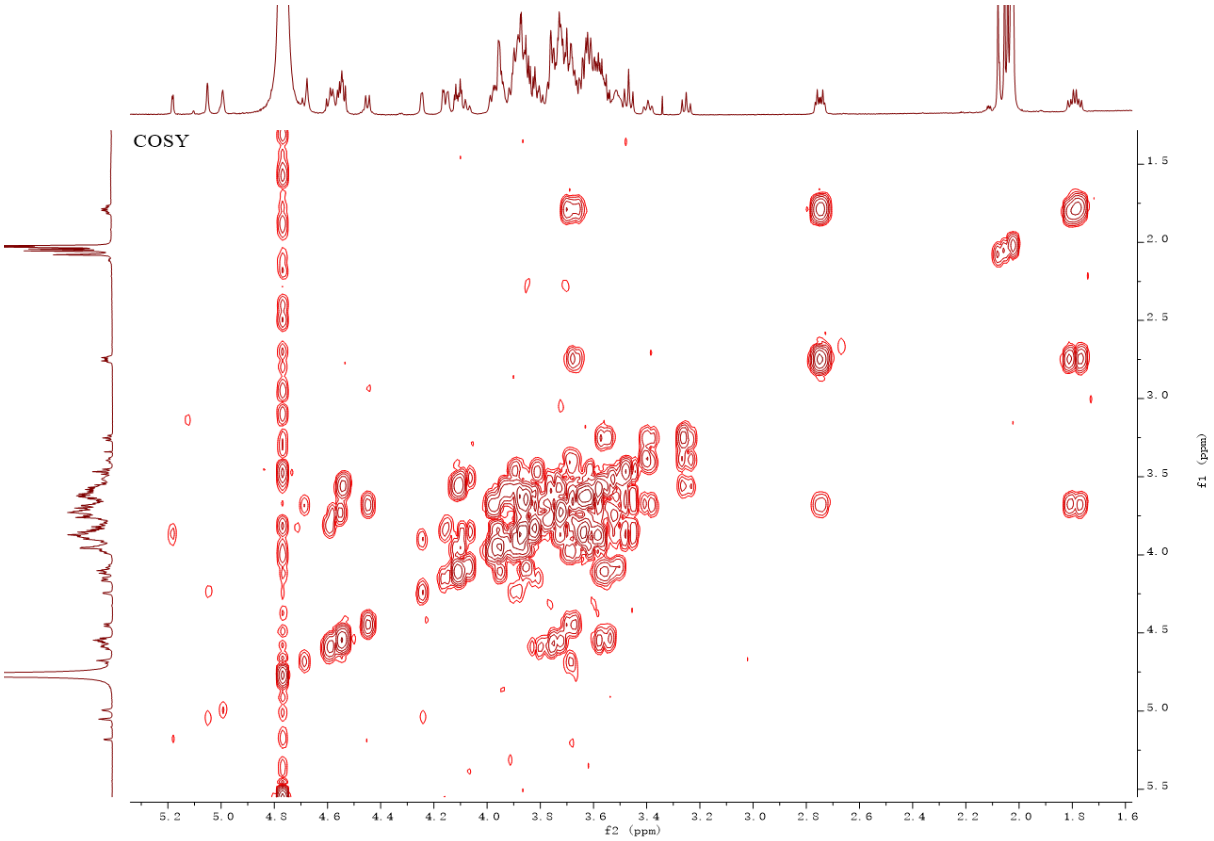
COSY
f2 (ppm)
f1 (ppm)

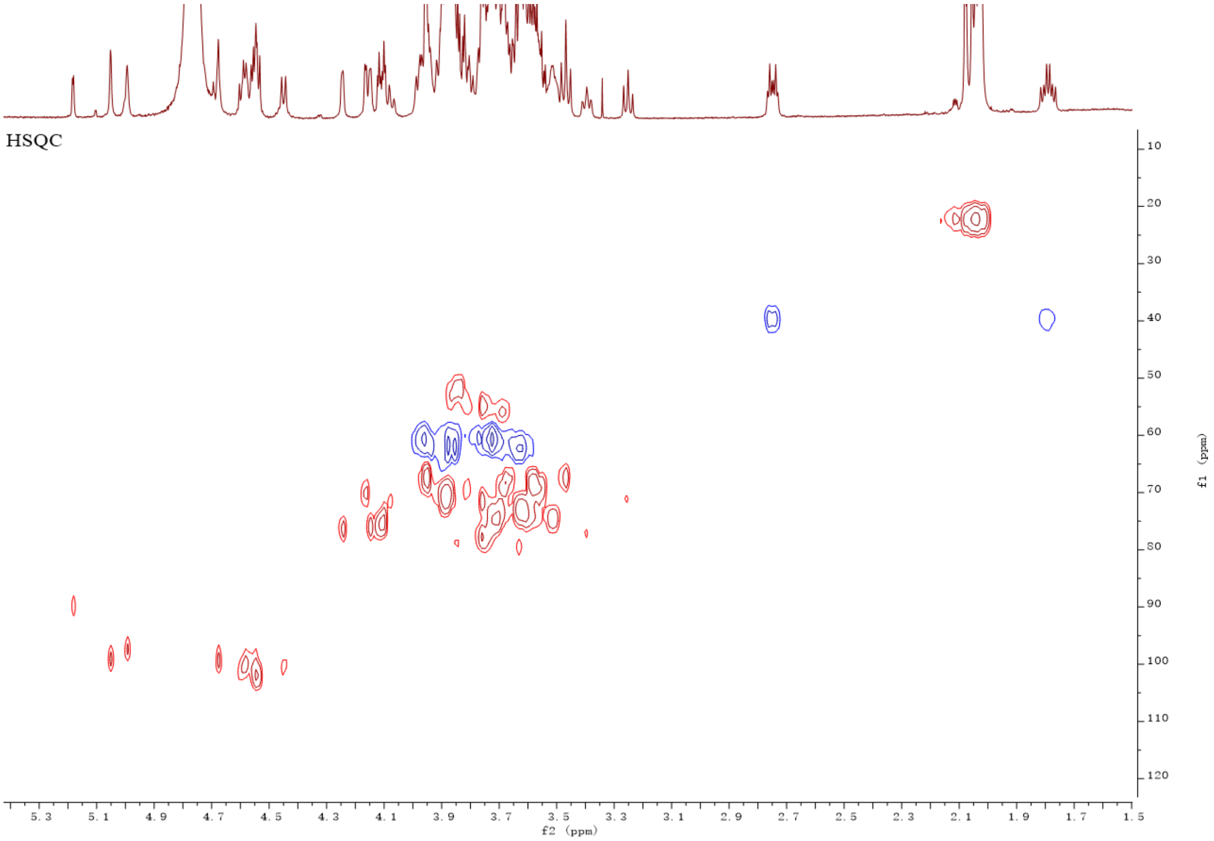
HSQC
f2 (ppm)
f1 (ppm)

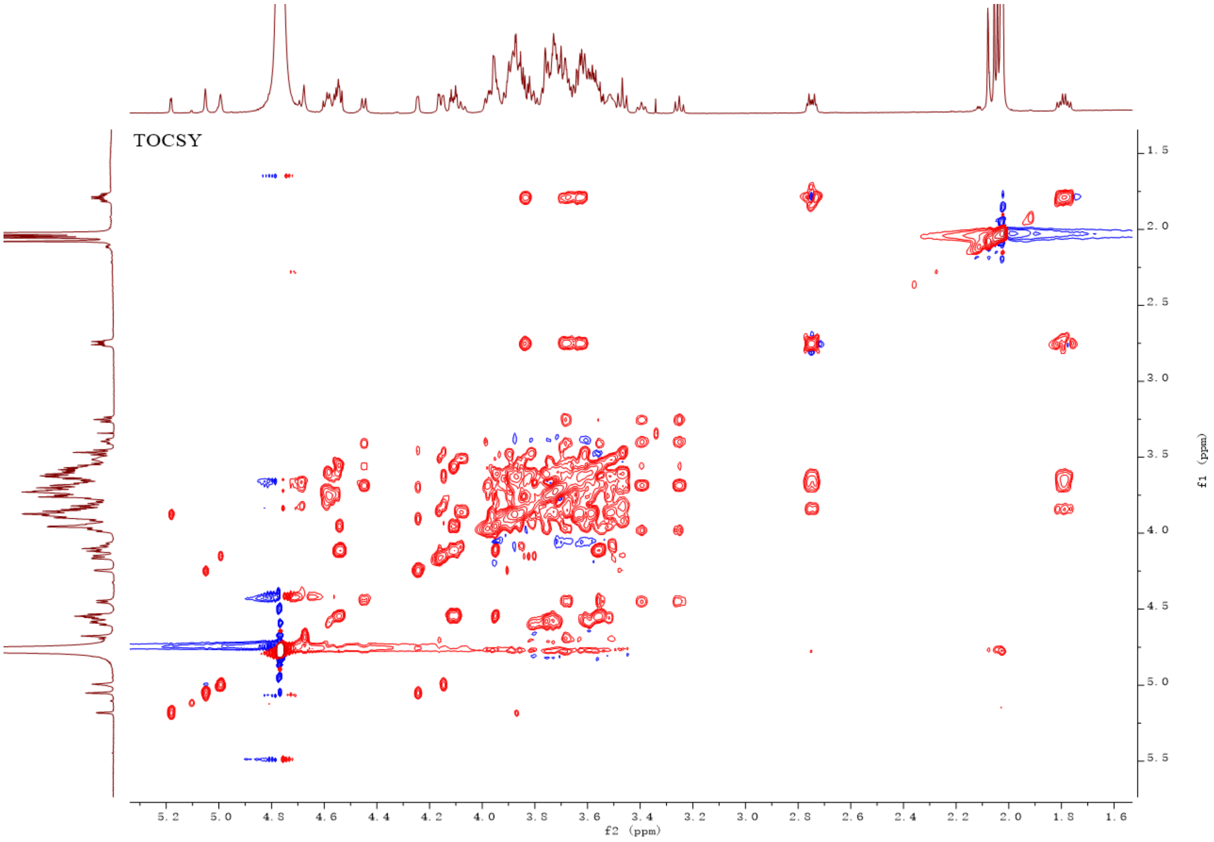
TOCSY
f2 (ppm)
f1 (ppm)

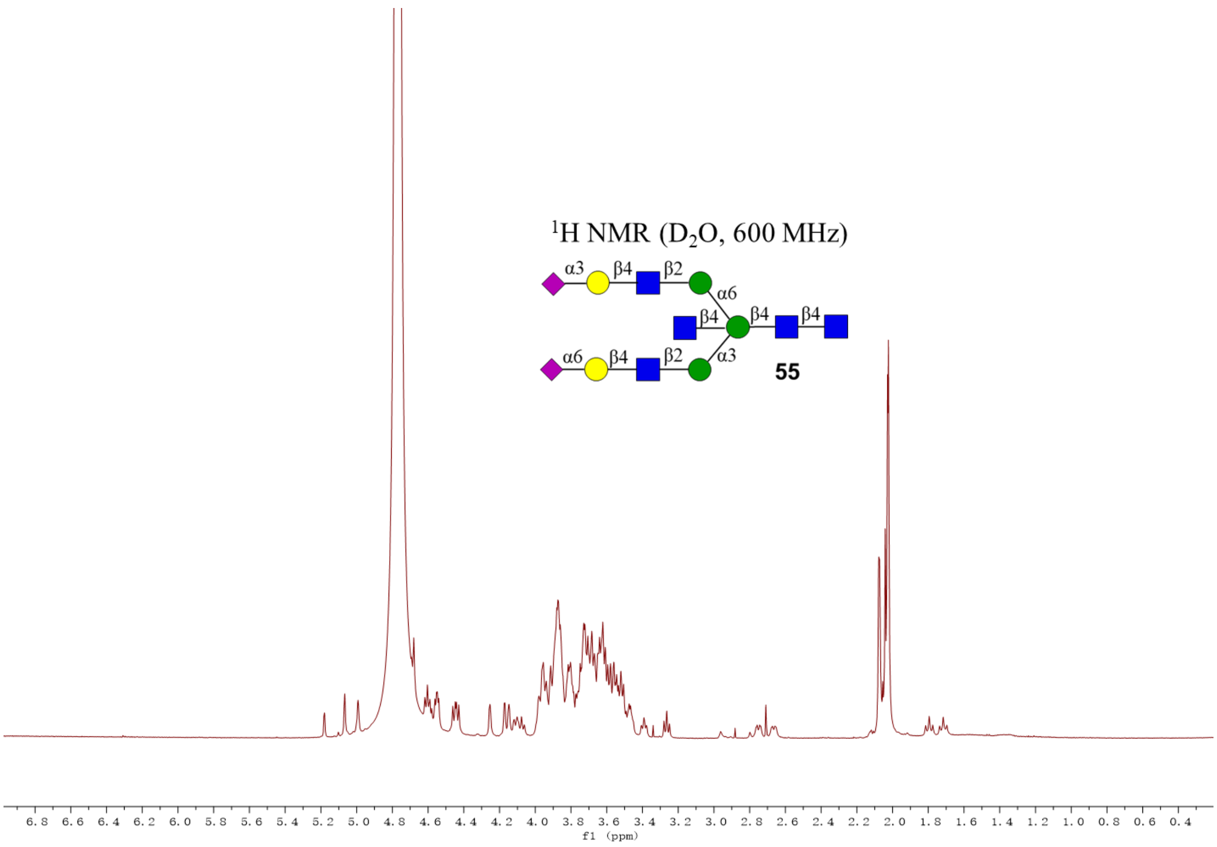

^{1}H NMR (D$_2$O, 600 MHz)
α3
β4
β2
α6
β4
β4
β4
α6
β4
β2
α3
55
f1 (ppm)

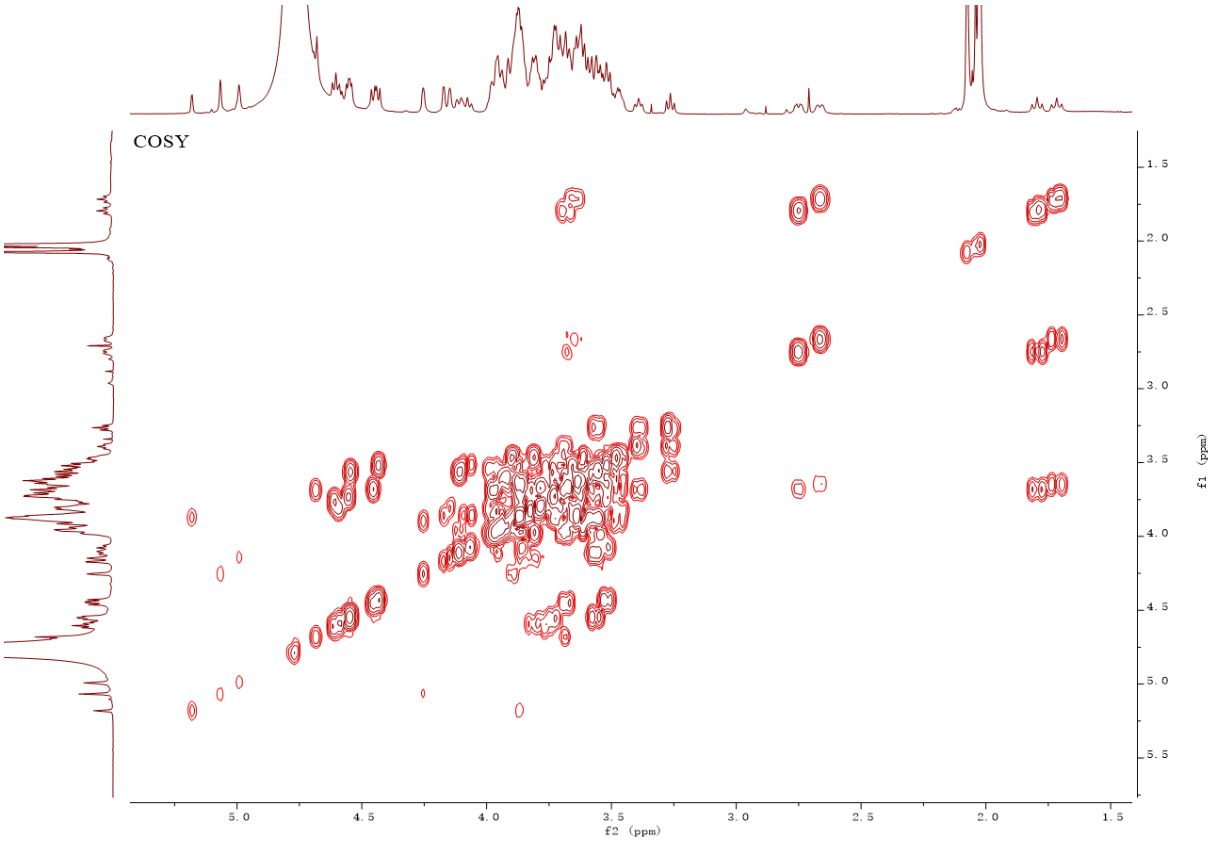

COSY
f2 (ppm)
f1 (ppm)

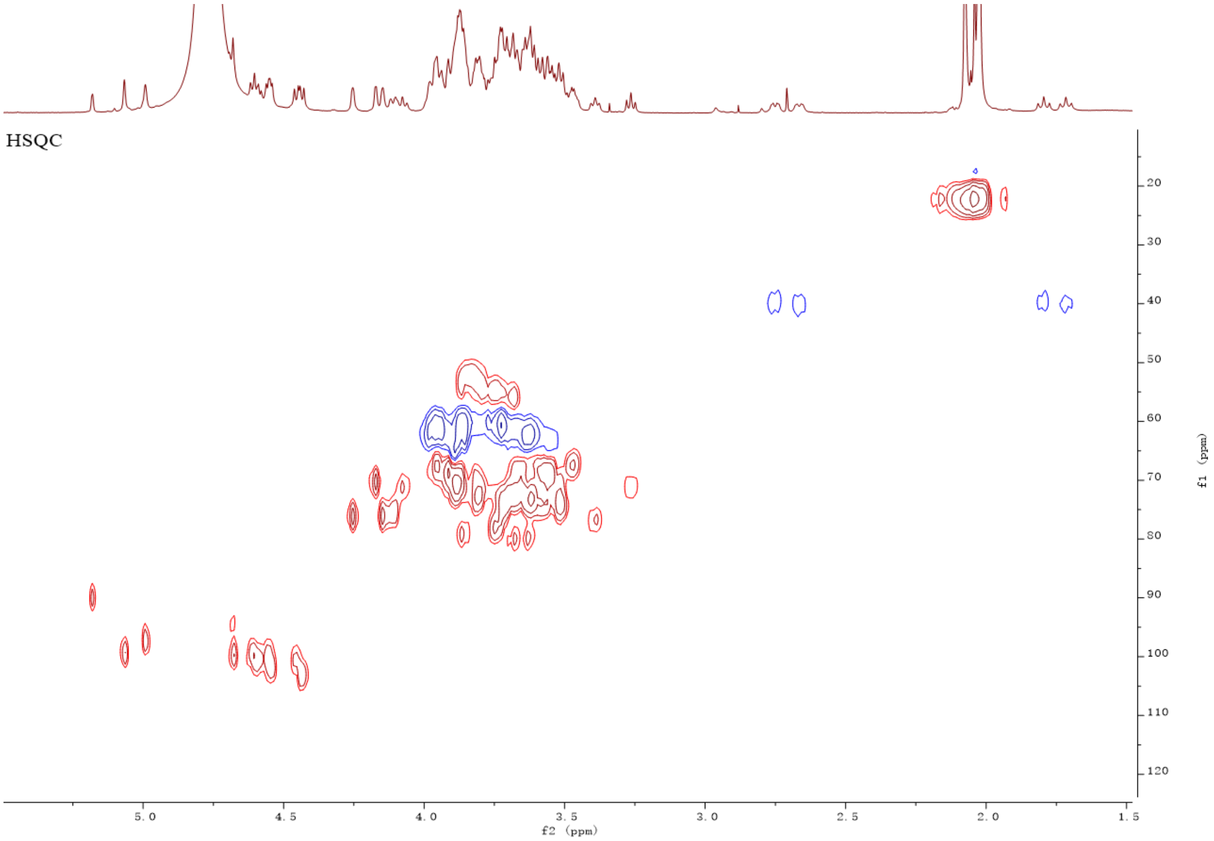
HSQC
f2 (ppm)
f1 (ppm)

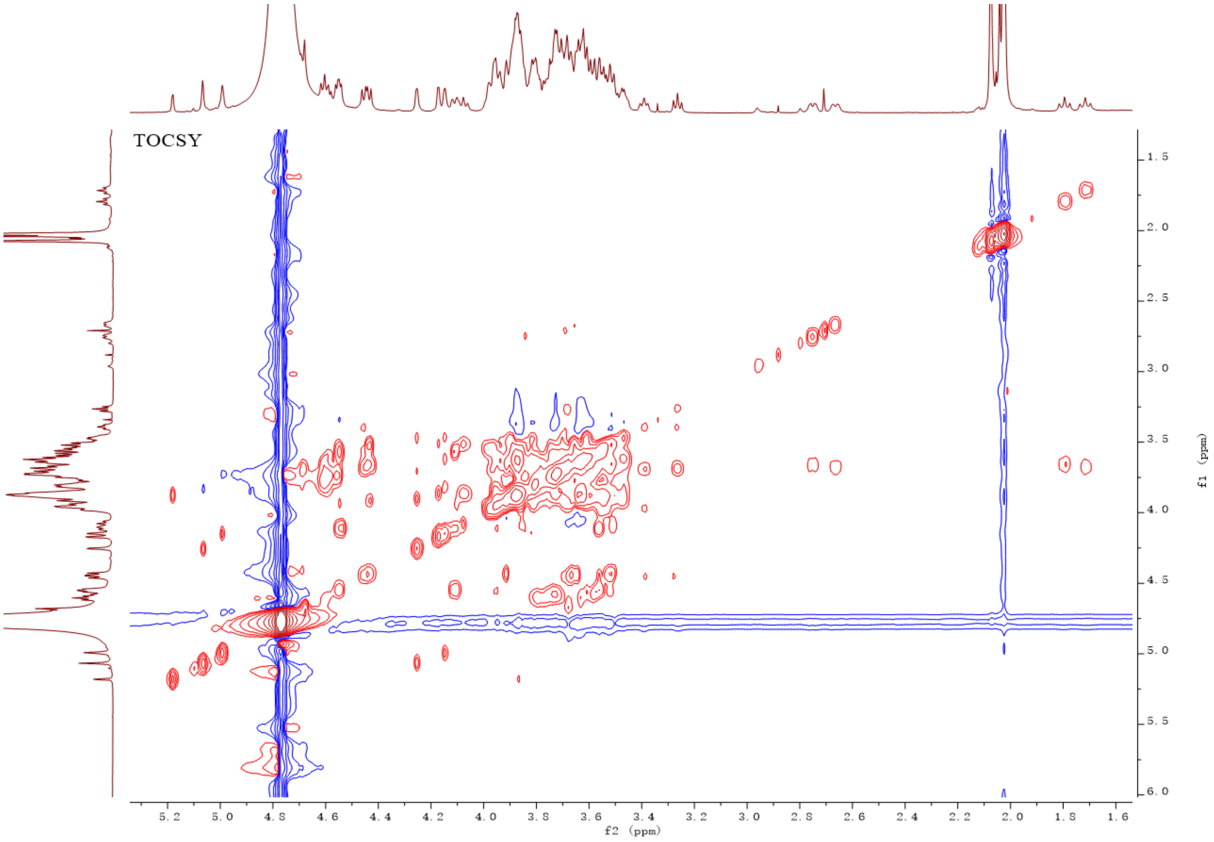
TOCSY
f2 (ppm)
f1 (ppm)

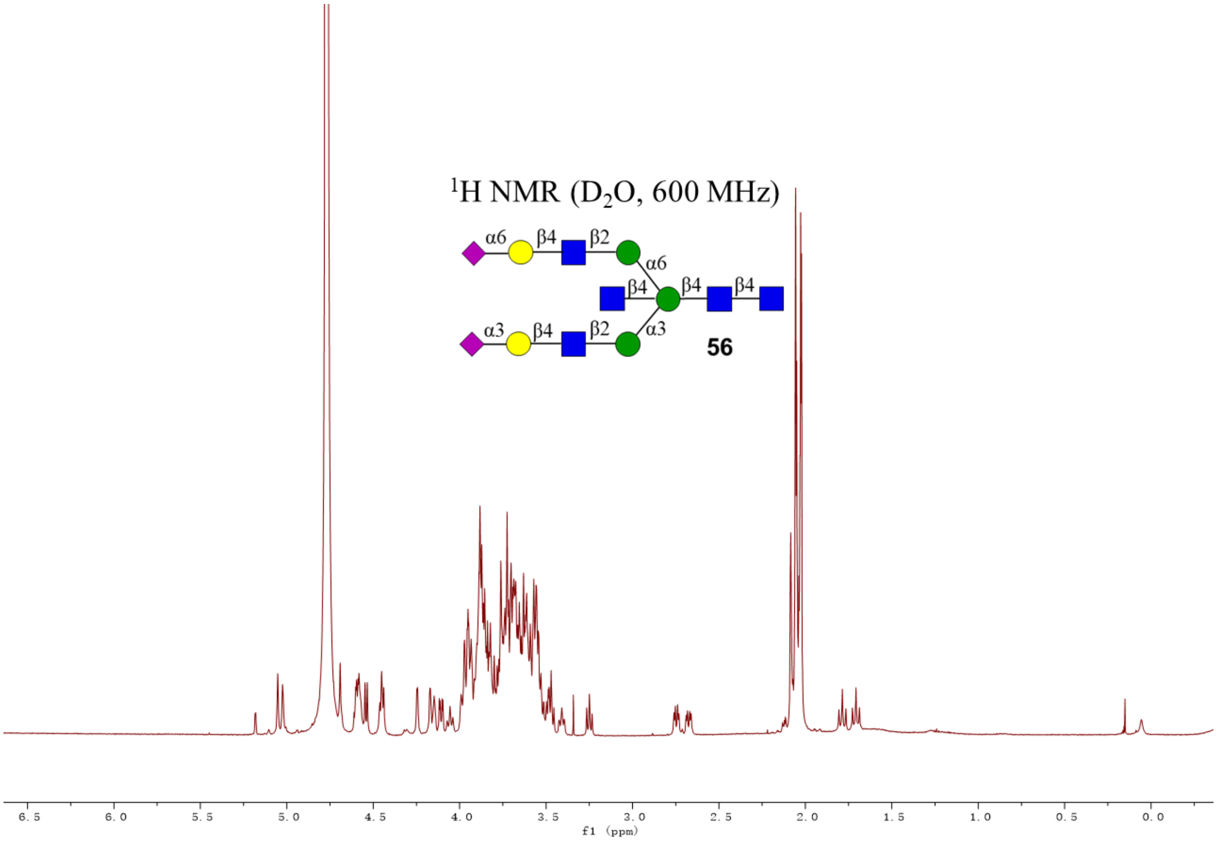
^{1}H NMR (D_2O, 600 MHz)
α6 β4 β2
α6
β4 β4 β4
α3 β4 β2 α3
56
6.5 6.0 5.5 5.0 4.5 4.0 3.5 3.0 2.5 2.0 1.5 1.0 0.5 0.0
f1 (ppm)

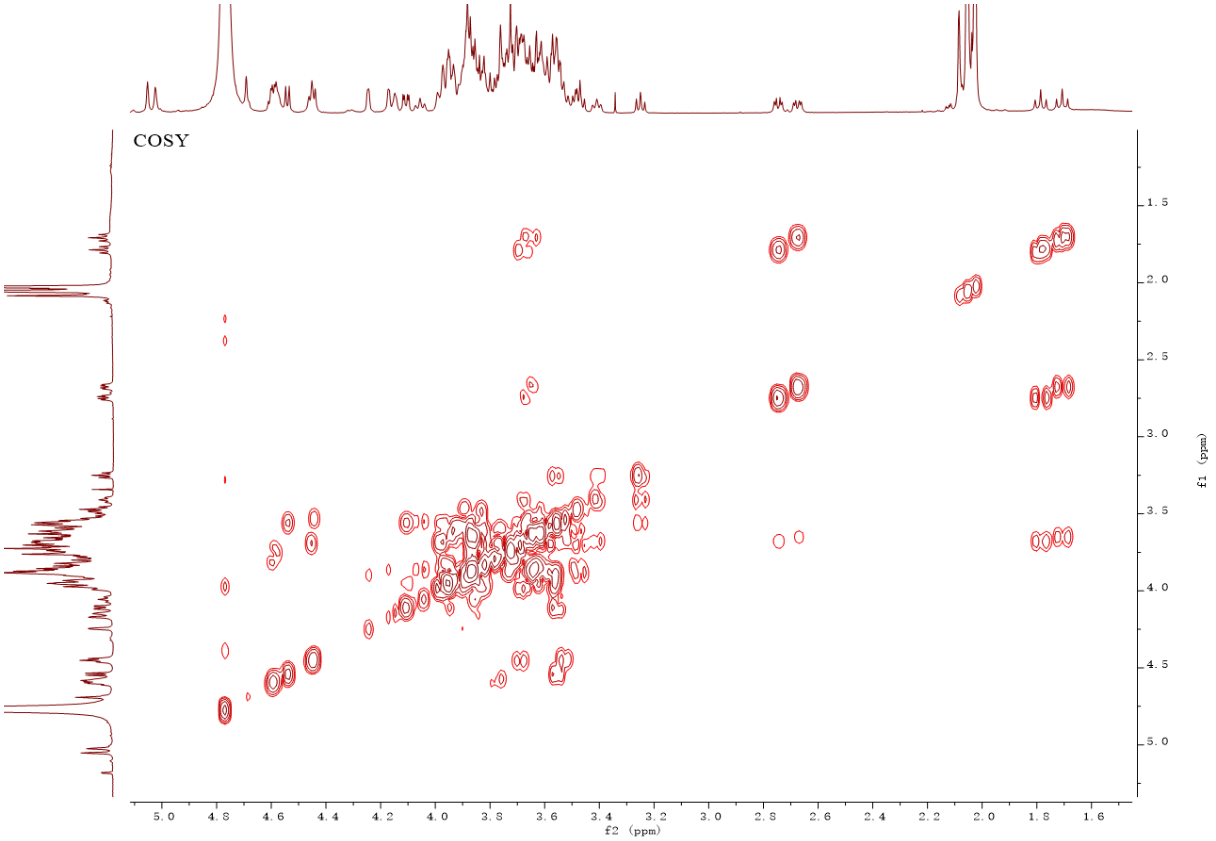
COSY
5.0 4.8 4.6 4.4 4.2 4.0 3.8 3.6 3.4 3.2 3.0 2.8 2.6 2.4 2.2 2.0 1.8 1.6
f2 (ppm)
1.5 2.0 2.5 3.0 3.5 4.0 4.5 5.0
f1 (ppm)

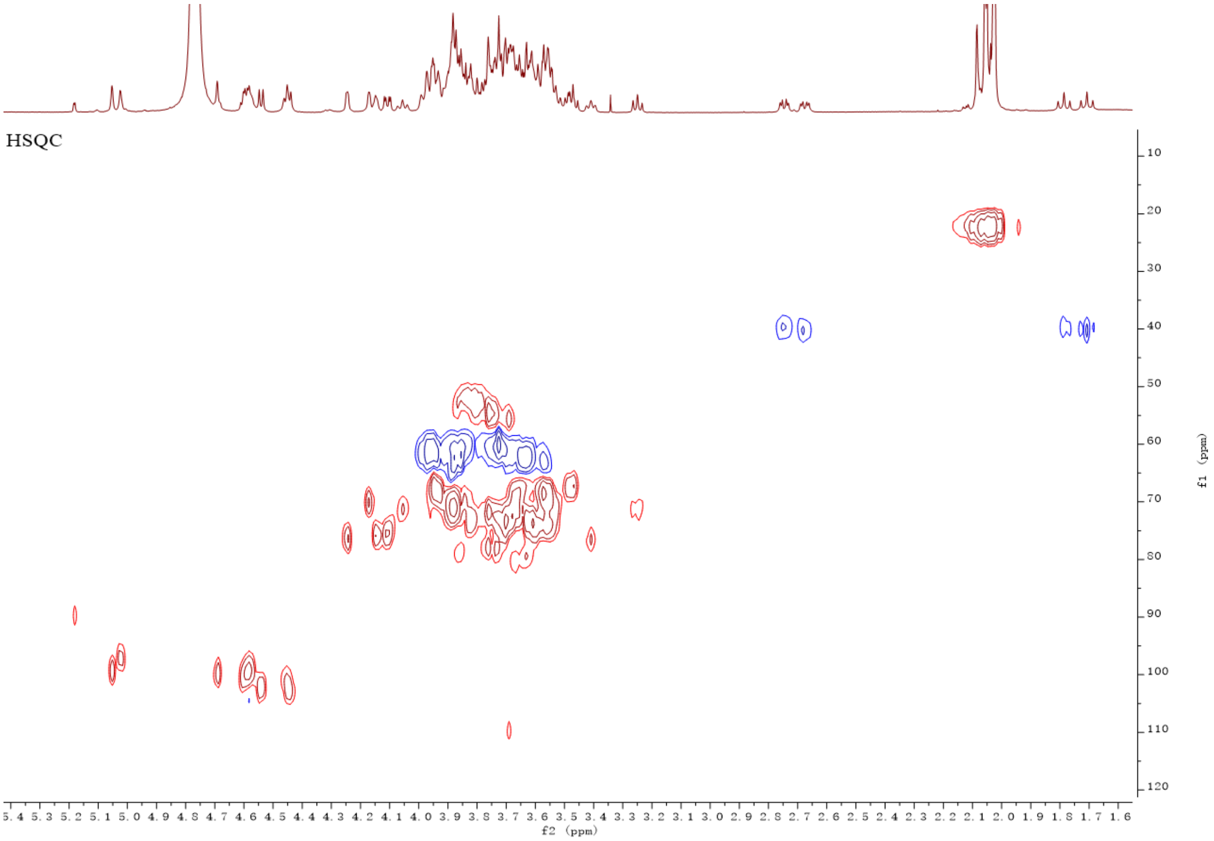
HSQC
f2 (ppm)
f1 (ppm)

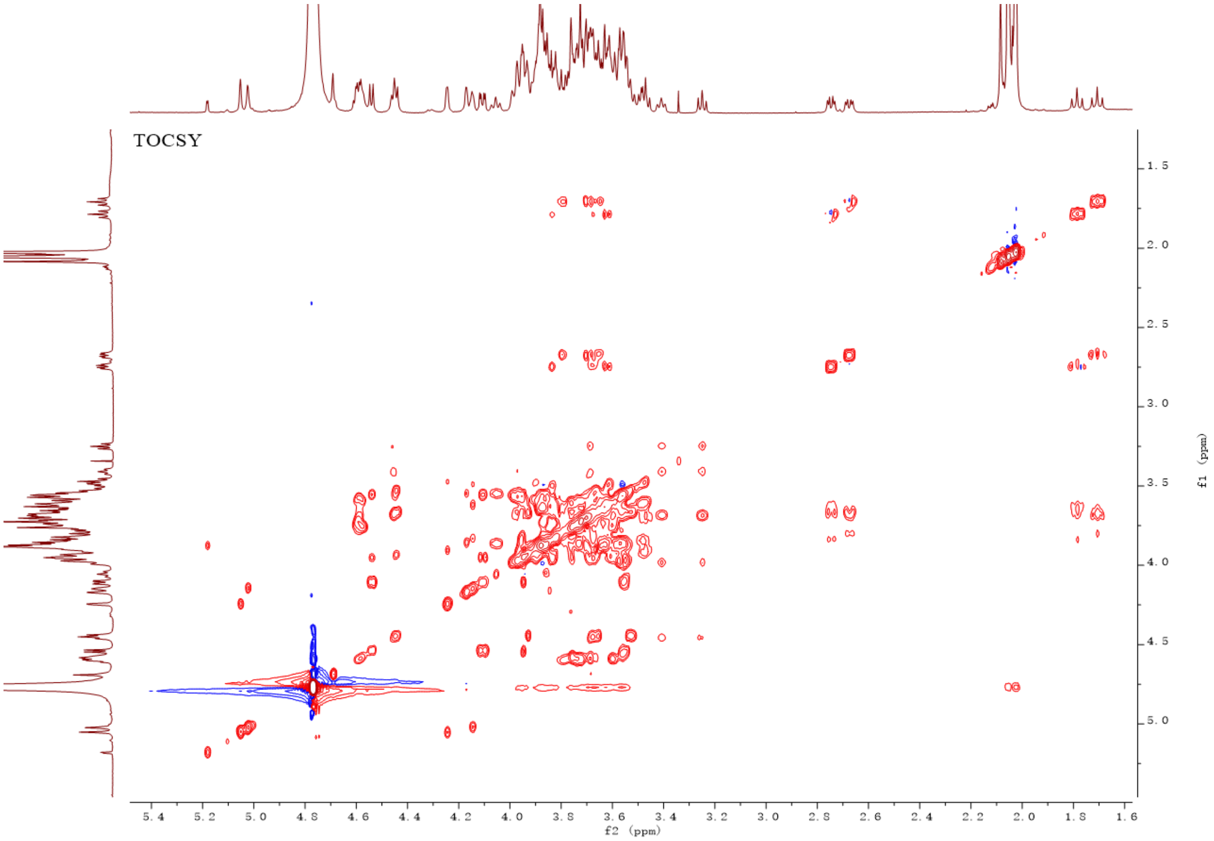
TOCSY
f2 (ppm)
f1 (ppm)

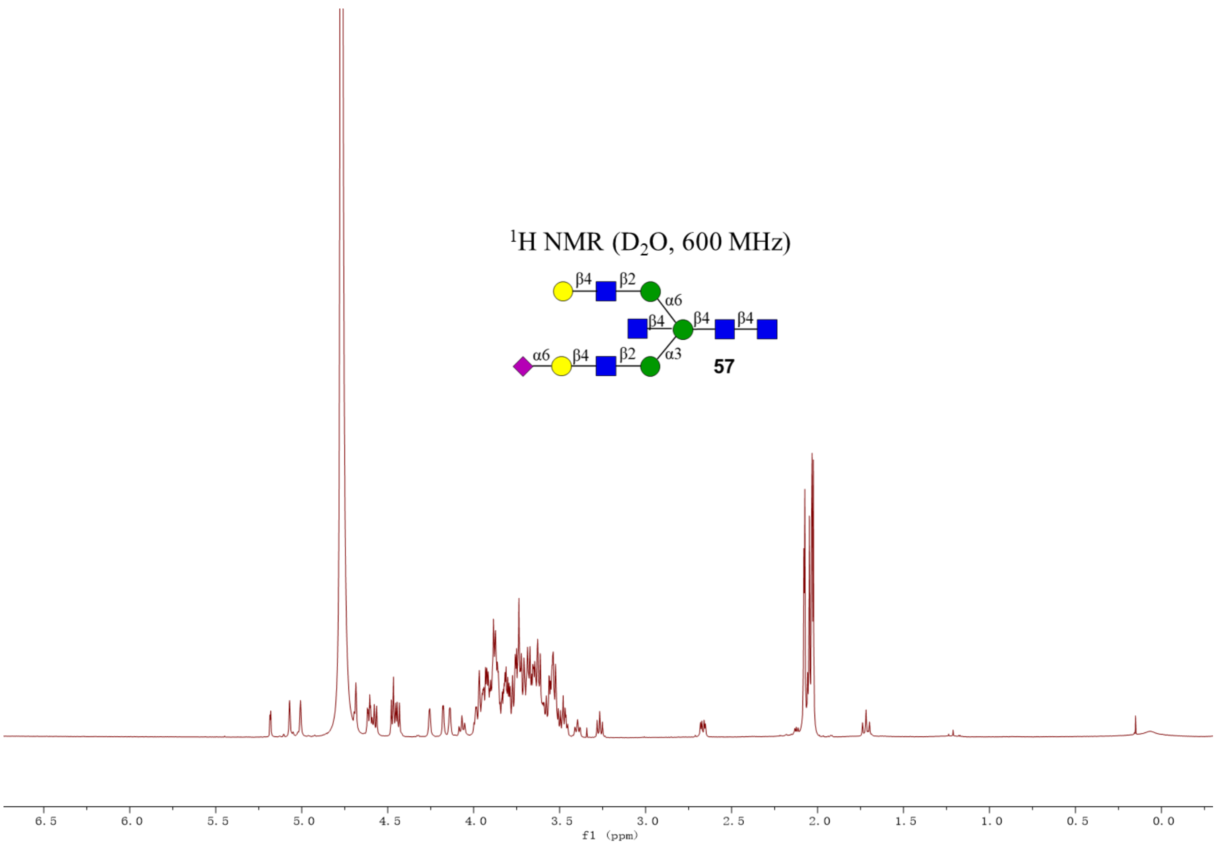

^{1}H NMR (D_2O, 600 MHz)
57
f1 (ppm)

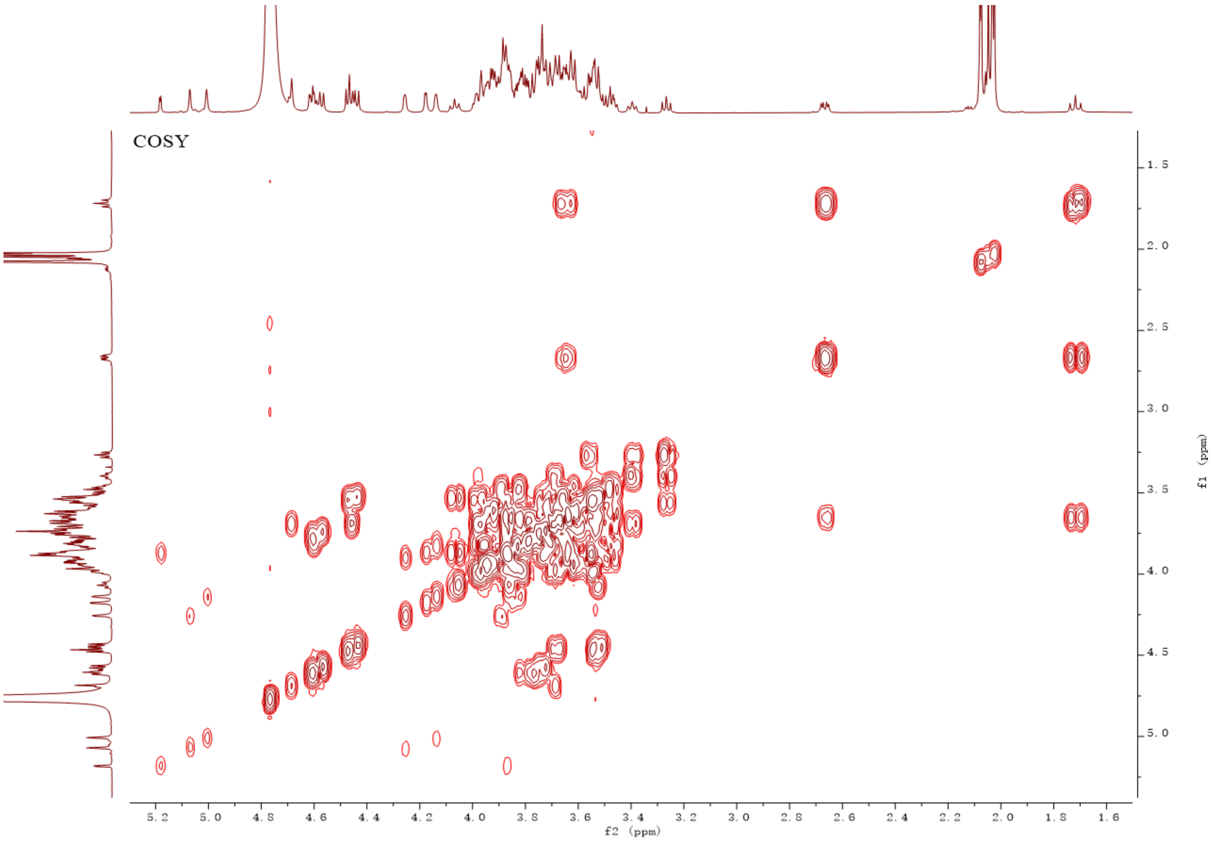

COSY
f2 (ppm)
f1 (ppm)

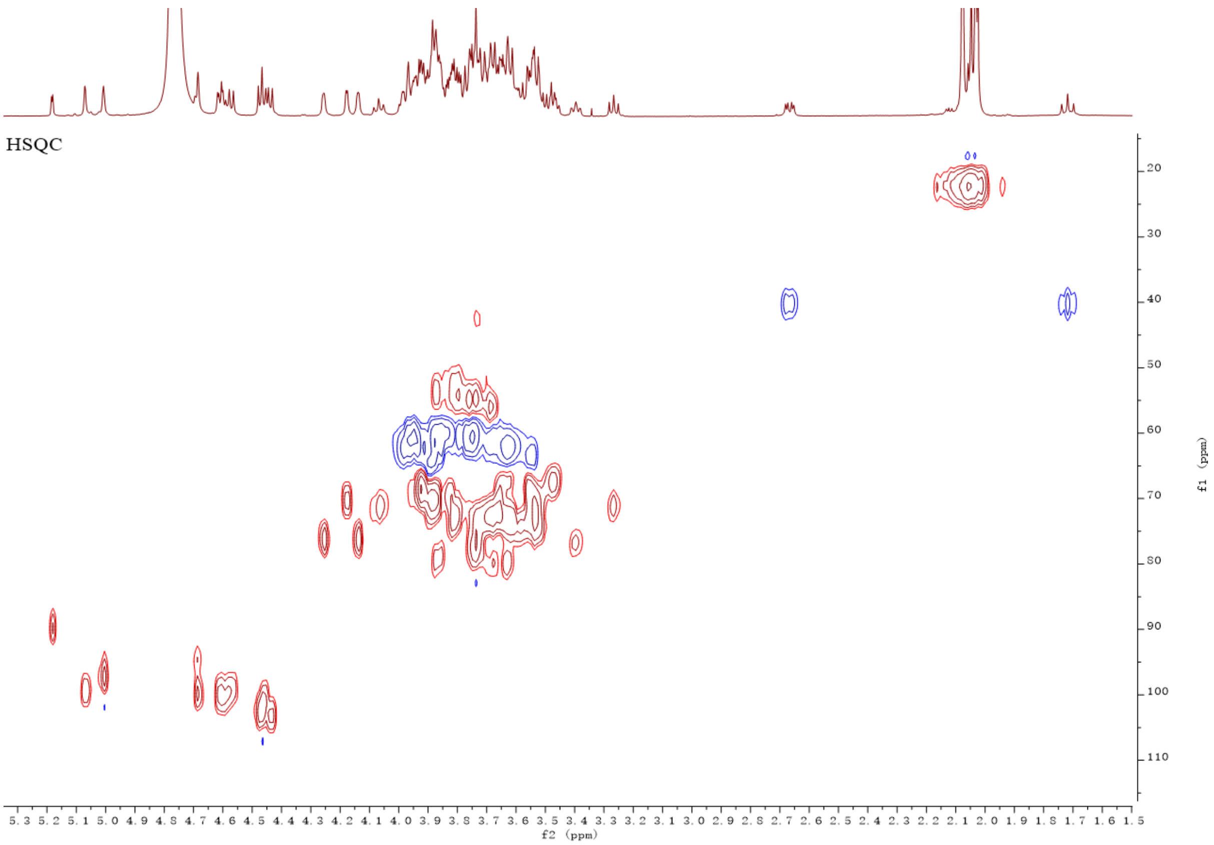
HSQC
f2 (ppm)
f1 (ppm)

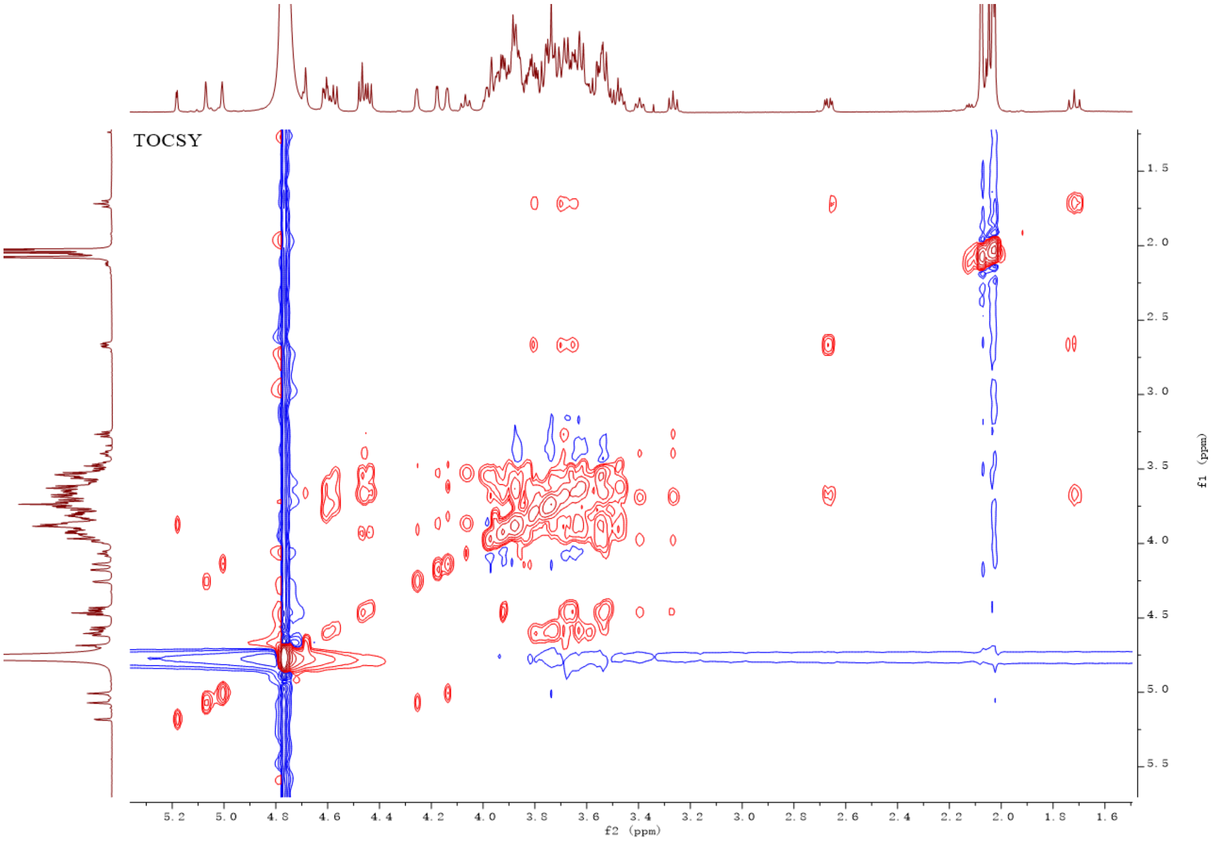
TOCSY
f2 (ppm)
f1 (ppm)

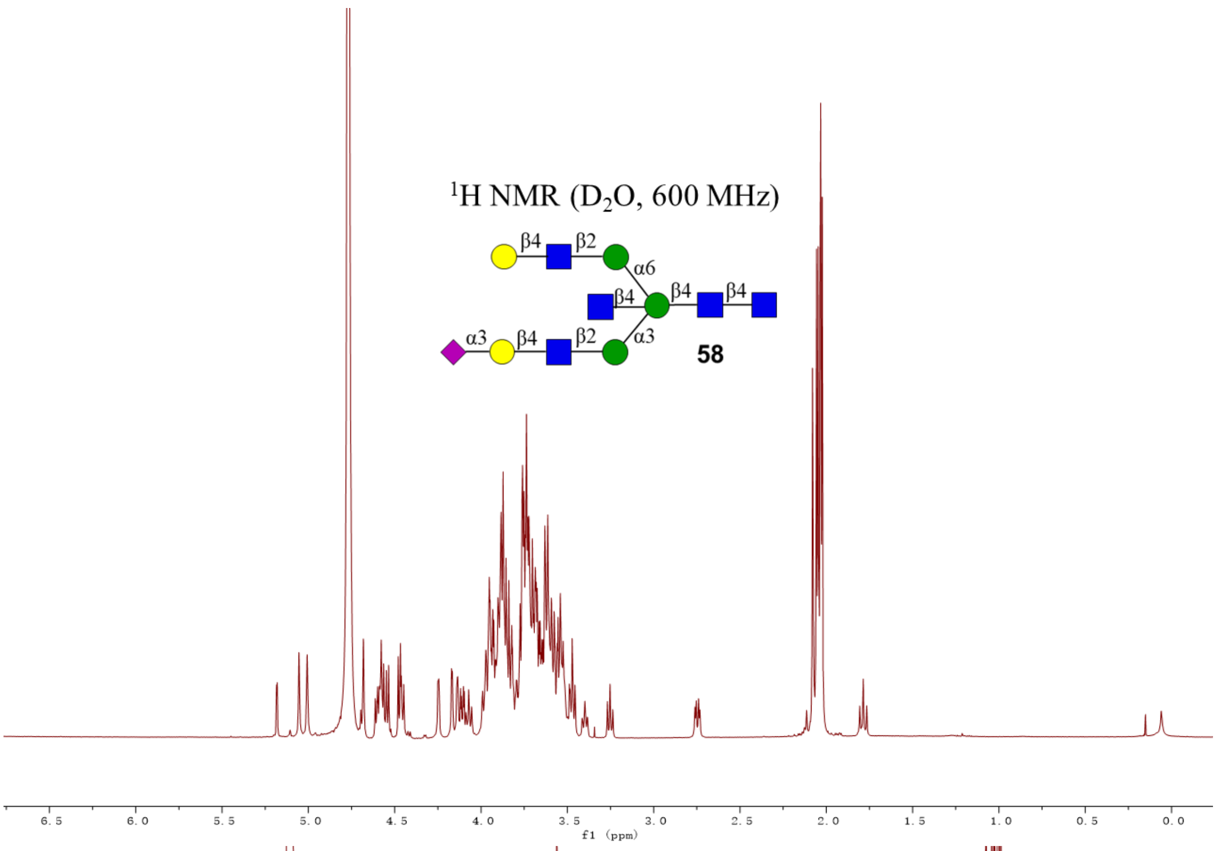

¹H NMR (D₂O, 600 MHz)
β4
β2
α6
β4
β4
β4
α3
β4
β2
α3
58
f1 (ppm)

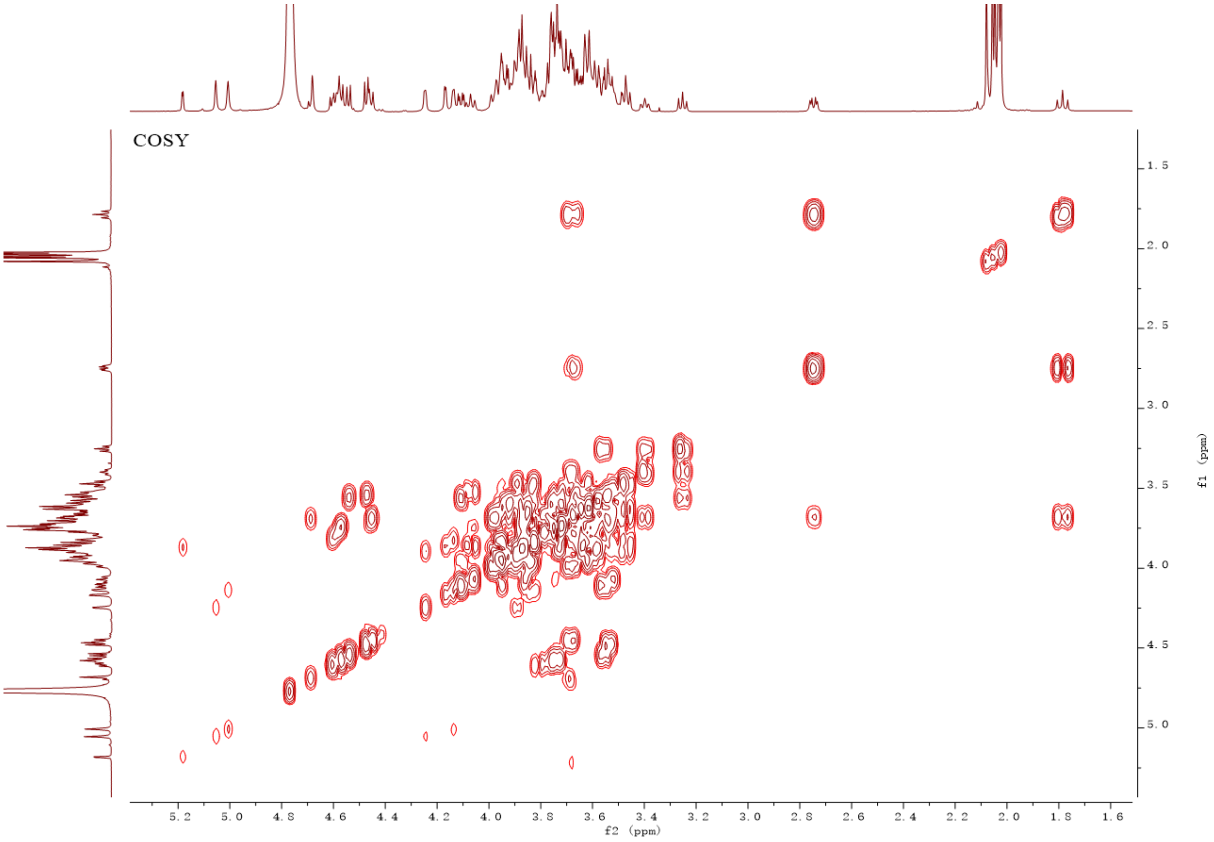

COSY
f2 (ppm)
f1 (ppm)

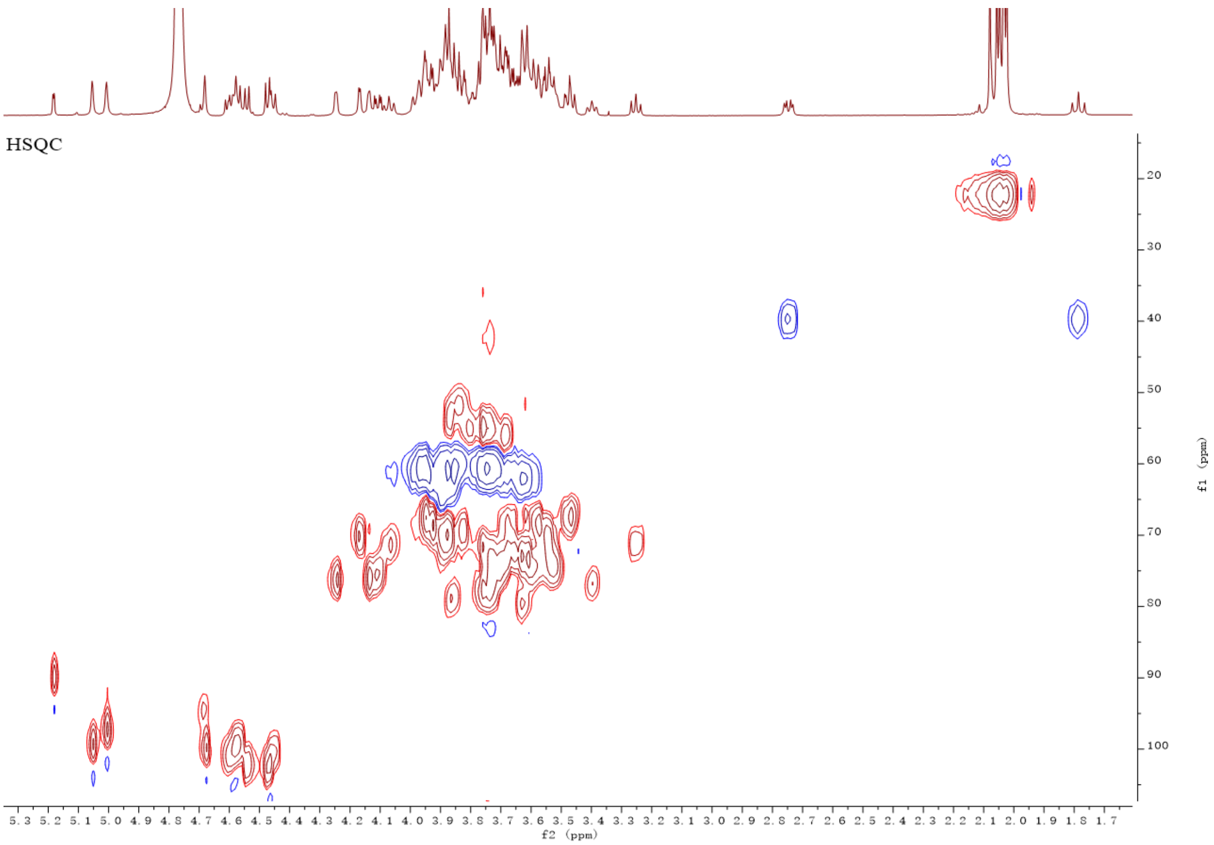
HSQC
f2 (ppm)
f1 (ppm)

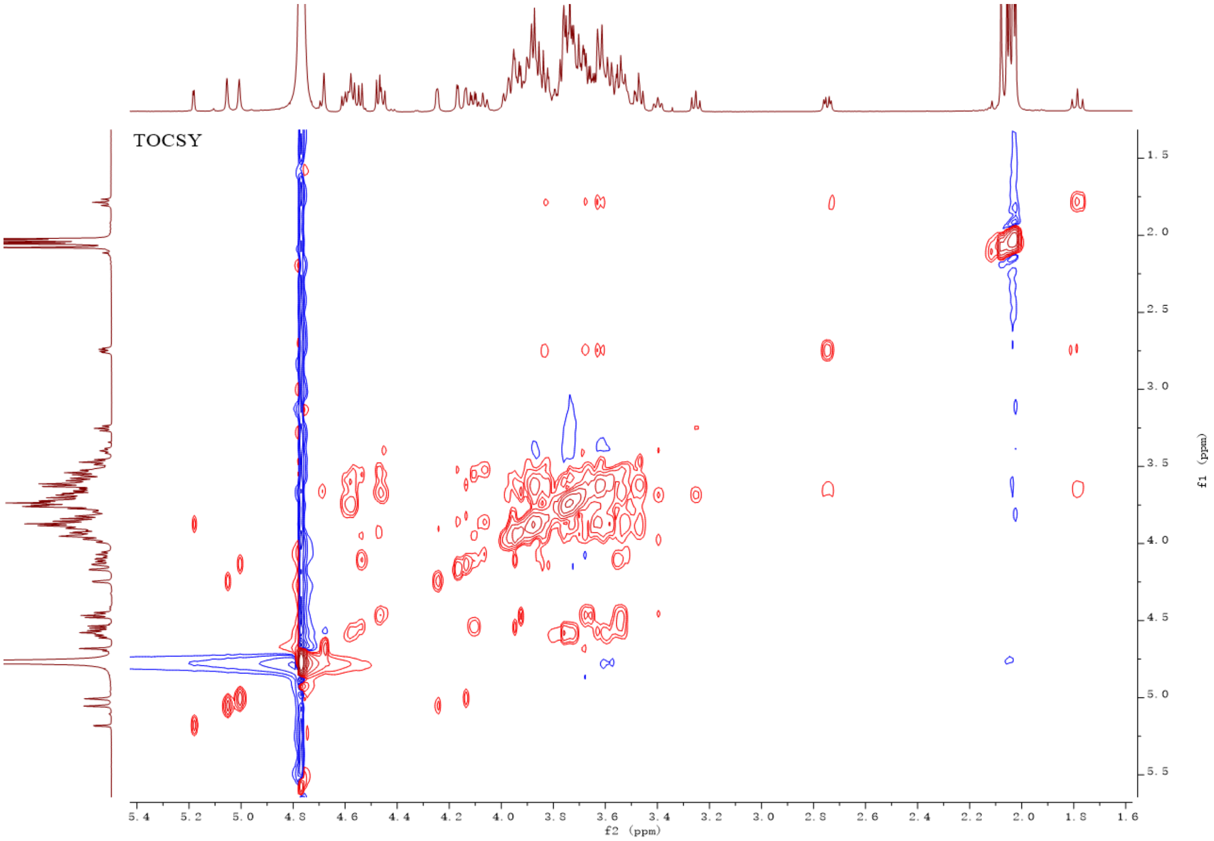
TOCSY
f2 (ppm)
f1 (ppm)

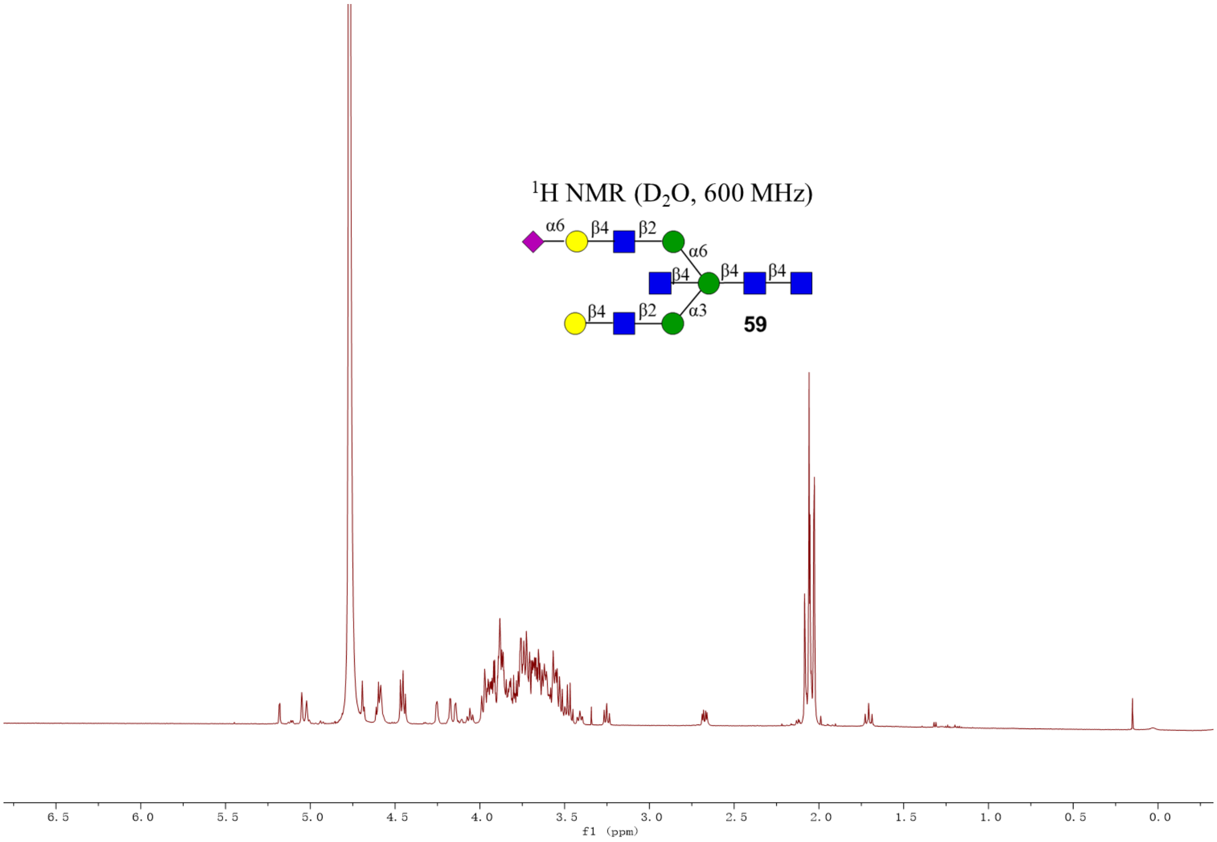

^1H NMR (D_2O, 600 MHz)
α6
β4
β2
α6
β4
β4
β4
β4
β2
α3
59
f1 (ppm)

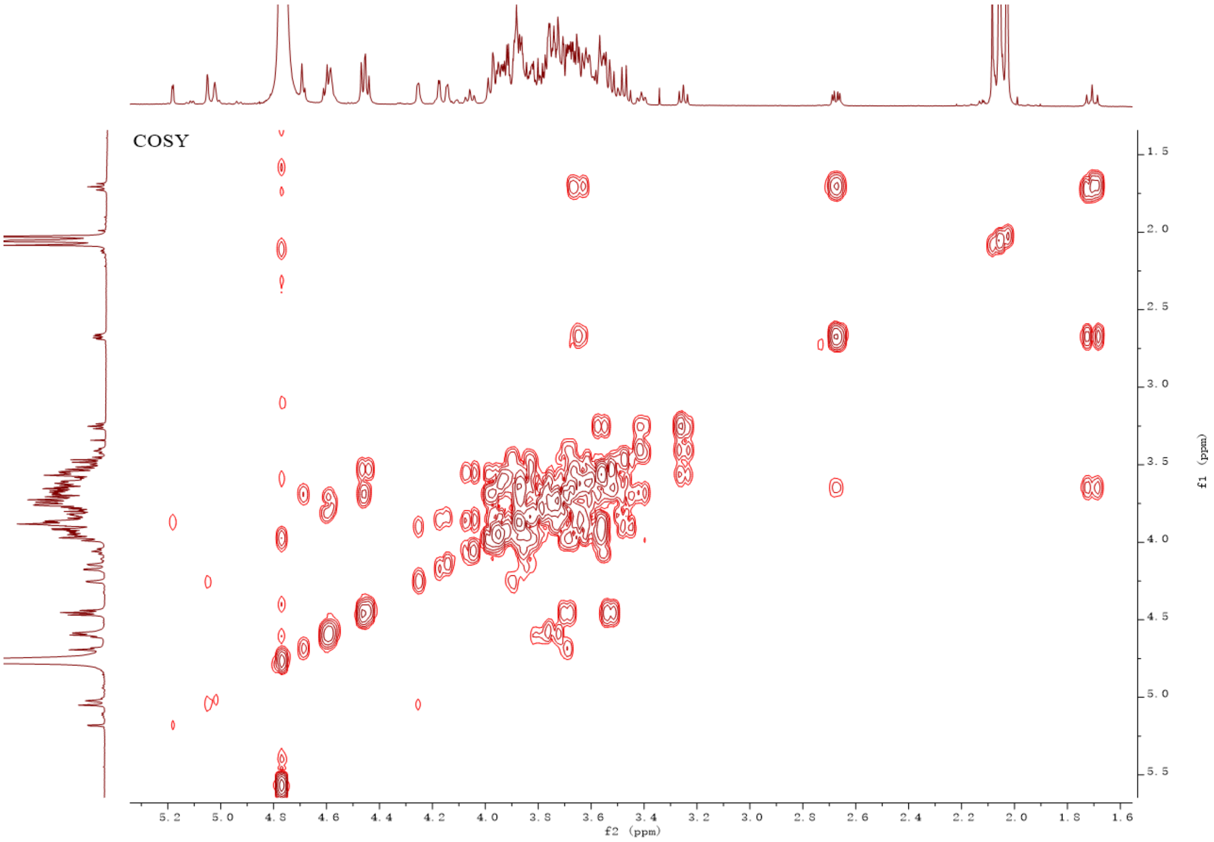

COSY
f2 (ppm)
f1 (ppm)

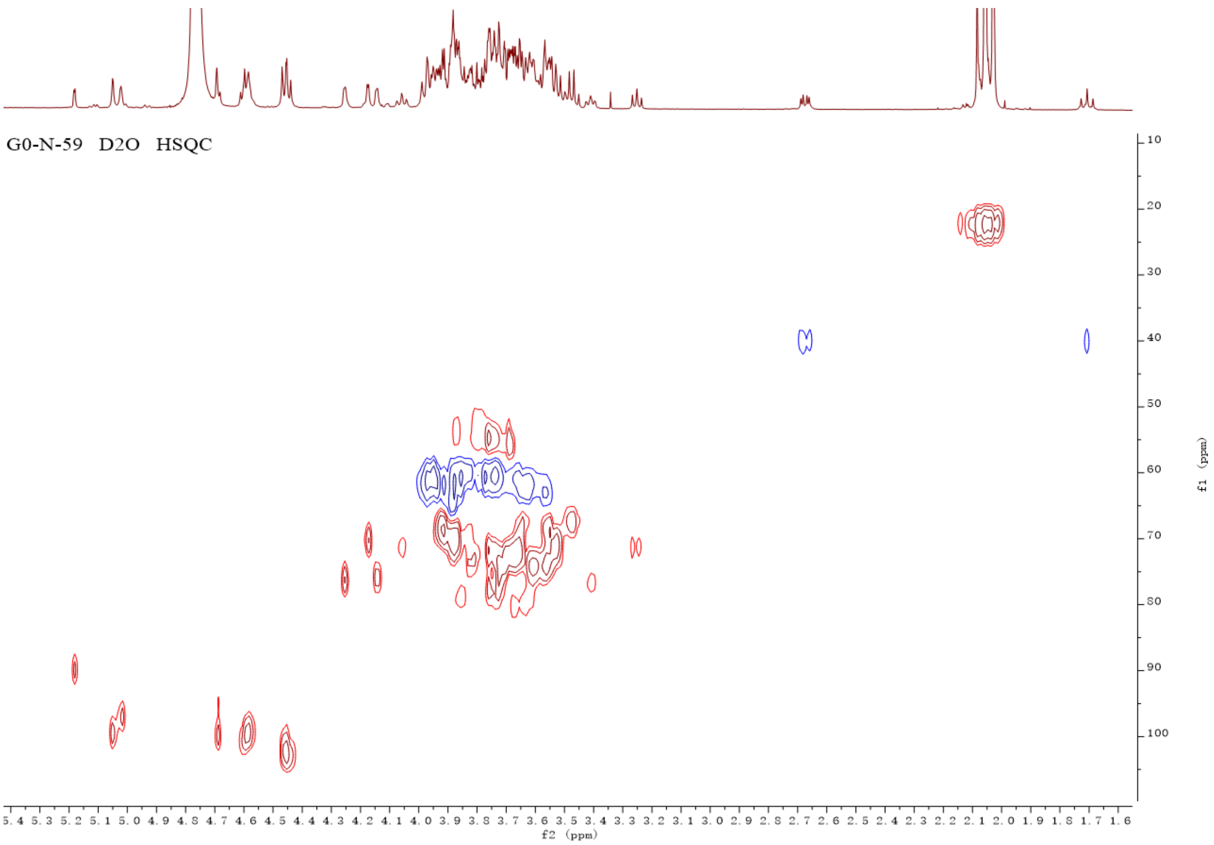
G0-N-59 D2O HSQC
f2 (ppm)
f1 (ppm)

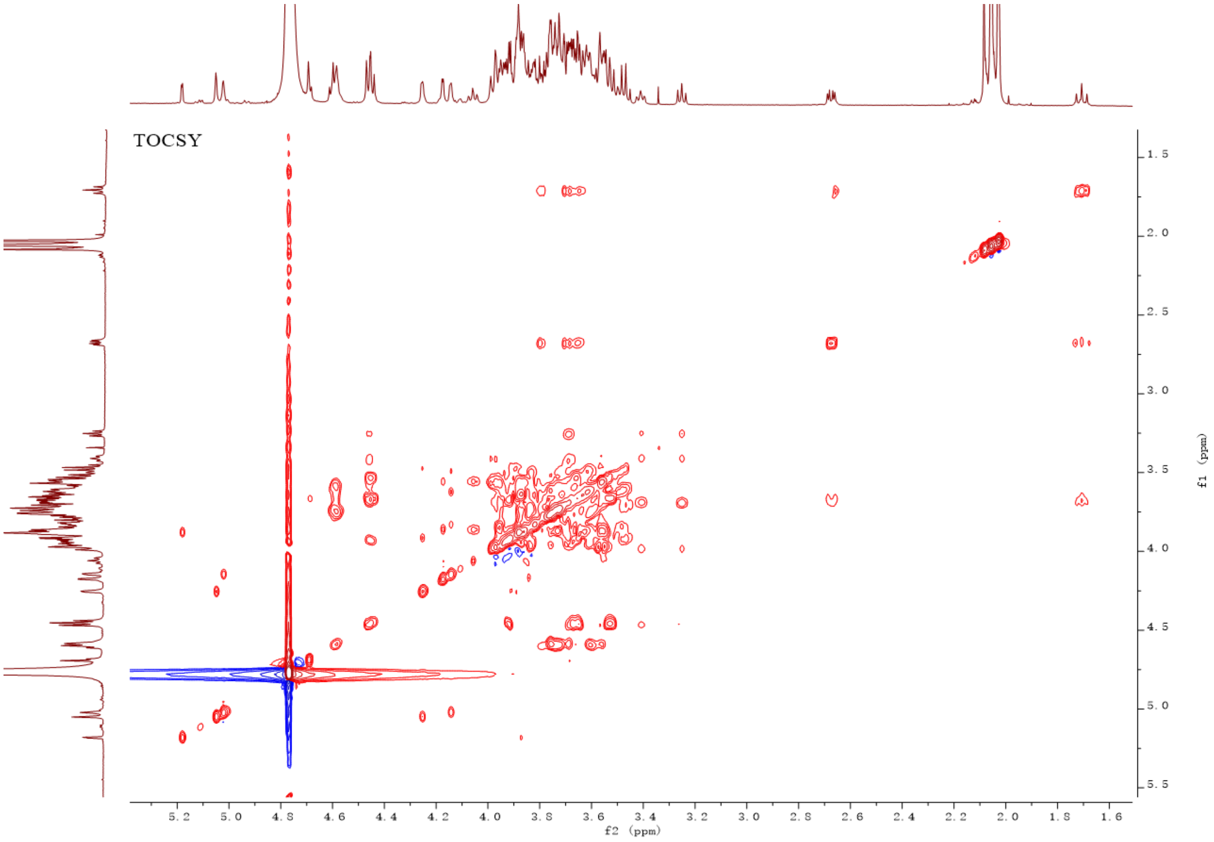
TOCSY
f2 (ppm)
f1 (ppm)

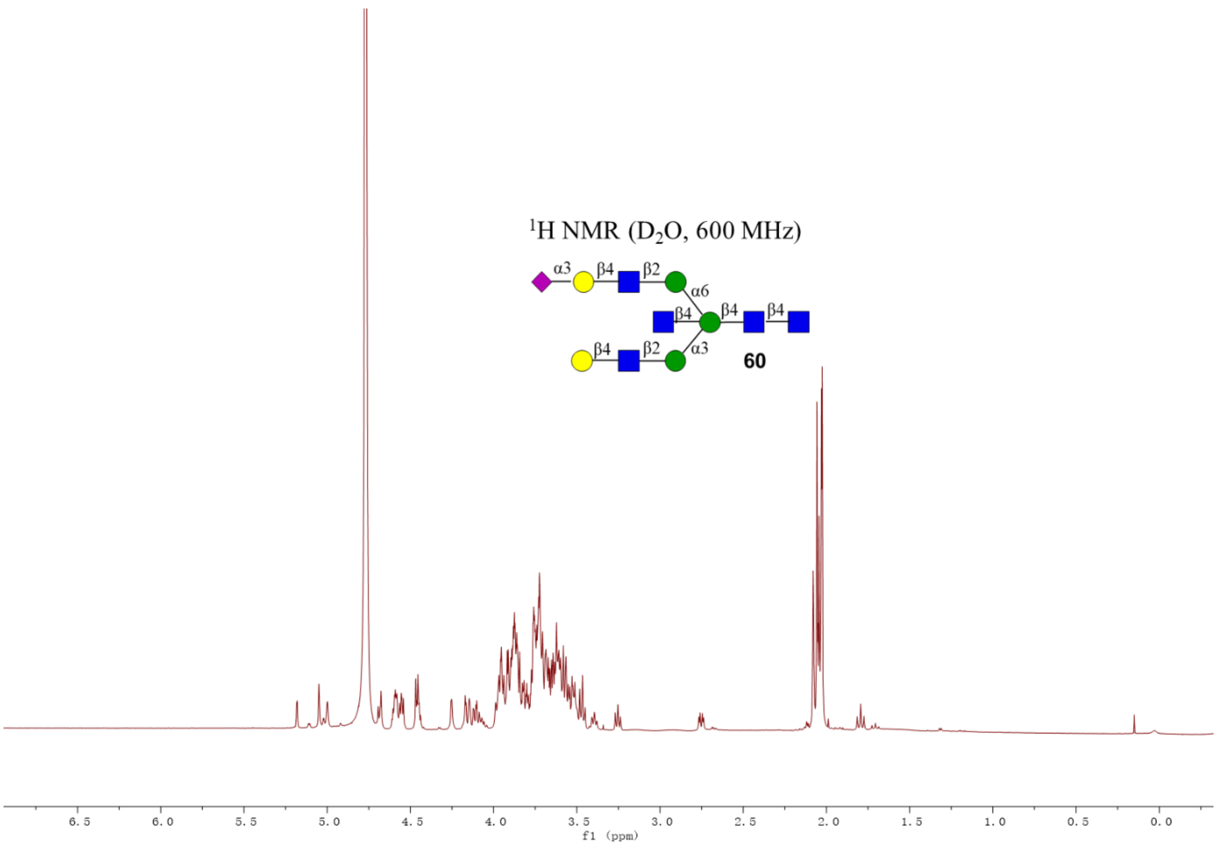
^{1}H NMR (D_2O, 600 MHz)
α3
β4
β2
α6
β4
β4
β4
α3
β4
β2
60
f1 (ppm)

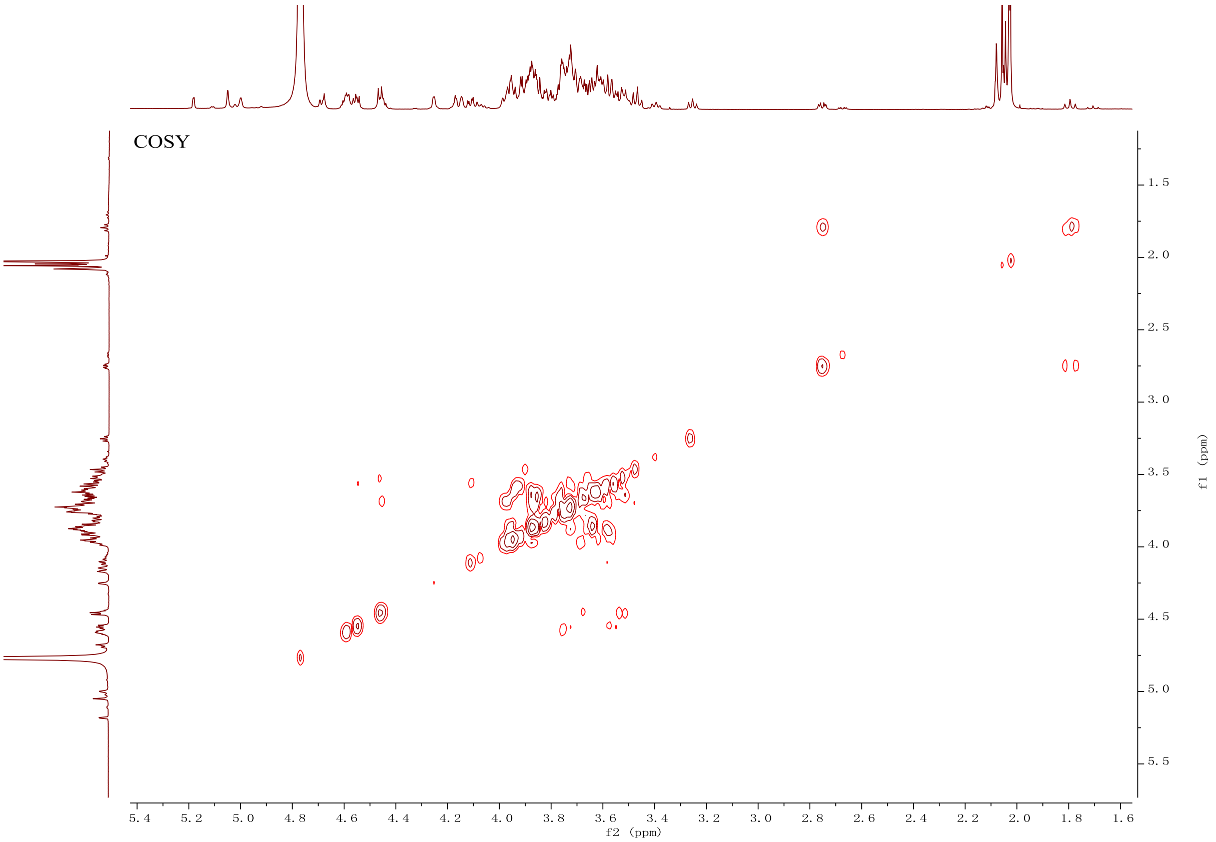
COSY
f2 (ppm)
f1 (ppm)

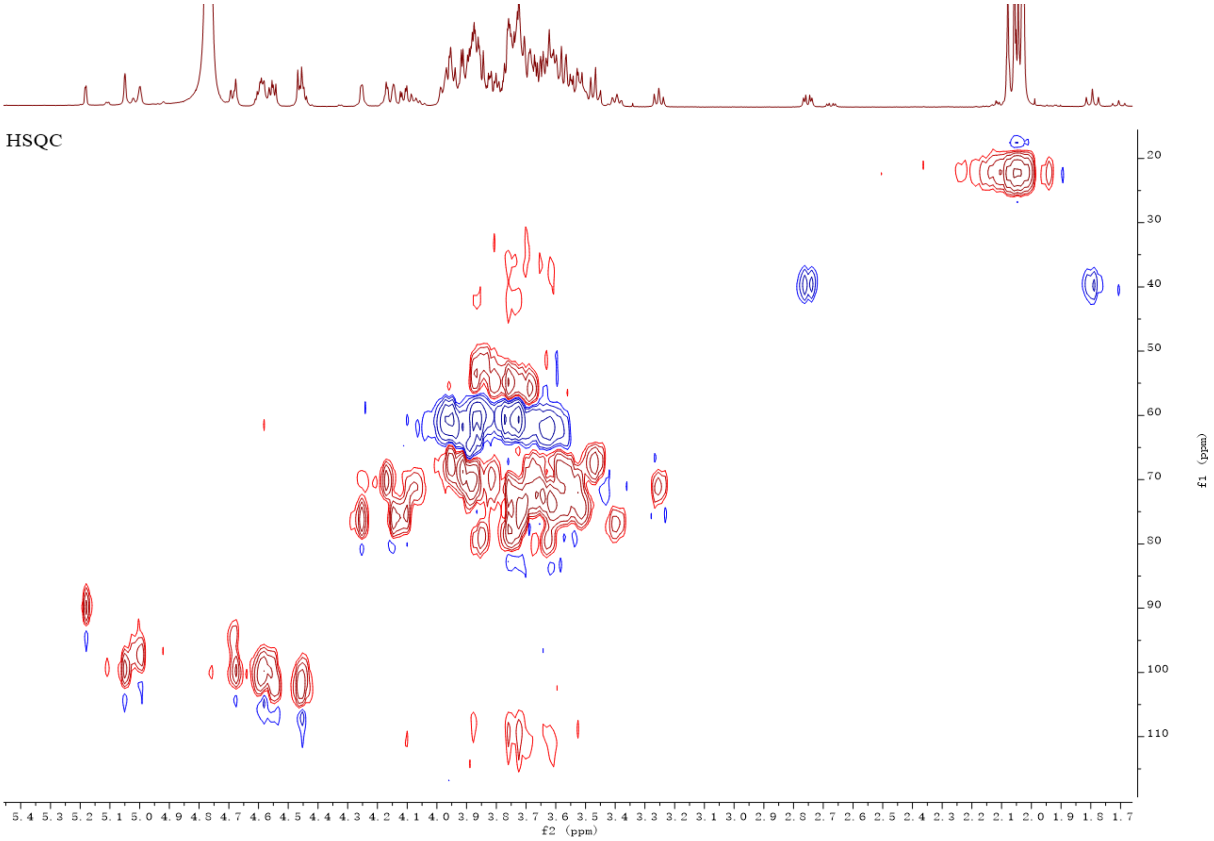
HSQC
f1 (ppm)
f2 (ppm)

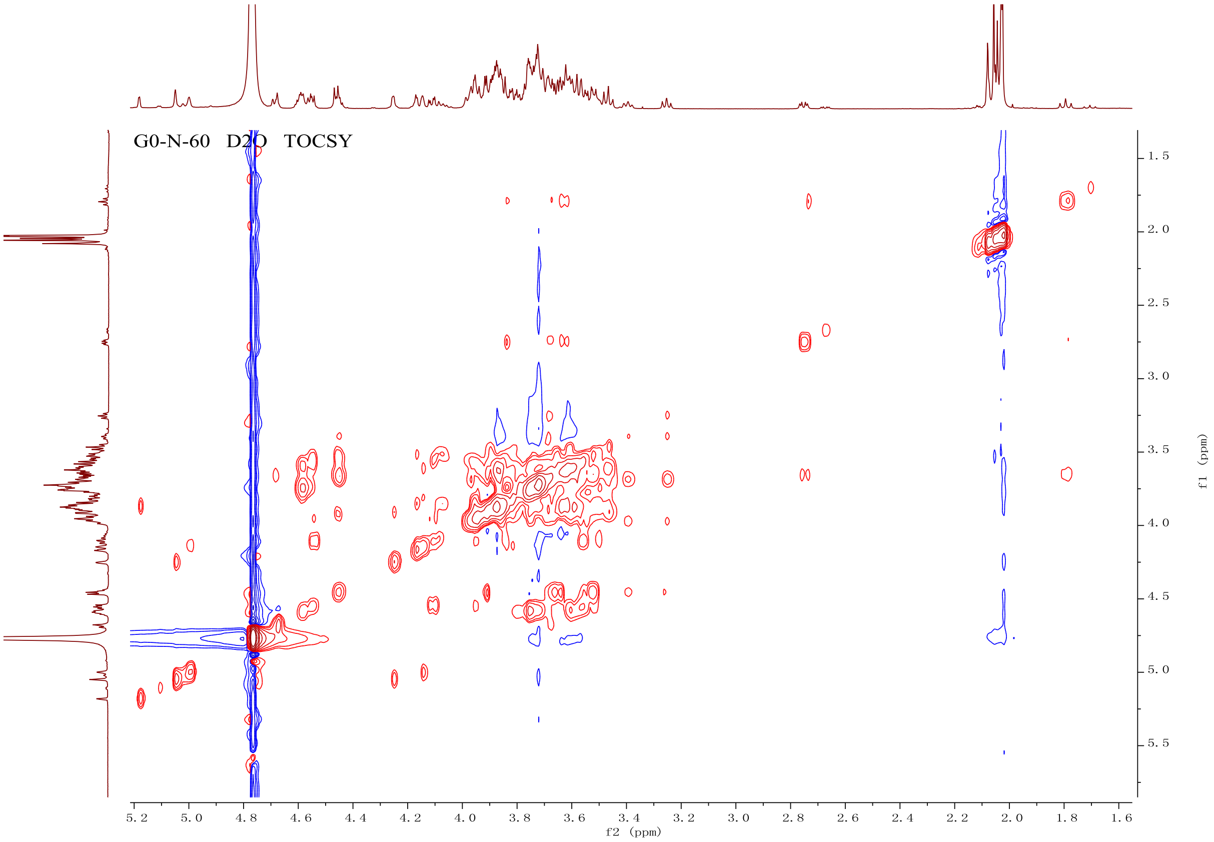
G0-N-60 D2O TOCSY
f1 (ppm)
f2 (ppm)

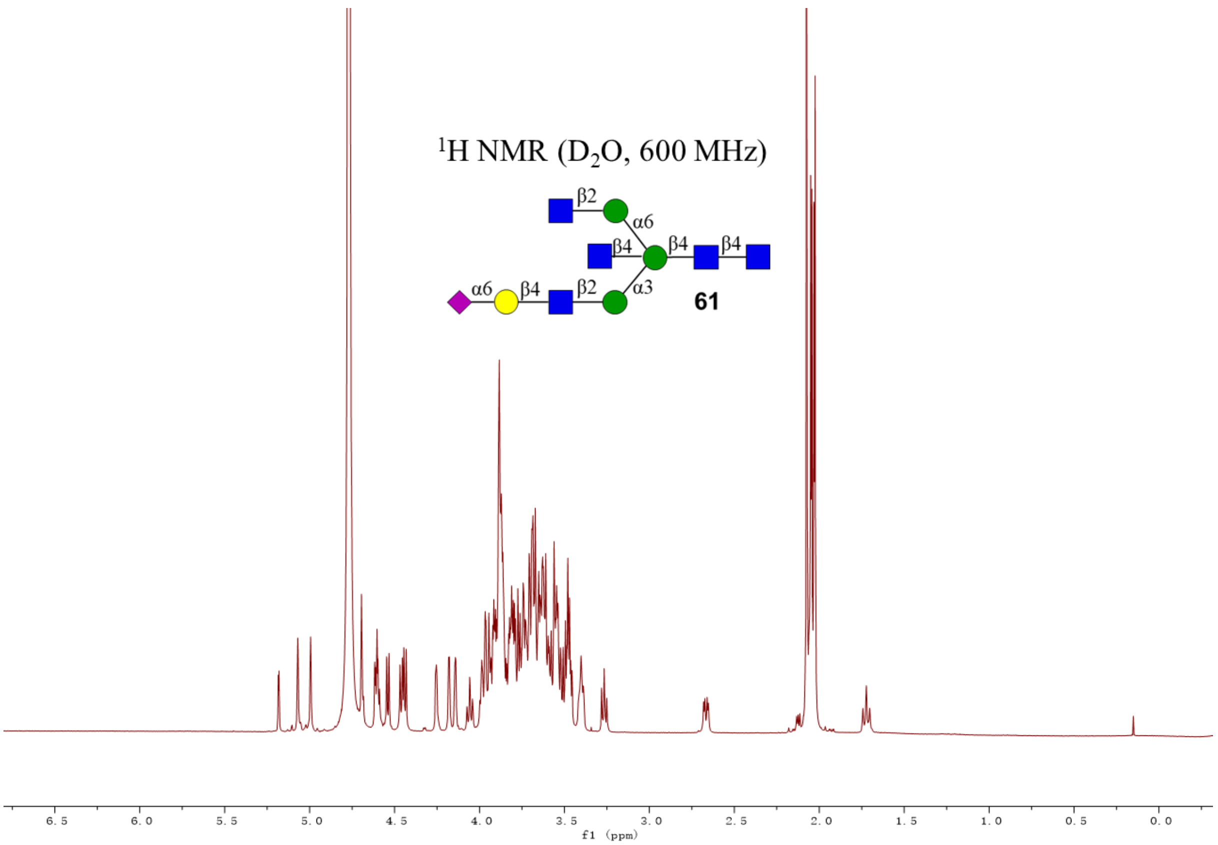
¹H NMR (D₂O, 600 MHz)
β2
α6
β4
β4
β4
α6
β4
β2
α3
61
6.5 6.0 5.5 5.0 4.5 4.0 3.5 3.0 2.5 2.0 1.5 1.0 0.5 0.0
f1 (ppm)

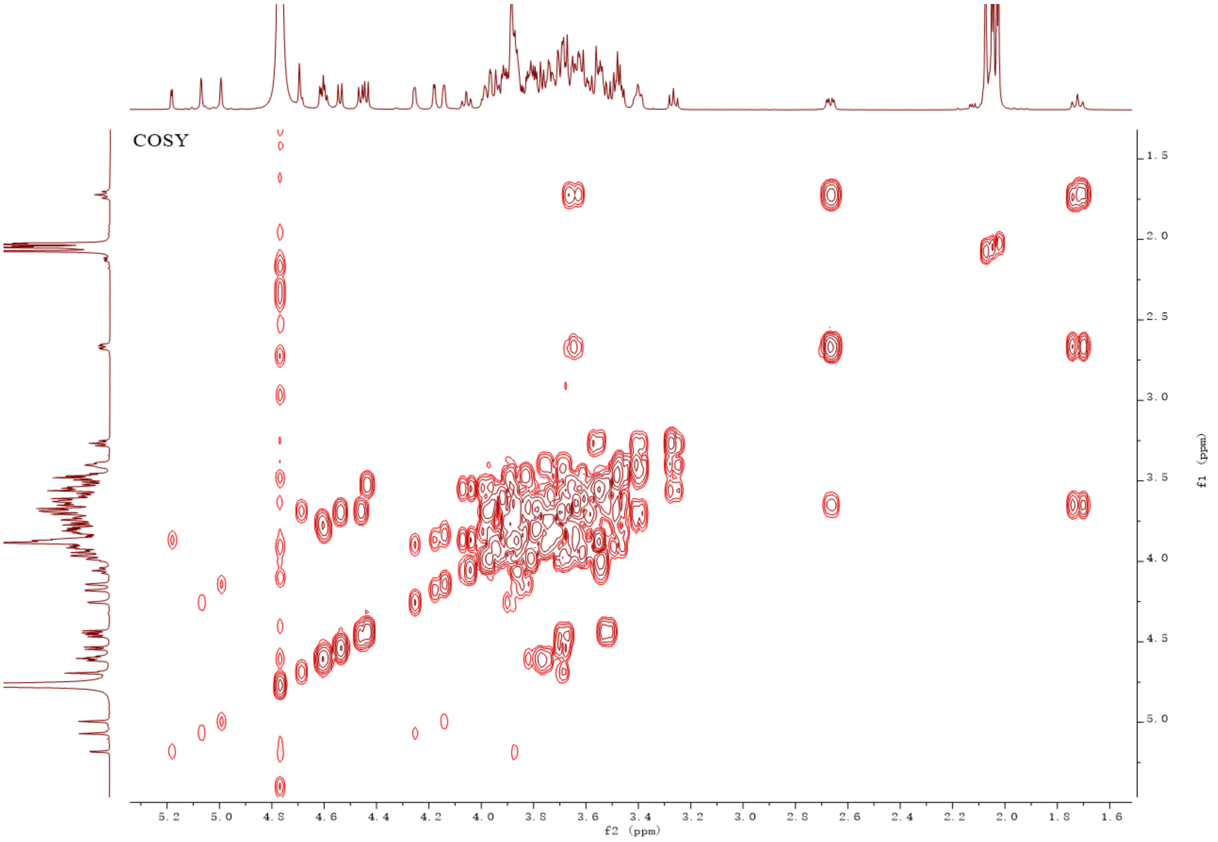
COSY
5.2 5.0 4.8 4.6 4.4 4.2 4.0 3.8 3.6 3.4 3.2 3.0 2.8 2.6 2.4 2.2 2.0 1.8 1.6
f2 (ppm)
1.5 2.0 2.5 3.0 3.5 4.0 4.5 5.0
f1 (ppm)

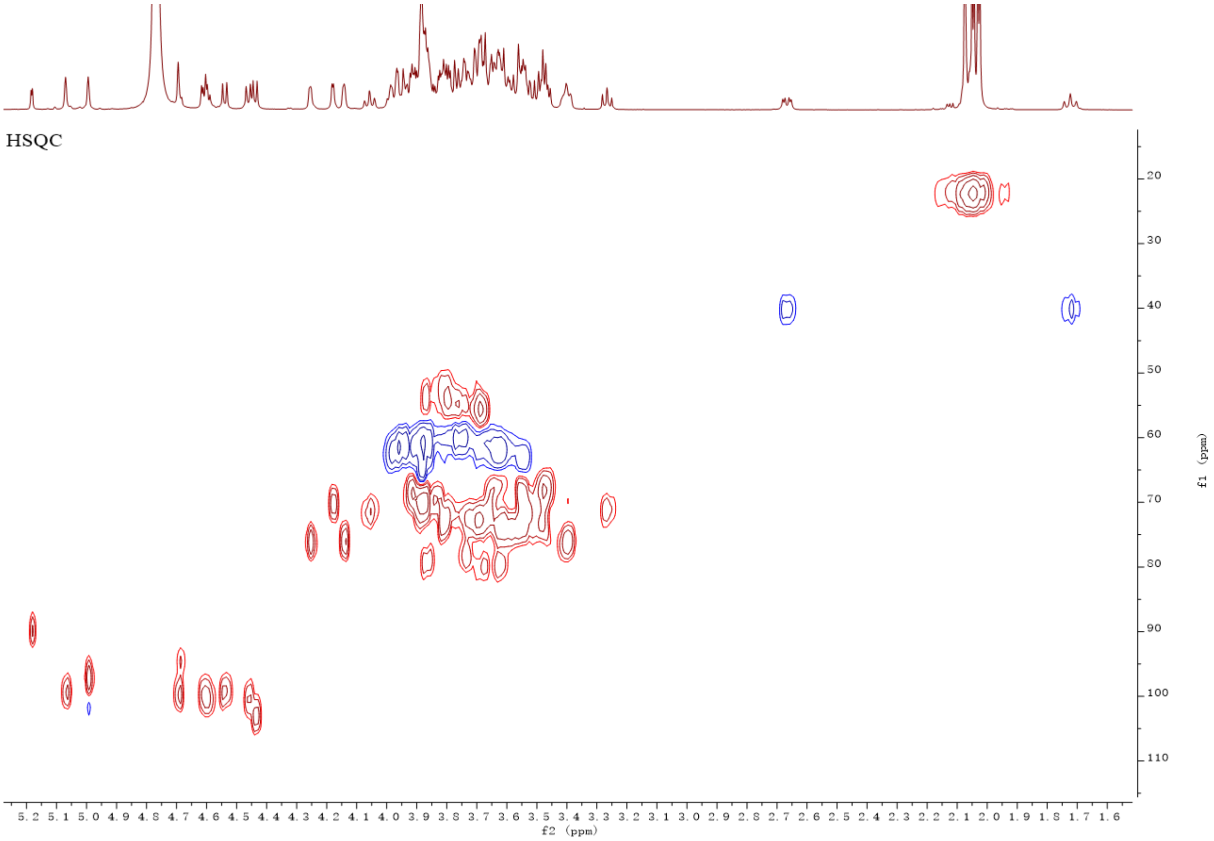
HSQC
f2 (ppm)
f1 (ppm)

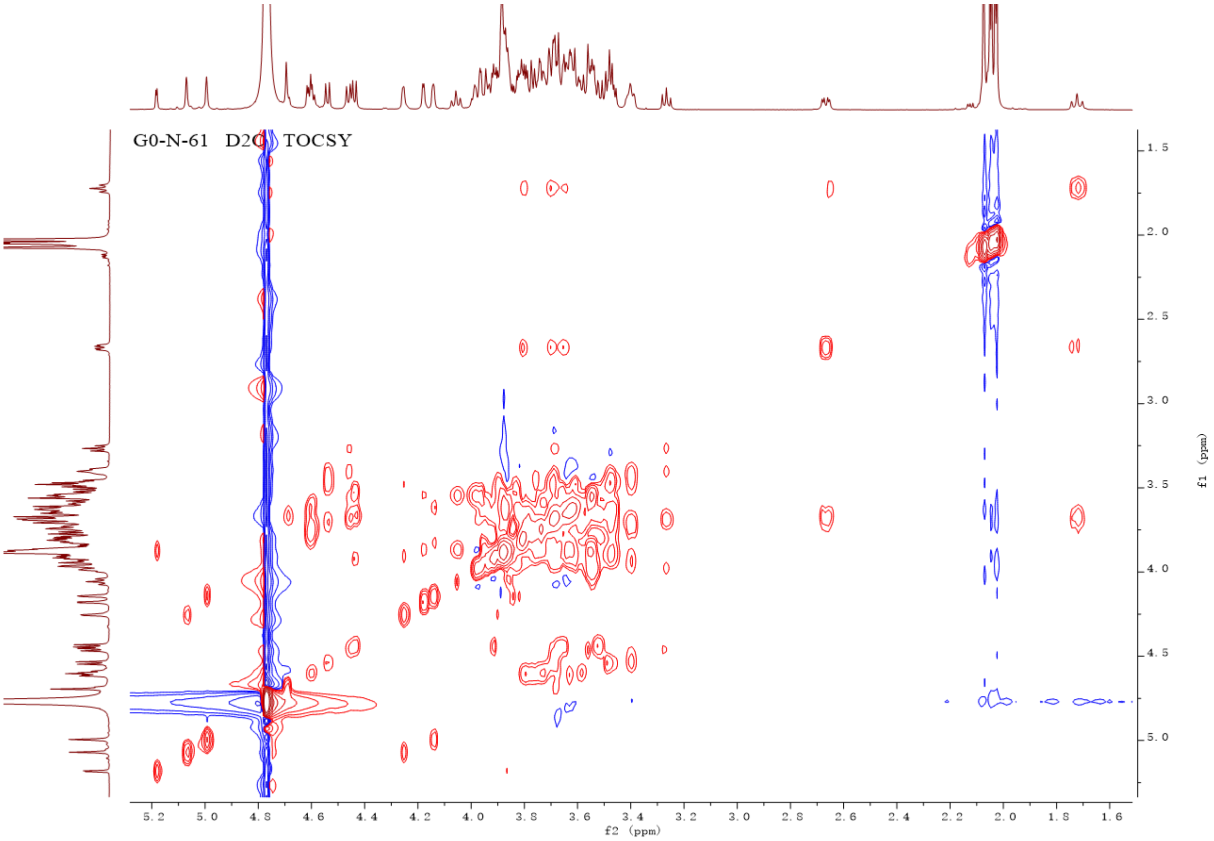
G0-N-61 D2O TOCSY
f2 (ppm)
f1 (ppm)

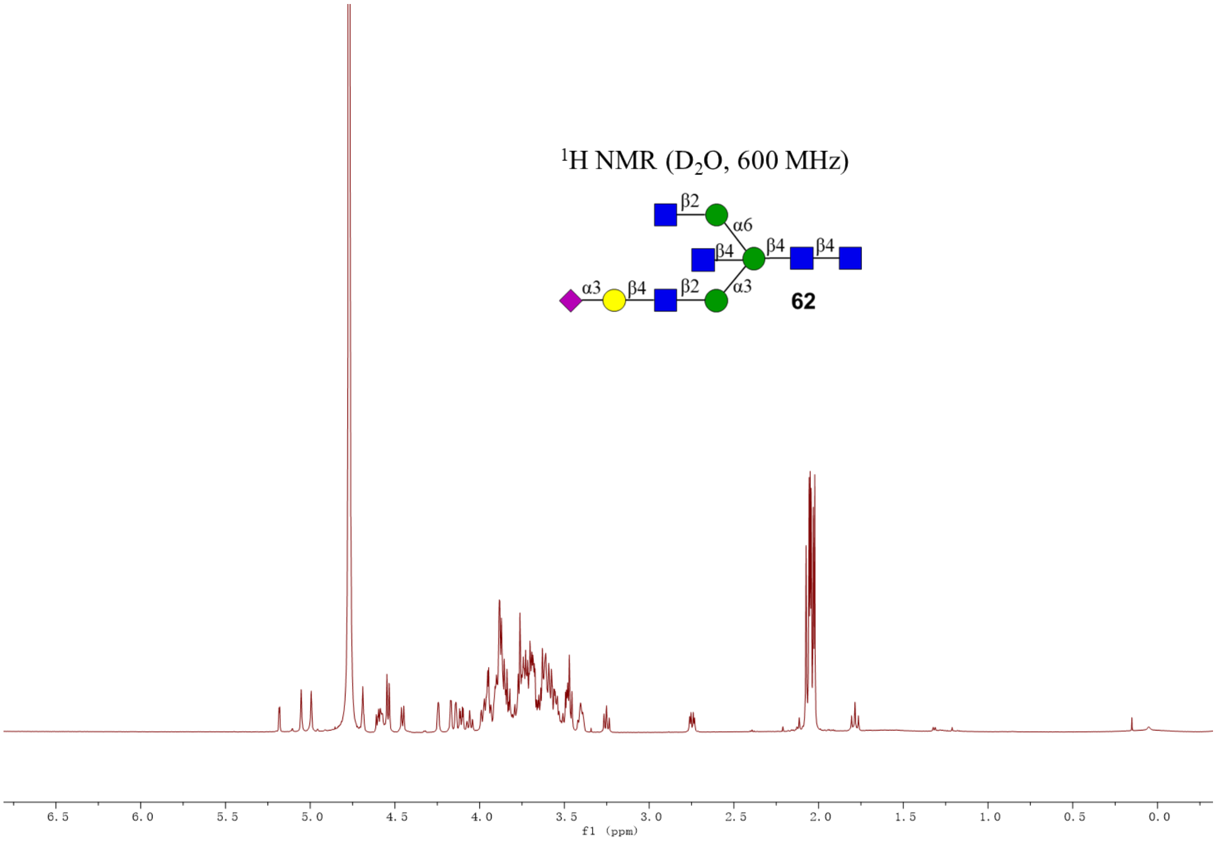

^{1}H NMR (D_2O, 600 MHz)
β2
α6
β4
β4
β4
α3
β4
β2
α3
62
f1 (ppm)

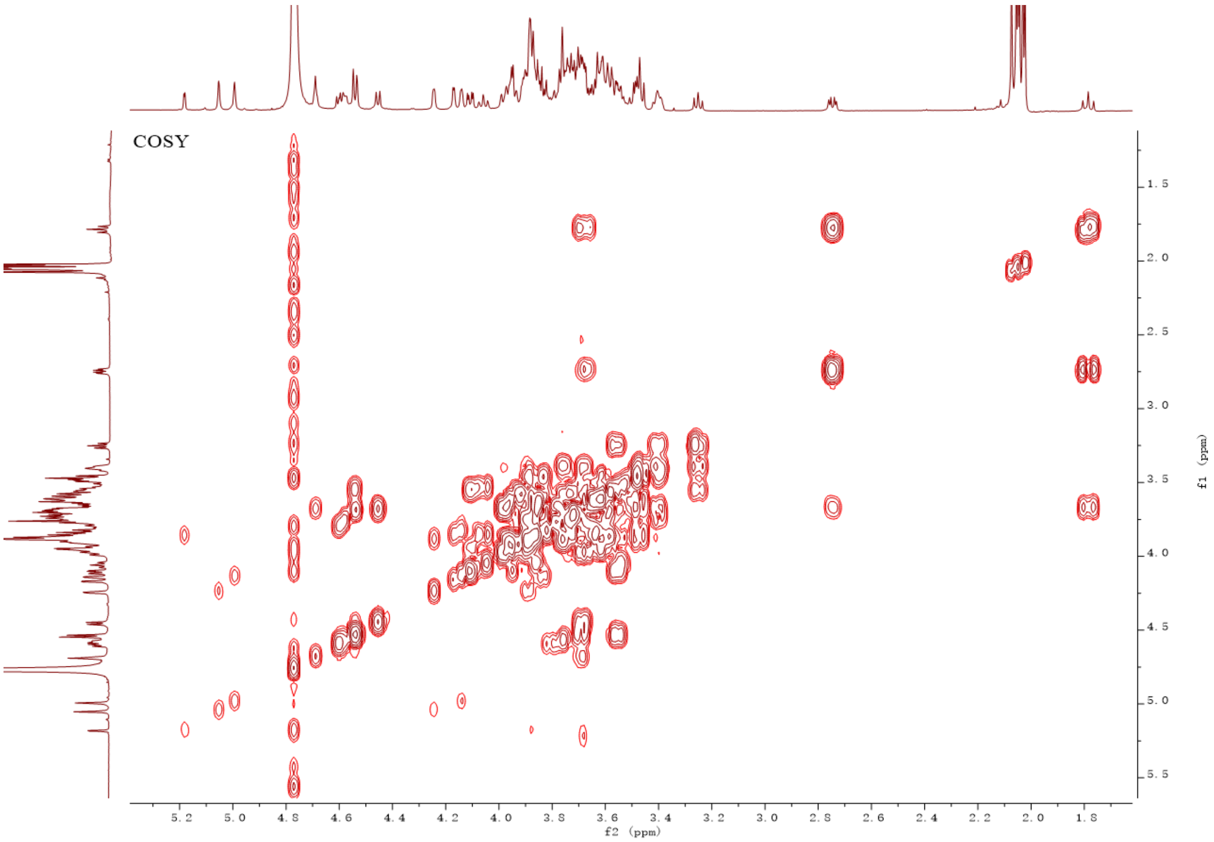

COSY
f2 (ppm)
f1 (ppm)

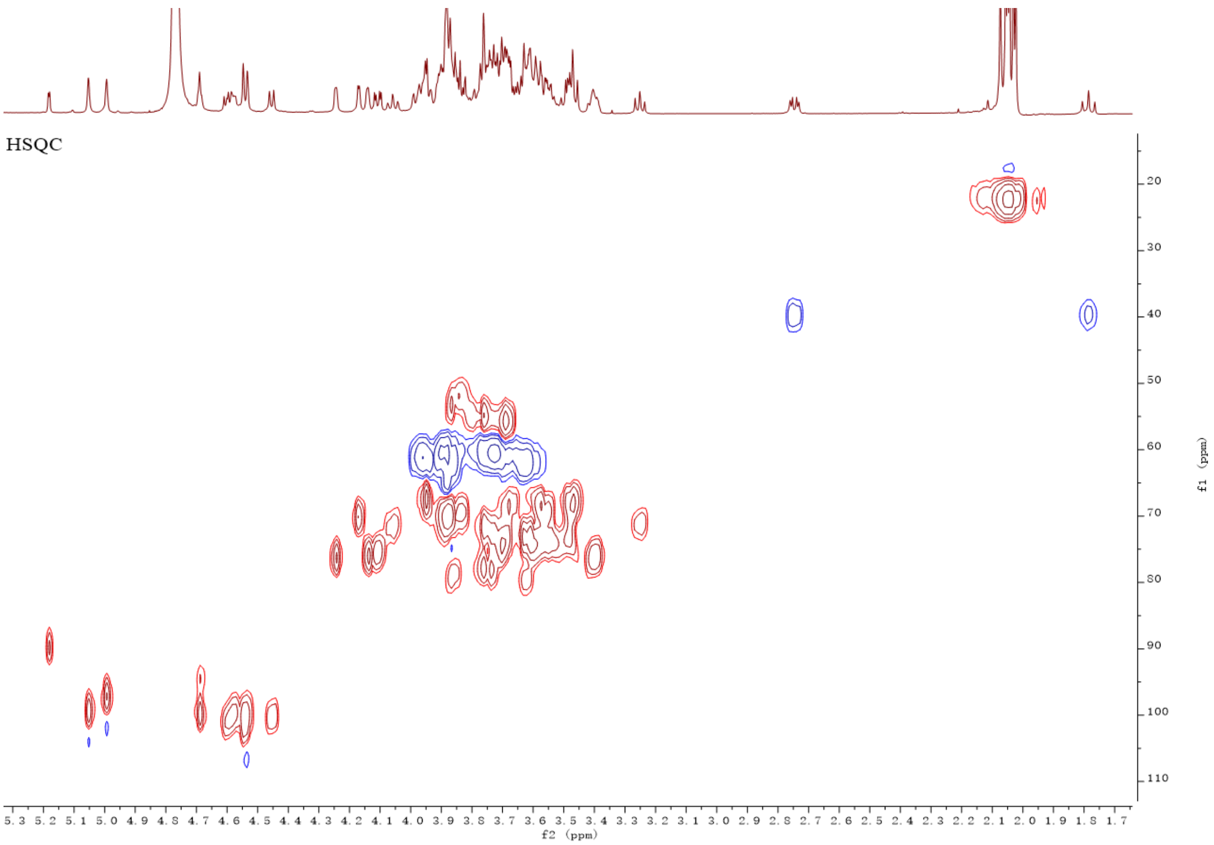
HSQC
f2 (ppm)
f1 (ppm)

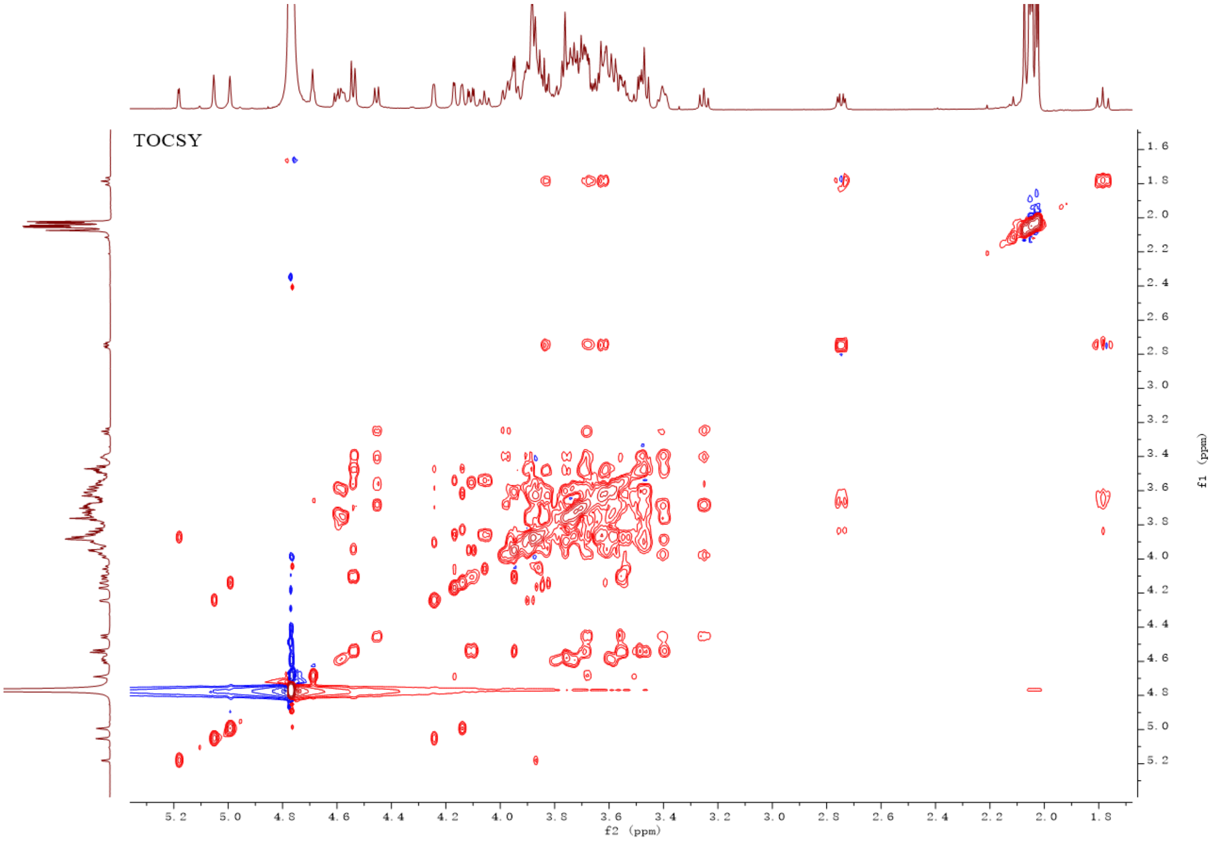
TOCSY
f2 (ppm)
f1 (ppm)

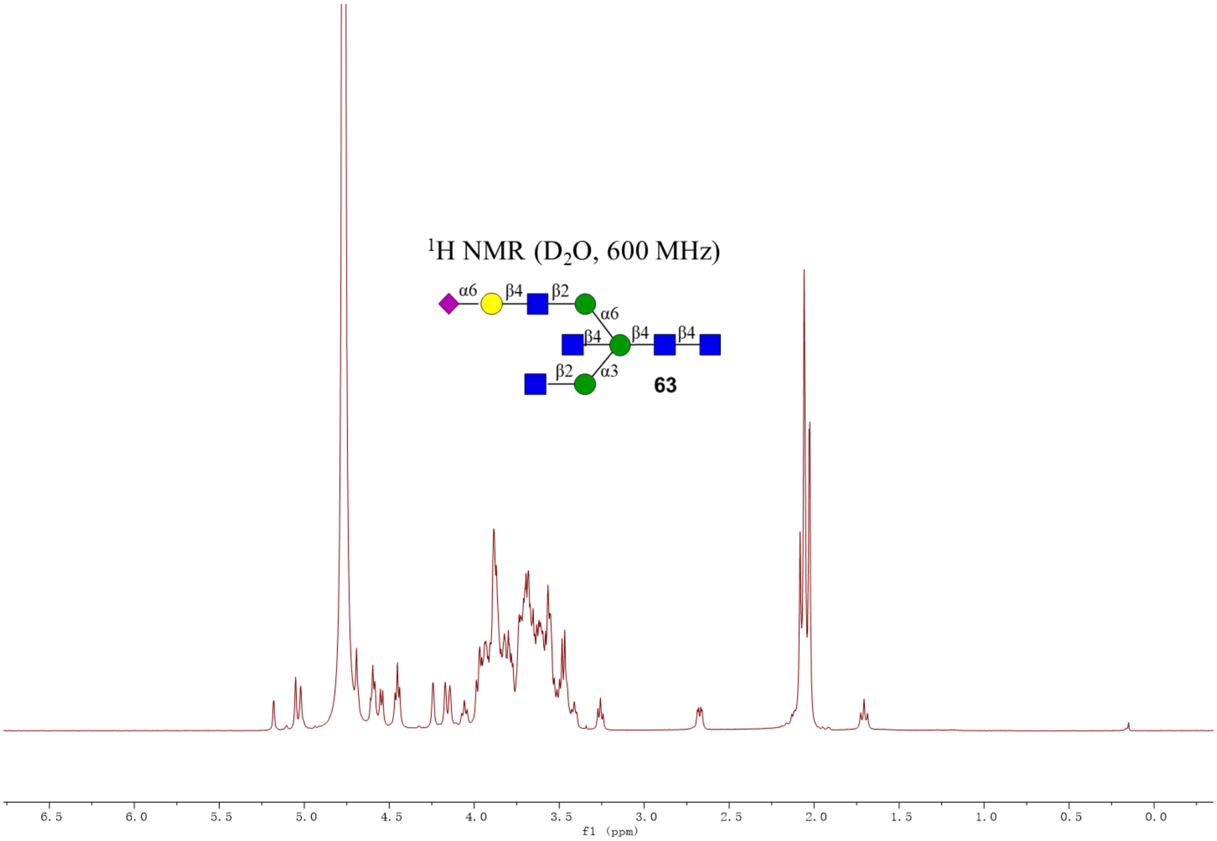

^{1}H NMR (D_2O, 600 MHz)
α6
β4
β2
α6
β4
β4
β4
β2
α3
63
6.5 6.0 5.5 5.0 4.5 4.0 3.5 3.0 2.5 2.0 1.5 1.0 0.5 0.0
f1 (ppm)

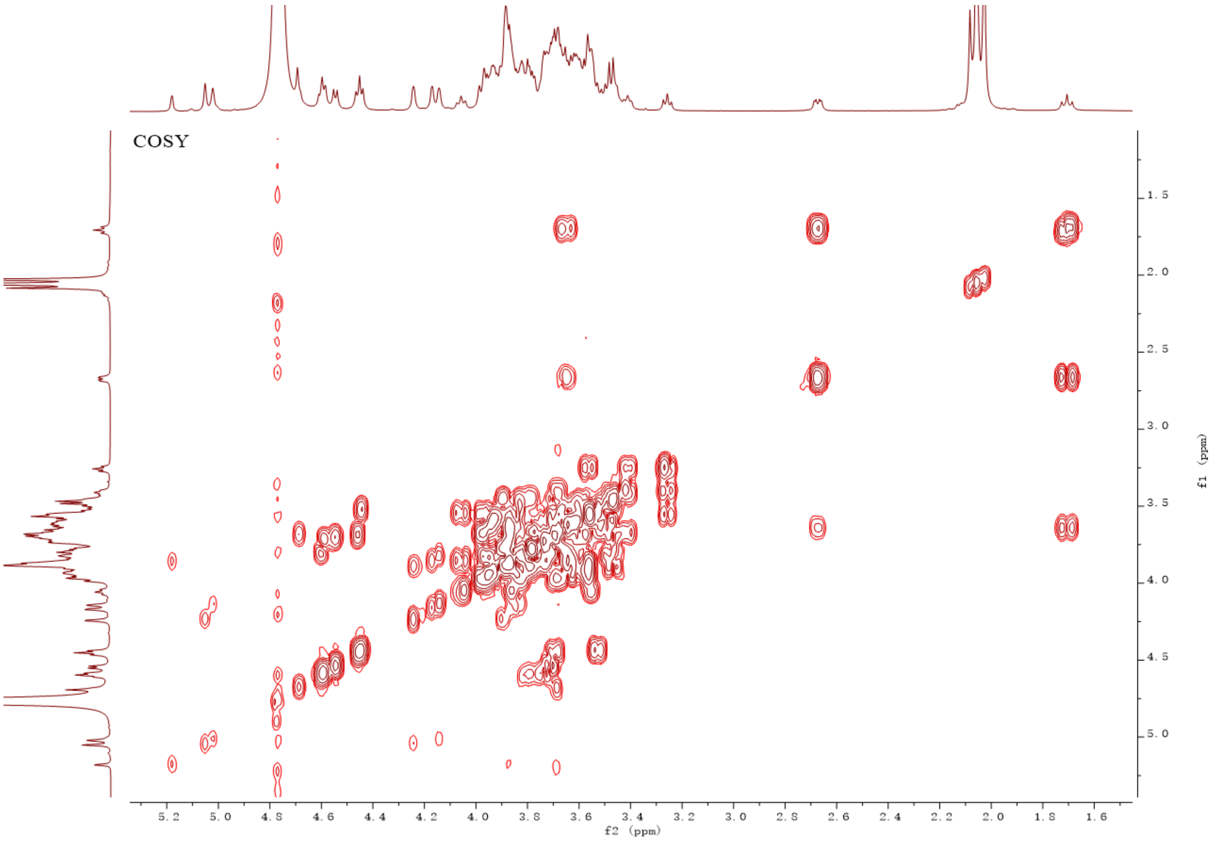

COSY
5.2 5.0 4.8 4.6 4.4 4.2 4.0 3.8 3.6 3.4 3.2 3.0 2.8 2.6 2.4 2.2 2.0 1.8 1.6
f2 (ppm)
1.5 2.0 2.5 3.0 3.5 4.0 4.5 5.0
f1 (ppm)

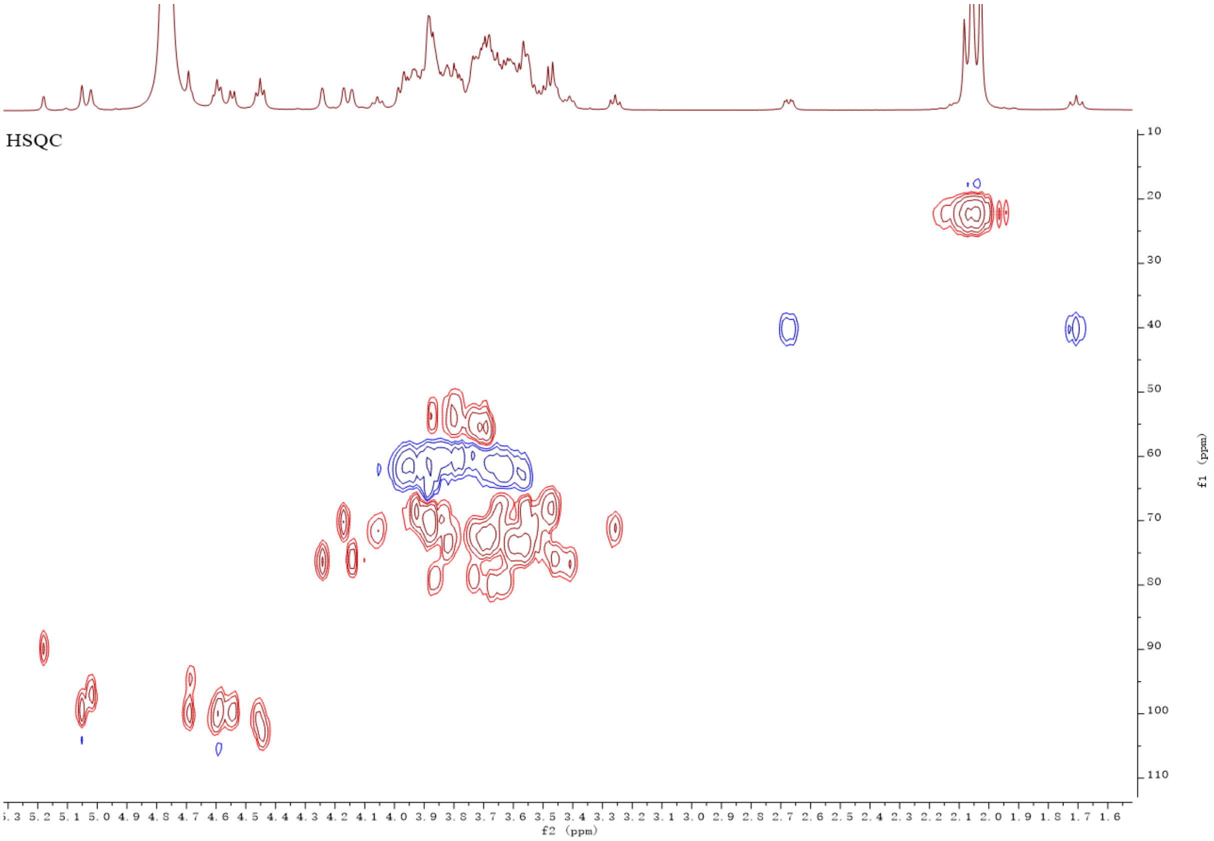
HSQC
f2 (ppm)
f1 (ppm)

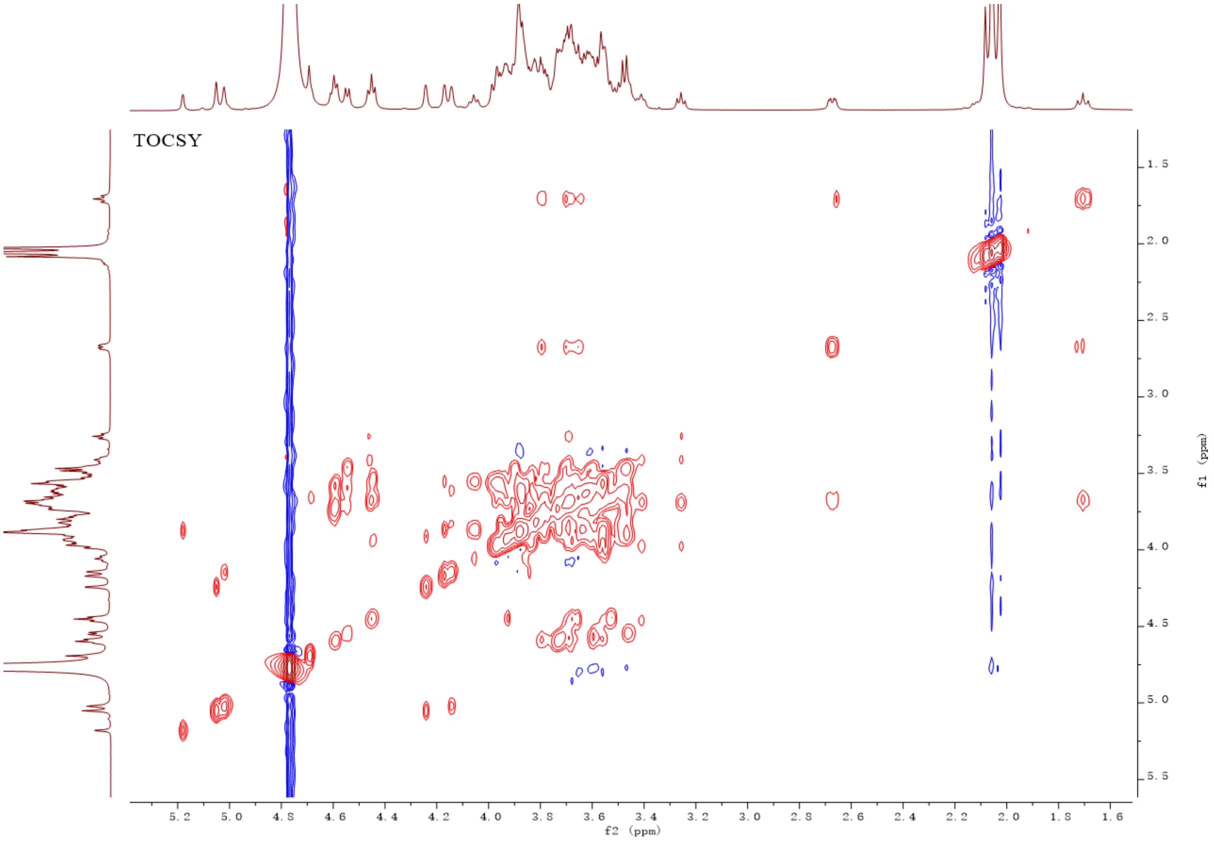
TOCSY
f2 (ppm)
f1 (ppm)

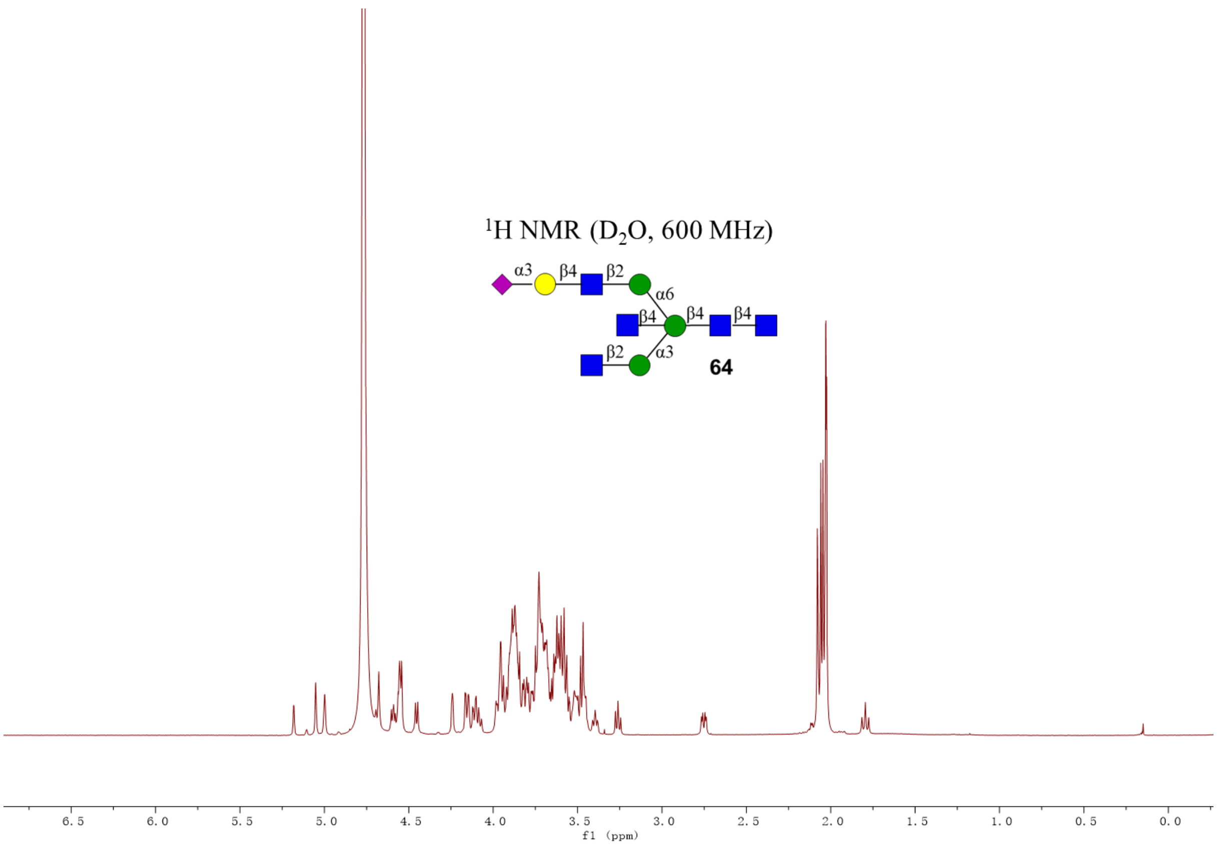
^{1}H NMR (D_2O, 600 MHz)
α3
β4
β2
α6
β4
β4
β4
β2
α3
64
f1 (ppm)

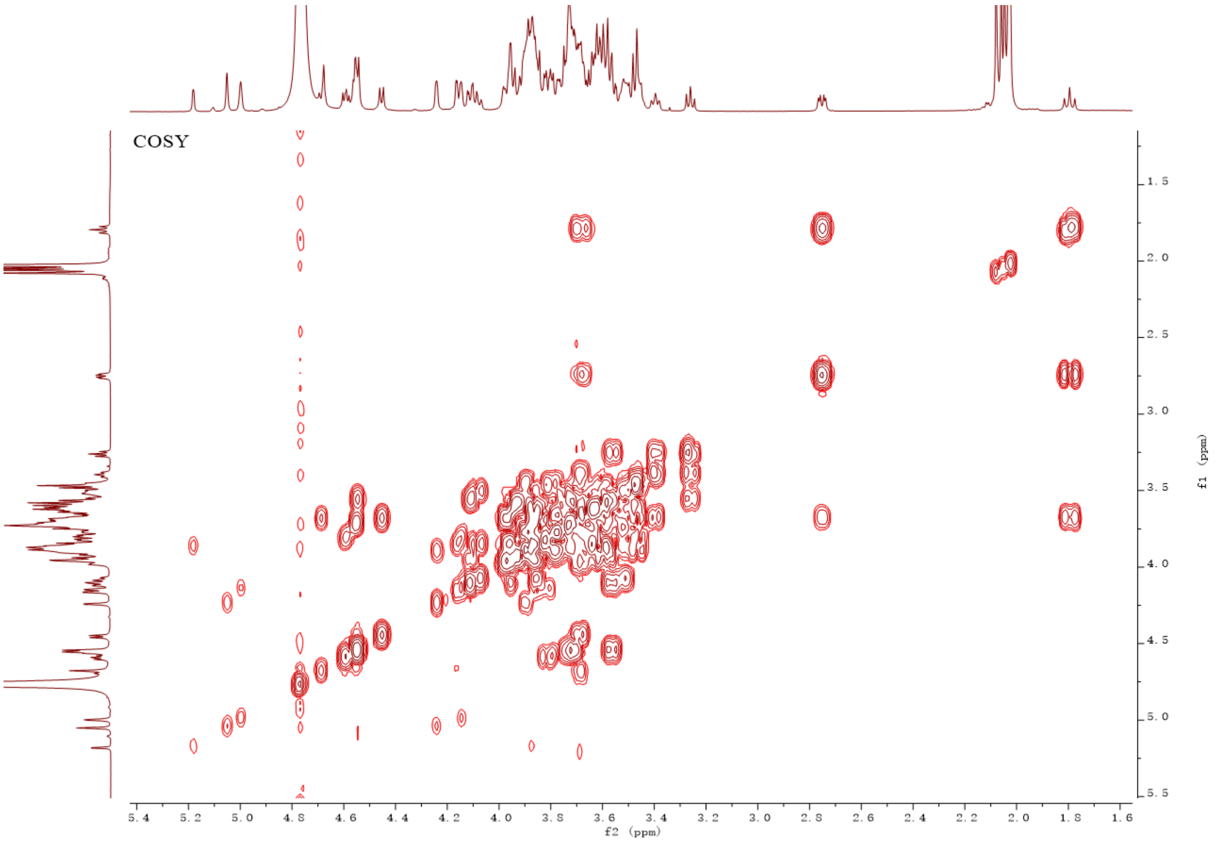
COSY
f2 (ppm)
f1 (ppm)

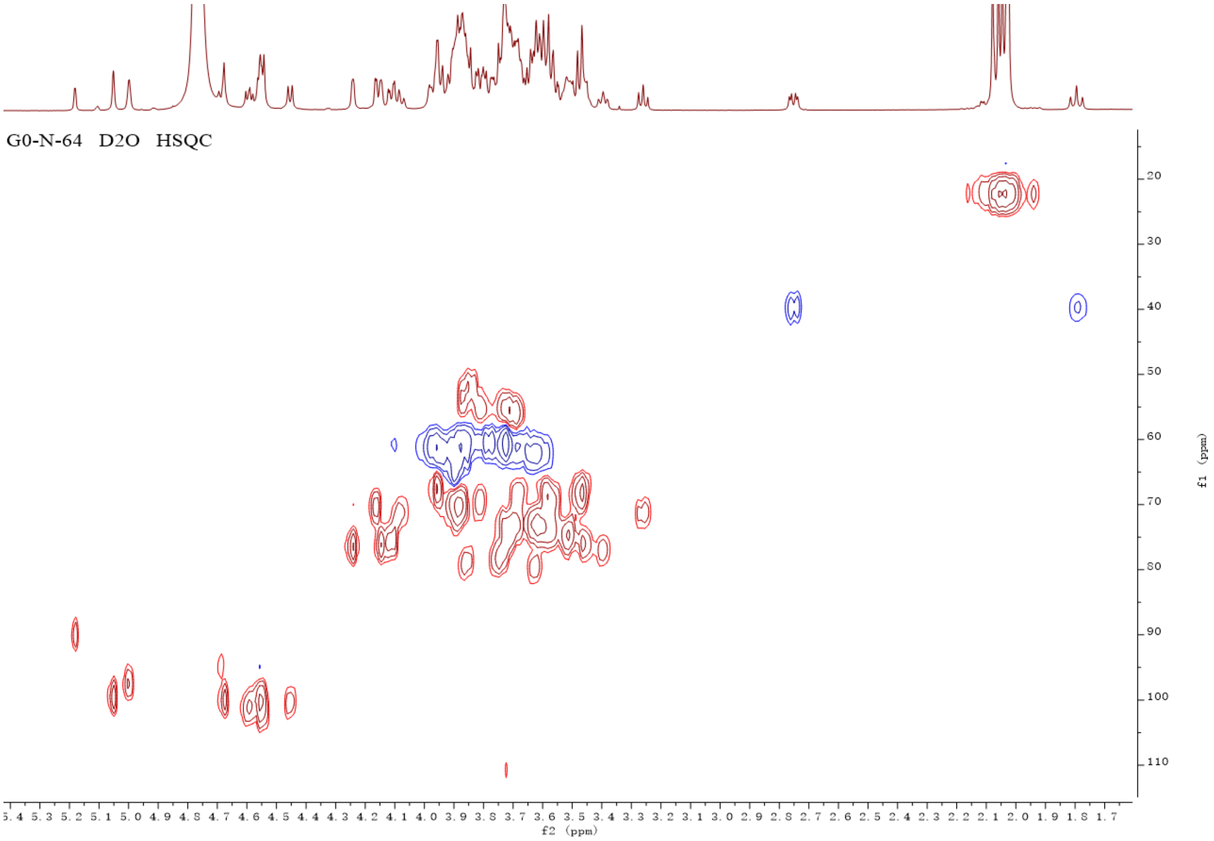
G0-N-64 D2O HSQC
f2 (ppm)
f1 (ppm)

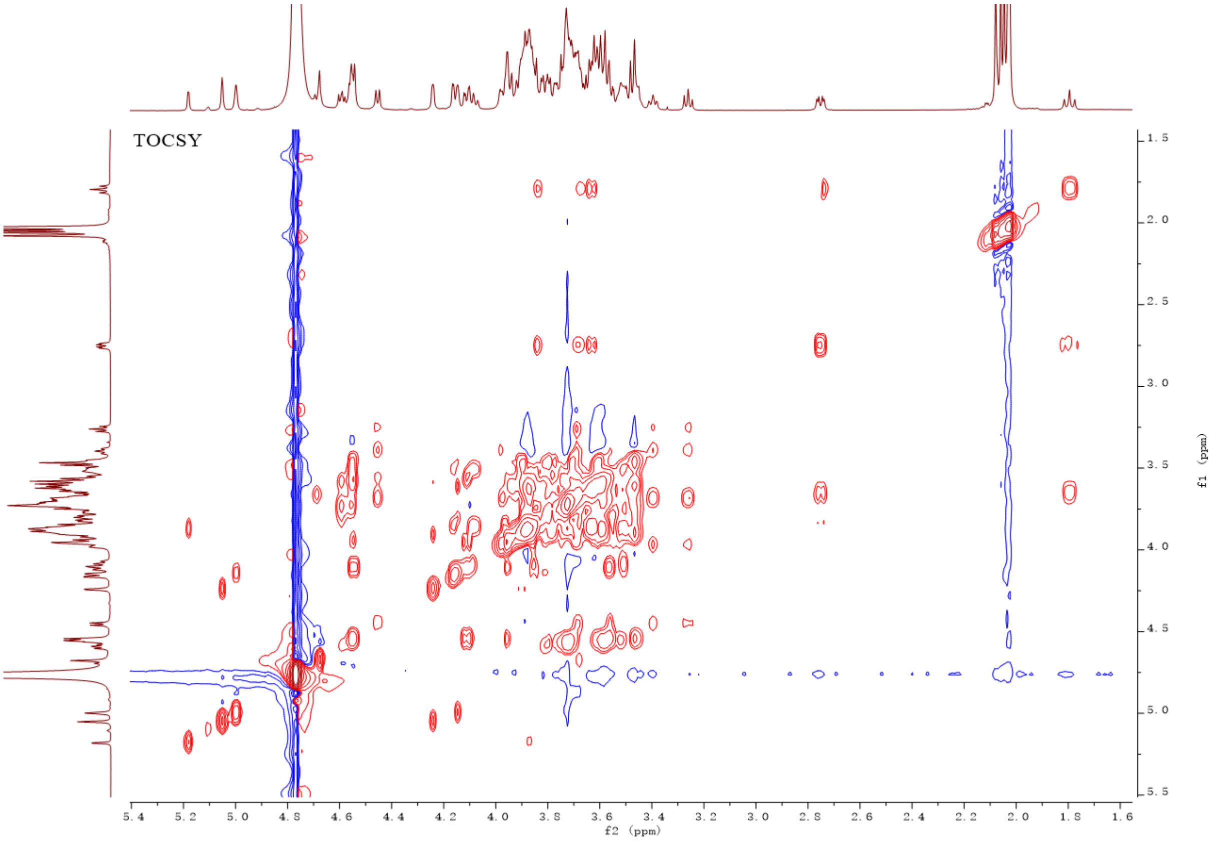
TOCSY
f2 (ppm)
f1 (ppm)
